# Vicinal Stereocenter Construction via α‑Boryl Carbanions from Borylated Cyclopropanes

**DOI:** 10.1021/jacs.5c12433

**Published:** 2025-10-28

**Authors:** Tereza Pavlickova, Noam Orbach, Alexander Kaushansky, Ilan Marek

**Affiliations:** Schulich Faculty of Chemistry and the Resnick Sustainability Center for Catalysis, 26747 Technion−Israel Institute of Technology, Haifa 3200009, Israel

## Abstract

We report generation and stereo­controlled electrophilic trapping of α-boryl carbanions via the selective anionic ring-opening of stereo­defined iodomethyl cyclopropylboronic esters. The strategy relies on a lithium–iodine exchange to generate borata alkene intermediates, affording vicinal tri- and tetrasubstituted boronic esters with excellent levels of diastereo­selectivity after electrophilic trapping. The stereo­control arises from conformational preferences of the boron alkylidene, driven by its unique electronic structure. This work expands the synthetic utility of α-boryl carbanions and demonstrates their potential in the stereo­controlled construction of sp^3^-rich organoboron frameworks.

## Graphical abstract

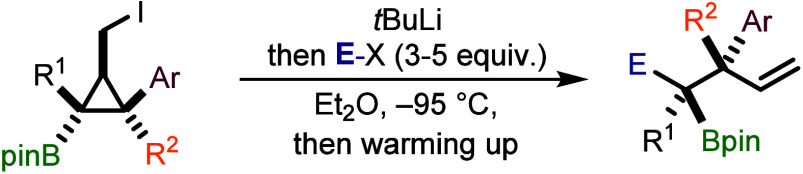

Organoboron compounds are highly valuable synthetic intermediates due to their versatile functionalization reactions such as Suzuki–Miyaura cross-coupling, oxidation, Matteson homologation, metal-catalyzed enantioselective reactions, and transmetalation into various stereo­defined organometallic species.^1–7^ Due to the wide applications of organoboron compounds, the synthesis of sp^3^-rich organoboron compounds bearing stereo­defined adjacent stereo­centers remains strategically important.^8–10^ Particularly appealing is the construction of vicinal tri- and tetrasubstituted centers bearing boronic esters within acyclic systems.^11,12^ Our group previously reported the synthesis of acyclic boronic esters bearing tertiary and quaternary centers via the ring-opening of stereo­defined cyclopropyl boronic esters (Scheme 1a).^13–15^ This approach relies on the addition of an organolithium reagent to a cyclopropyl boronic ester containing a suitable leaving group to form a boronate complex. A subsequent 1,2-metalate rearrangement takes place along with ring-opening^16^ to furnish tertiary pinacol­boranes possessing the desired adjacent stereo­centers with excellent diastereo­selectivity. Inspired by this work, and recognizing the significance of these stereo­defined molecular frameworks, we wondered whether they could be accessed through an entirely different approach—namely, the diastereo­selective reaction of α-boryl carbanions with electrophiles (Scheme 1b).

**Scheme 1:**
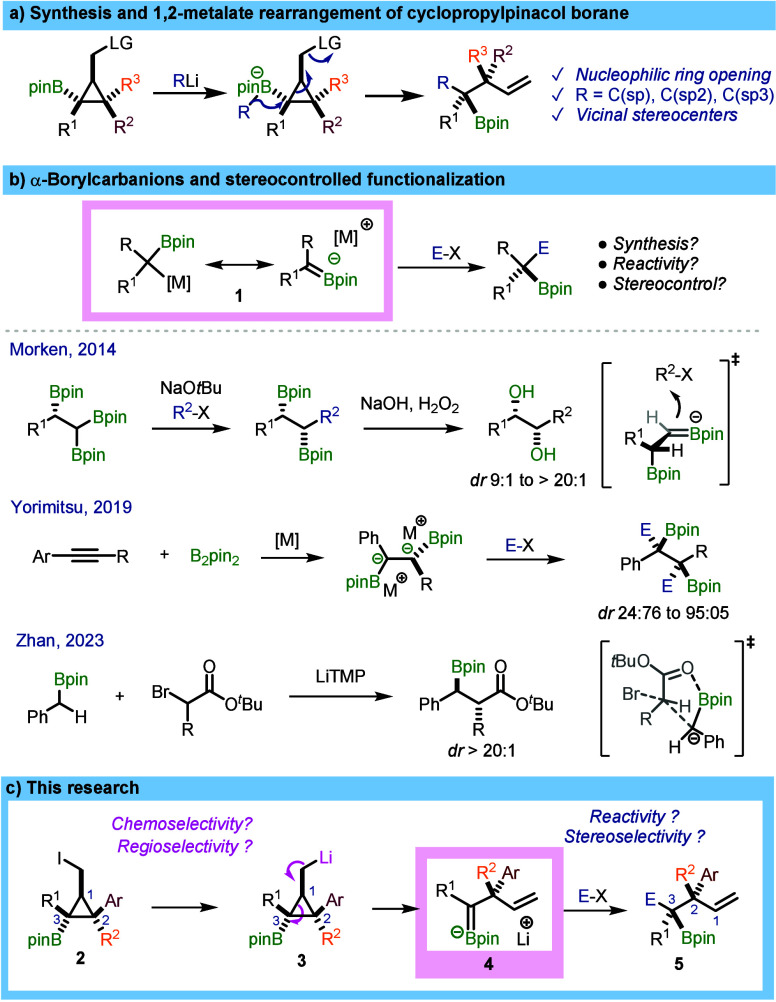
State of the Art and Proposed Transformation

α-Boryl carbanions **1**, also referred to as boron alkylidenes or borata alkenes, are stabilized through electron delocalization toward the boron atom, forming a π_C–B_ bond.^17,18^ In recent years, these intermediates have emerged as versatile synthetic intermediates,^19,20^ enabling a wide range of transformations including cyclization and ring-opening reactions,^21–27^ addition to aldehydes and imines,^28–31^ conjugate addition,^32,33^ allylation,^34^ and net [2+2] cycloaddition.^35^

Despite these significant developments, diastereo­selective intermolecular nucleophilic substitution remains a considerable challenge. Morken reported that deborylation of 1,1,2-tris­(boronates) can generate α-boryl carbanions, which upon alkylation and subsequent oxidation provide 1,2-diols in high yield and with excellent diastereo­selectivity.^36^ This selectivity is attributed to an interaction between the σ_C–B_ and the adjacent π_C–B_ orbitals, which is proposed to stabilize a specific conformer of the carbanion, guiding the selective electrophile trapping. A few years later, Yorimitsu observed various levels of diastereo­selectivity in the formation of acyclic products via double electrophilic trapping of a dianion formed by the diborative reduction of substituted arylacetylenes.^37^ More recently, Zhan reported a diastereo­selective deprotonation–alkylation of benzyl boronate, rationalized by a five-membered-ring chelation model.^38^ Our proposed approach would involve the preparation of borata alkenes **4** from stereo­defined cyclopropyl boronic esters **2** (Scheme 1c). The sequence would begin with a lithium–iodine exchange reaction, triggering a selective ring-opening of resulting the lithiated cyclopropylboronic esters **3** to afford the expected borata alkene intermediates **4**. This intermediate can then be subsequently trapped by electrophiles to furnish acyclic boronic esters bearing vicinal stereo­centers, **5** (Scheme 1c). This approach has the potential to unlock access to challenging classes of densely substituted boronic esters and provides direct access to borata alkenes without requiring additional stabilizing groups. However, several key challenges must be addressed for this approach to succeed:

1. How can the desired lithium–iodine exchange be selectively performed while avoiding the formation of the pinacol boronic ester ate-complex,^14,15^ which would trigger a 1,2-metalate rearrangement, as reported in Scheme 1a?
2. How can the selectivity of the ring-opening of **3** be directed toward the unique cleavage of the C_1_–C_3_ bond rather than the C_1_–C_2_ bond, particularly since the latter would generate a benzylic carbanion?
3. How can the diastereo­selectivity of the reaction with electrophiles be controlled to provide a single diastereomer of **5**?

To tackle these questions, we began by synthesizing a library of cyclopropyl methyl iodides **2** using our previously developed methodology.^15^ This strategy relies on the Cu-catalyzed hydro- and carboborylation of cyclopropenyl esters (see Supporting Information).^13,14^ The resulting esters were reduced to the corresponding alcohols and then converted into mesylates, followed by Finkelstein exchange to yield the desired iodides (Scheme 2). This modular process allows for broad variation in substitution patterns. A diverse set of cyclopropylboronic methyl iodides **2a**–**n** bearing a range of aryl groups, including electron-rich, electron-poor, and heteroaryl substituents, was successfully synthesized with excellent control over both regio- and diastereo­selectivity.

**Scheme 2:**
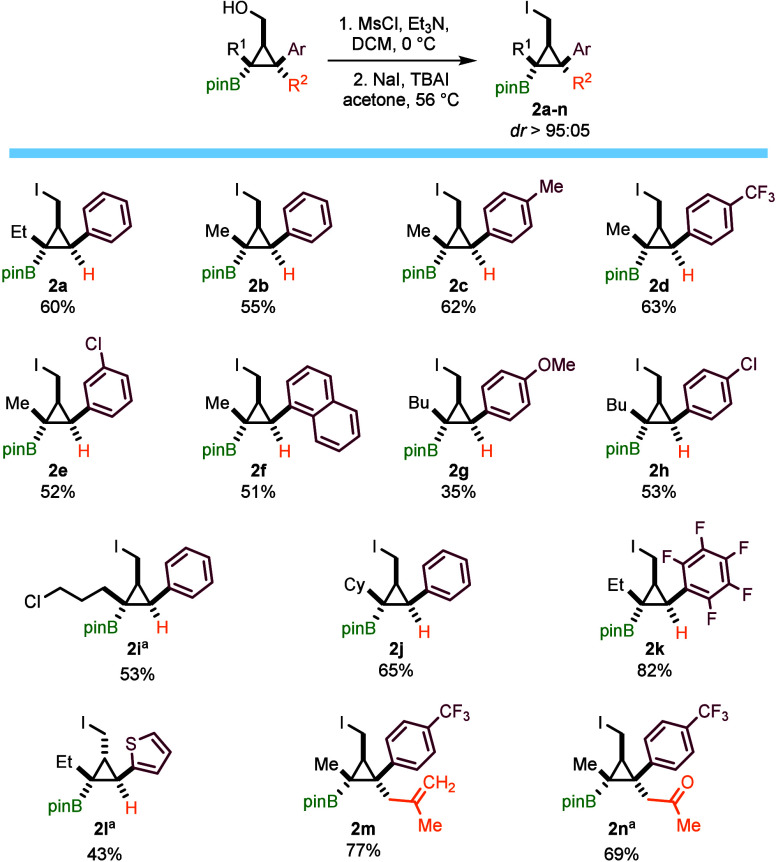
Synthesis of the Starting Materials

a Appel reaction was used instead.^15^

With convenient access to a variety of cyclopropylboronic methyl iodides **2**, we started to investigate the combined selective ring-opening and electrophilic reactions of the resulting α-boryl carbanions. After a thorough optimization of the reaction conditions (see Supporting Information for details), we identified the optimal protocol for generating α-boryl carbanions **4**: lithium–iodine exchange was carried out at −95 °C in diethyl ether using *t*BuLi, followed by the addition of Me_3_SiCl (Scheme 3, conditions A). Under these conditions, **5a** was obtained in moderate yield with an excellent diastereo­meric ratio. Alternatively, the reaction could also be conducted in THF instead of diethyl ether, provided that Me_3_SiCl was pre-added to the reaction mixture (internal quench, Scheme 3, conditions B).

**Scheme 3:**
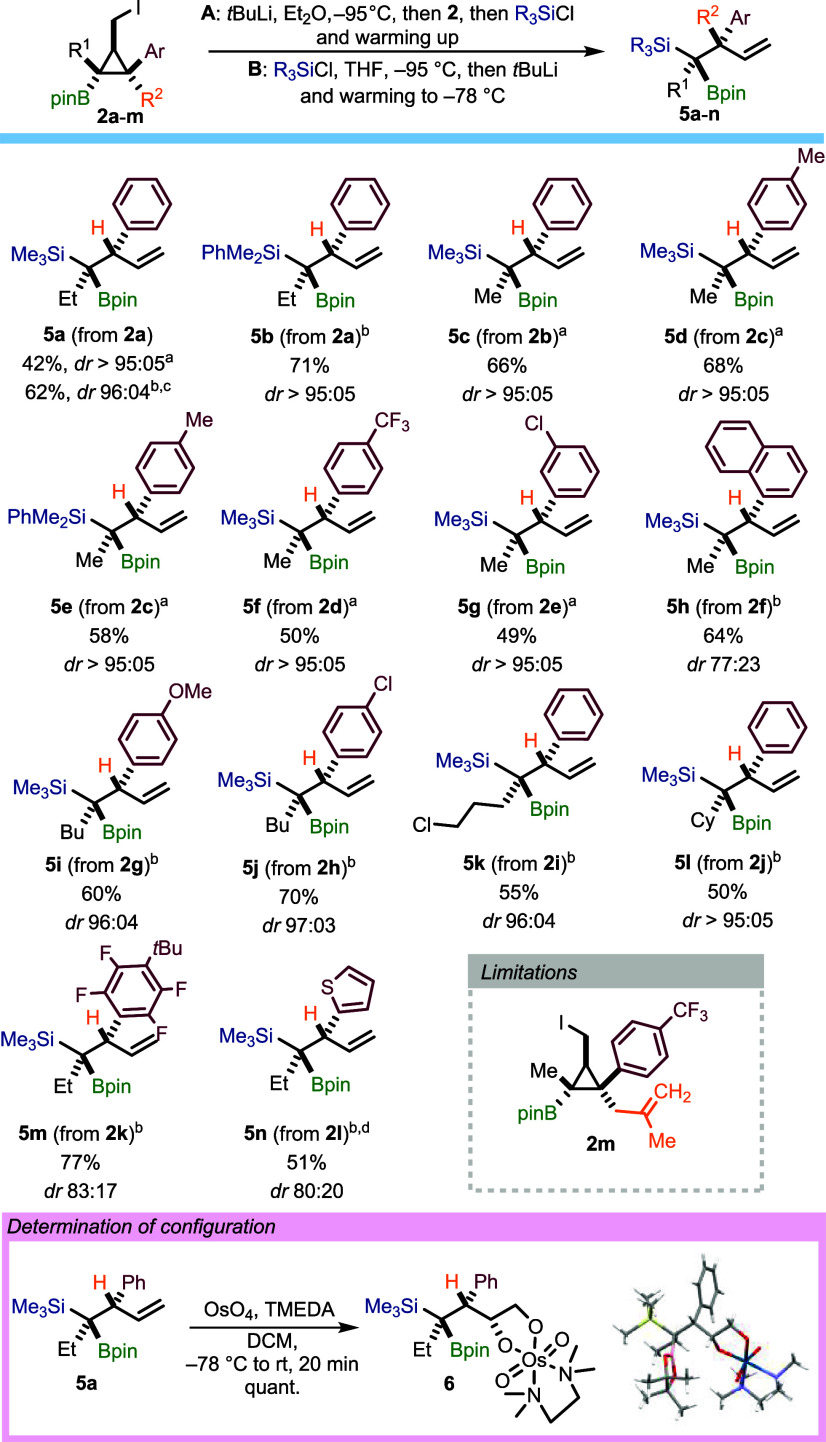
Scope of Ring-Opening/Silylation

a Conditions A were used.
b Conditions B were used.
c From 1.7 mmol of **2a**: 58% yield, *dr* 94:06.
d Along with 20 mol% of oversilylated product.

With these conditions in hand, we next investigated the scope and limitations of the reaction (Scheme 3). Both sets of conditions produced the same stereo­chemical outcome, although yields and diastereo­meric ratios varied slightly between them. The combined ring-opening and silylation reactions of **2a** proceeded efficiently with both Me_3_SiCl and PhMe_2_SiCl as electrophiles, providing the desired products **5a** and **5b** with excellent selectivity (Scheme 3). Similarly, clean transformations were observed across substrates **2b–e**, for both electron-rich and electron-poor aryl groups, affording acyclic boronic esters **5c**–**g** in good yields as single diastereomers. The naphthyl-substituted pinacol­borane **5h** was also synthesized in 66% yield, albeit with a moderate diastereo­meric ratio. Expanding the alkyl chain at R^1^ to butyl and cyclohexyl groups did not hamper the reaction outcome, and the corresponding products **5i**–**l** were obtained in 50–70% yield and with good selectivity. Importantly, compound **2i**, featuring a functional group within the R^1^ chain, was well-tolerated under the reaction conditions, affording silylated alkyl chloride **5k** in 55% yield.

Further modifications revealed certain limitations of the method. For instance, ring-opening of perfluoro­phenylated **2k** proceeded with a high yield of 77% and moderate diastereo­meric ratio, producing **5m** as a single product via simultaneous ring-opening, silylation, and *para*-selective S_N_Ar with *t*BuLi (Scheme 3).^39,40^ In the case of **2l**, with BPin and methylene iodide groups in the *syn* configuration, the reaction provided alkylsilane **5n** with the same stereo­chemical outcome, suggesting that the C_1_ stereo­chemistry does not alter the reactivity. Minor oversilylation was observed, likely resulting from *ortho*-lithiation of the thienyl ring followed by electrophilic trapping with Me_3_SiCl. Compound **2m** provided a complex mixture, presumably due to the high steric demand and the sensitive alkene functionality. **5a** was prepared on a 700 mg scale without a significant drop in yield and diastereo­meric ratio. The relative configuration was confirmed by X-ray crystallography analysis of **6**, which was readily obtained by treating **5a** with OsO_4_/TMEDA.^41^ The configurations of all other products were assigned by analogy.

Intrigued by the observed high diastereo­selectivity, we investigated the reaction mechanism employing density functional theory (DFT) calculations. Substrate **2b** was selected as a representative substrate, with Me_3_SiCl serving as the model electrophile. To simplify the system, we modified the boronic ester to an ethylene glycol derivative.

First, we studied the ring-opening of cyclopropyl methyl lithium **3b**, which generates the α-boryl carbanion intermediate **4b** (Scheme 4). The process commences with the formation of a transient intermediate **4b′**, passing through nearly barrierless transition state (TS1b, ΔΔG^⧧^ = 0.5 kcal/mol, Scheme 4). Subsequently, intermediate **4b′** undergoes virtually barrierless isomerization to form the more stable intermediate **4b**, characterized by its boron alkylidene properties: a short C–B bond length (1.47 Å compared to 1.54 Å in **3b**) and a planar carbanionic center (∑(∠C) = 359° compared to 351° in **3b**).^17^ We examined the possibility of the benzylic anion **8b** resulting from C_1_–C_2_ bond cleavage (Scheme 1c). We found that **8b** lies 8.3 kcal/mol higher than **4b**, suggesting a strong thermodynamic preference toward the observed ring-opening (see Supporting Information for details).

**Scheme 4:**
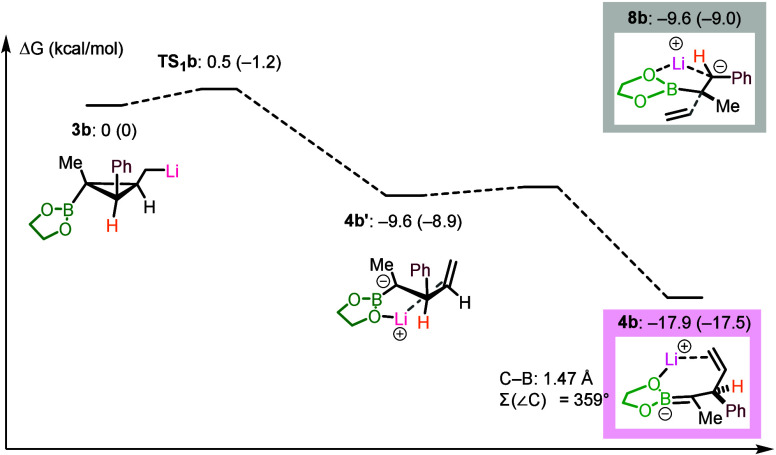
Ring-Opening of a Lithiated Cyclopropyl Boronic Ester^a^

a Calculated using the M062x-D3(0)/​def2-TZVP/​CPCM­(THF)//​M062x-D3(0)/​def2-SVP/CPCM­(THF) level of theory (see Supporting Information). ΔH values are shown in parentheses.

We then turned our attention to understand the origin of the diastereo­selectivity in the silylation step to form **5c**. Initial models using a free anion or implicitly solvated lithium-coordinated anion failed to match the experimental selectivity (see Supporting Information for details). These failures prompted the inclusion of explicit solvent molecules, revealing that the formation of the disolvated intermediate, **4b·2THF**, is highly favorable (ΔΔG = −11.8 kcal/mol, Scheme 5; for comparison between one, two, and three THF molecules see Supporting Information). Since the steric repulsion of the boronic ester is expected to be influential, the model substrate included a full pinacol boronic ester.

**Scheme 5:**
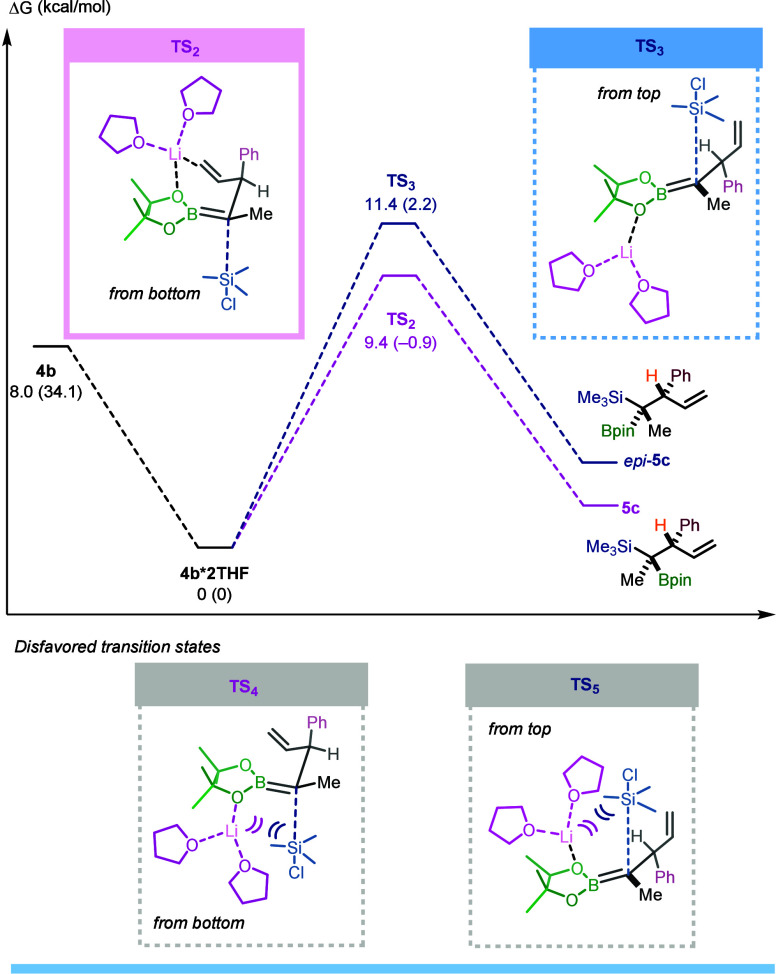
Silylation Reaction of a Solvated α-Boryl Carbanion^a^

a Calculated using the M062x-D3(0)/​def2-TZVP/​CPCM­(THF)//​M06L-D3(0)/​def2-SVP/​CPCM­(THF) level of theory (see Supporting Information). ΔH values are shown in parentheses.

The silylation of **4b·2THF** proceeds through two competing transition states, **TS**_**2**_ and **TS**_**3**_, leading to products **5c** (observed product) and *epi-* **5c**, respectively (Scheme 5). The conformational search (using CREST)^42^ revealed that the electrophile approaches exclusively from the face opposite the coordinating lithium cation (the complementary transition states are **TS**_**4**_ and **TS**_**5**_, Scheme 5; see Supporting Information for details). The calculated activation barrier for **TS**_**2**_ (ΔΔG^⧧^ = 9.4 kcal/mol) is 2.0 kcal/mol lower than the barrier for **TS**_**3**_ (ΔΔG^⧧^ = 11.4 kcal/mol), in agreement with the experimental outcome. The energetic preference arises from a stabilizing lithium cation-vinyl interaction in **TS**_**2**_, while no stabilizing interactions between the lithium cation and vinyl or phenyl groups appear for **TS**_**3**_.^43^ Presumably, other **TS**_**3**_ rotamers possessing a lithium cation-vinyl interaction are destabilized by elevated repulsive interactions, rendering them higher in energy than this lowest-energy conformer.

This intricate interplay of coordinative and counteracting repulsive interactions highlights the complexity of the system. Future work will focus on further elucidating this complex network of stabilizing and destabilizing interactions through experimental and computational investigations. Having established the scope and mechanism of the silylation reaction, we next evaluated the generality of the method by expanding the scope to a broader range of electrophiles (Scheme 6). Ring-opening of **2a** and subsequent reaction with methyl iodide provided the corresponding acyclic boronic ester **7a** in 62% yield and high 91:09 *dr*. Reactions with primary alkyl iodides, both linear (R = Bu) and branched (R = *s*Bu), resulted in clean reactions, affording **7b** and **7c** in 57% and 63% yields, respectively, as single diastereomers. When R^1^ = Me, a slight decrease of the diastereo­selectivity was observed with primary alkyl iodides (**7e**–**h**, Scheme 6). Nevertheless, products **7d**–**g** bearing electron-rich aryl substituents (Ph or *p*-Tol) were obtained in high diastereo­meric ratios and 40–78% yield. When a *p*-CF_3_ group was introduced into the substrate, the diastereo­meric ratio decreased further, and boronic ester **7h** was obtained in 57% yield as a 78:22 mixture of diastereomers. In contrast, extending the alkyl chain (R^1^ = Bu) led to improved diastereo­selectivity, providing alkyl boronic esters **7i** and **7j** as single diastereomers.

**Scheme 6:**
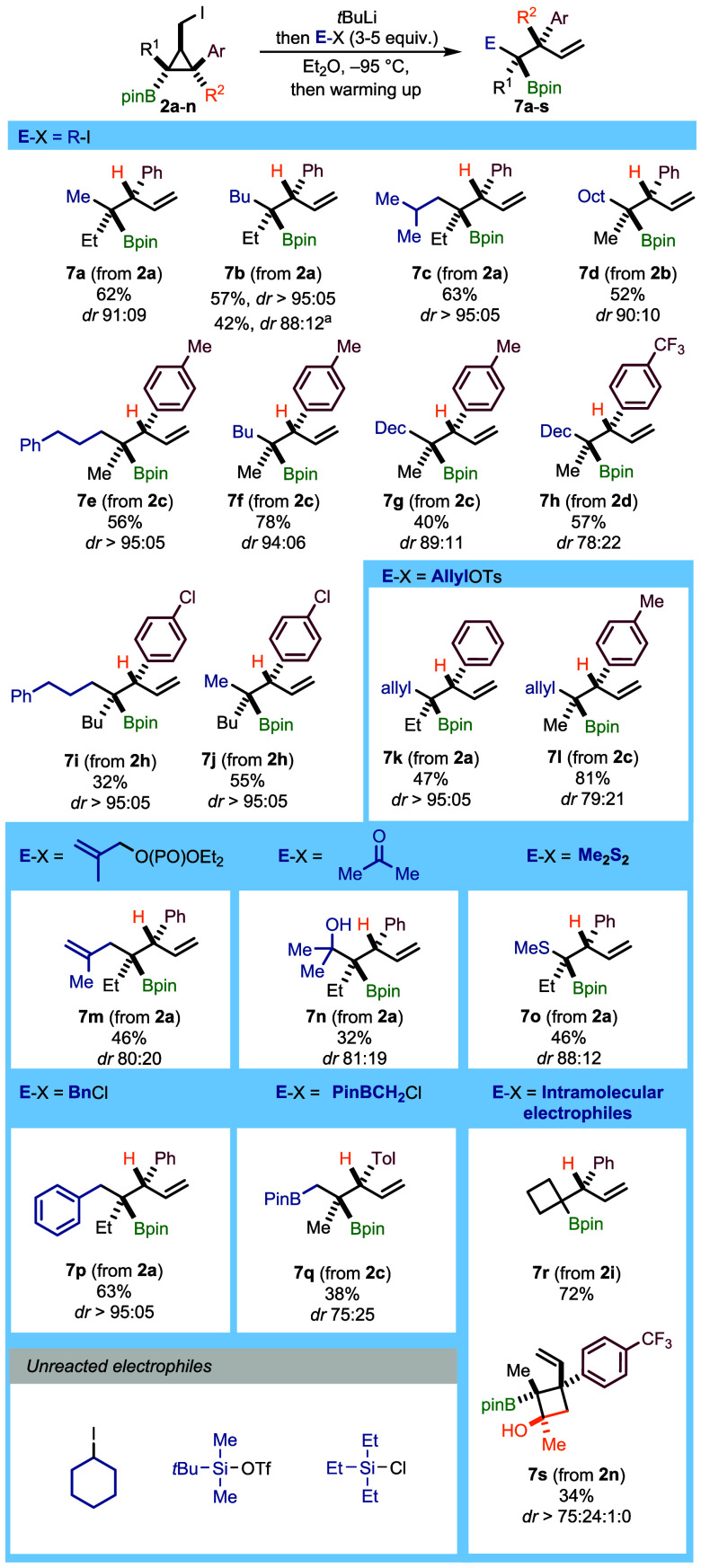
Scope of Electrophiles

a With BuBr instead of BuI; with BuCl: 35% yield, *dr* 88:12.

The structures of **7a**, **7b**, and related alkylated products were unambiguously assigned through comparison of their ^1^H and ^13^C spectra with data from our previous work on 1,2-metalate rearrangement.^14^ The observed configurations corresponded to electrophilic attack on the same diastereo­topic face of the boron alkylidene as in the silylation reactions.

The reaction also proved compatible with electrophiles beyond alkyl iodides: compound **7b** could be obtained in lower yields using BuBr and even BuCl as electrophiles with only a minor drop of *dr*. On the other hand, secondary alkyl iodides such as cyclohexyl iodide were unreactive under the reaction conditions. Similarly, bulky silyl chloride failed to react under the reaction conditions.

Allyl, methallyl, and benzyl reactants furnished compounds **7k**–**m** and **7p** in 46–81% yield and with high to excellent diastereo­selectivity. Reaction of **2a** with acetone provided β-hydroxy­pinacol­borane **7n** in modest yield and diastereo­selectivity. A sulfur-based electrophile was also tolerated, producing sulfide **7o** in moderate yield and with good diastereo­selectivity. Remarkably, Matteson-type homologation of a relatively congested ate complex was also feasible to give an orthogonally functionalized 1,2-bisboronic ester.

Additionally, we examined a small set of intra­molecular reactions. Chloride **2i** underwent smooth intra­molecular substitution under typical conditions in the absence of an external electrophile, furnishing cyclobutane **7r** in 72% yield (see Scheme 3, compound **5k**, for comparison). Finally, ketone **2n** reacted with complete chemo­selectivity for the lithium–iodine exchange.

The corresponding acyclic intermediate cyclized to form a borylated cyclobutanol with three stereo­centers in a 75:25 diastereo­meric ratio. The relative configuration of the major diastereomer of **7s** was determined by NOESY NMR analysis (see Supporting Information).

In conclusion, we exploited α-boryl carbanion intermediates as stereo­genic platforms in a series of highly diastereo­selective nucleophilic substitution reactions. This work introduces a novel strategy for accessing these important intermediates from easily accessible stereo­defined borylated cyclopropanes. Merging the anion-stabilizing effect of the boron atom with strain-release-driven fragmentation allowed for high chemo- and regio­selectivity, as demonstrated by the broad substrate scope and functional group tolerance. A variety of different electrophiles were employed to give acyclic boronic esters bearing vicinal tri- and tetrasubstituted stereo­centers. In contrast to our previous work on 1,2-metalate rearrangement, this transformation represents a formal umpolung reactivity, expanding the scope of cyclopropyl­boronic esters in stereo­selective synthesis.

## Supplementary Material



## Data Availability

The data underlying this study are available in the published article and its Supporting Information.

## Notes

General procedures, product characterization, and NMR spectra (PDF)
