# Supplementary material for: Vicinal Stereocenter Construction via α‑Boryl Carbanions from Borylated Cyclopropanes
Source: J Am Chem Soc. 2025 Oct 28;147(45):41204–9. doi: 10.1021/jacs.5c12433 (PMC13255249; doi:10.1021/jacs.5c12433)
Supplement: Supplementary file 1 [file ja5c12433_si_001.pdf]

Supporting information

Experimental and computational details and characterization data (PDF)

Vicinal Stereocenter Construction via α-Boryl Carbanion from Borylated Cyclopropanes

Tereza Pavlíčková, Noam Orbach, Alexander Kaushansky and Ilan Marek*

Table of Contents

1. General information 3

2. Synthesis of starting materials 4

2.1 Cyclopropenes S1-S12 4

2.2 Cyclopropyl carbinols 2–OH 7

2.3 Iodides 2a-m 15

3. Ring opening 24

3.1 Optimization of the ring opening/silylation 24

3.2 Scope of ring opening/silylation 25

3.3 Determination of the stereochemical outcome 37

3.4 Scope of electrophiles 39

4. Computational Details 54

4.1 Conformational search and analysis 54

4.2 Thermodynamics of the reaction 55

4.3 Ring opening of cyclopropylmethyl lithium 55

4.3.1 Formation of borata alkene intermediate **4b** 55

4.3.2 Formation of benzylic anion **8b** 56

4.4 Silylation reaction mechanism via monomer intermediate 57

4.4.1 Free anion model 57

4.4.2 Lithium-coordinated anion model 57

5. NMR spectra 61

6. References 231

1. General information

Air- and moisture-sensitive reactions were conducted in flame-dried glassware under a positive pressure of argon. Solvents were dried via distillation according to standard procedures (CH_2Cl_2, Et_3N) or used from a solvent purification system (THF, Et_2O; Pure-Solv.® Purification System) and stored at least 72 hours over activated 4Å molecular sieves before usage. Commercially available reagents were used as purchased unless otherwise stated. Commercially available organolithium reagents were titrated twice against *N*-benzylbenzamide before usage. Thin-layer chromatography (TLC) was conducted with E. Merck silica gel 60 F254 pre-coated plates (0.25 mm) and visualized by exposure to UV light (254 nm) or stained with acidic *p*-anisaldehyde, cerium molybdate, or potassium permanganate solutions. Column chromatography was performed using Fluka silica gel 60 Å (40-63 mm, 230-400 mesh). PE (60-80 °C boiling range) was used for chromatographic separations. NMR spectra were recorded in $CDCl_3$ on a *Bruker Avance* instrument. ^1H NMR chemical shifts are provided using TMS as external standard (internal reference at δ = 7.26 ppm) and are reported as follows: chemical shift in ppm [multiplicity, coupling constant(s) *J* in Hz, integral]. The following abbreviations were used for peak multiplicities: br = broad, m = multiplet, s = singlet, d = doublet, t = triplet, q = quadruplet, quint = quintuplet, sext = sextuplet, sept = septuplet or combinations thereof. Carbon (^{13}C, APT) chemical shifts are referenced against the residual central solvent peak (δ = 77.16 ppm for $CDCl_3$) and are given in ppm. Boron (^{11}B), fluorine (^{19}F) and phosphorus (^{31}P) shifts are given in ppm using external calibration. High-resolution mass spectroscopy (HRMS) was carried out on a FTICR instrument at the Mass Spectroscopy Unit of the Schulich Faculty of Chemistry at the Technion-Israel Institute of Technology. Diastereomeric ratios (*dr*) were determined either by crude ^1H NMR (relaxation delay D1 = 6 s) or by GC/FID analysis using an Agilent Technologies 7820A GC with an Agilent Technologies 19091J-413 (30 m × 0.3 mm) column.

2. Synthesis of starting materials

2.1 Cyclopropenes S1-S12

Cyclopropenes **S1**-**S8** are known compounds prepared according to literature procedures; the experimental results were in agreement with literature reports.[1] Cyclopropenes **S9**-**S12** were synthesized according to a literature procedure from corresponding cyclopropenyl stannanes;[2] the yields and data are reported below.

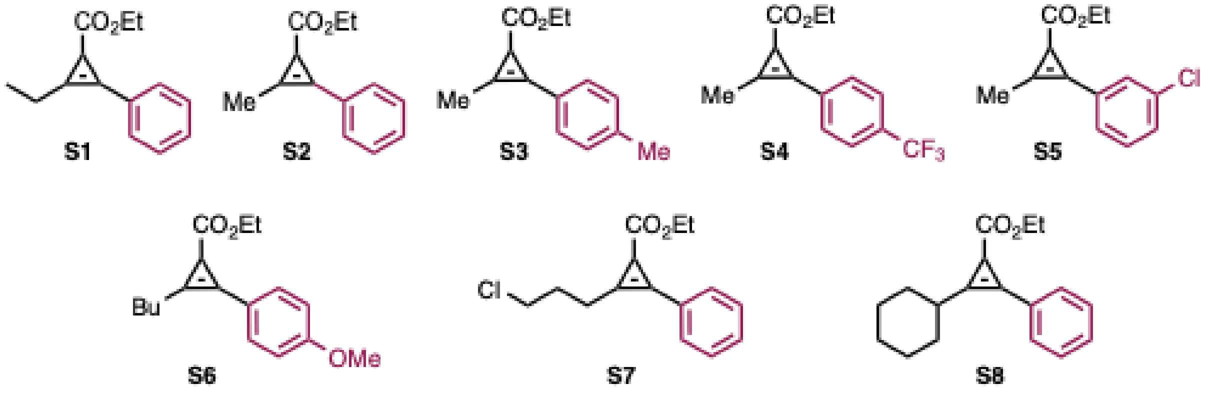

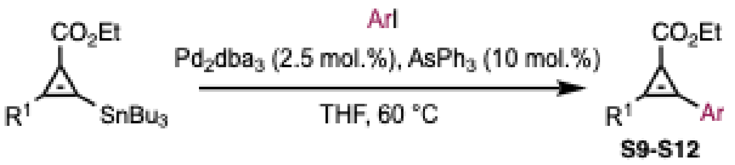

Ethyl 2-butyl-3-(4-chlorophenyl)cycloprop-2-ene-1-carboxylate S9

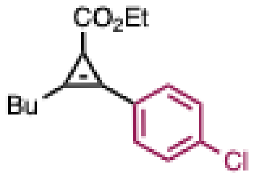

Chemical Formula: $C_{16}H_{19}ClO_2$
Molecular Weight: 278.78

Prepared according to a literature procedure[2] from corresponding cyclopropenyl stannane (2.29 g, 5.00 mmol) and *p*-chlorophenyl iodide (1.31 g, 5.50 mmol).

Yield: 1.06 g (3.80 mmol, 76%) as a pale yellow oil.

R*f* = 0.40 (PE/EtOAc = 90:10).

^{1}H NMR (400 MHz, $CDCl_3$): δ 7.41-7.30 (m, 4H), 4.14 (qd, *J* = 7.1, 4.0 Hz, 2H), 2.66 (t, *J* = 7.3 Hz, 2H), 2.42 (s, 1H), 1.76-1.65 (m, 2H), 1.51-1.35 (m, 2H), 1.24 (t, *J* = 7.1 Hz, 3H), 0.95 (t, *J* = 7.3 Hz, 3H) ppm.

^{13}C NMR (101 MHz, $CDCl_3$): δ 175.7, 134.6, 130.6, 129.1, 125.8, 111.5, 103.9, 60.3, 29.6, 25.3, 22.6, 22.3, 14.5, 13.9 ppm.

HRMS (APCI+) m/z: calcd. for $C_{16}H_{20}ClO_2^+$ [*M*+H]$^+$: 279.1146, found: 279.1145.

Ethyl 2-ethyl-3-(thiophen-2-yl)cycloprop-2-ene-1-carboxylate S10

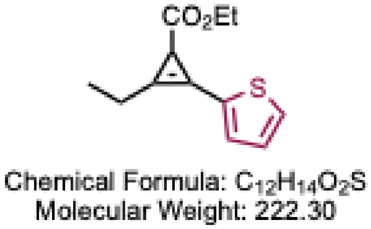

Prepared according to a literature procedure[2] from corresponding cyclopropenyl stannane (2.15 g, 5.00 mmol) and 2-thienyl iodide (1.16 g, 5.50 mmol).

Yield: 480 mg (2.16 mmol, 43%) as a pale yellow oil.

R*f* = 0.50 (PE/EtOAc = 90:10).

^{1}H NMR (400 MHz, $CDCl_3$): δ 7.40 (dd, *J* = 5.0, 1.2 Hz, 1H), 7.10 (dd, *J* = 3.7, 1.1 Hz, 1H), 7.04 (dd, *J* = 5.1, 3.6 Hz, 1H), 4.23-4.06 (m, 2H), 2.69-2.57 (m, 2H), 2.48 (s, 1H), 1.31 (t, *J* = 7.5 Hz, 3H), 1.24 (t, *J* = 7.1 Hz, 3H) ppm.

^{13}C NMR (101 MHz, $CDCl_3$) δ 175.3, 129.9, 128.8, 128.5, 127.7, 109.3, 98.6, 60.3, 23.8, 19.1, 14.5, 11.9 ppm.

HRMS (APCI+) m/z: calcd. for $C_{12}H_{15}O_2S^+$ [*M*+H]$^+$: 223.0787, found: 223.0769.

Ethyl 2-ethyl-3-(perfluorophenyl)cycloprop-2-ene-1-carboxylate S11

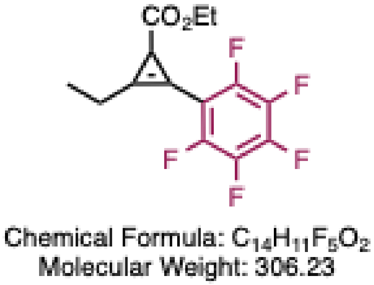

Prepared according to a literature procedure[2] from corresponding cyclopropenyl stannane (2.15 g, 5.00 mmol) and perfluorophenyl iodide (1.62 g, 5.5 mmol).

Yield: 951 mg (3.11 mmol, 62%) as a pale yellow oil.

R*f* = 0.50 (PE/EtOAc = 90:10).

^{1}H NMR (400 MHz, $CDCl_3$): δ 4.16 (dtt, *J* = 17.9, 10.8, 7.1 Hz, 2H), 2.74 (qd, *J* = 7.8, 5.0 Hz, 2H), 2.52 (s, 1H), 1.24 (t, *J* = 7.0 Hz, 5H), 1.21 (s, 1H) ppm.

^{13}C NMR (101 MHz, $CDCl_3$): δ 174.8, 146.2-145.9 (m), 143.7-143.3 (m), 142.9-142.7 (m), 140.4-140.1 (m), 139.2-138.9 (m), 136.7-136.5 (m), 118.3 (q, J_{C-F} = 3.9 Hz), 92.2, 60.7, 21.8, 19.3, 14.4, 11.0 ppm.

^{19}F NMR (377 MHz, $CDCl_3$): δ –140.78–-140.99 (m, 2F), –153.08 (t, J = 20.6 Hz, 1F), –162.17 (td, J = 21.3, 8.1 Hz, 2F) ppm.

HRMS (APCI+) m/z: calcd. for $C_{14}H_{12}F_5O_2^+$ [M+H]$^+$: 307.0734, found: 307.0752.

Ethyl 2-methyl-3-(naphthalen-1-yl)cycloprop-2-ene-1-carboxylate S12

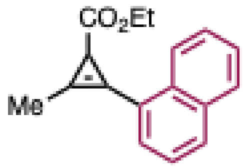

Chemical Formula: $C_{17}H_{16}O_2$
Molecular Weight: 252.31

Prepared according to a literature procedure[2] from corresponding cyclopropenyl stannane (1.47 g, 3.55 mmol) and 1-naphtyl iodide (992 mg, 3.91 mmol).

Yield: 570 mg (2.26 mmol, 64%) as a pale yellow oil.

R*f* = 0.35 (PE/EtOAc = 90:10).

^{1}H NMR (400 MHz, $CDCl_3$): δ 8.26 (d, J = 8.2 Hz, 1H), 7.89 (d, J = 8.1 Hz, 1H), 7.87-7.80 (m, 1H), 7.69-7.60 (m, 1H), 7.59-7.45 (m, 3H), 4.27-4.10 (m, 2H), 2.57 (s, 3H), 2.53 (s, 1H), 1.26 (t, J = 7.1 Hz, 3H) ppm.

^{13}C NMR (101 MHz, $CDCl_3$): δ 175.9, 133.6, 131.7, 129.6, 129.0, 128.7, 127.0, 126.4, 125.7, 125.3, 124.7, 107.8, 103.9, 60.3, 21.8, 14.6, 12.0 ppm.

HRMS (APCI+) m/z: calcd. for $C_{17}H_{17}O_2^+$ [M+H]$^+$: 253.1223, found: 253.1245.

2.2 Cyclopropyl carbinols **2**–OH

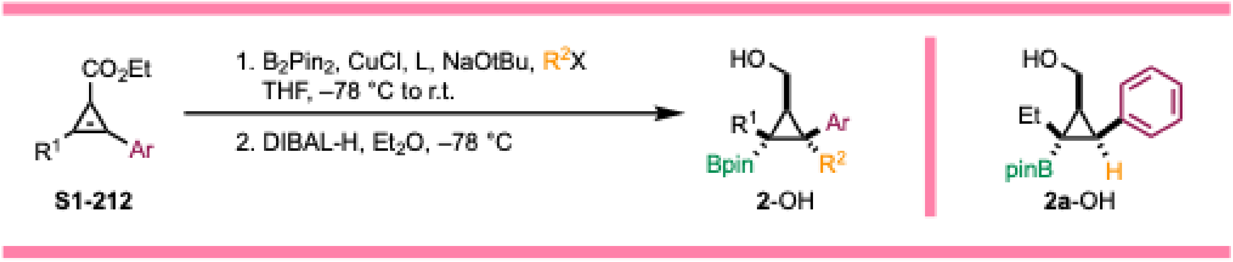

Cyclopropyl carbinol **2a**-OH is a known compound prepared according to a literature procedure from **S1**;[3] the experimental results were in agreement with the literature report.[3b] Cyclopropyl carbinols **2b-l**-OH and **S13** were synthesized according to a literature procedure from corresponding cyclopropenes;[3] the yields and data are reported below.

((1*S,2*R**,3*R**)-2-Methyl-3-phenyl-2-(4,4,5,5-tetramethyl-1,3,2-dioxaborolan-2-yl)cyclopropyl)methanol 2b**-OH

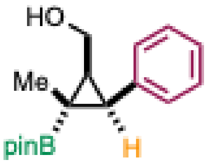

Chemical Formula: $C_{17}H_{25}BO_3$
Molecular Weight: 288.19

Prepared according to a literature procedure from **S2**[3] (382 mg, 1.89 mmol).

Yield: 240 mg (830 μmol, 44% over two steps, *dr* > 95:05 as determined by ^{1}H NMR spectroscopy) as a white solid.

R*f* = 0.40 (PE/Et_2O 1:1).

^{1}H NMR (400 MHz, $CDCl_3$): δ 7.30-7.24 (m, 2H), 7.24-7.16 (m, 3H), 3.84 (dd, *J* = 11.4, 6.7 Hz, 1H), 3.56 (dd, *J* = 11.4, 8.1 Hz, 1H), 2.46 (d, *J* = 8.7 Hz, 1H), 1.70-1.60 (m, 2H), 1.26 (s, 6H), 1.25 (s, 6H), 0.96 (s, 3H) ppm.

^{13}C NMR (101 MHz, $CDCl_3$): δ 136.5, 131.1, 128.3, 126.3, 83.5, 60.3, 29.9, 27.5, 24.8, 24.8, 10.6 ppm.
Note: Carbon atom attached to boron is not visible due to quadrupolar relaxation.

^{11}B NMR (128 MHz, $CDCl_3$): δ 33.9 ppm.

HRMS (TOF ESI+) m/z: calcd. for $C_{17}H_{27}BO_4^+$ [*M*+H_2O]$^+$: 306.2002, found: 306.1986.

((1*S,2*R**,3*R**)-2-Methyl-2-(4,4,5,5-tetramethyl-1,3,2-dioxaborolan-2-yl)-3-(*p*-tolyl)cyclopropyl)methanol 2c-OH**

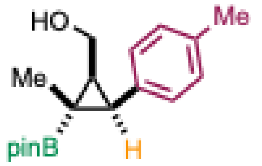

Prepared according to a literature procedure from **S3**[3] (654 mg, 3.49 mmol).

Yield: 600 mg (1.99 mmol, 57% over two steps, *dr* > 95:05 as determined by ^{1}H NMR spectroscopy) as a colorless oil.

R*f* = 0.40 (PE/Et_2O 1:1).

^{1}H NMR (400 MHz, $CDCl_3$): δ 7.14-6.98 (m, 4H), 3.87-3.81 (m, 1H), 3.56 (t, *J* = 9.4 Hz, 1H), 2.41 (d, *J* = 8.4 Hz, 1H), 2.31 (s, 3H), 1.62 (q, *J* = 7.6 Hz, 1H), 1.35 (s, 1H), 1.25 (s, 12H), 0.96 (s, 3H) ppm.

^{13}C NMR (101 MHz, $CDCl_3$): δ 135.8, 133.3, 130.9, 129.1, 83.4, 60.4, 29.6, 27.6, 24.8, 21.2, 10.6 ppm.
Note: Carbon atom attached to boron is not visible due to quadrupolar relaxation.

^{11}B NMR (128 MHz, $CDCl_3$): δ 33.8 ppm.

HRMS (APCI+) m/z: calcd. for $C_{18}H_{26}BO_2^+$ [*M*–H_2O+H]$^+$: 285.2020, found: 285.2030.

((1*S,2*R**,3*R**)-2-Methyl-2-(4,4,5,5-tetramethyl-1,3,2-dioxaborolan-2-yl)-3-(4-(trifluoromethyl)phenyl)cyclopropyl)methanol 2d-OH**

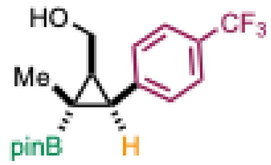

Prepared according to a literature procedure from **S4**[3] (303 mg, 1.12 mmol).

Yield: 200 mg (651 µmol, 50% over two steps, *dr* > 95:05 as determined by ^{1}H NMR spectroscopy) as a colorless oil.

R*f* = 0.40 (PE/Et_2O 1:1).

^{1}H NMR (400 MHz, $CDCl_3$): δ 7.51 (d, *J* = 8.0 Hz, 2H), 7.34 (d, *J* = 7.9 Hz, 2H), 3.75 (dd, *J* = 11.4, 7.1 Hz, 1H), 3.58 (dd, *J* = 11.4, 7.6 Hz, 1H), 2.44 (d, *J* = 8.5 Hz, 1H), 2.06 (br s, 1H), 1.66 (q, *J* = 7.8 Hz, 1H), 1.24 (s, 6H), 1.23 (s, 6H), 0.92 (s, 3H) ppm.

^{13}C NMR (101 MHz, $CDCl_3$): δ 140.8, 131.5, 128.52 (q, $J_{C–F}$ = 32.1 Hz), 125.79 (d, $J_{C–F}$ = 273.1 Hz), 125.20 (q, $J_{C–F}$ = 3.8 Hz), 83.6, 59.9, 29.5, 27.3, 24.8, 24.7, 10.6 ppm.
Note: Carbon atom attached to boron is not visible due to quadrupolar relaxation.

^{19}F NMR (377 MHz, $CDCl_3$): δ –62.4 ppm.

^{11}B NMR (128 MHz, $CDCl_3$): δ 32.0 ppm.

HRMS (APCI+) m/z: calcd. for $C_{18}H_{23}BF_3O_2^+$ [*M*–H_2O+H]$^+$: 339.1738, found: 339.1765.

((1*S,2*R**,3*R**)-3-(3-Chlorophenyl)-2-methyl-2-(4,4,5,5-tetramethyl-1,3,2-dioxaborolan-2-yl)cyclopropyl)methanol 2e**-OH

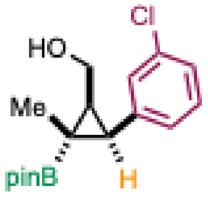

Chemical Formula: $C_{17}H_{24}BClO_3$
Molecular Weight: 322.64

Prepared according to a literature procedure from **S4**[3] (303 mg, 1.12 mmol).

Yield: 200 mg (651 µmol, 50% over two steps, *dr* > 95:05 as determined by ^{1}H NMR spectroscopy) as a colorless oil.

R*f* = 0.40 (PE/Et_2O 1:1).

^{1}H NMR (400 MHz, $CDCl_3$): δ 7.23-7.15 (m, 3H), 7.15-7.06 (m, 1H), 3.79 (dt, *J* = 11.3, 6.3 Hz, 1H), 3.57 (t, *J* = 8.8 Hz, 1H), 2.40 (d, *J* = 8.6 Hz, 1H), 1.70-1.57 (m, 2H), 1.25 (s, 6H), 1.24 (s, 6H), 0.94 (s, 3H) ppm.

^{13}C NMR (101 MHz, $CDCl_3$): δ 138.6, 134.0, 131.1, 129.6, 129.3, 126.5, 83.6, 60.0, 29.5, 27.3, 24.8, 24.8, 10.6 ppm.
Note: Carbon atom attached to boron is not visible due to quadrupolar relaxation.

^{11}B NMR (128 MHz, $CDCl_3$): δ 33.1 ppm.

HRMS (APCI+) m/z: calcd. for $C_{17}H_{23}BClO_2^+$ [*M*–H_2O+H]$^+$: 305.1480, found: 305.1508.

((1*S,2*R**,3*R**)-2-Methyl-3-(naphthalen-1-yl)-2-(4,4,5,5-tetramethyl-1,3,2-dioxaborolan-2-yl)cyclopropyl)methanol 2f**-OH

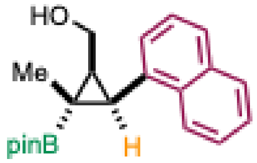

Chemical Formula: $C_{21}H_{27}BO_3$
Molecular Weight: 338.25

Prepared according to a literature procedure from **S12**[3] (471 mg, 1.87 mmol).

Yield: 190 mg (562 µmol, 30% over two steps, *dr* > 95:05 as determined by ^{1}H NMR spectroscopy) as a white solid.

R*f* = 0.40 (PE/Et_2O 1:1).

^{1}**H NMR** (400 MHz, $CDCl_3$): δ 8.34-8.05 (m, 1H), 7.87-7.80 (m, 1H), 7.73 (d, *J* = 7.8 Hz, 1H), 7.49 (ddd, *J* = 6.4, 3.7, 1.7 Hz, 2H), 7.43-7.33 (m, 2H), 3.99 (dd, *J* = 11.0, 5.5 Hz, 1H), 3.69 (t, *J* = 9.1 Hz, 1H), 2.68 (d, *J* = 8.5 Hz, 1H), 1.89 (q, *J* = 8.2 Hz, 1H), 1.34 (s, 6H), 1.32 (s, 6H), 0.87 (s, 3H) ppm.

^{13}**C NMR** (101 MHz, $CDCl_3$): δ 134.0, 133.9, 133.2, 128.6, 128.0, 127.2, 125.9, 125.7, 125.4, 124.8, 83.7, 60.6, 28.6, 27.8, 25.0, 24.9, 10.3 ppm.
Note: Carbon atom attached to boron is not visible due to quadrupolar relaxation.

^{11}**B NMR** (128 MHz, $CDCl_3$): δ 33.9 ppm.

HRMS (APCI+) m/z: calcd. for $C_{21}H_{26}BO_2^+$ [*M*–H_2O+H]$^+$: 321.2026, found: 321.2047.

((1*S,2*R**,3*R**)-2-Ethyl-3-(perfluorophenyl)-2-(4,4,5,5-tetramethyl-1,3,2-dioxaborolan-2-yl)cyclopropyl)methanol 2k**-OH

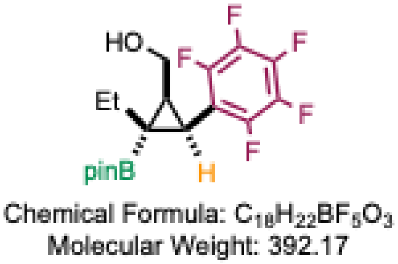

Prepared according to a literature procedure from **S11**[3] (924 mg, 3.02 mmol).

Yield: 581 mg (1.45 mmol, 48% over two steps, *dr* > 95:05 as determined by ^{1}H NMR spectroscopy) as a white solid.

R*f* = 0.40 (PE/Et_2O 1:1).

^{1}**H NMR** (400 MHz, $CDCl_3$): δ 3.90 (dt, *J* = 11.7, 5.2 Hz, 1H), 3.59 (dt, *J* = 11.6, 7.3 Hz, 1H), 1.98 (d, *J* = 8.6 Hz, 1H), 1.87 (td, *J* = 8.0, 6.1 Hz, 1H), 1.84-1.74 (m, 1H), 1.57 (s, 1H), 1.25 (s, 6H), 1.24 (s, 6H), 1.04 (t, *J* = 7.3 Hz, 3H), 0.70 (dq, *J* = 14.5, 7.3 Hz, 1H) ppm.

^{13}**C NMR** (101 MHz, $CDCl_3$): δ 148.3-147.9 (m), 145.7 (t, $J_{C–F}$ = 9.8 Hz), 141.5-141.03 (m), 139.2-138.6 (m), 136.4 (t, $J_{C–F}$ = 16.1 Hz), 111.0 (td, $J_{C–F}$ = 19.1, 4.2 Hz), 83.7, 59.6 (t, $J_{C–F}$ = 4.9 Hz), 27.1, 24.9, 24.5, 19.5, 19.2, 13.2 ppm.
Note: Carbon atom attached to boron is not visible due to quadrupolar relaxation.

^{19}**F NMR** (377 MHz, $CDCl_3$) δ –138.73 (dd, *J* = 23.6, 7.8 Hz, 2F), –156.27 (t, *J* = 20.1 Hz, 1F), –162.42 (tt, *J* = 21.7, 6.3 Hz, 2F) ppm.

^{11}**B NMR** (128 MHz, $CDCl_3$): δ 32.9 ppm.

HRMS (APCI+) m/z: calcd. for $C_{18}H_{21}BF_5O_2^+$ [*M*–H_2O+H]$^+$: 375.1549, found: 385.1559.

((1*R,2*S**,3*R**)-2-Ethyl-2-(4,4,5,5-tetramethyl-1,3,2-dioxaborolan-2-yl)-3-(thiophen-2-yl)cyclopropyl)methanol 2l-OH**

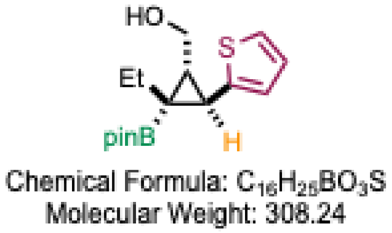

Prepared according to a literature procedure from **S10**[3] (278 mg, 1.25 mmol).

Yield: 184 mg (596 μμολ, 48% over two steps, *dr* > 95:05 as determined by ^{1}H NMR spectroscopy) as a colorless oil.

R*f* = 0.40 (PE/Et_2O 1:1).

^{1}H NMR (400 MHz, $CDCl_3$): δ 7.09 (d, *J* = 5.0 Hz, 1H), 6.97-6.87 (m, 1H), 6.79 (d, *J* = 3.4 Hz, 1H), 3.98 (d, *J* = 6.9 Hz, 1H), 3.58 (t, *J* = 9.2 Hz, 1H), 2.42 (d, *J* = 5.6 Hz, 1H), 1.59-1.50 (m, 1H), 1.38-1.24 (m, 1H), 1.28 (s, 6H), 1,27 (s, 6H), 1.16 (dd, *J* = 14.0, 7.2 Hz, 1H), 0.84 (t, *J* = 7.3 Hz, 3H) ppm.
Note: The O*H* resonance was not visible.

^{13}C NMR (101 MHz, $CDCl_3$): δ 142.7, 126.6, 125.7, 123.4, 83.7, 63.8, 35.0, 27.0, 25.0, 24.7, 24.3, 13.2 ppm.
Note: Carbon atom attached to boron is not visible due to quadrupolar relaxation.

^{11}B NMR (128 MHz, $CDCl_3$): δ 32.8 ppm.

HRMS (APCI+) m/z: calcd. for $C_{16}H_{26}BO_3S^+$ [*M*–H_2O+H]$^+$: 309.1696, found: 309.1716.

Note: The compound quickly cyclized to form the corresponding mixed boronic ester; full characterization data including ^{1}H, ^{13}C and ^{11}B NMR spectra is given for iodide **2h** instead.

((1*S,2*R**,3*R**)-2-Butyl-3-(4-chlorophenyl)-2-(4,4,5,5-tetramethyl-1,3,2-dioxaborolan-2-yl)cyclopropyl)methanol 2h-OH**

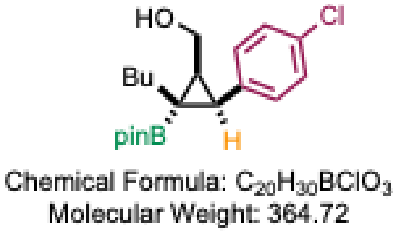

Prepared according to a literature procedure from **S9**[3] (661 mg, 2.37 mmol).

Yield: 320 mg (878 µmol, 37% over two steps, *dr* > 95:05 as determined by ^{1}H NMR spectroscopy) as a colorless oil.

R*f* = 0.40 (PE/Et_2O 1:1).

^{1}H NMR (400 MHz, $CDCl_3$): δ 7.23 (dd, *J* = 13.2, 8.2 Hz, 4H), 3.81 (dd, *J* = 11.2, 6.5 Hz, 1H), 3.67 (t, *J* = 9.7 Hz, 1H), 2.29 (d, *J* = 8.6 Hz, 1H), 1.80 (br s, 1H), 1.65 (td, *J* = 8.3, 6.4 Hz, 1H), 1.59-1.36 (m, 3H), 1.24 (s, 6H), 1.22 (s, 6H), 1.31-1.09 (m, 3H), 0.80 (t, *J* = 7.0 Hz, 3H) ppm.

^{13}**C NMR** (101 MHz, $CDCl_3$): δ 135.4, 132.4, 132.0, 128.4, 83.4, 59.8, 32.0, 28.9, 27.3, 26.7, 24.9, 24.4, 23.4, 14.2 ppm.
Note: Carbon atom attached to boron is not visible due to quadrupolar relaxation.

^{11}**B NMR** (128 MHz, $CDCl_3$): δ 32.6 ppm.

HRMS (APCI+) m/z: calcd. for $C_{20}H_{29}BClO_2^+$ [*M*–H_2O+H]$^+$: 347.1944, found: 347.1910.

((1*S,2*R**,3*R**)-2-Butyl-3-(4-methoxyphenyl)-2-(4,4,5,5-tetramethyl-1,3,2-dioxaborolan-2-yl)cyclopropyl)methanol 2g**-OH

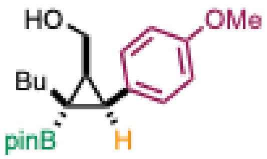

Prepared according to a literature procedure from **S6**[3] (1.80 g, 6.58 mmol).

Yield: 1.19 g (3.75 mmol, 57% over two steps, *dr* > 95:05 as determined by ^{1}H NMR spectroscopy) as a colorless oil.

R*f* = 0.30 (PE/Et_2O 1:1).

^{1}**H NMR** (400 MHz, $CDCl_3$): δ 7.22-7.17 (m, 2H), 6.87-6.75 (m, 2H), 3.85-3.64 (m, 2H), 3.78 (s, 3H), 2.29 (d, *J* = 8.6 Hz, 1H), 1.69-1.58 (m, 1H), 1.54-1.35 (m, 3H), 1.25 (s, 6H), 1.22 (s, 6H), 1.30-1.13 (m, 3H), 0.82 (t, *J* = 7.1 Hz, 3H) ppm.

^{13}**C NMR** (101 MHz, $CDCl_3$): δ 158.0, 131.9, 128.9, 113.7, 83.2, 60.2, 55.3, 32.2, 28.7, 27.5, 26.7, 25.0, 24.5, 23.5, 14.2 ppm.
Note: Carbon atom attached to boron is not visible due to quadrupolar relaxation.

^{11}**B NMR** (128 MHz, $CDCl_3$): δ 34.6 ppm.

HRMS (TOF ESI+) m/z: calcd. for $C_{21}H_{35}BO_5^+$ [*M*+H_2O]$^+$: 378.2578, found: 378.2561.

((1*S,2*R**,3*R**)-2-Cyclohexyl-3-phenyl-2-(4,4,5,5-tetramethyl-1,3,2-dioxaborolan-2-yl)cyclopropyl)methanol 2j**-OH

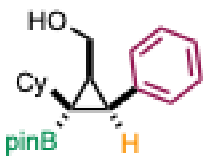

Prepared according to a literature procedure from **S8**[3] (571 mg, 2.23 mmol).

Yield: 325 mg (913 µmol, 41% over two steps, *dr* > 95:05 as determined by ^{1}H NMR spectroscopy) as a colorless oil.

R*f* = 0.35 (PE/Et_2O 1:1).

^{1}H NMR (400 MHz, $CDCl_3$): δ 7.37 (d, *J* = 7.4 Hz, 2H), 7.31-7.21 (m, 2H), 7.17 (t, *J* = 7.2 Hz, 1H), 4.17-4.02 (m, 1H), 4.01-3.84 (m, 1H), 2.49 (d, *J* = 8.7 Hz, 1H), 1.75 (br s, 1H), 1.71-1.59 (m, 3H), 1.55 (dd, *J* = 13.6, 9.1 Hz, 2H), 1.50-1.42 (m, 2H), 1.34-1.17 (m, 2H), 1.24 (s, 6H), 1.23 (s, 6H), 1.11 (dt, *J* = 24.2, 13.0 Hz, 1H), 0.99-0.71 (m, 2H) ppm.

^{13}C NMR (101 MHz, $CDCl_3$): δ 137.9, 130.2, 128.0, 125.8, 82.9, 60.1, 36.1, 32.1, 31.3, 29.7, 28.9, 27.1, 27.0, 26.4, 24.8, 24.6 ppm.
Note: Carbon atom attached to boron is not visible due to quadrupolar relaxation.

^{11}B NMR (128 MHz, $CDCl_3$): δ 32.9 ppm.

HRMS (TOF ESI+) m/z: calcd. for $C_{22}H_{33}BO_3Na^+$ [*M*+Na]$^+$: 379.2420, found: 379.2402.

((1*S,2*R**,3*R**)-2-(3-Chloropropyl)-3-phenyl-2-(4,4,5,5-tetramethyl-1,3,2-dioxaborolan-2-yl)cyclopropyl)methanol 2i**-OH

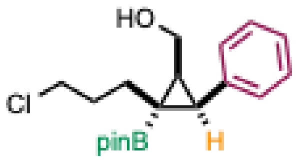

Chemical Formula: $C_{19}H_{28}BClO_3$
Molecular Weight: 350.69

Prepared according to a literature procedure from **S7**[3] (723 mg, 2.74 mmol).

Yield: 500 mg (1.43 mmol, 52% over two steps, *dr* > 95:05 as determined by ^{1}H NMR spectroscopy) as a colorless oil.

R*f* = 0.45 (PE/Et_2O 1:1).

^{1}H NMR (400 MHz, $CDCl_3$): δ 7.32-7.14 (m, 5H), 3.92-3.83 (m, 1H), 3.77-3.68 (m, 1H), 3.57-3.34 (m, 2H), 2.42 (d, *J* = 8.7 Hz, 1H), 2.05 (tt, *J* = 11.2, 5.7 Hz, 1H), 1.84 (ddd, *J* = 13.7, 6.9, 3.4 Hz, 1H), 1.76-1.64 (m, 1H), 1.57 (s, 1H), 1.25 (s, 6H), 1.23 (s, 6H), 1.00 (td, *J* = 13.6, 4.1 Hz, 1H), 0.92-0.77 (m, 1H) ppm.

^{13}C NMR (101 MHz, $CDCl_3$): δ 136.5, 130.8, 128.4, 126.4, 83.5, 59.9, 45.7, 32.7, 29.8, 27.5, 25.0, 24.6, 24.5 ppm.
Note: Carbon atom attached to boron is not visible due to quadrupolar relaxation.

^{11}B NMR (128 MHz, $CDCl_3$): δ 33.1 ppm.

HRMS (APCI+) m/z: calcd. for $C_{19}H_{27}BClO_2^+$ [*M*–H_2O+H]$^+$: 333.1793, found: 333.1804.

((1*R,2*S**,3*R**)-2-Methyl-3-(2-methylallyl)-2-(4,4,5,5-tetramethyl-1,3,2-dioxaborolan-2-yl)-3-(4-(trifluoromethyl)phenyl)cyclopropyl)methanol 2m**-OH

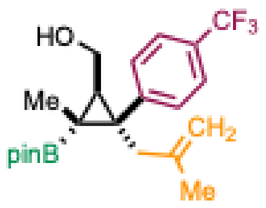

Chemical Formula: $C_{22}H_{30}BF_3O_3$
Molecular Weight: 410.28

Prepared according to a literature procedure from **S4**[3] (2.19 g, 8.12 mmol).

Yield: 1.64 g (4.02 mmol, 50% over two steps, *dr* > 95:05 as determined by ^{1}H NMR spectroscopy) as a colorless oil.

R*f* = 0.40 (PE/Et_2O 1:1).

^{1}H NMR (400 MHz, $CDCl_3$): δ 7.49 (d, *J* = 8.0 Hz, 2H), 7.29 (d, *J* = 7.9 Hz, 2H), 4.50 (d, *J* = 1.9 Hz, 1H), 4.32 (d, *J* = 2.3 Hz, 1H), 3.73 (t, *J* = 9.6 Hz, 1H), 3.62 (dd, *J* = 11.3, 7.4 Hz, 1H), 2.48 (d, *J* = 13.8 Hz, 1H), 2.27 (d, *J* = 13.8 Hz, 1H), 1.87 (s, 1H), 1.70 (t, *J* = 7.5 Hz, 1H), 1.65 (s, 3H), 1.28 (s, 6H), 1.25 (s, 6H), 0.82 (s, 3H) ppm.

^{13}C NMR (101 MHz, $CDCl_3$): δ 143.6, 143.5, 131.7, 128.9 (q, J_{C-F} = 32.3 Hz), 124.9 (q, J_{C-F} = 3.8 Hz), 124.5 (q, J_{C-F} = 271.8 Hz), 112.8, 83.7, 60.9, 48.9, 39.0, 34.2, 25.3, 24.7, 23.0, 13.7 ppm.
Note: Carbon atom attached to boron is not visible due to quadrupolar relaxation.

^{19}F NMR (377 MHz, $CDCl_3$): δ –62.3 ppm.

^{11}B NMR (128 MHz, $CDCl_3$): δ 32.4 ppm.

1-((1*R,2*S**,3*R**)-3-(Hydroxymethyl)-2-methyl-2-(4,4,5,5-tetramethyl-1,3,2-dioxaborolan-2-yl)-1-(4-(trifluoromethyl)phenyl)cyclopropyl)propan-2-one 2n**-OH

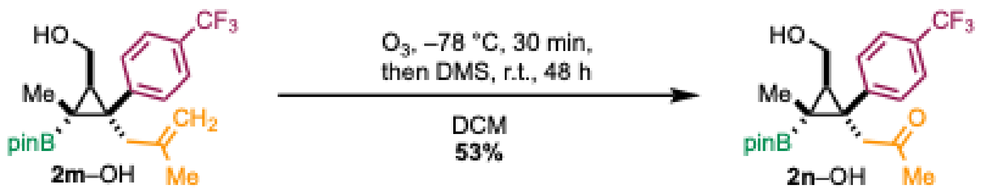

Cyclopropyl carbinol **2m**-OH (290 mg, 682 µmol) was dissolved in dry DCM (5.0 mL) in a flame-dried Schlenk flask under argon. The solution was cooled to –78 °C and ozone was bubbled through the mixture for 30 min, until lightly blue color was observed. The mixture was purged with argon and dimethyl sulfide (300 µL, 4.10 mmol) was added. The cold bath was removed and the mix was stirred for 24 h at room temperature. The volatiles were removed under reduced pressure and the crude residue was purified by flash column chromatography (silica gel, gradient PE/EtOAc 99:1 to 50:50) to yield **2n**-OH (150 mg 1.43 mmol, 53%, *dr* > 95:05 as determined by ^{1}H NMR spectroscopy) as a colorless oil.

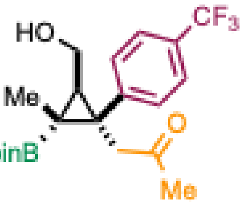

Chemical Formula: $C_{21}H_{28}BF_3O_4$
Molecular Weight: 412.26

R*f* = 0.20 (PE/Et_2O 1:1).

^{1}H NMR (400 MHz, $CDCl_3$): δ 7.52 (d, *J* = 8.2 Hz, 2H), 7.46 (d, *J* = 8.2 Hz, 2H), 3.73-3.52 (m, 2H), 2.97 (s, 2H), 2.14 (s, 1H), 1.93 (br s, 3H), 1.60-1.47 (m, 1H), 1.26 (s, 6H), 1.23 (s, 6H), 0.84 (s, 3H) ppm.

^{13}C NMR (101 MHz, $CDCl_3$): δ 207.9, 143.8, 132.0, 128.7 (q, $J_{C–F}$ = 32.3 Hz), 125.3 (q, $J_{C–F}$ = 3.7 Hz), 124.4 (q, $J_{C–F}$ = 271.8 Hz), 83.7, 60.6, 53.8, 35.1, 34.4, 30.6, 25.1, 24.9, 13.9 ppm.

^{19}F NMR (377 MHz, $CDCl_3$): δ –62.4 ppm.

^{11}B NMR (128 MHz, $CDCl_3$): δ 32.6 ppm.

HRMS (APCI+) m/z: calcd. for $C_{21}H_{29}BF_3O_4^+$ [*M*+H]$^+$: 413.2106, found: 413.2132.

2.3 Iodides **2a-m**

Compounds **2h**, **2l** and **2n** were prepared by Appel reaction according to a previously published procedure.[3b]

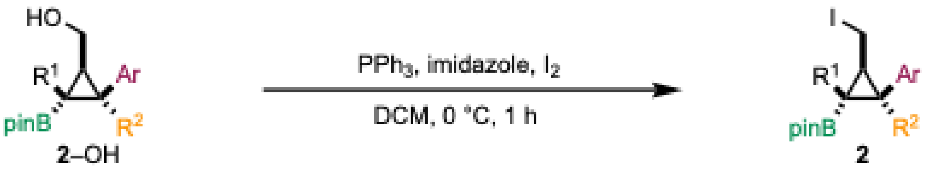

2-((1*S,2*S**,3*R**)-1-Ethyl-2-(iodomethyl)-3-(thiophen-2-yl)cyclopropyl)-4,4,5,5-tetramethyl-1,3,2-dioxaborolane 2l**

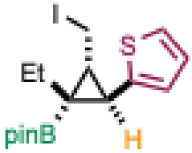

Chemical Formula: $C_{16}H_{24}BIO_2S$
Molecular Weight: 418.14

Prepared according to a literature procedure[3b] from **2l**-OH (180 mg, 350 µmol).

Yield: 63 mg (150 µmol, 43%, *dr* > 95:05 as determined by ^{1}H NMR spectroscopy) as a yellow oil.

^{1}H NMR (400 MHz, $CDCl_3$): δ 7.02 (d, *J* = 5.1 Hz, 1H), 6.82 (dd, *J* = 4.9, 3.6 Hz, 1H), 6.70 (d, *J* = 3.4 Hz, 1H), 3.59 (dd, *J* = 9.5, 6.7 Hz, 1H), 3.41 (t, *J* = 9.5 Hz, 1H), 2.45 (d, *J* = 5.6 Hz, 1H), 1.68 (dt, *J* = 9.4, 6.3

Hz, 1H), 1.37-1.11 (m, 1H), 1.21 (s, 6H), 1.20 (s, 6H), 0.99 (dt, *J* = 13.9, 7.1 Hz, 1H), 0.82 (t, *J* = 7.3 Hz, 3H) ppm.

^{13}C NMR (101 MHz, $CDCl_3$): δ 142.2, 126.7, 125.7, 123.7, 83.6, 37.5, 34.9, 25.3, 24.9, 24.1, 13.1, 10.4 ppm. *Note*: Carbon atom attached to boron is not visible due to quadrupolar relaxation.

^{11}B NMR (128 MHz, $CDCl_3$): δ 32.7 ppm.

HRMS (APCI+) m/z: calcd. for $C_{16}H_{25}BIO_2S^+$ [*M*+H]$^+$: 419.0713, found: 419.0724.

2-((1*R,2*S**,3*R**)-1-(3-Chloropropyl)-2-(iodomethyl)-3-phenylcyclopropyl)-4,4,5,5-tetramethyl-1,3,2-dioxaborolane 2i**

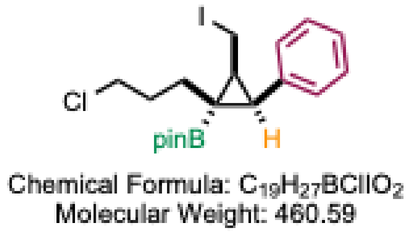

Prepared according to a literature procedure[3b] from **2i**-OH (400 mg, 1.14 mmol).

Yield: 276 mg (600 µmol, 63%, *dr* > 95:05 as determined by ^{1}H NMR spectroscopy) as a yellow oil.

^{1}H NMR (400 MHz, $CDCl_3$): δ 7.36-7.29 (m, 2H), 7.29-7.25 (m, 2H), 7.25-7.14 (m, 1H), 3.54 (t, *J* = 6.5 Hz, 1H), 3.49-3.36 (m, 2H), 3.35-3.21 (m, 1H), 2.45 (d, *J* = 8.6 Hz, 1H), 2.17-1.88 (m, 4H), 1.65-1.53 (m, 1H), 1.24 (s, 6H), 1.22 (s, 6H) ppm.

^{13}C NMR (101 MHz, $CDCl_3$) δ 135.6, 130.8, 128.5, 126.6, 83.6, 45.6, 32.7, 32.1, 30.0, 24.9, 24.5, 23.9, 4.5 ppm.
Note: Carbon atom attached to boron is not visible due to quadrupolar relaxation.

^{11}B NMR (128 MHz, $CDCl_3$): δ 32.3 ppm.

HRMS (APCI+) m/z: calcd. for $C_{19}H_{28}BClIO_2^+$ [*M*+H]$^+$: 461.0916, found: 461.0937.

1-((1*R,2*S**,3*R**)-3-(Iodomethyl)-2-methyl-2-(4,4,5,5-tetramethyl-1,3,2-dioxaborolan-2-yl)-1-(4-(trifluoromethyl)phenyl)cyclopropyl)propan-2-one 2n**

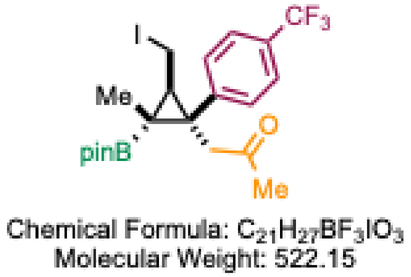

Prepared according to a literature procedure[3b] from **2n**-OH (143 mg, 347 µmol).

Yield: 125 mg (240 µmol, 69%, dr > 95:05 as determined by ^{1}H NMR spectroscopy) as a yellow oil.

^{1}H NMR (400 MHz, CDCl3): δ 7.53 (d, J = 8.2 Hz, 2H), 7.43 (d, J = 8.1 Hz, 2H), 3.46 (dd, J = 9.9, 5.9 Hz, 1H), 3.07-2.91 (m, 2H), 2.84 (t, J = 9.9 Hz, 1H), 1.91-1.82 (m, 1H), 1.90 (s, 3H), 1.25 (s, 6H), 1.22 (s, 6H), 0.93 (s, 3H) ppm.

^{13}C NMR (101 MHz, CDCl3): δ 206.8, 143.4, 132.0, 128.7 (q, J_{C-F} = 33.4 Hz), 125.37 (q, J_{C-F} = 3.7 Hz), 122.94 (q, J_{C-F} = 272.3 Hz), 83.7, 53.6, 38.6, 36.4, 30.4, 24.9, 12.5, 5.4 ppm.
Note: Carbon atom attached to boron is not visible due to quadrupolar relaxation.

^{11}B NMR (128 MHz, $CDCl_3$): δ 32.1 ppm.

HRMS (APCI+) m/z: calcd. for $C_{21}H_{28}BF_3IO_2^+$ [M+H]$^+$: 523.1123, found: 523.1125.

Mesylation/Finkelstein exchange GP1:

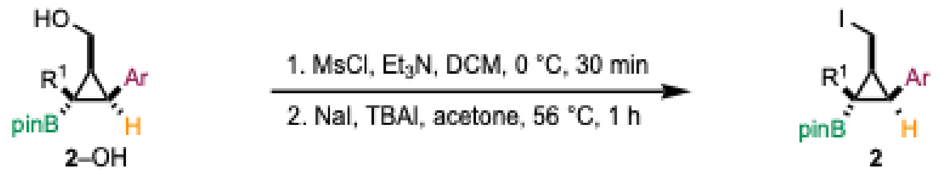

Borylated cyclopropyl carbinol 2-OH was dissolved in dry DCM (0.1M) in a flame-dried Schlenk flask. The mixture was cooled to 0 °C, Et_3N (3.0 equiv.) was added, followed by dropwise addition of MsCl (typically 1.2 equiv.). The mixture was stirred at this temperature until full consumption of starting material was indicated by TLC (typically 30 min). Satd. NH_4Cl solution was added and the mixture was allowed to come to room temperature with stirring. The layers were separated and the aqueous one was extracted with DCM. The combined organic layers were washed with satd. $NaHCO_3$, water, dried over Na_2SO_4, filtered and concentrated under reduced pressure.
The crude mesylate was dissolved in acetone (0.25M) and NaI (5 equiv.) and TBAI (0.1 equiv.) were added. The mixture was stirred at 56 °C for one hour, when it was allowed to come to room temperature and poured onto PE. The precipitate was filtered over Celite® and the filtrate was washed with satd. Na_2SO_3, water and brine, dried over Na_2SO_4, filtered and concentrated under reduced pressure to give crude iodide, which contained the desired iodide and residual impurities. To this residue was added *p*-xylene (0.25 equiv.), the mixture was dissolved in $CDCl_3$ (*ca* 1 mL), homogenized and the content of title iodide **2** was quantified by ^{1}H NMR spectroscopy (relaxation delay D1 = 6 s) using the characteristic C*H*$_2$I resonance integration (δ 3.2-3.4 ppm)[3b] to determine the yield. The crude product was subsequently re-evaporated twice from toluene, dried in vacuo, and used promptly in the ring-opening step.

2-((1*R,2*S**,3*R**)-1-ethyl-2-(iodomethyl)-3-phenylcyclopropyl)-4,4,5,5-tetramethyl-1,3,2-dioxaborolane 2a**

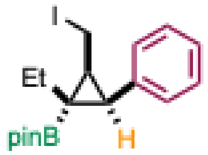

Chemical Formula: $C_{18}H_{26}BIO_2$
Molecular Weight: 412.12

Prepared according to general procedure **GP1** from 2a-OH (227 mg, 750 µmol).

Yield: 186 mg (450 µmol, 60%, *dr* > 95:05 as determined by ^{1}H NMR spectroscopy) as a yellow oil.

The analytical data are in accordance with the published values.[3b]

2-((1*R,2*S**,3*R**)-2-(Iodomethyl)-1-methyl-3-phenylcyclopropyl)-4,4,5,5-tetramethyl-1,3,2-dioxaborolane 2b**

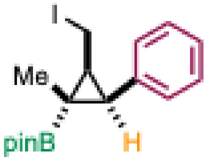

Chemical Formula: $C_{17}H_{24}BIO_2$
Molecular Weight: 398.09

Prepared according to general procedure **GP1** from **2b**-OH (185 mg, 642 µmol).

Yield: 140 mg (352 µmol, 55%, *dr* > 95:05 as determined by ^{1}H NMR spectroscopy) as a yellow oil.

^{1}H NMR (400 MHz, $CDCl_3$): δ 7.38-7.29 (m, 2H), 7.29-7.17 (m, 3H), 3.45 (dd, *J* = 10.0, 6.4 Hz, 1H), 2.99 (t, *J* = 9.7 Hz, 1H), 2.49 (d, *J* = 8.6 Hz, 1H), 2.05-1.93 (m, 1H), 1.28 (s, 6H), 1.27 (s, 6H), 0.96 (s, 3H) ppm.

^{13}C NMR (101 MHz, $CDCl_3$): δ 135.6, 131.1, 128.4, 126.5, 83.5, 32.8, 29.5, 24.8, 24.7, 9.4, 5.6 ppm.
Note: Carbon atom attached to boron is not visible due to quadrupolar relaxation.

^{11}B NMR (128 MHz, $CDCl_3$): δ 33.0 ppm.

HRMS (APCI+) m/z: calcd. for $C_{17}H_{25}BIO_2^+$ [*M*–H_2O+H]$^+$: 399.0987, found: 399.0985.

2-((1*R,2*S**,3*R**)-2-(Iodomethyl)-1-methyl-3-(*p*-tolyl)cyclopropyl)-4,4,5,5-tetramethyl-1,3,2-dioxaborolane 2c**

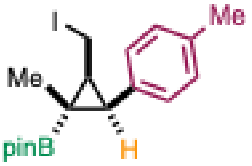

Chemical Formula: $C_{18}H_{26}BIO_2$
Molecular Weight: 412.12

Prepared according to general procedure **GP1** from **2c**-OH (227 mg, 750 µmol).

Yield: 190 mg (462 µmol, 62%, *dr* > 95:05 as determined by ^{1}H NMR spectroscopy) as a yellow oil.

^{1}H NMR (400 MHz, $CDCl_3$): δ 7.19-7.12 (m, 4H), 3.47 (dd, *J* = 9.9, 6.3 Hz, 1H), 3.01 (t, *J* = 9.7 Hz, 1H), 2.47 (d, *J* = 8.5 Hz, 1H), 2.35 (s, 3H), 2.03-1.94 (m, 1H), 1.30 (s, 6H), 1.28 (s, 6H), 0.98 (s, 3H) ppm.

^{13}C NMR (101 MHz, $CDCl_3$): δ 136.0, 132.4, 130.9, 129.2, 83.5, 32.5, 29.5, 24.8, 24.7, 21.2, 9.4, 5.8 ppm.
Note: Carbon atom attached to boron is not visible due to quadrupolar relaxation.

^{11}B NMR (128 MHz, $CDCl_3$): δ 32.8 ppm.

HRMS (APCI+) m/z: calcd. for $C_{18}H_{27}BIO_2^+$ [*M*+H]$^+$: 413.1143, found: 413.1121.

2-((1*R,2*S**,3*R**)-2-(Iodomethyl)-1-methyl-3-(4-(trifluoromethyl)phenyl)cyclopropyl)-4,4,5,5-tetramethyl-1,3,2-dioxaborolane 2d**

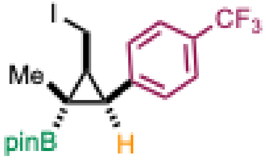

Chemical Formula: $C_{18}H_{23}BF_3IO_2$
Molecular Weight: 466.09

Prepared according to general procedure **GP1** from **2d**-OH (113 mg, 317 µmol).

Yield: 93 mg (200 µmol, 62%, *dr* > 95:05 as determined by ^{1}H NMR spectroscopy) as a yellow oil.

^{1}H NMR (400 MHz, $CDCl_3$): δ 7.57 (d, J = 8.1 Hz, 2H), 7.39 (d, J = 7.9 Hz, 2H), 3.35 (dd, J = 10.0, 6.8 Hz, 1H), 2.98 (t, J = 9.5 Hz, 1H), 2.50 (d, J = 8.6 Hz, 1H), 2.08-1.97 (m, 1H), 1.28 (s, 6H), 1.27 (s, 6H), 0.95 (s, 3H) ppm.

^{13}C NMR (101 MHz, $CDCl_3$): δ 140.0, 131.4, 129.7 (q, J_{C-F} = 63.1 Hz), 127.2 (q, J_{C-F} = 292.9 Hz), 125.4 (q, J_{C-F} = 3.7 Hz)83.8, 32.3, 29.5, 24.8, 24.8, 9.5, 4.4 ppm.
Note: Carbon atom attached to boron is not visible due to quadrupolar relaxation.

^{11}B NMR (128 MHz, $CDCl_3$): δ 32.7 ppm.

HRMS (APCI+) m/z: calcd. for $C_{18}H_{24}BF_3IO_2^+$ [*M*+H]$^+$: 467.0861, found: 467.0881.

2-((1*R,2*R**,3*S**)-2-(3-Chlorophenyl)-3-(iodomethyl)-1-methylcyclopropyl)-4,4,5,5-tetramethyl-1,3,2-dioxaborolane 2e**

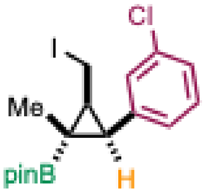

Chemical Formula: $C_{17}H_{23}BClIO_2$
Molecular Weight: 432.53

Prepared according to general procedure **GP1** from **2e**-OH (130 mg, 403 µmol).

Yield: 91 mg (210 µmol, 52%, *dr* > 95:05 as determined by ^{1}H NMR spectroscopy) as a yellow oil.

^{1}H NMR (400 MHz, $CDCl_3$): δ 7.34-7.22 (m, 2H), 7.19 (d, J = 6.2 Hz, 1H), 7.11 (s, 1H), 3.43 (dd, J = 9.9, 6.5 Hz, 1H), 3.02 (t, J = 9.7 Hz, 1H), 2.48 (d, J = 8.4 Hz, 1H), 2.08-1.96 (m, 1H), 1.32 (s, 6H), 1.30 (s, 6H), 0.99 (s, 3H) ppm.

^{13}C NMR (101 MHz, $CDCl_3$): δ 137.7, 134.2, 131.1, 129.7, 129.2, 126.8, 83.7, 32.3, 29.4, 24.8, 24.7, 9.4, 4.7 ppm.
Note: Carbon atom attached to boron is not visible due to quadrupolar relaxation.

^{11}B NMR (128 MHz, $CDCl_3$): δ 32.8 ppm.

HRMS (APCI+) m/z: calcd. for $C_{17}H_{24}BClIO_2^+$ [*M*+H]$^+$: 433.0603, found: 433.0583.

2-((1*R,2*S**,3*R**)-2-(Iodomethyl)-1-methyl-3-(naphthalen-1-yl)cyclopropyl)-4,4,5,5-tetramethyl-1,3,2-dioxaborolane 2f**

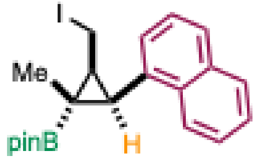

Prepared according to general procedure **GP1** from **2f**-OH (120 mg, 355 µmol).

Yield: 81 mg (180 µmol, 51%, *dr* > 95:05 as determined by ^{1}H NMR spectroscopy) as a yellow oil.

^{1}H NMR (400 MHz, $CDCl_3$): δ 8.17-8.07 (m, 1H), 7.90-7.83 (m, 1H), 7.81-7.73 (m, 1H), 7.58-7.36 (m, 4H), 3.76 (dd, J = 9.9, 5.3 Hz, 1H), 3.03 (t, J = 10.4 Hz, 1H), 2.73 (d, J = 8.4 Hz, 1H), 2.27 (ddd, J = 10.8, 8.5, 5.4 Hz, 1H), 1.37 (s, 6H), 1.35 (s, 6H), 0.90 (s, 3H) ppm.

^{13}C NMR (101 MHz, $CDCl_3$): δ 134.0, 133.9, 132.6, 128.7, 127.8, 127.4, 126.0, 125.8, 125.3, 124.9, 83.8, 31.9, 30.0, 24.9, 24.8, 9.0, 6.3 ppm.
Note: Carbon atom attached to boron is not visible due to quadrupolar relaxation.

^{11}B NMR (128 MHz, $CDCl_3$): δ 33.3 ppm.

HRMS (APCI+) m/z: calcd. for $C_{18}H_{27}BIO_2^+$ [*M*+H]$^+$: 449.1149, found: 449.1143.

2-((1*R,2*S**,3*R**)-1-Ethyl-2-(iodomethyl)-3-(perfluorophenyl)cyclopropyl)-4,4,5,5-tetramethyl-1,3,2-dioxaborolane 2k**

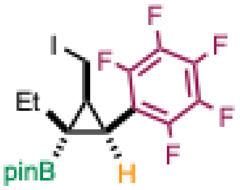

Prepared according to general procedure **GP1** from **2k**-OH (125 mg, 319 µmol).

Yield: 131 mg (260 µmol, 82%, *dr* > 95:05 as determined by ^{1}H NMR spectroscopy) as a yellow oil.

^{1}H NMR (400 MHz, $CDCl_3$): δ 3.78-3.62 (m, 1H), 2.92 (t, J = 10.5 Hz, 1H), 2.17 (td, J = 9.8, 4.1 Hz, 1H), 2.05 (d, J = 8.6 Hz, 1H), 1.62 (dq, J = 14.4, 7.3 Hz, 1H), 1.26 (s, 6H), 1.24 (s, 6H), 1.16 (t, J = 7.3 Hz, 3H), 0.99 (dt, J = 14.4, 7.1 Hz, 1H) ppm.

^{13}C NMR (101 MHz, $CDCl_3$): δ 148.1-147.7 (m), 145.7-145.1 (m), 139.2-138.7 (m), 136.7-136.2 (m), 110.9-109.0 (m), 83.9, 67.4 (t, J_{C-F} = 6.4 Hz), 37.6, 24.9, 24.5, 23.1, 19.6, 18.9, 12.7 ppm.
Note: Carbon atom attached to boron is not visible due to quadrupolar relaxation.

^{19}F NMR (377 MHz, $CDCl_3$): δ –137.02 (dd, J = 23.0, 7.2 Hz, 2F), –155.79 (t, J = 21.0 Hz, 1F), –162.16 (td, J = 22.9, 7.6 Hz, 2F) ppm.

^{11}**B NMR** (128 MHz, $CDCl_3$): δ 32.8 ppm.

HRMS (APCI+) m/z: calcd. for $C_{18}H_{22}BF_5IO_2^+$ [*M*+H]$^+$: 503.0672, found: 503.0693.

2-((1*R,2*R**,3*S**)-1-Butyl-2-(4-chlorophenyl)-3-(iodomethyl)cyclopropyl)-4,4,5,5-tetramethyl-1,3,2-dioxaborolane 2h**

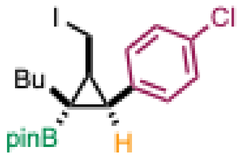

Chemical Formula: $C_{20}H_{29}BClIO_2$
Molecular Weight: 474.61

Prepared according to general procedure **GP1** from 2h-OH (118 mg, 324 µmol).

Yield: 81 mg (170 µmol, 53%, *dr* > 95:05 as determined by ^{1}H NMR spectroscopy) as a yellow oil.

^{1}**H NMR** (400 MHz, $CDCl_3$): δ 7.33 (d, *J* = 8.2 Hz, 2H), 7.27 (d, *J* = 8.3 Hz, 2H), 3.42 (dd, *J* = 9.9, 5.7 Hz, 1H), 3.09 (t, *J* = 9.5 Hz, 1H), 2.36 (d, *J* = 8.5 Hz, 1H), 2.02 (d, *J* = 5.7 Hz, 1H), 1.50 (td, *J* = 12.8, 4.0 Hz, 3H), 1.46-1.34 (m, 4H), 1.26 (s, 6H), 1.24 (s, 6H), 1.04-0.89 (m, 2H), 0.84 (t, *J* = 7.3 Hz, 3H) ppm.

^{13}**C NMR** (101 MHz, $CDCl_3$): δ 134.5, 132.4, 132.4, 128.5, 83.5, 31.9, 30.9, 29.6, 26.1, 24.9, 24.5, 23.5, 14.2, 4.7 ppm.
Note: Carbon atom attached to boron is not visible due to quadrupolar relaxation.

^{11}**B NMR** (128 MHz, $CDCl_3$): δ 32.3 ppm.

HRMS (APCI+) m/z: calcd. for $C_{20}H_{30}BClIO_2^+$ [*M*+H]$^+$: 475.1067, found: 475.1072.

2-((1*R,2*S**,3*R**)-1-Butyl-2-(iodomethyl)-3-(4-methoxyphenyl)cyclopropyl)-4,4,5,5-tetramethyl-1,3,2-dioxaborolane 2g**

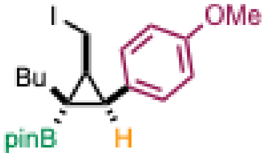

Chemical Formula: $C_{21}H_{32}BIO_3$
Molecular Weight: 470.20

Prepared according to general procedure **GP1** from 2g-OH (144 mg, 400 µmol).

Yield: 47 mg (100 µmol, 25%, *dr* > 95:05 as determined by ^{1}H NMR spectroscopy) as a yellow oil.

^{1}**H NMR** (400 MHz, $CDCl_3$): δ 7.32 (d, *J* = 8.2 Hz, 2H), 7.07 (d, *J* = 8.5 Hz, 2H), 3.80 (s, 3H), 3.42 (dd, *J* = 9.8, 5.8 Hz, 1H), 3.17 (t, *J* = 9.3 Hz, 1H), 2.37 (d, *J* = 8.5 Hz, 1H), 2.01 (td, *J* = 8.8, 5.8 Hz, 1H), 1.54 (ddd, *J* = 13.9, 7.2, 4.3 Hz, 3H), 1.48-1.36 (m, 4H), 1.28 (s, 6H), 1.27 (s, 6H), 1.07-0.75 (m, 2H) ppm.

^{13}**C NMR** (101 MHz, $CDCl_3$): δ 158.2, 132.0, 127.9, 113.7, 83.3, 55.3, 32.0, 30.8, 29.7, 26.1, 24.9, 24.5, 23.6, 14.2, 5.5 ppm.
Note: Carbon atom attached to boron is not visible due to quadrupolar relaxation.

^{11}**B NMR** (128 MHz, $CDCl_3$): δ 30.7 ppm.

HRMS (APCI+) m/z: calcd. for $C_{21}H_{32}BIO_2^+$ [*M*–HI+H]$^+$: 343.2439, found: 343.2451.

2-((1*R,2*S**,3*R**)-1-Cyclohexyl-2-(iodomethyl)-3-phenylcyclopropyl)-4,4,5,5-tetramethyl-1,3,2-dioxaborolane 2j**

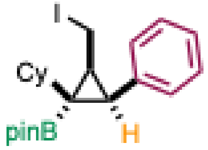

Prepared according to general procedure **GP1** from **2j**-OH (137 mg, 384 μmol).

Yield: 117 mg (150 μmol, 65%, *dr* > 95:05 as determined by ^{1}H NMR spectroscopy) as a yellow oil.

^{1}**H NMR** (400 MHz, $CDCl_3$): δ 7.32 (d, *J* = 7.4 Hz, 2H), 7.16 (ddd, *J* = 21.5, 10.3, 4.4 Hz, 3H), 3.69 (dd, *J* = 9.8, 5.5 Hz, 1H), 3.32 (t, *J* = 10.0 Hz, 1H), 2.43 (d, *J* = 8.7 Hz, 1H), 1.93 (td, *J* = 10.1, 5.6 Hz, 1H), 1.77-1.26 (m, 6H), 1.25-0.90 (m, 5H), 1.15 (s, 12H) ppm.

^{13}**C NMR** (101 MHz, $CDCl_3$): δ 137.1, 130.1, 128.0, 126.0, 83.1, 35.9, 32.5, 32.0, 31.3, 31.1, 27.1, 26.9, 26.3, 24.8, 24.7, 6.1 ppm.
Note: Carbon atom attached to boron is not visible due to quadrupolar relaxation.

^{11}**B NMR** (128 MHz, $CDCl_3$): δ 32.3 ppm.

HRMS (APCI+) m/z: calcd. for $C_{22}H_{33}BIO_2^+$ [*M*+H]$^+$: 467.1618, found: 467.1620.

((1*R,2*S**,3*R**)-2-Methyl-3-(2-methylallyl)-2-(4,4,5,5-tetramethyl-1,3,2-dioxaborolan-2-yl)-3-(4-(trifluoromethyl)phenyl)cyclopropyl)methanol 2m**

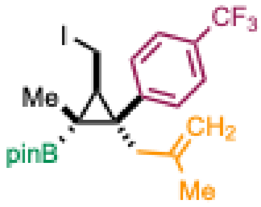

Prepared according to general procedure **GP1** from **2m**-OH (123 mg, 300 μmol).

Yield: 120 mg (230 μmol, 77%, *dr* > 95:05 as determined by ^{1}H NMR spectroscopy) as a colorless oil.

^{1}**H NMR** (400 MHz, $CDCl_3$): δ 7.52 (d, *J* = 7.9 Hz, 2H), 7.26 (d, *J* = 8.4 Hz, 2H), 4.58 – 4.49 (m, 1H), 4.32 (s, 1H), 3.47 (dd, *J* = 9.9, 6.1 Hz, 1H), 2.93 (t, *J* = 9.9 Hz, 1H), 2.51 – 2.35 (m, 2H), 2.07 (dd, *J* = 9.9, 6.2 Hz, 1H), 1.62 (s, 3H), 1.28 (s, 6H), 1.26 (s, 6H), 0.84 (s, 3H) ppm.

^{13}**C NMR** (101 MHz, $CDCl_3$): δ 143.0, 131.6, 129.0, 128.5 (q, J_{C-F} = 32.3 Hz), 125.1 (q, J_{C-F} = 3.8 Hz), 124.4 (q, J_{C-F} = 272.3 Hz), 113.2, 83.8, 48.5, 42.6, 36.1, 25.1, 24.8, 23.2, 12.6, 6.0 ppm.

Note: Carbon atom attached to boron is not visible due to quadrupolar relaxation.

^{19}F NMR (377 MHz, $CDCl_3$): δ –62.3 ppm.

^{11}B NMR (128 MHz, $CDCl_3$): δ 32.4 ppm.

HRMS (APCI+) m/z: calcd. for $C_{22}H_{30}BF_3IO_3^+$ [*M*+H_2O+H]$^+$: 537.1279, found: 537.1272.

3. Ring opening

3.1 Optimization of the ring opening/silylation

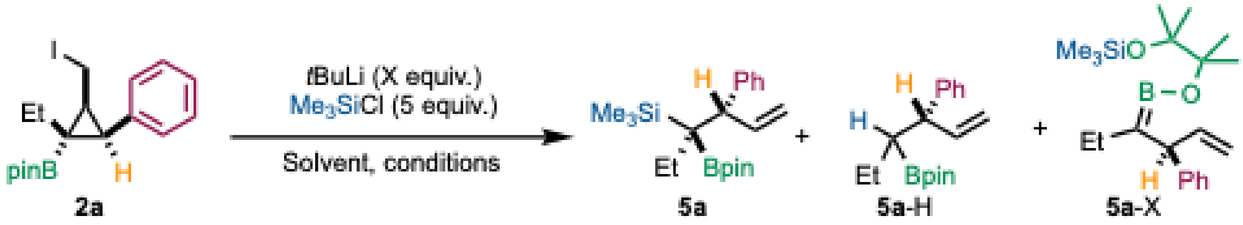

Entry	*t*-BuLi (equiv.)	Solvent	Conditions	5a (%)	5a (*dr*)[a]	5a-H[b] (%)	5a-X[c] (%)
**1**[d]	2.2	Pe/Et_2O 3:2	–110°C to r.t., 16 h	30	>95:05	traces	20
**2**	2.2	Et_2O/THF/Pentane 4:4:1	–110 to –78°C, 1 h	**70**	**>95:05**	28	0
**3**[d]	2.2	Pe/Et_2O/TMEDA 6:4:1	–110°C to –78 °C, 1 h	31	80:20	46	0
**4**	2.2	THF	–95 to–78°C, 1 h	25	96:04	43	0
**5**[d]	2.2	THF	–78°C, 1 h	**87**	84:16	13	0
**6**[d]	2.5	THF	–95 to–78°C, 1 h	**65**	**96:04**	13	0
**7**	4	THF	–95 to–78°C, 1 h	43	>95:05	0	21
**8**[e]	2.2	Et_2O	–95 °C to –30°C, 90 min	30	>95:05	12	27
**9**[e,f]	2.2	Et_2O	–95 °C to –30°C, 90 min	36	>95:05	<20	30
**10**[e]	2.5	Et_2O	–95 °C to –30°C, 90 min	**50**	**>95:05**	12	27
**11**[e]	4	Et_2O	–95 °C to –30°C, 90 min	38	>95:05	0	23
**12**[e,g]	1.3	Et_2O	–95 °C to –30°C, 90 min	20	>95:05	62	traces

Table S1. Optimization of the lithium/halogen-exchange mediated ring opening/silylation. Yields were determined by ^{1}H NMR spectroscopy of the crude mixture using an internal standard. [a]As determined by GC chromatography or by ^{1}H and ^{29}Si NMR analysis of the crude mixture. [b]The analytical data matched a sample synthesized independently on preparative scale (vide infra). [c] Structure tentatively assigned based on a characteristic ^{11}B and ^{13}C NMR resonances. [d]Internal quench conditions. [e] Inverse addition (**2a** into a solution of *t*BuLi). [f] With 2.5 equiv. TMSCl. [g] Starting material **2a** fully consumed. *Note:* Using a different organolithium reagent than *t*BuLi resulted in either mixture of Li/I exchange and elimination (*n*BuLi), 1,2-metalate rearrangement (MeLi) or no reaction (MesLi).

3.2 Scope of ring opening/silylation

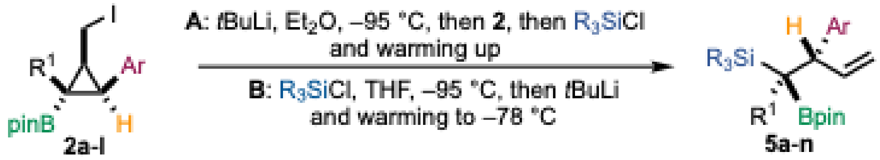

General procedure for Li/halogen exchange-mediated ring opening/silylation-GP2 (conditions A): A flame-dried Schlenk flask was charged with dry Et_2O (0.2M with respect to **2**, typically 1 mL) under argon and a solution of *t*BuLi (2.5 equiv., typically 1.4M in pentane, freshly titrated before use against *N*-benzyl benzamide)[4] was added at –78 °C. The mixture was cooled to –95 °C with good stirring and a solution of iodide **2** (typically 200 µmol) in dry Et_2O (0.2M) was added via a cannula. A bright yellow precipitate formed. R_3SiCl (5 equiv.) was added and the mixture was stirred with warming up to the required temperature while monitored by TLC against the proton-quench product **5**-H (R*f* ≈ 0.45 vs. 0.70 for silylated compounds **5**). Completion was accompanied by decoloration and was typically reached after 30-40 min of stirring at -78 to -30 °C. The reaction mixture was subsequently quenched with MeOH (10 equiv.), diluted with Et_2O and filtered over a short plug of silica gel. The crude residue was purified by flash column chromatography (silica gel, gradient PE/Et_2O 999:1 to 99:1; majority of compounds elute at 997:3) to obtain title compounds **5**.
General procedure for Li/halogen echange-mediated ring opening/silylation-GP3 (conditions B): A flame-dried Schlenk flask was charged with a solution of iodide **2** (typically 200 µmol) in dry THF (0.1M) and the solution was cooled to –95 °C with good stirring. R_3SiCl (5 equiv.) was added, followed by a dropwise addition of a solution of *t*BuLi (2.5 equiv., typically 1.4M in pentane, freshly titrated before use against *N*-benzyl benzamide).[4] The mixture was stirred with warming to –78 °C for 30-40 min. Typically, the reaction mixture turned briefly yellow to red and heterogeneous immediately after the addition of *t*BuLi and the color quickly disappeared as the reaction reached completion. The reaction mixture was subsequently quenched with MeOH (10 equiv.), diluted with Et_2O and filtered over a short plug of silica gel. The crude residue was purified by flash column chromatography (silica gel, gradient PE/Et_2O 999:1 to 99:1; majority of compounds elute at 997:3) to obtain title compounds **5**.

Trimethyl((3*S,4*S**)-4-phenyl-3-(4,4,5,5-tetramethyl-1,3,2-dioxaborolan-2-yl)hex-5-en-3-yl)silane 5a**

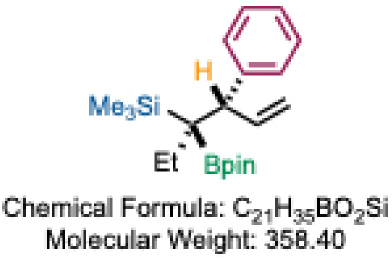

Prepared according to general procedure **GP2** from **2a** (42 mg, 100 µmol) and Me_3SiCl (63 µL, 500 µmol).

Yield: 15 mg (43 µmol, 42%, *dr* > 95:05 as determined by ^1H NMR spectroscopy) as a colorless oil.

Prepared according to general procedure **GP3** from **2a** (107 mg, 260 µmol) and Me_3SiCl (165 µL, 1.3 mmol).

Yield: 58 mg (162 µmol, 62%) as a partially separable mixture of diastereomers (*dr* 96:04 as determined by ^1H NMR spectroscopy) as a colorless oil.

Prepared according to general procedure **GP3** from **2a** (730 mg, 1.77 mmol).

Yield: 385mg (1.03 mmol, 58%) as a partially separable mixture of diastereomers (*dr* 94:06 as determined by ^{1}H NMR spectroscopy) as a colorless oil.

R*f* = 0.65 (PE/Et_2O 40:1).

Major diastereomer:
^{1}H NMR (400 MHz, $CDCl_3$): δ 7.49-7.36 (m, 3H), 7.36-7.23 (m, 2H), 7.23-7.11 (m, 1H), 6.79-6.61 (m, 1H), 5.04-4.89 (m, 2H), 3.71 (d, *J* = 8.9 Hz, 1H), 1.82 (dq, *J* = 14.8, 7.4 Hz, 1H), 1.65-1.49 (m, 1H), 1.33 (s, 6H), 1.30 (s, 6H), 0.99 (t, *J* = 7.5 Hz, 3H), 0.07 (s, 9H) ppm.

^{13}C NMR (101 MHz, $CDCl_3$): δ 145.3, 144.1, 129.1, 128.1, 126.0, 114.2, 83.0, 52.7, 25.5, 23.3, 12.5, -0.0 ppm.
Note: Carbon atom attached to boron is not visible due to quadrupolar relaxation.

^{29}Si NMR (80 MHz, $CDCl_3$) δ 5.0 ppm.

Minor diastereomer:
^{1}H NMR (400 MHz, $CDCl_3$): δ 7.49-7.36 (m, 3H), 7.36-7.23 (m, 2H), 7.23-7.11 (m, 1H), 6.79-6.61 (m, 1H), 5.04-4.89 (m, 2H), 3.71 (d, *J* = 8.9 Hz, 1H), 1.82 (dq, *J* = 14.8, 7.4 Hz, 1H), 1.65-1.49 (m, 1H), 1.33 (s, 6H), 1.30 (s, 6H), 0.99 (t, *J* = 7.5 Hz, 3H), 0.07 (s, 9H) ppm.

^{13}C NMR (101 MHz, $CDCl_3$): δ 143.6, 139.8, 128.5, 128.1, 126.1, 115.8, 83.2, 56.8, 28.5, 27.8, 24.7, 24.6, 18.2 ppm.
Note: Carbon atom attached to boron is not visible due to quadrupolar relaxation.

^{29}Si NMR (80 MHz, $CDCl_3$) δ 4.7 ppm.

^{11}B NMR (128 MHz, $CDCl_3$): δ 34.3 ppm.

HRMS (APCI+) m/z: calcd. for $C_{21}H_{36}BO_2Si^+$ [*M*+H]$^+$: 359.2578, found: 359.2572.

Dimethyl(phenyl)((3*S,4*S**)-4-phenyl-3-(4,4,5,5-tetramethyl-1,3,2-dioxaborolan-2-yl)hex-5-en-3-yl)silane 5b**

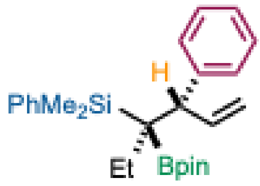

Chemical Formula: $C_{26}H_{37}BO_2Si$
Molecular Weight: 420.48

Prepared according to general procedure **GP3** from **2a** (62 mg, 150 µmol) and Me_3SiCl (128 µL, 750 µmol).

Yield: 45 mg (107 µmol, 71%, *dr* > 95:05 as determined by ^{1}H NMR spectroscopy) as a colorless oil.

R*f* = 0.55 (PE/Et_2O 40:1).

^{1}H NMR (400 MHz, $CDCl_3$): δ 7.75-7.50 (m, 2H), 7.43-7.05 (m, 8H), 6.69 (dt, *J* = 17.4, 9.4 Hz, 1H), 4.95 (dd, *J* = 27.4, 13.6 Hz, 2H), 3.68 (d, *J* = 8.5 Hz, 1H), 1.87-1.58 (m, 2H), 1.32 (s, 6H), 1.29 (s, 6H), 0.86 (t, *J* = 7.4 Hz, 3H), 0.34 (s, 3H), 0.30 (s, 3H) ppm.

^{13}C NMR (101 MHz, $CDCl_3$) δ 145.2, 144.2, 139.2, 135.2, 129.4, 128.6, 128.1, 127.3, 126.1, 114.6, 83.2, 53.3, 25.9, 25.4, 24.1, 12.9, -1.2 ppm.
Note: Carbon atom attached to boron is not visible due to quadrupolar relaxation.

^{29}Si NMR (80 MHz, $CDCl_3$) δ 0.2 ppm.

^{11}B NMR (128 MHz, $CDCl_3$): δ 34.2 ppm.

HRMS (APCI+) m/z: calcd. for $C_{26}H_{38}BO_2Si^+$ $[M+H]^+$: 421.2740, found: 421.2729.

Trimethyl((2*S,3*S**)-3-phenyl-2-(4,4,5,5-tetramethyl-1,3,2-dioxaborolan-2-yl)pent-4-en-2-yl)silane 5c**

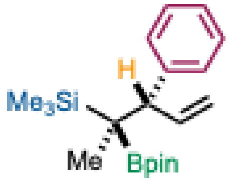

Chemical Formula: $C_{20}H_{33}BO_2Si$
Molecular Weight: 344.38

Prepared according to general procedure **GP2** from **2b** (70 mg, 175 µmol) and Me_3SiCl (67 µL, 525 µmol).

Yield: 40 mg (116 µmol, 66%, *dr* > 95:05 as determined by ^{1}H NMR spectroscopy) as a colorless oil.

R*f* = 0.72 (PE/Et_2O 40:1).

^{1}H NMR (400 MHz, $CDCl_3$): δ 7.33-7.21 (m, 4H), 7.17-7.08 (m, 1H), 6.53 (dt, J = 17.2, 9.6 Hz, 1H), 5.09-4.83 (m, 2H), 3.45 (d, J = 9.2 Hz, 1H), 1.27 (s, 6H), 1.23 (s, 6H), 1.03 (s, 3H), -0.07 (s, 9H) ppm.

^{13}C NMR (101 MHz, $CDCl_3$): δ 144.9, 142.6, 129.1, 128.1, 126.1, 114.6, 83.1, 54.8, 25.6, 25.4, 14.8, -1.8 ppm.
Note: Carbon atom attached to boron is not visible due to quadrupolar relaxation.

^{29}Si NMR (80 MHz, $CDCl_3$) δ 5.7 ppm.

^{11}B NMR (128 MHz, $CDCl_3$): δ 35.0 ppm.

HRMS (APCI+) m/z: calcd. for $C_{20}H_{34}BO_2Si^+$ $[M+H]^+$: 345.2416, found: 345.2399.

Trimethyl((2*S,3*S**)-2-(4,4,5,5-tetramethyl-1,3,2-dioxaborolan-2-yl)-3-(*p*-tolyl)pent-4-en-2-yl)silane 5d**

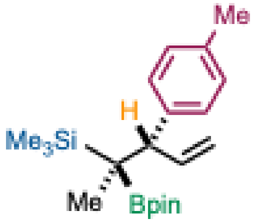

Chemical Formula: $C_{21}H_{35}BO_2Si$
Molecular Weight: 358.40

Prepared according to general procedure **GP2** from **2c** (66 mg, 160 µmol) and Me_3SiCl (61 µL, 480 µmol).

Yield: 39 mg (109 µmol, 68%, *dr* > 95:05 as determined by ^1H NMR spectroscopy) as a colorless oil.

R*f* = 0.75 (PE/Et_2O 40:1).

^{1}H NMR (400 MHz, $CDCl_3$): δ 7.16 (d, *J* = 7.8 Hz, 2H), 7.05 (d, *J* = 7.8 Hz, 2H), 6.52 (dt, *J* = 17.2, 9.7 Hz, 1H), 5.04-4.89 (m, 2H), 3.42 (d, *J* = 9.3 Hz, 1H), 2.30 (s, 3H), 1.27 (s, 6H), 1.24 (s, 6H), 1.02 (s, 3H), -0.05 (s, 9H) ppm.

^{13}C NMR (101 MHz, $CDCl_3$): δ 142.8, 142.0, 135.5, 128.9, 128.8, 114.3, 83.0, 54.5, 25.6, 25.4, 21.1, 14.9, -1.8 ppm.
Note: Carbon atom attached to boron is not visible due to quadrupolar relaxation.

^{29}Si NMR (80 MHz, $CDCl_3$) δ 5.7 ppm.

^{11}B NMR (128 MHz, $CDCl_3$): δ 34.5 ppm.

HRMS (APCI+) m/z: calcd. for $C_{21}H_{36}BO_2Si^+$ [*M*+H]$^+$: 359.2572, found: 359.2594.

Dimethyl(phenyl)((2*S,3*S**)-2-(4,4,5,5-tetramethyl-1,3,2-dioxaborolan-2-yl)-3-(*p*-tolyl)pent-4-en-2-yl)silane 5e**

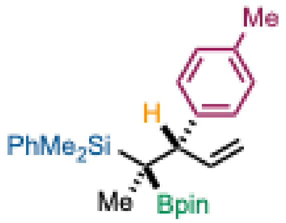

Chemical Formula: $C_{26}H_{37}BO_2Si$
Molecular Weight: 420.48

Prepared according to general procedure **GP2** from **2c** (72 mg, 175 µmol) and Me_3SiCl (105 µL, 613 µmol).

Yield: 43 mg (102 µmol, 58%, *dr* > 95:05 as determined by ^1H NMR spectroscopy) as a colorless oil.

R*f* = 0.55 (PE/Et_2O 40:1).

^{1}H NMR (400 MHz, $CDCl_3$): δ 7.36 (d, *J* = 7.2 Hz, 2H), 7.18-7.09 (m, 3H), 6.87 (d, *J* = 8.0 Hz, 2H), 6.81 (d, *J* = 7.9 Hz, 2H), 6.58-6.32 (m, 1H), 4.79 (dd, *J* = 20.5, 13.6 Hz, 2H), 3.13 (d, *J* = 9.2 Hz, 1H), 2.10 (s, 3H), 1.07 (s, 6H), 1.05 (s, 6H), 0.90 (s, 3H), 0.15 (s, 3H), 0.07 (s, 3H) ppm.

^{13}C NMR (101 MHz, $CDCl_3$): δ 142.7, 142.0, 138.1, 135.5, 135.2, 128.9, 128.8, 128.7, 127.3, 114.8, 83.1, 54.6, 25.7, 25.5, 21.1, 15.8, -2.5, -2.6 ppm.
Note: Carbon atom attached to boron is not visible due to quadrupolar relaxation.

^{29}Si NMR (80 MHz, $CDCl_3$) δ 0.4 ppm.

^{11}B NMR (128 MHz, $CDCl_3$): δ 34.5 ppm.

HRMS (APCI+) m/z: calcd. for $C_{26}H_{38}BO_2Si^+$ [*M*+H]$^+$: 421.2729, found: 421.2725.

Trimethyl((2*S,3*S**)-2-(4,4,5,5-tetramethyl-1,3,2-dioxaborolan-2-yl)-3-(4-(trifluoromethyl)phenyl)pent-4-en-2-yl)silane 5f**

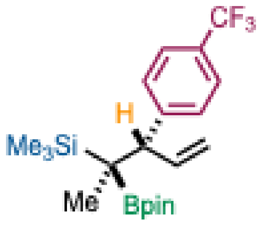

Chemical Formula: $C_{21}H_{32}BF_3O_2Si$
Molecular Weight: 412.38

Prepared according to general procedure **GP2** from **2d** (93 mg, 200 µmol) and Me_3SiCl (76 µL, 600 µmol).

Yield: 41 mg (99 µmol, 50%, *dr* > 95:05 as determined by ^{1}H NMR spectroscopy) as a colorless oil.

R*f* = 0.72 (PE/Et_2O 40:1).

^{1}H NMR (400 MHz, $CDCl_3$): δ 7.49 (d, *J* = 8.1 Hz, 2H), 7.39 (d, *J* = 7.9 Hz, 2H), 6.56 (dt, *J* = 18.2, 9.5 Hz, 1H), 5.01 (dd, *J* = 13.5, 2.5 Hz, 2H), 3.44 (d, *J* = 9.4 Hz, 1H), 1.27 (s, 6H), 1.23 (s, 6H), 0.99 (s, 3H), -0.03 (s, 9H) ppm.

^{13}C NMR (101 MHz, $CDCl_3$): δ 149.4, 141.9, 129.3, 128.3 (q, $J_{C\text{-}F}$ = 32.1 Hz), 125.0 (q, $J_{C\text{-}F}$ = 3.7 Hz), 124.5 (d, $J_{C\text{-}F}$ = 271.8 Hz),115.4, 83.2, 55.2, 25.5, 25.4, 15.3, -1.8 ppm.
Note: Carbon atom attached to boron is not visible due to quadrupolar relaxation.

^{29}Si NMR (80 MHz, $CDCl_3$): δ 6.3 ppm.

^{19}F NMR (377 MHz, $CDCl_3$): δ –62.2 ppm.

^{11}B NMR (128 MHz, $CDCl_3$): δ 35.0 ppm.

HRMS (APCI+) m/z: calcd. for $C_{21}H_{33}BF_3O_2Si^+$ [*M*+H]$^+$: 413.2289, found: 413.2283.

((2*S,3*S**)-3-(3-Chlorophenyl)-2-(4,4,5,5-tetramethyl-1,3,2-dioxaborolan-2-yl)pent-4-en-2-yl)trimethylsilane 5g**

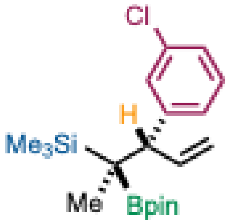

Chemical Formula: $C_{20}H_{32}BClO_2Si$
Molecular Weight: 378.82

Prepared according to general procedure **GP2** from **2e** (108 mg, 250 µmol) and Me_3SiCl (159 µL, 1.25 mmol).

Yield: 46 mg (121 µmol, 49%, *dr* > 95:05 as determined by ^{1}H NMR spectroscopy) as a colorless oil.

R*f* = 0.65 (PE/Et_2O 40:1).

^{1}H NMR (400 MHz, $CDCl_3$): δ 7.38-7.34 (m, 1H), 7.20-7.06 (m, 3H), 6.66-6.45 (m, 1H), 5.06-4.91 (m, 2H), 3.32 (d, *J* = 9.5 Hz, 1H), 1.28 (s, 6H), 1.23 (s, 6H), 0.97 (s, 3H), -0.01 (s, 9H) ppm.

^{13}C NMR (101 MHz, $CDCl_3$): δ 147.6, 142.2, 134.0, 129.2, 128.7, 127.4, 126.2, 115.1, 83.2, 55.4, 25.6, 25.4, 15.7, -1.7 ppm.
Note: Carbon atom attached to boron is not visible due to quadrupolar relaxation.

^{29}Si NMR (80 MHz, $CDCl_3$): δ 6.2 ppm.

^{11}B NMR (128 MHz, $CDCl_3$): δ 35.0 ppm.

HRMS (APCI+) m/z: calcd. for $C_{14}H_{20}ClSi^+$ [*M*–BPin+H]$^+$: 251.1023, found: 251.0991.

Trimethyl((2*S,3*S**)-3-(naphthalen-1-yl)-2-(4,4,5,5-tetramethyl-1,3,2-dioxaborolan-2-yl)pent-4-en-2-yl)silane 5h**

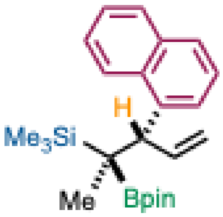

Chemical Formula: $C_{24}H_{35}BO_2Si$
Molecular Weight: 394.44

Prepared according to general procedure **GP3** from **2f** (58 mg, 130 µmol) and Me_3SiCl (82 µL, 650 µmol).

Yield: 33 mg (84 µmol, 64%) as an inseparable mixture of diastereomers (*dr* 77:23 as determined by ^{1}H NMR spectroscopy) as a colorless oil.

R*f* = 0.60 (PE/Et_2O 40:1).

Major diastereomer:
^{1}H NMR (400 MHz, $CDCl_3$): δ 8.27 (d, *J* = 8.4 Hz, 1H), 7.84 (d, *J* = 7.9 Hz, 1H), 7.78 (d, *J* = 7.0 Hz, 1H), 7.76-7.54 (m, 1H), 7.46 (ddd, *J* = 28.1, 14.6, 7.1 Hz, 3H), 6.77 (dt, *J* = 17.7, 9.6 Hz, 1H), 5.26-4.91 (m, 2H), 4.42 (d, *J* = 9.4 Hz, 1H), 1.34 (s, 6H), 1.29 (s, 6H), 0.92 (s, 3H), 0.10 (s, 9H) ppm.

^{13}C NMR (101 MHz, $CDCl_3$): δ 143.4, 142.7, 134.0, 132.4, 129.1, 126.4, 125.6, 125.4, 125.2 (2C), 123.5, 115.1, 114.1, 83.1, 47.8, 25.5, 25.5, 16.3, -1.5 ppm.
Note: Carbon atom attached to boron is not visible due to quadrupolar relaxation.

^{29}Si NMR (80 MHz, $CDCl_3$): δ 7.1 ppm.

Minor diastereomer:
^{1}H NMR (400 MHz, $CDCl_3$): δ 8.33 (d, *J* = 8.5 Hz, 1H), 7.78 (d, *J* = 7.0 Hz, 2H), 7.74-7.61 (m, 1H), 7.55-7.35 (m, 3H), 6.32 (dt, *J* = 17.1, 9.2 Hz, 1H), 5.06 (d, *J* = 17.0 Hz, 1H), 5.03-4.91 (m, 1H), 4.76 (d, *J* = 8.6 Hz, 1H), 1.44 (s, 3H), 0.73 (s, 6H), 0.66 (s, 6H), 0.16 (s, 9H) ppm.

^{13}C NMR (101 MHz, $CDCl_3$): δ 143.4, 142.0, 141.0, 134.2, 131.8, 128.6, 126.5, 125.8, 125.4, 125.2, 125.1, 125.0, 114.1, 82.8, 44.2, 25.4, 24.3, 13.8, -1.5 ppm.
Note: Carbon atom attached to boron is not visible due to quadrupolar relaxation.

^{29}Si NMR (80 MHz, $CDCl_3$): δ 6.4 ppm.

[11]**B NMR** (128 MHz, $CDCl_3$): δ 34.4 ppm.

HRMS (APCI+) m/z: calcd. for $C_{24}H_{36}BO_2Si^+$ [M+H]$^+$: 395.2578, found: 395.2583.

((3*S,4*S**)-3-(4-Methoxyphenyl)-4-(4,4,5,5-tetramethyl-1,3,2-dioxaborolan-2-yl)oct-1-en-4-yl)trimethylsilane 5i**

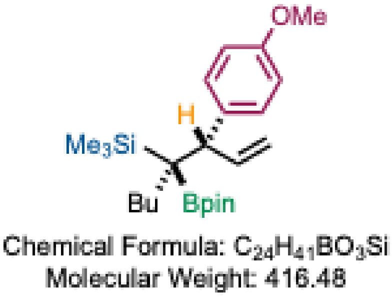

Prepared according to general procedure **GP3** from **2g** (75 mg, 160 µmol) and Me_3SiCl (61 µL, 480 µmol).

Yield: 40 mg (96 µmol, 60%) as a partially separable mixture of diastereomers (*dr* 96:04 as determined by ^{1}H NMR spectroscopy) as a colorless oil.

R*f* = 0.55 (PE/Et_2O 40:1).

Major diastereomer:
[1]**H NMR** (400 MHz, $CDCl_3$): δ 7.25 (d, *J* = 7.8 Hz, 2H), 6.79 (d, *J* = 8.3 Hz, 2H), 6.64 (dt, *J* = 18.1, 9.0 Hz, 1H), 4.96-4.91 (m, 1H), 4.91-4.86 (m, 1H), 3.78 (s, 3H), 3.61 (d, *J* = 9.0 Hz, 1H), 1.70-1.61 (m, 1H), 1.49-1.30 (m, 3H), 1.28 (s, 6H), 1.25 (s, 6H), 1.23-1.09 (m, 2H), 0.84 (t, *J* = 7.3 Hz, 3H), 0.03 (s, 9H) ppm.

[13]**C NMR** (101 MHz, $CDCl_3$): δ 157.8, 144.4, 137.6, 129.9, 113.8, 113.4, 82.9, 55.3, 51.9, 30.6, 29.7, 25.6, 25.5, 24.1, 14.3, 0.0 ppm.
Note: Carbon atom attached to boron is not visible due to quadrupolar relaxation.

[29]**Si NMR** (80 MHz, $CDCl_3$): δ 4.7 ppm.

Minor diastereomer:
[1]**H NMR** (400 MHz, $CDCl_3$): δ 7.25 (d, *J* = 7.8 Hz, 2H), 6.79 (d, *J* = 8.3 Hz, 2H 6.41 (dt, *J* = 18.5, 9.6 Hz, 1H), 5.00-4.92 (m, 2H), 3.79 (s, 3H), 3.70 (d, *J* = 9.6 Hz, 1H), 1.88 (dt, *J* = 10.4, 6.4 Hz, 1H), 1.49-1.30 (m, 3H), 1.20 (s, 6H), 1.11 (s, 6H), 1.23-1.09 (m, 2H), 0.88 (t, *J* = 7.4 Hz, 3H), 0.04 (s, 9H) ppm.

[13]**C NMR** (101 MHz, $CDCl_3$): δ 157.8, 144.4, 137.6, 130.5, 114.5, 113.2, 82.7, 55.2, 52.1, 30.6, 29.7, 25.7, 25.1, 23.7, 14.3, 0.5 ppm.
Note: Carbon atom attached to boron is not visible due to quadrupolar relaxation.

[29]**Si NMR** (80 MHz, $CDCl_3$): δ 4.6 ppm.

[11]**B NMR** (128 MHz, $CDCl_3$): δ 35.4 ppm.

HRMS (APCI+) m/z: calcd. for $C_{24}H_{42}BO_3Si^+$ [M+H]$^+$: 417.2991, found: 417.3012.

((3*S,4*S**)-3-(4-Chlorophenyl)-4-(4,4,5,5-tetramethyl-1,3,2-dioxaborolan-2-yl)oct-1-en-4-yl)trimethylsilane 5j**

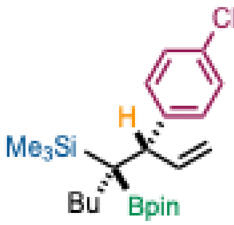

Chemical Formula: $C_{23}H_{38}BClO_2Si$
Molecular Weight: 420.90

Prepared according to general procedure **GP3** from **2h** (55 mg, 116 µmol) and Me_3SiCl (44 µL, 348 µmol).

Yield: 34 mg (81 µmol, 70%) as a partially separable mixture of diastereomers (*dr* 97:03 as determined by ^{1}H NMR spectroscopy) as a colorless oil.

R*f* = 0.65 (PE/Et_2O 40:1).

Major diastereomer:
^{1}H NMR (400 MHz, $CDCl_3$): δ 7.30 (d, *J* = 8.5 Hz, 2H), 7.20 (d, *J* = 8.1 Hz, 2H), 6.64 (dt, *J* = 18.2, 9.3 Hz, 1H), 4.97-4.95 (m, 1H), 4.92 (d, *J* = 3.7 Hz, 1H), 3.60 (d, *J* = 9.2 Hz, 1H), 1.72-1.59 (m, 1H), 1.45-1.34 (m, 1H), 1.27 (s, 6H), 1.24 (s, 6H), 1.34-1.03 (m, 4H), 0.83 (t, *J* = 7.2 Hz, 3H), 0.05 (s, 9H) ppm.

^{13}C NMR (101 MHz, $CDCl_3$): δ 144.2, 143.6, 131.6, 130.4, 128.2, 114.6, 83.0, 52.34, 30.7, 29.5, 25.6, 25.4, 24.0, 14.3, 0.1 ppm.
Note: Carbon atom attached to boron is not visible due to quadrupolar relaxation.

^{29}Si NMR (80 MHz, $CDCl_3$): δ 5.1 ppm.

Minor diastereomer:
^{1}H NMR (400 MHz, $CDCl_3$): δ 7.34 (d, *J* = 8.3 Hz, 2H), 7.18 (d, *J* = 7.9 Hz, 2H), 6.38 (dt, *J* = 17.1, 9.9 Hz, 1H), 5.03-4.95 (m, 2H), 3.72 (d, *J* = 9.6 Hz, 1H), 1.72-1.59 (m, 1H), 1.45-1.34 (m, 1H), 1.19 (s, 6H), 1.11 (s, 6H), 1.34-1.03 (m, 4H), 0.85 (t, *J* = 7.4 Hz, 3H), 0.02 (s, 9H) ppm.

^{13}C NMR (101 MHz, $CDCl_3$) δ 144.2, 143.6, 131.6, 131.0, 127.9, 115.3, 83.0, 52.28, 30.7, 29.5, 25.8, 25.1, 24.0, 14.3, 0.4 ppm.
Note: Carbon atom attached to boron is not visible due to quadrupolar relaxation.

^{29}Si NMR (80 MHz, $CDCl_3$): δ 4.8 ppm.

^{11}B NMR (128 MHz, $CDCl_3$): δ 35.0 ppm.

HRMS (APCI+) m/z: calcd. for $C_{23}H_{39}BClO_2Si^+$ $[M+H]^+$: 421.2495, found: 421.2518.

((3*S,4*S**)-7-Chloro-3-phenyl-4-(4,4,5,5-tetramethyl-1,3,2-dioxaborolan-2-yl)hept-1-en-4-yl)trimethylsilane 5k**

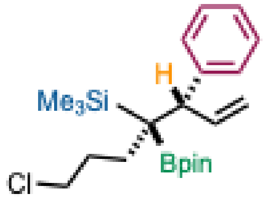

Prepared according to general procedure **GP3** from **2i** (92 mg, 200 µmol) and Me_3SiCl (127 µL, 1.00 mmol).

Yield: 45 mg (111 µmol, 55%) as an inseparable mixture of diastereomers (*dr* 96:04 as determined by ^1H NMR spectroscopy) as a colorless oil.

R*f* = 0.65 (PE/Et_2O 40:1).

Major diastereomer:
^{1}H NMR (400 MHz, $CDCl_3$): δ 7.32 (d, *J* = 7.7 Hz, 2H), 7.23 (t, *J* = 7.3 Hz, 2H), 7.15 (t, *J* = 6.7 Hz, 1H), 6.58 (dt, *J* = 17.9, 9.0 Hz, 1H), 4.94 (d, *J* = 3.9 Hz, 1H), 4.92-4.88 (m, 1H), 3.61 (d, *J* = 8.7 Hz, 1H), 3.42-3.24 (m, 2H), 1.96-1.83 (m, 1H), 1.83-1.66 (m, 2H), 1.48-1.37 (m, 1H), 1.27 (s, 6H), 1.24 (s, 6H), 0.00 (s, 9H) ppm.

^{13}C NMR (101 MHz, $CDCl_3$): δ 144.9, 143.7, 129.0, 128.3, 126.3, 114.5, 83.2, 53.3, 46.2, 31.0, 28.5, 25.5, 25.5, -0.3 ppm.
Note: Carbon atom attached to boron is not visible due to quadrupolar relaxation.

^{29}Si NMR (80 MHz, $CDCl_3$): δ 5.4 ppm.

Minor diastereomer:
^{1}H NMR (400 MHz, $CDCl_3$): δ 7.32 (d, *J* = 7.7 Hz, 2H), 7.23 (t, *J* = 7.3 Hz, 2H), 7.15 (t, *J* = 6.7 Hz, 1H), 6.40 (dt, *J* = 17.2, 9.0 Hz, 1H), 5.00 (d, *J* = 6.4 Hz, 1H), 4.92-4.88 (m, 1H), 3.79 (d, *J* = 9.5 Hz, 1H), 3.42-3.24 (m, 2H), 2.07-1.94 (m, 1H), 1.83-1.66 (m, 2H), 1.48-1.37 (m, 1H), 1.10 (s, 6H), 1.05 (s, 6H), 0.06 (s, 9H) ppm.

^{13}C NMR (101 MHz, $CDCl_3$): δ 144.9, 143.7, 129.5, 127.9, 126.3, 115.6, 83.2, 52.6, 46.7, 31.0, 28.5, 25.9, 25.0, 0.2 ppm.
Note: Carbon atom attached to boron is not visible due to quadrupolar relaxation.

^{29}Si NMR (80 MHz, $CDCl_3$): δ 4.9 ppm.

^{11}B NMR (128 MHz, $CDCl_3$): δ 33.8 ppm.

HRMS (APCI+) m/z: calcd. for $C_{22}H_{37}BClO_2Si^+$ $[M+H]^+$: 407.2339, found: 407.2369.

((1*S,2*S**)-1-Cyclohexyl-2-phenyl-1-(4,4,5,5-tetramethyl-1,3,2-dioxaborolan-2-yl)but-3-en-1-yl)trimethylsilane 5l**

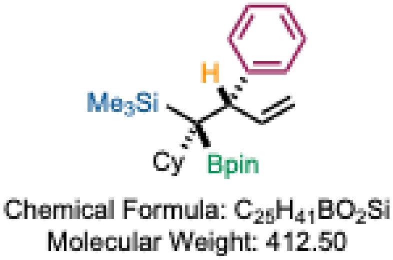

Prepared according to general procedure **GP3** from **2j** (93 mg, 200 µmol) and Me_3SiCl (150 µL, 1.18 mmol).

Yield: 40 mg (97 µmol, 49%, *dr* > 95:05 as determined by ^{1}H NMR spectroscopy) as a colorless oil.

R*f* = 0.75 (PE/Et_2O 40:1).

^{1}H NMR (400 MHz, $CDCl_3$) δ 7.59 (d, *J* = 7.4 Hz, 2H), 7.21 (t, *J* = 7.4 Hz, 2H), 7.13 (t, *J* = 7.1 Hz, 1H), 6.71 (dt, *J* = 17.3, 9.8 Hz, 1H), 4.91 (d, *J* = 17.6 Hz, 1H), 4.90-4.79 (m, 1H), 3.64 (d, *J* = 9.6 Hz, 1H), 1.94-1.76 (m, 2H), 1.75-1.54 (m, 2H), 1.52-1.40 (m, 2H), 1.35 (s, 6H), 1.31 (s, 6H), 1.27-1.14 (m, 2H), 1.10-0.94 (m, 2H), 0.92-0.74 (m, 1H), 0.16 (s, 9H) ppm.

^{13}C NMR (101 MHz, $CDCl_3$) δ 148.7, 144.5, 129.3, 128.1, 125.8, 113.9, 82.9, 52.8, 43.3, 33.9, 30.1, 28.5, 27.8, 26.5, 26.1, 25.6, 1.5 ppm.
Note: Carbon atom attached to boron is not visible due to quadrupolar relaxation.

^{29}Si NMR (80 MHz, $CDCl_3$): δ 6.2 ppm.

^{11}B NMR (128 MHz, $CDCl_3$): δ 34.5 ppm.

HRMS (APCI+) m/z: calcd. for $C_{25}H_{42}BO_2Si^+$ [*M*+H]$^+$: 413.3047, found: 413.3024.

((3*S,4*S**)-4-(4-(*tert*-Butyl)-2,3,5,6-tetrafluorophenyl)-3-(4,4,5,5-tetramethyl-1,3,2-dioxaborolan-2-yl)hex-5-en-3-yl)trimethylsilane 5m**

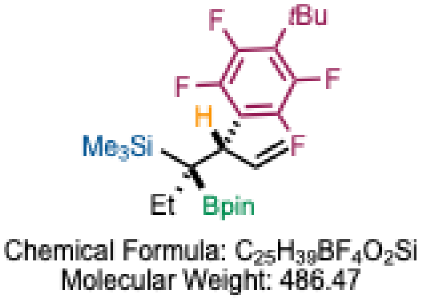

Prepared according to general procedure **GP3** from **2k** (64 mg, 127 µmol) and Me_3SiCl (81 µL, 637 µmol).

Yield: 48 mg (99 µmol, 77%) as an inseparable mixture of diastereomers (*dr* 81:19 as determined by ^{1}H NMR spectroscopy) as a colorless oil.

R*f* = 0.60 (PE/Et_2O 40:1).

Major diastereomer:

^{1}H NMR (400 MHz, $CDCl_3$): δ 6.69-6.51 (m, 1H), 5.18-4.92 (m, 2H), 4.28 (d, *J* = 9.1 Hz, 1H), 1.75 (dq, *J* = 14.7, 7.4 Hz, 1H), 1.46 (s, 9H), 1.49-1.40 (m, 1H), 1.27 (s, 12H), 0.88 (t, *J* = 7.4 Hz, 3H), 0.08 (s, 9H) ppm.

^{13}C NMR (101 MHz, $CDCl_3$): δ 147.1-146.6 (m), 144.6-144.3 (m), 140.2, 124.7 (d, $J_{C\text{-}F}$ = 13.2 Hz), 120.3 (d, $J_{C\text{-}F}$ = 17.2 Hz), 115.5, 83.0, 42.7, 36.7, 31.02, 31.00, 30.92, 25.8, 25.6, 24.4, 12.6, -0.1 ppm.
Note: Carbon atom attached to boron is not visible due to quadrupolar relaxation.

^{19}F NMR (377 MHz, $CDCl_3$): δ –139.2 (d, *J* = 20.4 Hz), –139.3 (d, *J* = 20.4 Hz) ppm.

^{29}Si NMR (80 MHz, $CDCl_3$): δ 7.1 ppm.

Minor diastereomer:
^{1}H NMR (400 MHz, $CDCl_3$): δ 6.50-6.33 (m, 1H), 5.15-4.99 (m, 2H), 4.28 (d, *J* = 9.1 Hz, 1H), 1.85 (q, *J* = 7.2 Hz, 1H), 1.49-1.42 (m, 1H), 1.44 (s, 9H), 1.14 (s, 12H), 1.04 (d, *J* = 7.4 Hz, 3H), 0.14 (s, 9H) ppm.

^{13}C NMR (101 MHz, $CDCl_3$): δ 147.1-146.6 (m), 144.6-144.3 (m), 137.6, 124.6 (d, $J_{C\text{-}F}$ = 13.2 Hz), 120.1 (d, $J_{C\text{-}F}$ = 17.2 Hz), 117.0, 82.9, 43.0, 36.7, 31.00, 30.92, 30.86, 25.7, 25.0, 22.5, 13.6, 0.5 ppm.
Note: Carbon atom attached to boron is not visible due to quadrupolar relaxation.

^{19}F NMR (377 MHz, $CDCl_3$): δ -139.3 (d, *J* = 20.4 Hz), -139.4 (d, *J* = 20.4 Hz) ppm.

^{29}Si NMR (80 MHz, $CDCl_3$): δ 6.4 ppm.

^{11}B NMR (128 MHz, $CDCl_3$): δ 34.9 ppm.

HRMS (APCI+) m/z: calcd. for $C_{25}H_{40}BF_4O_2Si^+$ $[M+H]^+$: 487.2821, found: 487.2832.

Trimethyl((3*S,4*R**)-3-(4,4,5,5-tetramethyl-1,3,2-dioxaborolan-2-yl)-4-(thiophen-2-yl)hex-5-en-3-yl)silane 5n and trimethyl((3*S**,4*R**)-3-(4,4,5,5-tetramethyl-1,3,2-dioxaborolan-2-yl)-4-(5-(trimethylsilyl)thiophen-2-yl)hex-5-en-3-yl)silane 5n**–Si

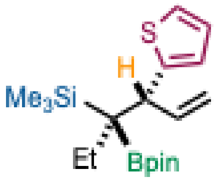

Chemical Formula: $C_{19}H_{33}BO_2SSi$
Molecular Weight: 364.43

Prepared according to general procedure **GP3** from **2l** (63 mg, 150 µmol) and Me_3SiCl (95 µL, 750 µmol).

Yield: 28 mg (77 µmol, 51%) as an inseparable mixture of diastereomers (*dr* 80:20 as determined by ^{1}H NMR spectroscopy), inseparable from oversilylated byproduct **5n**-Si as an inseparable mixture of diastereomers (7 mg, 16 µmol, 11%, *dr* 80:20 as determined by ^{1}H NMR spectroscopy) as a colorless oil.

R*f* = 0.85 (PE/Et_2O 40:1).

Major diastereomer:
^{1}H NMR (400 MHz, $CDCl_3$): δ 7.12 (d, *J* = 3.0 Hz, 1H), 6.91 (d, *J* = 3.5 Hz, 2H), 6.57-6.43 (m, 1H), 5.03-4.88 (m, 2H), 4.07 (d, *J* = 8.4 Hz, 1H), 1.85 (dt, *J* = 14.8, 7.4 Hz, 1H), 1.66 (dt, *J* = 15.0, 7.6 Hz, 1H), 1.29 (s, 6H), 1.27 (s, 6H), 1.01 (t, *J* = 7.5 Hz, 3H), 0.01 (s, 9H) ppm.

^{13}**C NMR** (101 MHz, $CDCl_3$): δ 147.7, 143.7, 126.3, 125.1, 123.3, 114.3, 83.1, 47.7, 25.8, 25.3, 23.2, 12.7, -0.4 ppm.
Note: Carbon atom attached to boron is not visible due to quadrupolar relaxation.

^{29}**Si NMR** (80 MHz, $CDCl_3$): δ 4.8 ppm.

Minor diastereomer:
^{1}**H NMR** (400 MHz, $CDCl_3$): δ 7.13 (d, *J* = 3.5 Hz, 1H), 6.91 (d, *J* = 2.9 Hz, 2H), 6.35-6.25 (m, 1H), 5.07-4.97 (m, 2H), 4.12 (d, *J* = 9.5 Hz, 1H), 1.85 (dt, *J* = 14.8, 7.4 Hz, 1H), 1.66 (dt, *J* = 15.0, 7.6 Hz, 1H), 1.29 (s, 6H), 1.26 (s, 6H), 1.04-0.96 (m, 3H), 0.01 (s, 9H) ppm.

^{13}**C NMR** (101 MHz, $CDCl_3$): δ 147.7, 143.5, 126.4, 126.0, 125.3, 114.3, 83.1, 47.8, 25.8, 25.3, 23.2, 12.6, -0.2 ppm.
Note: Carbon atom attached to boron is not visible due to quadrupolar relaxation.

^{29}**Si NMR** (80 MHz, $CDCl_3$): δ 4.7 ppm.

^{11}**B NMR** (128 MHz, $CDCl_3$): δ 33.8 ppm.

HRMS (APCI+) m/z: calcd. for $C_{19}H_{34}BO_2SSi^+$ [*M*+H]$^+$: 365.2136, found: 365.2168.

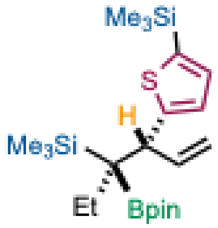

Chemical Formula: $C_{22}H_{41}BO_2SSi_2$
Molecular Weight: 436.61

R*f* = 0.90 (PE/Et_2O 40:1).

Major diastereomer:
^{1}**H NMR** (400 MHz, $CDCl_3$): δ 7.09 (d, *J* = 5.1 Hz, 1H), 6.88 (d, *J* = 5.0 Hz, 1H), 6.53-6.43 (m, 1H), 5.01 (d, *J* = 8.9 Hz, 2H), 4.12 (d, *J* = 9.5 Hz, 1H), 2.01-1.88 (m, 1H), 1.75-1.66 (m, 1H), 1.20 (s, 6H), 1.15 (s, 6H), 1.00 (t, *J* = 7.5 Hz, 3H), 0.27 (s, 9H), 0.08 (s, 9H).

^{13}**C NMR** (101 MHz, $CDCl_3$): δ 153.3, 147.7, 141.0, 133.4, 125.1, 115.21, 83.0, 48.1, 25.80, 25.0, 22.6, 12.7, 0.22, 0.16 ppm.
Note: Carbon atom attached to boron is not visible due to quadrupolar relaxation.

^{29}**Si NMR** (80 MHz, $CDCl_3$): δ 7.3 ppm.

Minor diastereomer:
^{1}**H NMR** (400 MHz, $CDCl_3$): δ 7.01 (d, *J* = 3.3 Hz, 1H), 6.86 (d, *J* = 3.6 Hz, 1H), 6.36-6.21 (m, 1H), 5.08-4.97 (m, 2H), 4.16 (d, *J* = 9.2 Hz, 1H), 2.01-1.86 (m, 1H), 1.78-1.66 (m, 1H), 1.18 (s, 6H), 1.10 (s, 6H), 0.98 (t, *J* = 7.2 Hz, 3H), 0.26 (s, 9H), 0.09 (s, 9H) ppm.

^{13}**C NMR** (101 MHz, $CDCl_3$): δ 153.3, 147.7, 141.0, 133.3, 126.5, 115.24, 82.9, 48.2, 25.76, 25.0, 22.6, 12.7, 0.24, 0.13 ppm.
Note: Carbon atom attached to boron is not visible due to quadrupolar relaxation.

^{29}**Si NMR** (80 MHz, $CDCl_3$): δ 7.4 ppm.

^{11}**B NMR** (128 MHz, $CDCl_3$): δ 33.8 ppm.

HRMS (APCI+) m/z: calcd. for $C_{22}H_{42}BO_2SSi_2^+$ [*M*+H]$^+$: 437.2532, found: 437.2561.

3.3 Determination of the stereochemical outcome

Dioxo[*N*,*N*,*N*,*N*-tetramethylethane-1,2-diaminetetramethylehylendiamine][(2*R,3*R**,4*S**)-3-phenyl-4-(4,4,5,5-tetramethyl-1,3,2-dioxaborolan-2-yl)-4-(trimethylsilyl)hexane-1,2-diol]osmium complex 6**

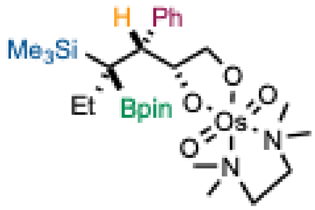

Chemical Formula: $C_{27}H_{51}BN_2O_6OsSi$
Molecular Weight: 728.84

According to a literature procedure, a flame-dried Schlenk flask was charged with a solution of **5a** (36 mg, 100 µmol) and TMEDA (17 µL, 111 µmol) in dry DCM (1 mL) and a solution of OsO_4 (25 mg, 100 µmol, 1M in DCM) was added dropwise at –78 °C. After 15 min, the reaction was complete as indicated by TLC analysis. The volatiles were removed under reduced pressure and the crude residue was purified by flash column chromatography (silica gel, gradient DCM/MeOH 99:1 to 90:10) to obtain desired osmate **6** (73 mg, 100 µmol, quantitative yield, *dr* > 95:05 as determined by ^{1}H NMR spectroscopy) as a reddish metallic solid.

R*f* = 0.40 (DCM/MeOH 9:1).

^{1}**H NMR** (400 MHz, $CDCl_3$): δ 7.39 (d, *J* = 6.7 Hz, 2H), 7.17-7.00 (m, 3H), 4.77 (ddd, *J* = 10.9, 4.7, 1.8 Hz, 1H), 4.26 (dd, *J* = 9.6, 4.6 Hz, 1H), 3.86 (t, *J* = 10.1 Hz, 1H), 3.80 (d, *J* = 1.5 Hz, 1H), 3.12-2.91 (m, 4H), 2.84 (s, 3H), 2.76 (s, 3H), 2.74 (s, 6H), 2.14 (tq, *J* = 15.0, 7.5 Hz, 1H), 1.78 (dq, *J* = 14.7, 7.4 Hz, 1H), 1.33 (t, *J* = 7.4 Hz, 3H), 1.29 (s, 6H), 1.27 (s, 6H), -0.26 (s, 9H) ppm.

^{13}**C NMR** (101 MHz, $CDCl_3$): δ 141.4, 132.8, 126.7, 125.9, 95.0, 84.8, 83.0, 64.2, 52.4, 52.0, 51.2, 50.8, 46.5, 26.0, 25.1, 21.9, 13.3, -1.1 ppm.

^{11}**B NMR** (128 MHz, $CDCl_3$): δ 35.9 ppm.

HRMS (APCI+) m/z: calcd. for $C_{27}H_{52}BN_2O_6OsSi^+$ [*M*+H]$^+$: 731.3297, found: 731.3320.

Recrystallization by vapour diffusion (DCM/Et_2O 1:2 with pentane as antisolvent) yielded X-ray quality crystals. The identity and purity of the analyzed crystal was subsequently re-confirmed by ^{1}H NMR analysis.

Crystal structure of 6

The single-crystal of light brown plate (**6**; Marek2R) from Et_2O/DCM/pentane, in Paratone–N oil and mounted on a Rigaku Oxford Diffraction - XtaLAB Synergy-S Data collection was performed using monochromated Mo Kα radiation, λ = 0.71073 Å. Accurate cell parameters were obtained with the amount of indicated reflections. Using Olex2,[5] the structure was solved with the olex2.solve[6] structure solution program using Charge Flipping and refined with the ShelXL[7] refinement package using Least Squares minimization. All non-hydrogen atoms were refined with anisotropic displacement parameters. The hydrogen atoms were refined isotropically in calculated positions using a riding model with their U_{iso} values constrained to 1.5 times the U_{eq} of their pivot atoms for terminal sp^3 carbon atoms and 1.2 times for all other carbon atoms. Software used for molecular graphics: Mercury 2022.3.0.[8]

Crystal structure determination of 6

Crystal data		6 (Marek2R)	
CCDC number	2471551	Calculated density (mg/m^3)	1.532
Empirical formula	$C_{27}H_{51}BN_2O_6OsSi$	Absorption coefficient (mm^{-1})	4.113
Formula weight	728.79	F(000)	2960
Temperature (K)	100.15	Crystal size (mm)	0.27 × 0.15 × 0.09
Wavelength (Å)	0.71073	2Theta range	4.676 - 60.034
Crystal system,	monoclinic	Reflection collected/unique	24994 / 7202
space group	I2/a	Rint	0.0745
a (Å)	42.2448(2)	Completeness (%)	99.2
b (Å)	11.0969(4)	Absorption correction	semi-empirical
c (Å)	13.5035(4)	Data/restraints/ parameters	7202/0/355
alpha	90	Goodness-of-fit on F^2	1.040
beta	93.121(3)	R1, wR2 [I>2sigma(I)]	0.0549, 0.1269
gamma	90	R1, wR2 (all data)	0.0736, 0.1336
Volume (Å^3)	6320.8(4)	Largest diff. peak and hole	4.98/-2.05
Z	8	Diffractometer	XtaLAB Synergy-S

Datablock Marek2R - ellipsoid plot

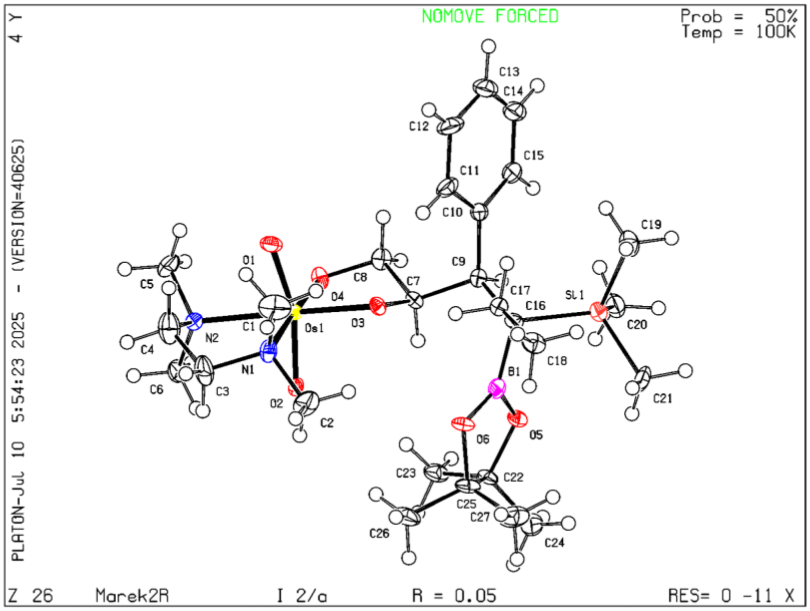

3.4 Scope of electrophiles

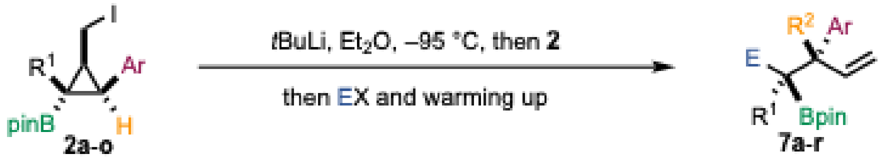

General procedure for Li/halogen exchange-mediated ring opening/electrophilic trapping – GP4: A flame-dried Schlenk flask was charged with dry Et_2O (0.2M with respect to **2**, typically 1 mL) under argon and a solution of *t*BuLi (2.5 equiv., typically 1.4M in pentane, freshly titrated before use against *N*-benzyl benzamide)[4] was added at –78 °C. The mixture was cooled to –95 °C with good stirring and a solution of iodide **2** (typically 200 µmol) in dry Et_2O (0.2M) was added via a cannula. A bright yellow precipitate formed. Electrophile (3-5 equiv.) was added and the mixture was stirred with warming up to the required temperature while monitored by TLC against the proton-quench product **5**-H (R*f* ≈ 0.45). Completion was accompanied by discoloration and was typically reached after 30-40 min of stirring at –78 to –30 °C. The reaction mixture was subsequently quenched with MeOH (10 equiv.), diluted with Et_2O and filtered over a short plug of silica gel. The crude residue was purified by column chromatography (gradient PE/Et_2O 999:1 to 99:1; majority of compounds elute at 997:3) to obtain title compounds **7**.

4,4,5,5-Tetramethyl-2-(4-phenylhex-5-en-3-yl)-1,3,2-dioxaborolane 5a-H

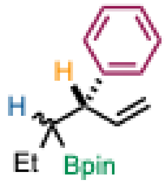

Chemical Formula: $C_{18}H_{27}BO_2$
Molecular Weight: 286.22

Prepared according to general procedure **GP4** from **2a** (59 mg, 143 µmol) and MeOH (50 µL, 1.24 mmol).

Yield: 41 mg (143 µmol, quantitative) as an inseparable mixture of diastereomers (*dr* 53:47 as determined by ^{1}H NMR spectroscopy) as a colorless oil.

R*f* = 0.45 (PE/Et_2O 40:1).

Major diastereomer:
^{1}H NMR (400 MHz, $CDCl_3$): δ 7.32-7.21 (m, 2H), 7.20-7.05 (m, 3H), 5.84 (ddd, *J* = 16.9, 10.1, 9.1 Hz, 1H), 5.01 (ddd, *J* = 16.9, 1.9, 0.9 Hz, 1H), 4.99-4.91 (m, 1H), 3.28-3.17 (m, 1H), 1.73-1.61 (m, 1H), 1.45-1.35 (m, 1H), 1.32-1.12 (m, 13H), 0.83 (t, *J* = 7.4 Hz, 3H) ppm.

^{13}C NMR (101 MHz, $CDCl_3$): δ 145.0, 142.9, 128.4, 128.1, 126.2, 114.3, 83.0, 52.6, 24.8, 24.6, 23.0, 13.8 ppm.
Note: Carbon atom attached to boron is not visible due to quadrupolar relaxation.

Minor diastereomer:
^{1}H NMR (400 MHz, $CDCl_3$): δ 7.32-7.21 (m, 2H), 7.20-7.05 (m, 3H), 6.01 (ddd, *J* = 17.1, 10.2, 8.4 Hz, 1H), 5.08 (ddd, *J* = 17.1, 1.8, 1.0 Hz, 1H), 4.99-4.89 (m, 1H), 3.33 (dd, *J* = 11.0, 8.4 Hz, 1H), 1.45-1.35 (m, 1H), 1.26 (s, 2H), 0.97 (s, 6H), 0.93 (s, 6H), 0.83 (t, *J* = 7.4 Hz, 3H) ppm.

^{13}C NMR (101 MHz, $CDCl_3$): δ 144.4, 142.5, 128.5, 127.9, 126.1, 114.1, 83.3, 52.1, 25.1, 25.1, 22.8, 13.7 ppm.
Note: Carbon atom attached to boron is not visible due to quadrupolar relaxation.

^{11}B NMR (128 MHz, $CDCl_3$): δ 33.6 ppm.

HRMS (APCI+) m/z: calcd. for $C_{18}H_{28}BO_2^+$ [*M*+H]$^+$: 287.2177, found: 287.2185.

4,4,5,5-Tetramethyl-2-((3*S,4*R**)-3-methyl-4-phenylhex-5-en-3-yl)-1,3,2-dioxaborolane 7a**

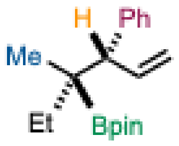

Chemical Formula: $C_{19}H_{29}BO_2$
Molecular Weight: 300.25

Prepared according to general procedure **GP4** from **2a** (62 mg, 150 µmol) and methyl iodide (47 mL, 750 µmol).

Yield: 28 mg (93 µmol, 62%) as an inseparable mixture of diastereomers (*dr* 91:09 as determined by ^{1}H NMR spectroscopy) as a colorless oil.

R*f* = 0.55 (PE/Et_2O 40:1).

Major diastereomer:
^{1}H NMR (400 MHz, $CDCl_3$): δ 7.39-7.20 (m, 4H), 7.21-7.10 (m, 1H), 6.23 (dt, *J* = 16.8, 9.9 Hz, 1H), 5.11-4.94 (m, 2H), 3.32 (d, *J* = 9.7 Hz, 1H), 1.53 (dt, *J* = 13.1, 6.5 Hz, 1H), 1.29-1.22 (m, 1H), 1.17 (s, 6H), 1.08 (s, 6H), 0.98 (s, 3H), 0.86 (t, *J* = 7.5 Hz, 3H) ppm.

^{13}C NMR (101 MHz, $CDCl_3$) δ 143.5, 139.0, 129.4, 127.9, 126.1, 116.0, 83.2, 57.7, 30.4, 25.4, 24.8, 17.9, 10.5 ppm.
Note: Carbon atom attached to boron is not visible due to quadrupolar relaxation.

Minor diastereomer:
^{1}H NMR (400 MHz, $CDCl_3$): δ 7.39-7.20 (m, 4H), 7.21-7.10 (m, 1H), 6.34 (dt, *J* = 17.0, 9.8 Hz, 1H), 5.11-4.94 (m, 2H), 3.31 (d, *J* = 9.4 Hz, 1H), 1.53 (dt, *J* = 13.1, 6.5 Hz, 1H), 1.29-1.22 (m, 1H), 1.17 (s, 6H), 1.08 (s, 6H), 0.98 (s, 3H), 0.81(t, *J* = 7.4 Hz, 3H) ppm.

^{13}C NMR (101 MHz, $CDCl_3$): δ 143.5, 140.2, 129.5, 127.8, 126.1, 115.8, 83.4, 58.0, 31.2, 25.3, 24.7, 17.9, 10.4 ppm.
Note: Carbon atom attached to boron is not visible due to quadrupolar relaxation.

^{11}B NMR (128 MHz, $CDCl_3$): δ 34.0 ppm.

The NMR data are in accordance to previously published values.[3a]

HRMS (APCI+) m/z: calcd. for $C_{19}H_{30}BO_2^+$ [*M*+H]$^+$: 301.2333, found: 301.2342.

2-((3*R,4*R**)-4-Ethyl-3-phenyloct-1-en-4-yl)-4,4,5,5-tetramethyl-1,3,2-dioxaborolane 7b**

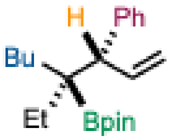

Chemical Formula: $C_{22}H_{35}BO_2$
Molecular Weight: 342.33

Prepared according to general procedure **GP4** from **2a** (62 mg, 150 µmol) and ***n*-butyl iodide** (85 µL, 750 µmol).

Yield: 29 mg (85 µmol, 57%, dr > 95:05 as determined by ^{1}H NMR spectroscopy) as a colorless oil.

Prepared according to general procedure **GP4** from **2a** (45 mg, 110 µmol) and ***n*-butyl bromide** (60 µL, 550 µmol).

Yield: 46 µmol (42%, *dr* 88:12 as determined by ^{1}H NMR spectroscopy).

Prepared according to general procedure **GP4** from **2a** (45 mg, 110 µmol) and ***n*-butyl chloride** (58 µL, 660 µmol).

Yield: 39 µmol (35%, *dr* 88:12 as determined by ^{1}H NMR spectroscopy).

R*f* = 0.65 (PE/Et_2O 40:1).

^{1}H NMR (400 MHz, $CDCl_3$): δ 7.28-7.24 (m, 4H), 7.17 (dd, *J* = 8.7, 4.3 Hz, 1H), 6.52-6.37 (m, 1H), 5.01 (d, *J* = 11.3 Hz, 1H), 5.02-4.95 (m, 1H), 3.25 (d, *J* = 9.8 Hz, 1H), 1.49-1.36 (m, 2H), 1.36-1.22 (m, 6H), 1.29 (s, 6H), 1.26 (s, 6H), 0.92 (t, *J* = 7.1 Hz, 3H), 0.80 (t, *J* = 7.4 Hz, 3H) ppm.

^{13}C NMR (101 MHz, $CDCl_3$): δ 144.0, 141.3, 128.9, 128.0, 126.0, 115.0, 83.2, 56.8, 32.8, 26.8, 25.4, 25.3, 23.9, 23.8, 14.4, 8.3 ppm.
Note: Carbon atom attached to boron is not visible due to quadrupolar relaxation.

^{11}B NMR (128 MHz, $CDCl_3$): δ 34.0 ppm.

The NMR data are in accordance to the previously published values.[3a]

HRMS (APCI+) m/z: calcd. for $C_{22}H_{36}BO_2^+$ $[M+H]^+$: 343.2803, found: 343.2810.

2-((3*R,4*R**)-4-Ethyl-6-methyl-3-phenylhept-1-en-4-yl)-4,4,5,5-tetramethyl-1,3,2-dioxaborolane 7c**

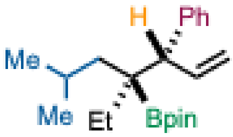

Chemical Formula: $C_{22}H_{35}BO_2$
Molecular Weight: 342.33

Prepared according to general procedure **GP4** from **2a** (82 mg, 200 µmol) and isobutyl iodide (162 µL, 600 µmol).

Yield: 46 mg (134 µmol, 67%, dr > 95:05 as determined by ^{1}H NMR spectroscopy) as a colorless oil.

R*f* = 0.65 (PE/Et_2O 40:1).

^{1}H NMR (400 MHz, $CDCl_3$): δ 7.22-7.14 (m, 4H), 7.11-7.03 (m, 1H), 6.37 (dt, *J* = 16.8, 9.9 Hz, 1H), 4.97-4.90 (m, 1H), 4.92-4.84 (m, 1H), 3.18 (d, *J* = 9.6 Hz, 1H), 1.58 (dq, *J* = 13.0, 6.8 Hz, 1H), 1.50-1.37 (m, 2H), 1.37-1.27 (m, 1H), 1.22 (s, 6H), 1.26-1.12 (m, 1H), 1.18 (s, 6H), 0.91-0.87 (m, 3H), 0.87-0.82 (m, 3H), 0.77 (t, *J* = 7.3 Hz, 3H) ppm.

^{13}C NMR (101 MHz, $CDCl_3$): δ 144.0, 141.4, 129.0, 128.8, 126.0, 115.0, 83.3, 57.2, 43.1, 25.8, 25.7, 25.5, 25.3, 24.6, 23.5, 8.0 ppm.
Note: Carbon atom attached to boron is not visible due to quadrupolar relaxation.

^{11}B NMR (128 MHz, $CDCl_3$): δ 33.8 ppm.

HRMS (APCI+) m/z: calcd. for $C_{22}H_{36}BO_2^+$ [*M*+H]$^+$: 343.2808, found: 343.2801.

4,4,5,5-Tetramethyl-2-((3*R*,4*R*)-4-methyl-3-phenyldodec-1-en-4-yl)-1,3,2-dioxaborolane 7d

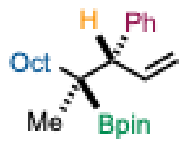

Chemical Formula: $C_{25}H_{41}BO_2$
Molecular Weight: 384.41

Prepared according to general procedure **GP4** from **2b** (70 mg, 175 µmol) and *n*-octyl iodide (95 µL, 525 µmol).

Yield: 35 mg (91 µmol, 52%) as an inseparable mixture of diastereomers (*dr* 90:10 as determined by ^{1}H NMR spectroscopy) as a colorless oil.

R*f* = 0.55 (PE/Et_2O 40:1).

Major diastereomer:
^{1}H NMR (400 MHz, $CDCl_3$): δ 7.30-7.21 (m, 3H), 7.18 (d, *J* = 7.4 Hz, 2H), 6.34 (dt, *J* = 17.1, 9.8 Hz, 1H), 5.07 (dd, *J* = 17.3, 2.0 Hz, 1H), 5.02 (dd, *J* = 9.7, 2.1 Hz, 1H), 3.30 (d, *J* = 9.4 Hz, 1H), 1.55-1.43 (m, 1H), 1.26 (s, 6H), 1.25 (s, 6H), 1.22 (s, 3H), 1.33-1.16 (m, 10H), 0.89 (t, *J* = 7.4 Hz, 3H), 0.86 (t, *J* = 7.4 Hz, 3H) ppm.

^{13}C NMR (101 MHz, $CDCl_3$): δ 142.4, 140.3, 129.6, 127.8, 126.0, 115.8, 83.3, 58.2, 39.0, 32.0, 30.7, 29.7, 29.4, 26.2, 25.4, 25.2, 22.8, 17.7, 14.3 ppm.
Note: Carbon atom attached to boron is not visible due to quadrupolar relaxation.

Minor diastereomer:
^{1}H NMR (400 MHz, $CDCl_3$): δ 7.30-7.21 (m, 3H), 7.16 (d, *J* = 6.8 Hz, 2H), 6.22 (dt, *J* = 17.0, 9.9 Hz, 1H), 5.07 (dd, *J* = 17.3, 2.0 Hz, 1H), 5.07-4.97 (m, 1H), 3.34 (d, *J* = 9.5 Hz, 1H), 1.47-1.37 (m, 1H), 1.26 (s, 6H), 1.25 (s, 6H), 1.22 (s, 3H), 1.33-1.16 (m, 10H), 0.89 (t, *J* = 7.4 Hz, 3H), 0.86 (t, *J* = 7.4 Hz, 3H) ppm.

^{13}C NMR (101 MHz, $CDCl_3$): δ 142.4, 140.3, 129.5, 127.9, 126.0, 115.8, 83.3, 57.7, 39.0, 32.0, 30.8, 29.7, 29.4, 26.2, 25.0, 24.8, 22.8, 17.7, 14.3 ppm.
Note: Carbon atom attached to boron is not visible due to quadrupolar relaxation.

^{11}B NMR (128 MHz, $CDCl_3$): δ 35.5 ppm.

HRMS (APCI+) m/z: calcd. for $C_{25}H_{42}BO_2^+$ [*M*+H]$^+$: 385.3272, found: 385.3279.

4,4,5,5-Tetramethyl-2-((3*R,4*R**)-4-methyl-7-phenyl-3-(*p*-tolyl)hept-1-en-4-yl)-1,3,2-dioxaborolane 7e**

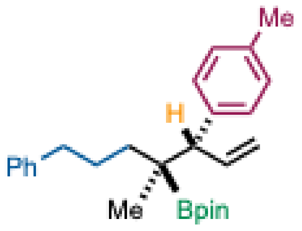

Chemical Formula: $C_{27}H_{37}BO_2$
Molecular Weight: 404.40

Prepared according to general procedure **GP4** from **2c** (78 mg, 190 µmol) and 1-phenylprop-3-yl iodide (107 µL, 665 µmol).

Yield: 43 mg (106 µmol, 56%, *dr* > 95:05 as determined by ^{1}H NMR spectroscopy) as a colorless oil.

R*f* = 0.45 (PE/Et_2O 40:1).

^{1}H NMR (400 MHz, $CDCl_3$) δ 7.44 (t, *J* = 7.3 Hz, 2H), 7.38-7.33 (m, 2H), 7.31 (d, *J* = 7.3 Hz, 4H), 7.28-7.17 (m, 1H), 6.51 (dt, *J* = 17.0, 9.8 Hz, 1H), 5.25 (d, *J* = 16.9 Hz, 1H), 5.22-5.17 (m, 1H), 3.47 (d, *J* = 9.4 Hz, 1H), 2.70 (d, *J* = 6.8 Hz, 2H), 2.50 (s, 3H), 2.58-2.41 (m, 1H), 1.85-1.71 (m, 1H), 1.71-1.58 (m, 1H), 1.43 (s, 12H), 1.21-1.15 (m, 1H), 1.08 (s, 3H) ppm.

^{13}C NMR (101 MHz, $CDCl_3$): δ 143.1, 140.3, 139.3, 135.5, 129.4, 128.6, 128.4, 128.3, 125.7, 115.7, 83.4, 57.6, 38.8, 37.2, 28.4, 25.4, 25.2, 21.1, 17.7 ppm.
Note: Carbon atom attached to boron is not visible due to quadrupolar relaxation.

^{11}B NMR (128 MHz, $CDCl_3$): δ 35.4 ppm.

HRMS (APCI+) m/z: calcd. for $C_{27}H_{38}BO_2^+$ [*M*+H]$^+$: 405.2959, found: 405.3006.

4,4,5,5-Tetramethyl-2-((3*R,4*R**)-4-methyl-3-(*p*-tolyl)oct-1-en-4-yl)-1,3,2-dioxaborolane 7f**

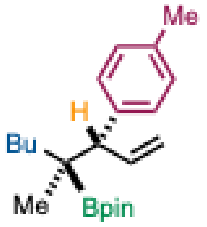

Chemical Formula: $C_{22}H_{35}BO_2$
Molecular Weight: 342.33

Prepared according to general procedure **GP4** from **2c** (91 mg, 220 µmol) and *n*-butyl iodide (75 µL, 660 µmol).

Yield: 64 mg (172 µmol, 78%) as a partially separable mixture of diastereomers (*dr* 94:06 as determined by ^{1}H NMR spectroscopy) as a colorless oil.

Major diastereomer:
^{1}H NMR (400 MHz, $CDCl_3$): δ 7.11-7.02 (m, 4H), 6.33 (dt, *J* = 17.0, 9.8 Hz, 1H), 5.06 (dd, *J* = 17.1, 1.9 Hz, 1H), 5.01 (d, *J* = 2.2 Hz, 1H), 3.28 (d, *J* = 9.5 Hz, 1H), 2.31 (s, 3H), 1.56-1.42 (m, 1H), 1.26 (s, 6H), 1.25 (s, 6H), 1.35-1.10 (m, 5H), 0.89 (s, 3H), 0.85 (t, *J* = 7.1 Hz, 3H) ppm.

^{13}C NMR (101 MHz, $CDCl_3$): δ 140.5, 139.4, 135.4, 129.4, 128.5, 115.6, 83.3, 57.8, 38.6, 28.4, 25.4, 25.2, 23.8, 21.1, 17.7, 14.3 ppm.
Note: Carbon atom attached to boron is not visible due to quadrupolar relaxation.

Minor diastereomer:
^{1}H NMR (400 MHz, $CDCl_3$): δ 7.11-7.02 (m, 4H), 6.22 (dt, *J* = 16.7, 9.9 Hz, 1H), 5.03-4.90 (m, 2H), 3.30 (d, *J* = 11.8 Hz, 1H), 2.29 (s, 3H), 1.56-1.42 (m, 1H), 1.26 (s, 6H), 1.25 (s, 6H), 1.35-1.10 (m, 5H), 0.89 (s, 3H), 0.85 (t, *J* = 7.1 Hz, 3H) ppm.

^{13}C NMR (101 MHz, $CDCl_3$): δ 140.5, 139.4, 135.4, 129.3, 128.6, 115.8, 83.2, 57.3, 38.6, 28.4, 25.9, 24.8, 23.9, 21.3, 17.7, 14.3 ppm.
Note: Carbon atom attached to boron is not visible due to quadrupolar relaxation.

^{11}B NMR (128 MHz, $CDCl_3$): δ 34.1 ppm.

HRMS (APCI+) m/z: calcd. for $C_{28}H_{46}BO_2^+$ [*M*–H]$^+$: 341.2646, found: 341.2646.

4,4,5,5-Tetramethyl-2-((3*R,4*R**)-4-methyl-3-(*p*-tolyl)oct-tetradec-1-en-4-yl)-1,3,2-dioxaborolane 7g**

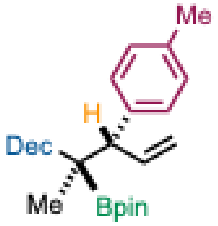

Chemical Formula: $C_{28}H_{47}BO_2$
Molecular Weight: 426.49

Prepared according to general procedure **GP4** from **2c** (78 mg, 190 µmol) and *n*-decyl iodide (128 µL, 600 µmol).

Yield: 32 mg (76 µmol, 40%) as a partially separable mixture of diastereomers (*dr* 89:11 as determined by ^{1}H NMR spectroscopy) as a colorless oil.

R*f* = 0.65 (PE/Et_2O 40:1).

Major diastereomer:
^{1}H NMR (400 MHz, $CDCl_3$): δ 7.19-6.91 (m, 4H), 6.32 (dt, *J* = 17.0, 9.7 Hz, 1H), 5.10-4.96 (m, 2H), 3.27 (d, *J* = 9.4 Hz, 1H), 2.30 (s, 3H), 1.53-1.38 (m, 1H), 1.26 (s, 6H), 1.25 (s, 6H), 1.35-0.98 (m, 17H), 0.89-0.86 (m, 6H) ppm.

^{13}C NMR (101 MHz, $CDCl_3$): δ 140.5, 139.4, 135.4, 129.4, 128.5, 115.6, 83.3, 57.7, 39.0, 32.1, 30.7, 29.8, 29.7, 29.7, 29.5, 26.2, 25.4, 25.2, 22.8, 21.1, 17.7, 14.3 ppm.
Note: Carbon atom attached to boron is not visible due to quadrupolar relaxation.

Minor diastereomer:
^{1}H NMR (400 MHz, $CDCl_3$): δ 7.19-6.91 (m, 4H), 6.24-6.11 (m, 1H), 5.10-4.96 (m, 2H), 3.30 (d, *J* = 9.1 Hz, 1H), 2.29 (s, 3H), 1.53-1.38 (m, 1H), 1.26 (s, 6H), 1.25 (s, 6H), 1.35-0.98 (m, 17H), 0.89-0.86 (m, 6H) ppm.

^{13}C NMR (101 MHz, $CDCl_3$): δ 140.5, 139.4, 135.4, 129.3, 128.6, 115.6, 83.3, 57.3, 38.2, 32.1, 30.7, 29.8, 29.7, 29.7, 29.5, 26.2, 25.0, 24.8, 22.8, 21.1, 17.7, 14.3 ppm.
Note: Carbon atom attached to boron is not visible due to quadrupolar relaxation.
^{11}B NMR (128 MHz, $CDCl_3$): δ 35.8 ppm.

HRMS (APCI+) m/z: calcd. for $C_{28}H_{46}BO_3^+$ [*M*–H+O]$^+$: 441.3540, found: 441.3539.

4,4,5,5-Tetramethyl-2-((3*R,4*R**)-4-methyl-3-(4-(trifluoromethyl)phenyl)tetradec-1-en-4-yl)-1,3,2-dioxaborolane 7h**

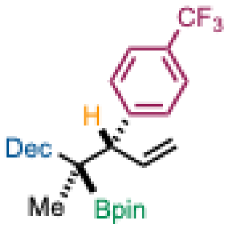

Chemical Formula: $C_{28}H_{44}BF_3O_2$
Molecular Weight: 480.46

Prepared according to general procedure **GP4** from **2d** (93 mg, 200 μmol) and *n*-decyl iodide (142 μL, 665 μmol).

Yield: 55 mg (114 μmol, 57%) as a partially separable mixture of diastereomers (*dr* 78:22 as determined by ^{19}F and ^{1}H NMR spectroscopy) as a colorless oil.

R*f* = 0.65 (PE/Et_2O 40:1).

Major diastereomer:
^{1}H NMR (400 MHz, $CDCl_3$): δ 7.50 (d, *J* = 8.1 Hz, 2H), 7.29 (d, *J* = 8.1 Hz, 2H), 6.31 (dt, *J* = 17.5, 9.5 Hz, 1H), 5.13-5.02 (m, 2H), 3.37 (d, *J* = 9.3 Hz, 1H), 1.54-1.39 (m, 1H), 1.26 (s, 6H), 1.24 (s, 6H), 1.33-1.05 (m, 17H), 0.93-0.83 (m, 6H) ppm.

^{13}C NMR (101 MHz, $CDCl_3$): δ 146.8, 139.5, 129.8, 128.3 (q, $J_{C\text{-}F}$ = 32.3 Hz), 124.7 (q, $J_{C\text{-}F}$ = 3.7 Hz), 124.5 (q, $J_{C\text{-}F}$ = 271.8 Hz), 116.6, 83.5, 58.0, 38.9, 32.1, 30.7, 29.73 (3C), 29.5, 26.1, 25.4, 25.2, 22.8, 17.8, 14.3 ppm.
Note: Carbon atom attached to boron is not visible due to quadrupolar relaxation.

^{19}F NMR (377 MHz, $CDCl_3$) δ –62.26 ppm.

Minor diastereomer:

^{1}**H NMR** (400 MHz, $CDCl_3$): δ 7.50 (d, *J* = 8.1 Hz, 2H), 7.40 (d, *J* = 8.0 Hz, 2H), 6.20 (dt, *J* = 16.9, 9.8 Hz,1H), 5.13-4.96 (m, 2H), 3.40 (d, *J* = 11.2 Hz, 1H), 1.54-1.39 (m, 1H), 1.33-1.05 (m, 17H), 1.17 (s, 6H), 1.07 (s, 6H), 0.99 (s, 3H), 0.87 (t, *J* = 7.4 Hz, 3H) ppm.

^{13}**C NMR** (101 MHz, $CDCl_3$): δ 147.8, 138.1, 129.8, 128.3 (q, J_{C-F} = 32.3 Hz), 124.8 (q, J_{C-F} = 3.7 Hz), 124.5 (q, J_{C-F} = 271.8 Hz), 116.9, 83.4, 57.5, 38.2, 32.1, 30.7, 29.8, 29.74 (2C), 29.5, 26.1, 25.0, 24.8, 22.8, 18.4, 14.3.
Note: Carbon atom attached to boron is not visible due to quadrupolar relaxation.

^{19}**F NMR** (377 MHz, $CDCl_3$) δ –62.31 ppm.

^{11}**B NMR** (128 MHz, $CDCl_3$): δ 35.1 ppm.

HRMS (APCI+) m/z: calcd. for $C_{28}H_{43}BF_3O_3^+$ [*M*–H_2+O+H]$^+$: 495.5352, found: 495.3254.

2-((3*R,4*R**)-3-(4-Chlorophenyl)-4-(3-phenylpropyl)oct-1-en-4-yl)-4,4,5,5-tetramethyl-1,3,2-dioxaborolane 7i**

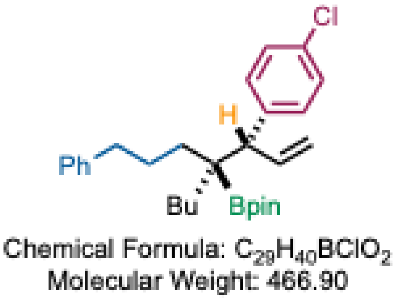

Prepared according to general procedure **GP4** from **2h** (95 mg, 200 µmol) and 1-phenylprop-3-yl iodide (97 µL, 600 µmol).

Yield: 30 mg (64 µmol, 32%, *dr* > 95:05 as determined by ^{1}H NMR spectroscopy) as a colorless oil.

R*f* = 0.40 (PE/Et_2O 40:1).

^{1}**H NMR** (400 MHz, $CDCl_3$): δ 7.29 (d, *J* = 7.8 Hz, 1H), 7.22-7.09 (m, 8H), 6.32 (dt, *J* = 16.9, 9.8 Hz, 1H), 5.04-4.98 (m, 1H), 4.96 (d, *J* = 17.0 Hz, 1H), 3.23 (d, *J* = 9.6 Hz, 1H), 2.64-2.52 (m, 2H), 1.66-1.58 (m, 1H), 1.52-1.37 (m, 2H), 1.24 (s, 6H), 1.23 (s, 6H), 1.35-1.04 (m, 7H), 0.81 (t, *J* = 6.8 Hz, 3H) ppm.

^{13}**C NMR** (101 MHz, $CDCl_3$): δ 143.1, 142.4, 140.7, 131.7, 130.4, 128.5, 128.4, 128.1, 125.7, 115.5, 83.3, 56.3, 37.1, 33.5, 31.4, 27.0, 26.1, 25.3, 25.3, 23.6, 14.4 ppm.
Note: Carbon atom attached to boron is not visible due to quadrupolar relaxation.

^{11}**B NMR** (128 MHz, $CDCl_3$): δ 33.5 ppm.

HRMS (APCI+) m/z: calcd. for $C_{29}H_{40}BClO_2$ [*M* +H]$^+$: 467.2888, found: 467.2889.

2-((3*R,4*S**)-3-(4-Chlorophenyl)-4-methyloct-1-en-4-yl)-4,4,5,5-tetramethyl-1,3,2-dioxaborolane 7j**

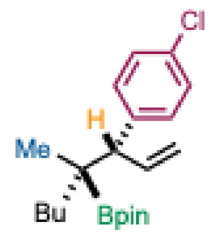

Prepared according to general procedure **GP4** from **2h** (95 mg, 200 μmol) and methyl iodide (37 μL, 600 μmol).

Yield: 40 mg (110 μmol, 55%, *dr* > 95:05 as determined by ^{1}H NMR spectroscopy) as a colorless oil.

R*f* = 0.60 (PE/Et_2O 40:1).

^{1}H NMR (400 MHz, $CDCl_3$): δ 7.22-7.18 (m, 4H), 6.17 (dt, *J* = 17.0, 9.8 Hz, 1H), 5.06 (d, *J* = 10.1 Hz, 1H), 5.00 (d, *J* = 17.0 Hz, 1H), 3.32 (d, *J* = 9.5 Hz, 1H), 1.49-1.36 (m, 2H), 1.35-1.19 (m, 4H), 1.17 (s, 6H), 1.08 (s, 6H), 0.96 (s, 3H), 0.86 (t, *J* = 6.9 Hz, 3H) ppm.

^{13}C NMR (101 MHz, $CDCl_3$): δ 142.1, 138.5, 131.8, 130.9, 128.0, 116.5, 83.3, 56.9, 37.8, 28.3, 25.4, 24.8, 23.8, 18.3, 14.3 ppm.
Note: Carbon atom attached to boron is not visible due to quadrupolar relaxation.

^{11}B NMR (128 MHz, $CDCl_3$): δ 33.2 ppm.

HRMS (APCI+) m/z: calcd. for $C_{21}H_{33}BClO_2$ [*M* +H]$^+$: 363.2257, found: 363.2273.

2-((3*R,4*R**)-4-Ethyl-3-phenylhepta-1,6-dien-4-yl)-4,4,5,5-tetramethyl-1,3,2-dioxaborolane 7k**

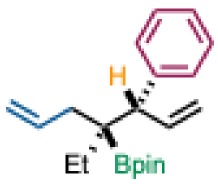

Prepared according to general procedure **GP4** from **2a** (74 mg, 175 μmol) and allyl tosylate (111 mg, 525 μmol).

Yield: 27 mg (82 μmol, 47%, *dr* > 95:05 as determined by ^{1}H NMR spectroscopy) as a colorless oil.

R*f* = 0.65 (PE/Et_2O 40:1).

^{1}H NMR (400 MHz, $CDCl_3$) δ 7.26-7.21 (m, 3H), 7.18-7.10 (m, 2H), 6.37 (dt, *J* = 16.7, 10.0 Hz, 1H), 5.85 (dddd, *J* = 16.8, 10.2, 8.0, 6.6 Hz, 1H), 5.09 (dt, *J* = 17.2, 1.9 Hz, 1H), 5.05-4.94 (m, 3H), 3.30 (d, *J* = 9.8 Hz, 1H), 2.37 (ddt, *J* = 14.4, 6.6, 1.6 Hz, 1H), 2.21 (dd, *J* = 14.4, 8.1 Hz, 1H), 1.40 (dt, *J* = 14.9, 7.3 Hz, 1H), 1.25 (s, 12H), 1.35-1.17 (m, 1H), 0.83 (t, *J* = 7.4 Hz, 3H).

^{13}C NMR (101 MHz, $CDCl_3$): δ 143.5, 140.8, 136.1, 129.2, 128.0, 126.1, 116.8, 115.5, 83.4, 56.6, 37.3, 25.4, 25.3, 24.9, 8.9 ppm.
Note: Carbon atom attached to boron is not visible due to quadrupolar relaxation.

^{11}**B NMR** (128 MHz, $CDCl_3$): δ 34.5 ppm.

HRMS (APCI+) m/z: calcd. for $C_{21}H_{32}BO_2$ [*M* +H]$^+$: 327.2490, found: 327.2509.

4,4,5,5-tetramethyl-2-((3*R,4*R**)-4-methyl-3-(*p*-tolyl)hepta-1,6-dien-4-yl)-1,3,2-dioxaborolane 7l**

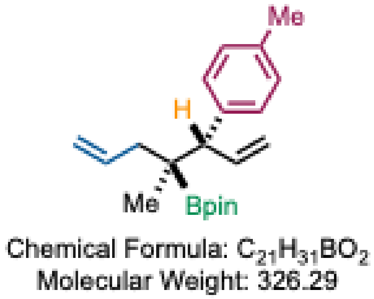

Prepared according to general procedure **GP4** from **2c** (72 mg, 175 µmol) and allyl tosylate (130 mg, 613 µmol).

Yield: 46 mg (141 µmol, 81%) as an inseparable mixture of diastereomers (*dr* 79:21 as determined by ^{1}H NMR spectroscopy) as a colorless oil.

R*f* = 0.65 (PE/Et_2O 40:1).

Major diastereomer:
^{1}**H NMR** (400 MHz, $CDCl_3$): δ 7.31-7.21 (m, 4H), 6.51 (dt, *J* = 17.0, 9.8 Hz, 1H), 5.95 (ddt, *J* = 17.3, 10.0, 7.3 Hz, 1H), 5.26 (d, *J* = 16.9 Hz, 1H), 5.22-5.17 (m, 1H), 5.17-5.11 (m, 2H), 3.48 (d, *J* = 9.5 Hz, 1H), 2.49 (s, 3H), 2.57-2.42 (m, 1H), 1.87 (dd, *J* = 13.3, 7.5 Hz, 1H), 1.43 (s, 12H), 1.08 (s, 3H) ppm.

^{13}**C NMR** (101 MHz, $CDCl_3$): δ 140.1, 139.01, 136.4, 135.6, 129.4, 128.6, 116.8, 115.9, 83.5, 57.6, 43.4, 25.3, 21.1, 17.8 ppm.
Note: Carbon atom attached to boron is not visible due to quadrupolar relaxation.

Minor diastereomer:
^{1}**H NMR** (400 MHz, $CDCl_3$): δ 7.35 (d, *J* = 8.0 Hz, 2H), 7.31-7.21 (m, 2H), 6.41 (dt, *J* = 16.9, 9.9 Hz, 1H), 6.09-5.98 (m, 1H), 5.26 (d, *J* = 16.9 Hz, 1H), 5.22-5.17 (m, 1H), 5.17-5.11 (m, 2H), 3.48 (d, *J* = 9.5 Hz, 1H), 2.49 (s, 3H), 2.57-2.42 (m, 1H), 2.14 (dd, *J* = 13.6, 7.8 Hz, 1H), 1.36 (s, 6H), 1.28 (s, 6H), 1.17 (s, 3H) ppm.

^{13}**C NMR** (101 MHz, $CDCl_3$): δ 139.03, 139.01, 136.9, 135.6, 129.3, 128.7, 116.6, 116.1, 83.3, 57.4, 42.6, 24.9, 21.1, 18.8.
Note: Carbon atom attached to boron is not visible due to quadrupolar relaxation.

^{11}**B NMR** (128 MHz, $CDCl_3$): δ 33.8 ppm.

HRMS (APCI+) m/z: calcd. for $C_{21}H_{32}BO_2$ [*M* +H]$^+$: 327.2490, found: 327.2503.

2-((4*R,5*R**)-4-Ethyl-2-methyl-5-phenylhepta-1,6-dien-4-yl)-4,4,5,5-tetramethyl-1,3,2-dioxaborolane 7m**

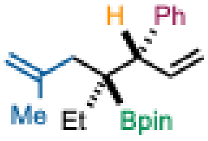

Prepared according to general procedure **GP4** from **2a** (39 mg, 95 µmol) and methallyl phosphate (59 mg, 285 µmol).

Yield: 15 mg (44 µmol, 46%) as an inseparable mixture of diastereomers (*dr* 80:20 as determined by ^{1}H NMR spectroscopy) as a colorless oil.

R*f* = 0.60 (PE/Et_2O 40:1).

Major diastereomer:
^{1}H NMR (400 MHz, $CDCl_3$): δ 7.33-7.21 (m, 3H), 7.20-7.08 (m, 2H), 6.44 (dt, *J* = 16.8, 9.8 Hz, 1H), 5.06-4.99 (m, 1H), 4.99-4.95 (m, 1H), 4.80 (s, 2H), 3.33 (d, *J* = 9.5 Hz, 1H), 2.40 (d, *J* = 14.8 Hz, 1H), 2.08 (d, *J* = 14.7 Hz, 1H), 1.77 (s, 3H), 1.56-1.46 (m, 1H), 1.42-1.32 (m, 1H), 1.29 (s, 6H), 1.25 (s, 6H), 0.84 (t, *J* = 7.4 Hz, 3H) ppm.

^{13}C NMR (101 MHz, $CDCl_3$): δ 144.6, 143.5, 141.1, 129.1, 128.0, 126.1, 115.5, 113.2, 83.5, 56.8, 41.9, 25.8, 25.6, 25.0, 23.9, 8.3 ppm.
Note: Carbon atom attached to boron is not visible due to quadrupolar relaxation.

Minor diastereomer:
^{1}H NMR (400 MHz, $CDCl_3$): δ 7.39-7.21 (m, 3H), 7.20-7.08 (m, 2H), 6.30 (dt, *J* = 18.7, 8.4 Hz, 1H), 5.06-4.99 (m, 1H), 4.99-4.95 (m, 1H), 4.75 (s, 1H), 4.69 (s, 1H), 3.40 (d, *J* = 9.6 Hz, 1H), 2.11 (d, *J* = 12.1 Hz, 1H), 1.70 (s, 3H), 1.67-1.60 (m, 1H), 1.56-1.46 (m, 1H), 1.42-1.32 (m, 1H), 1.26 (s, 6H), 1.24 (s, 6H), 0.92 (t, *J* = 7.3 Hz, 3H) ppm.

^{13}C NMR (101 MHz, $CDCl_3$) δ 144.8, 143.5, 141.0, 129.7, 127.9, 126.1, 115.3, 113.2, 83.5, 57.1, 40.3, 25.6, 25.5, 25.2, 23.9, 9.1 ppm.
Note: Carbon atom attached to boron is not visible due to quadrupolar relaxation.

^{11}B NMR (128 MHz, $CDCl_3$): δ 34.7 ppm.

HRMS (APCI+) m/z: calcd. for $C_{22}H_{34}BO_2$ [*M* +H]$^+$: 341.2646, found: 341.2654.

(3*S,4*R**)-3-Ethyl-2-methyl-4-phenyl-3-(4,4,5,5-tetramethyl-1,3,2-dioxaborolan-2-yl)hex-5-en-2-ol 7n**

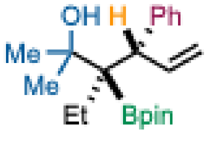

Prepared according to general procedure **GP4** from **2a** (76 mg, 186 µmol) and acetone (69 µL, 930 µmol).

Yield: 20 mg (58 µmol, 31%) as a partially separable mixture of diastereomers (*dr* 81:19 as determined by ^{1}H NMR spectroscopy) as a colorless oil.

R*f* = 0.20 (PE/Et_2O 40:1).

Major diastereomer:
^{1}**H NMR** (400 MHz, $CDCl_3$): δ 7.40 (d, *J* = 7.2 Hz, 2H), 7.28-7.21 (m, 2H), 7.21-7.12 (m, 1H), 6.66 (dt, *J* = 17.9, 9.2 Hz, 1H), 5.02 (s, 1H), 4.98 (d, *J* = 4.9 Hz, 1H), 3.79 (d, *J* = 9.2 Hz, 1H), 2.62 (br s, 1H), 1.77-1.63 (m, 1H), 1.53 (dt, *J* = 14.9, 7.5 Hz, 1H), 1.32 (s, 6H), 1.30 (s, 6H), 1.15 (s, 3H), 1.10 (s, 3H), 0.88 (t, *J* = 7.5 Hz, 3H).

^{13}**C NMR** (101 MHz, $CDCl_3$): δ 144.1, 143.4, 129.9, 128.2, 126.2, 115.5, 83.6, 76.5, 53.9, 28.2, 25.4, 25.2, 24.3, 11.8 ppm.
Note: Carbon atom attached to boron is not visible due to quadrupolar relaxation.

Minor diastereomer:
^{1}**H NMR** (400 MHz, $CDCl_3$): δ 7.38 (d, *J* = 6.9 Hz, 2H), 7.28-7.21 (m, 2H), 7.21-7.12 (m, 1H), 6.50 (dt, *J* = 16.8, 10.0 Hz, 1H), 5.05-5.00 (m, 1H), 5.00-4.89 (m, 1H), 3.82 (d, *J* = 9.3 Hz, 1H), 2.96 (s, 1H), 1.89-1.83 (m, 2H), 1.26 (s, 6H), 1.19 (s, 6H), 1.14 (s, 3H), 1.06 (s, 3H), 0.93 (t, *J* = 7.4 Hz, 3H).

^{13}**C NMR** (101 MHz, $CDCl_3$): δ 144.1, 141.8, 129.8, 128.3, 126.2, 115.2, 83.4, 76.3, 54.6, 30.4, 25.3, 25.2, 24.7, 13.0 ppm.
Note: Carbon atom attached to boron is not visible due to quadrupolar relaxation.

^{11}**B NMR** (128 MHz, $CDCl_3$): δ 34.0 ppm.

HRMS (APCI+) m/z: calcd. for $C_{21}H_{32}BO_2$ [M–H_2O+H]$^+$: 327.2490, found: 327.2496.

4,4,5,5-tetramethyl-2-((3*S,4*S**)-3-(Methylthio)-4-phenylhex-5-en-3-yl)-1,3,2-dioxaborolane 7o**

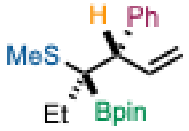

Chemical Formula: $C_{19}H_{29}BO_2S$
Molecular Weight: 332.31

Prepared according to general procedure **GP4** from **2a** (66 mg, 160 µmol) and dimethyl disulfide (43 µL, 480 µmol).

Yield: 21 mg (73 µmol, 46%) as an inseparable mixture of diastereomers (*dr* 88:12 as determined by ^{1}H NMR spectroscopy) as a colorless oil.

R*f* = 0.52 (PE/Et_2O 40:1).

Major diastereomer:
^{1}**H NMR** (400 MHz, $CDCl_3$): δ 7.43-7.37 (m, 2H), 7.30-7.12 (m, 3H), 6.52 (dt, *J* = 16.9, 9.8 Hz, 1H), 5.14-4.95 (m, 2H), 3.52 (d, *J* = 9.4 Hz, 1H), 1.91 (s, 3H), 1.68 (dt, *J* = 14.6, 7.3 Hz, 1H), 1.48 (dt, *J* = 14.7, 7.3 Hz, 1H), 1.24 (s, 12H), 0.98 (t, *J* = 7.4 Hz, 3H) ppm.

^{13}**C NMR** (101 MHz, $CDCl_3$): δ 142.7, 139.8, 129.8, 129.2, 128.2, 127.9, 126.6, 116.4, 115.9, 83.8, 56.5, 55.4, 26.5, 25.1, 24.9, 24.6, 11.7, 9.0 ppm.
Note: Carbon atom attached to boron is not visible due to quadrupolar relaxation.

Minor diastereomer:
^{1}**H NMR** (400 MHz, $CDCl_3$): δ 7.43-7.37 (m, 2H), 7.30-7.12 (m, 3H), 6.52 (dt, *J* = 16.9, 9.8 Hz, 1H), 5.14-4.95 (m, 2H), 3.64 (d, *J* = 9.1 Hz, 1H), 1.78 (s, 3H), 1.68 (dt, *J* = 14.6, 7.3 Hz, 1H), 1.48 (dd, *J* = 14.7, 7.3 Hz, 1H), 1.24 (s, 12H), 0.98 (t, *J* = 7.4 Hz, 3H).

^{13}**C NMR** (101 MHz, $CDCl_3$): δ 142.7, 139.8, 129.8, 127.9, 126.6, 116.4, 83.8, 55.4, 26.5, 25.1, 24.9, 11.7, 9.0 ppm.
Note: Carbon atom attached to boron is not visible due to quadrupolar relaxation.

^{11}**B NMR** (128 MHz, $CDCl_3$): δ 32.8 ppm.

HRMS (APCI+) m/z: calcd. for $C_{19}H_{30}BO_2S^+$ [*M*+H]$^+$: 333.2054, found: 333.2040.

2-((3*R,4*R**)-3-Benzyl-4-phenylhex-5-en-3-yl)-4,4,5,5-tetramethyl-1,3,2-dioxaborolane 7p**

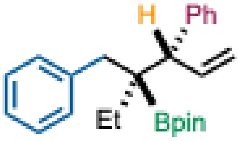

Chemical Formula: $C_{25}H_{33}BO_2$
Molecular Weight: 376.35

Prepared according to general procedure **GP4** from **2a** (103 mg, 250 μmol) and benzyl chloride (95 mg, 750 μmol).

Yield: 59 mg (157 μmol, 63%, *dr* > 95:05 as determined by ^{1}H NMR spectroscopy) as a colorless oil.

R*f* = 0.35 (PE/Et_2O 20:1).

^{1}**H NMR** (400 MHz, $CDCl_3$): δ 7.37-7.06 (m, 9H), 6.47 (dt, *J* = 17.0, 9.8 Hz, 1H), 5.21-4.98 (m, 2H), 3.32 (d, *J* = 9.3 Hz, 1H), 3.02 (d, *J* = 13.7 Hz, 1H), 2.69 (d, *J* = 13.7 Hz, 1H), 1.42 (dd, *J* = 13.0, 5.7 Hz, 1H), 1.26 (s, 6H), 1.17 (s, 6H), 0.92 (t, *J* = 7.3 Hz, 3H) ppm.

^{13}**C NMR** (101 MHz, $CDCl_3$): δ 143.5, 141.3, 140.0, 130.9, 129.2, 128.0, 127.8, 126.1, 125.9, 115.9, 83.5, 56.6, 38.7, 25.9, 25.2, 23.9, 8.9 ppm.
Note: Carbon atom attached to boron is not visible due to quadrupolar relaxation.

^{11}**B NMR** (128 MHz, $CDCl_3$): δ 34.5 ppm.

HRMS (APCI+) m/z: calcd. for $C_{25}H_{34}BO_2^+$ [*M*+H]$^+$: 377.2652, found: 377.2655.

2,2'-((2*R,3*R**)-2-Methyl-3-(*p*-tolyl)pent-4-ene-1,2-diyl)bis(4,4,5,5-tetramethyl-1,3,2-dioxaborolane) 7q**

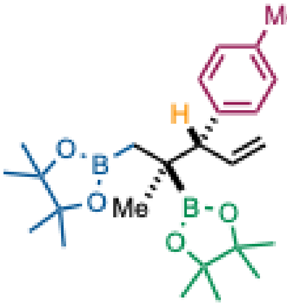

Chemical Formula: $C_{25}H_{40}B_2O_4$
Molecular Weight: 426.21

Prepared according to general procedure **GP4** from **2c** (82 mg, 200 µmol) and $PinBCH_2Cl$ (106 mg, 600 µmol).

Yield: 32 mg (75 µmol, 38%) as an inseparable mixture of diastereomers (*dr* 75:25 as determined by ^{1}H NMR spectroscopy) as a colorless oil.

R*f* = 0.15 (PE/Et_2O 20:1).

Major diastereomer:
^{1}H NMR (400 MHz, $CDCl_3$): δ 7.08 – 7.00 (m, 4H), 6.31 (dt, *J* = 16.9, 9.9 Hz, 1H), 5.11 – 4.96 (m, 2H), 3.25 (d, *J* = 9.4 Hz, 1H), 2.28 (s, 3H), 1.42 – 1.09 (m, 24H), 1.04 – 0.96 (m, 1H), 0.93 (s, 3H), 0.56 (d, *J* = 15.4 Hz, 1H) ppm.

^{13}C NMR (101 MHz, $CDCl_3$): δ 140.2, 139.1, 135.41, 129.6, 128.5, 115.7, 83.3, 82.9, 59.1, 25.4, 25.3, 25.2, 24.7, 21.1, 20.0 ppm.
Note: Carbon atoms attached to boron are not visible due to quadrupolar relaxation.

Minor diastereomer:
^{1}H NMR (400 MHz, $CDCl_3$): δ 7.11 (d, *J* = 8.0 Hz, 1H), 7.08 – 7.00 (m, 1H), 6.31 – 6.15 (m, 1H), 5.11 – 4.96 (m, 2H), 3.25 – 3.16 (m, 1H),.28 (s, 3H), 1.42 – 1.09 (m, 24H), 1.04 – 0.96 (m, 1H), 0.93 (s, 3H), 0.67 (d, *J* = 15.3 Hz, 1H) ppm.

^{13}C NMR (101 MHz, $CDCl_3$) δ 139.7, 139.1, 135.43, 129.5, 128.5, 116.0, 83.3, 82.8, 59.1, 25.3, 25.2, 25.1, 24.5, 22.0, 20.0 ppm.
Note: Carbon atoms attached to boron is not visible due to quadrupolar relaxation.

^{11}B NMR (128 MHz, $CDCl_3$): δ 33.3, 31.8 ppm.

HRMS (APCI+) m/z: calcd. for $C_{25}H_{41}B_2O_4^+$ [*M*+H]$^+$: 427.3191, found: 427.3191.

4,4,5,5-Tetramethyl-2-(1-(1-phenylallyl)cyclobutyl)-1,3,2-dioxaborolane 7r

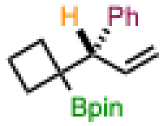

Chemical Formula: $C_{19}H_{27}BO_2$
Molecular Weight: 298.23

Prepared according to general procedure **GP4** from **2i** (92 mg, 200 µmol).

Yield: 43 mg (144 µmol, 72%) as a colorless oil.

R*f* = 0.60 (PE/Et_2O 20:1).

^{1}H NMR (400 MHz, $CDCl_3$) δ 7.37-7.09 (m, 5H), 6.30-6.04 (m, 1H), 5.23-4.94 (m, 2H), 3.37 (d, *J* = 8.9 Hz, 1H), 2.14-1.96 (m, 4H), 1.96-1.84 (m, 1H), 1.80-1.67 (m, 1H), 1.13 (s, 12H) ppm.

^{13}C NMR (101 MHz, $CDCl_3$) δ 143.6, 139.8, 128.5, 128.1, 126.1, 115.8, 83.2, 56.8, 28.5, 27.8, 24.7, 24.6, 18.2 ppm.
Note: Carbon atom attached to boron is not visible due to quadrupolar relaxation.

^{11}B NMR (128 MHz, $CDCl_3$): δ 34.4 ppm.

HRMS (APCI+) m/z: calcd. for $C_{19}H_{28}BO_2^+$ [*M*+H]$^+$: 299.2182, found: 299.2201.

(1*R,2*S**,3*S**)-1,2-dimethyl-2-(4,4,5,5-tetramethyl-1,3,2-dioxaborolan-2-yl)-3-(4-(trifluoromethyl)phenyl)-3-vinylcyclobutan-1-ol 7s**

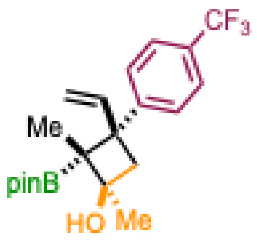

Chemical Formula: $C_{21}H_{28}BF_3O_3$
Molecular Weight: 396.26

Prepared according to general procedure **GP4** from **2n** (125 mg, 240 µmol).

Yield: 32 mg (81 µmol, 34%) as a partially separable mixture of diastereomers (*dr* > 75:24:01 as determined by ^{1}H NMR spectroscopy) as a colorless oil.

R*f* = 0.25 (PE/Et_2O 9:1).

Major diastereomer:

^{1}H NMR (400 MHz, $CDCl_3$): δ 7.49 (d, *J* = 8.1 Hz, 2H), 7.14 (d, *J* = 8.0 Hz, 2H), 6.53 (dd, *J* = 17.4, 10.6 Hz, 1H), 5.14 (d, *J* = 10.8 Hz, 1H), 4.92 (d, *J* = 17.4 Hz, 1H), 3.01 (d, *J* = 12.1 Hz, 1H), 2.54 (d, *J* = 12.1 Hz, 1H), 2.22 (br s, 1H), 1.31 (s, 3H), 1.28 (s, 3H), 1.02 (s, 6H), 0.75 (s, 6H) ppm.

^{13}C NMR (126 MHz, $CDCl_3$): δ 152.5, 145.5, 127.8, 126.8 (q, J_{C-F} = 275.8 Hz), 126.4, 125.2 (q, J_{C-F} = 4.1 Hz) 123.4, 114.0, 83.4, 75.9, 50.1, 41.3, 25.7, 24.8, 24.7, 12.9 ppm.
Note: Carbon atom attached to boron is not visible due to quadrupolar relaxation.

^{11}B NMR (128 MHz, $CDCl_3$): δ 33.3 ppm.

^{19}F NMR (377 MHz, $CDCl_3$) δ –62.3 ppm.

^{1}H-^{1}H NOEsy NMR – main resonances (^{1}H NMR shifts are displayed in ppm)

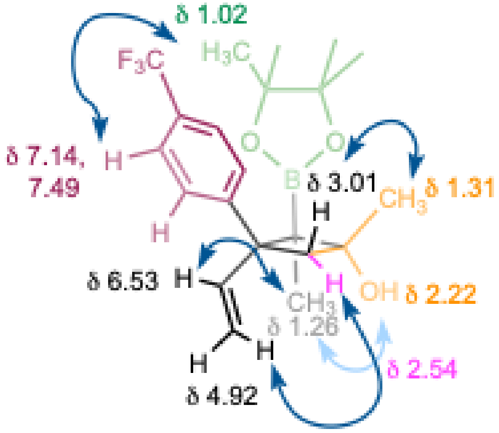

HRMS (APCI+) m/z: calcd. for $C_{21}H_{29}BF_3O_3^+$ [*M*+H]$^+$: 397.2156, found: 397.2173.

4. Computational Details

The calculations were performed using Orca 5.1.[9] For geometry optimizations and frequency calculations, functional BP86[10-12]-D3(bj)[13] with def2-SVP[14] basis set was used, denoted as the M2 method. For single-point calculations M062x[15]-D3(0) with def2-TZVP[14] were used, denoted as M1 method. All calculations (optimizations, frequency calculations, and single point energies) were performed in THF using the CPCM solvation model. PB86 functional in ORCA was treated with the RI-J approximation, whereas M062X was treated with the chain-of-sphere approximation[16] to evaluate exchange integrals (RIJCOSX[16]). The optimized minima and transition states were verified by harmonic vibrational analysis. All main transition-state structures were confirmed to connect corresponding reactants and products. The geometries of the lowest energy conformers of the discussed intermediates and transition states were refined (reoptimized) using M06L-D3(0) or M062X-D3(0) functionals with def2-SVP basis set, denoted as M3 and M4 methods. The electronic single-point energies were calculated by M1 with the inclusion of zero-point, enthalpy, and entropic corrections determined from vibrational frequencies calculated with M2-M4 methods (M1//M2-4).
Standard state correction of free energy: change going from the state of 1 mol of an ideal gas at 1 atm to the 1 M solution phase standard state, which is 1.89 kcal/mol at 298 K. The $\Delta G1atm \rightarrow 1M$ term affects the free energy of the reactions where molecularity is changed by Δn ($\Delta n \cdot \Delta G1atm \rightarrow 1M$).[17]
Transition state theory and Curtin-Hammet principle:
The selectivity of the reactions was analyzed using **Transition State Theory (TST)**. For reactions involving rapidly equilibrating intermediates, the **Curtin-Hammett principle** was also invoked. According to this framework, the product distribution is not governed by the relative populations of the ground-state conformers or intermediates. Instead, it is dictated by the difference in the Gibbs free energies of the competing transition states ($\Delta\Delta G^\ddagger$). The pathway with the lower free energy transition state is assumed to be the dominant one.
Neglecting Maxwell-Boltzmann distribution effects: In this work, a common and pragmatic simplification was employed, wherein selectivity was predicted by comparing the calculated free energies of only the single lowest-energy transition state structure for each pathway without accounting for the Boltzmann distributions of the studied transition state ensembles. It is assumed that either:

- The single, lowest-energy transition state conformer is overwhelmingly dominant for each pathway, making contributions from higher-energy conformers negligible.
- Alternatively, the conformational free energy contribution (the energy penalty from Boltzmann averaging multiple conformers) is similar for both competing transition states. In this case, the error introduced by neglecting this averaging would systematically cancel when calculating the relative energy difference ($\Delta\Delta G^\ddagger$), thus not affecting the predicted selectivity.

4.1 Conformational search and analysis

To find the lowest energy conformers, we employed a multi-step computational protocol, previously developed by our research team.[18] All intermediates and transition states were found with this protocol.
Conformational searches were performed using the CREST program[19], utilizing rapid semi-empirical GFN2-xTB calculations to efficiently generate an ensemble of conformers. These conformers were refined through a structured three-phase computational protocol (Scheme S1), gradually narrowing the number of candidates from an initial set of 40–130 to 6–10 stable structures. Final conformers with the lowest free energies were selected for constructing potential energy surfaces. Conformers within the electronic energy window of 15 kcal/mol (EEW1) were advanced. (Scheme S1)
Phase 1: Initial structures, guided by chemical intuition, were first optimized using method M2 and subsequently were used as starting points for conformational exploration. The CREST program was then employed to generate multiple conformers. Conformers within an electronic energy window of 15 kcal/mol (EEW1) were advanced.
Phase 2: The selected conformers underwent partial geometry optimization (method M2). Single-point energies were then calculated at a higher theoretical level using method M062X-d3(0)/def2-SVP. Conformers within a narrower window of 3 kcal/mol (EEW2) proceeded to Phase 3.
In the cases of transition states, this phase was modified by freezing specific parameters to ensure that the relative positions of active atoms did not change.

Phase 3: Full geometry optimizations and frequency analyses (methods M2, M3, or M4) were performed, including validation of minima and transition states. Then, single-point energies were calculated (method M1), yielding final electronic, enthalpic, and free energies (M1//M2-4).

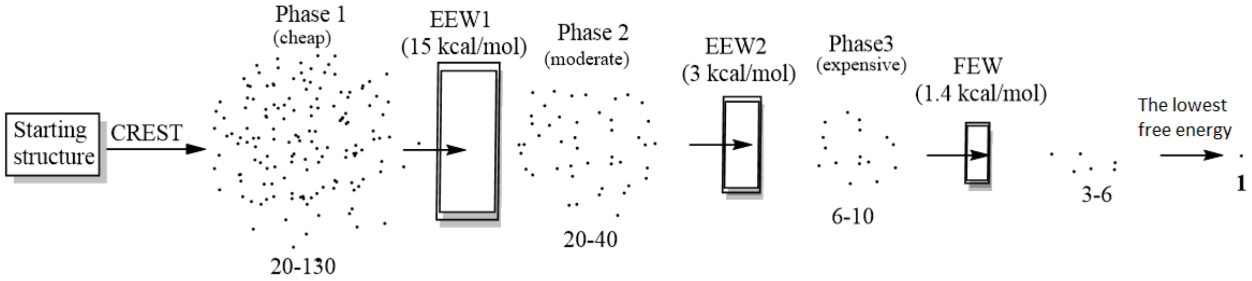

Scheme S1. Illustration of the workflow - proceeding through the phases with shrinking energy window filters and converging to the lowest energy isomer

4.2 Thermodynamics of the reaction

Before we started the mechanistic investigation, we studied the overall thermodynamics of the process. The reaction energies from the lithiated cyclopropyls to the silyl-substituted products are highly exergonic (ΔG values and ΔH values in brackets, Scheme S2).

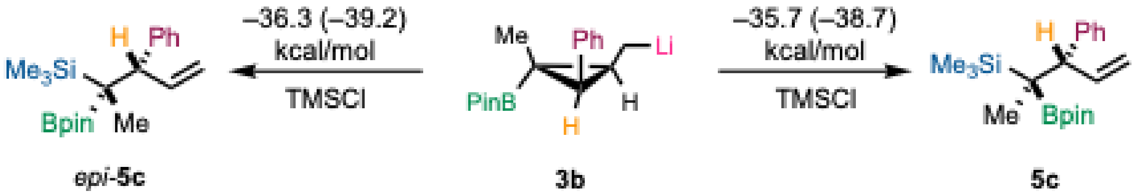

Scheme S2. Thermochemistry of the transformation. M062x-D3(0)/def2-TZVP/CPCM(THF)// M062x-D3(0)/def2-SVP/CPCM(THF).

4.3 Ring opening of cyclopropylmethyl lithium

4.3.1 Formation of borata alkene intermediate **4b**

The first step in the transformation is the ring-opening reaction of cyclopropylmethyl lithium species **3**. To simplify the calculations, we worked with a model using 1,3,2-dioxaboronic ester instead of 4,4,5,5-tetramethyl-1,3,2-dioxaboronic ester (a pinacol boronic ester without the methyl groups). We found that ring-opening reaction is nearly barrierless and exergonic (**TS_1b** 0.8 kcal/mol), leading to **4b’,** followed by a further decrease in energy, adopting the lowest isomer geometry **4b** (Scheme S3, ΔG values and ΔH values in brackets).

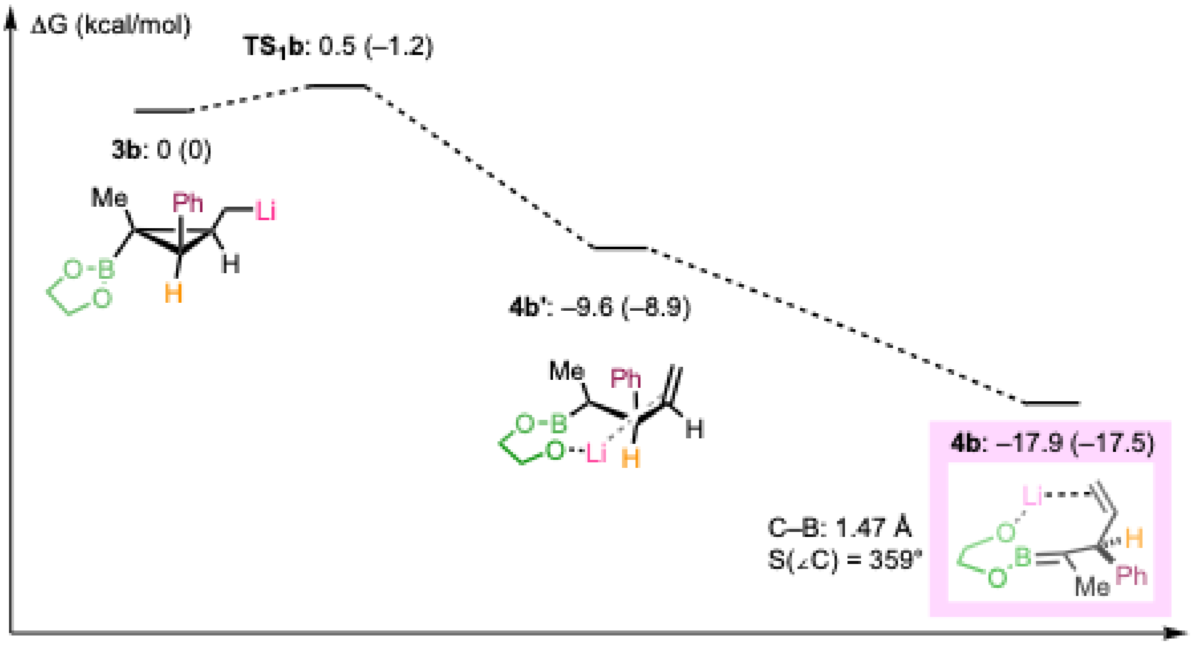

Scheme S3. Ring opening reaction mechanism. M062x-D3(0)/def2-TZVP/CPCM(THF)// M062x-D3(0)/def2-SVP/CPCM(THF).

During the conformational search for the ring-opening transition state, we identified another possible transition state, **STS₂**, that could lead directly to **4b**. However, its corresponding starting material is **S1**, which is the opposite diastereomer to our starting material **3b**. However, **S1** is elusive in the solution as it is 5.2 kcal/mol higher in energy compared to **4b** (Scheme S4, ΔG values and ΔH values in brackets).

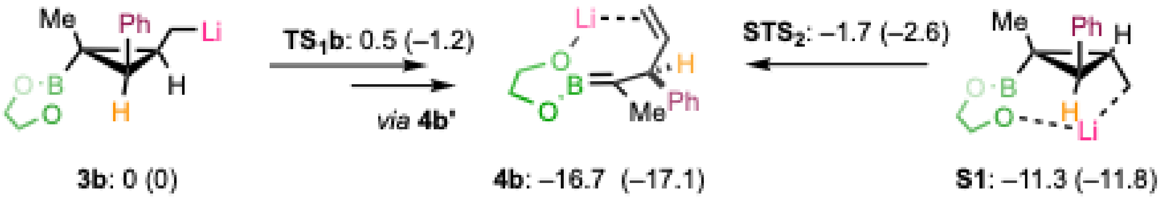

Scheme S4. Direct pathway towards intermediate **4b**.

4.3.2 Formation of benzylic anion **8b**

The ring-opening is regioselective towards the cleavage of C_2-C_3 bond and formation of **4b**. We computed the benzylic anion **8b** resulting from the disfavored C_1-C_2 opening, and found its energy is 8.3 kcal/mol higher than **4b** (Scheme S5). The high energy difference supports the sole formation of **4b**.
To isolate the effect of boryl group versus phenyl group on the stabilization of α-carbanion, we compared the anions without coordinated lithium cations. We found that **4b-free** is 5.8 kcal/mol lower in energy than **8b-free**, indicating that boryl group stabilizing an adjacent carbanion more than a phenyl group.

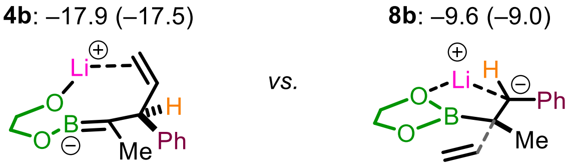

Scheme S5. Comparison between **4b** and **8b**

4.4 Silylation reaction mechanism via monomer intermediate

4.4.1 Free anion model

Following the ring-opening reaction studies, we proceeded to investigate the silylation reaction, aiming to explain the observed diastereoselectivity. Given the expected significant steric influence of the pinacol boronic ester, we chose to work with the full pinacol boron. We initiated our attempts with a simplified model excluding the lithium cation. We found transition states corresponding to a back-side attack on the silicon. Unfortunately, the energy difference between the diastereomeric transition states was low (only 1.2 kcal/mol), which is close to the calculation error (Scheme S6). As a result, we decided to include the lithium cations in the calculations.

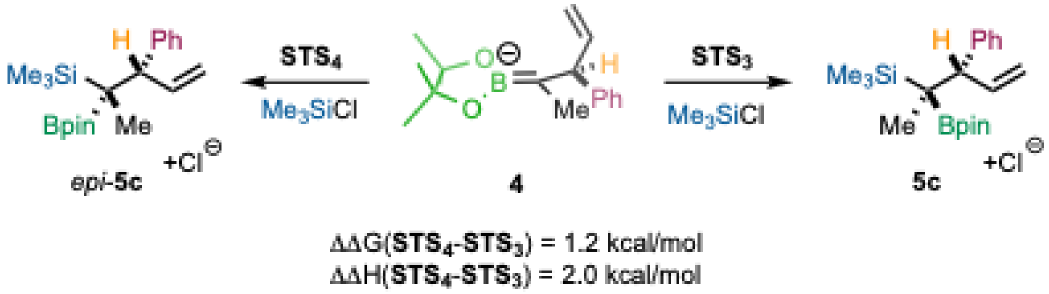

Scheme S6. Silylation reaction with free anion model; level of theory - M062x-D3(0)/def2-TZVP/CPCM(THF)// M06L-D3(0)/def2-SVP.

4.4.2 Lithium-coordinated anion model

Evaluation of electrophilic attack modes on Me_3SiCl

When the lithium cation is taken into consideration, there are several possibilities for the silyl trapping reactions (scheme S7):

1. Back-side attack on the silicon (**STS_5**).
2. Six-membered transition state leading to penta-coordinated silicon intermediate (**STS_6**).
3. Lithium cation-assisted back-side attack on the silicon (**STS_7**).

Among these three options, transition state **STS_7** exhibited the highest energy, likely due to the loss of stabilizing lithium-oxygen coordination present in both **STS_5** and **STS_6**. Furthermore, **STS_5** was 4 kcal/mol lower in energy than **STS_6**. This energy difference suggests that the stabilization gained from the Li-Cl interaction in **STS_6** is outweighed by the energetic penalty associated with a frontside electrophilic attack compared to a backside attack. Notably, the relative energy ordering of these three types of transition states remained consistent throughout all subsequent conformational analyses within all models.

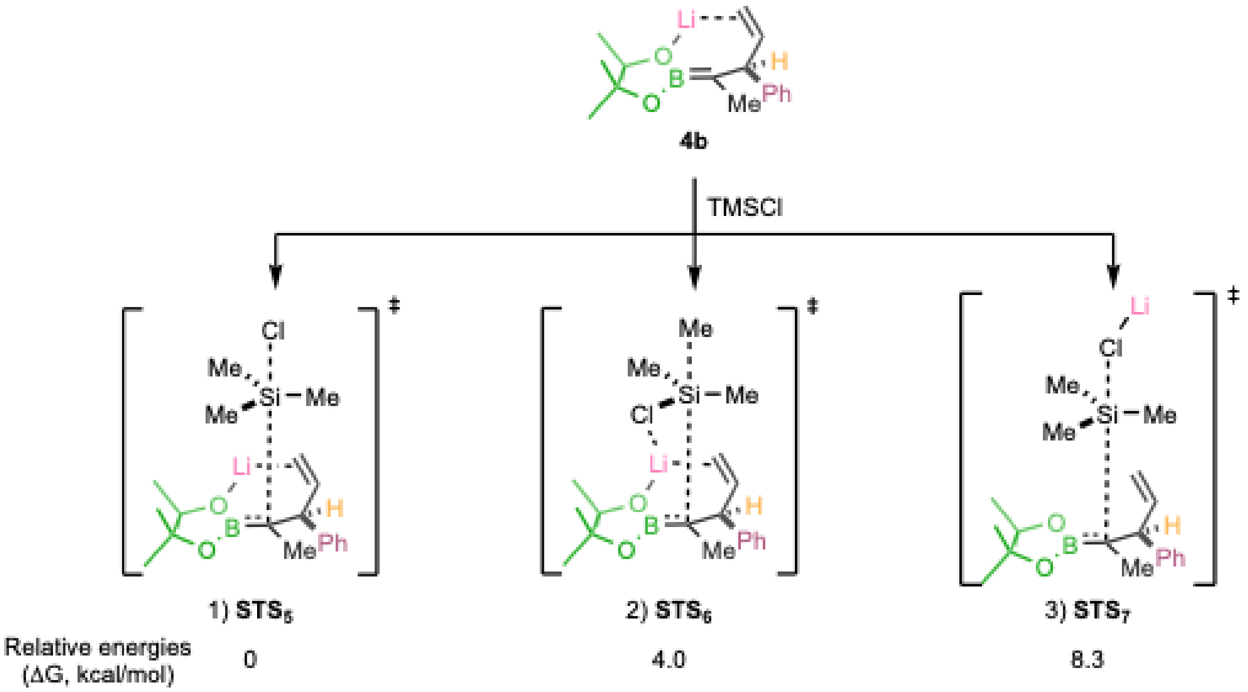

Scheme S7. Options for the silylation transition state. Level of theory M062x-D3(0)/def2-TZVP/CPCM(THF)// BP86-D3(bj)/def2-svp/CPCM(THF).

Investigation of the silylation selectivity

A conformational search including the two preferred modes (types 1 and 2) revealed that the lowest energy transition states are **STS$_8$** and **STS$_9$** (both type 1). Unfortunately, this analysis could not explain the observed selectivity (Scheme S8), as it resulted in reversed selectivity compared to experimental observations.

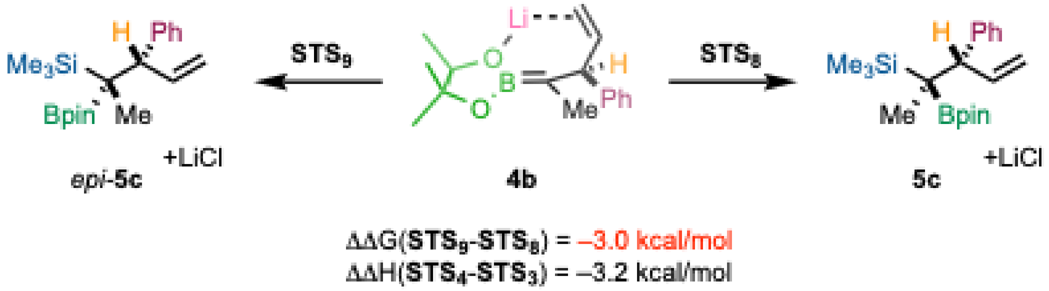

Scheme S8. Silylation reaction with lithium-coordinated anion model.

Study of explicit THF solvation

Since the tight-ion pair model did not match the experimental results, we sought to shift towards more separated ion pair model by incorporating explicit THF solvent molecules. We found that explicit solvation with one THF molecule is favorable by -8.1 kcal/mol. Adding a second solvent molecule further stabilizes the system by an additional 3.7 kcal/mol, resulting in an overall stabilization of **4b** by 11.8 kcal/mol (Scheme S9, ΔG values and ΔH values in brackets). The addition of a third THF was found to be endergonic (relative to **4b*2THF**); therefore, the tri-solvated model was not considered further.

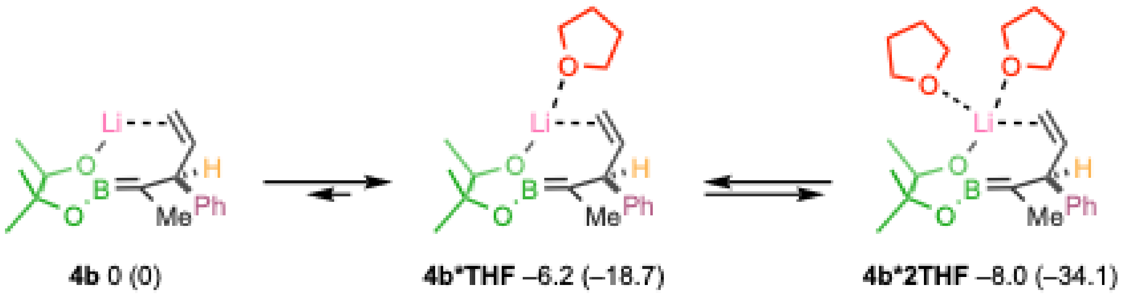

Scheme S9. Solvation of **4b** with one and two THF molecules.

We then investigated the mono-solvated silylation reaction transition states. Conformational searches revealed that the lowest-energy conformers, regardless of the side of attack, always possess an interaction between lithium and one of the oxygen atoms of the pinacol boronic ester, resulting in pairs of transition states for each case. Since there are two diastereomers, there are overall four transition states (Scheme S10).
For the mono-solvated case, the lowest transition states were identified as **STS_{13}** and **STS_{11}**, leading to **5c** and *epi*-**5c**, respectively. The energy difference is 0.9 kcal/mol, too low to be conclusive. Hence, we then computed the di-solvated model (Scheme S10, ΔG values and ΔH values in brackets).

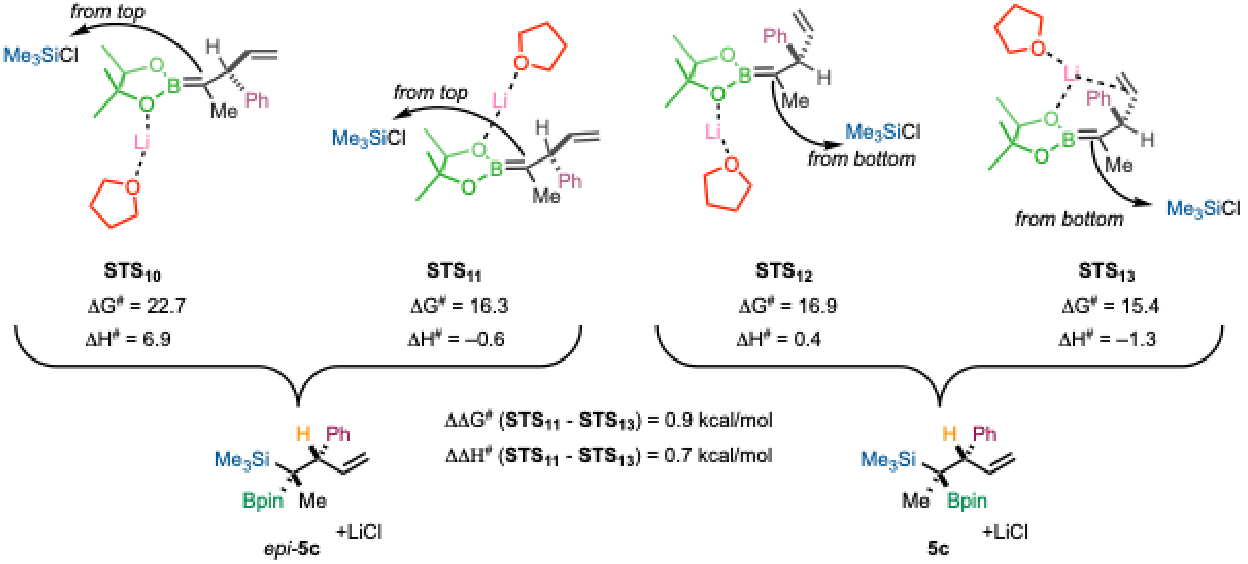

Scheme S10. Silylation reaction with one THF molecule model. M062x-D3(0)/def2-TZVP/CPCM(THF)// M06L-D3(0)/def2-SVP/ CPCM(THF)

In the di-solvated model, the preferred structures still possessed the oxygen-lithium interaction, similar to the mono-solvated model, resulting in two pairs of transition states. The lowest energy transition states were found to be **TS_2** and **TS_3**, producing **5c** and *epi*-**5c**, respectively. The energy difference is 2.0 kcal/mol, in good correlation with the experimental results (Scheme S11, ΔG values and ΔH values in brackets). The selectivity can be attributed to the coordination between the lithium cation and the vinyl group, which is dominant for **TS_2** but less accessible for **TS_3**.

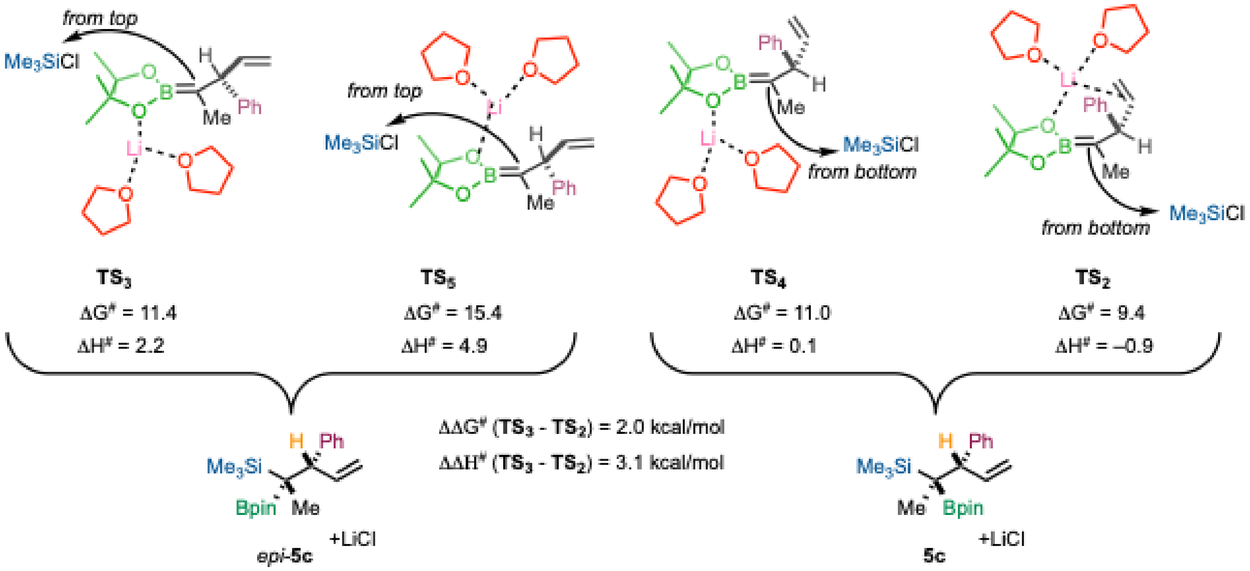

Scheme S11. Silylation reaction with two THF molecules model. M062x-D3(0)/def2-TZVP/CPCM(THF)// M06L-D3(0)/def2-SVP/CPCM(THF)

5. NMR spectra

((1*S**,2*R**,3*R**)-2-Methyl-3-phenyl-2-(4,4,5,5-tetramethyl-1,3,2-dioxaborolan-2-yl)cyclopropyl)methanol **2b**-OH

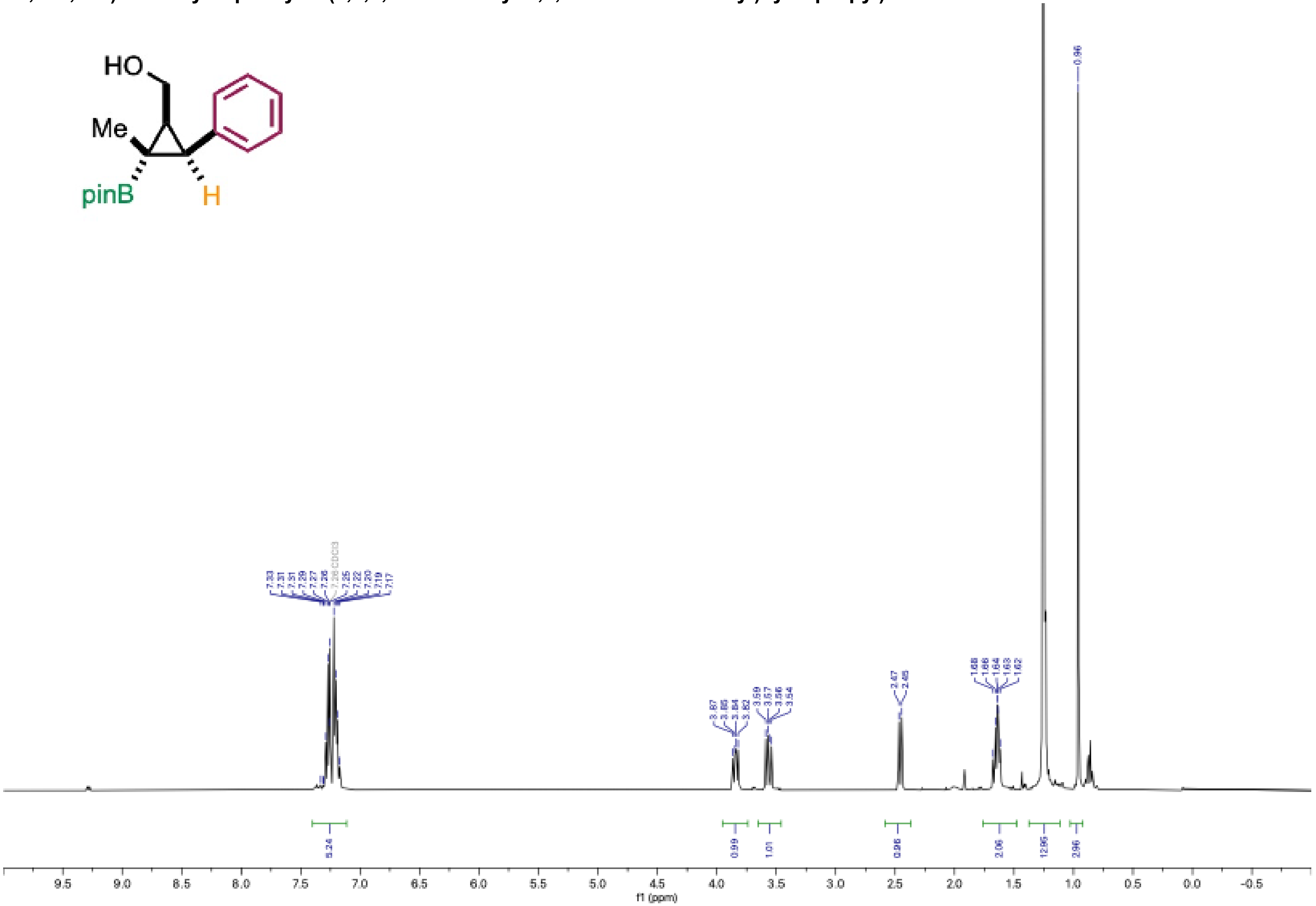

^{1}**H NMR** spectrum (400 MHz, $CDCl_3$)

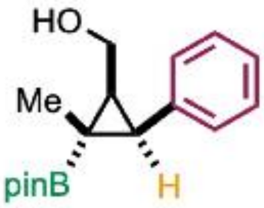

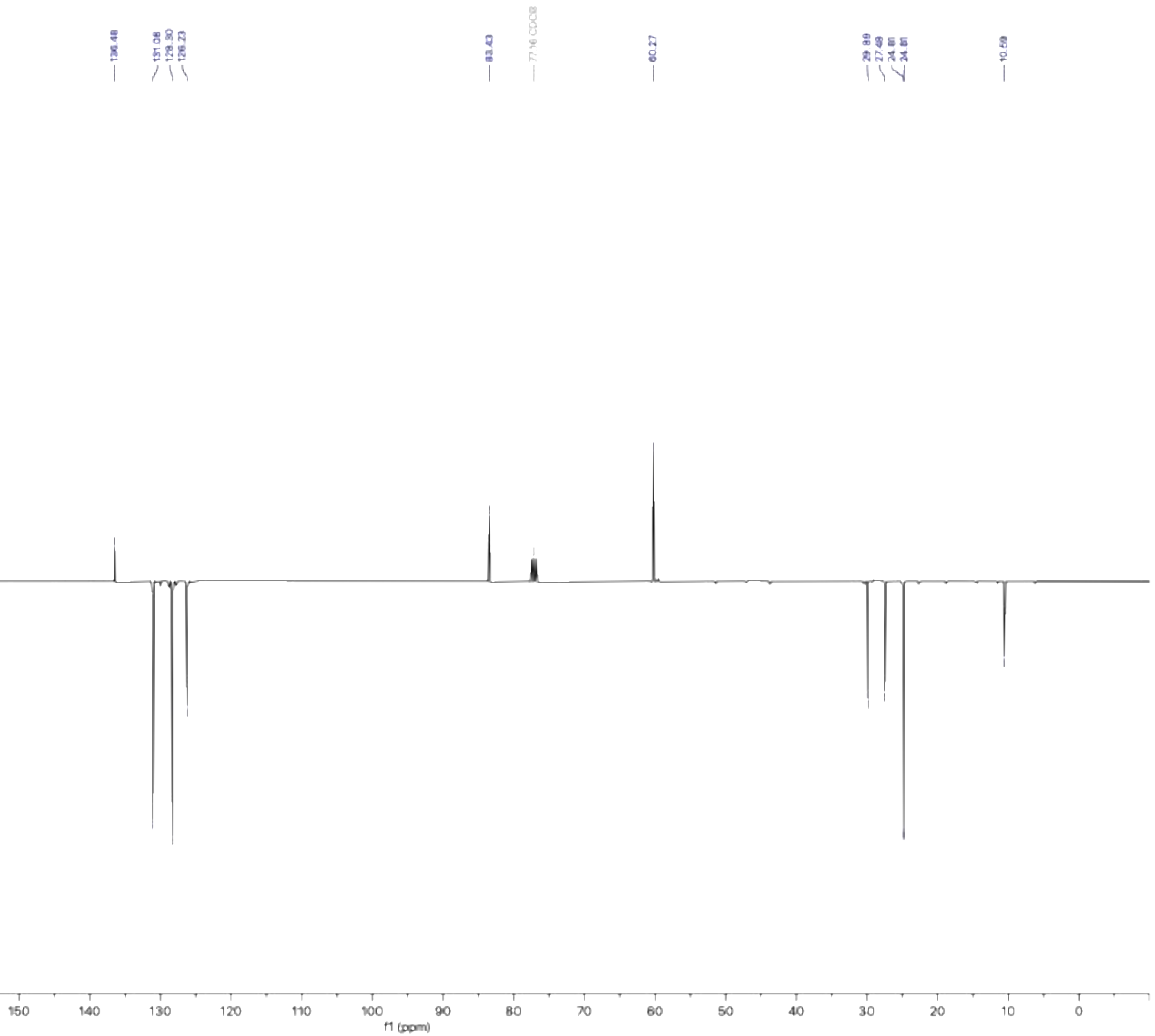

^{13}C NMR spectrum (101 MHz, $CDCl_3$)

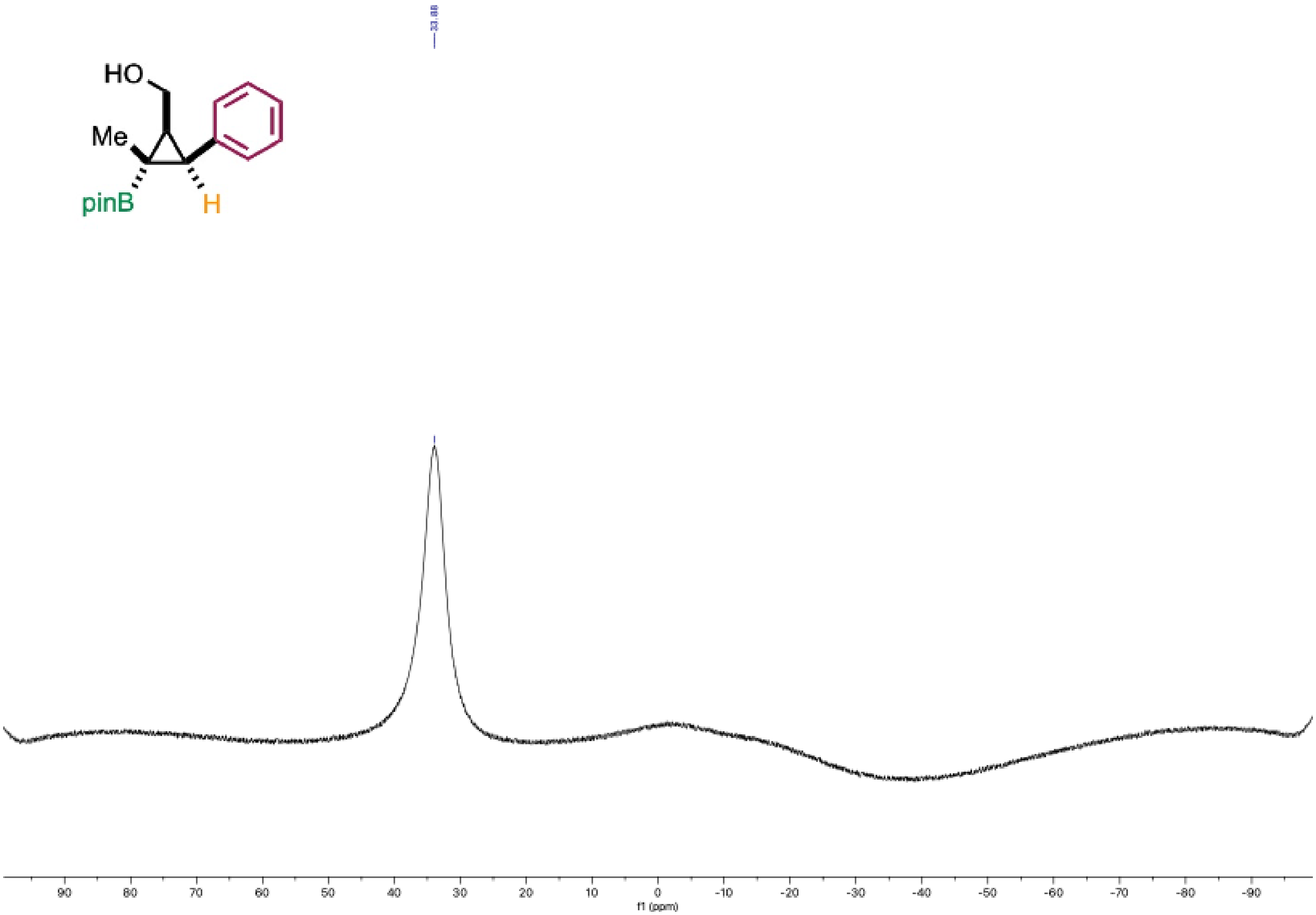

^{11}B NMR spectrum (128 MHz, $CDCl_3$)

((1*S**,2*R**,3*R**)-2-Methyl-2-(4,4,5,5-tetramethyl-1,3,2-dioxaborolan-2-yl)-3-(*p*tolyl)cyclopropyl)methanol **2c**-OH

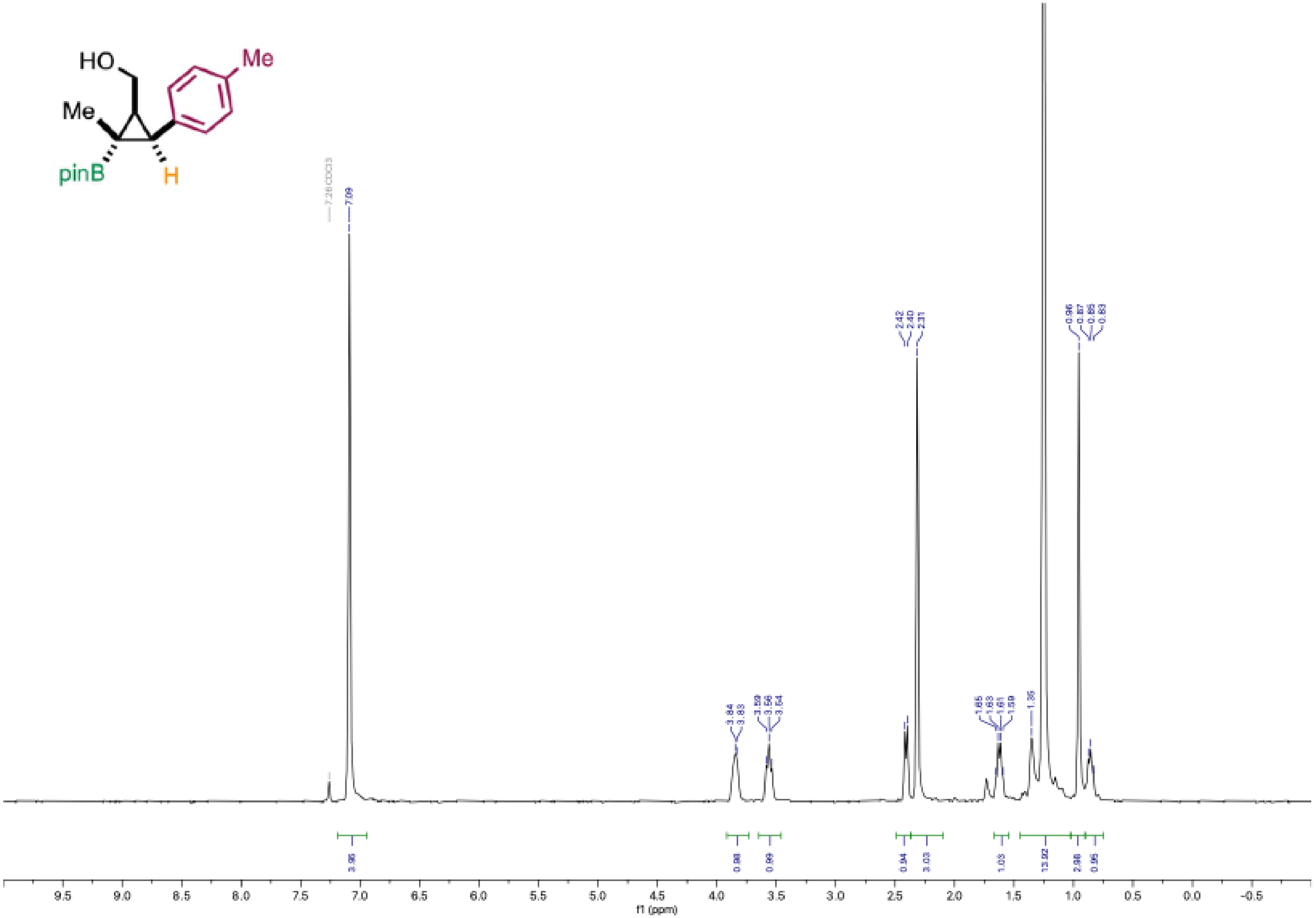

^{1}H NMR spectrum (400 MHz, $CDCl_3$)

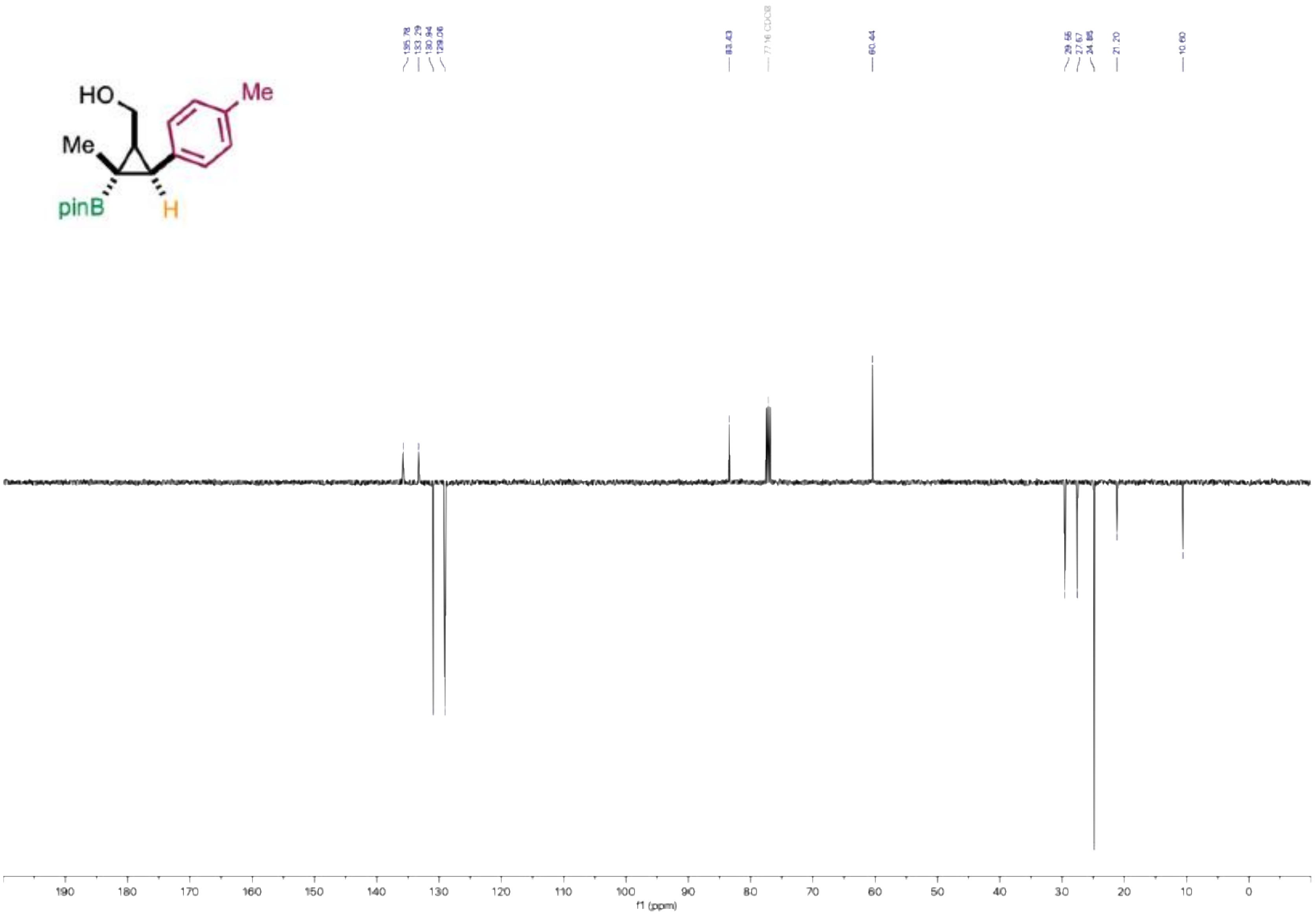

^{13}C NMR spectrum (101 MHz, $CDCl_3$)

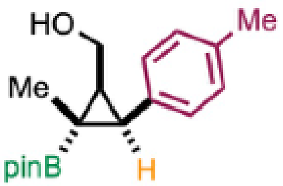

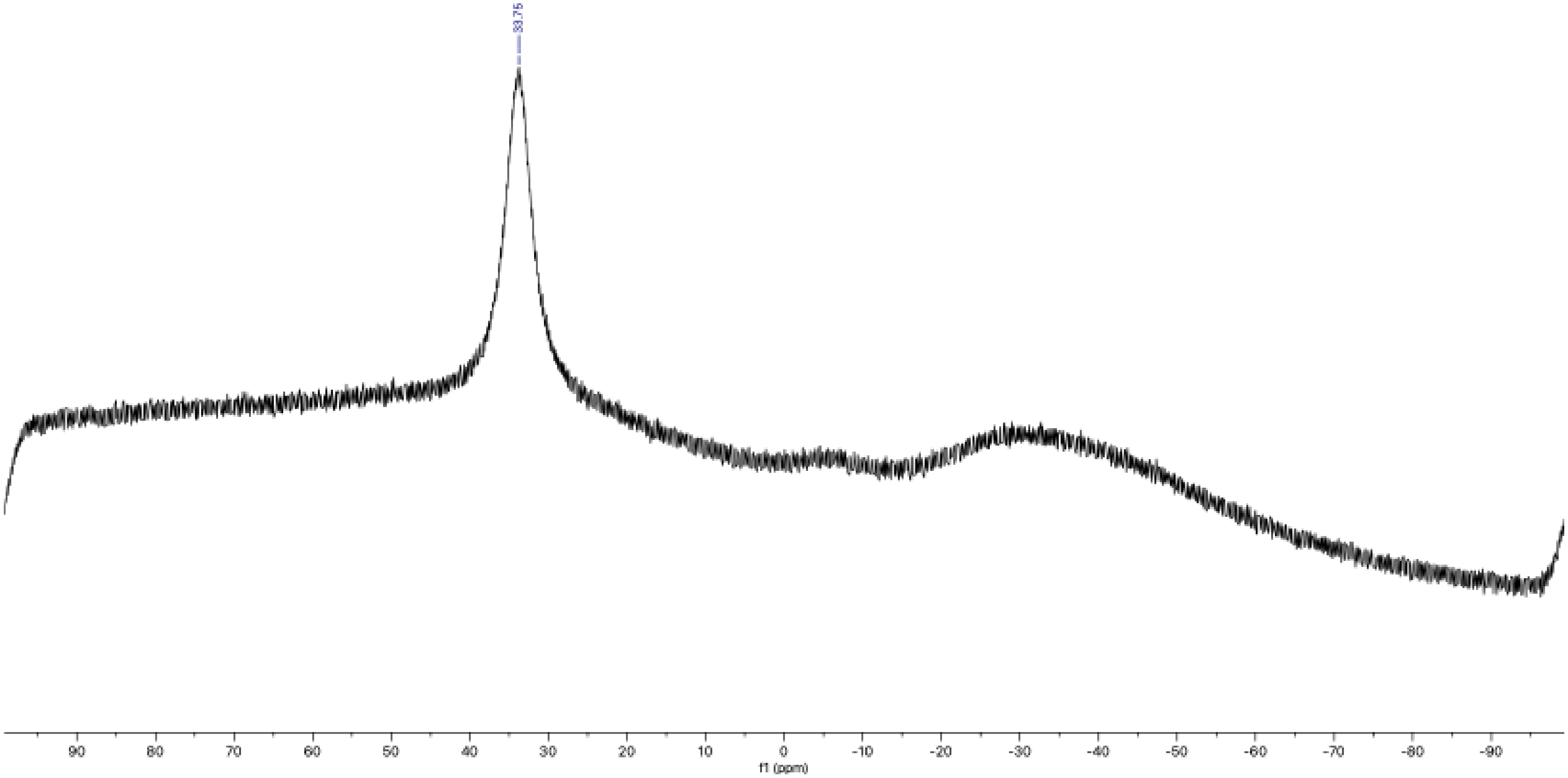

¹¹B NMR spectrum (128 MHz, $CDCl_3$)

((1*S,2*R**,3*R**)-2-Methyl-2-(4,4,5,5-tetramethyl-1,3,2-dioxaborolan-2-yl)-3-(4-(trifluoromethyl)phenyl)cyclopropyl)methanol 2d-OH**

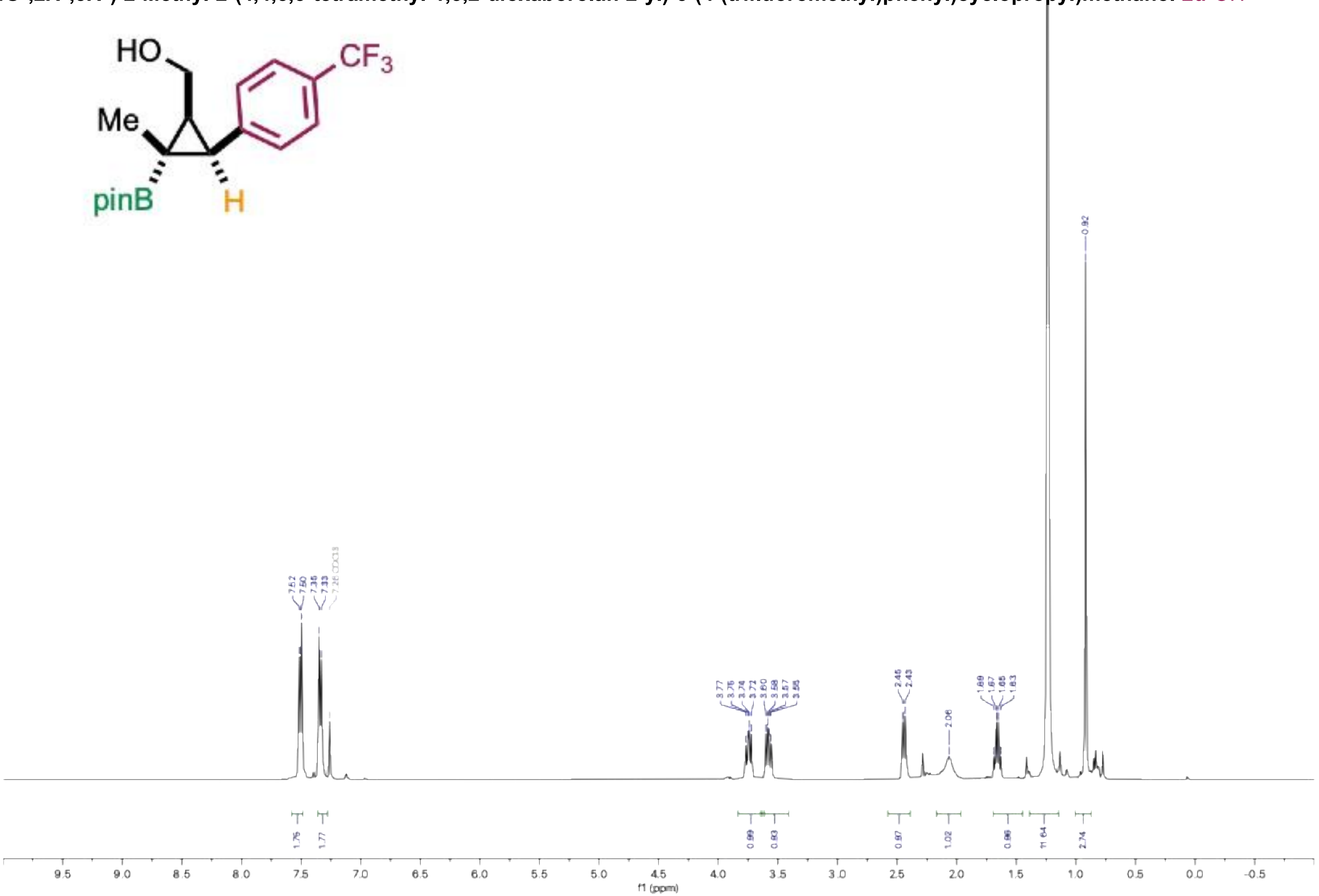

[1]**H NMR** spectrum (400 MHz, $CDCl_3$)

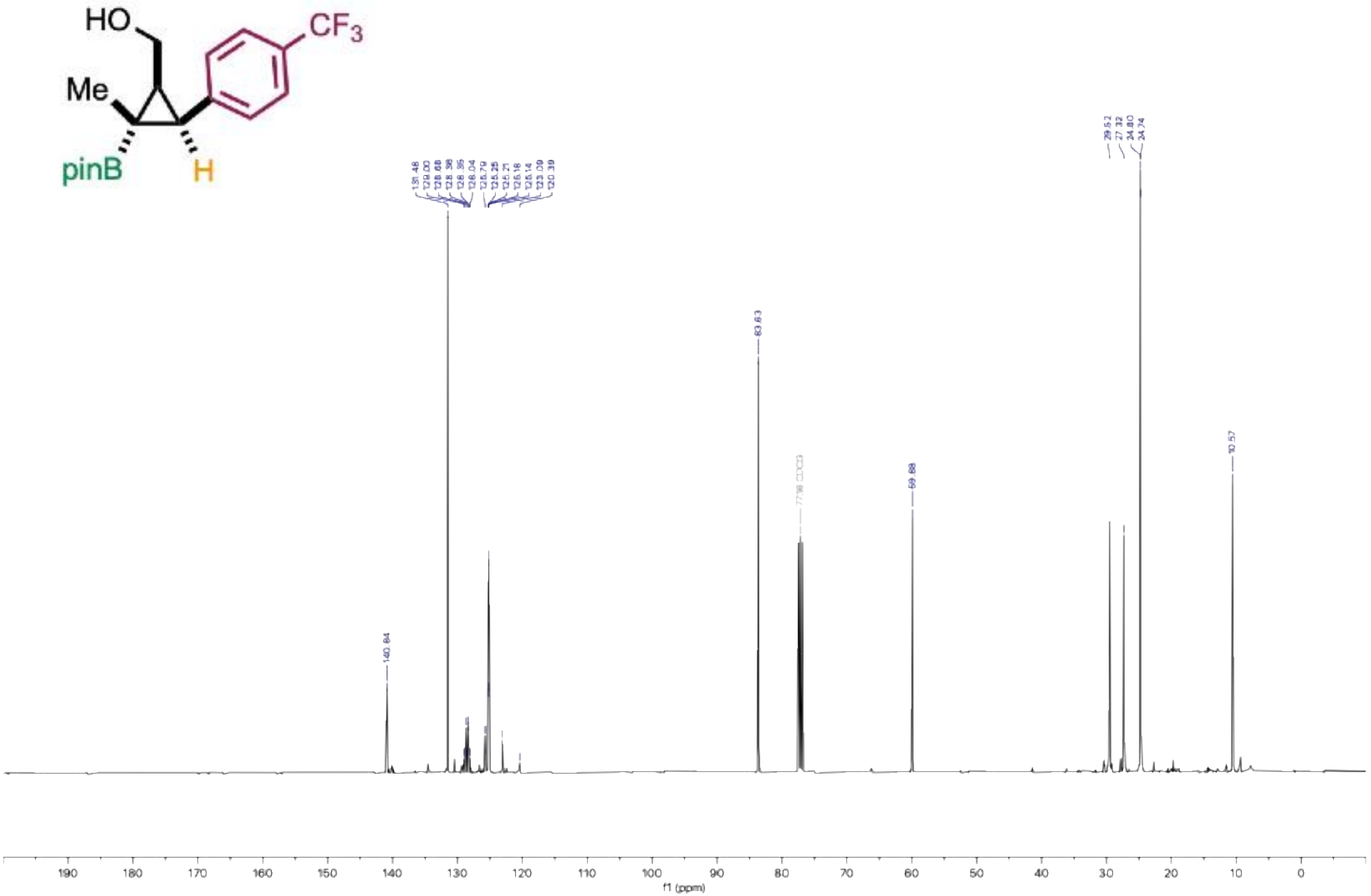

^{13}C NMR spectrum (101 MHz, $CDCl_3$)

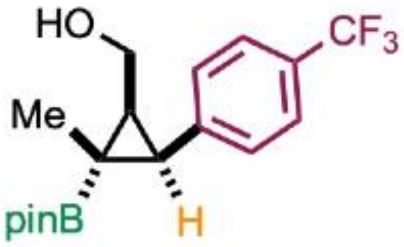

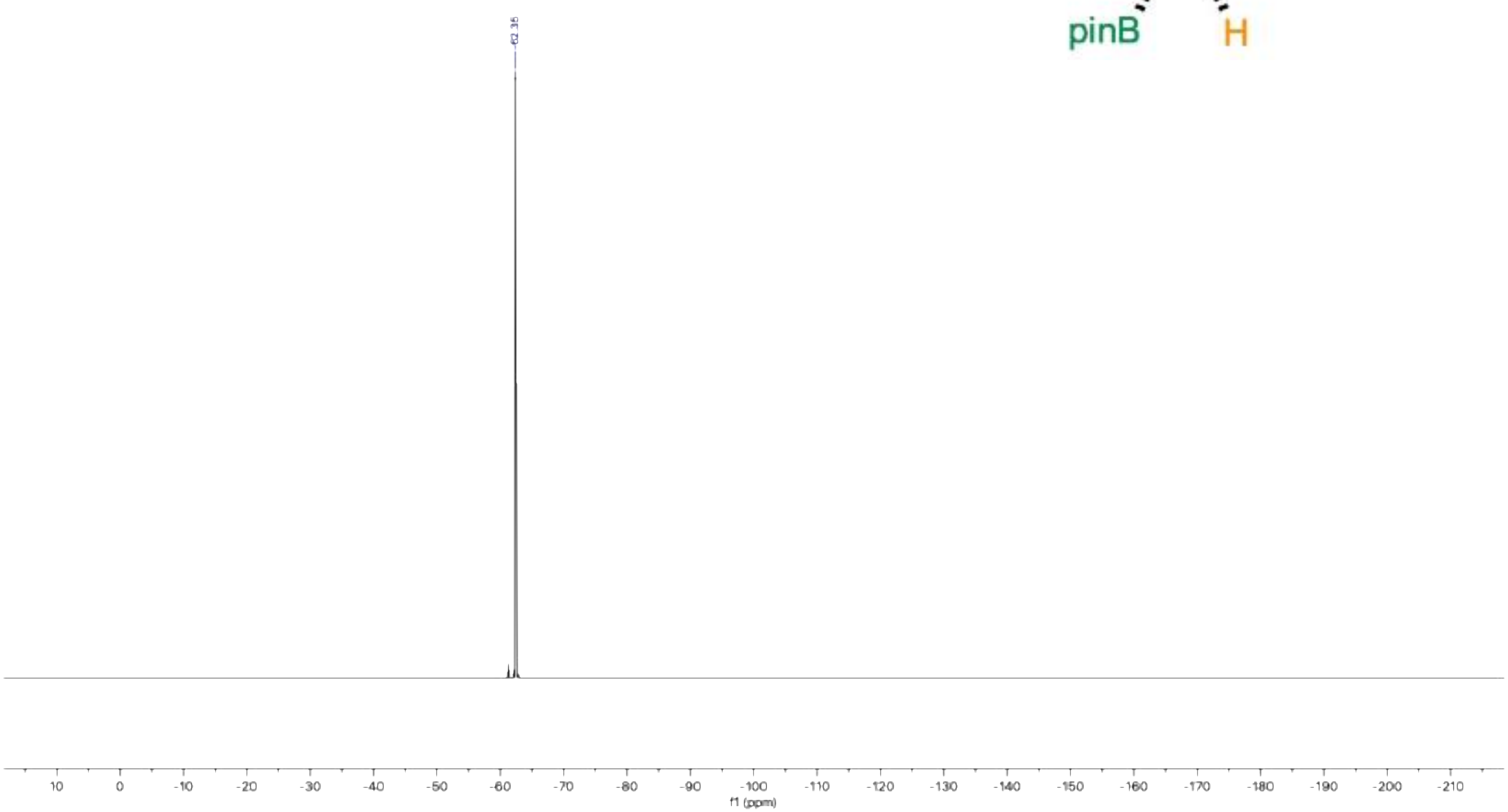

^{19}F NMR spectrum (377 MHz, $CDCl_3$)

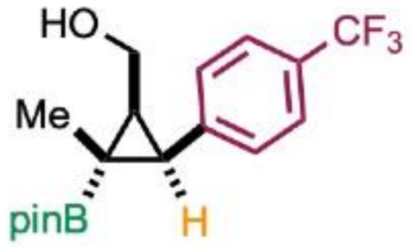

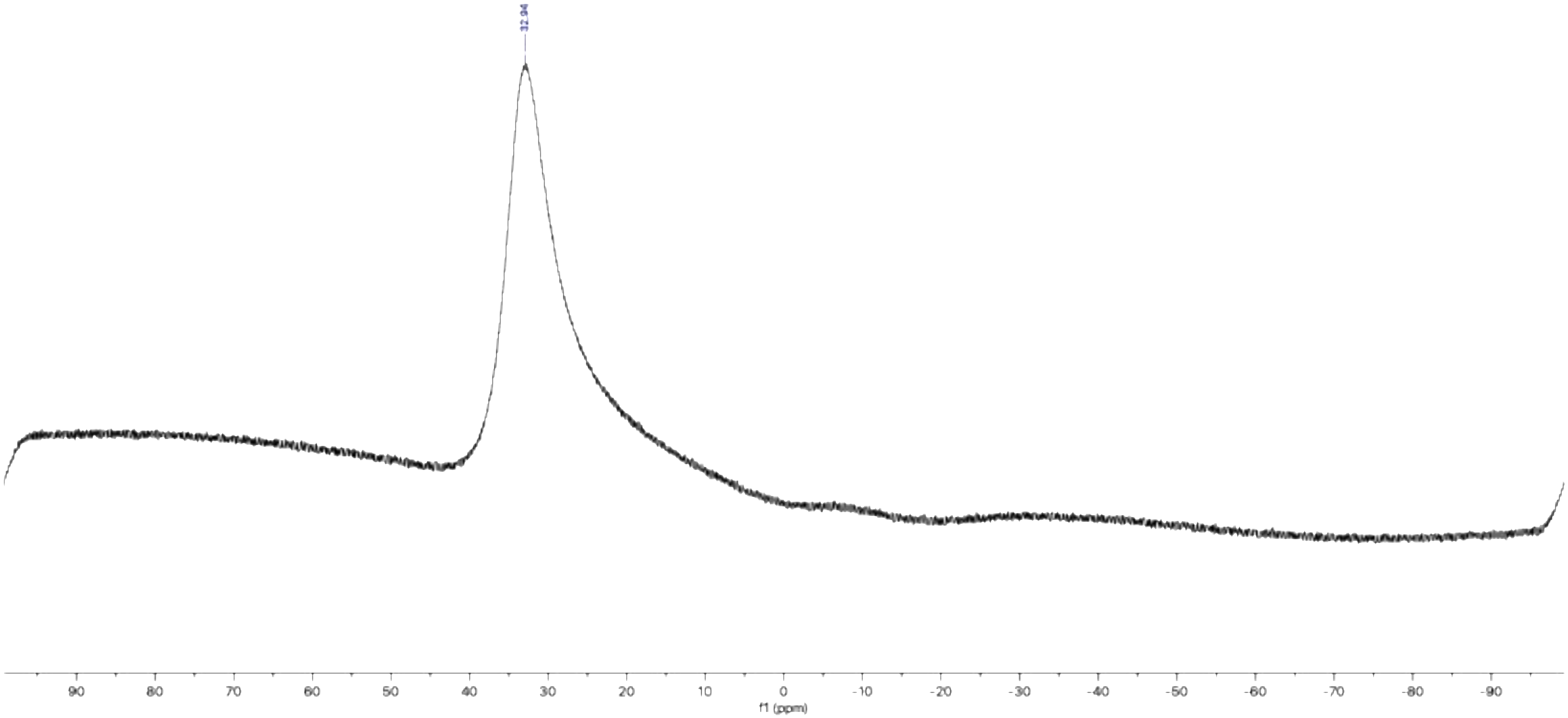

[11]**B NMR** spectrum (128 MHz, $CDCl_3$)

((1*S**,2*R**,3*R**)-3-(3-Chlorophenyl)-2-methyl-2-(4,4,5,5-tetramethyl-1,3,2-dioxaborolan-2-yl)cyclopropyl)methanol **2e**-OH

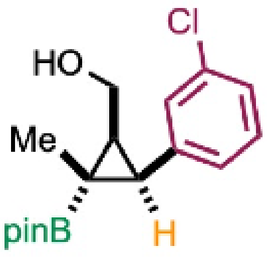

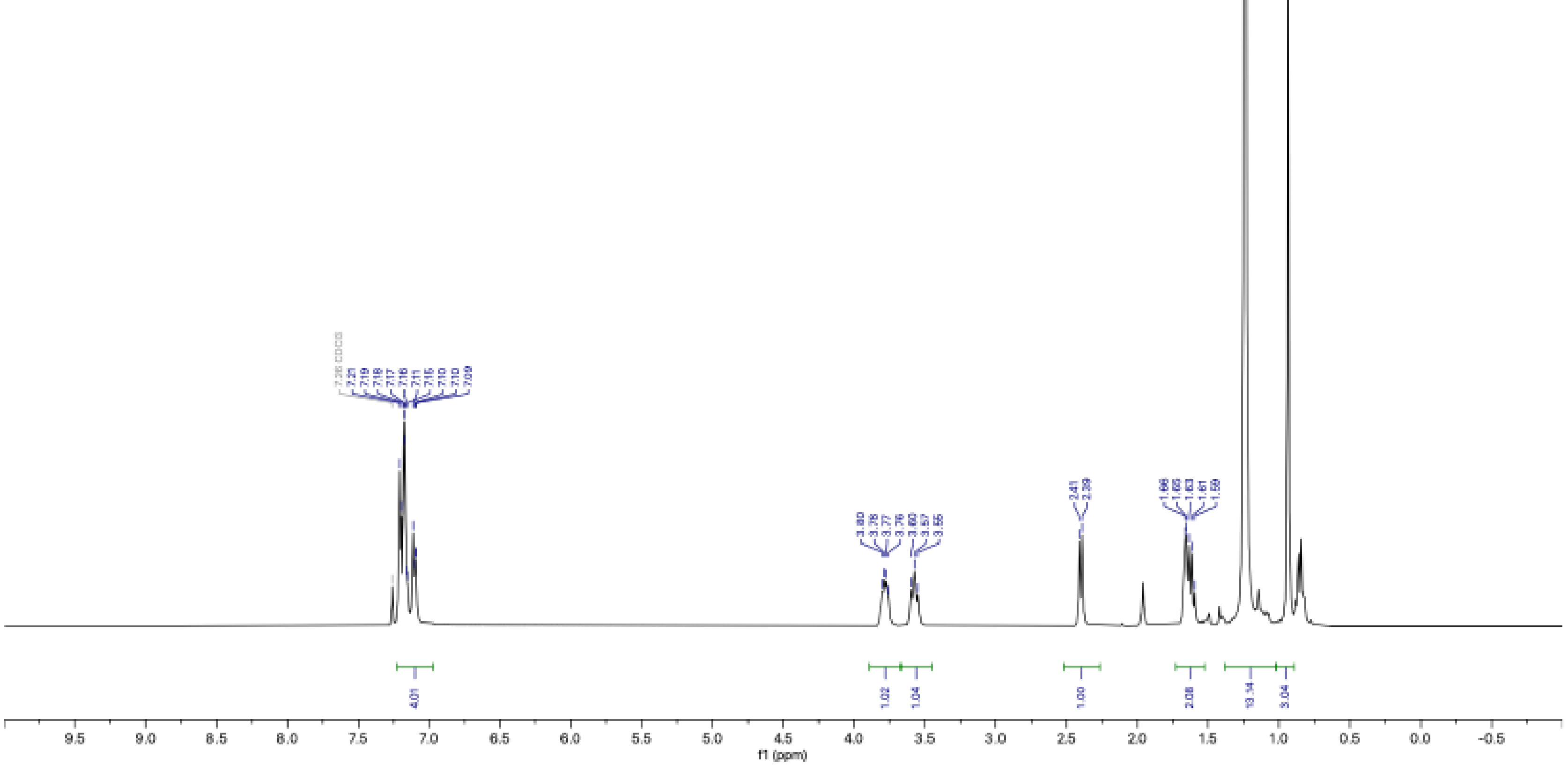

¹H NMR spectrum (400 MHz, $CDCl_3$)

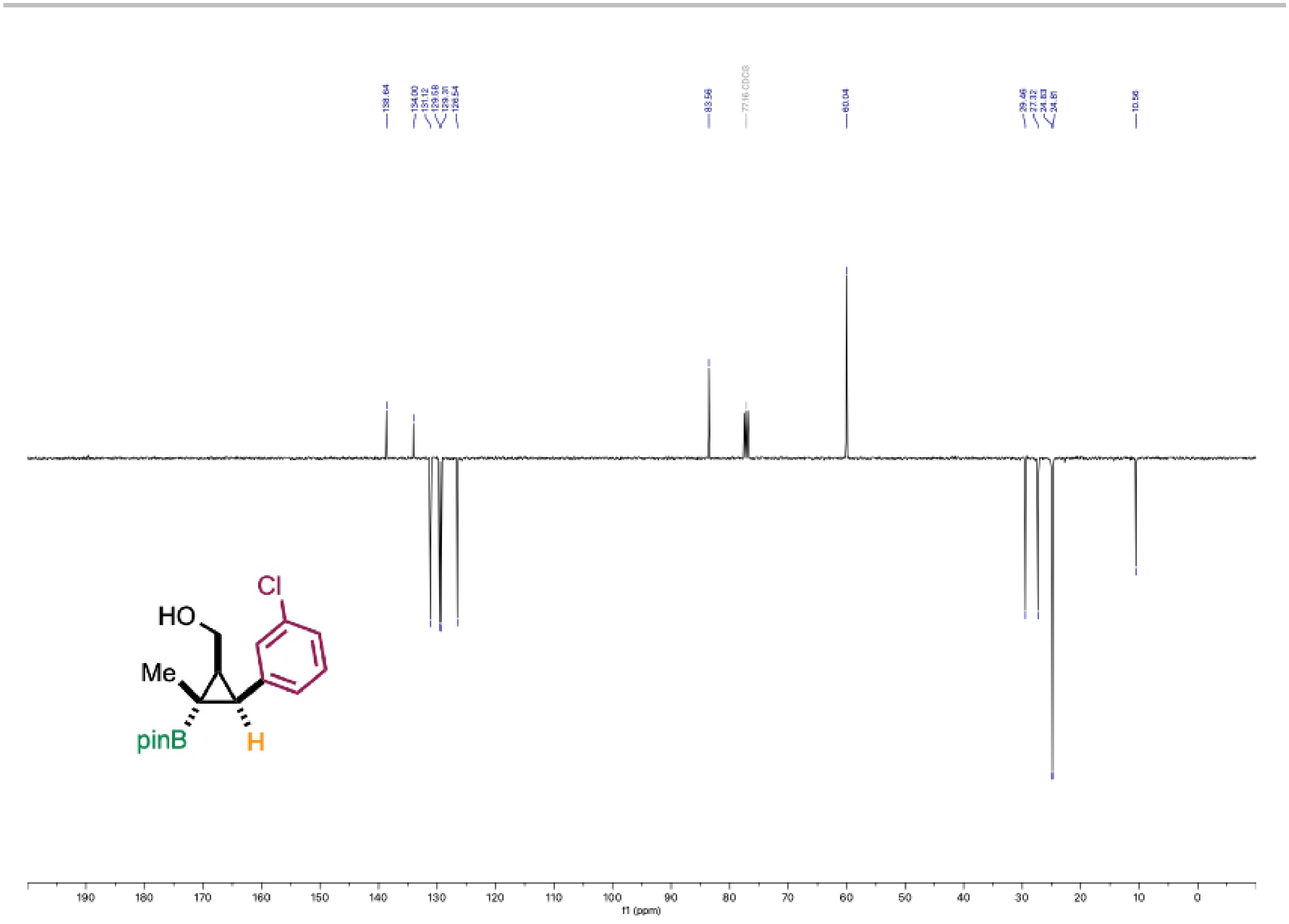

^{13}**C NMR** spectrum (101 MHz, $CDCl_3$)

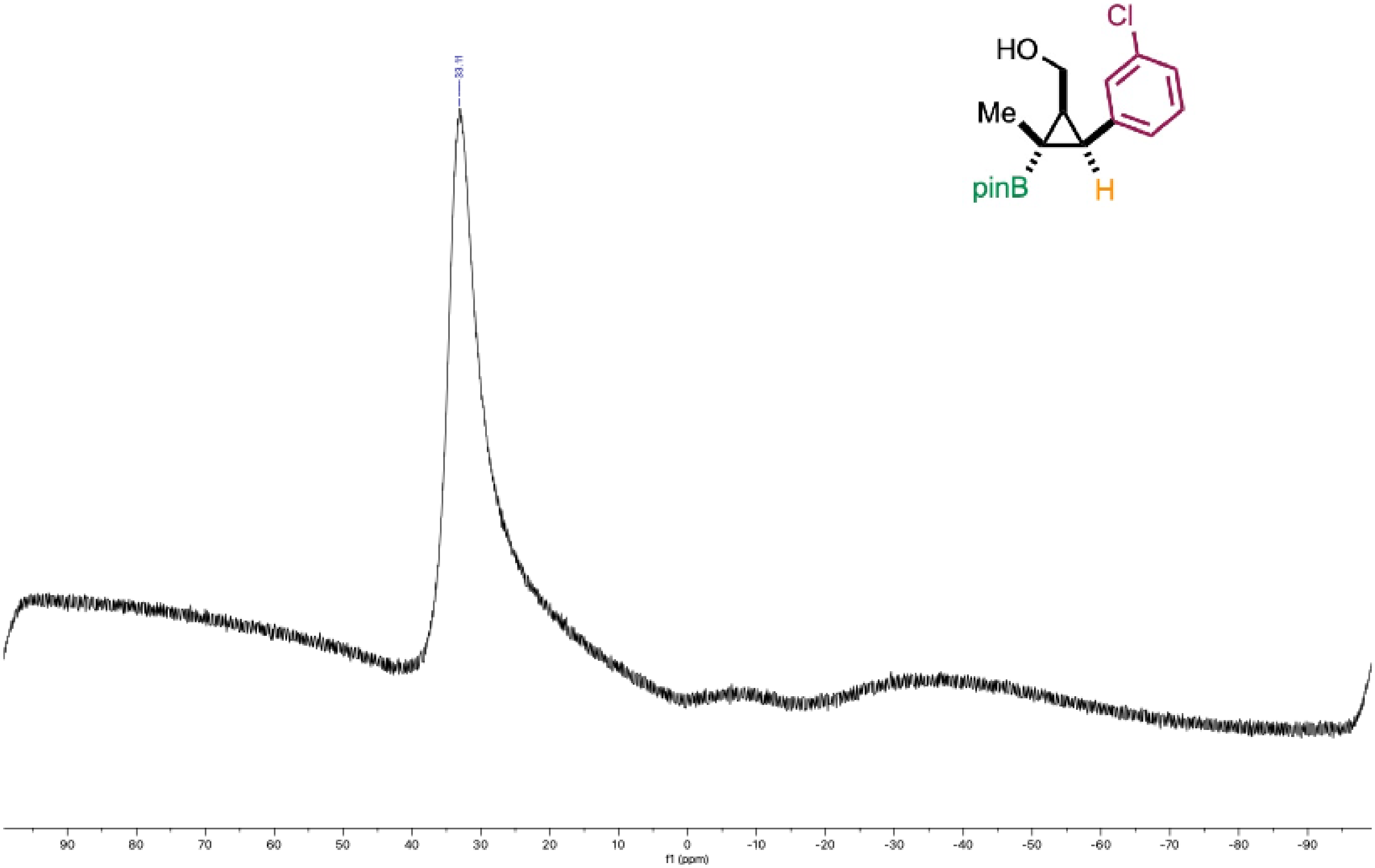

^{11}**B NMR** spectrum (128 MHz, $CDCl_3$)

((1*S,2*R**,3*R**)-2-Methyl-3-(naphthalen-1-yl)-2-(4,4,5,5-tetramethyl-1,3,2-dioxaborolan-2-yl)cyclopropyl)methanol 2f-OH**

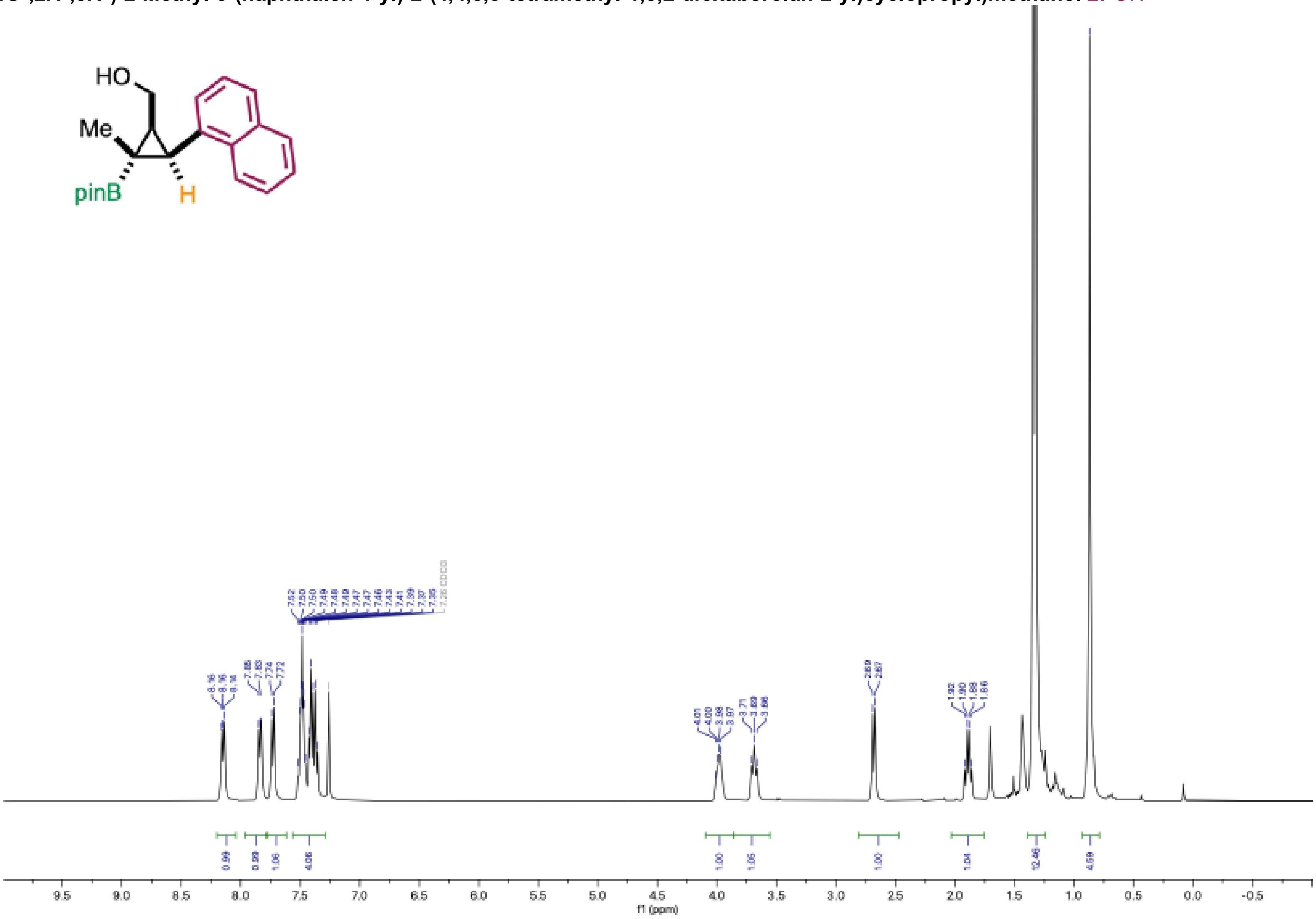

^{1}H NMR spectrum (400 MHz, $CDCl_3$)

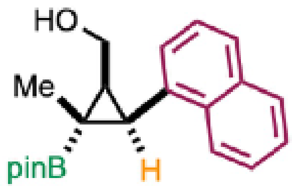

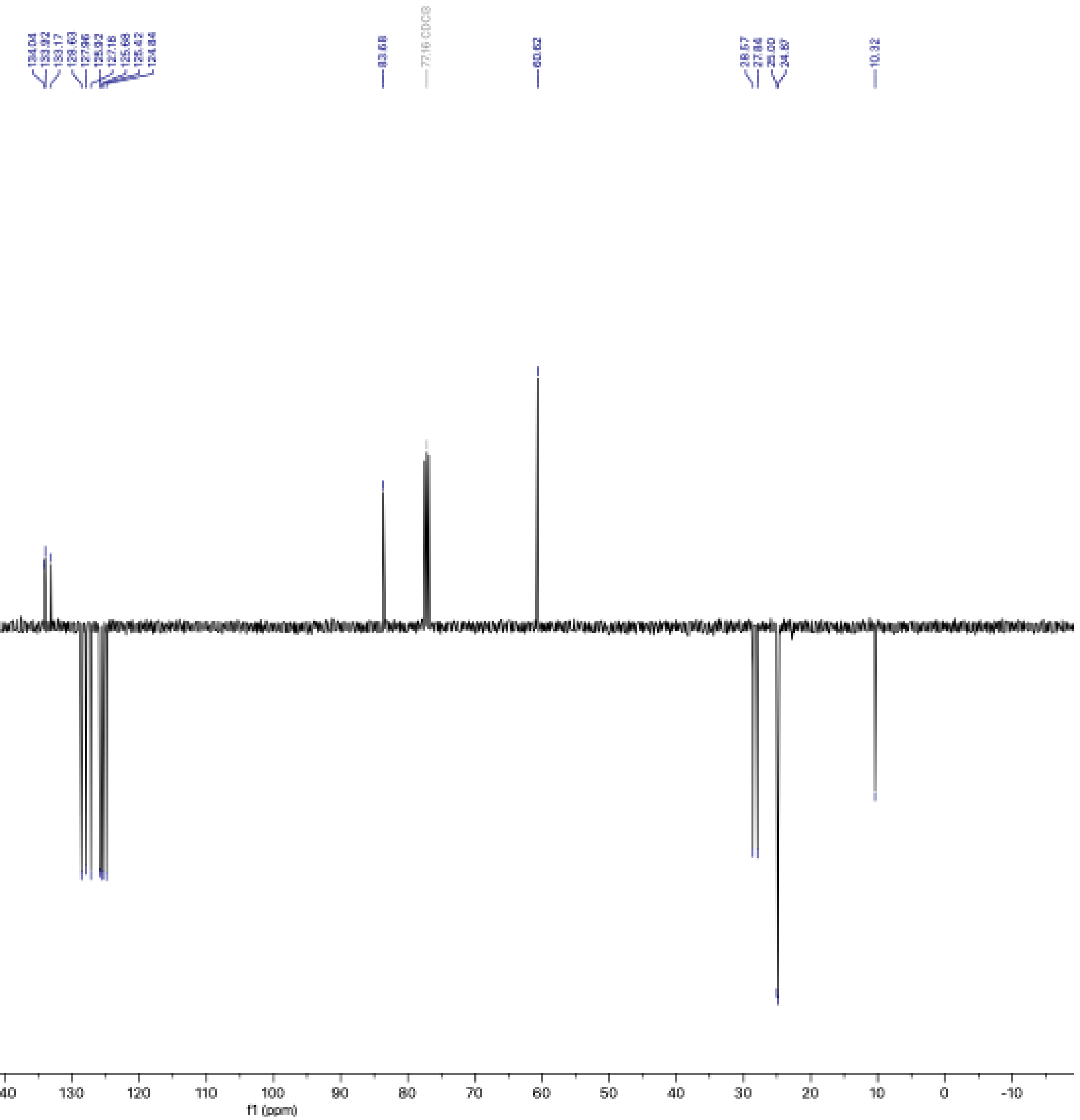

^{13}C NMR spectrum (101 MHz, $CDCl_3$)

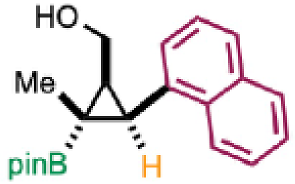

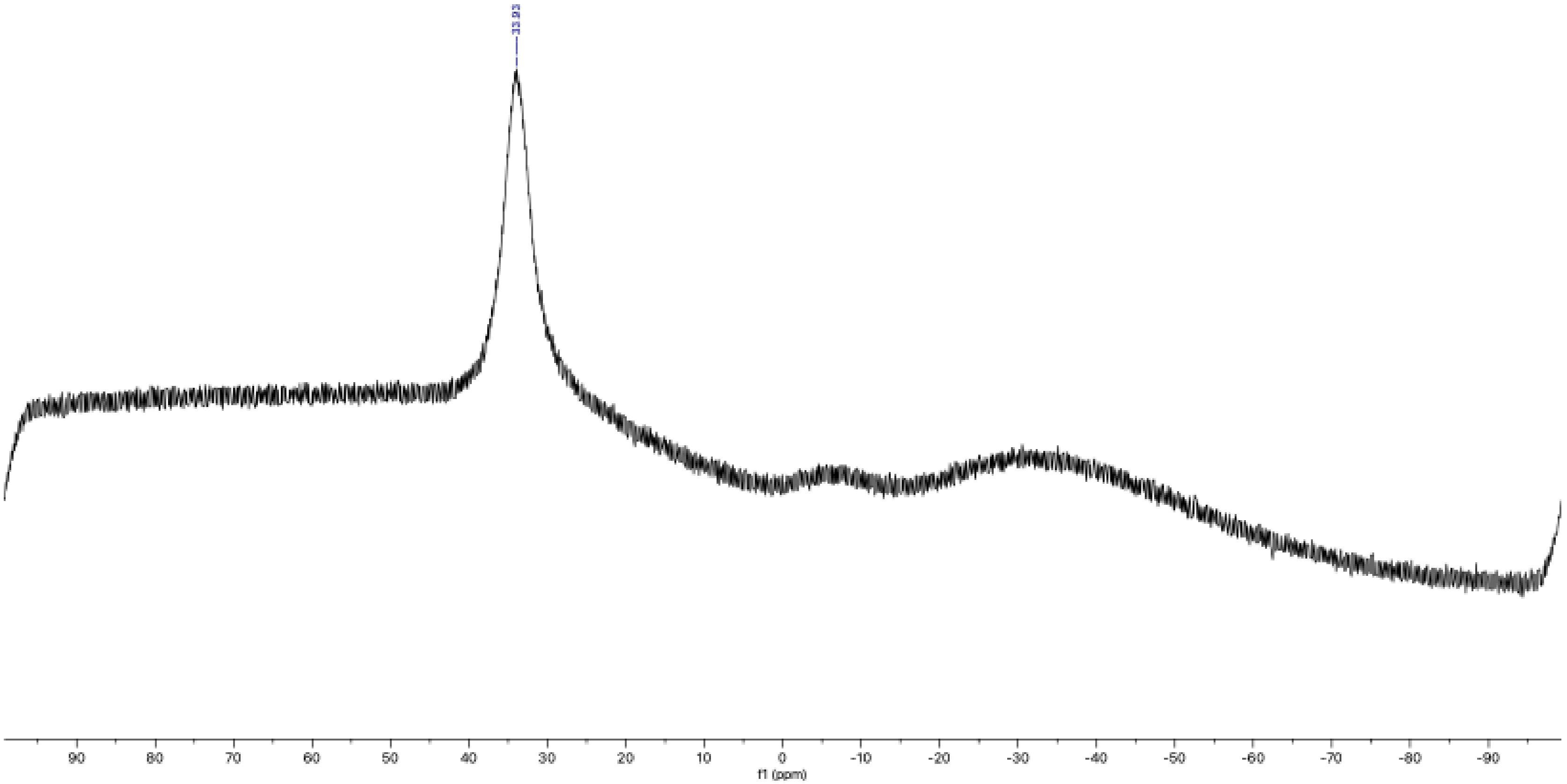

^{11}B NMR spectrum (128 MHz, $CDCl_3$)

((1*S,2*R**,3*R**)-2-Ethyl-3-(perfluorophenyl)-2-(4,4,5,5-tetramethyl-1,3,2-dioxaborolan-2-yl)cyclopropyl)methanol 2g-OH**

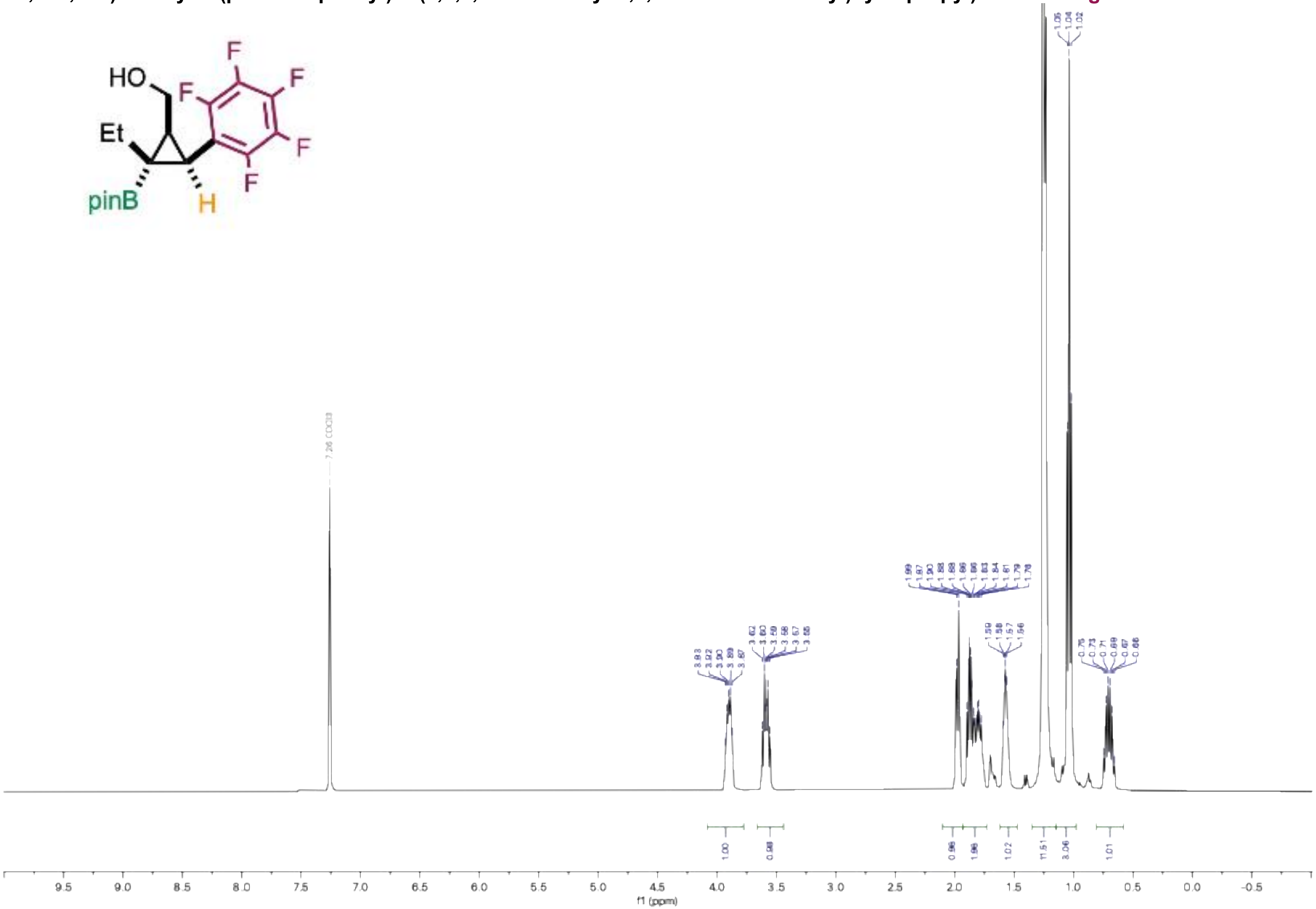

^{1}H NMR spectrum (400 MHz, $CDCl_3$)

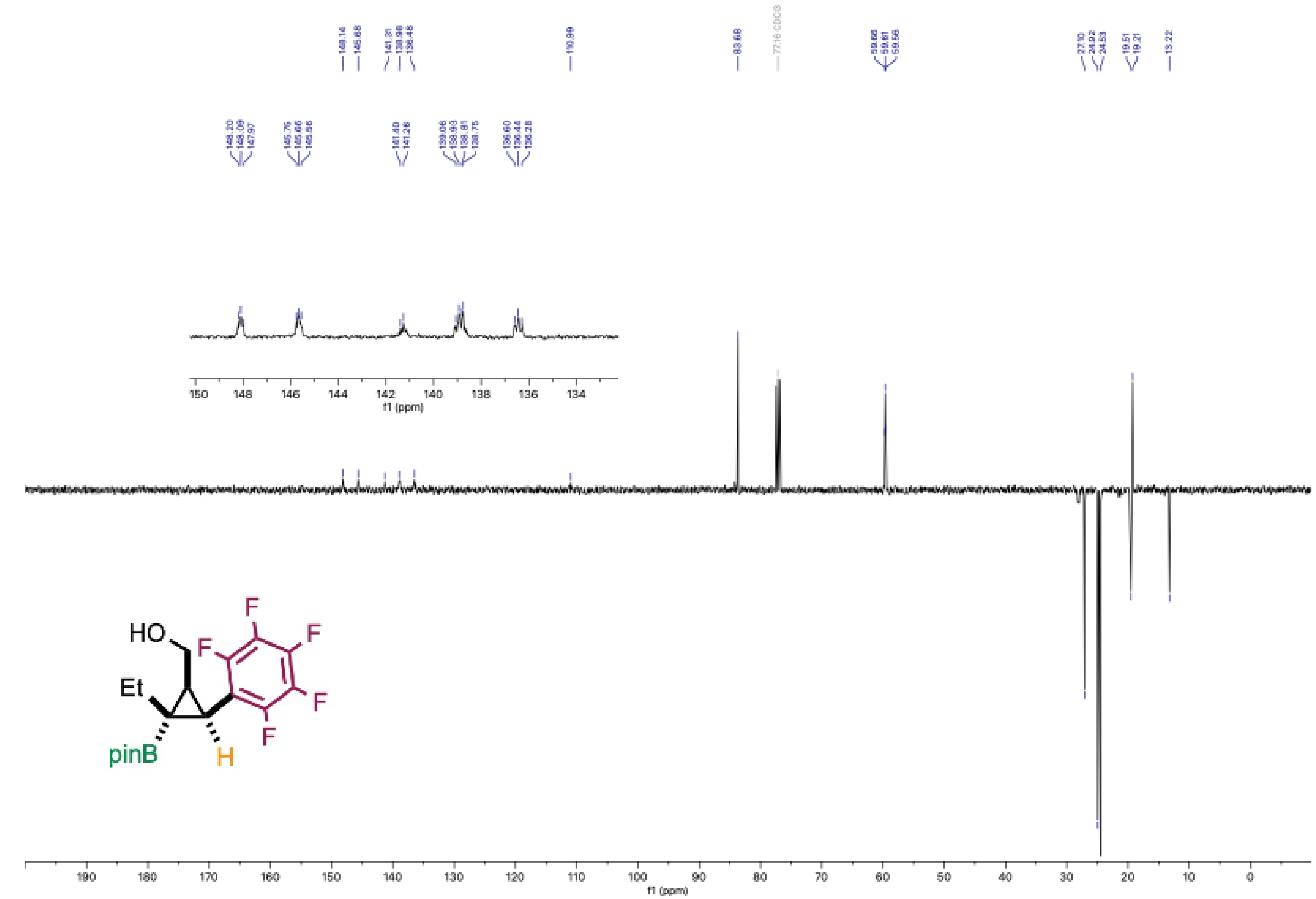

^{13}C NMR spectrum (101 MHz, $CDCl_3$)

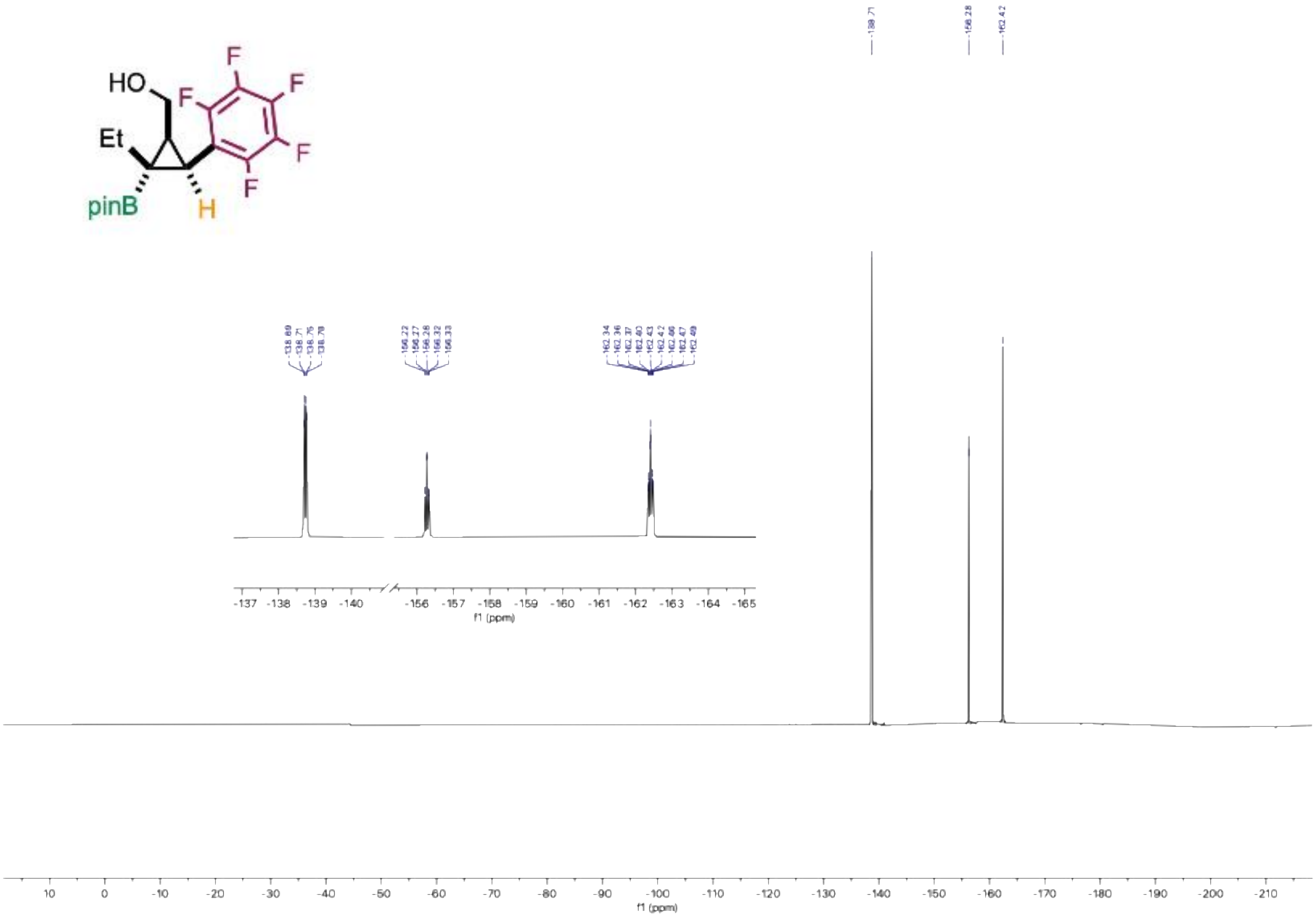

^{19}F NMR spectrum (377 MHz, $CDCl_3$)

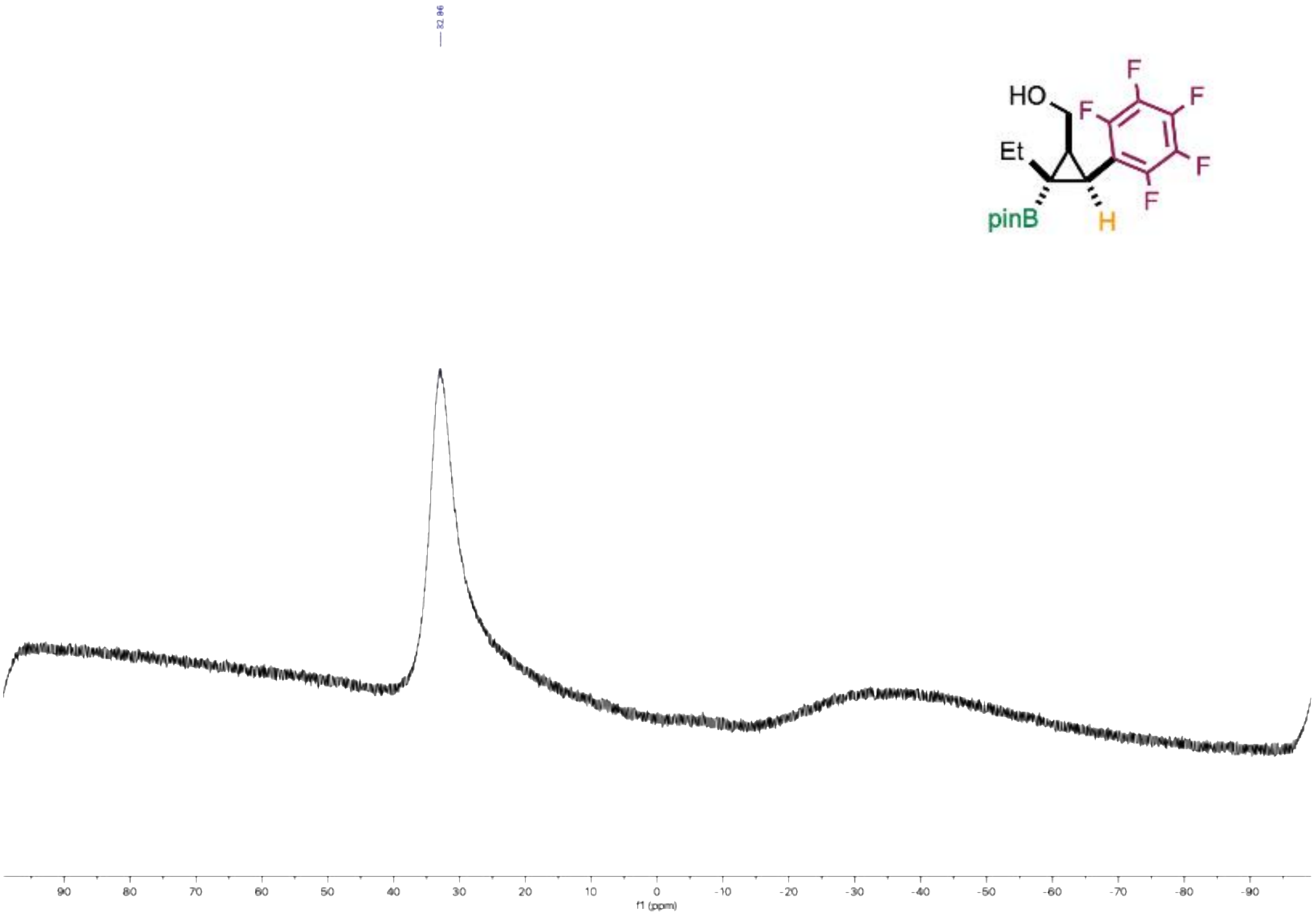

^{11}**B NMR** spectrum (128 MHz, $CDCl_3$)

((1*S,2*R**,3*R**)-2-Butyl-3-(4-chlorophenyl)-2-(4,4,5,5-tetramethyl-1,3,2-dioxaborolan-2-yl)cyclopropyl)methanol 2i**-OH

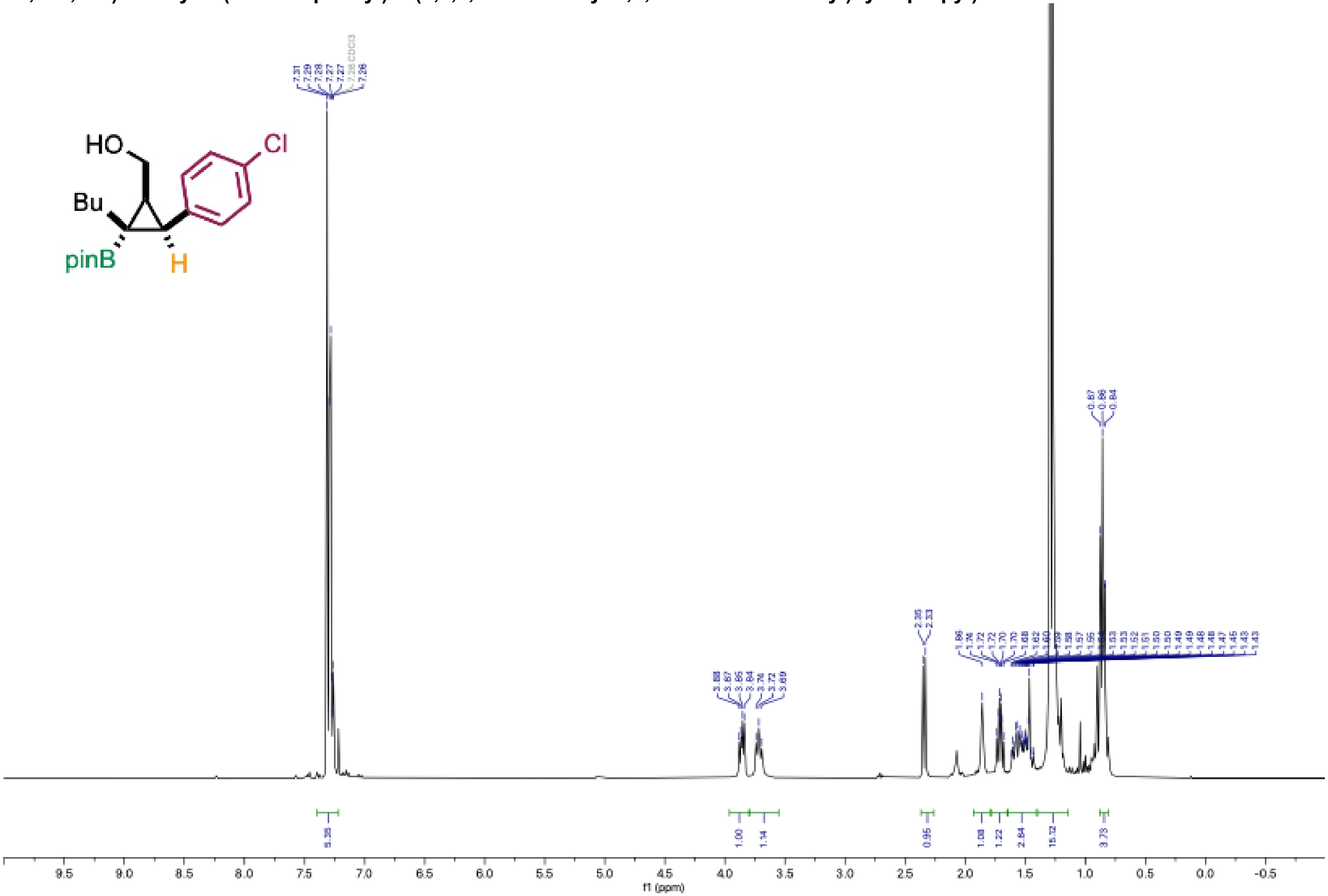

[1]**H NMR** spectrum (400 MHz, $CDCl_3$)

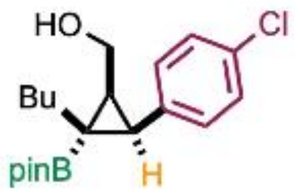

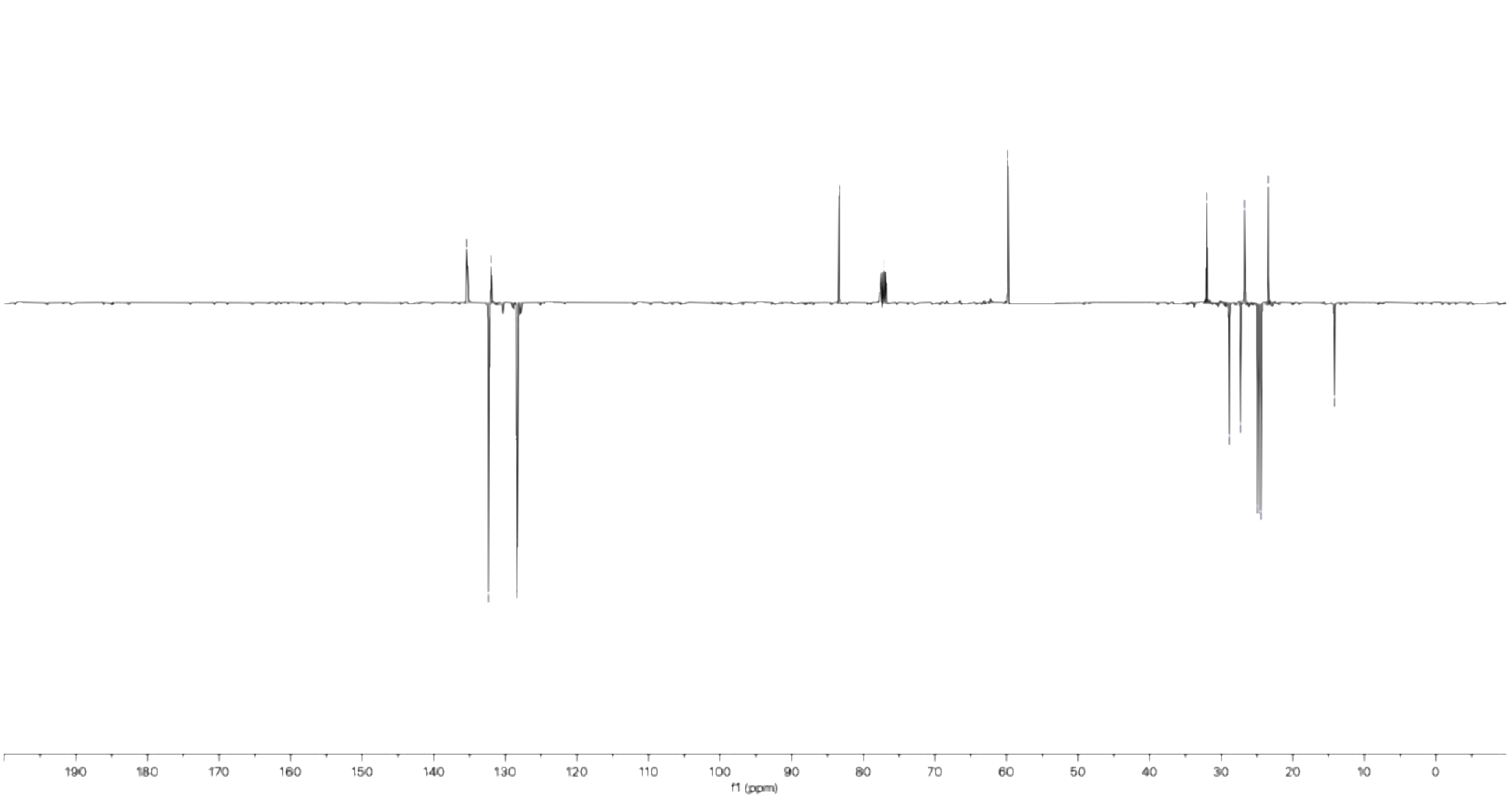

^{13}C NMR spectrum (101 MHz, $CDCl_3$)

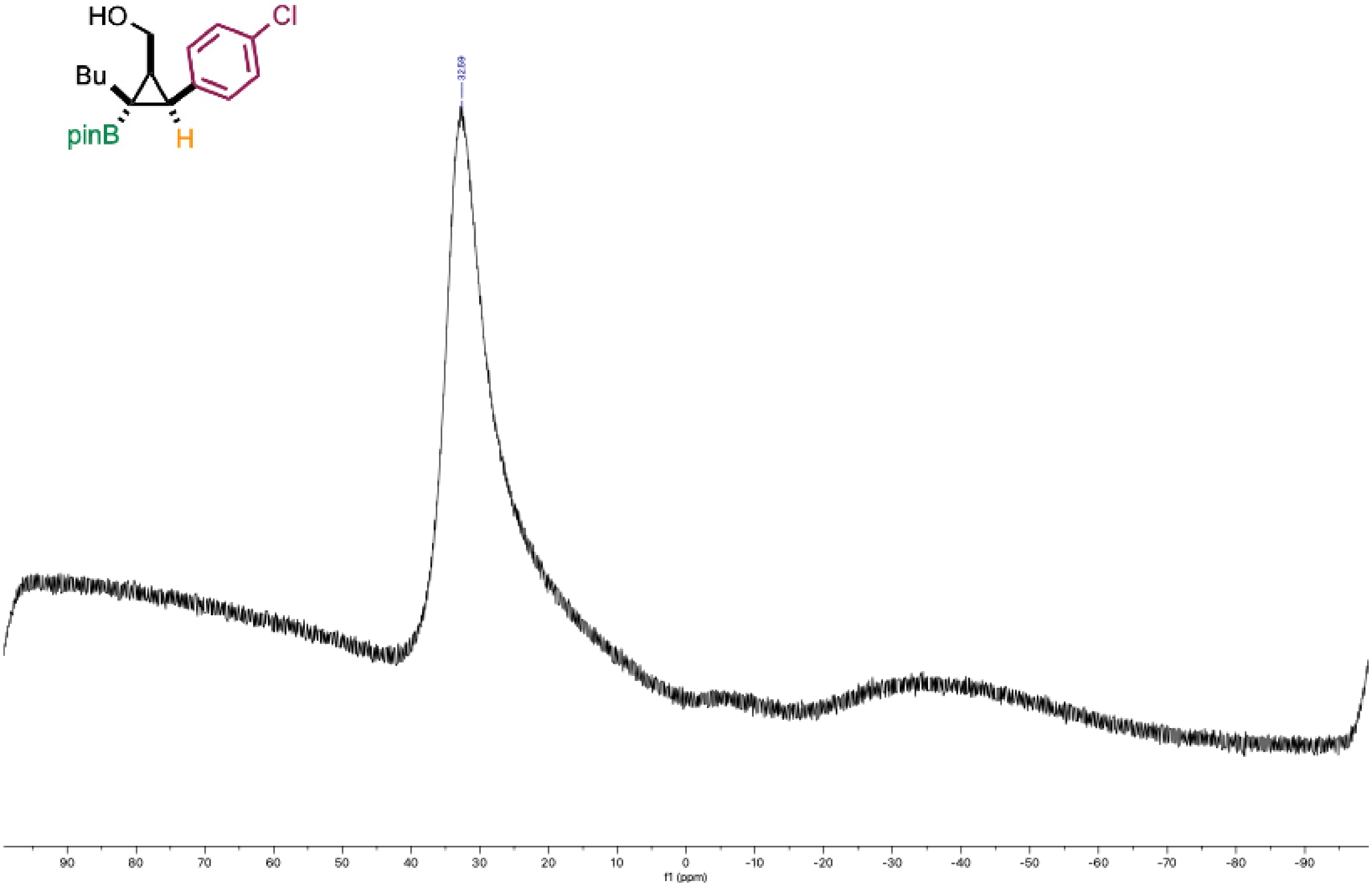

^{11}B NMR spectrum (128 MHz, $CDCl_3$)

((1*S,2*R**,3*R**)-2-Butyl-3-(4-methoxyphenyl)-2-(4,4,5,5-tetramethyl-1,3,2-dioxaborolan-2-yl)cyclopropyl)methanol 2j-OH**

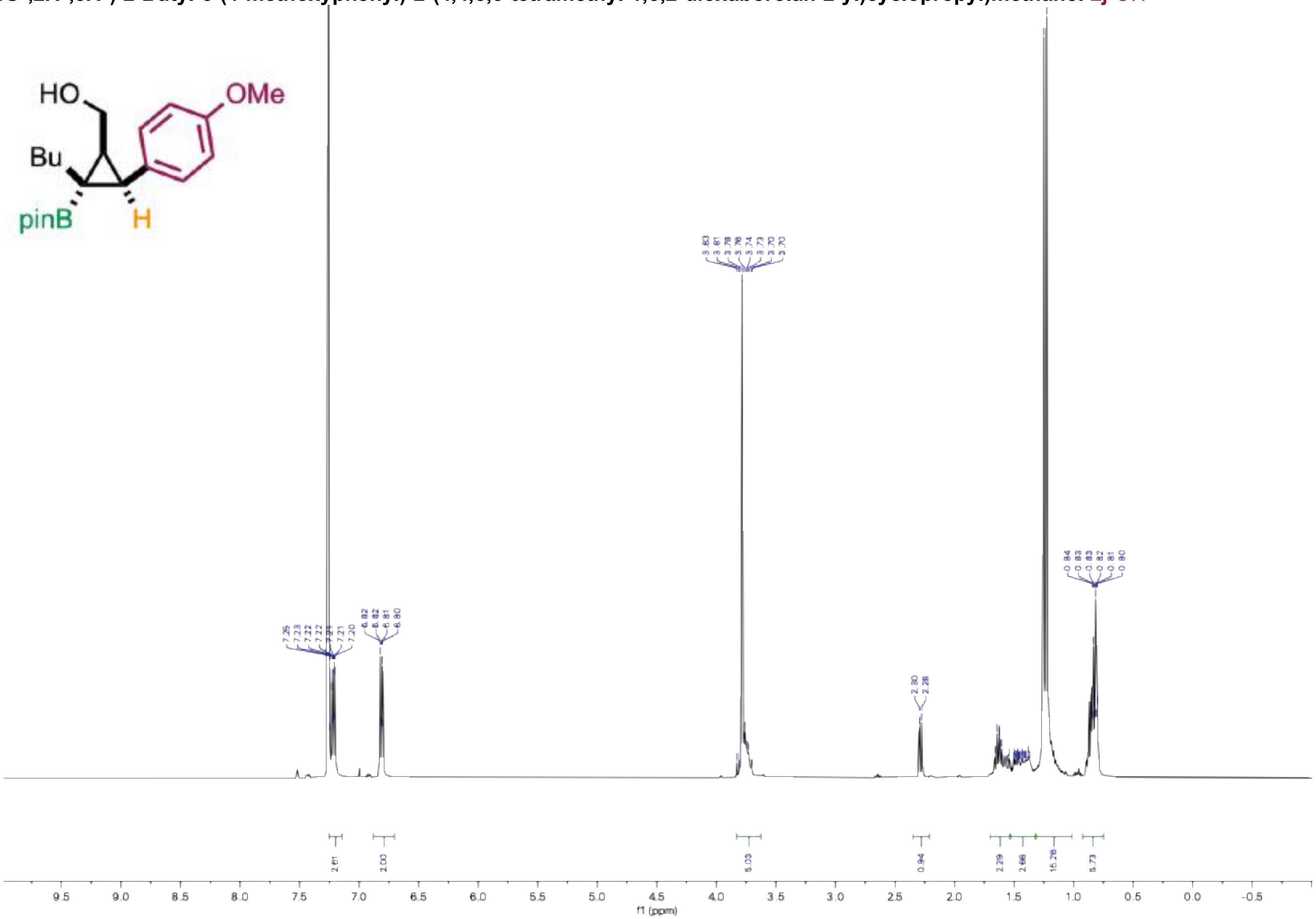

^{1}H NMR spectrum (400 MHz, $CDCl_3$)

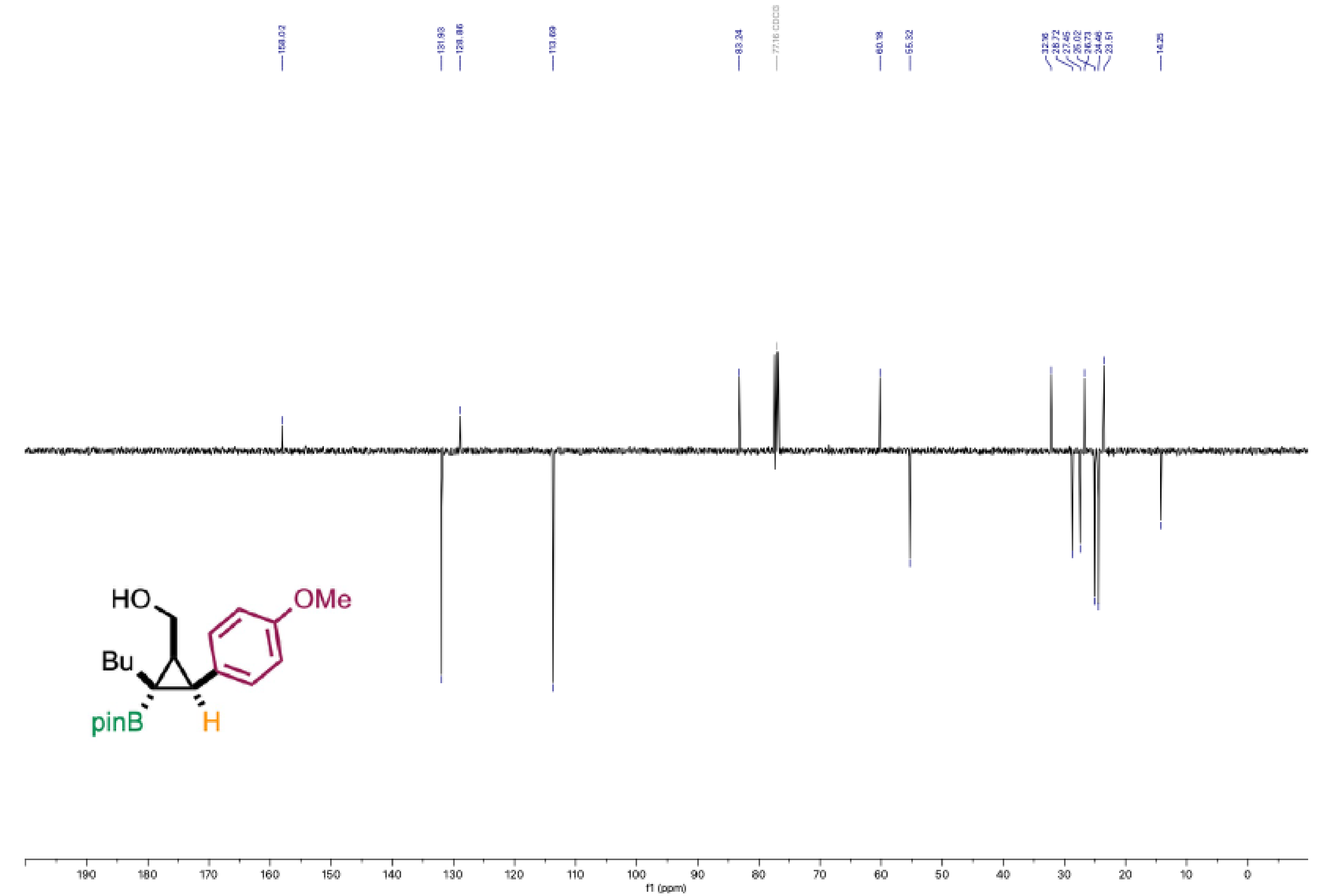

^{13}**C NMR** spectrum (101 MHz, $CDCl_3$)

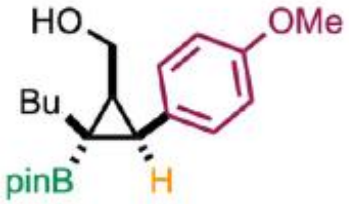

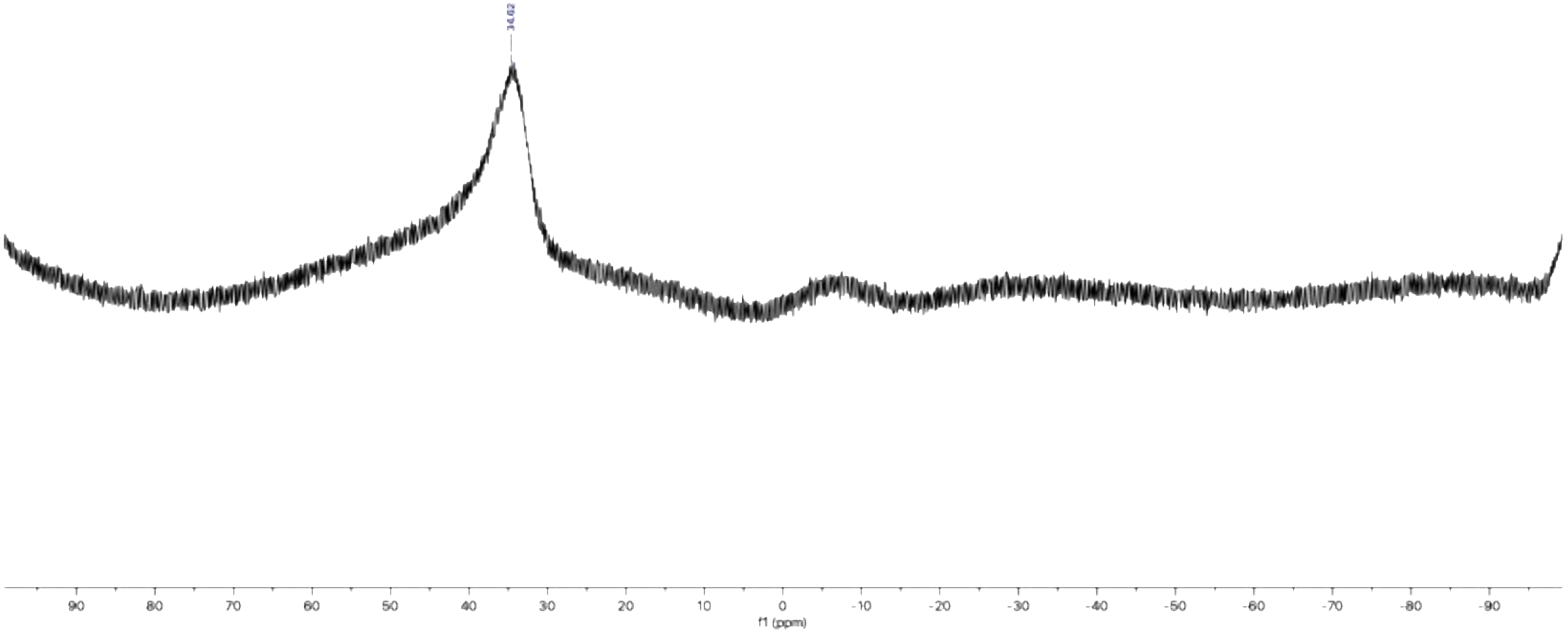

^{11}B NMR spectrum (128 MHz, $CDCl_3$)

((1*S,2*R**,3*R**)-2-Cyclohexyl-3-phenyl-2-(4,4,5,5-tetramethyl-1,3,2-dioxaborolan-2-yl)cyclopropyl)methanol 2k-OH**

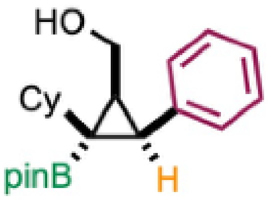

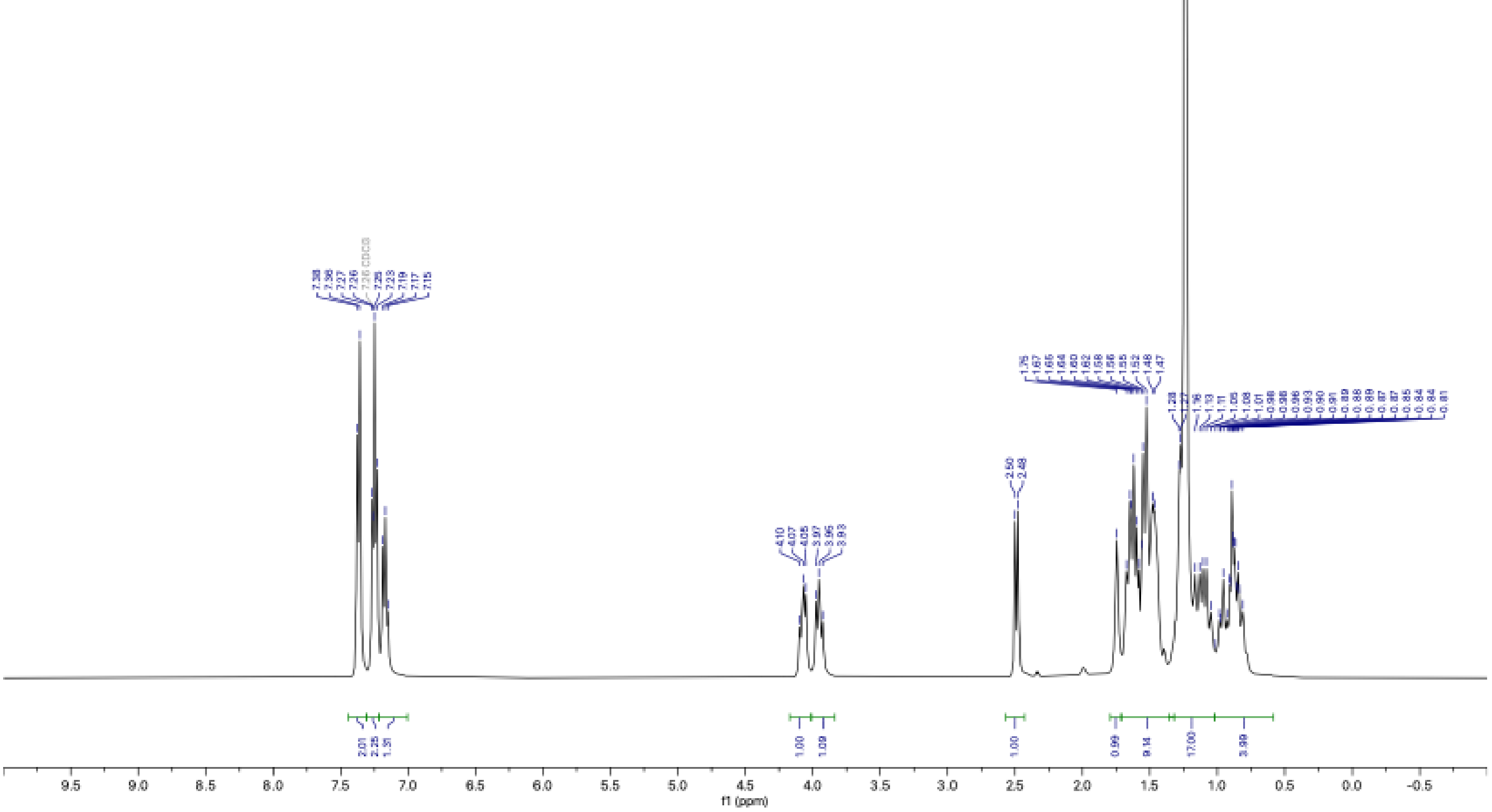

^{1}H NMR spectrum (400 MHz, $CDCl_3$)

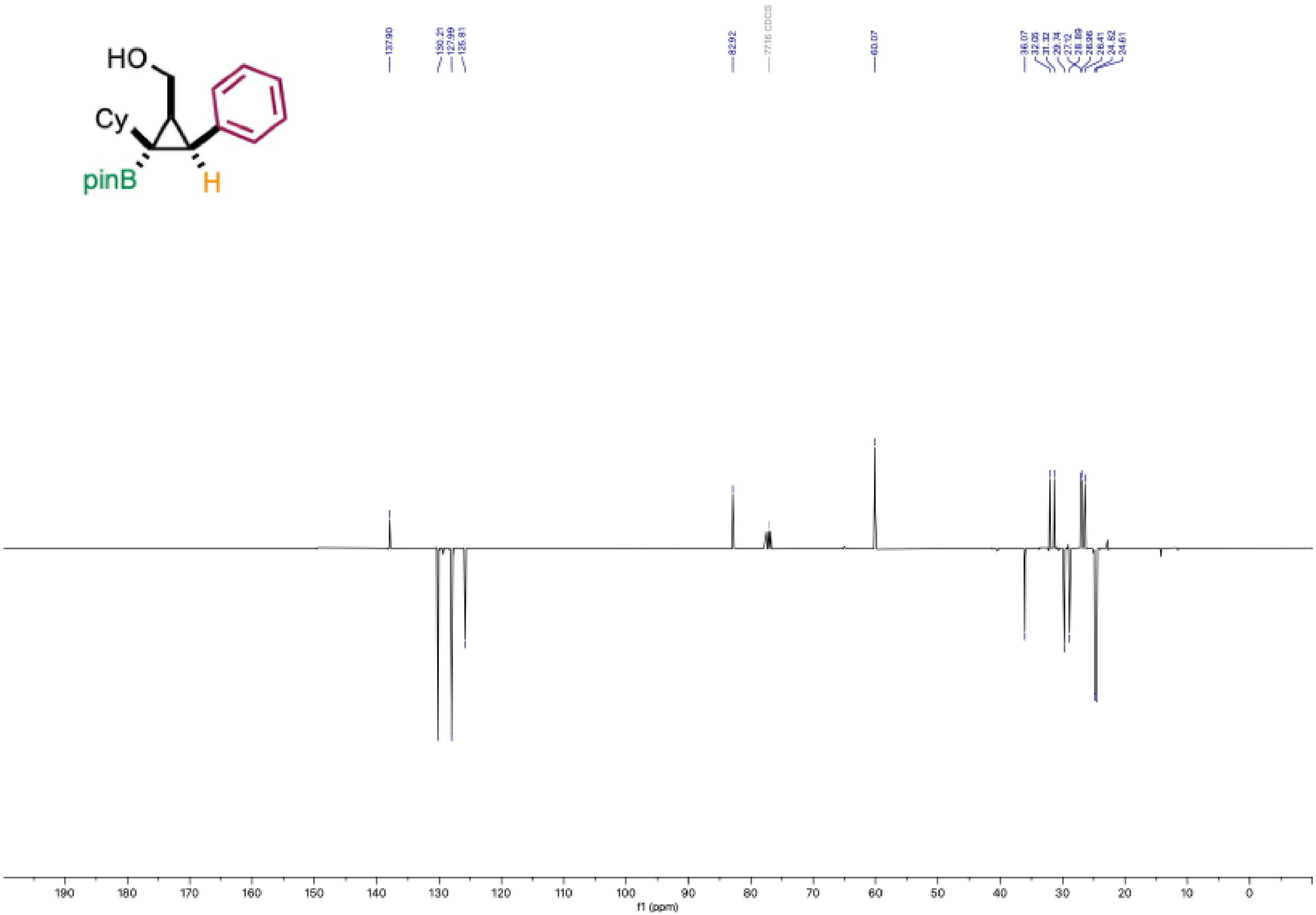

^{13}**C NMR** spectrum (101 MHz, $CDCl_3$)

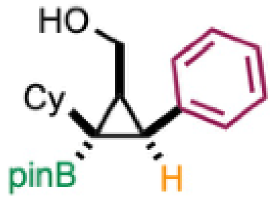

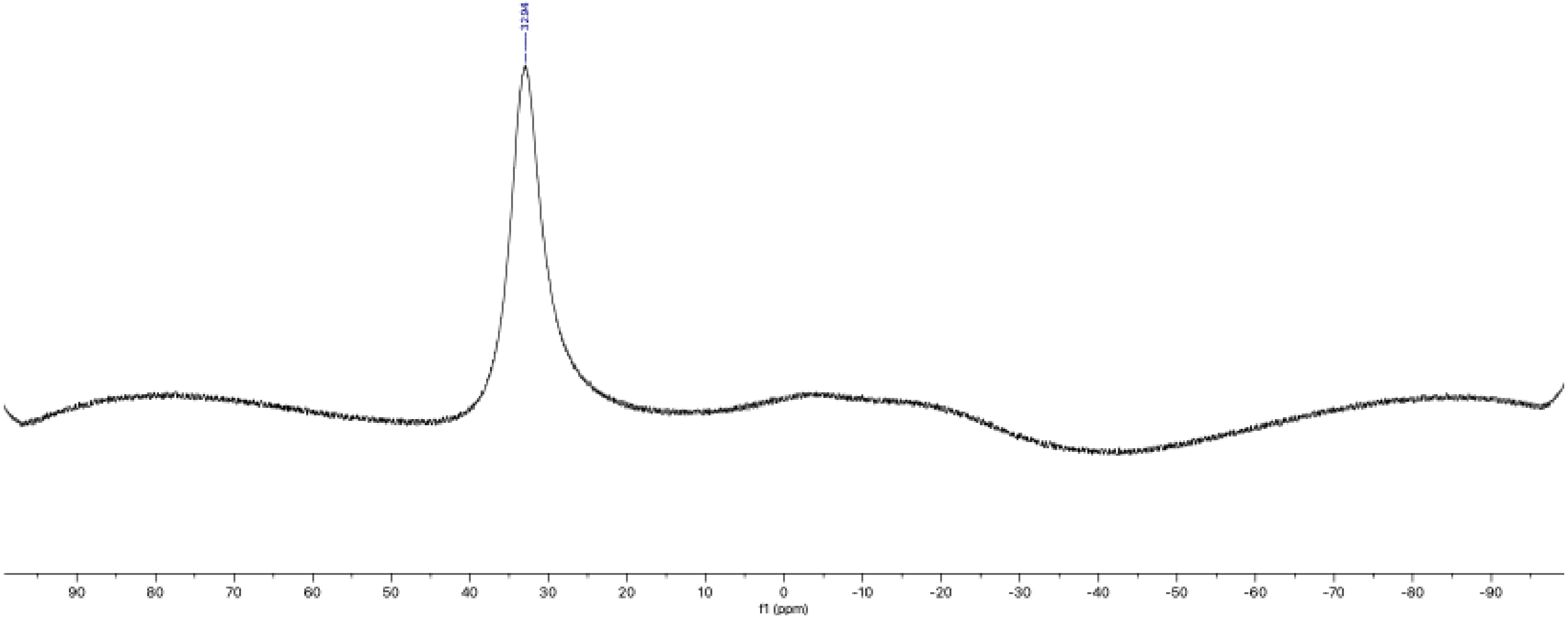

^{11}B NMR spectrum (128 MHz, $CDCl_3$)

((1*S,2*R**,3*R**)-2-(3-Chloropropyl)-3-phenyl-2-(4,4,5,5-tetramethyl-1,3,2-dioxaborolan-2-yl)cyclopropyl)methanol 2l-OH**

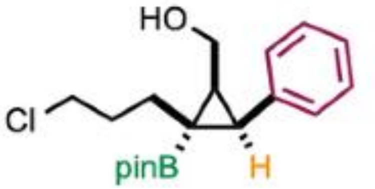

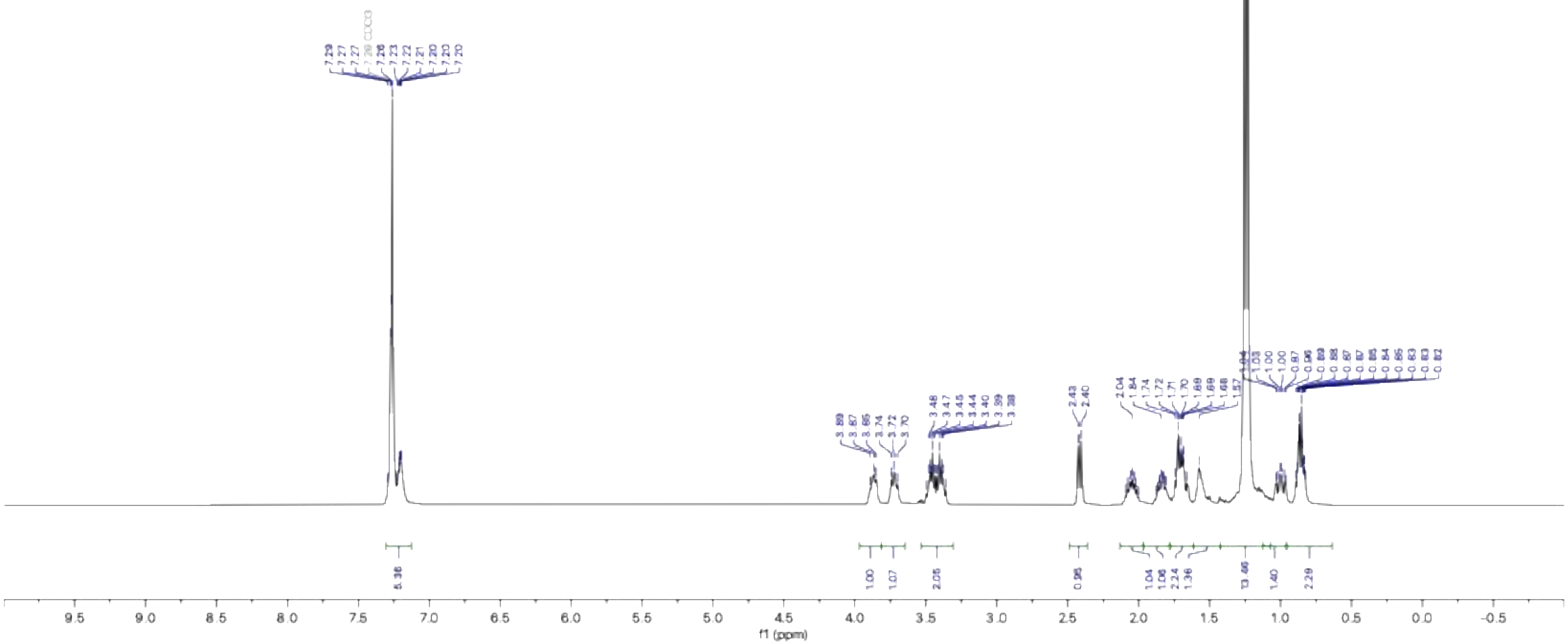

^{1}H NMR spectrum (400 MHz, $CDCl_3$)

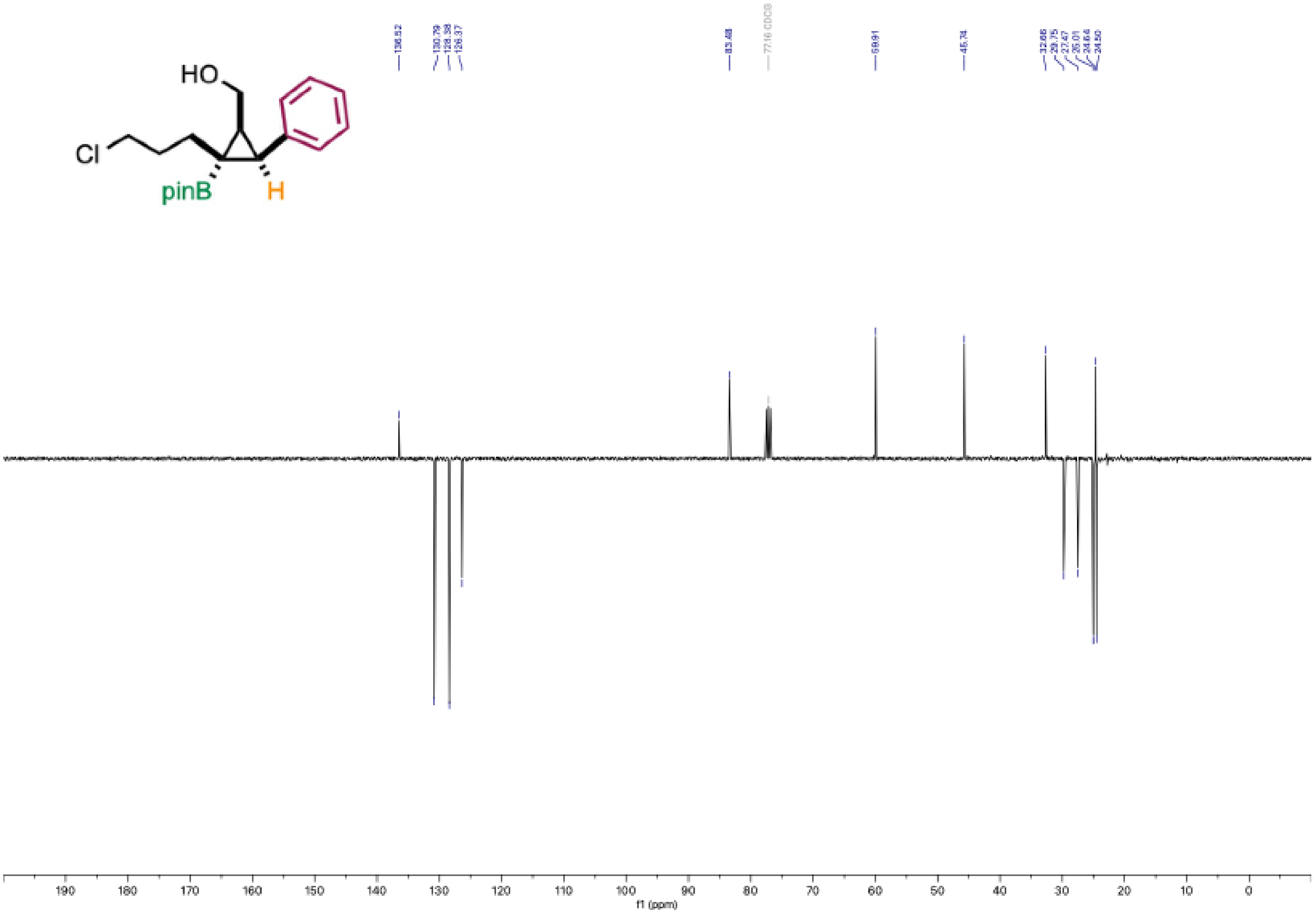

^{13}C NMR spectrum (101 MHz, $CDCl_3$)

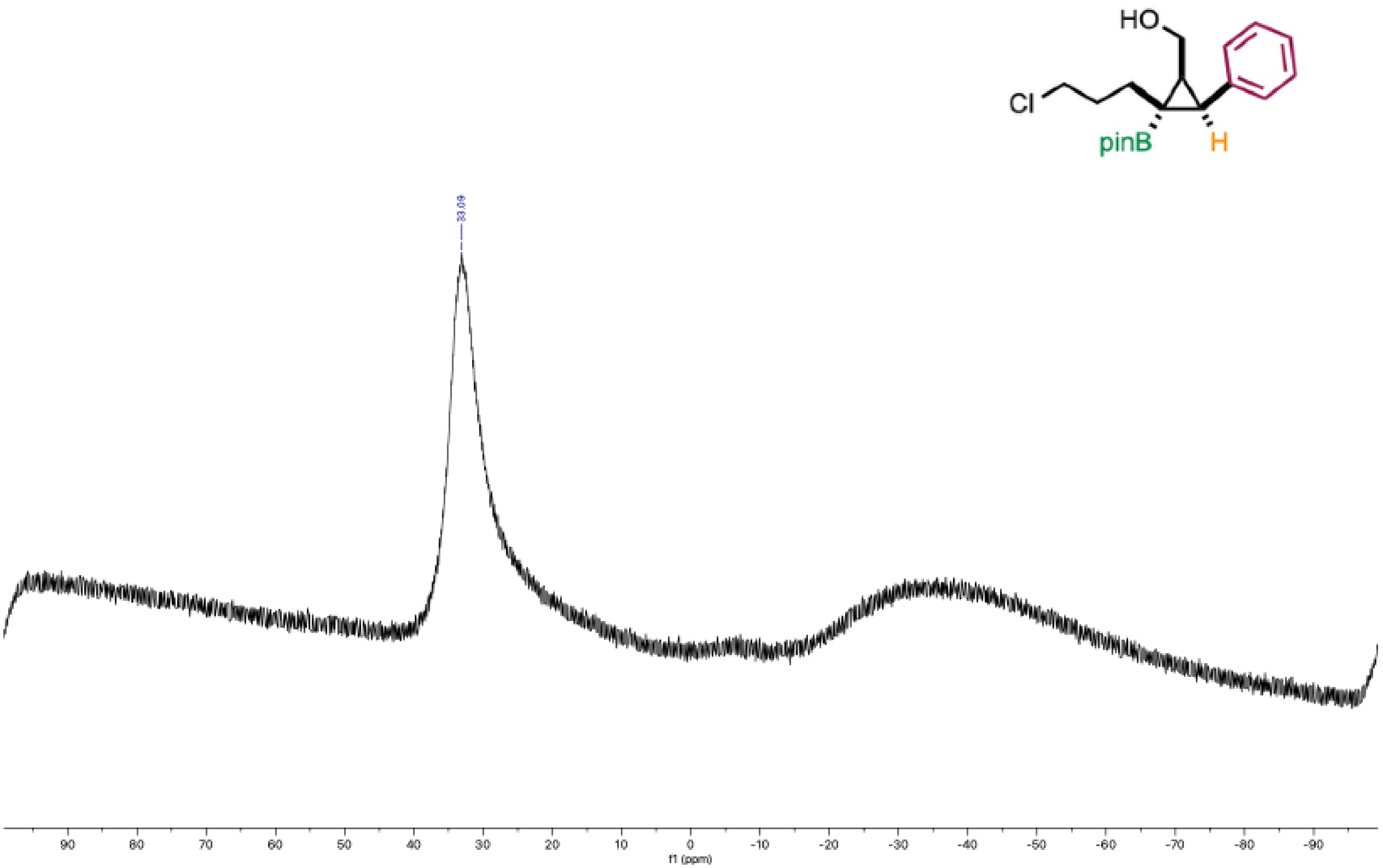

^{11}B NMR spectrum (128 MHz, $CDCl_3$)

((1*R,2*S**,3*R**)-2-Methyl-3-(2-methylallyl)-2-(4,4,5,5-tetramethyl-1,3,2-dioxaborolan-2-yl)-3-(4-(trifluoromethyl)phenyl)cyclopropyl)methanol 2m-OH**

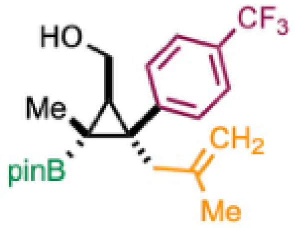

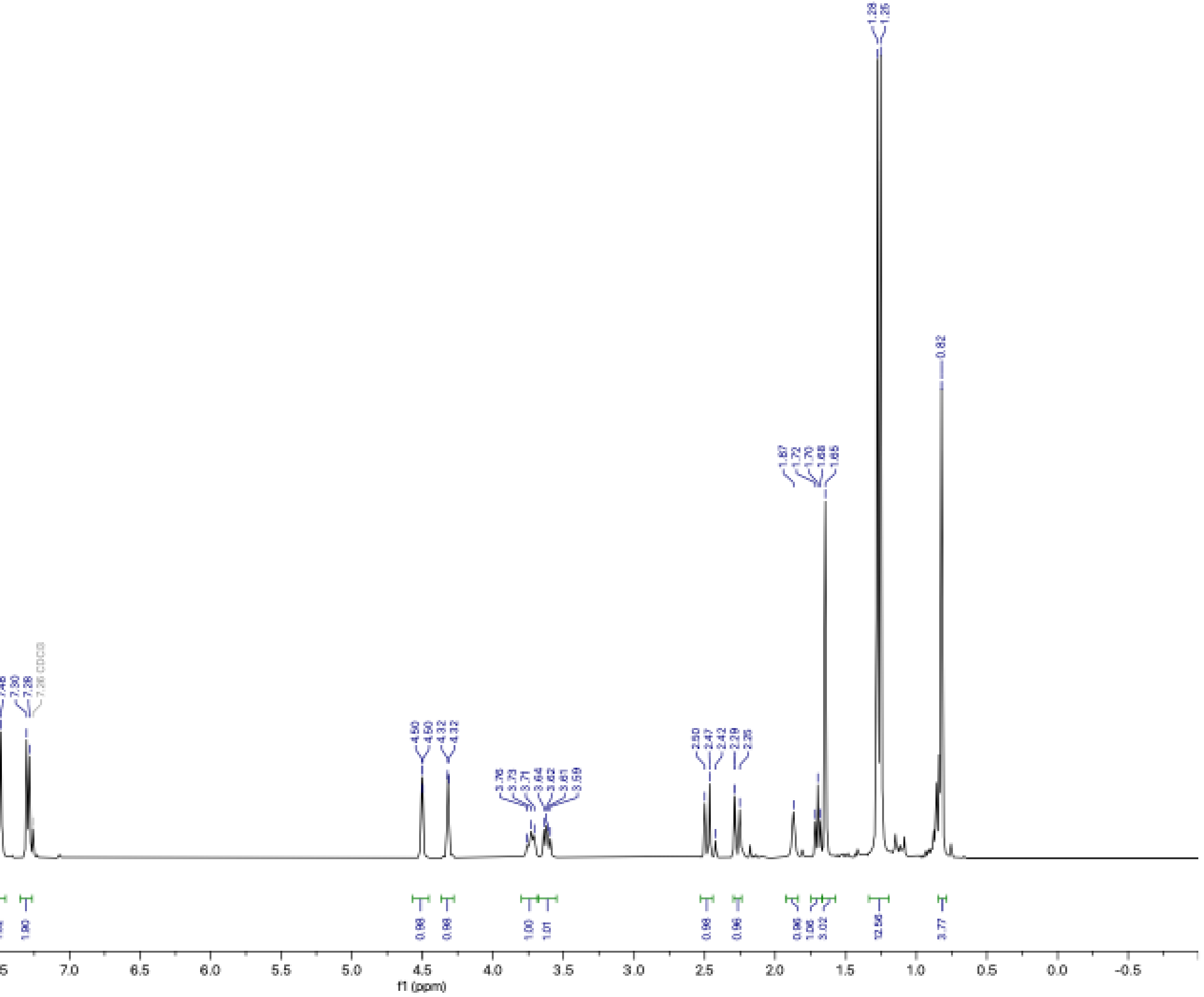

^{1}H NMR spectrum (400 MHz, $CDCl_3$)

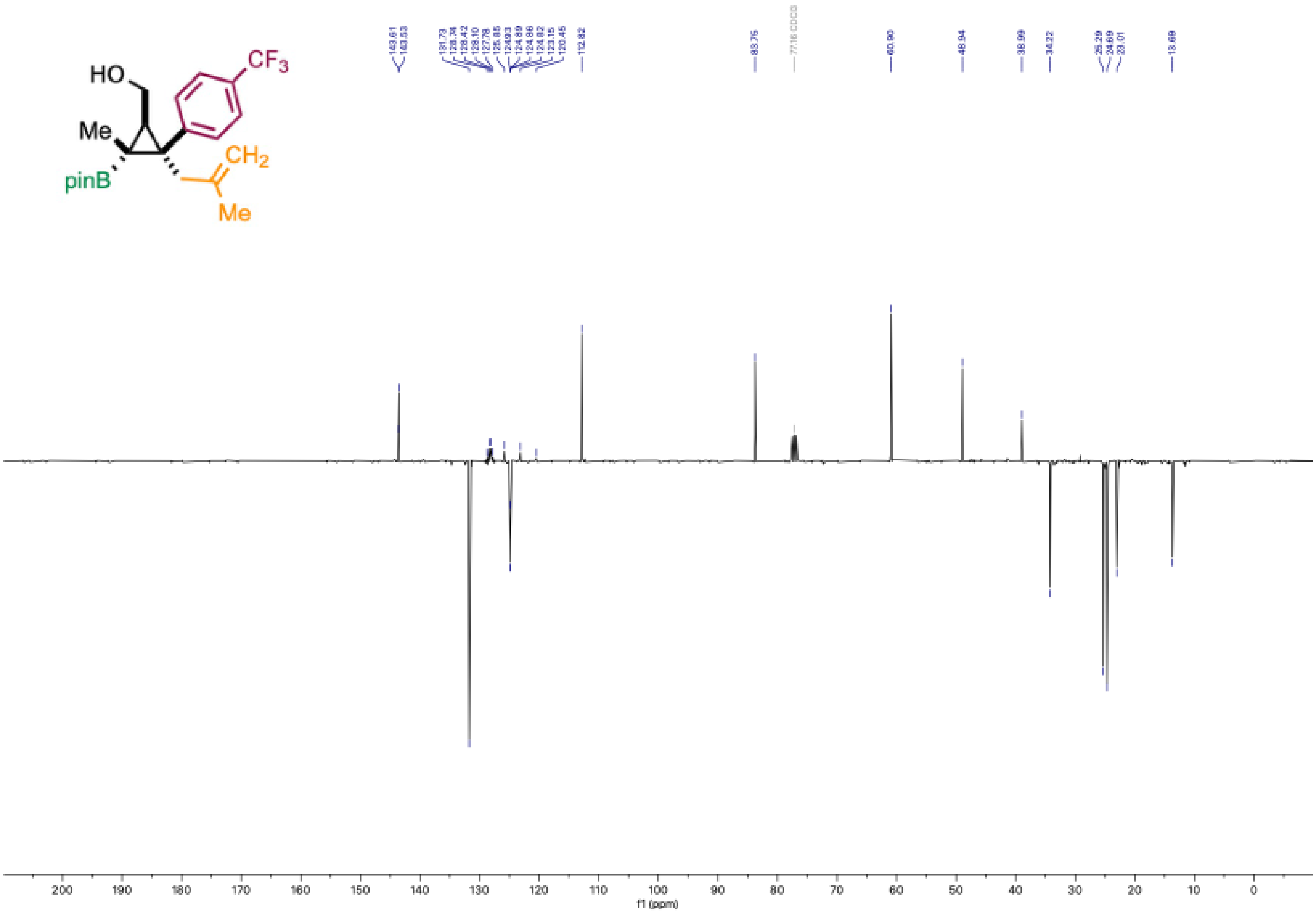

^{13}**C NMR** spectrum (101 MHz, $CDCl_3$)

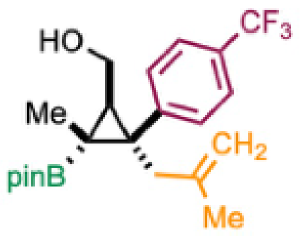

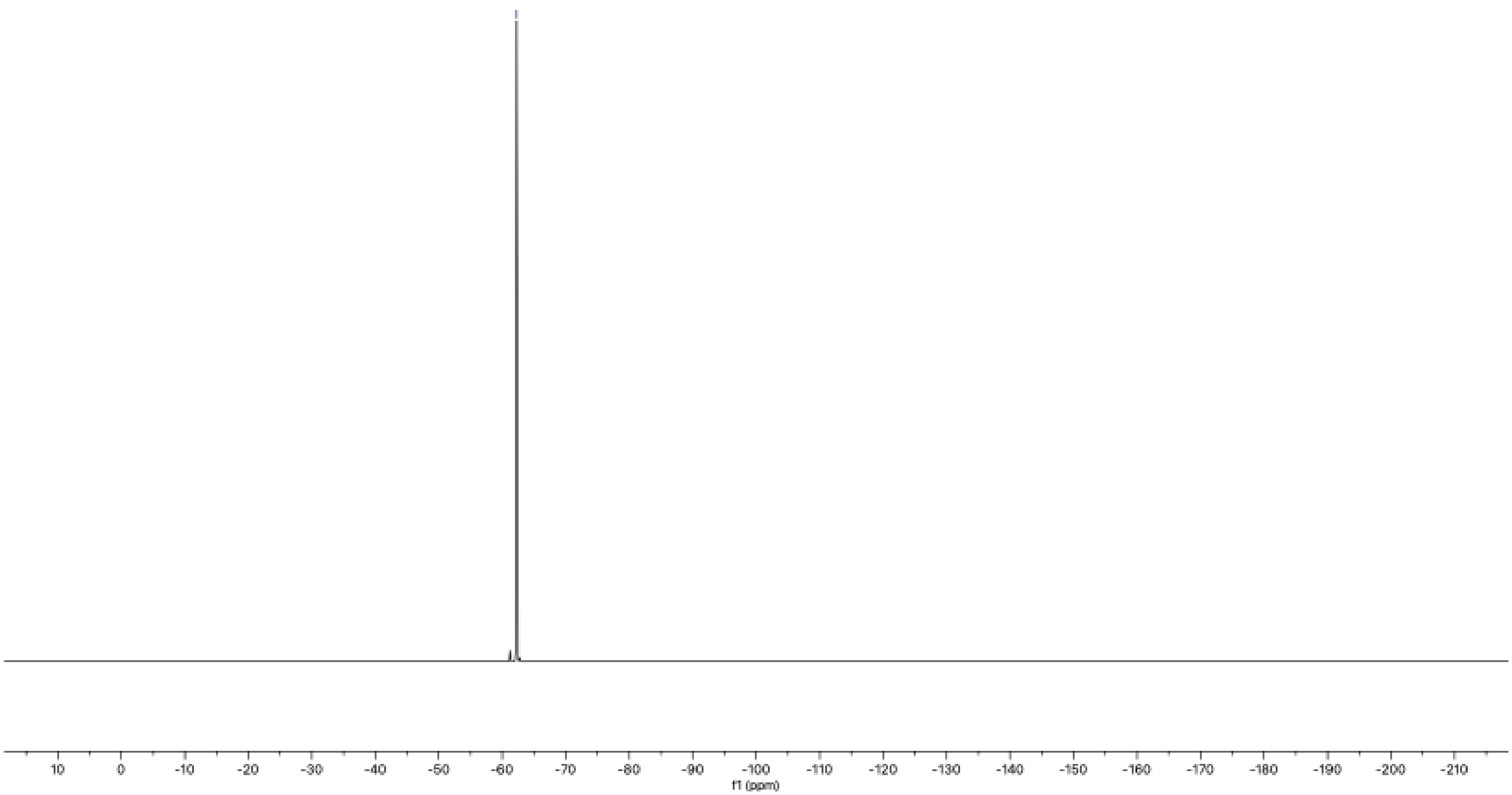

^{19}F NMR spectrum (377 MHz, $CDCl_3$)

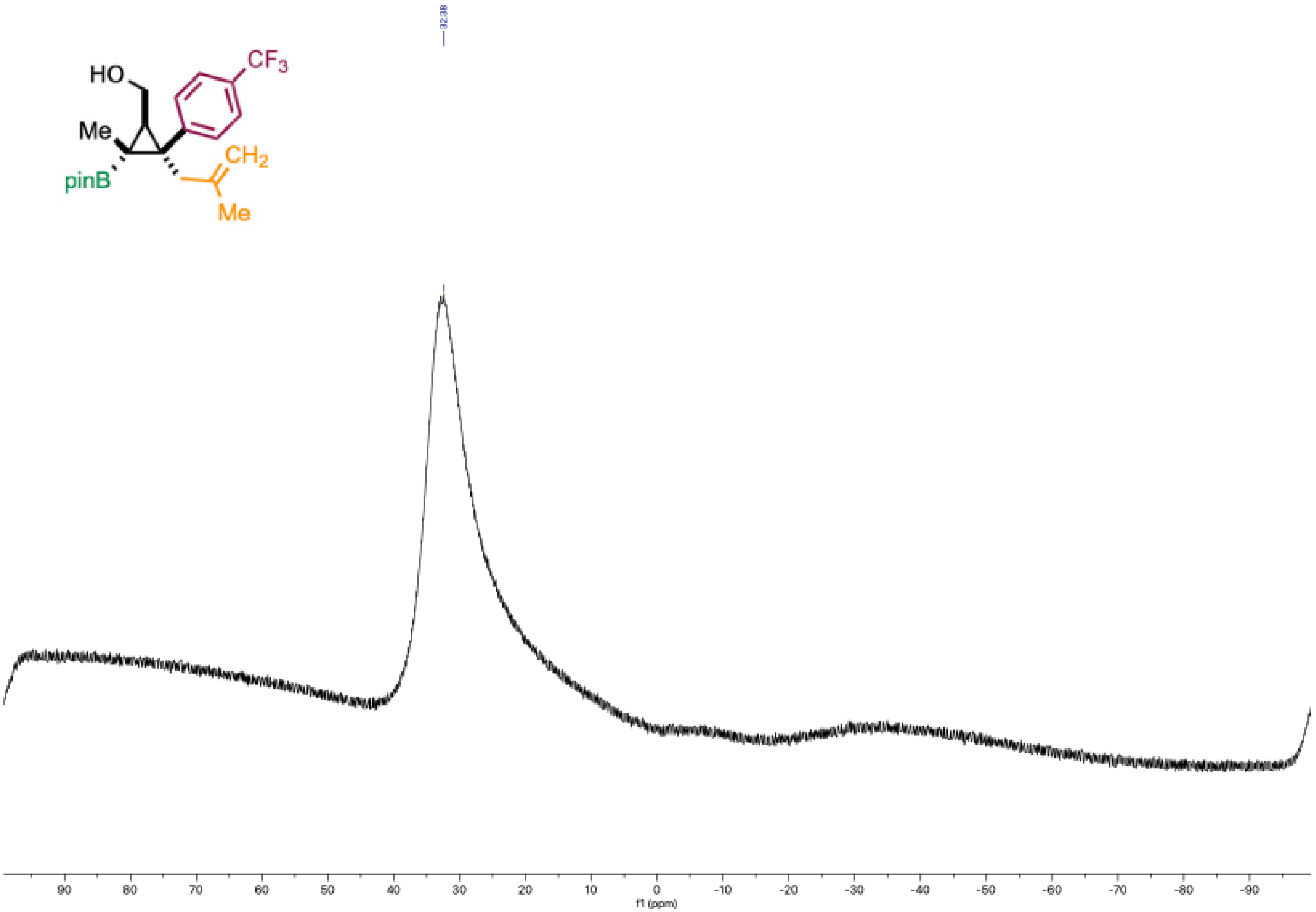

^{11}B NMR spectrum (128 MHz, $CDCl_3$)

1-((1*R,2*S**,3*R**)-3-(Hydroxymethyl)-2-methyl-2-(4,4,5,5-tetramethyl-1,3,2-dioxaborolan-2-yl)-1-(4-(trifluoromethyl)phenyl)cyclopropyl)propan-2-one 2n**-OH

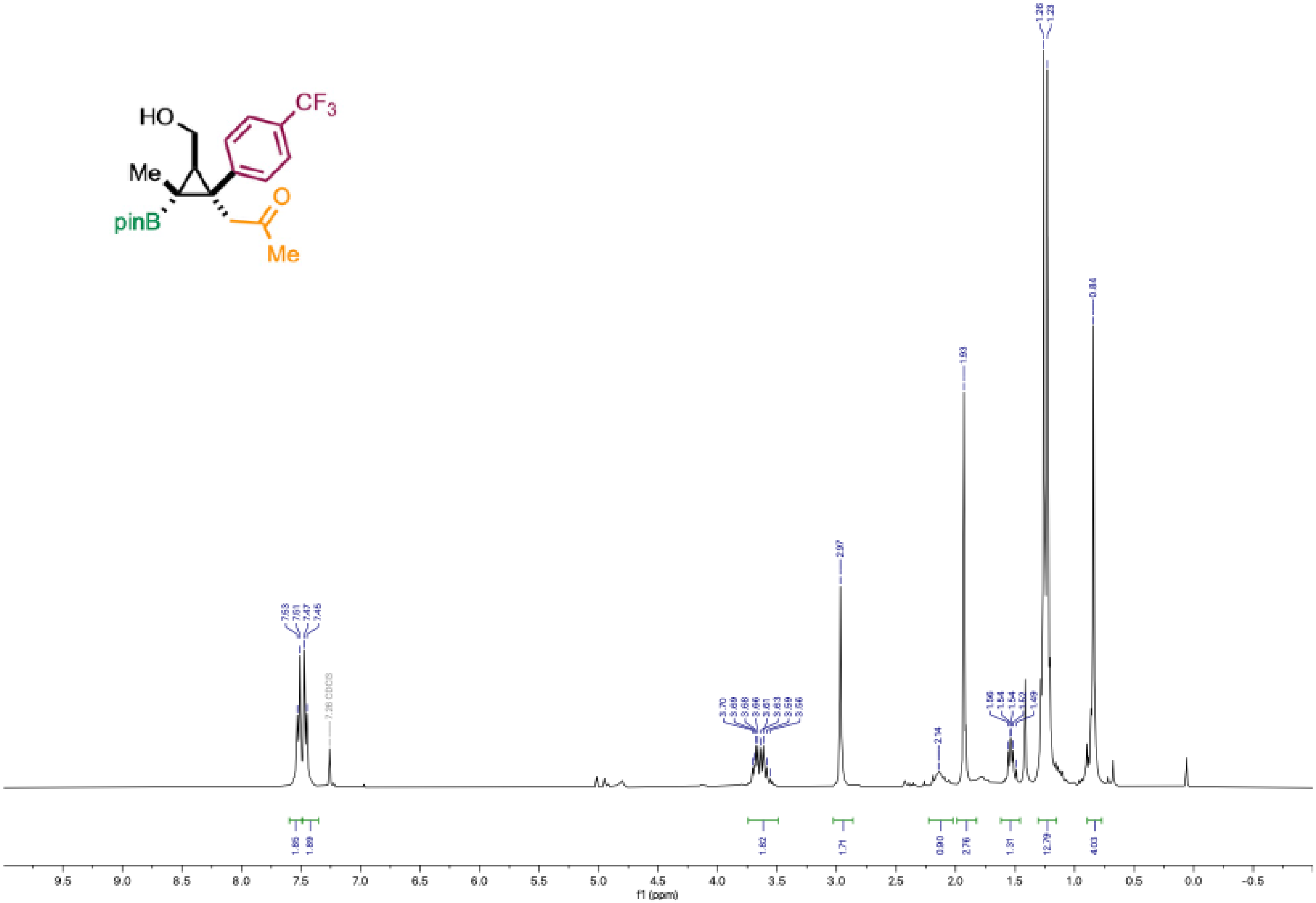

^{1}H NMR spectrum (400 MHz, $CDCl_3$)

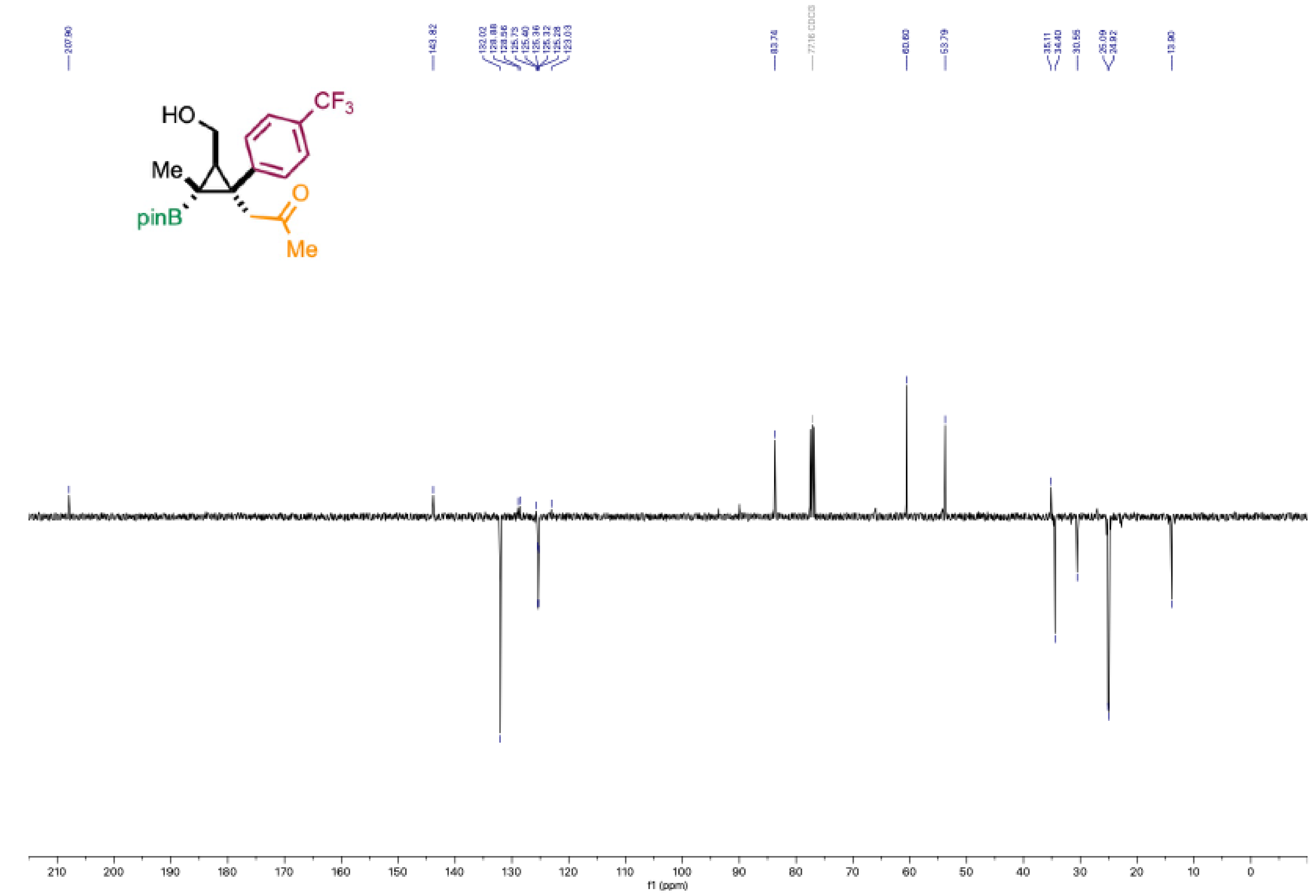

^{13}C NMR spectrum (101 MHz, $CDCl_3$)

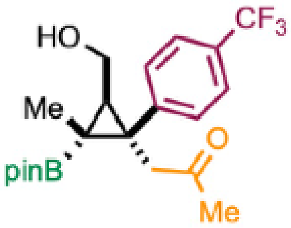

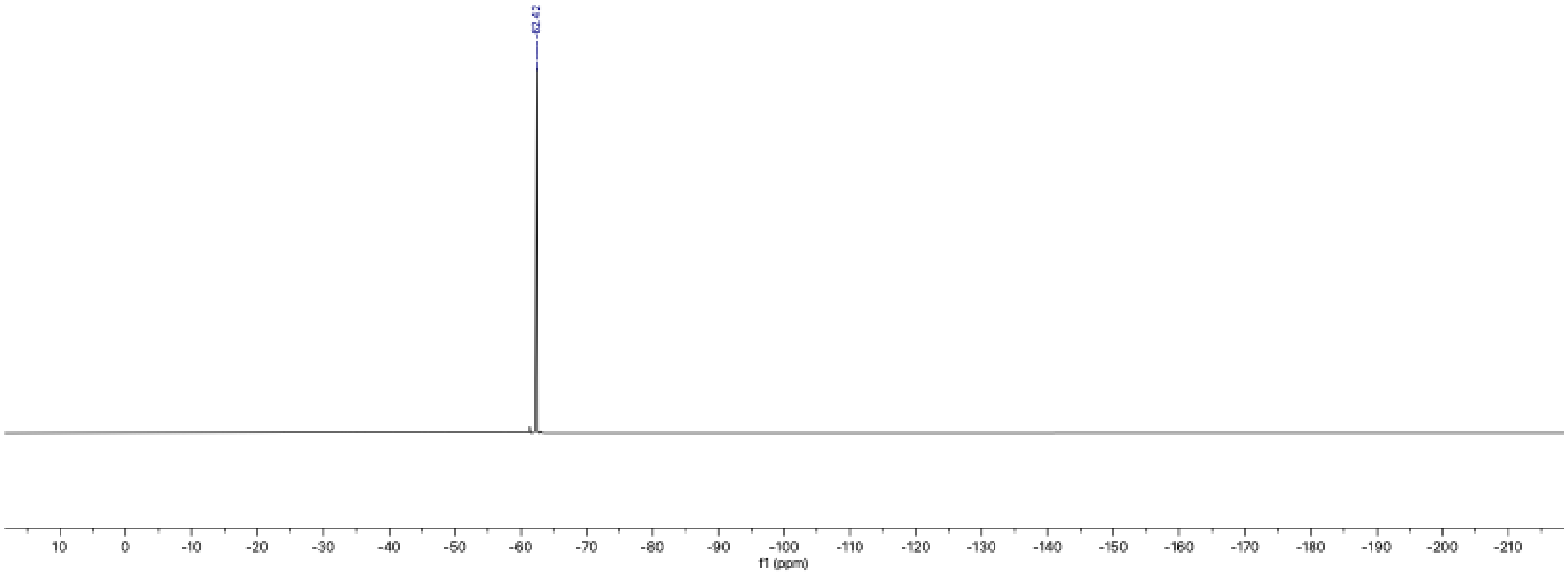

^{19}**F NMR** spectrum (377 MHz, $CDCl_3$)

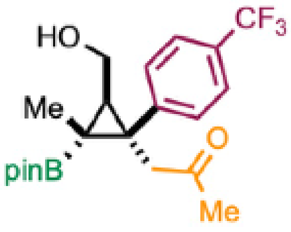

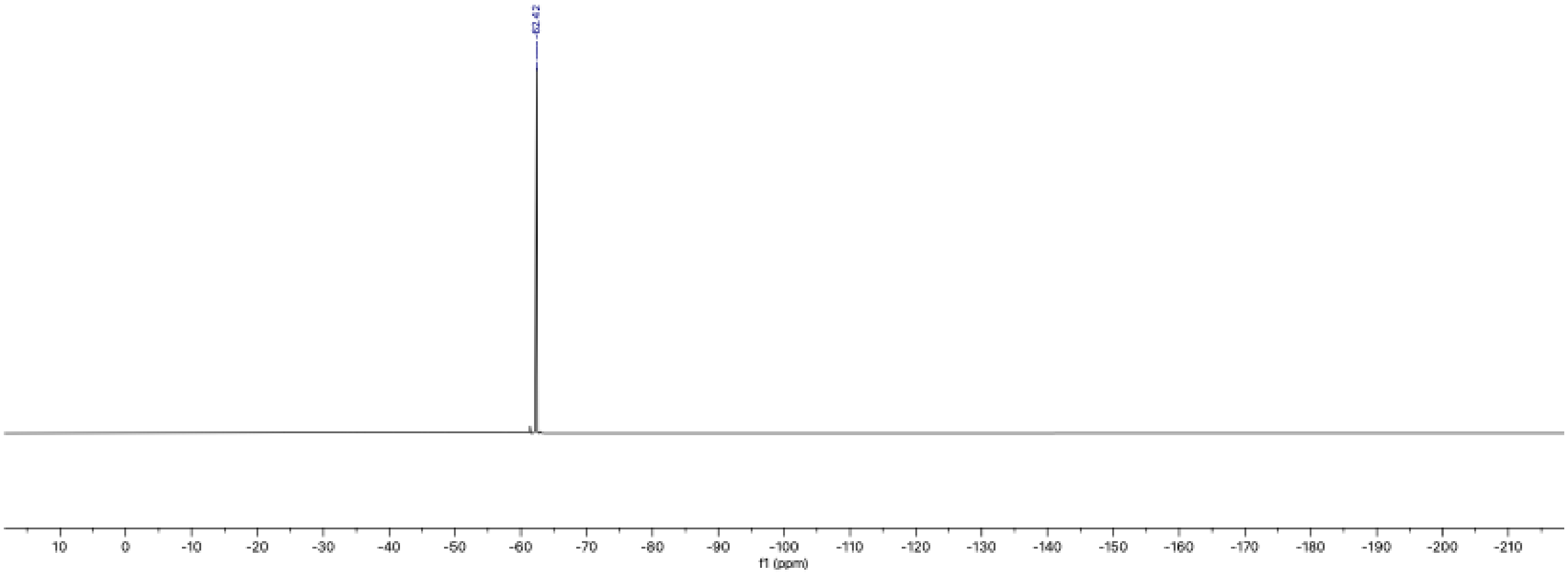

^{11}B NMR spectrum (128 MHz, $CDCl_3$)

2-((1*S,2*S**,3*R**)-1-Ethyl-2-(iodomethyl)-3-(thiophen-2-yl)cyclopropyl)-4,4,5,5-tetramethyl-1,3,2-dioxaborolane 2h**

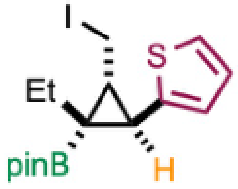

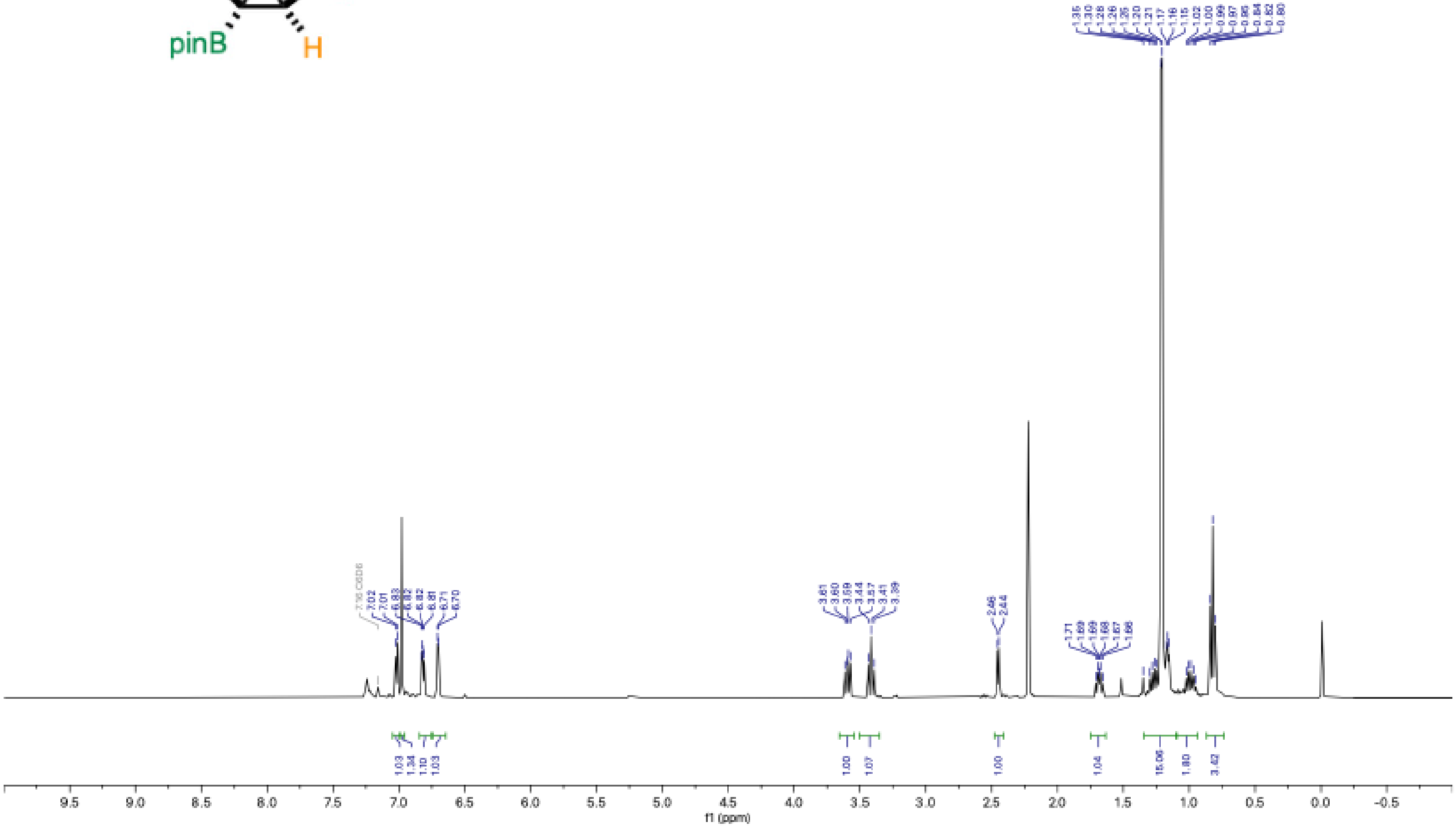

¹H NMR spectrum (400 MHz, $CDCl_3$)

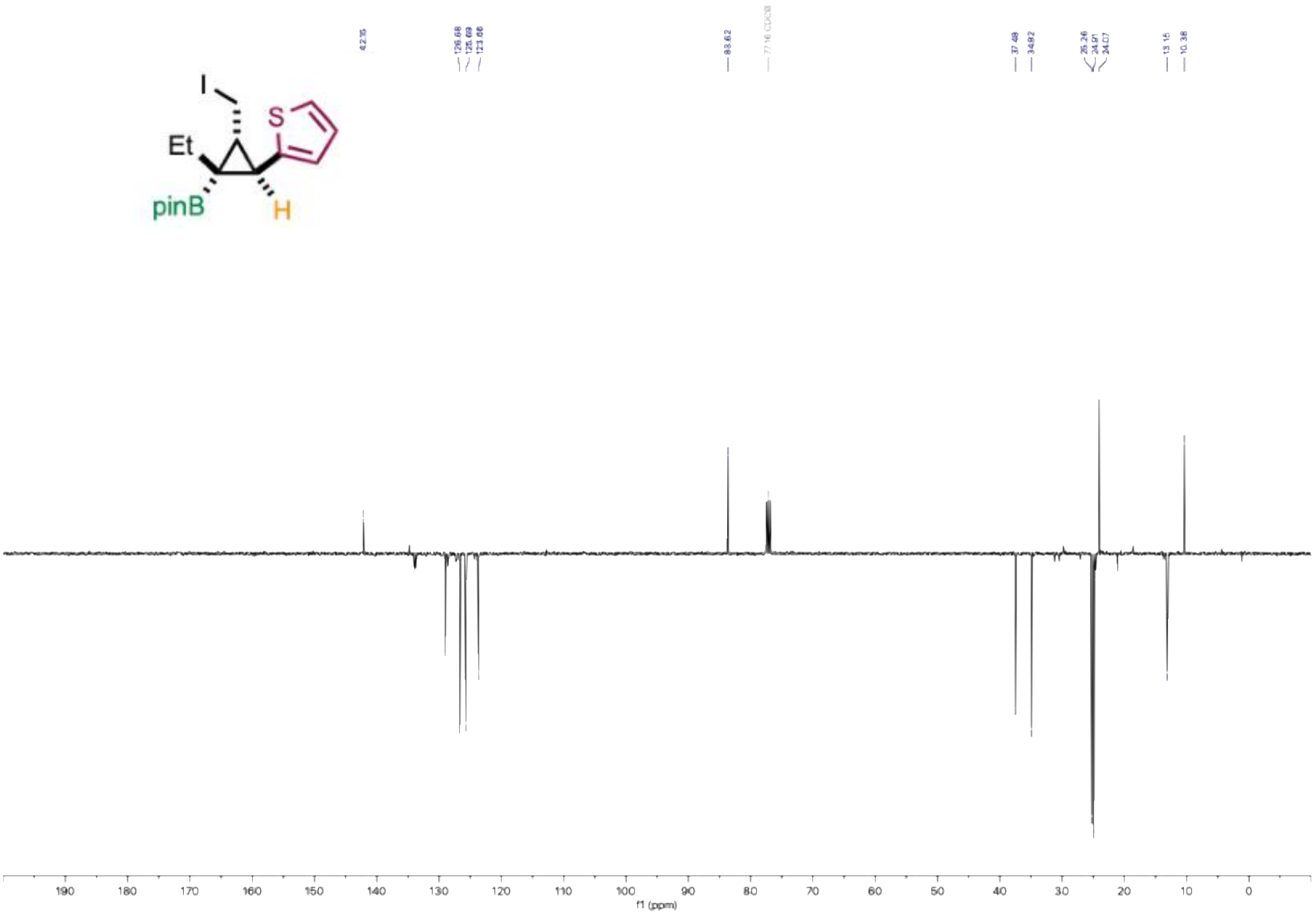

^{13}C NMR spectrum (101 MHz, $CDCl_3$)

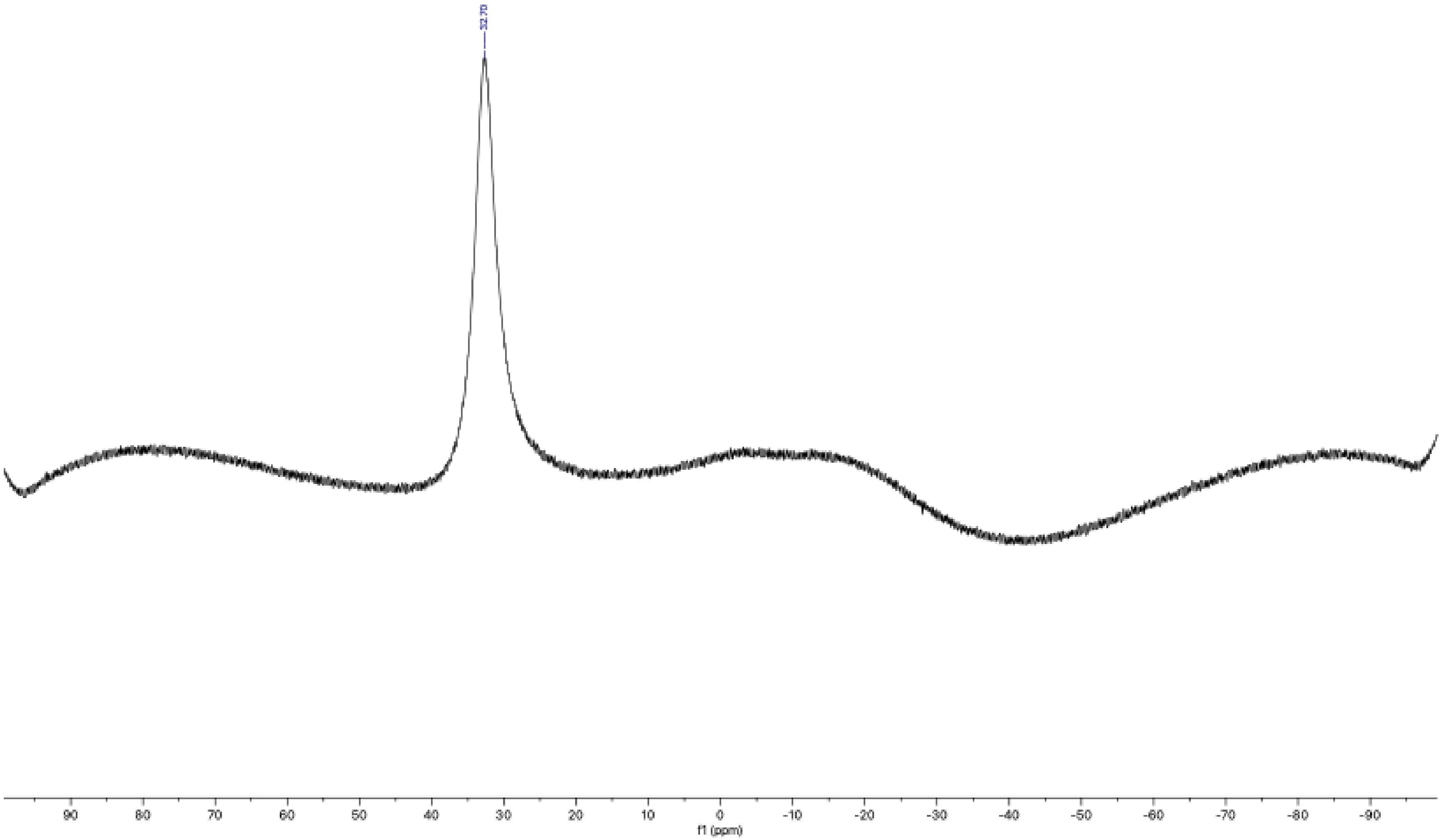

^{11}B NMR spectrum (128 MHz, $CDCl_3$)

Trimethyl((3*S,4*S**)-4-phenyl-3-(4,4,5,5-tetramethyl-1,3,2-dioxaborolan-2-yl)hex-5-en-3-yl)silane 5a**

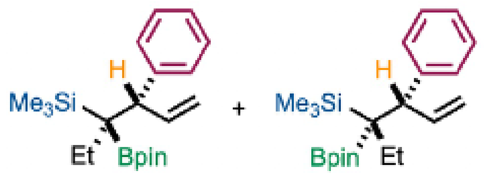

dr 90:10

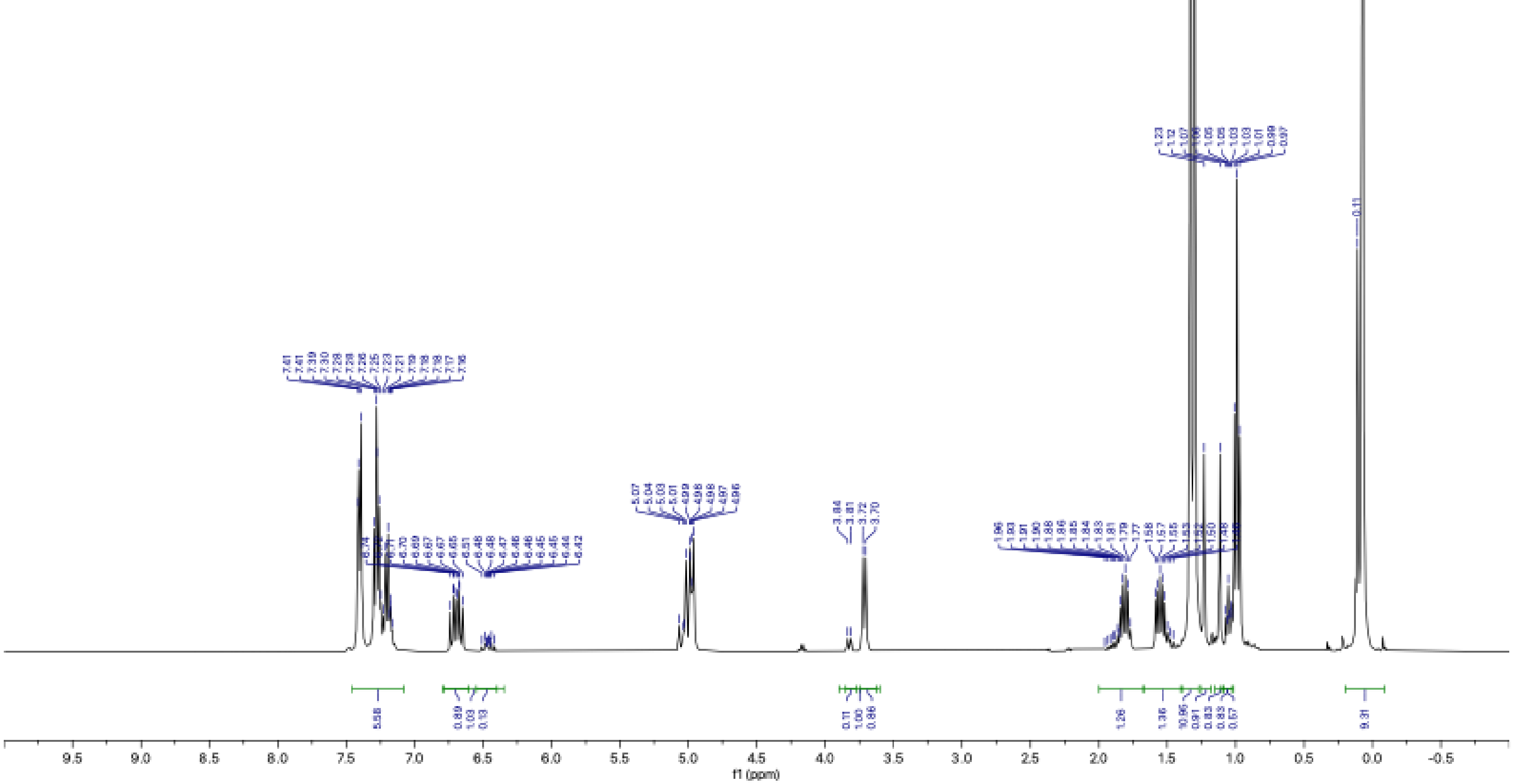

^{1}H NMR spectrum (400 MHz, $CDCl_3$)

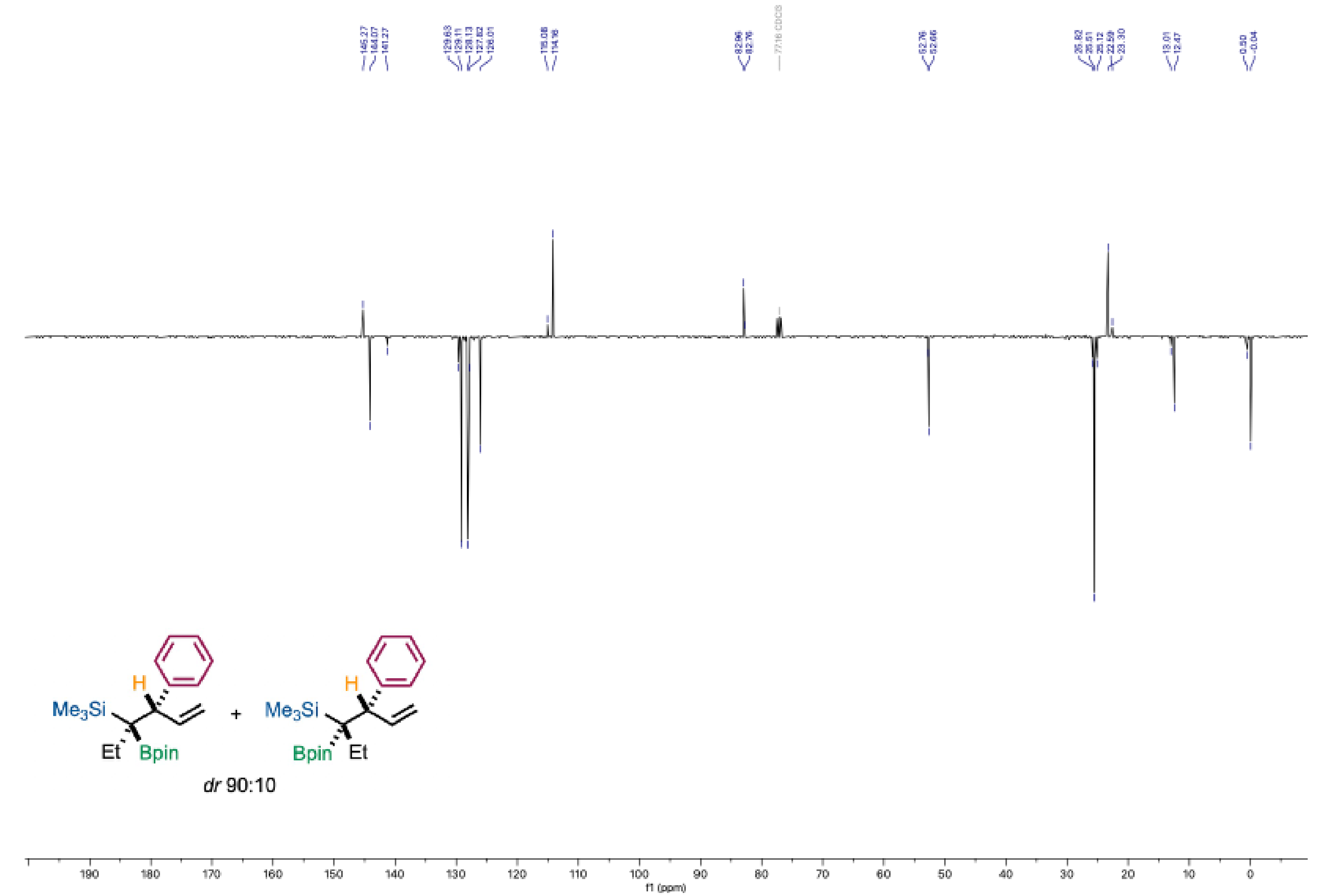

^{13}C NMR spectrum (101 MHz, $CDCl_3$)

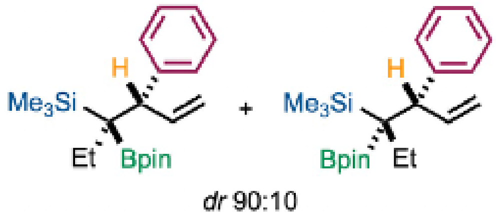

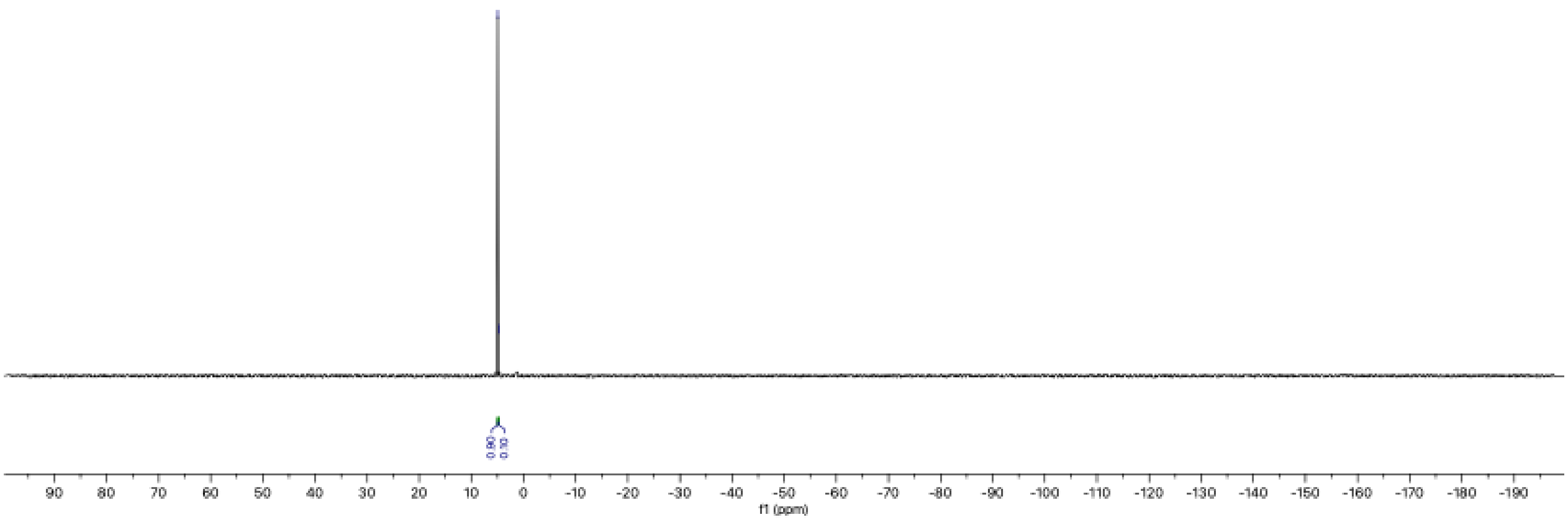

^{29}Si NMR spectrum (80 MHz, $CDCl_3$)

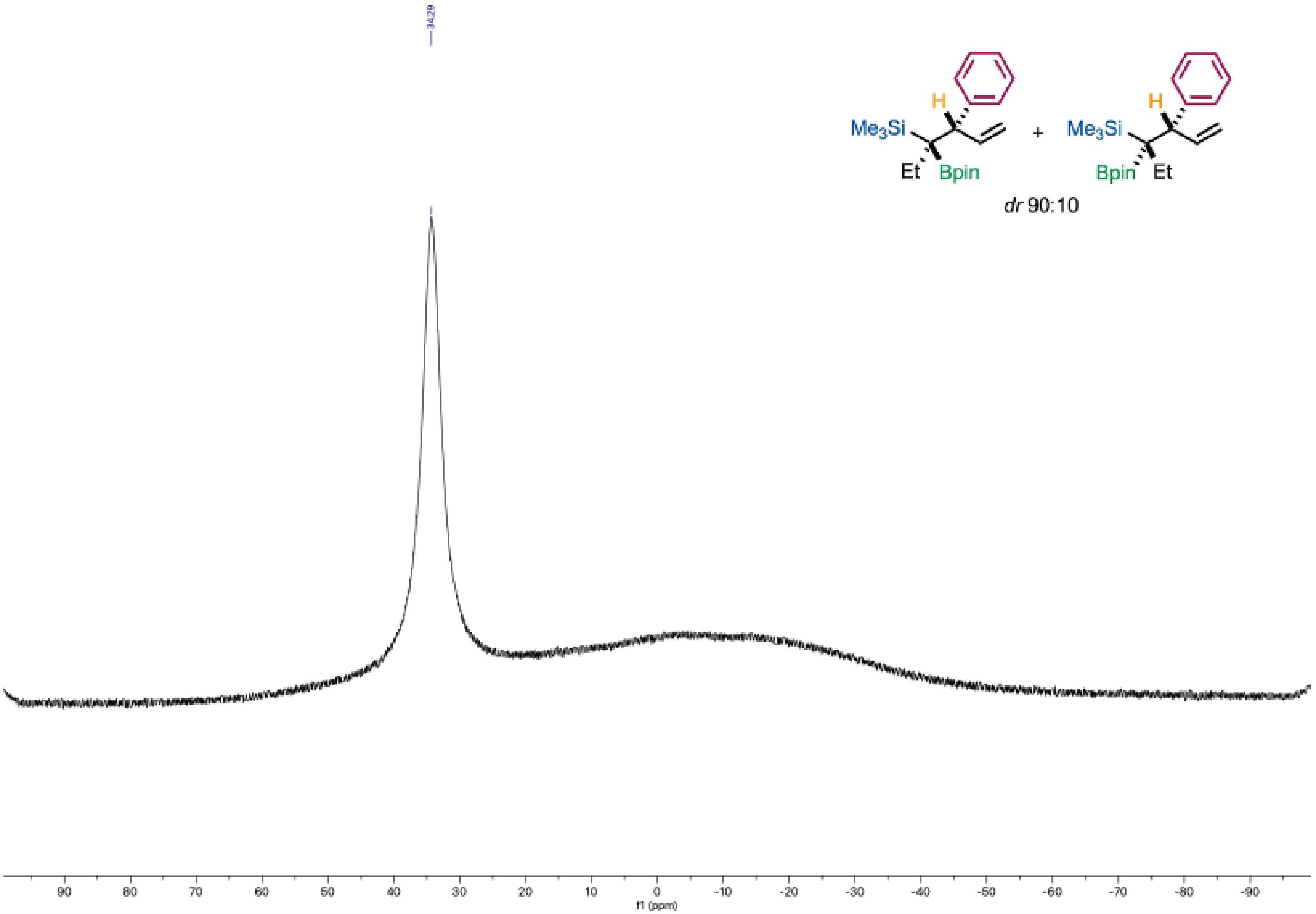

^{11}B NMR spectrum (128 MHz, $CDCl_3$)

Dimethyl(phenyl)((3*S,4*S**)-4-phenyl-3-(4,4,5,5-tetramethyl-1,3,2-dioxaborolan-2-yl)hex-5-en-3-yl)silane 5b**

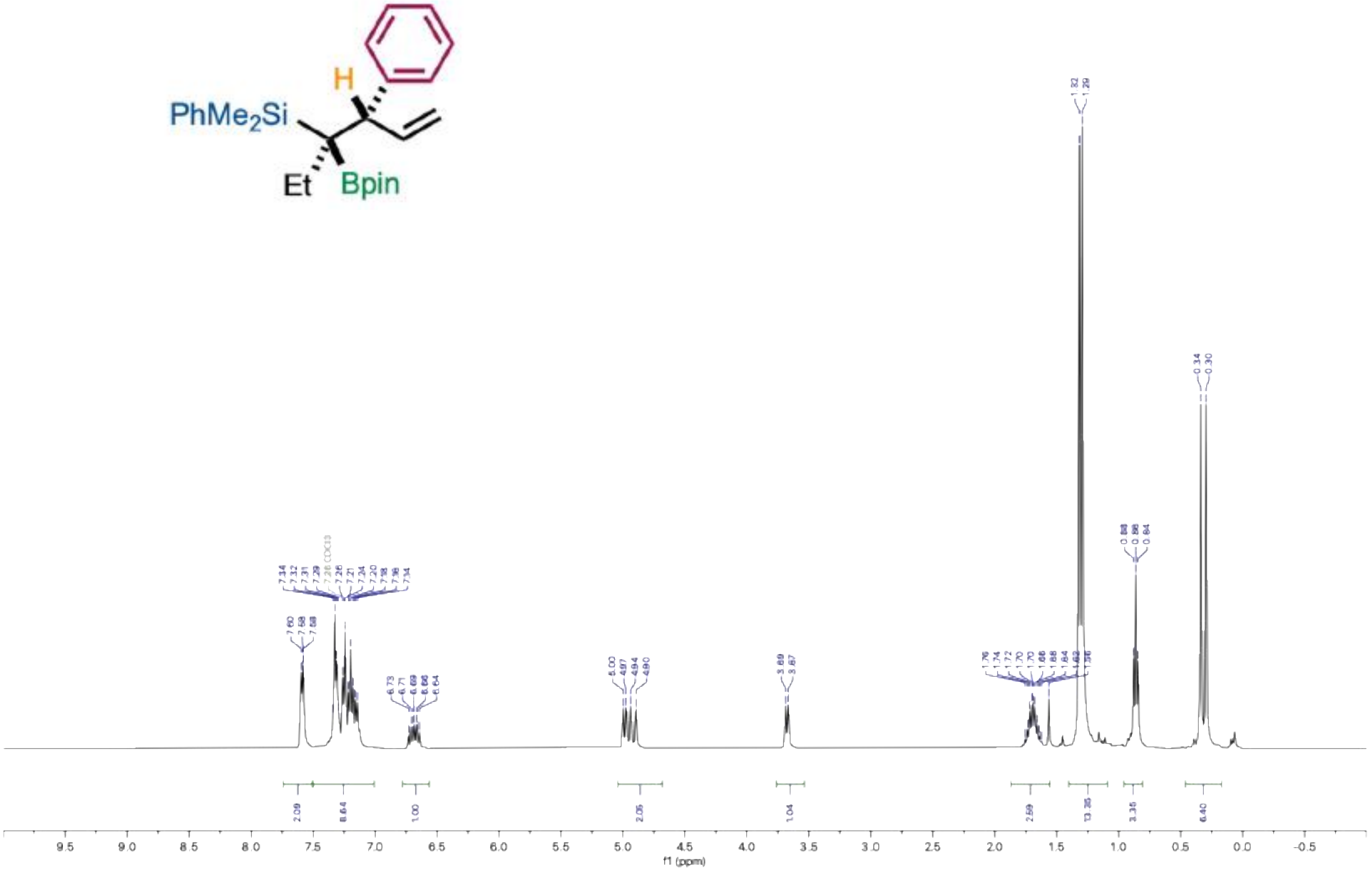

^{1}H NMR spectrum (400 MHz, $CDCl_3$)

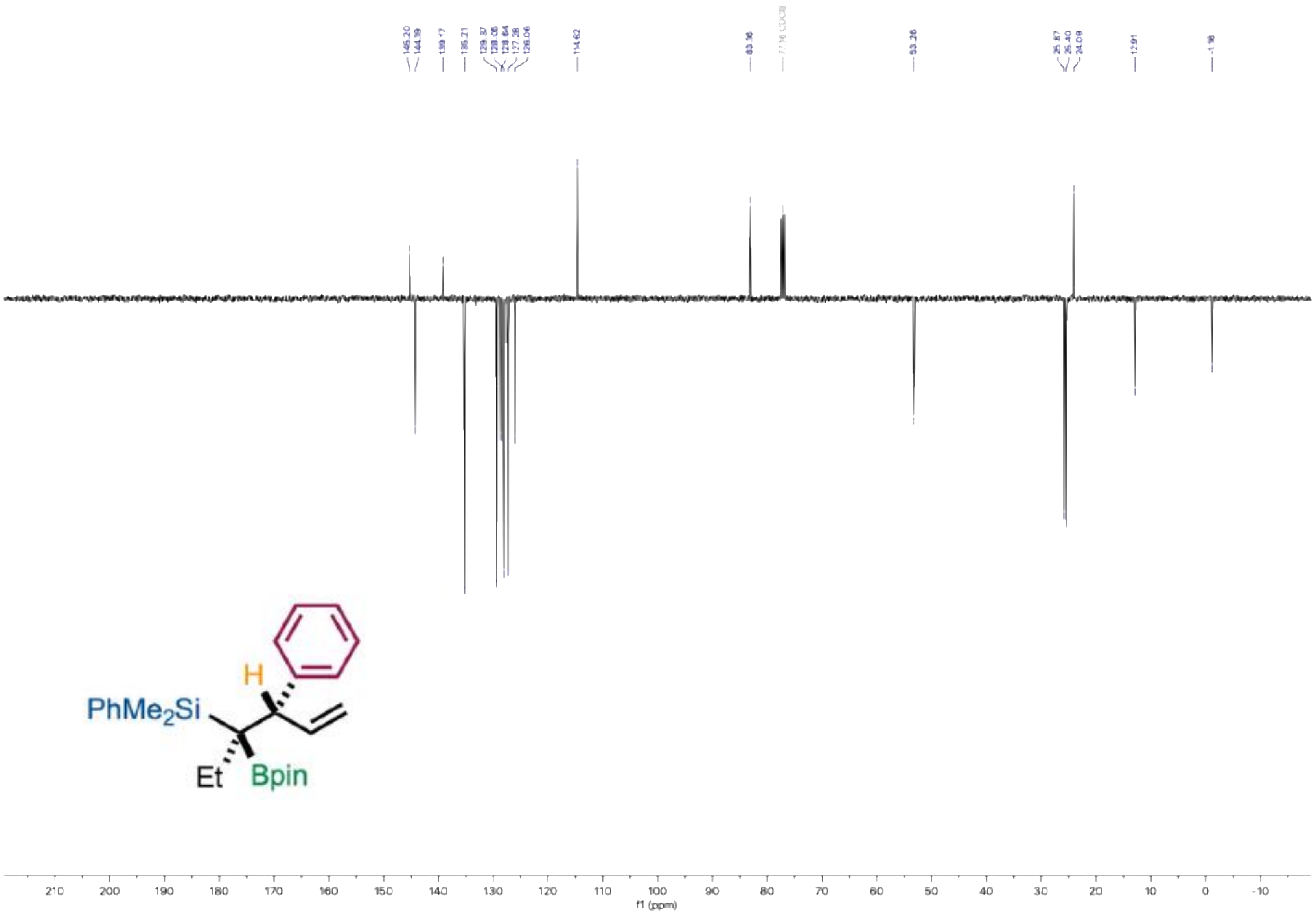

^{13}C NMR spectrum (101 MHz, $CDCl_3$)

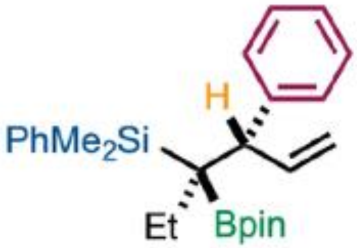

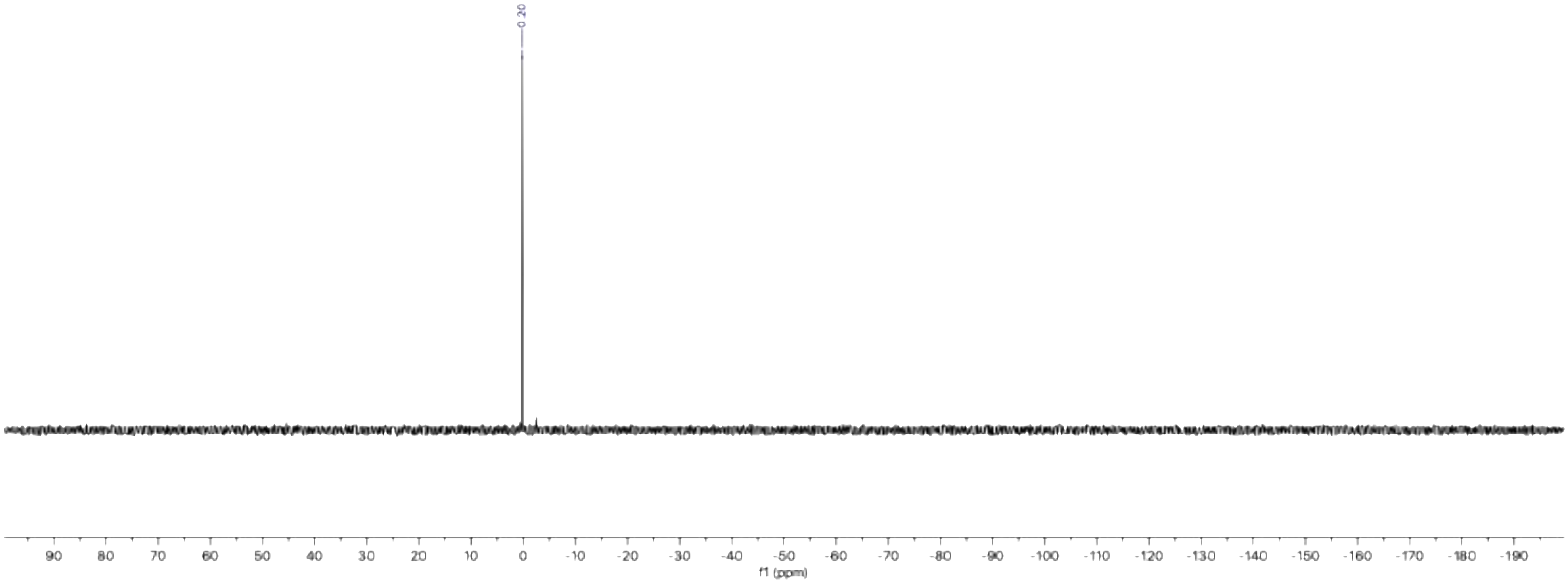

^{29}Si NMR spectrum (80 MHz, $CDCl_3$)

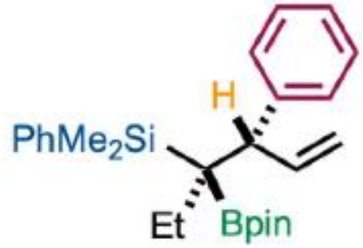

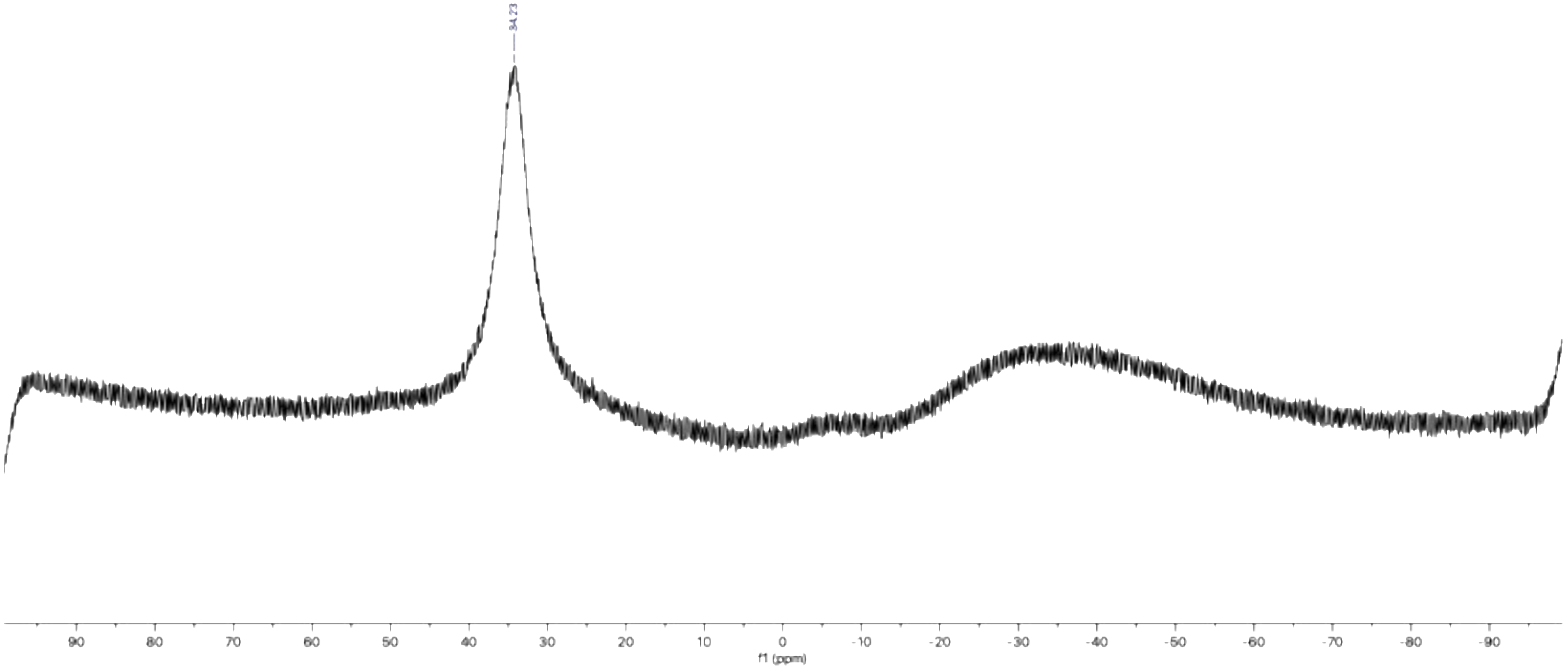

^{11}B NMR spectrum (128 MHz, $CDCl_3$)

Trimethyl((2*S,3*S**)-3-phenyl-2-(4,4,5,5-tetramethyl-1,3,2-dioxaborolan-2-yl)pent-4-en-2-yl)silane 5c**

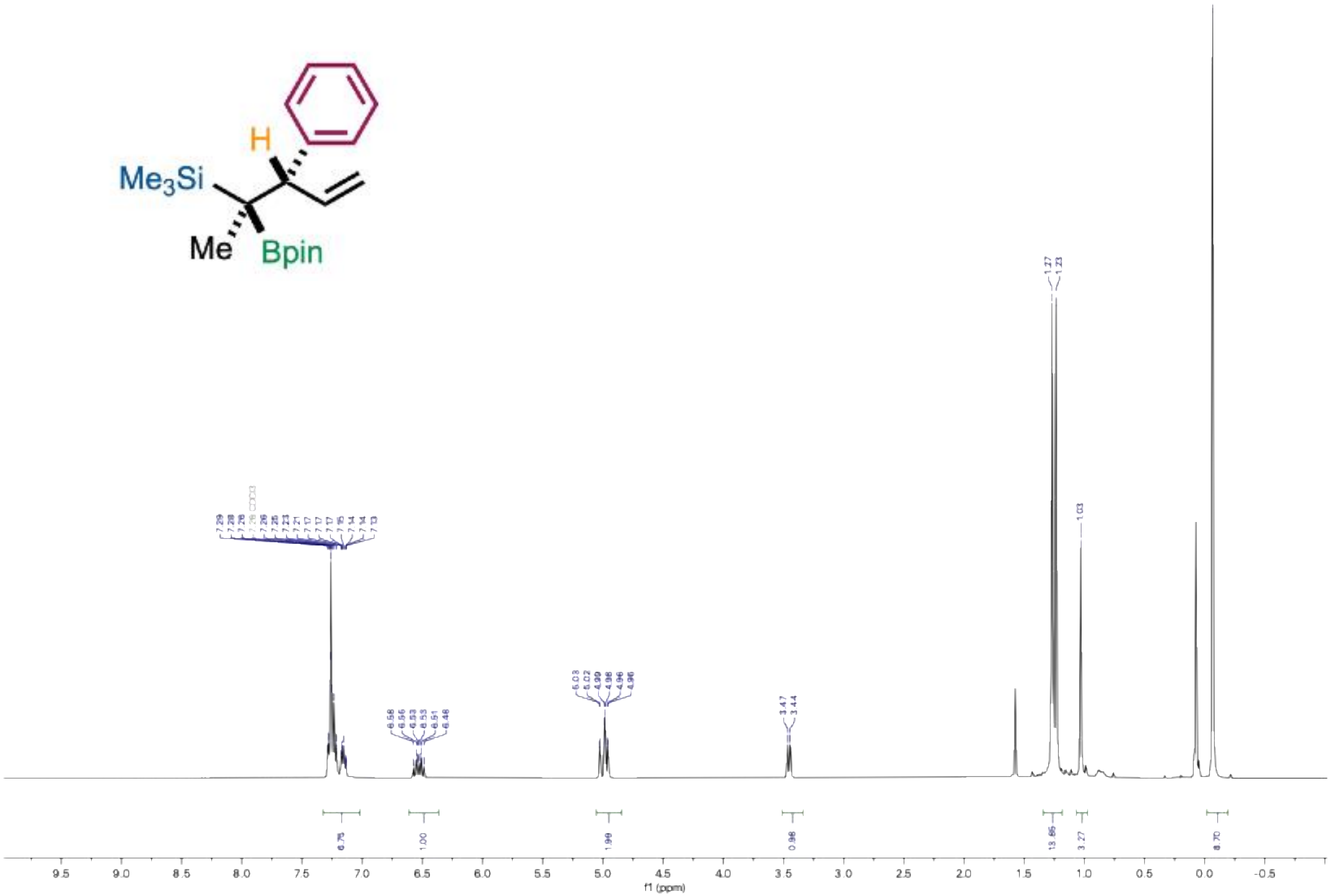

^{1}H NMR spectrum (400 MHz, $CDCl_3$)

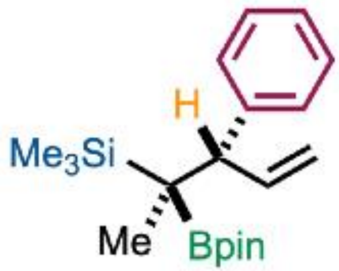

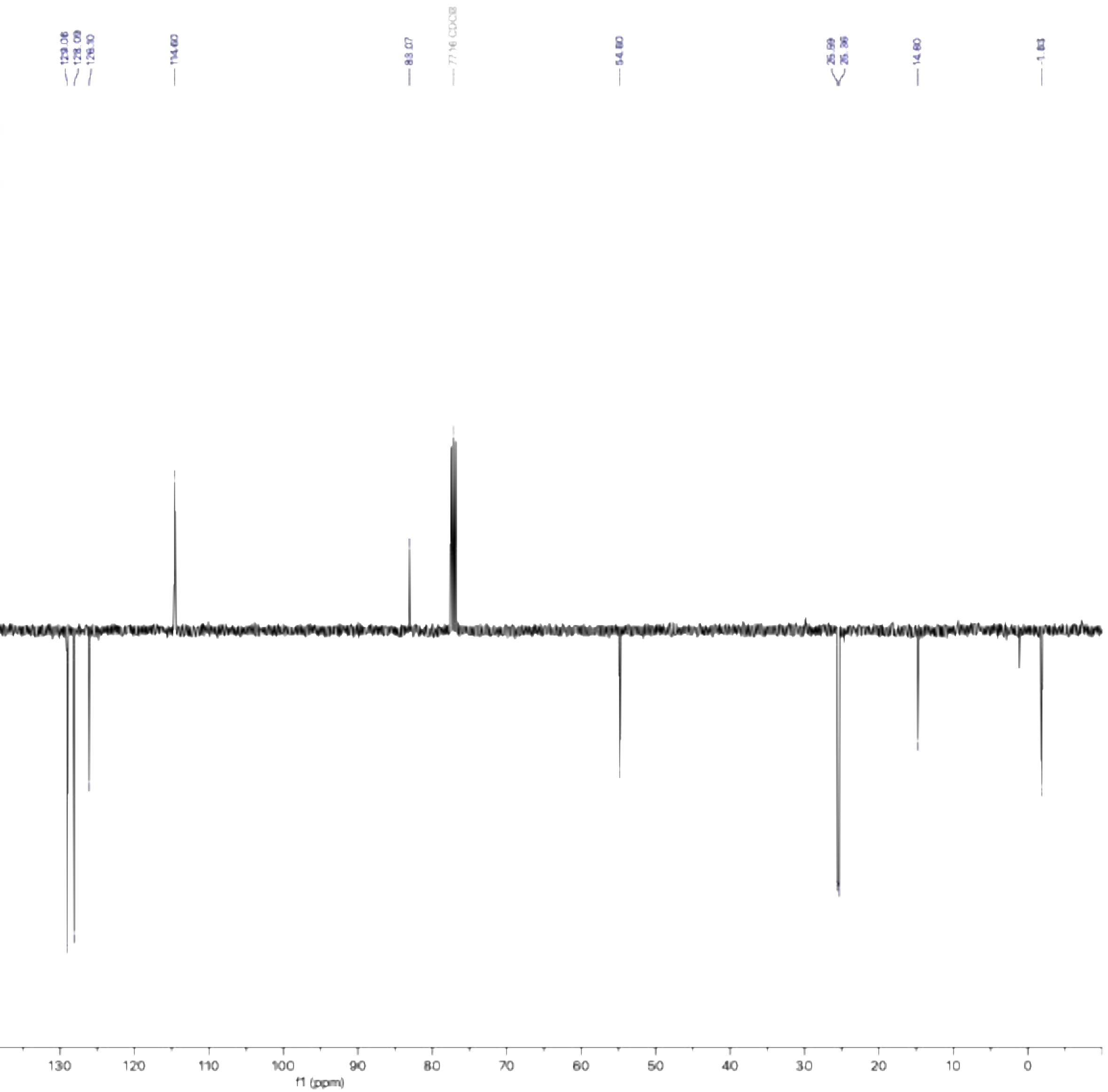

^{13}C NMR spectrum (101 MHz, $CDCl_3$)

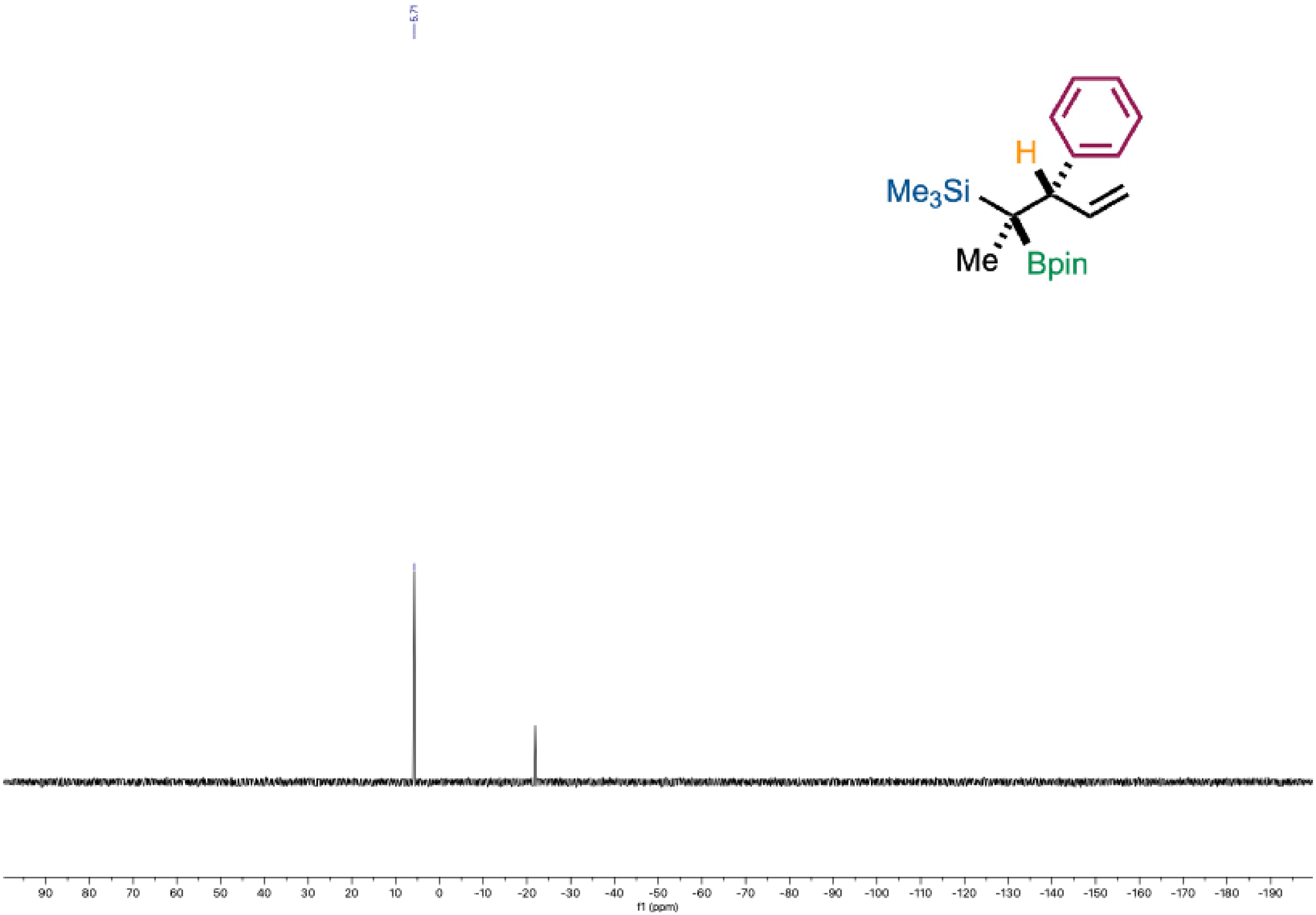

^{29}Si NMR spectrum (80 MHz, $CDCl_3$)

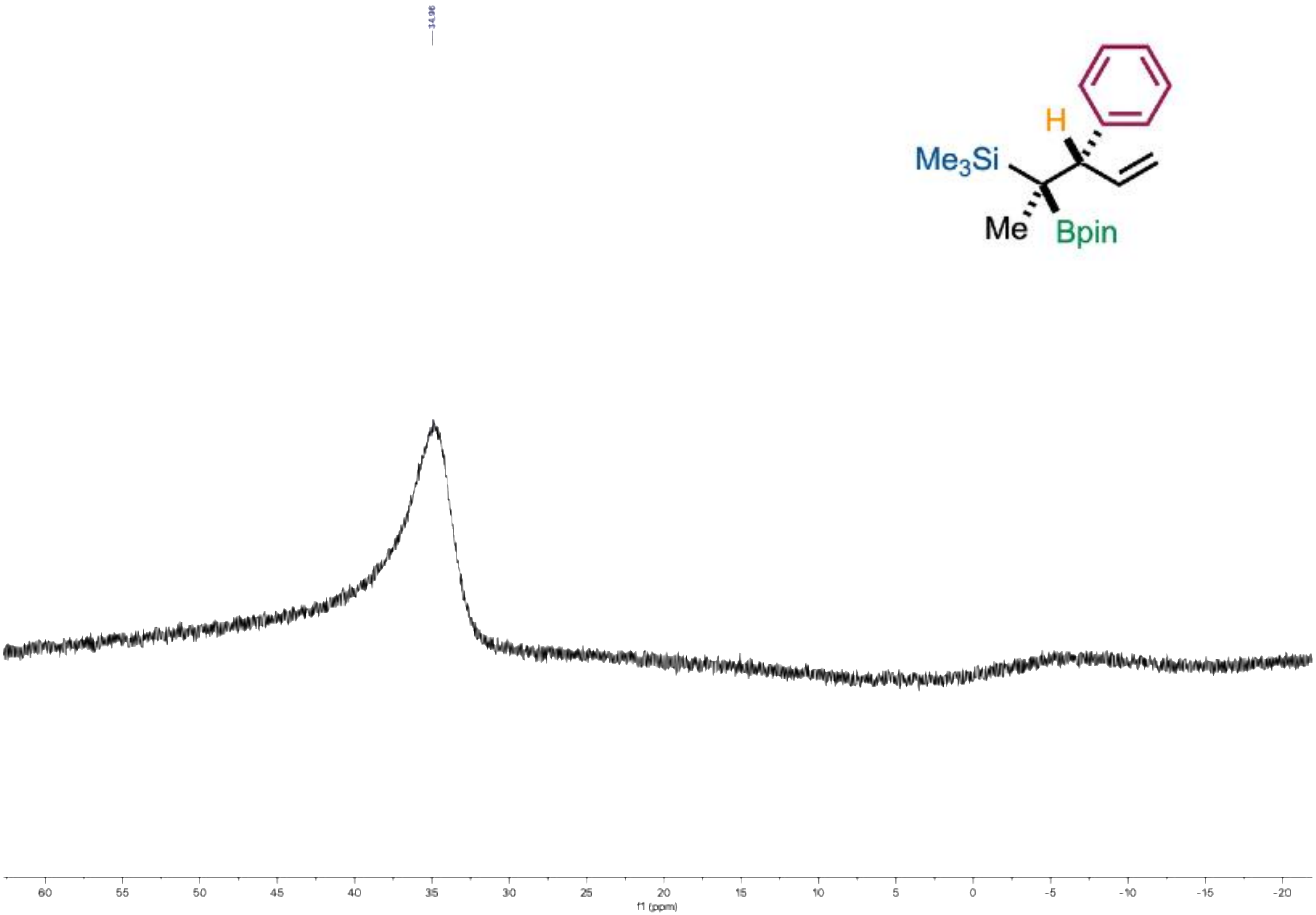

^{11}B NMR spectrum (128 MHz, $CDCl_3$)

Trimethyl((2*S,3*S**)-2-(4,4,5,5-tetramethyl-1,3,2-dioxaborolan-2-yl)-3-(*p*-tolyl)pent-4-en-2-yl)silane 5d**

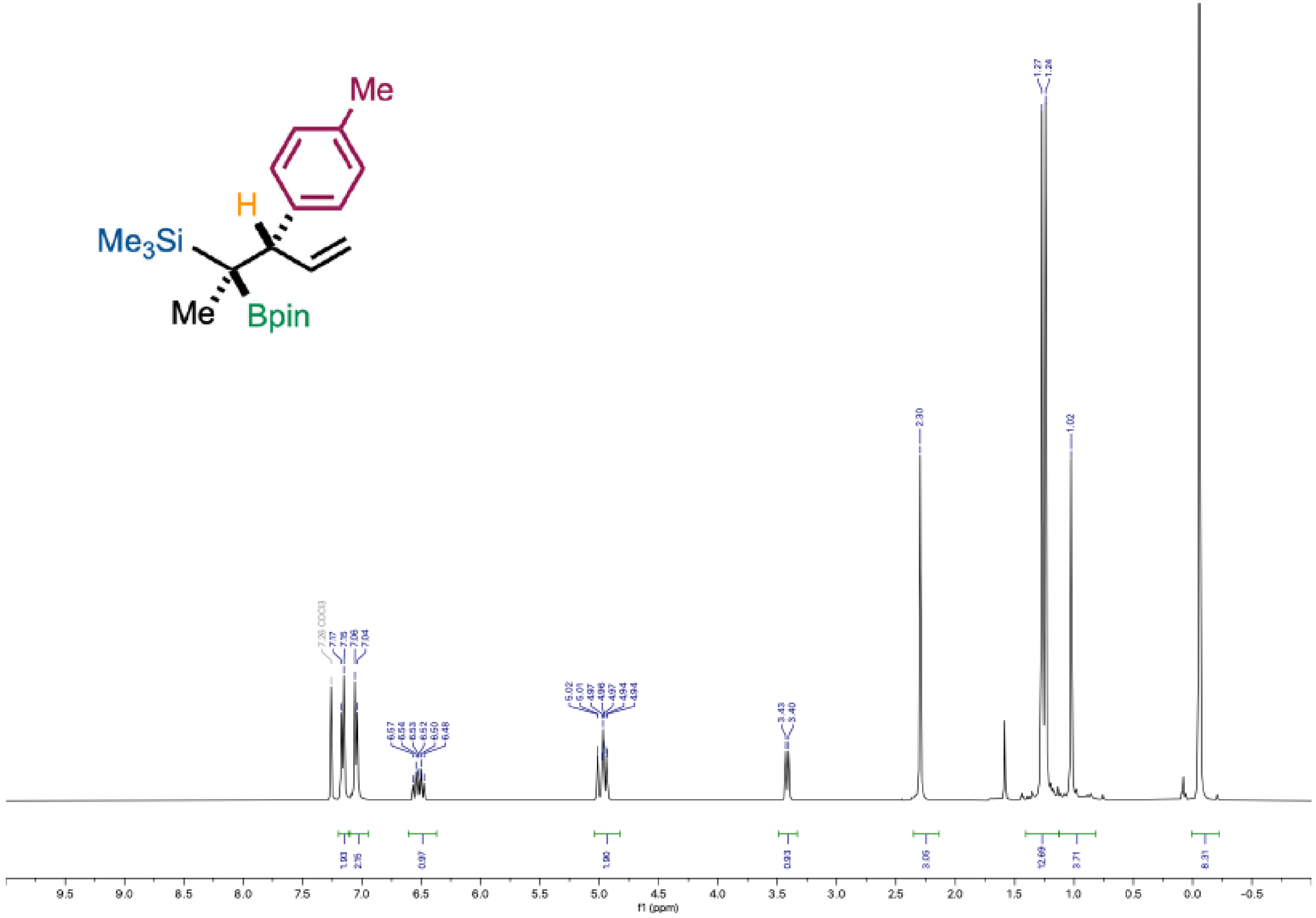

^{1}H NMR spectrum (400 MHz, $CDCl_3$)

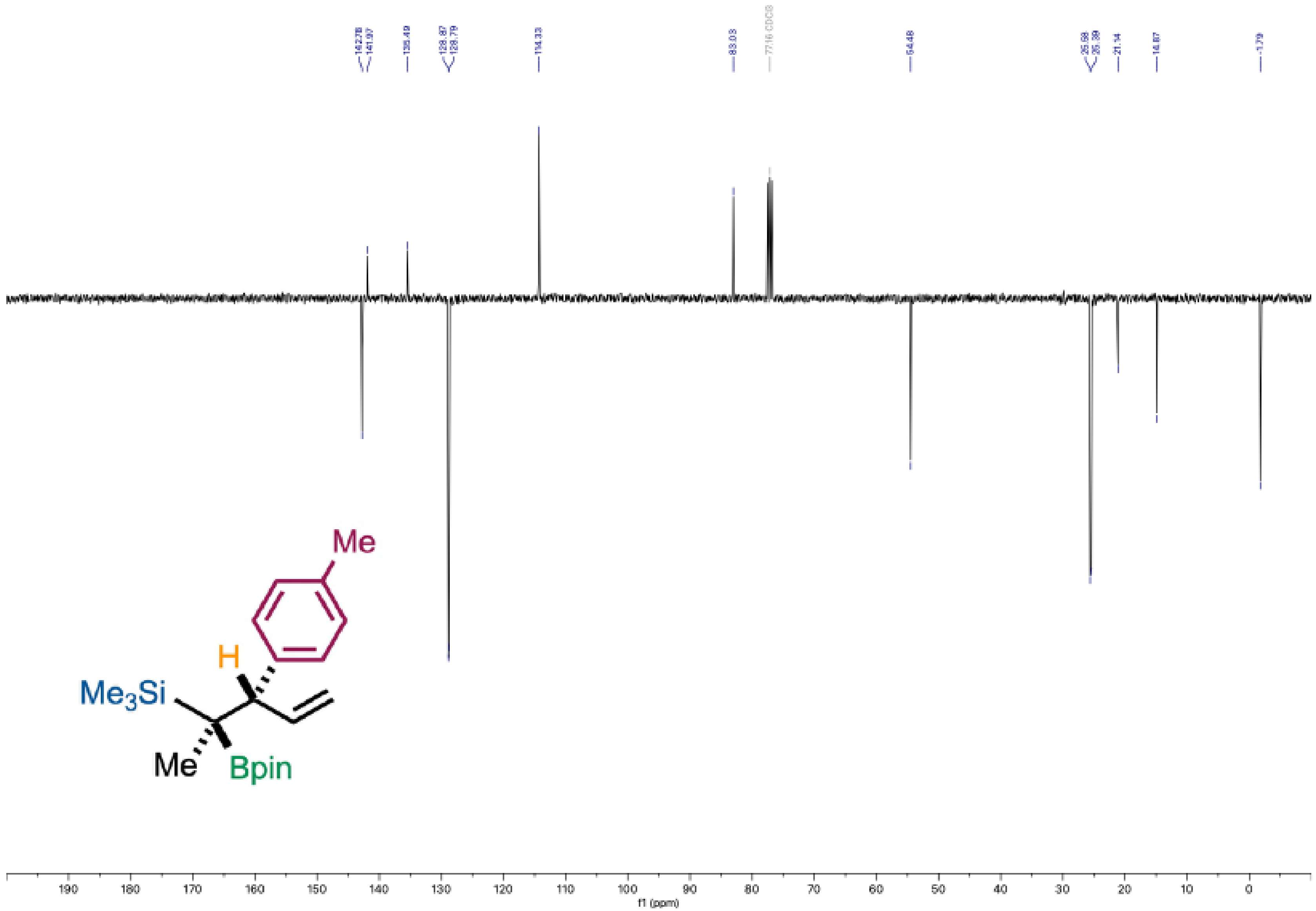

^{13}C NMR spectrum (101 MHz, $CDCl_3$)

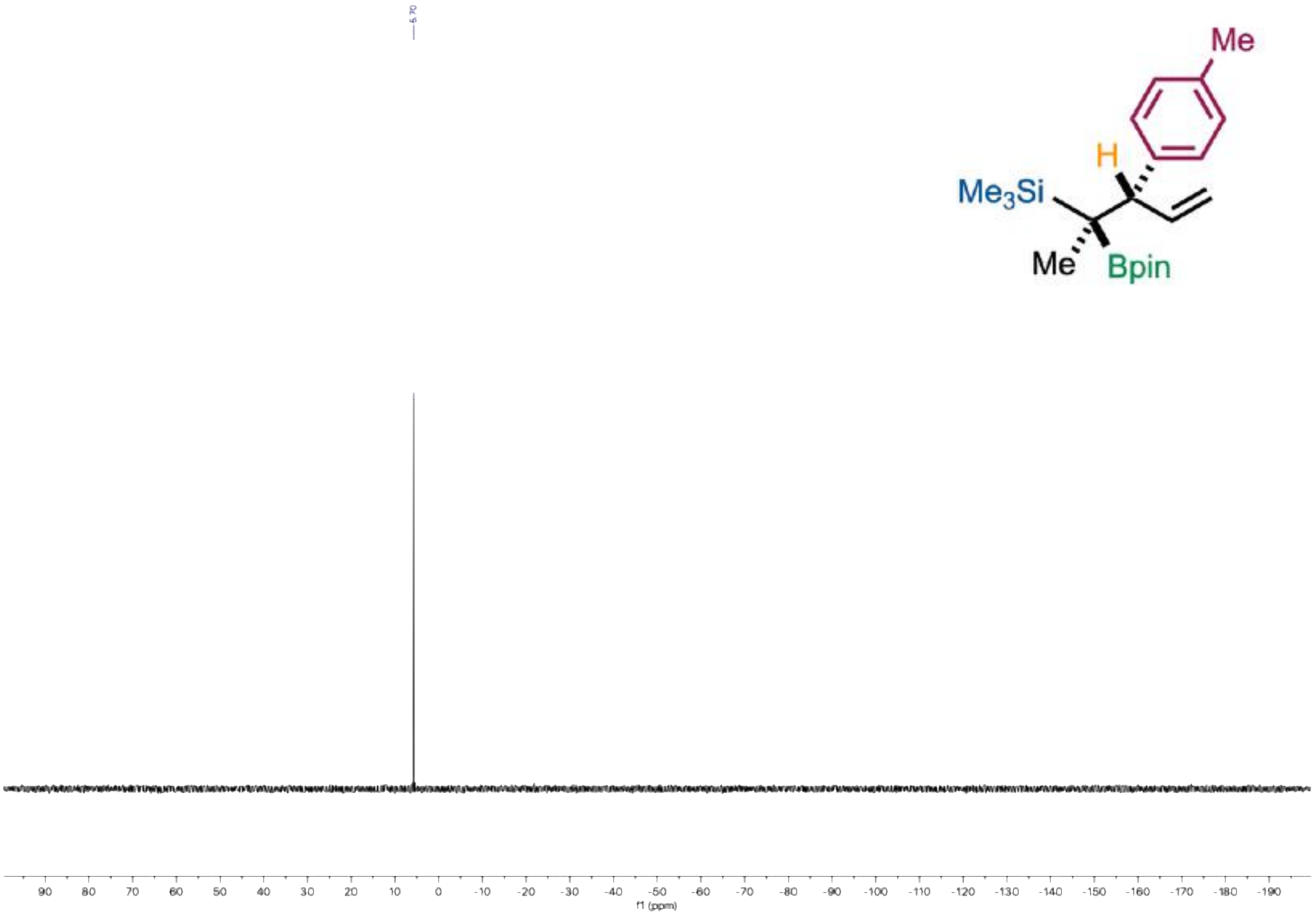

^{29}**Si NMR** spectrum (80 MHz, $CDCl_3$)

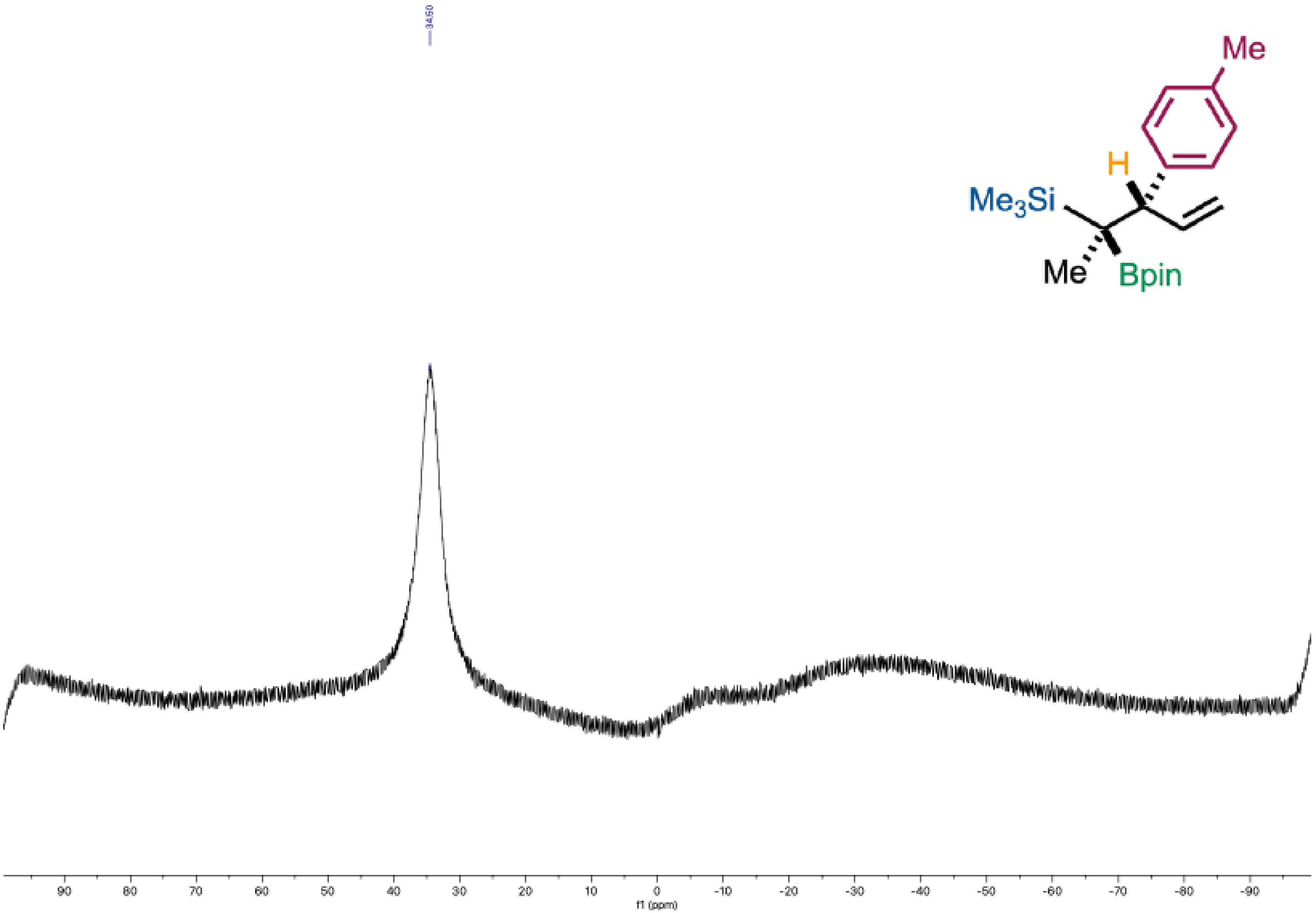

^{11}B NMR spectrum (128 MHz, $CDCl_3$)

Dimethyl(phenyl)((2*S,3*S**)-2-(4,4,5,5-tetramethyl-1,3,2-dioxaborolan-2-yl)-3-(*p*-tolyl)pent-4-en-2-yl)silane 5e**

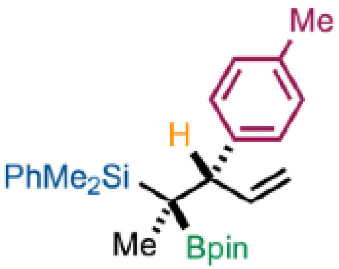

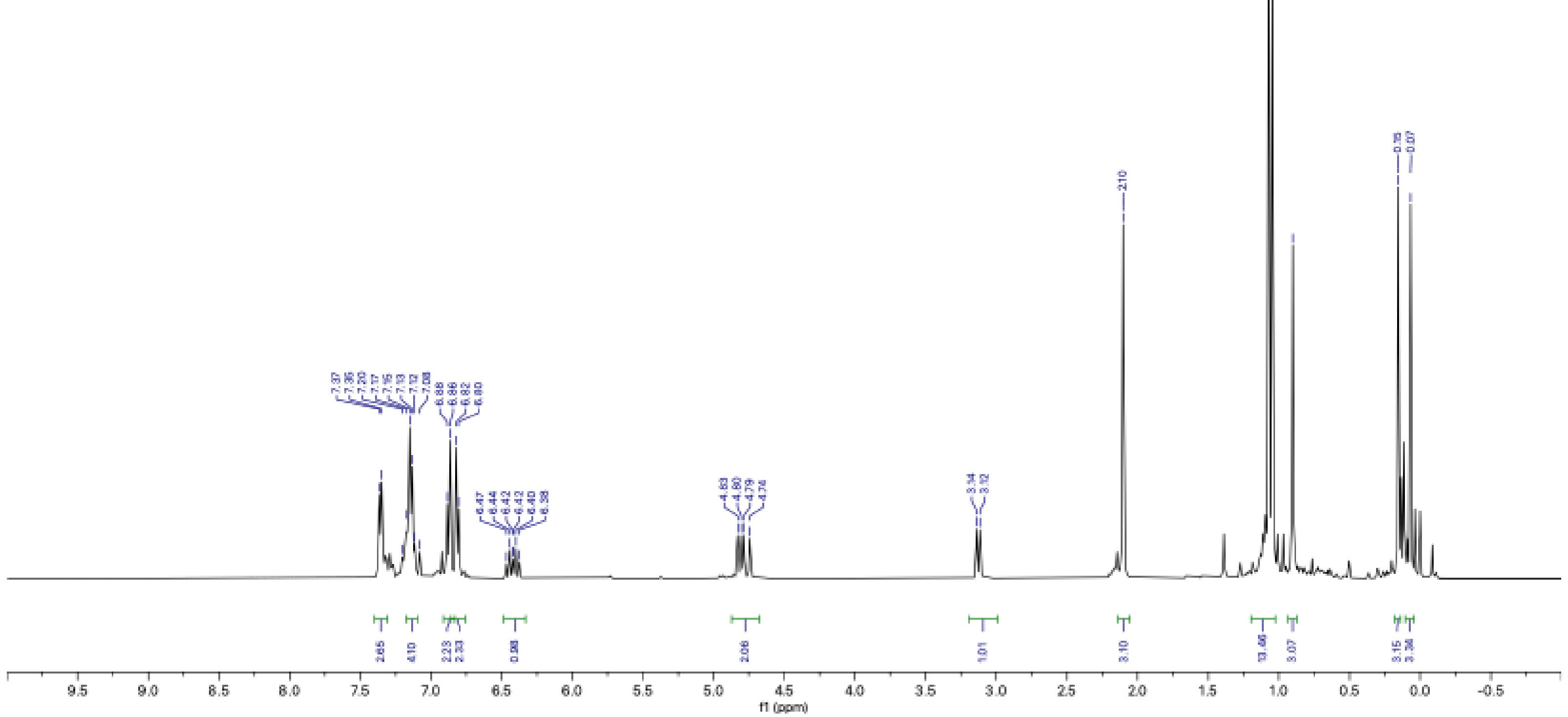

¹H NMR spectrum (400 MHz, $CDCl_3$)

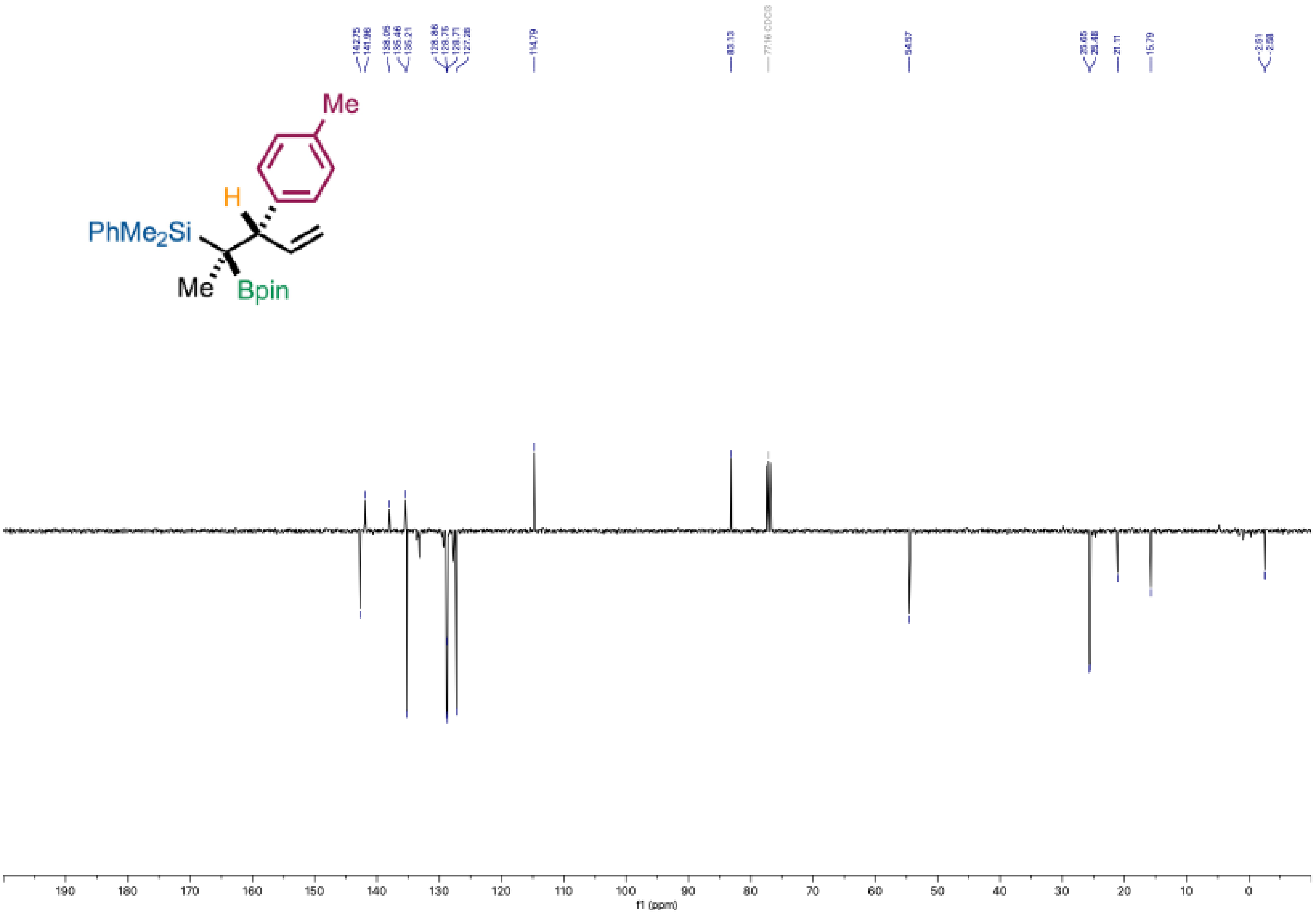

^{13}C NMR spectrum (101 MHz, $CDCl_3$)

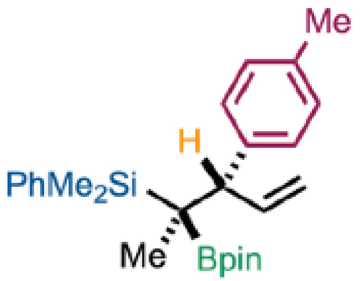

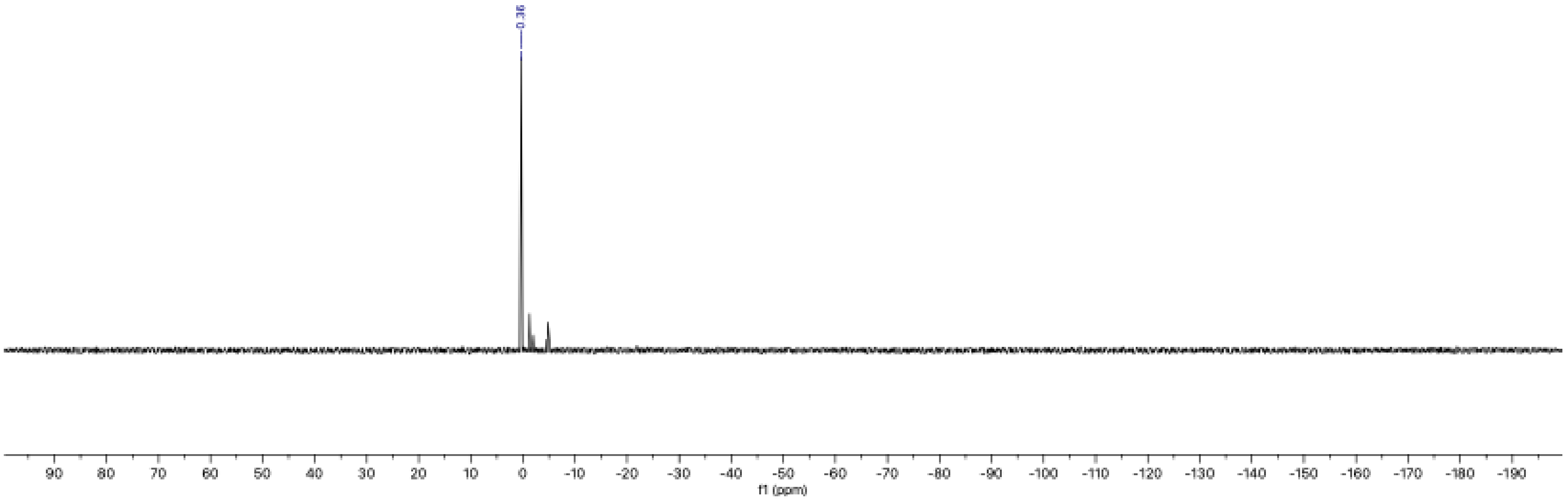

^{29}Si NMR spectrum (80 MHz, $CDCl_3$)

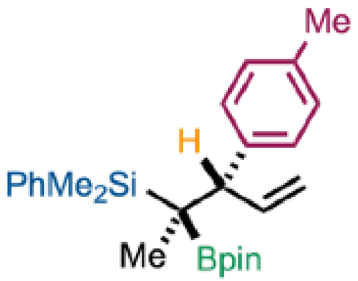

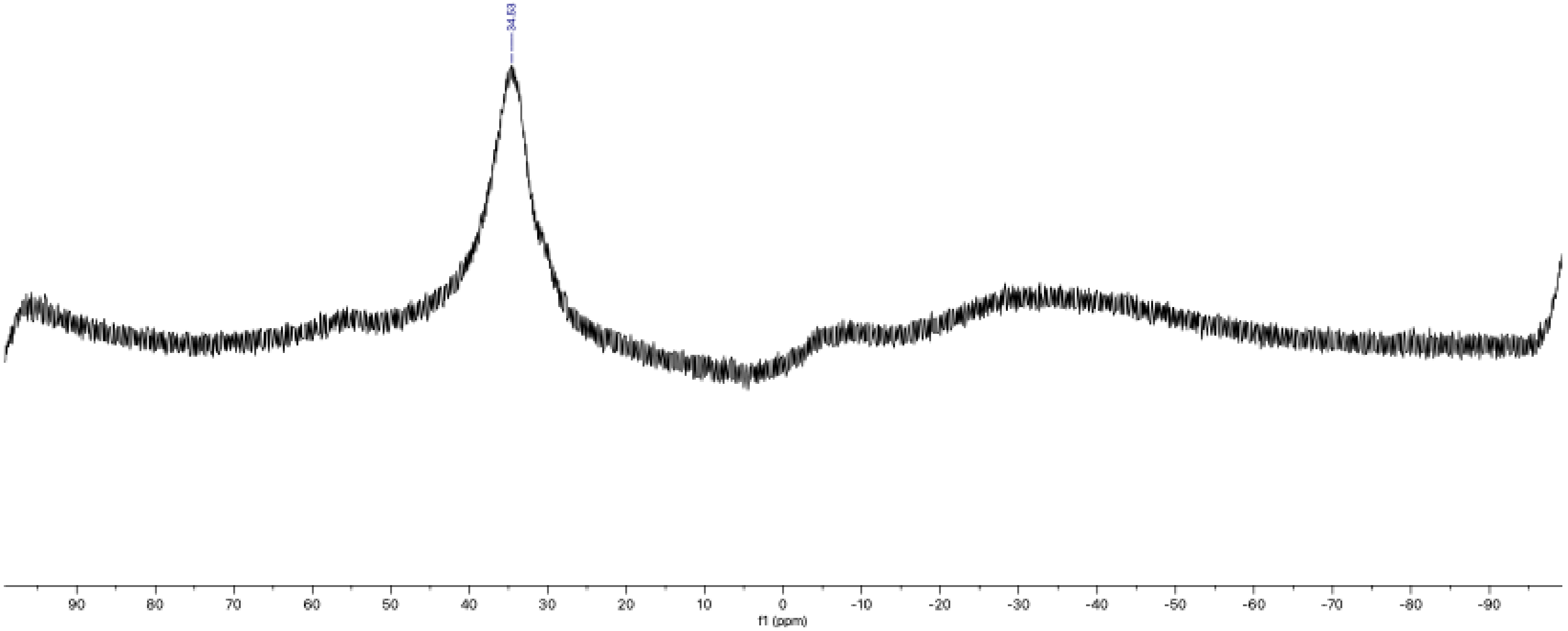

^{11}B NMR spectrum (128 MHz, $CDCl_3$)

Trimethyl((2*S,3*S**)-2-(4,4,5,5-tetramethyl-1,3,2-dioxaborolan-2-yl)-3-(4-(trifluoromethyl)phenyl)pent-4-en-2-yl)silane 5f**

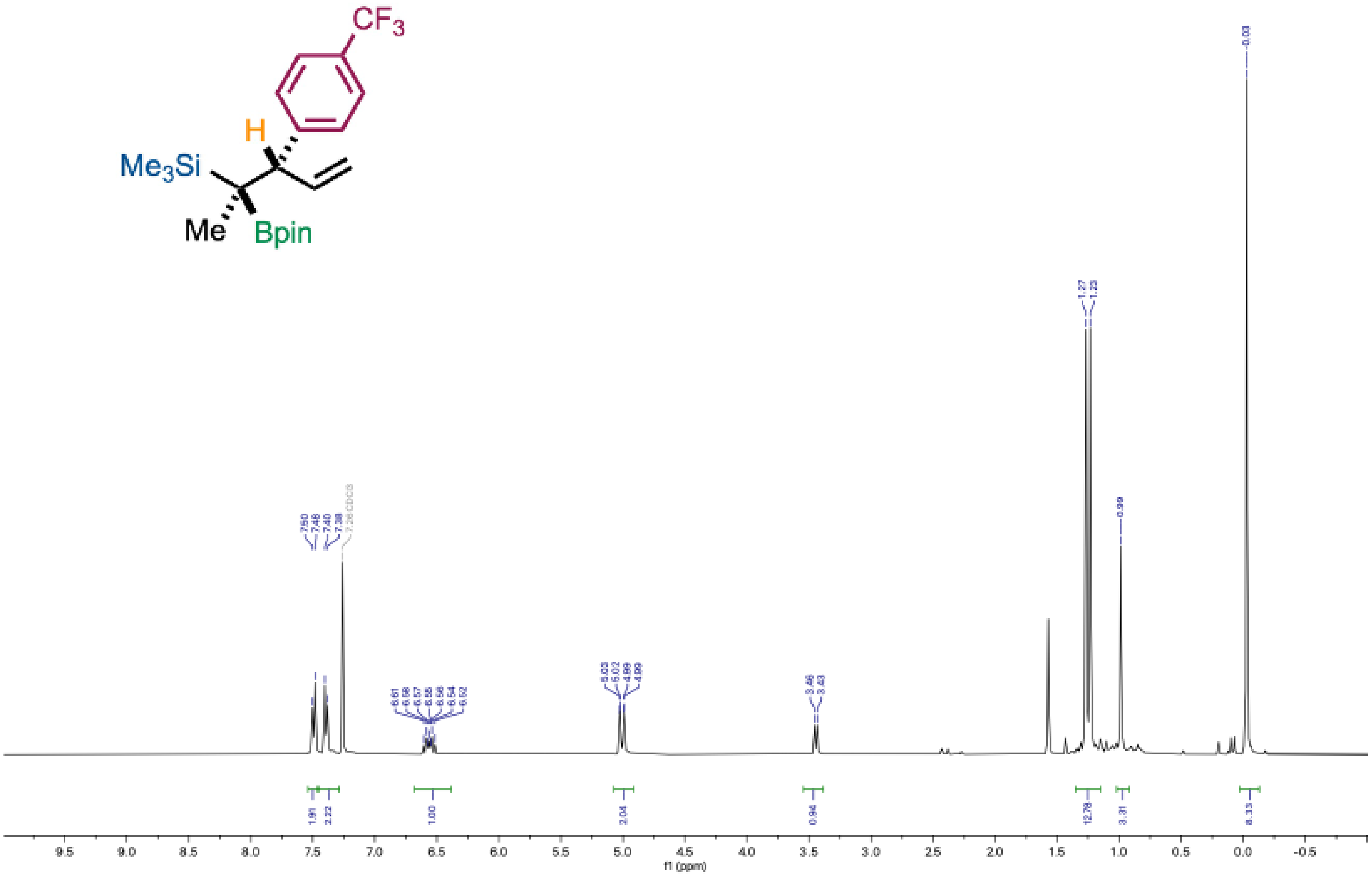

^{1}H NMR spectrum (400 MHz, $CDCl_3$)

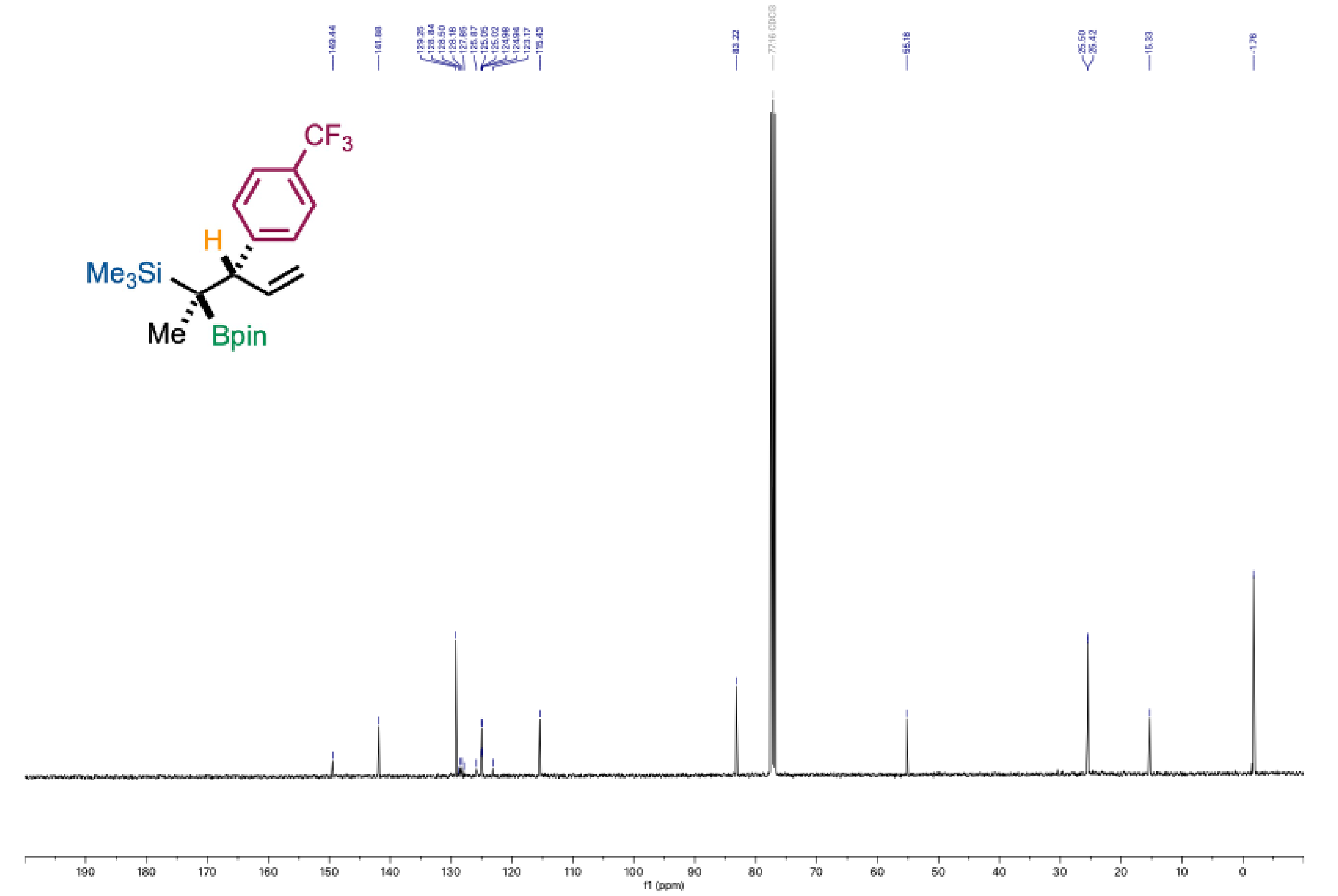

^{13}C NMR spectrum (101 MHz, $CDCl_3$)

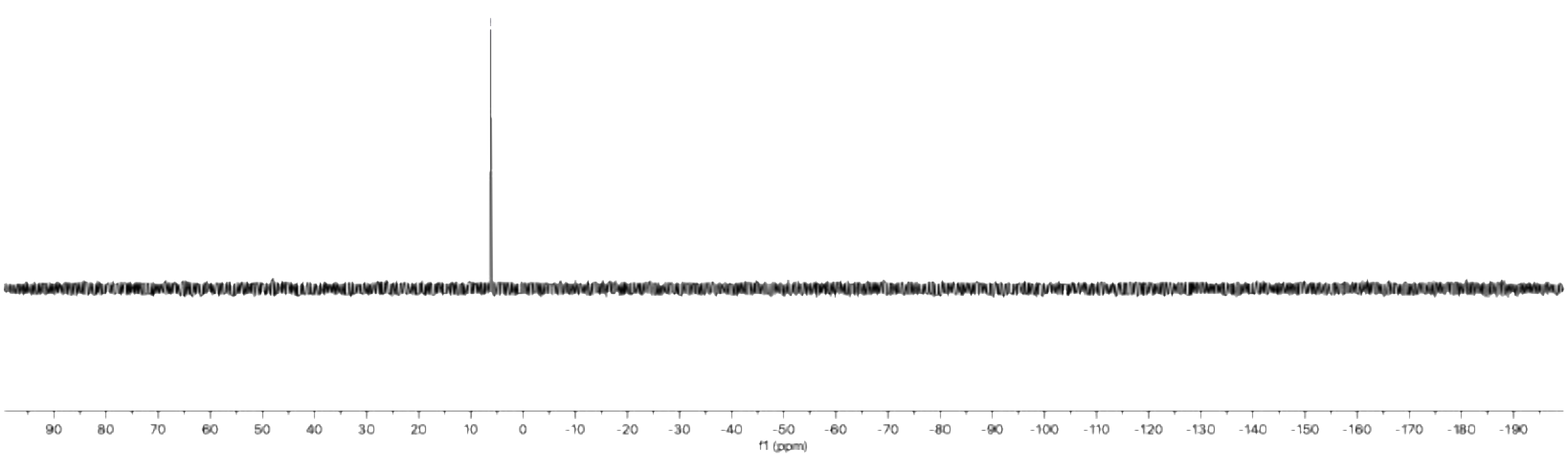

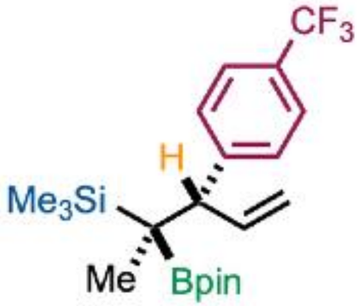

^{29}**Si NMR** spectrum (80 MHz, $CDCl_3$)

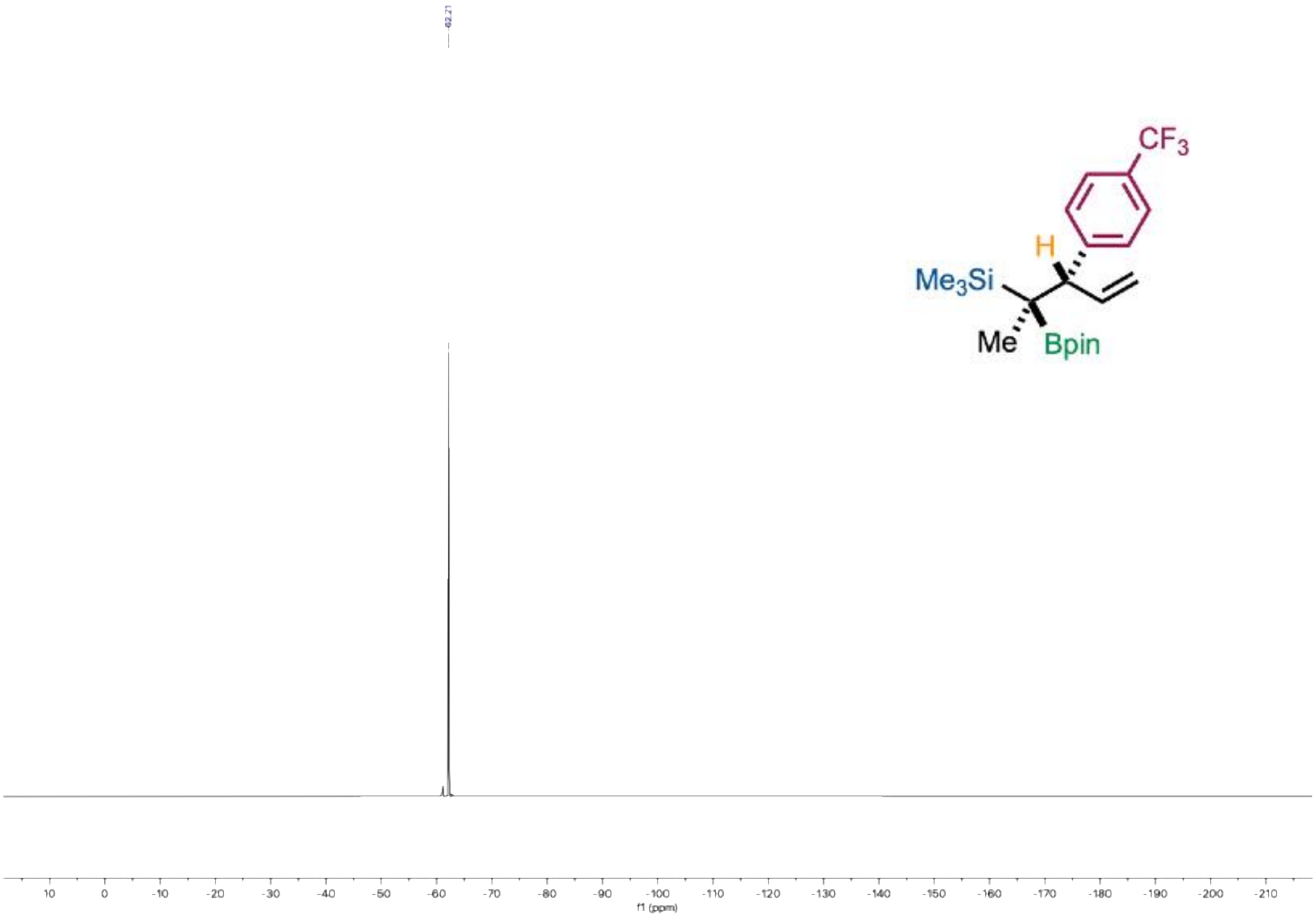

^{19}F NMR spectrum (377 MHz, $CDCl_3$)

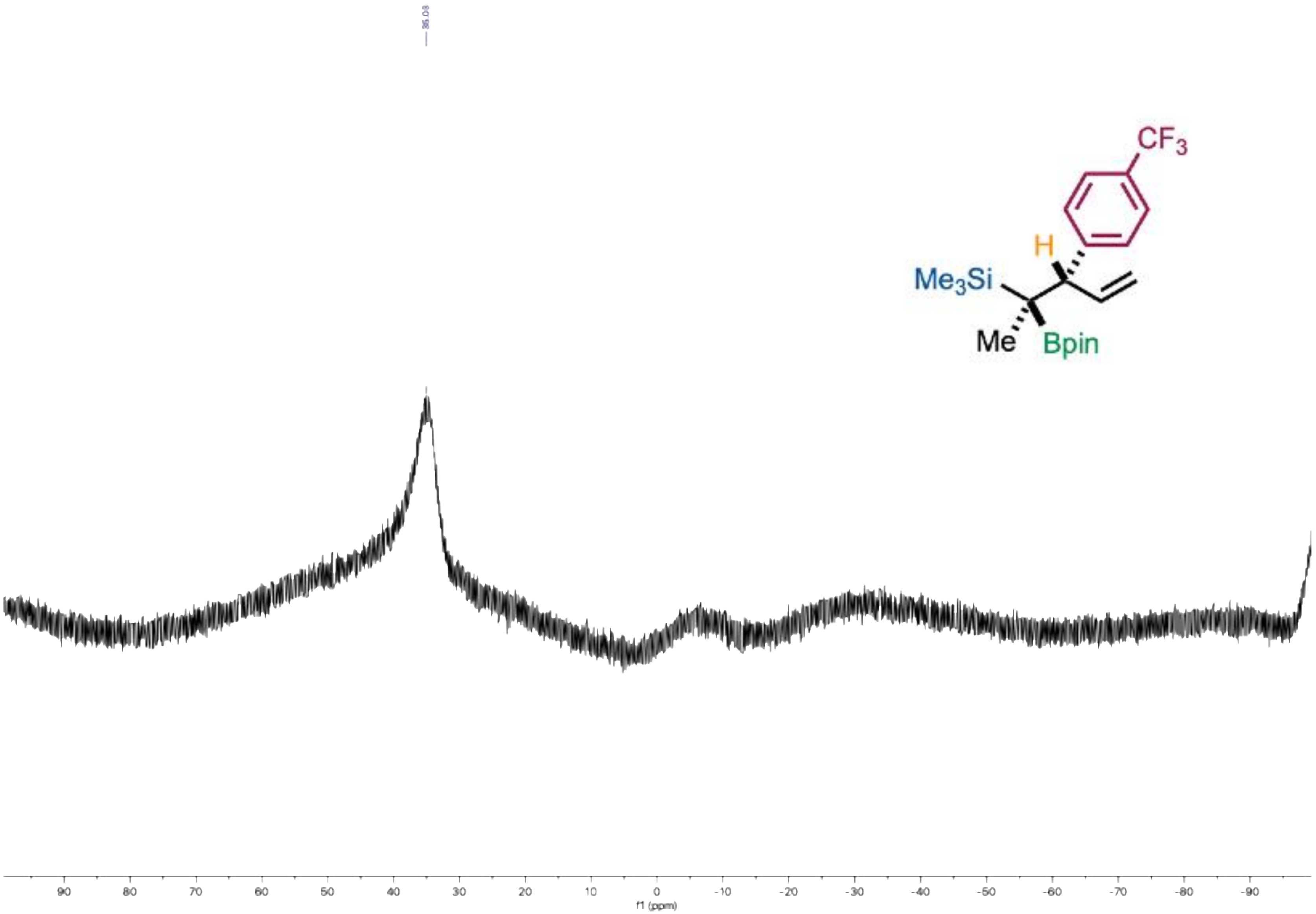

^{11}B NMR spectrum (128 MHz, $CDCl_3$)

((2*S,3*S**)-3-(3-Chlorophenyl)-2-(4,4,5,5-tetramethyl-1,3,2-dioxaborolan-2-yl)pent-4-en-2-yl)trimethylsilane 5g**

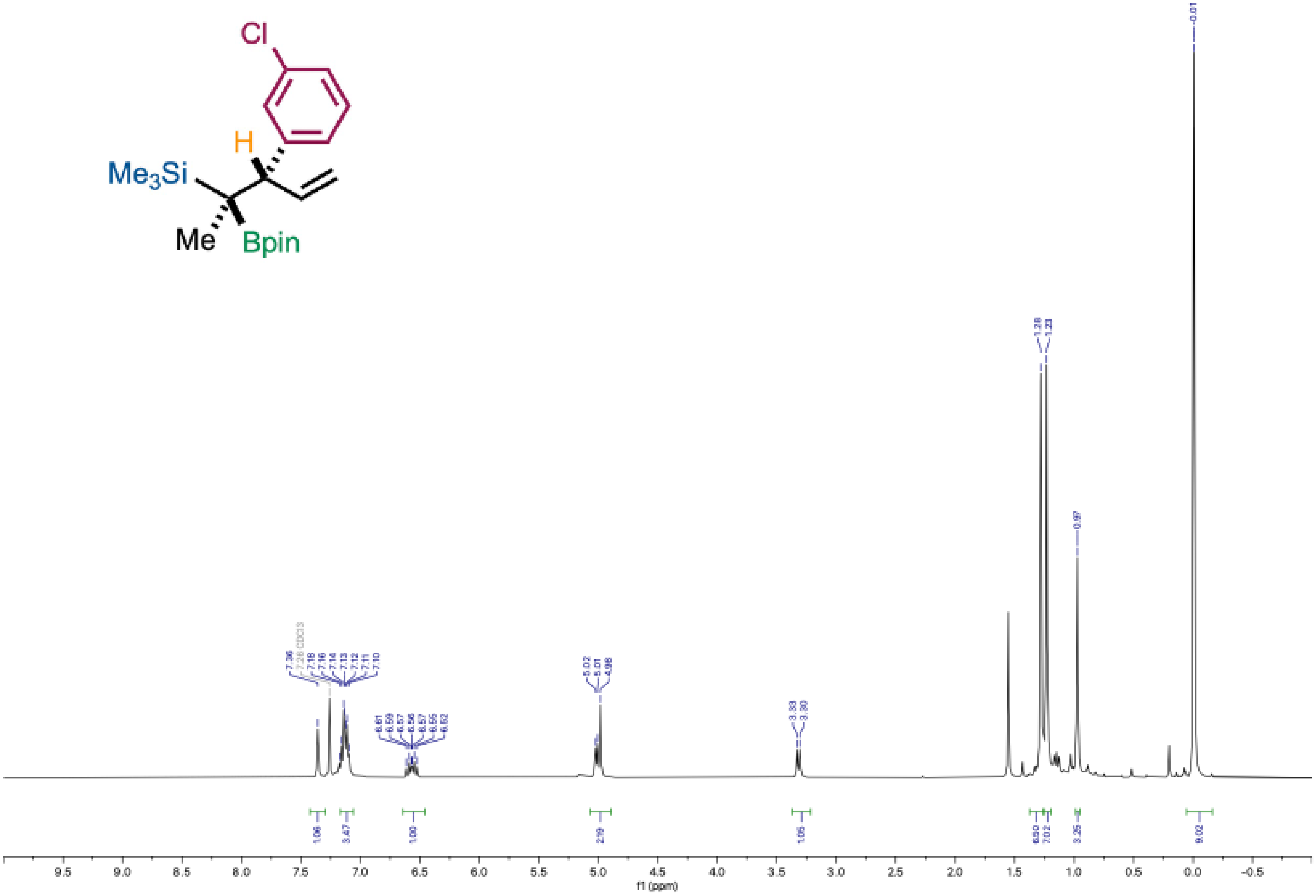

^{1}H NMR spectrum (400 MHz, $CDCl_3$)

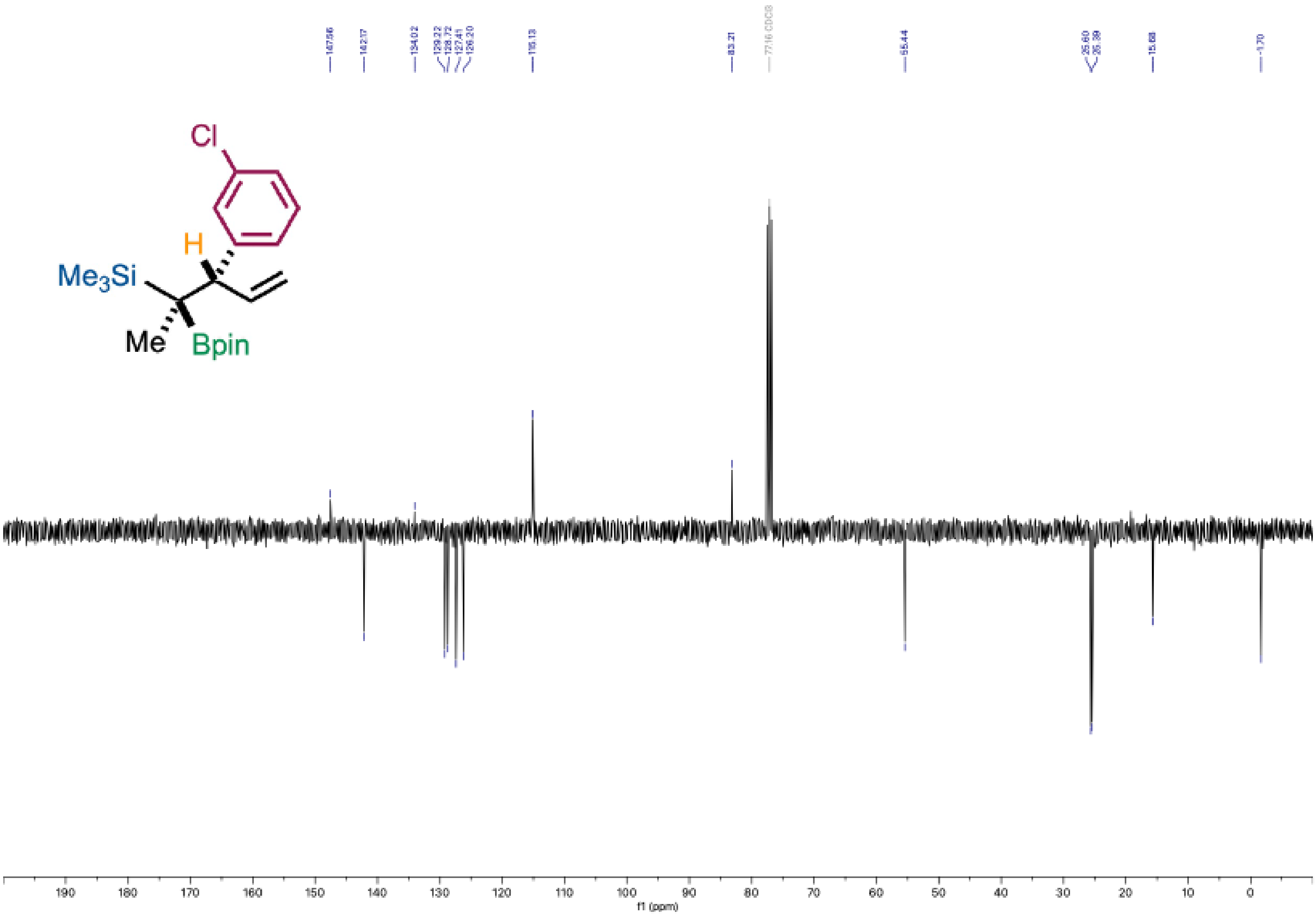

^{13}C NMR spectrum (101 MHz, $CDCl_3$)

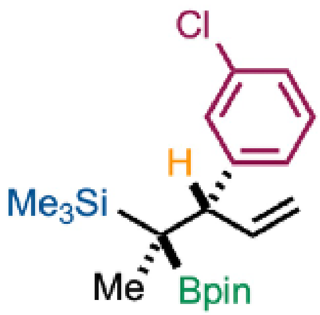

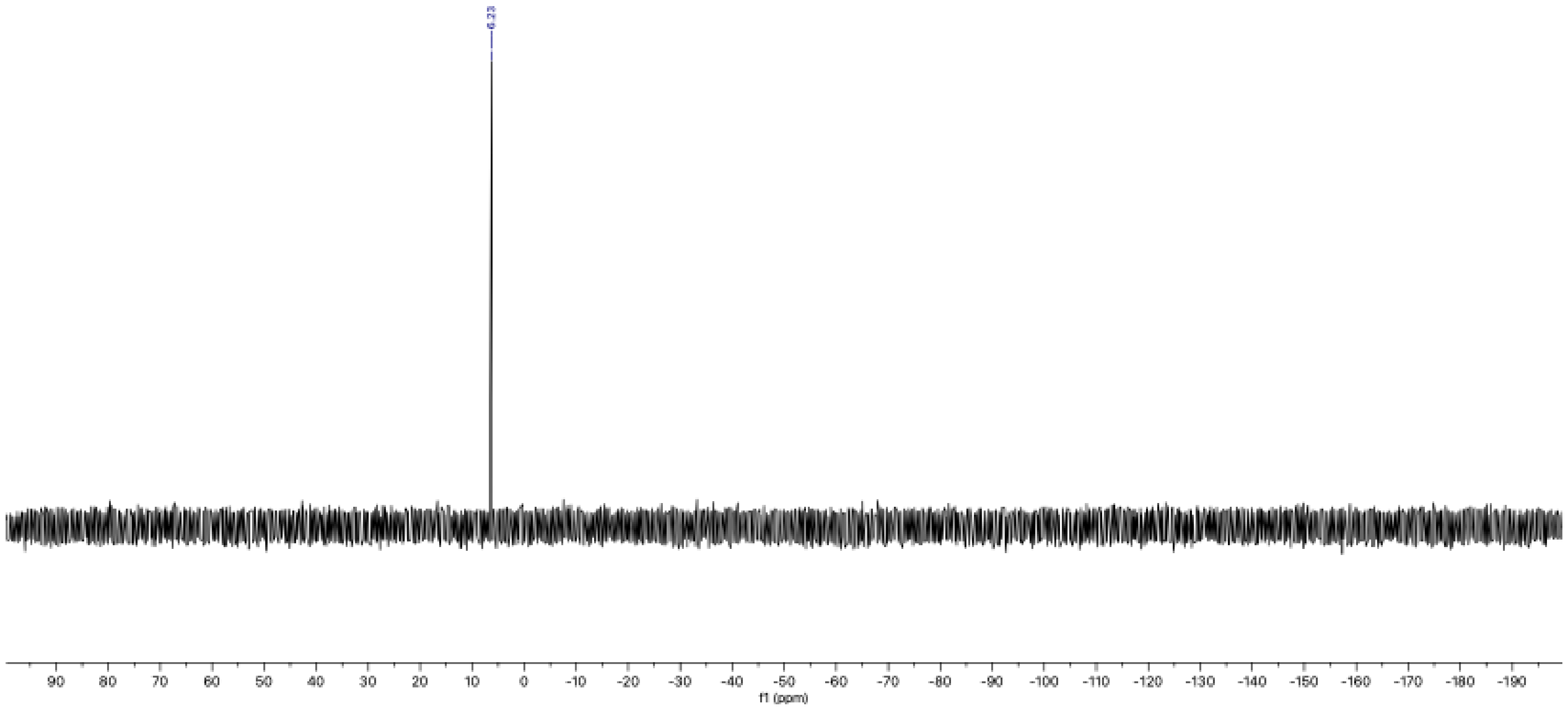

^{29}Si NMR spectrum (80 MHz, $CDCl_3$)

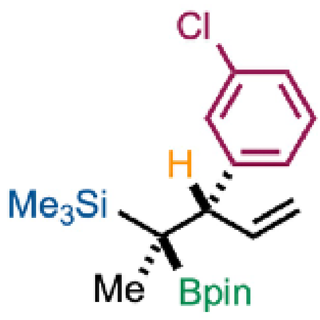

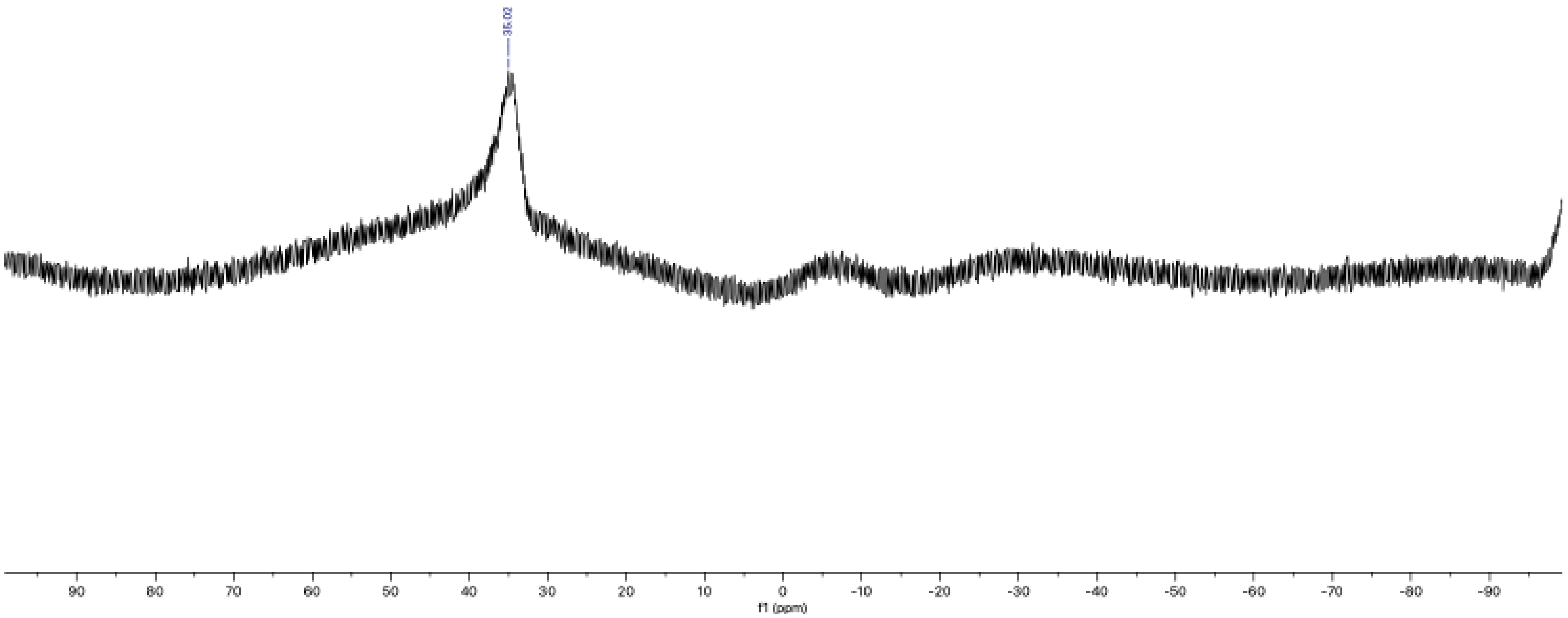

^{11}B NMR spectrum (128 MHz, $CDCl_3$)

Trimethyl((2*S,3*S**)-3-(naphthalen-1-yl)-2-(4,4,5,5-tetramethyl-1,3,2-dioxaborolan-2-yl)pent-4-en-2-yl)silane 5h**

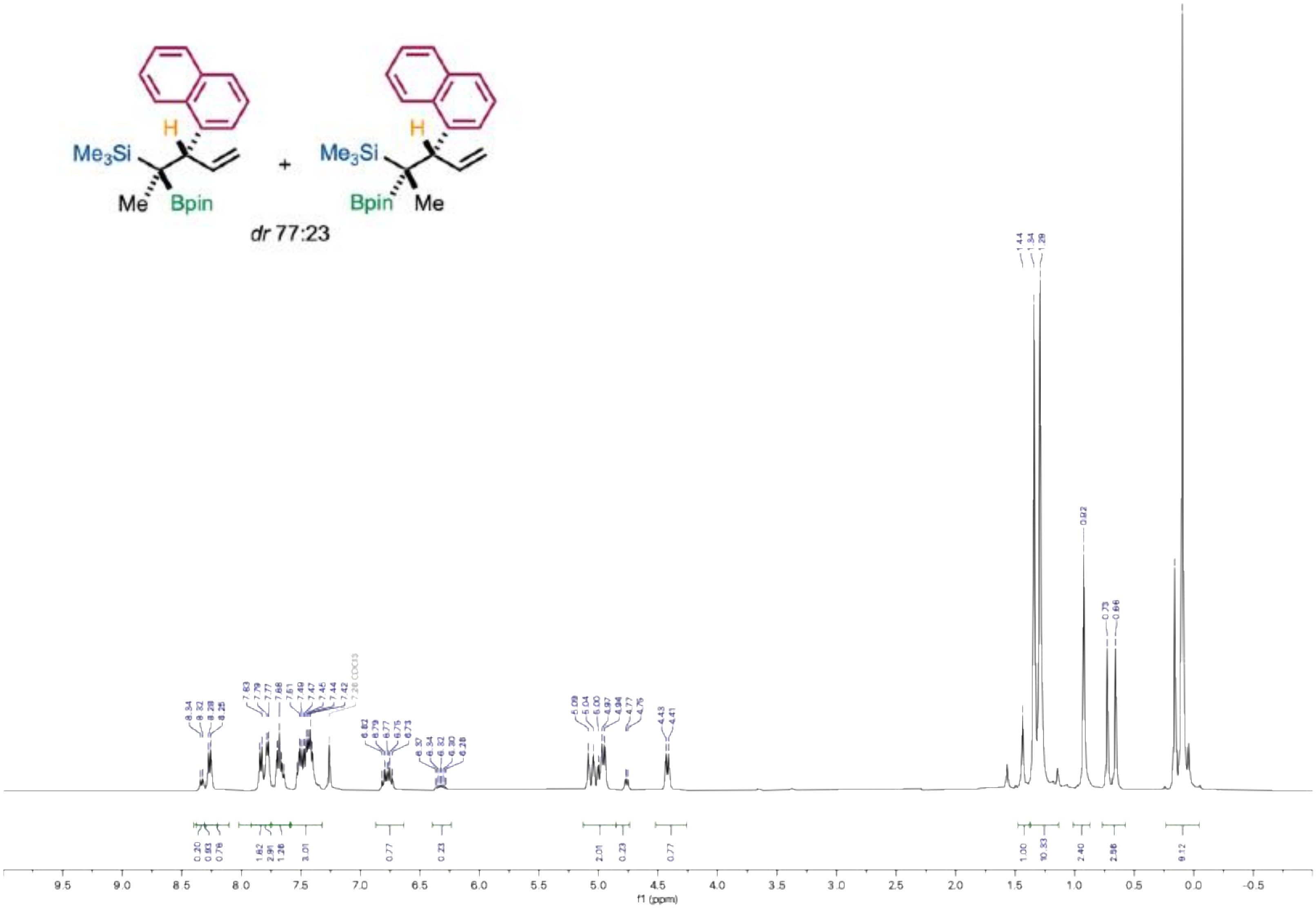

^{1}H NMR spectrum (400 MHz, $CDCl_3$)

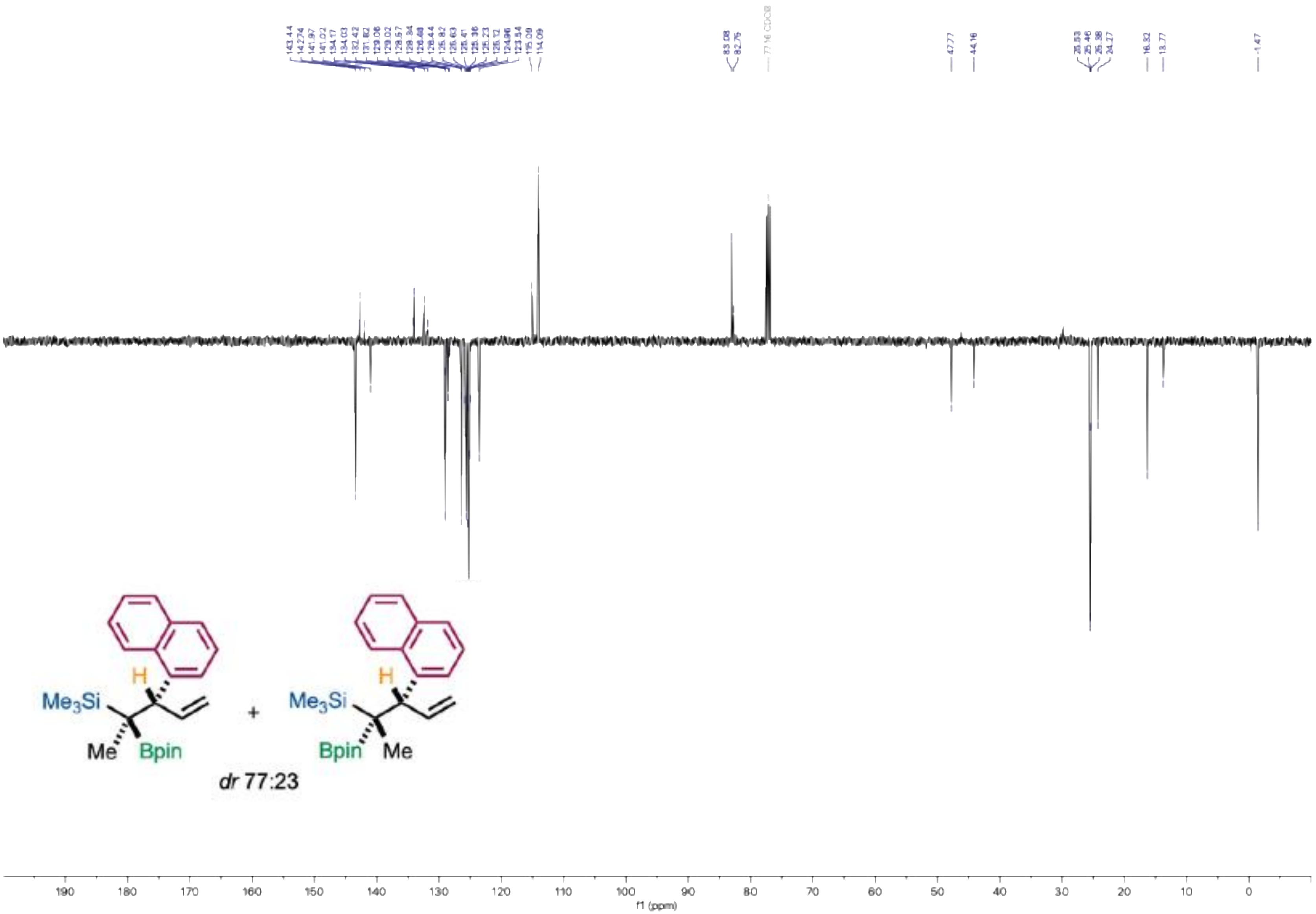

^{13}C NMR spectrum (101 MHz, $CDCl_3$)

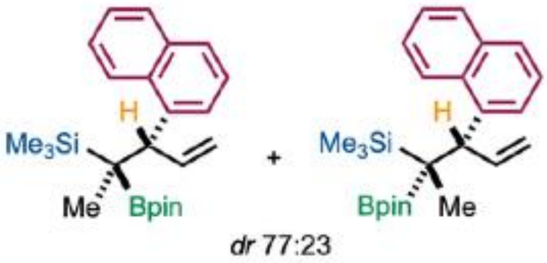

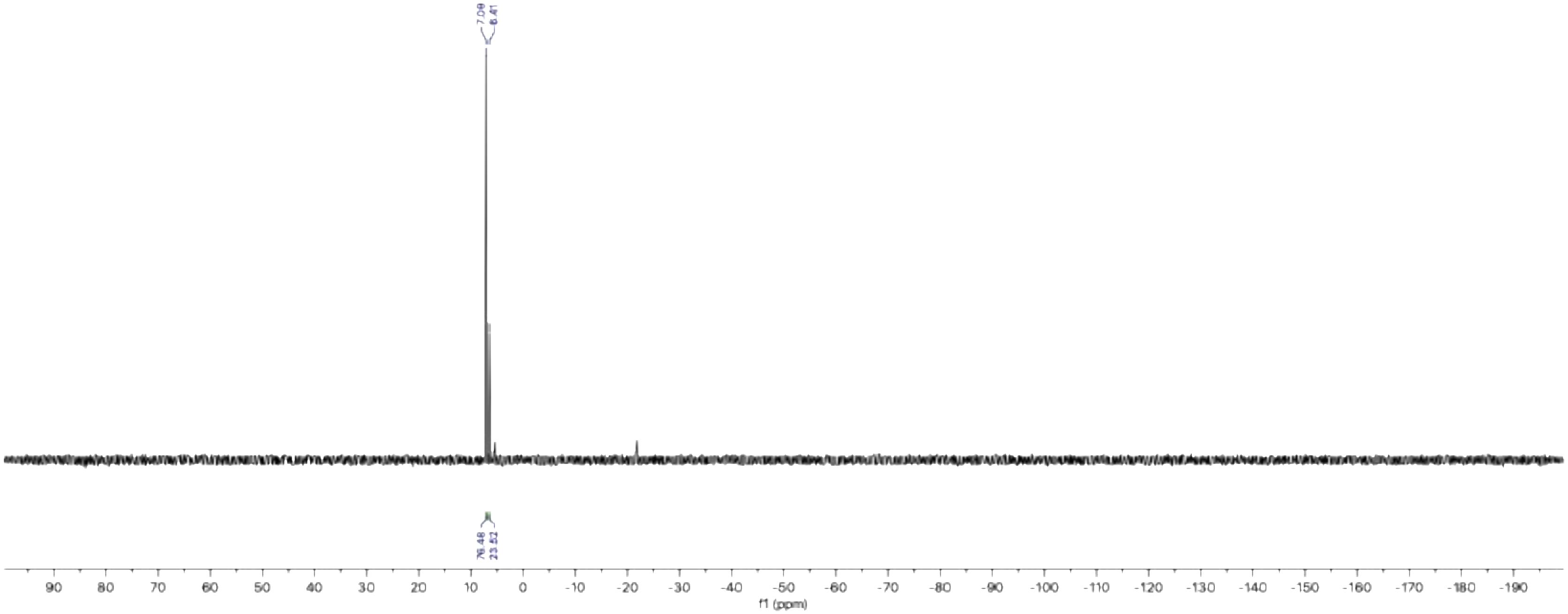

^{29}Si NMR spectrum (80 MHz, $CDCl_3$)

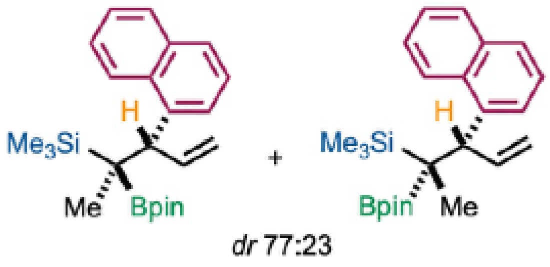

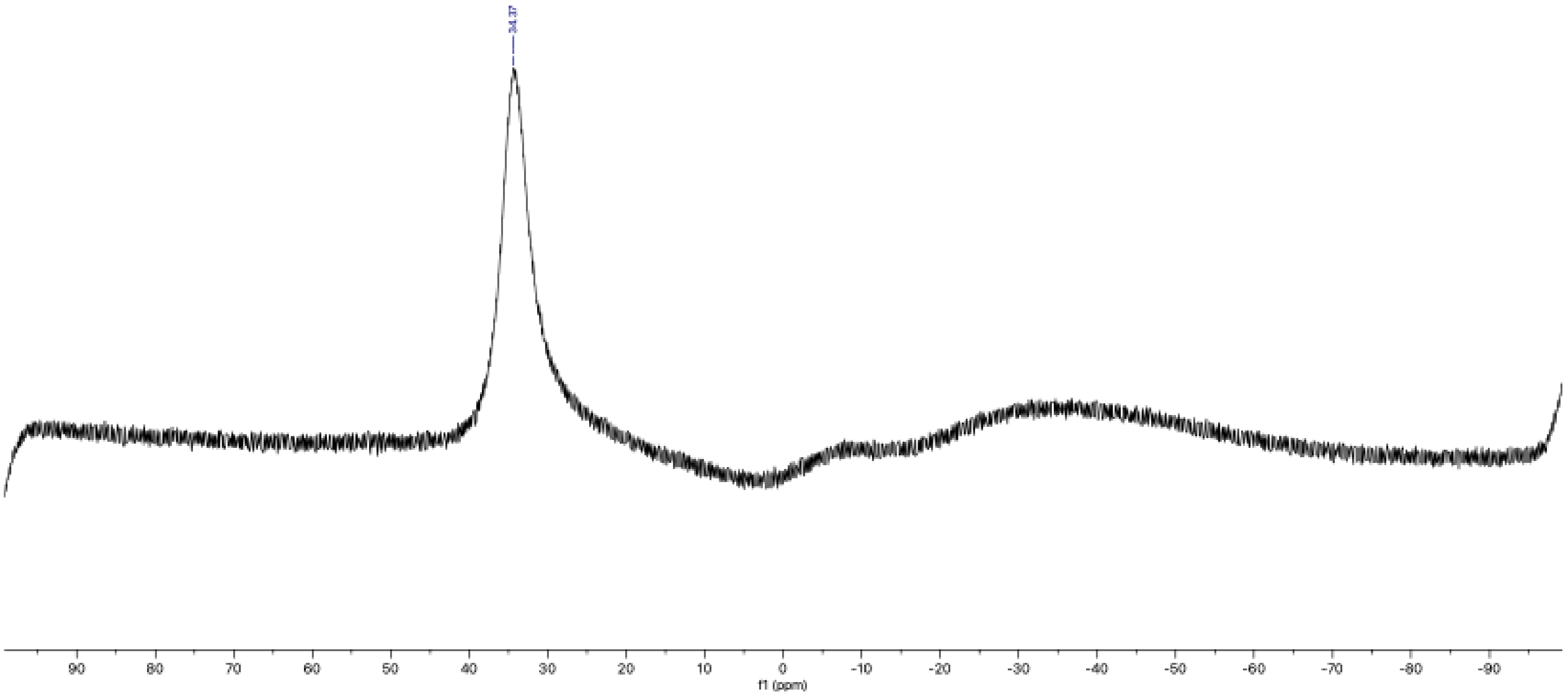

^{11}**B NMR** spectrum (128 MHz, $CDCl_3$)

((3*S,4*S**)-3-(4-Methoxyphenyl)-4-(4,4,5,5-tetramethyl-1,3,2-dioxaborolan-2-yl)oct-1-en-4-yl)trimethylsilane 5i**

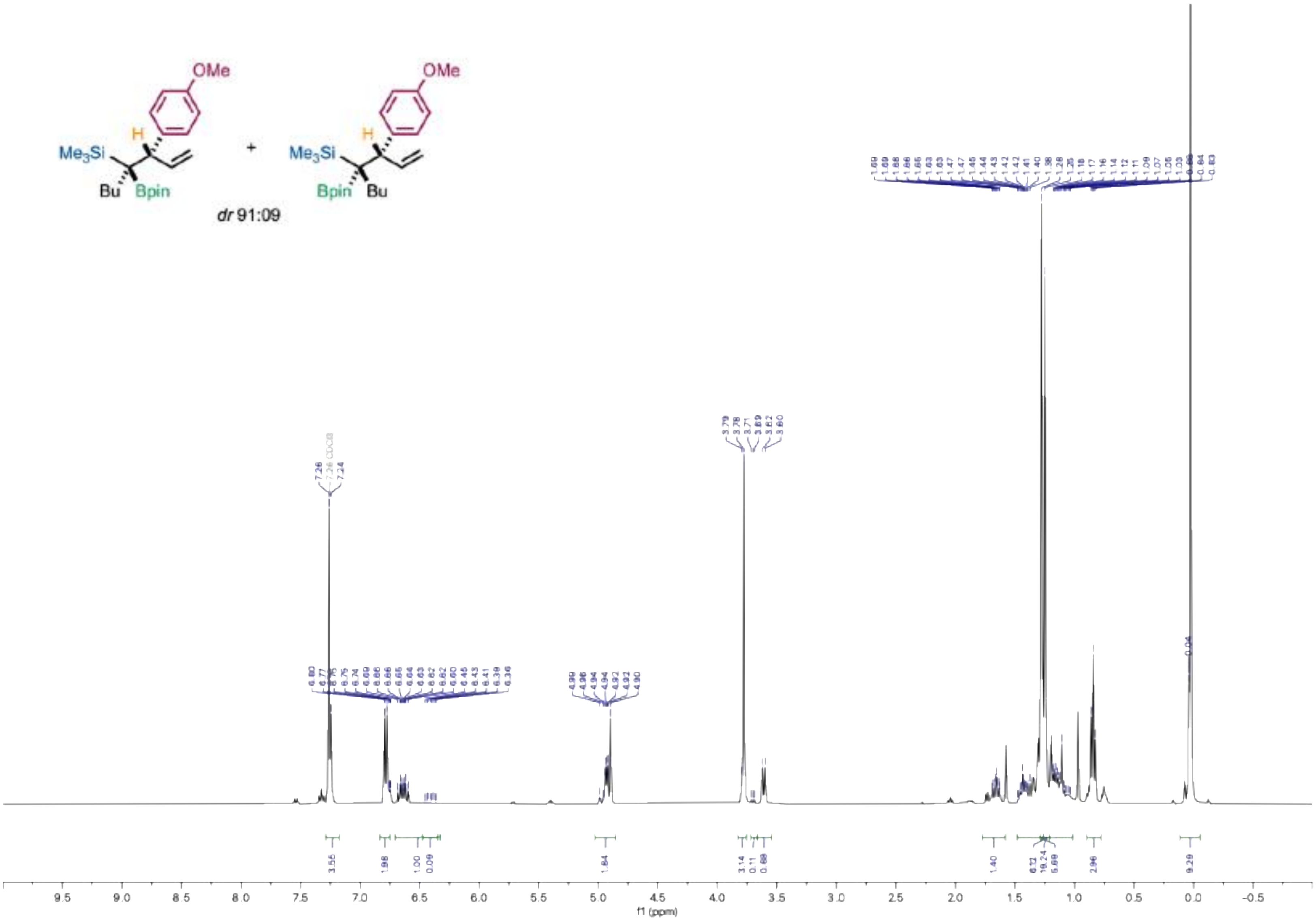

¹H NMR spectrum (400 MHz, $CDCl_3$)

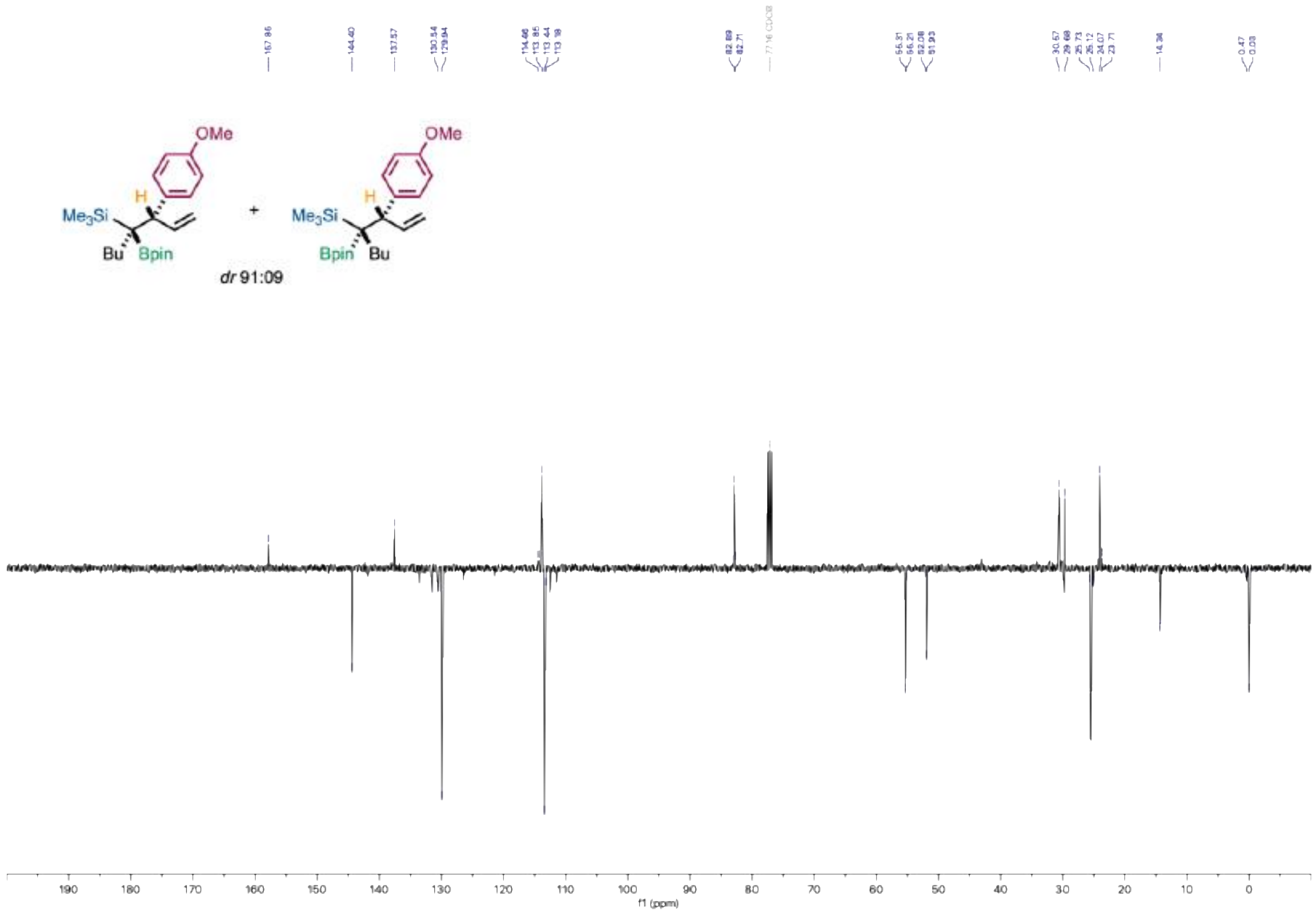

^{13}C NMR spectrum (101 MHz, $CDCl_3$)

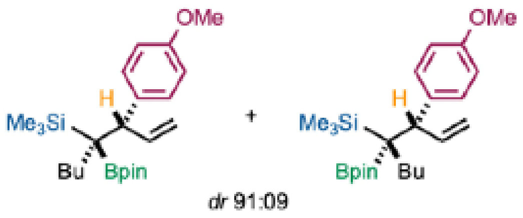

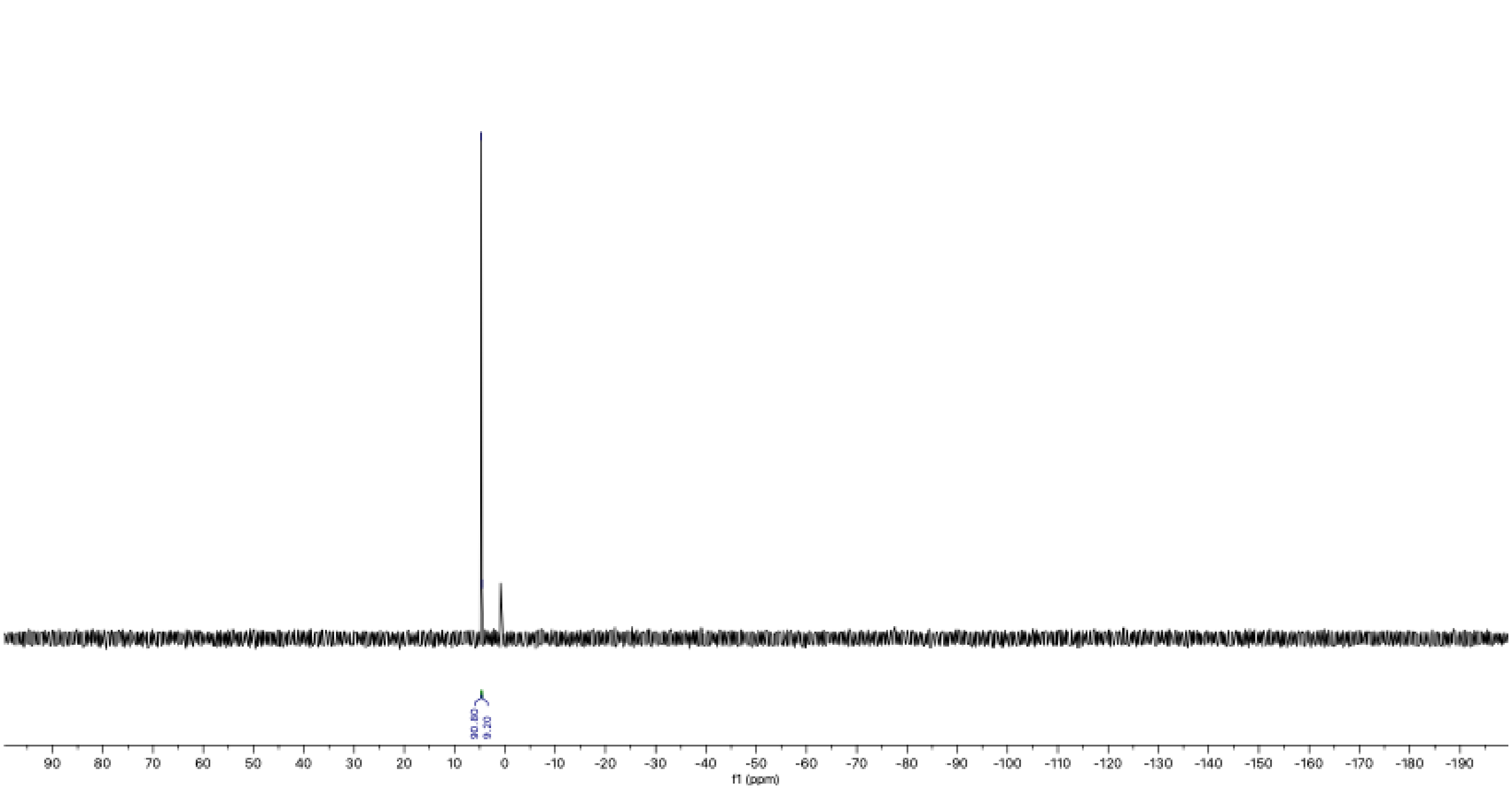

^{29}Si NMR spectrum (80 MHz, $CDCl_3$)

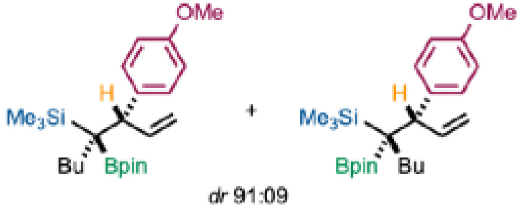

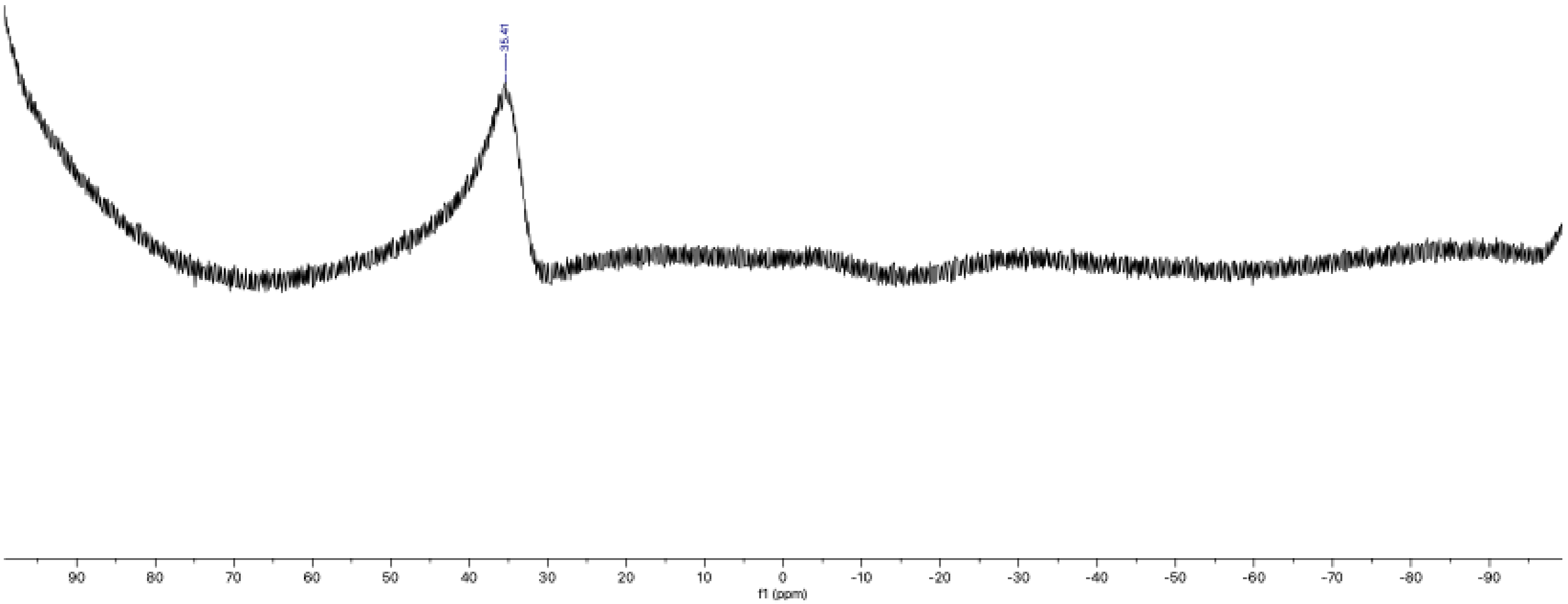

^{11}B NMR spectrum (128 MHz, $CDCl_3$)

((3*S,4*S**)-3-(4-Chlorophenyl)-4-(4,4,5,5-tetramethyl-1,3,2-dioxaborolan-2-yl)oct-1-en-4-yl)trimethylsilane 5j**

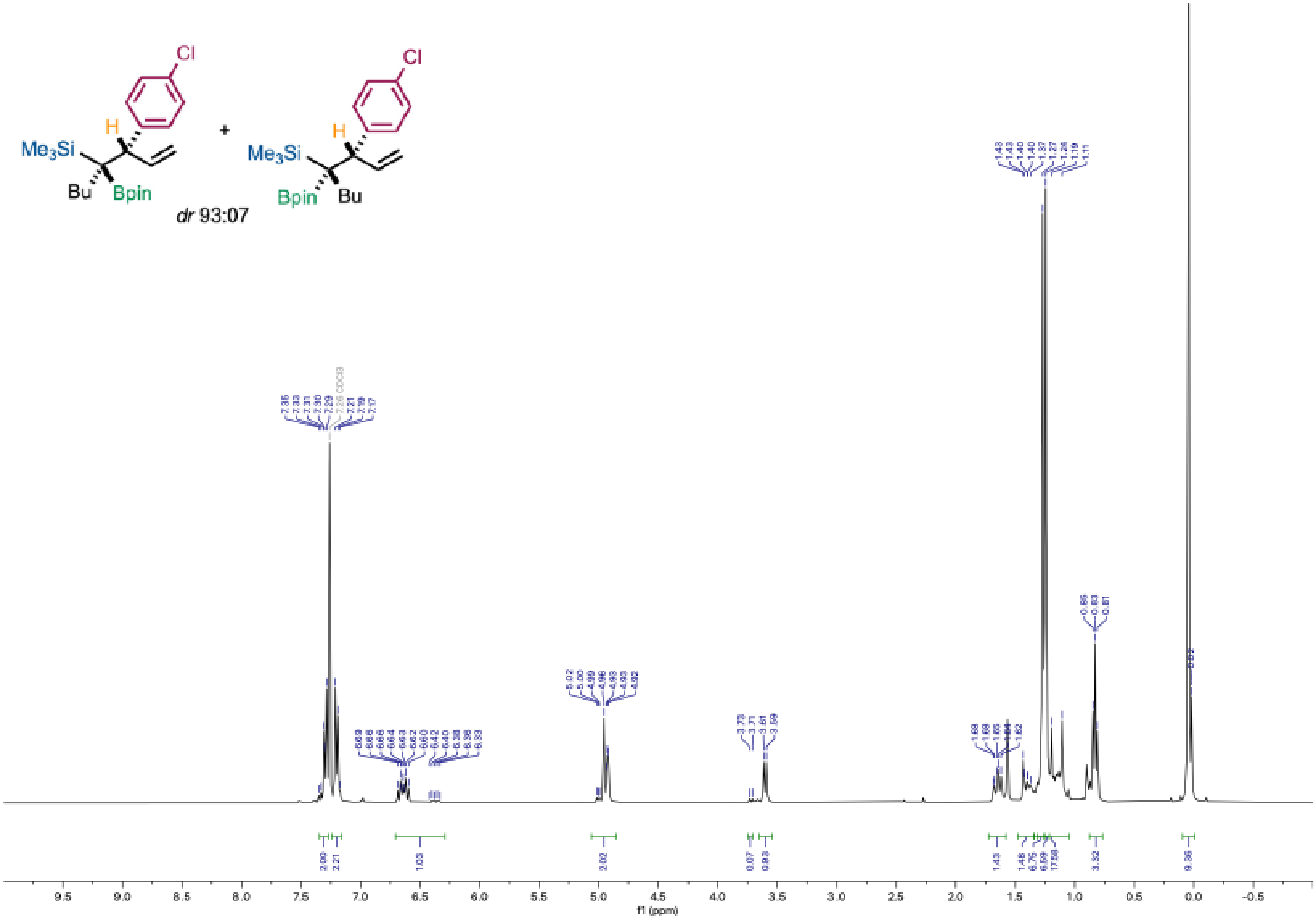

^{1}H NMR spectrum (400 MHz, $CDCl_3$)

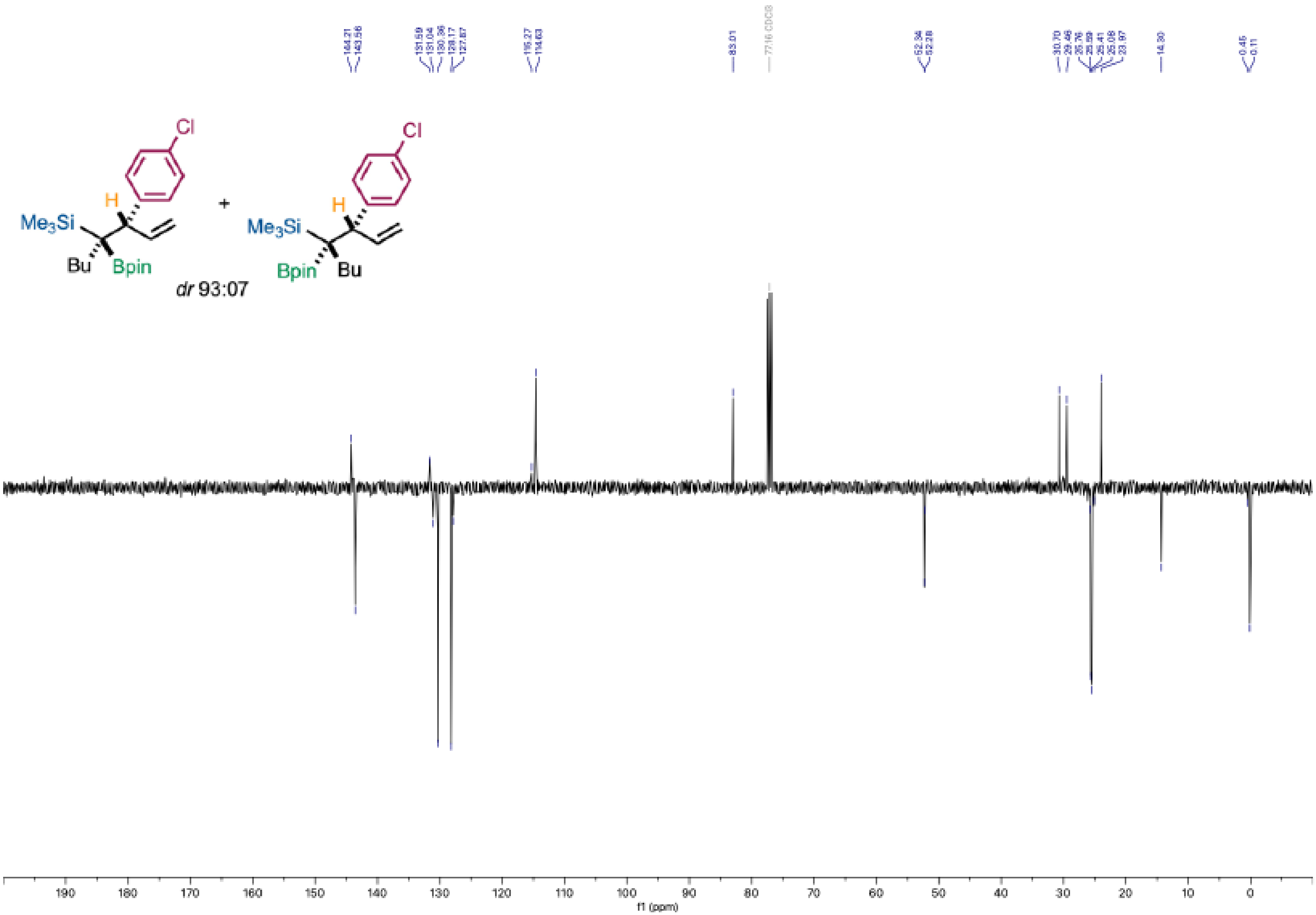

^{13}C NMR spectrum (101 MHz, $CDCl_3$)

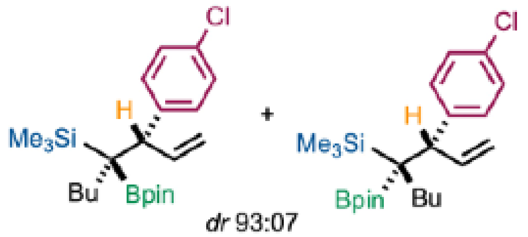

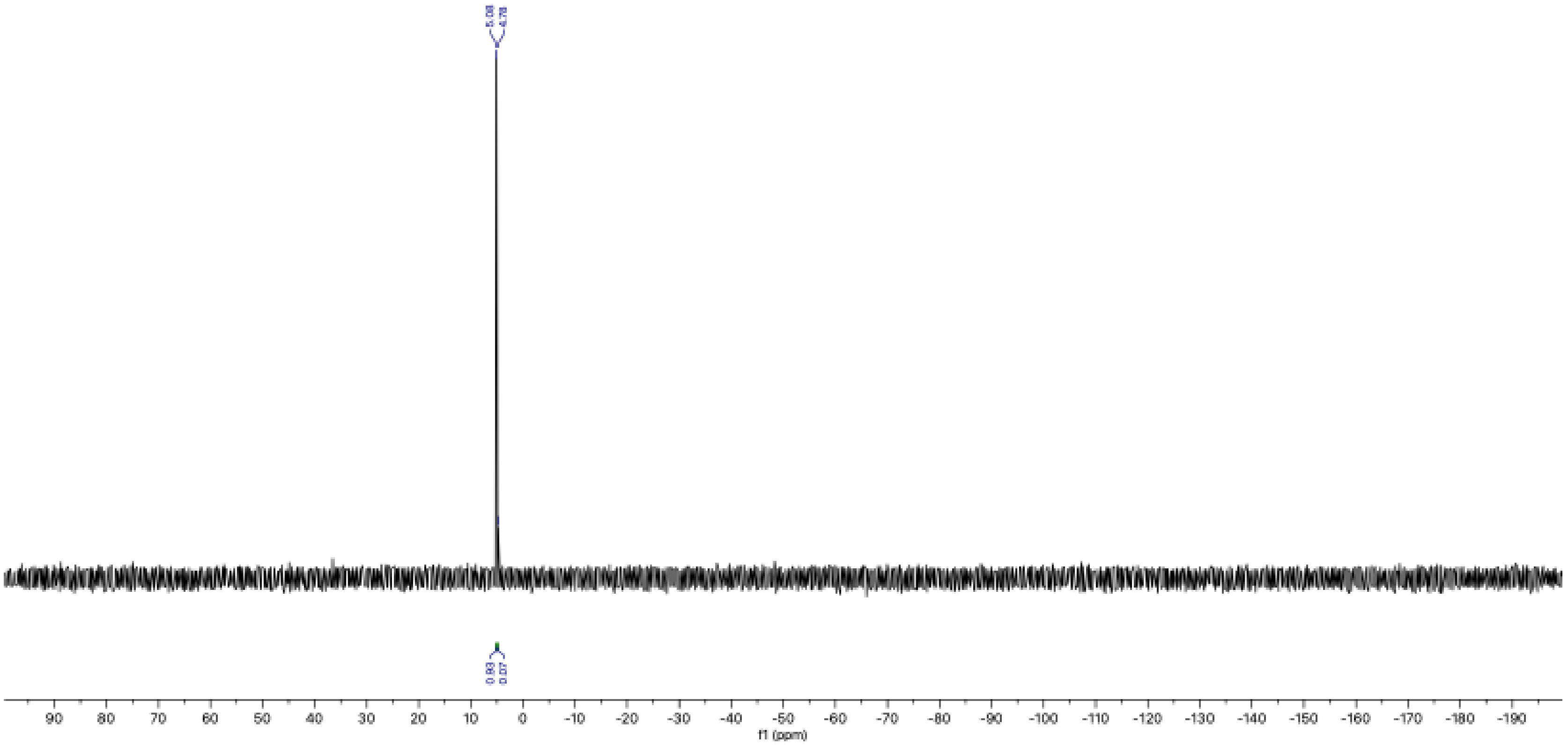

^{29}Si NMR spectrum (80 MHz, $CDCl_3$)

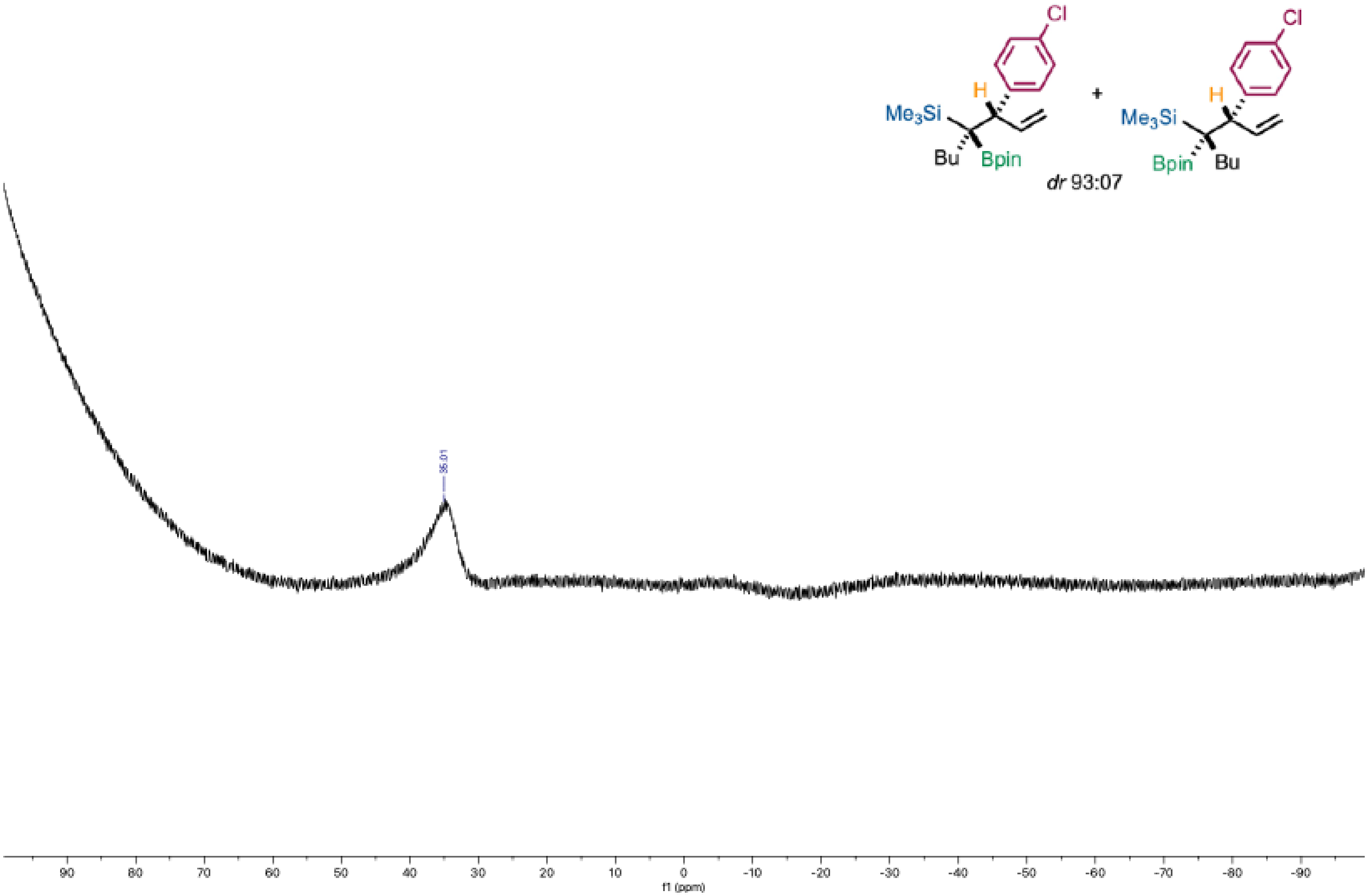

^{11}B NMR spectrum (128 MHz, $CDCl_3$)

((3*S**,4*S**)-7-Chloro-3-phenyl-4-(4,4,5,5-tetramethyl-1,3,2-dioxaborolan-2-yl)hept-1-en-4-yl)trimethylsilane **5k**

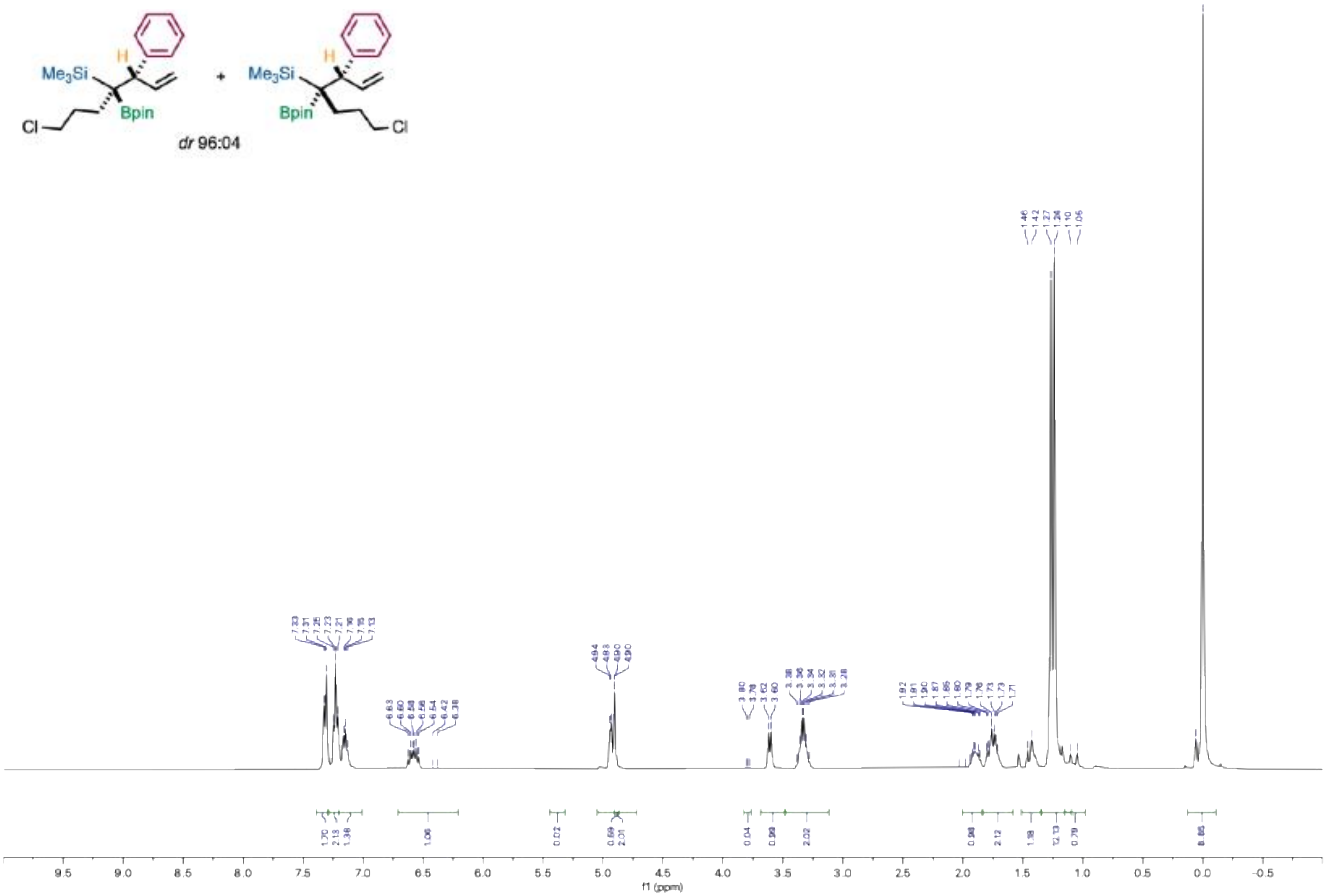

1H NMR spectrum (400 MHz, $CDCl_3$)

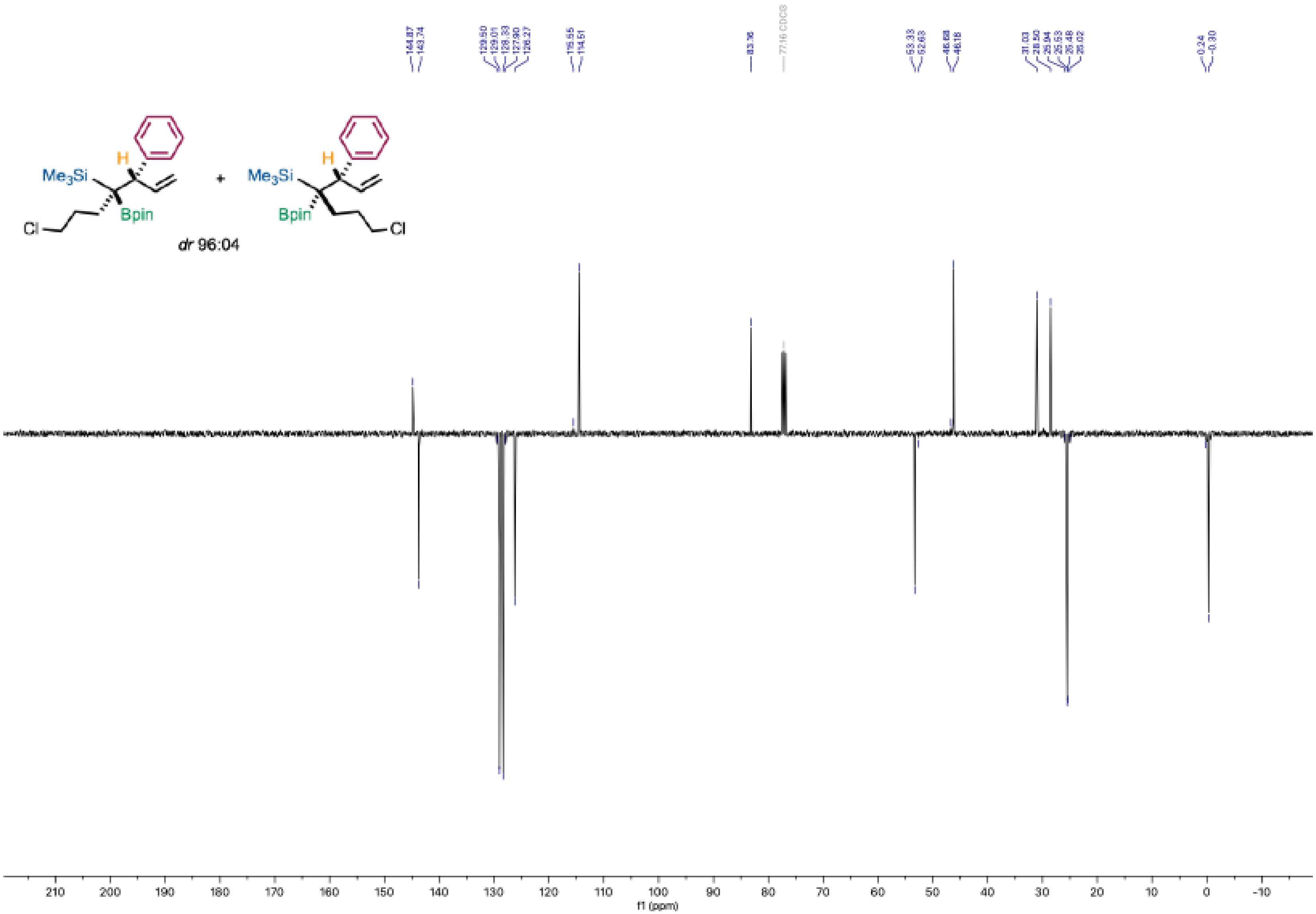

^{13}C NMR spectrum (101 MHz, $CDCl_3$)

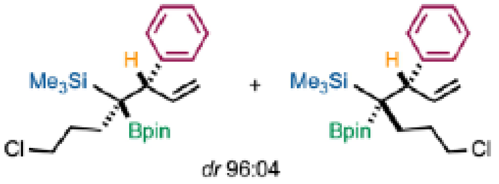

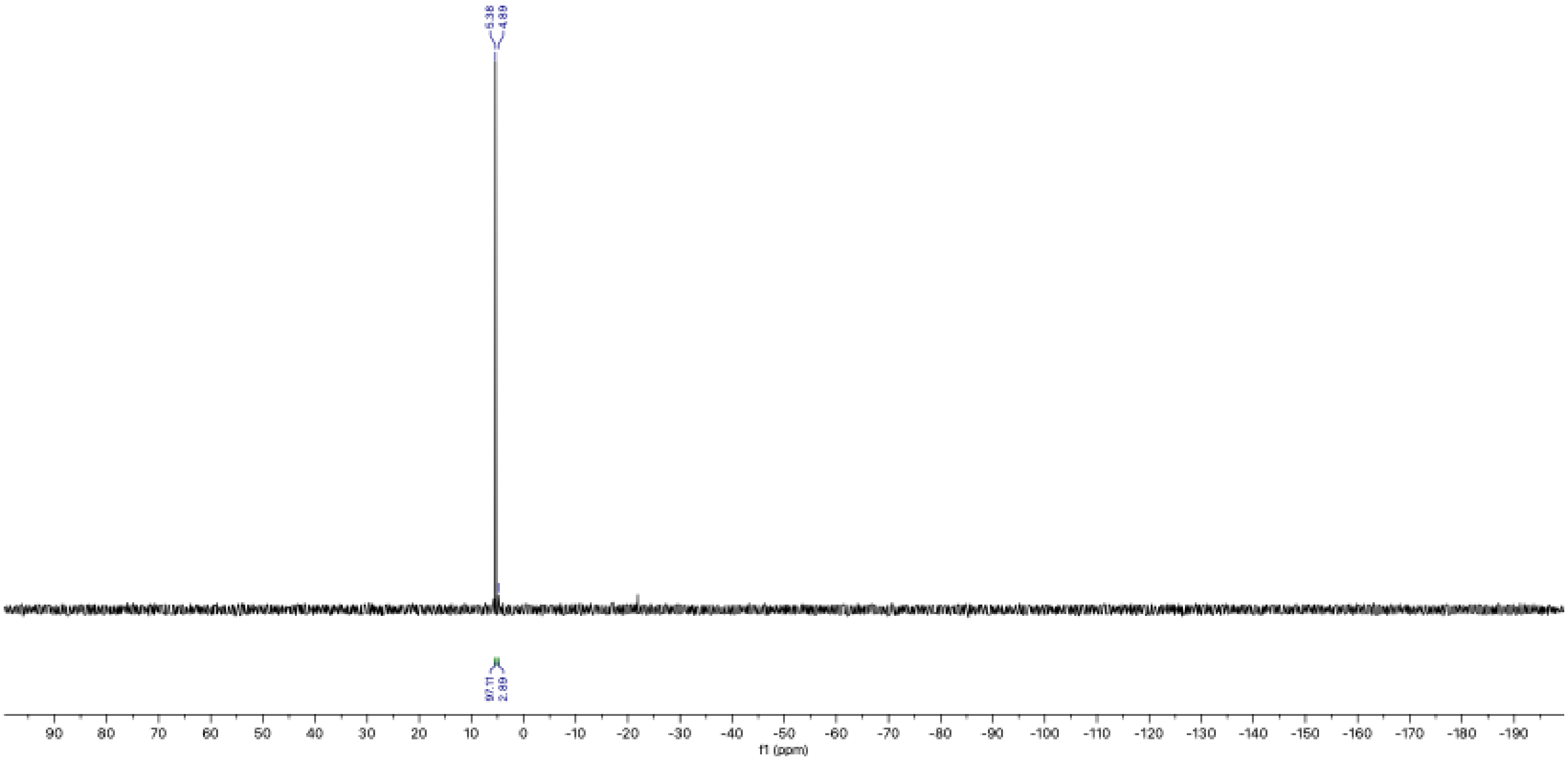

^{29}Si NMR spectrum (80 MHz, $CDCl_3$)

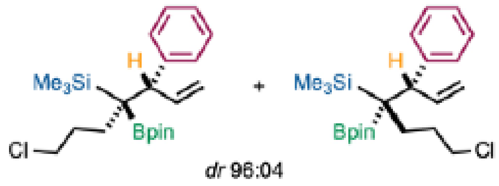

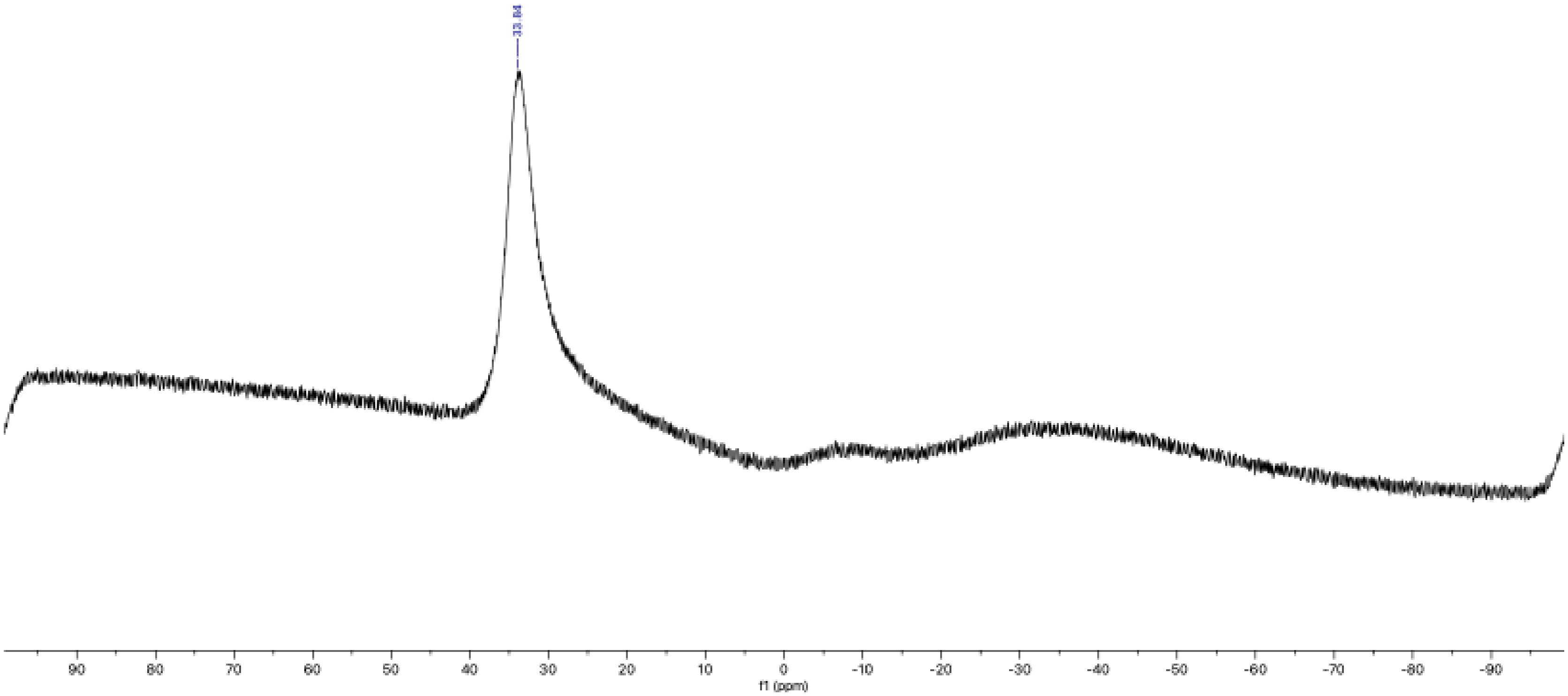

^{11}B NMR spectrum (128 MHz, $CDCl_3$)

((1*S,2*S**)-1-Cyclohexyl-2-phenyl-1-(4,4,5,5-tetramethyl-1,3,2-dioxaborolan-2-yl)but-3-en-1-yl)trimethylsilane 5l**

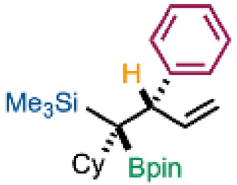

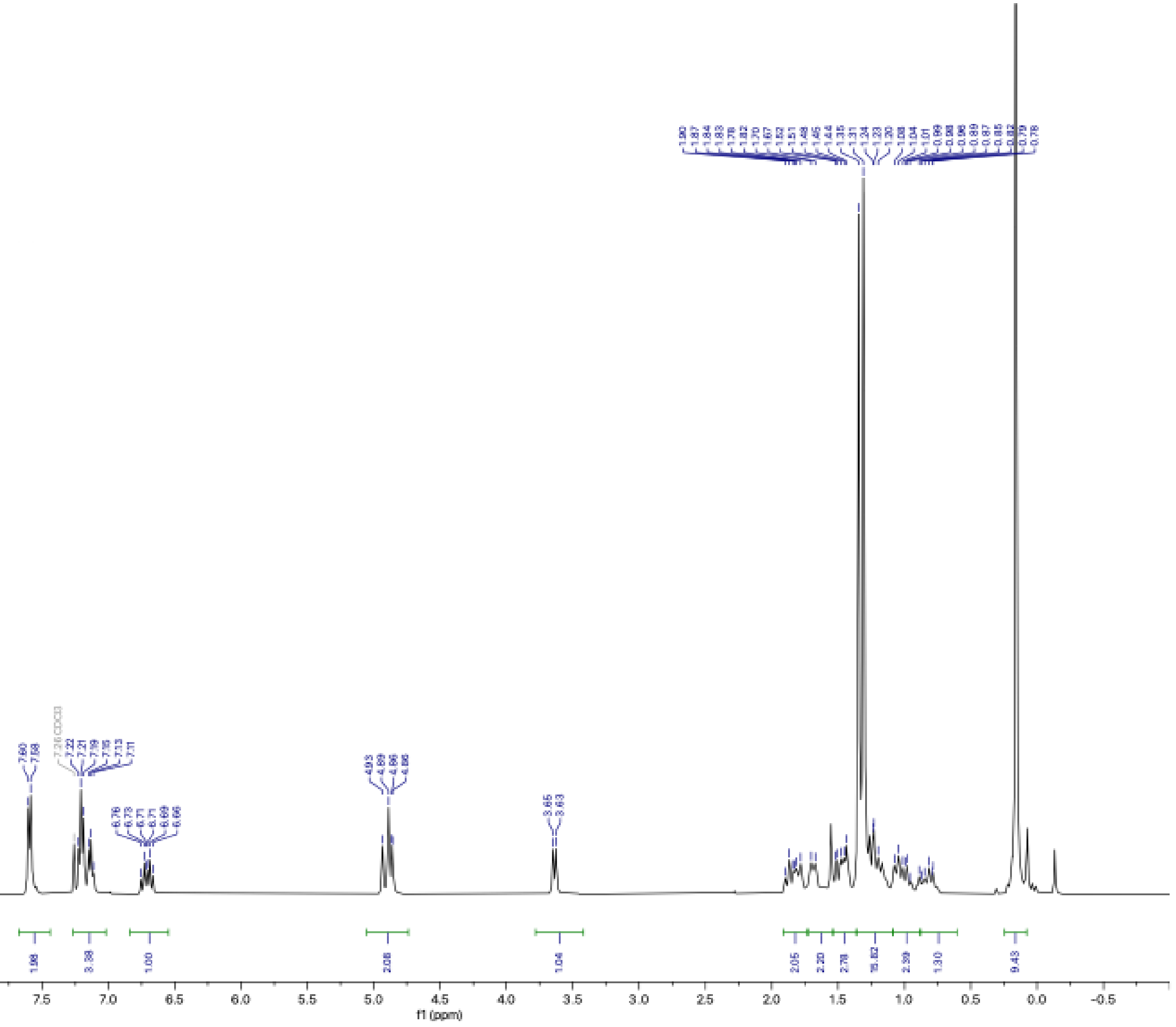

^{1}H NMR spectrum (400 MHz, $CDCl_3$)

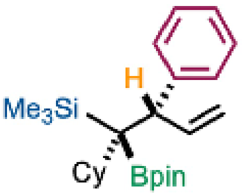

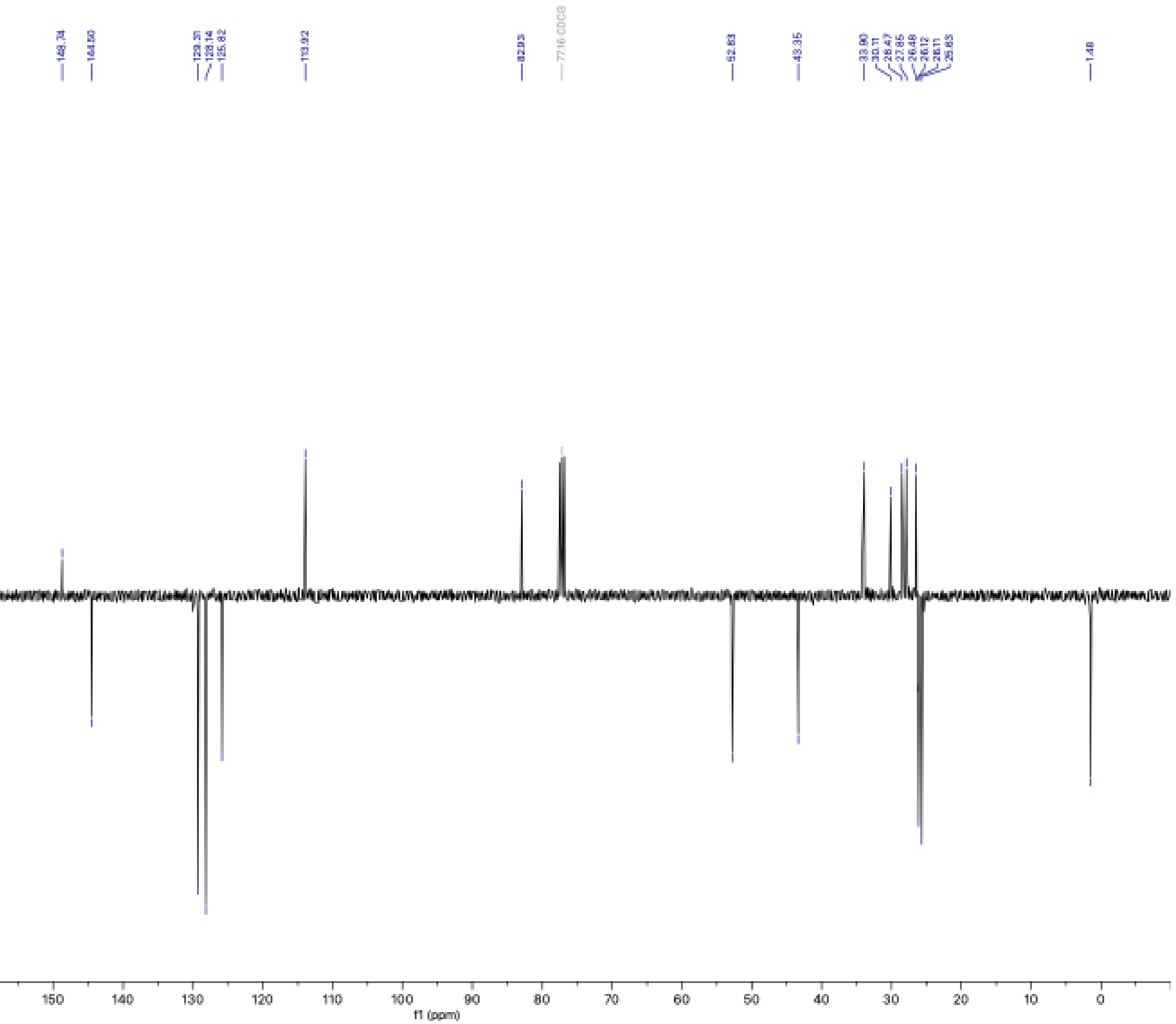

^{13}**C NMR** spectrum (101 MHz, $CDCl_3$)

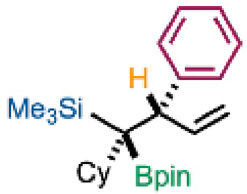

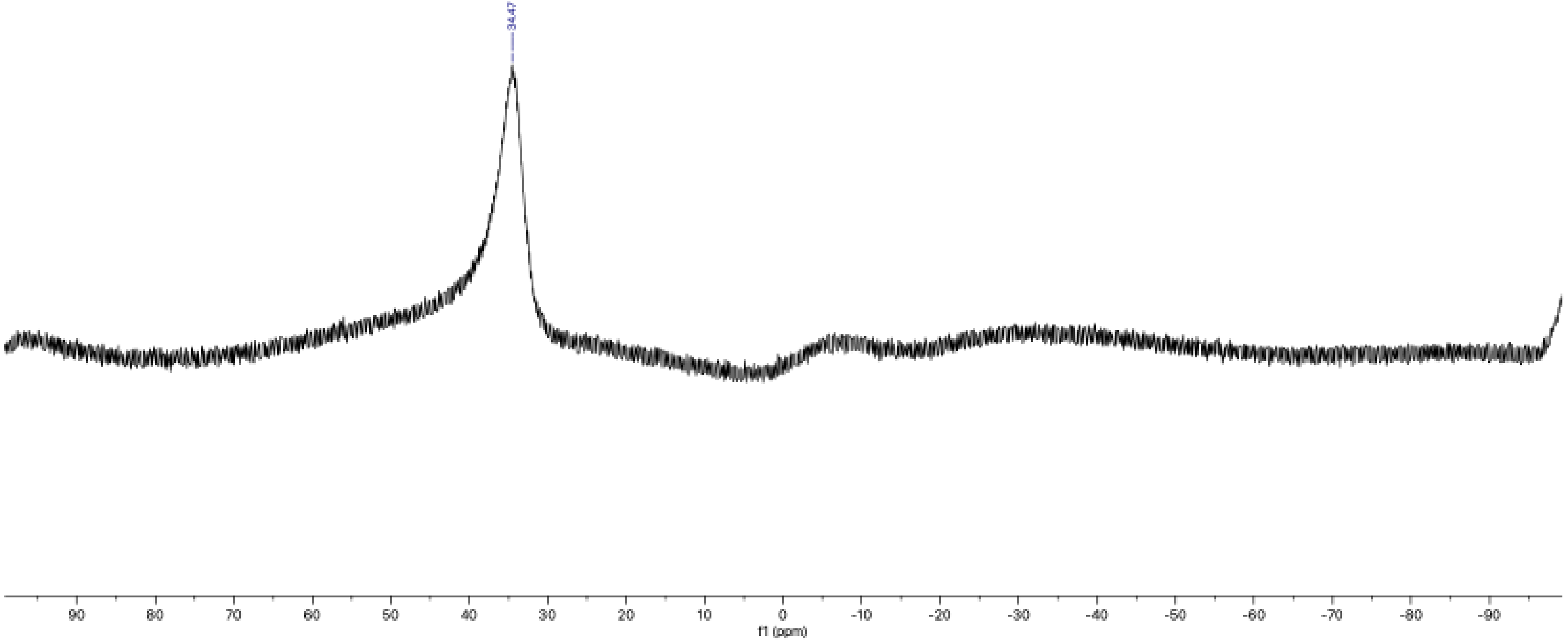

^{11}B NMR spectrum (128 MHz, $CDCl_3$)

((3*S,4*S**)-4-(4-(*tert*-Butyl)-2,3,5,6-tetrafluorophenyl)-3-(4,4,5,5-tetramethyl-1,3,2-dioxaborolan-2-yl)hex-5-en-3-yl)trimethylsilane 5m**

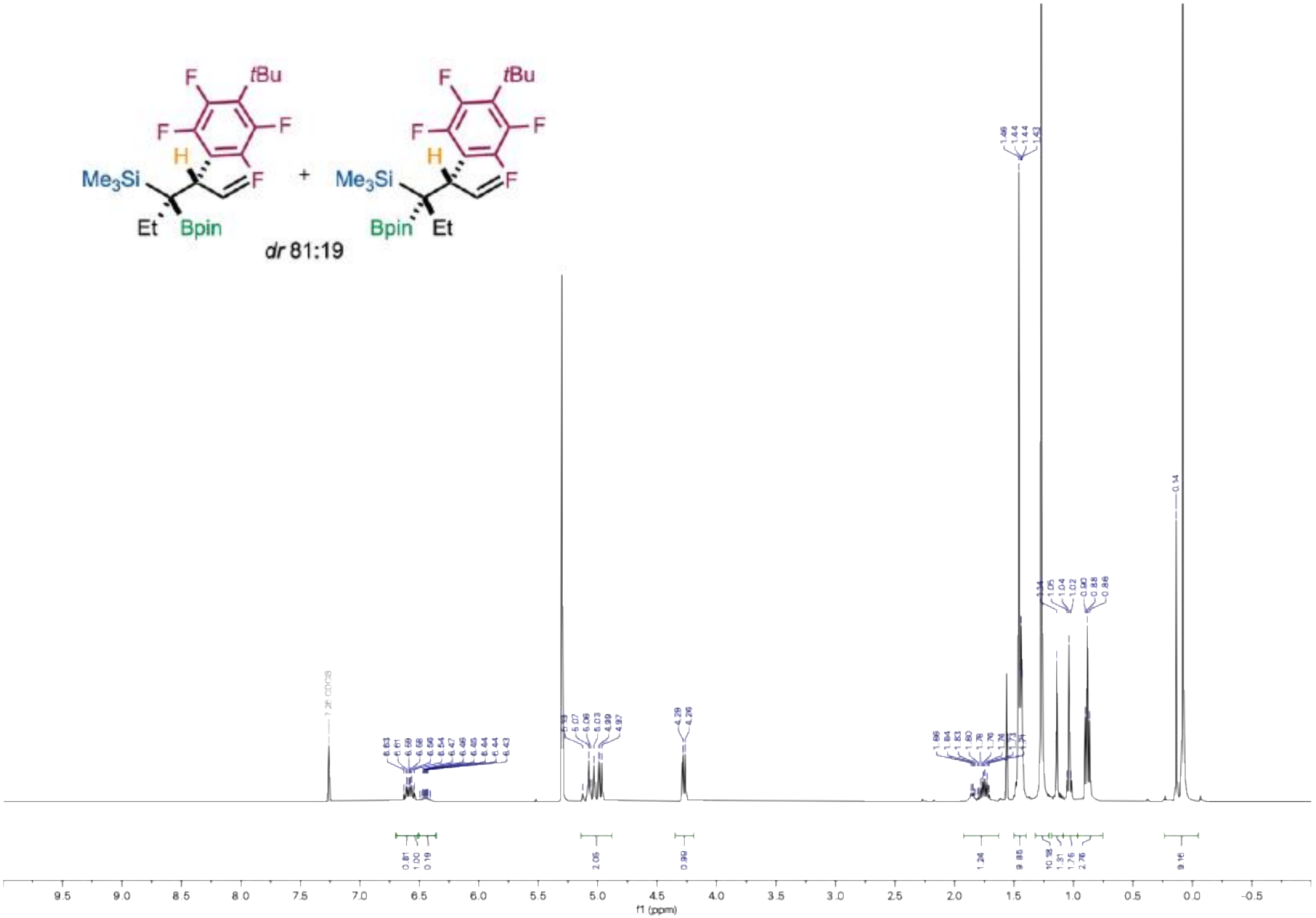

^{1}H NMR spectrum (400 MHz, $CDCl_3$)

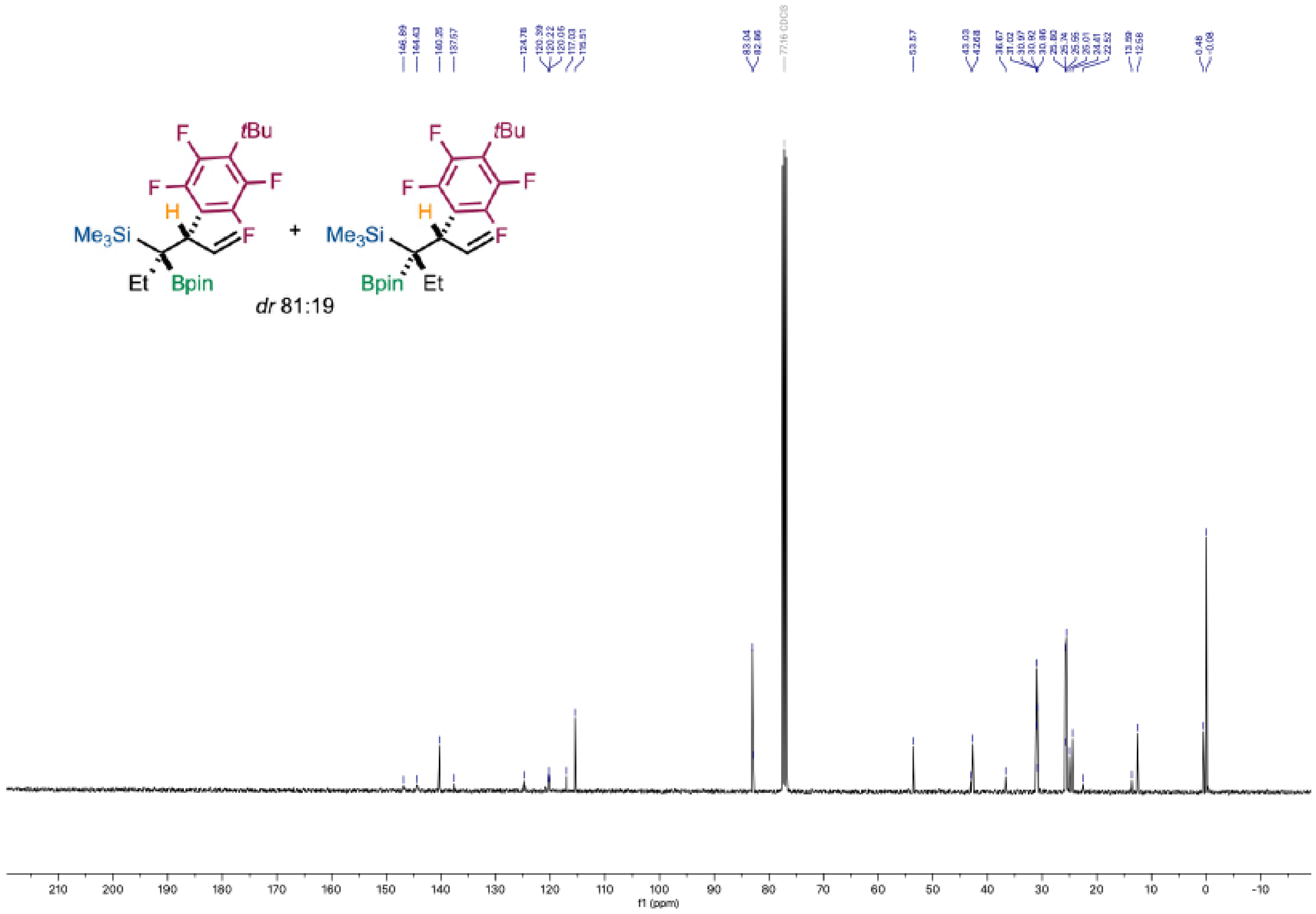

^{13}**C NMR** spectrum (101 MHz, $CDCl_3$)

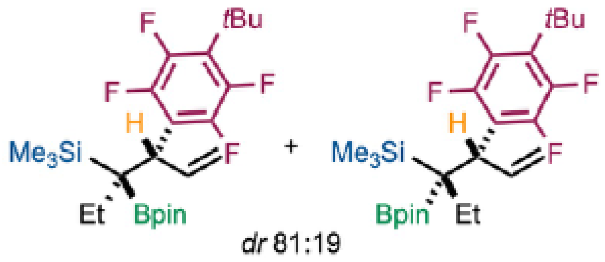

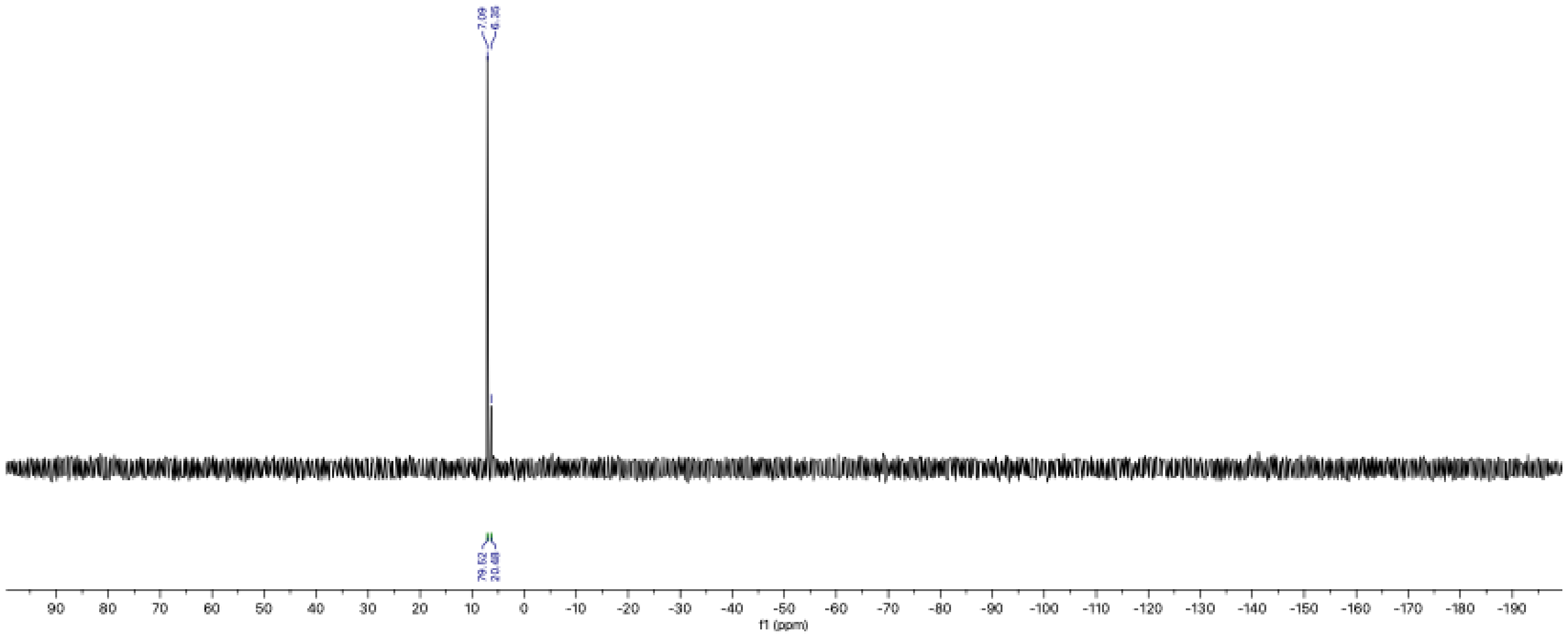

^{29}Si NMR spectrum (80 MHz, $CDCl_3$)

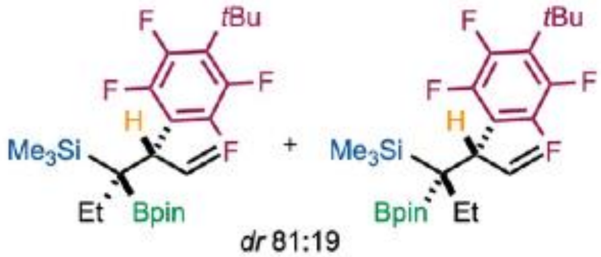

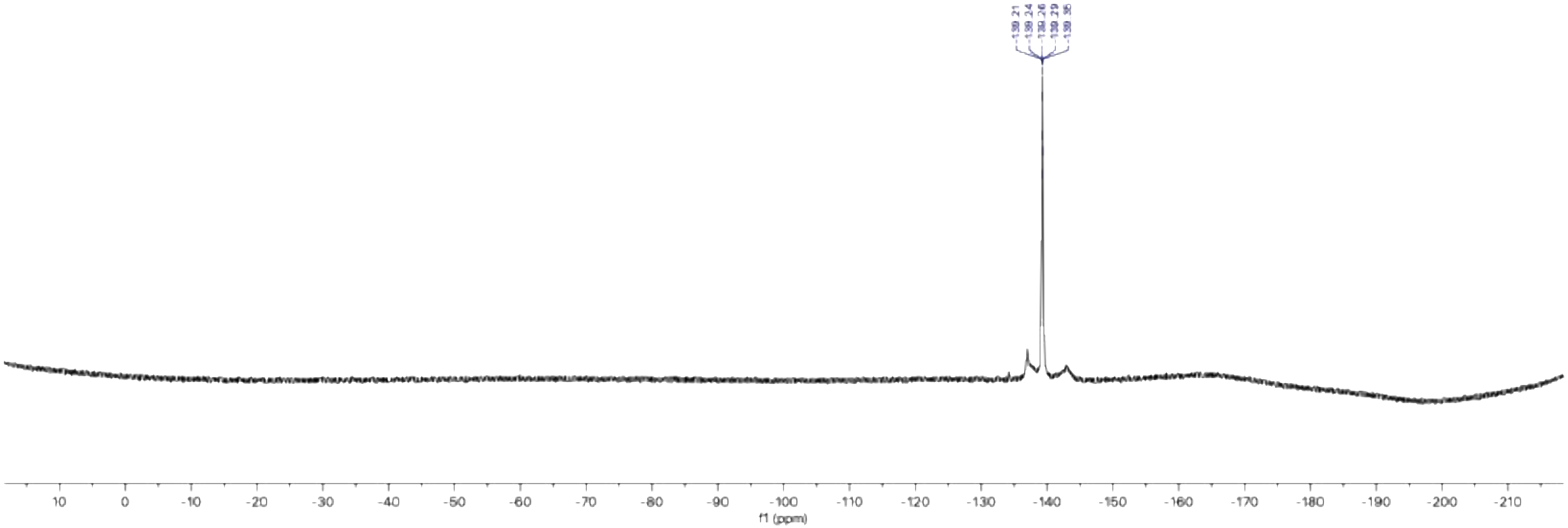

^{19}F NMR spectrum (377 MHz, $CDCl_3$)

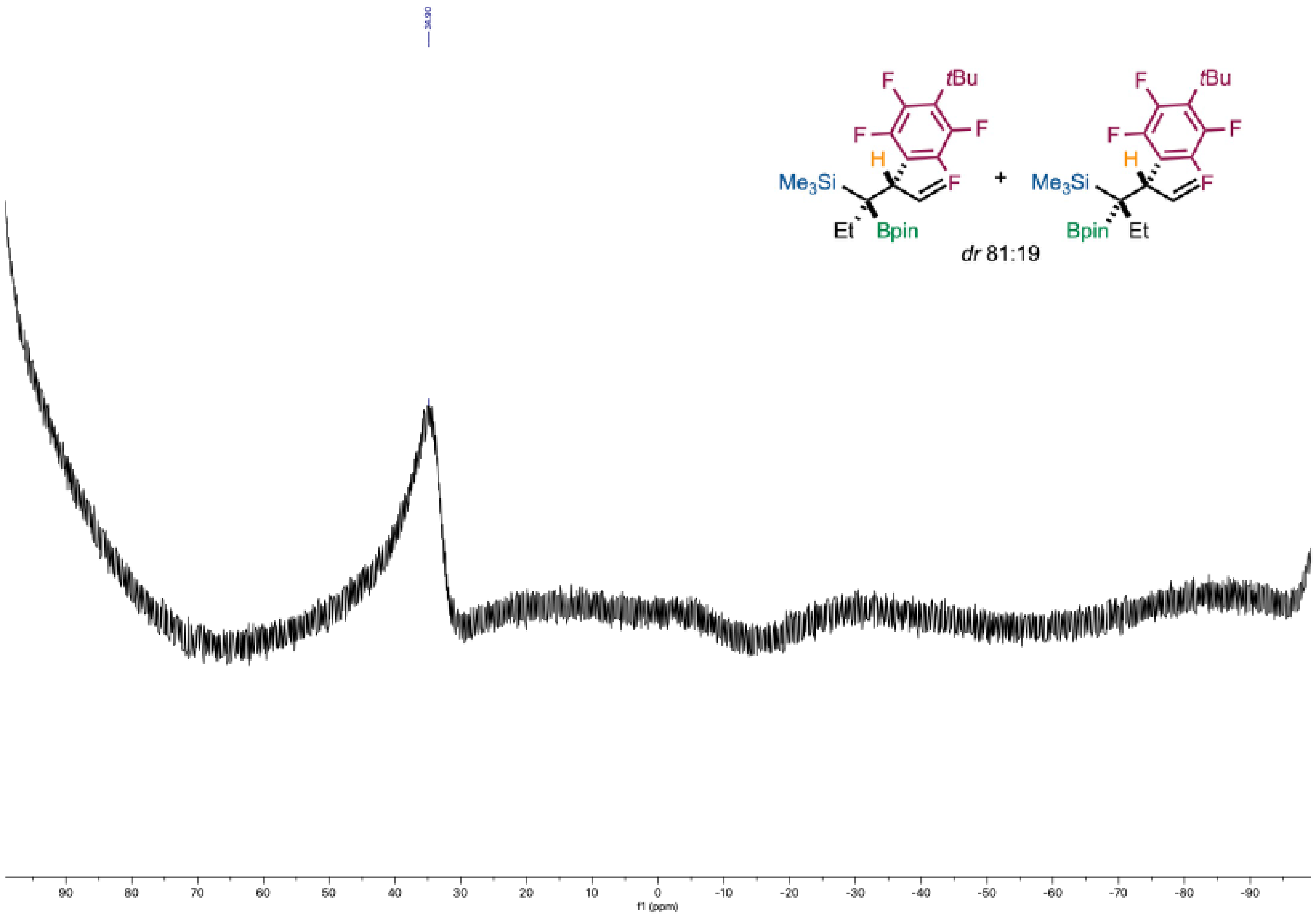

^{11}B NMR spectrum (128 MHz, $CDCl_3$)

Trimethyl((3*S,4*R**)-3-(4,4,5,5-tetramethyl-1,3,2-dioxaborolan-2-yl)-4-(thiophen-2-yl)hex-5-en-3-yl)silane 5n and trimethyl((3*S**,4*R**)-3-(4,4,5,5-tetramethyl-1,3,2-dioxaborolan-2-yl)-4-(5-(trimethylsilyl)thiophen-2-yl)hex-5-en-3-yl)silane 5n-Si**

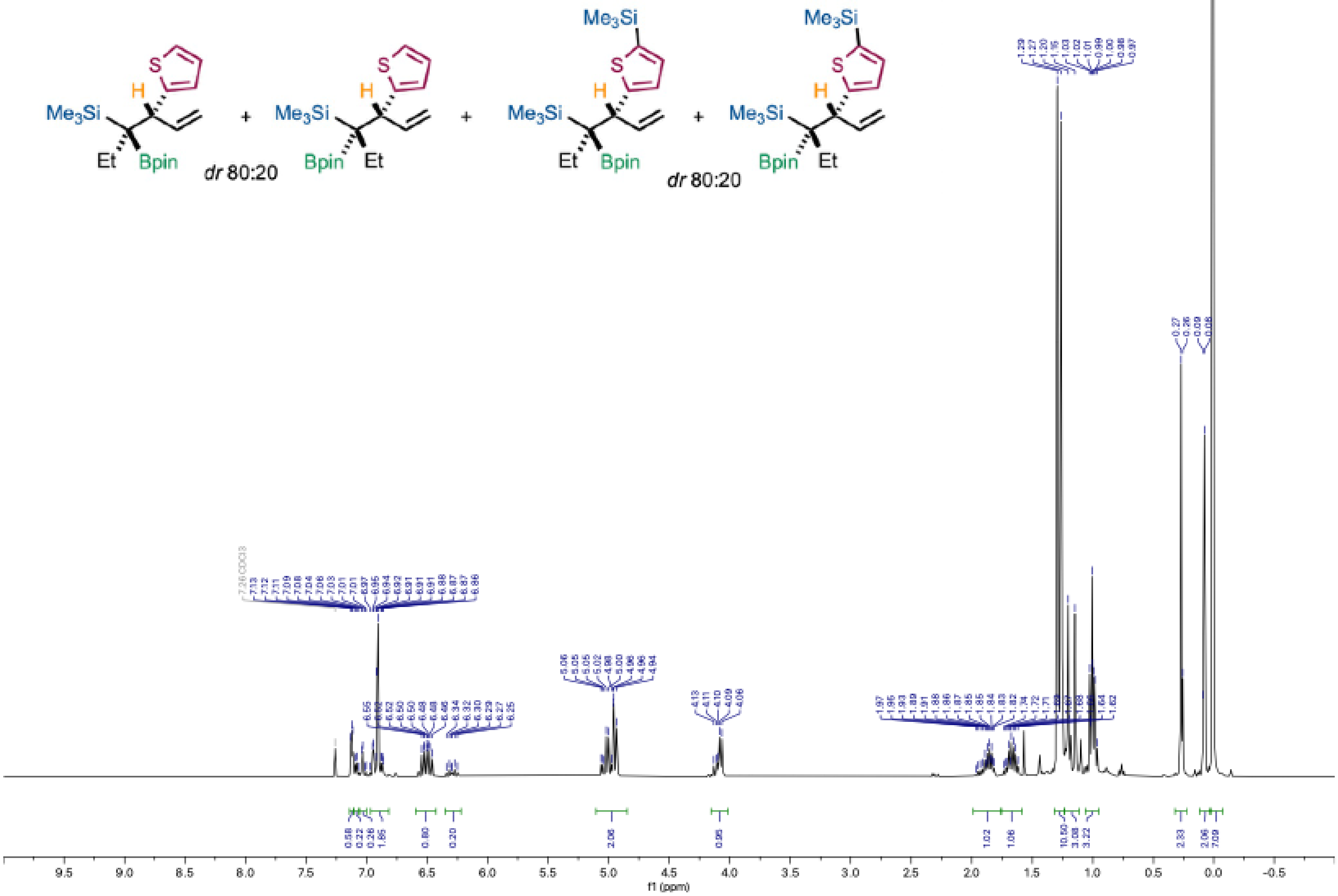

1H NMR spectrum (400 MHz, $CDCl_3$)

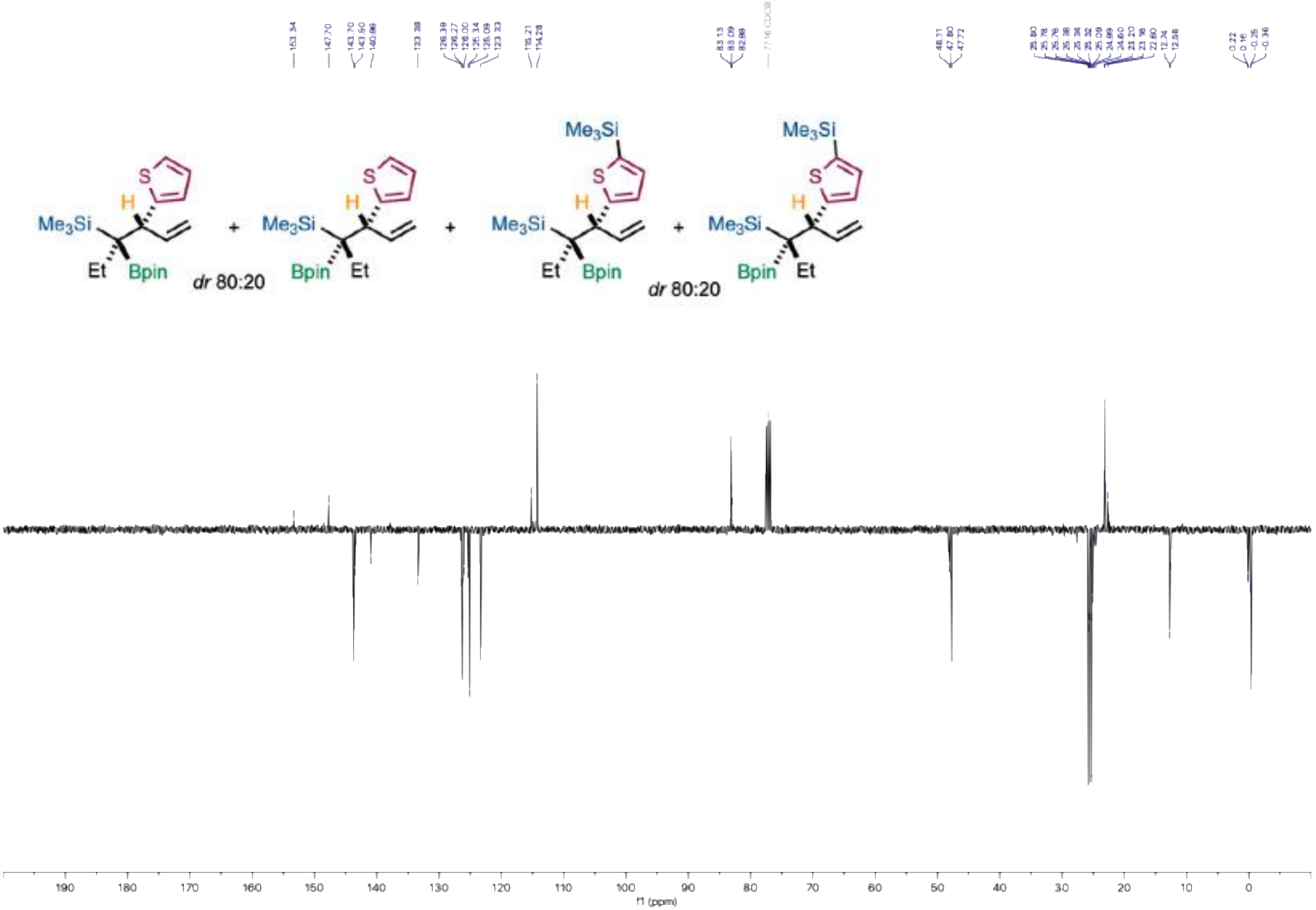

^{13}C NMR spectrum (101 MHz, $CDCl_3$)

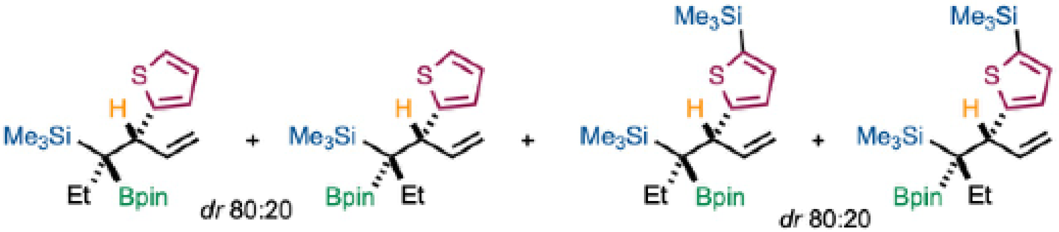

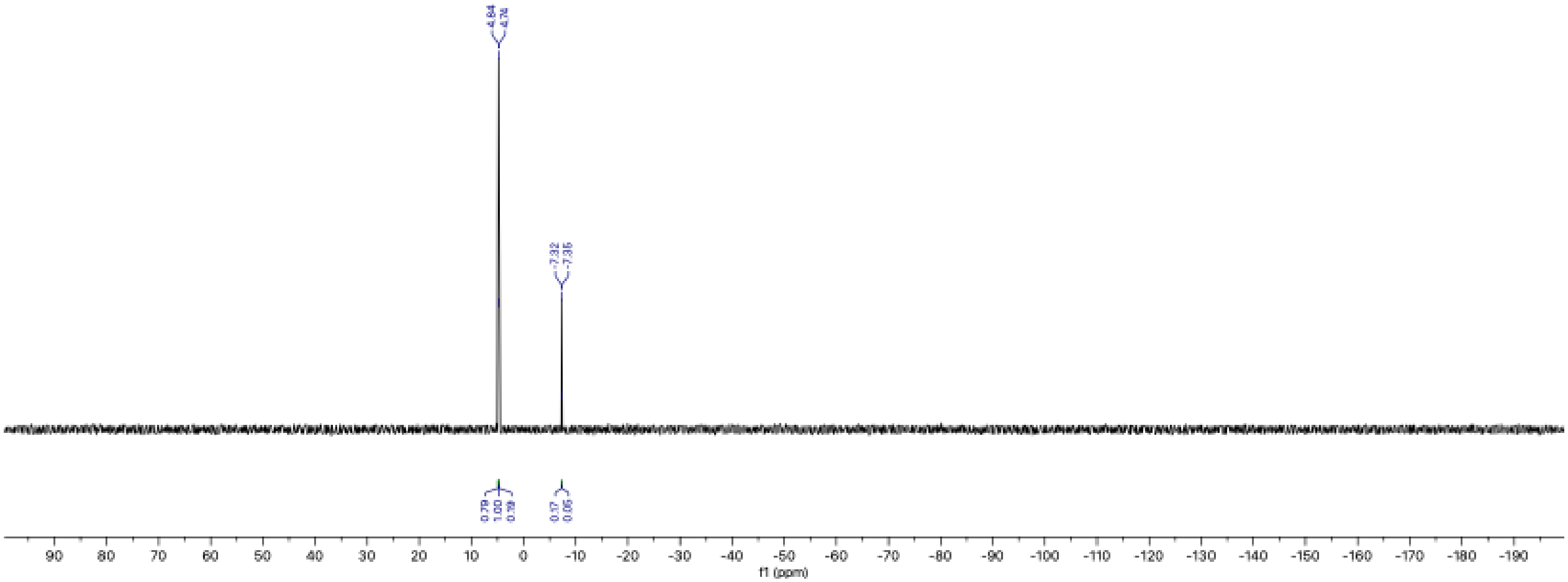

^{29}**Si NMR** spectrum (80 MHz, $CDCl_3$)

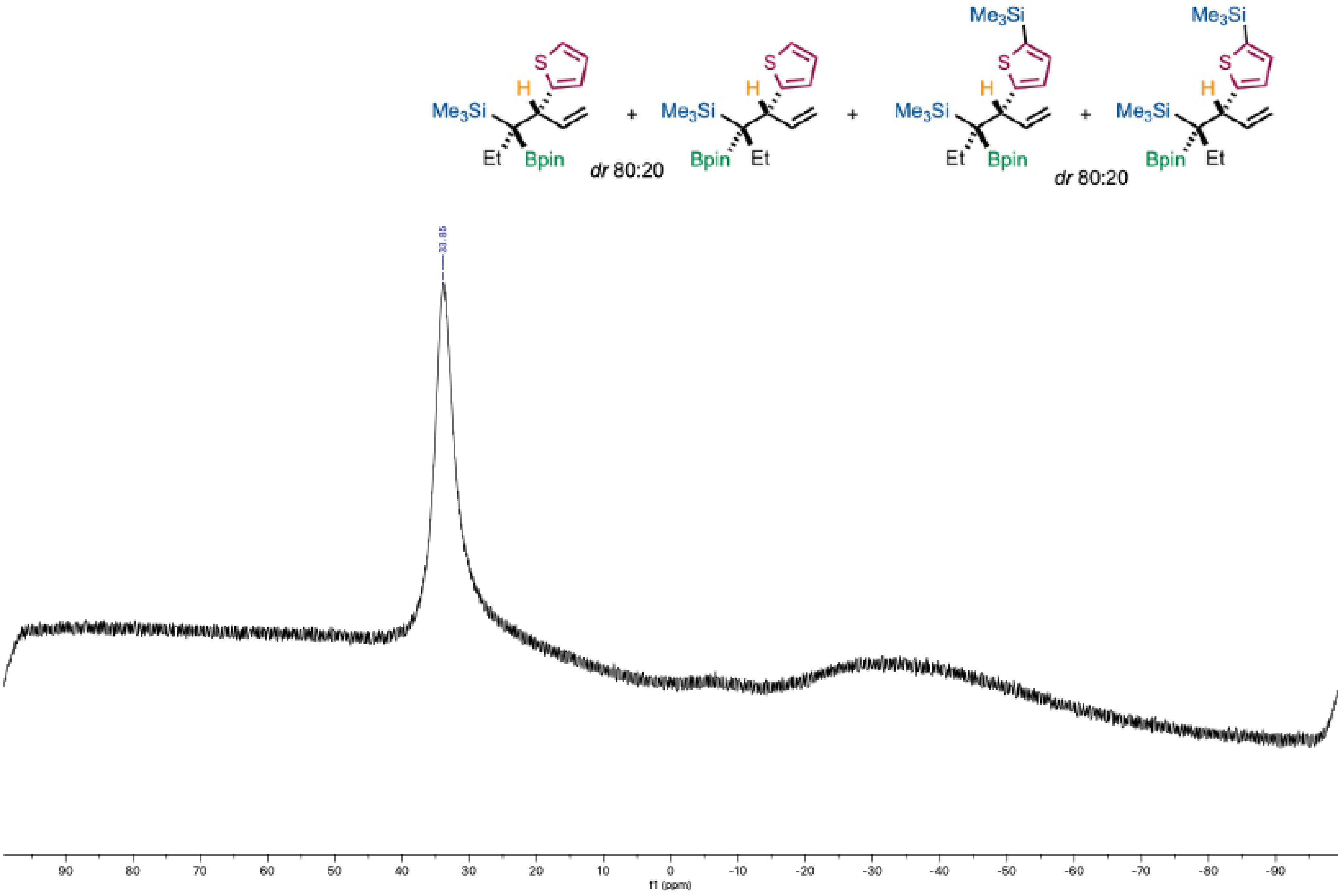

11B NMR spectrum (128 MHz, $CDCl_3$)

Dioxo[*N*,*N*,*N*,*N*-tetramethylethane-1,2-diaminetetramethylehylendiamine][(2*R,3*R**,4*S**)-3-phenyl-4-(4,4,5,5-tetramethyl-1,3,2-dioxaborolan-2-yl)-4-(trimethylsilyl)hexane-1,2-diol]osmium complex 6**

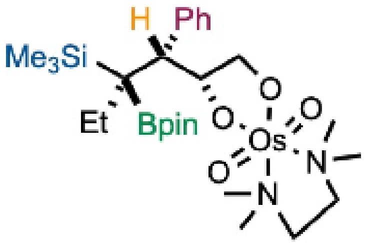

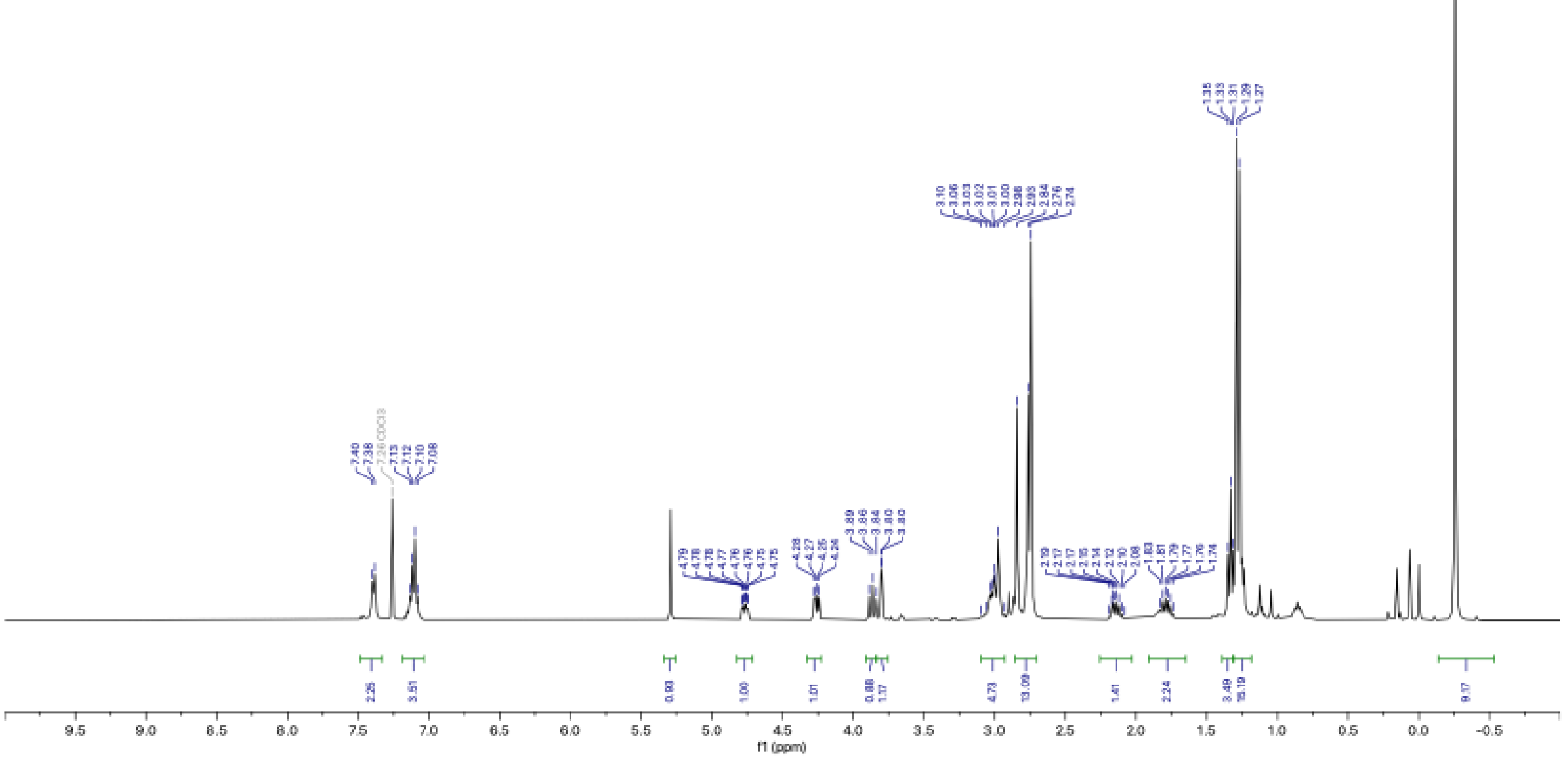

^{1}H NMR spectrum (400 MHz, $CDCl_3$)

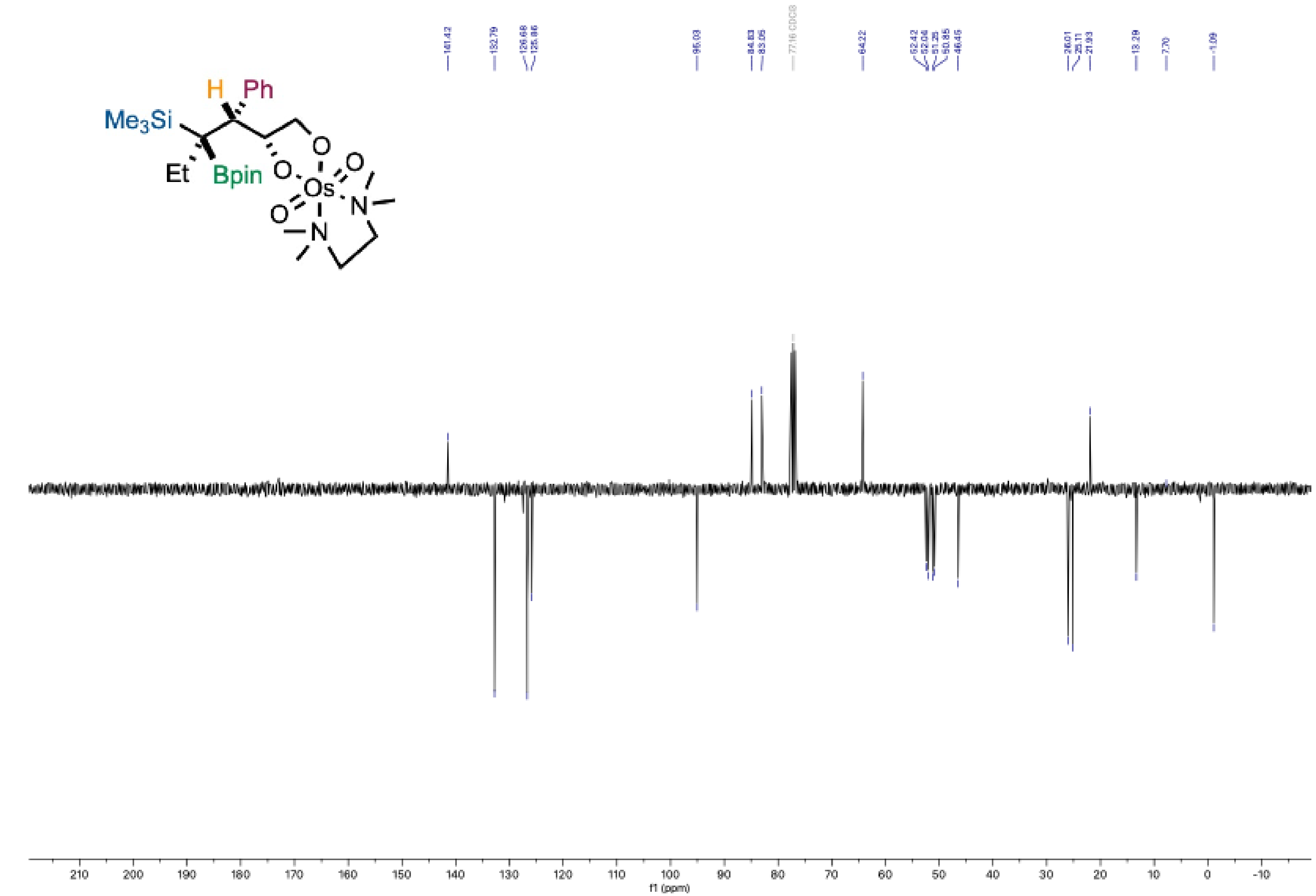

^{13}**C NMR** spectrum (101 MHz, $CDCl_3$)

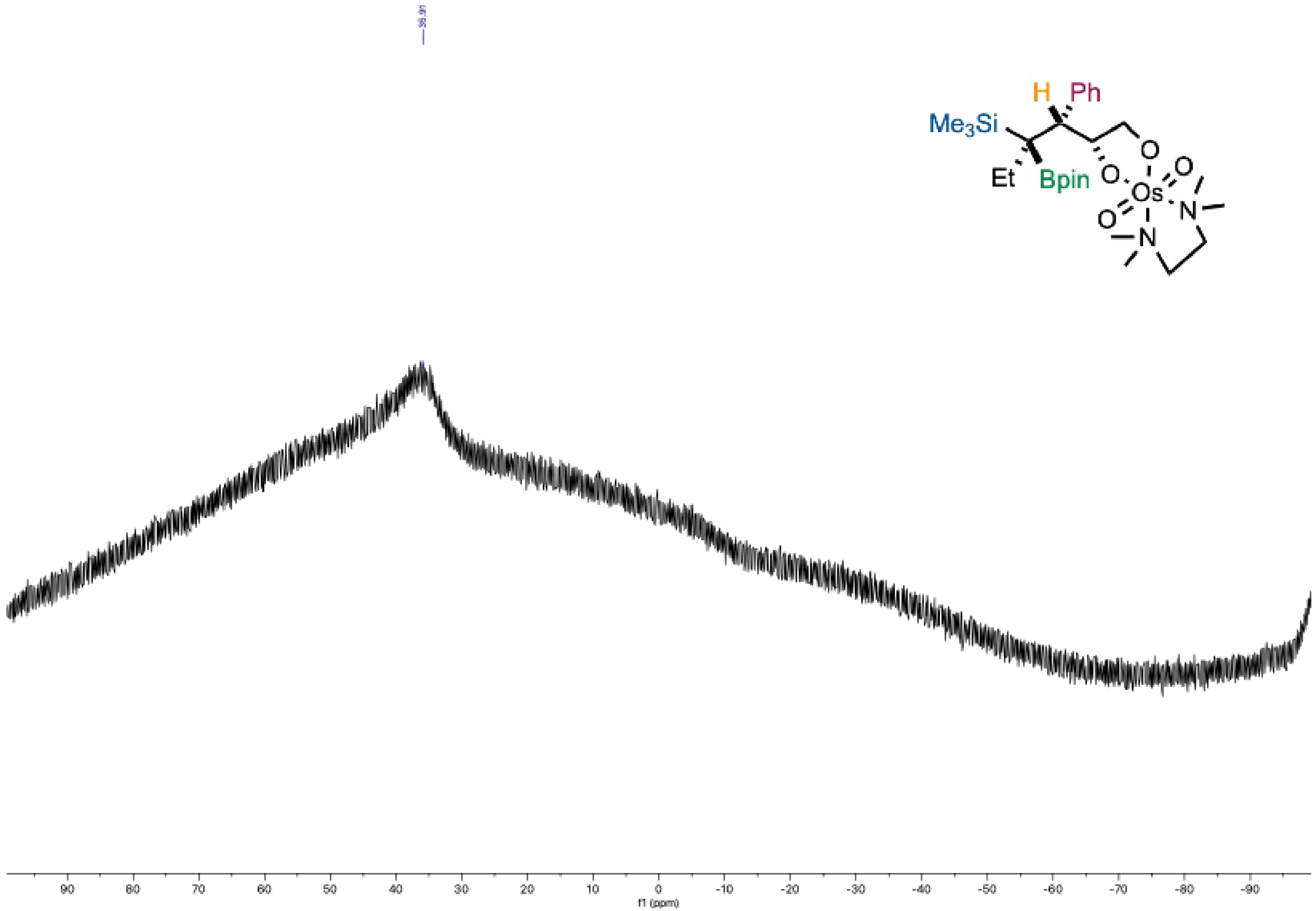

^{11}B NMR spectrum (128 MHz, $CDCl_3$)

4,4,5,5-Tetramethyl-2-(4-phenylhex-5-en-3-yl)-1,3,2-dioxaborolane 5a-H

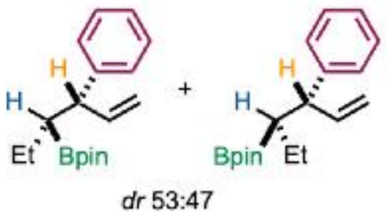

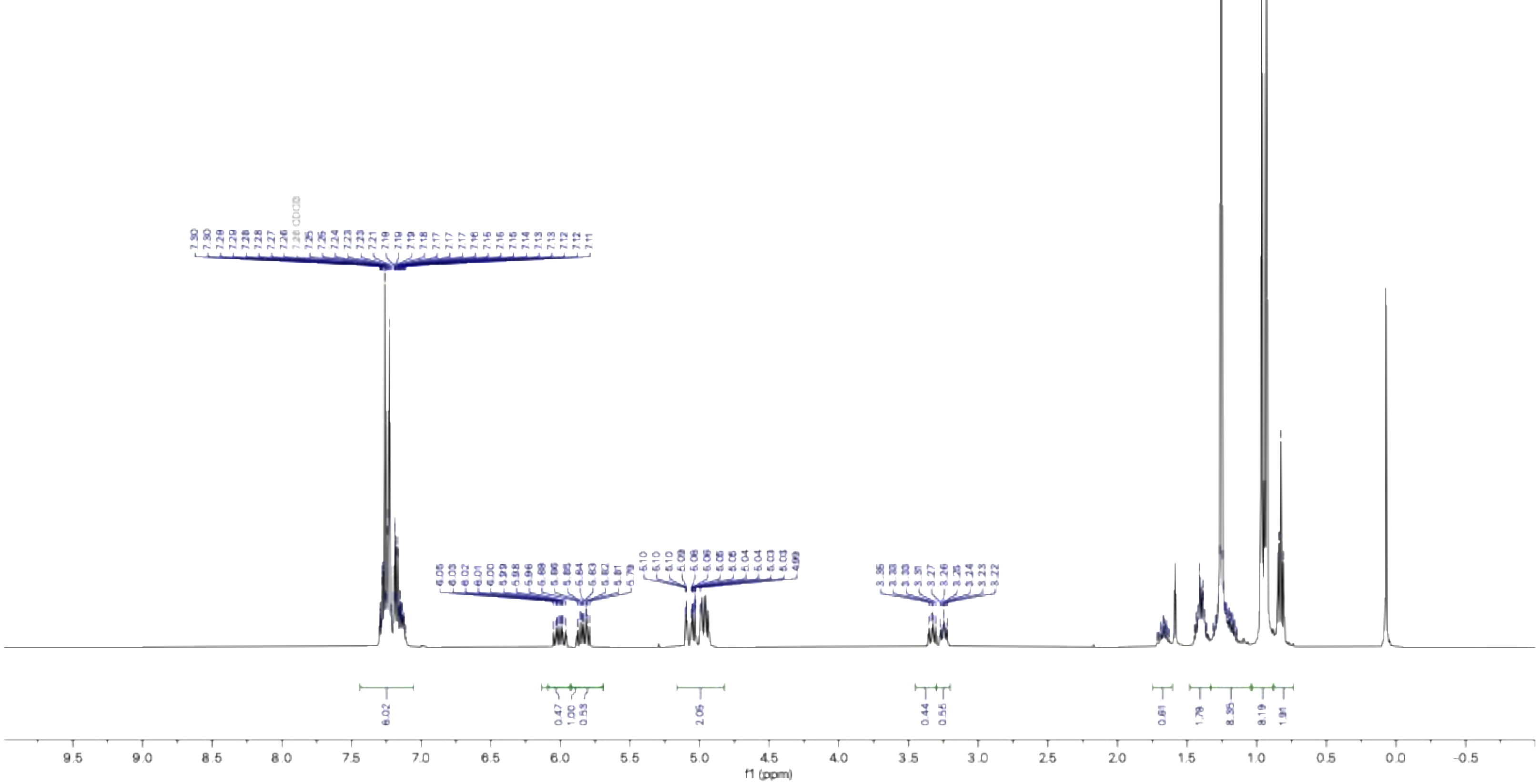

^{1}**H NMR** spectrum (400 MHz, $CDCl_3$)

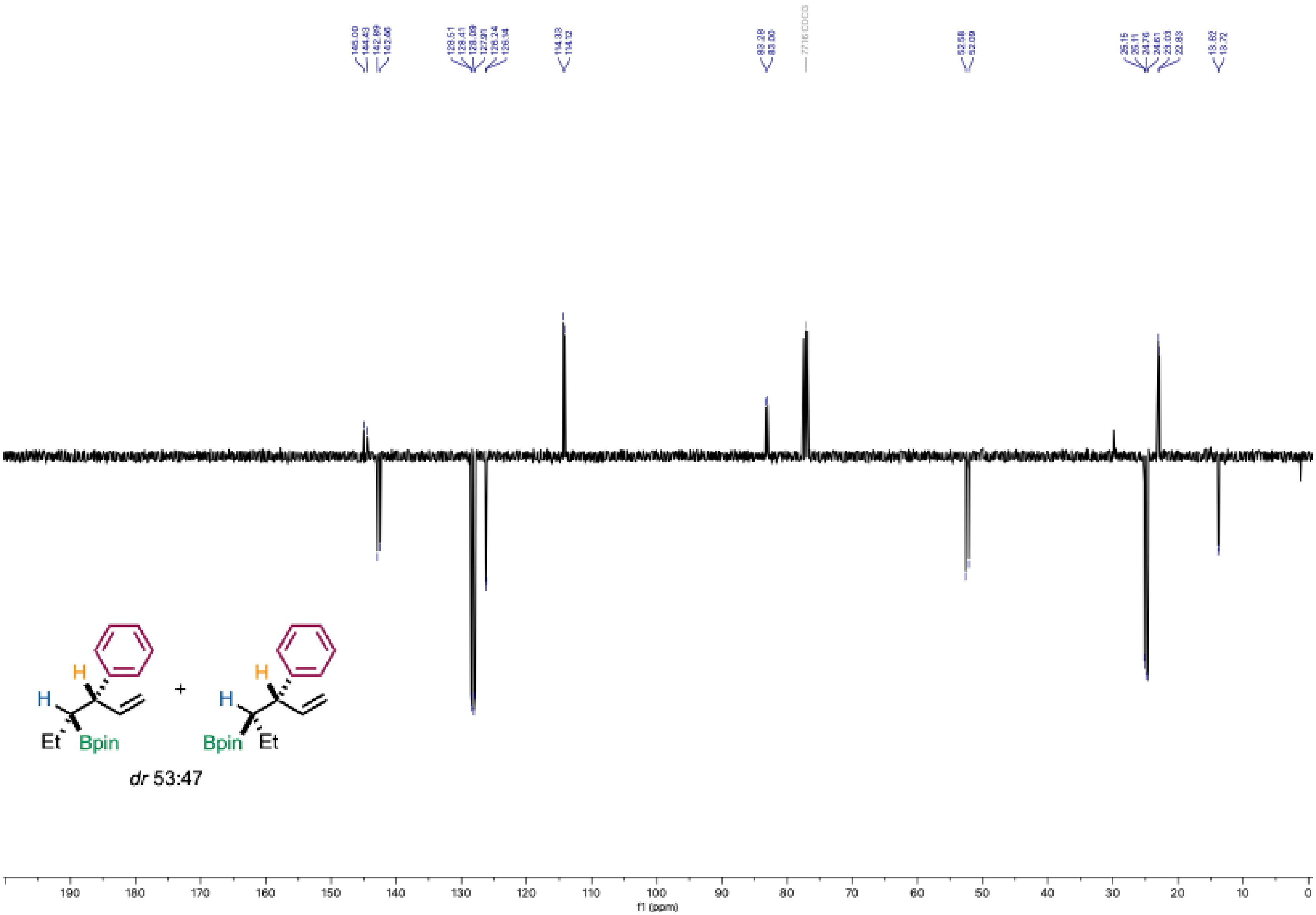

^{13}C NMR spectrum (101 MHz, $CDCl_3$)

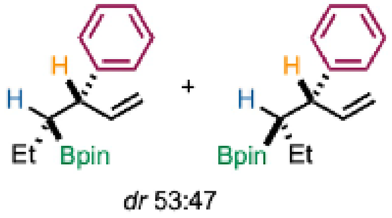

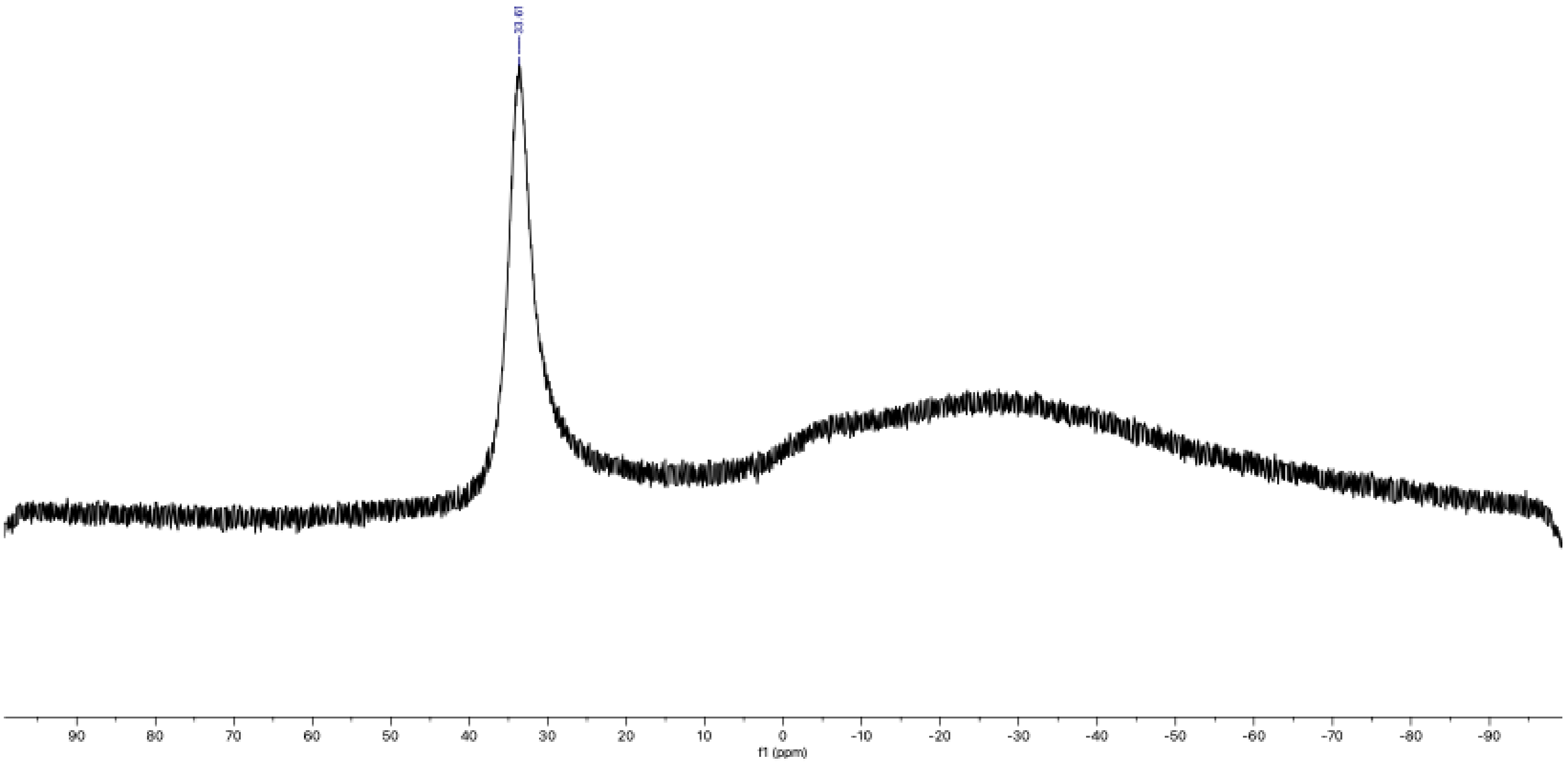

^{11}**B NMR** spectrum (128 MHz, $CDCl_3$)

4,4,5,5-Tetramethyl-2-((3*S,4*R**)-3-methyl-4-phenylhex-5-en-3-yl)-1,3,2-dioxaborolane 7a**

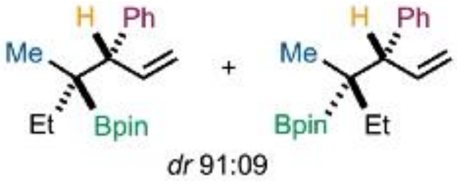

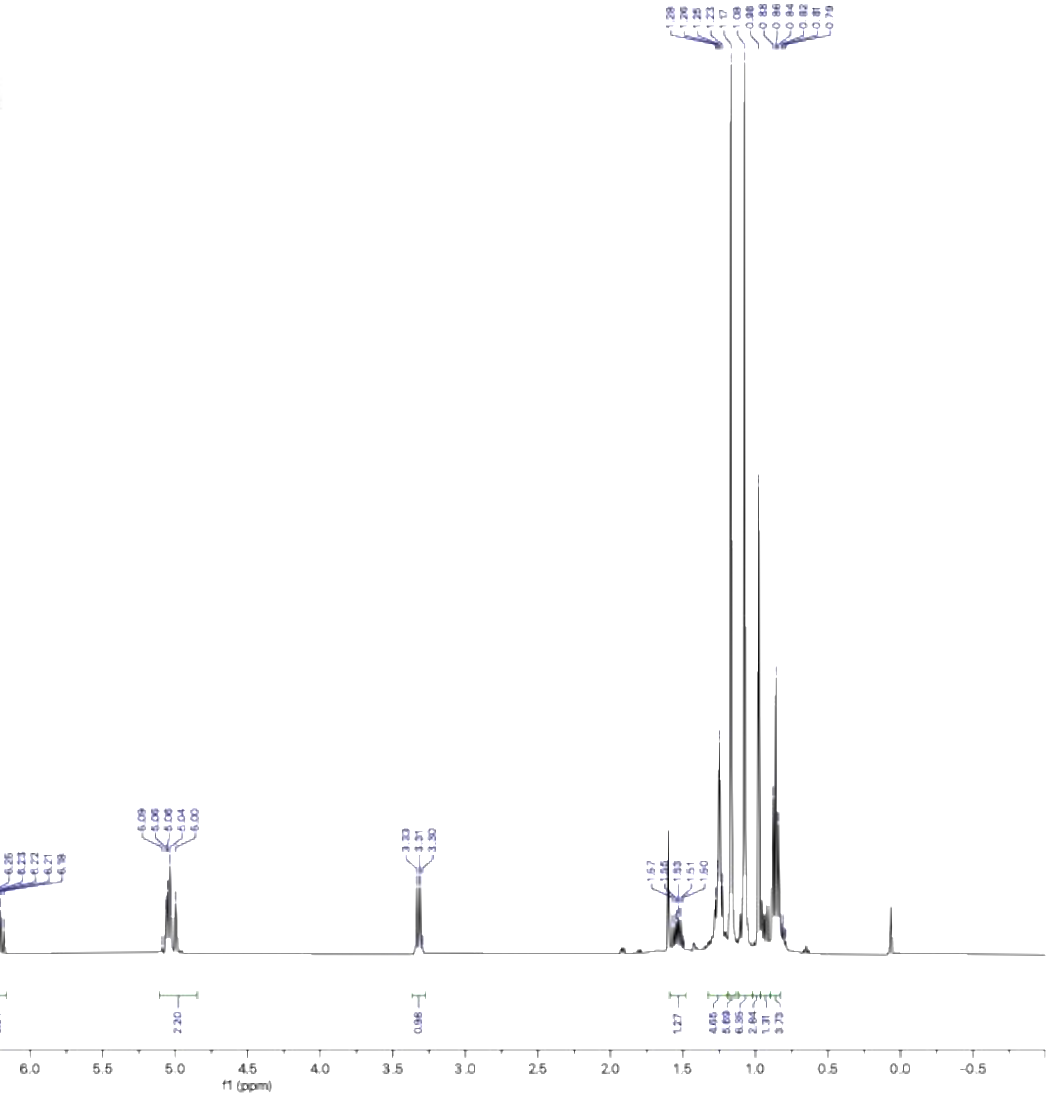

^{1}H NMR spectrum (400 MHz, $CDCl_3$)

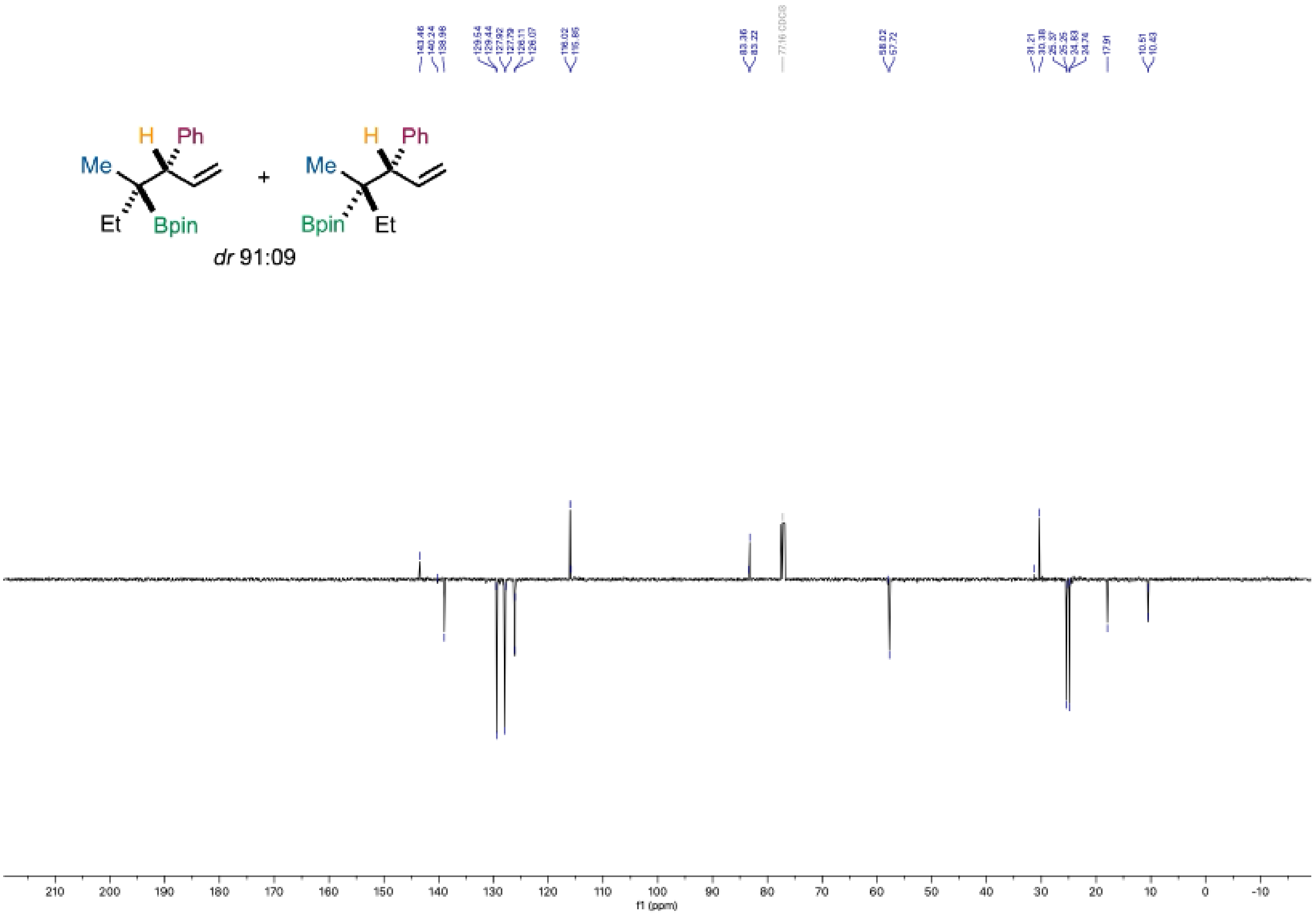

^{13}C NMR spectrum (101 MHz, $CDCl_3$)

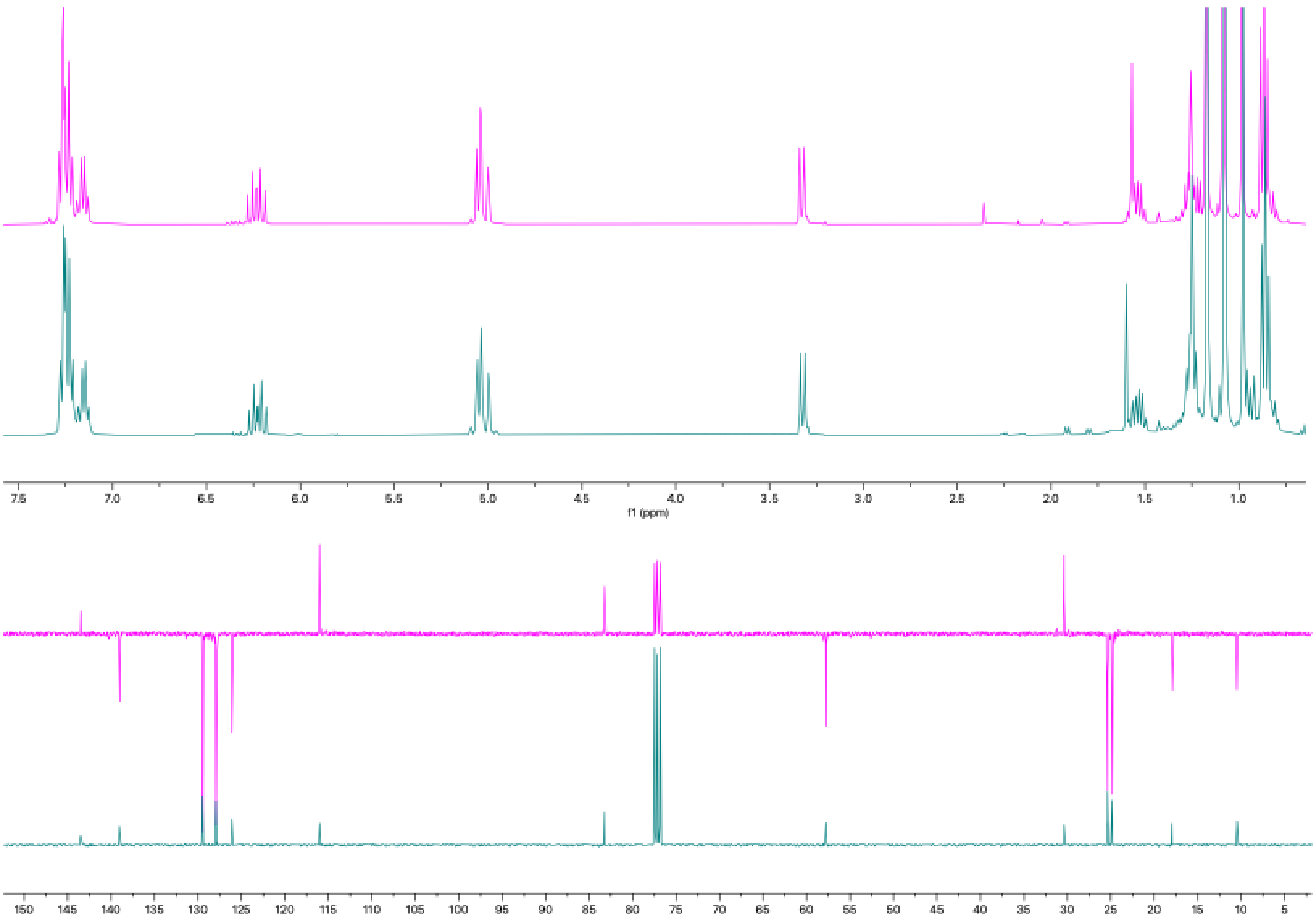

Stacked ^{1}H (top) and ^{13}C (bottom) NMR spectra of **7b** (pink) and the literature reference (teal).[3b]

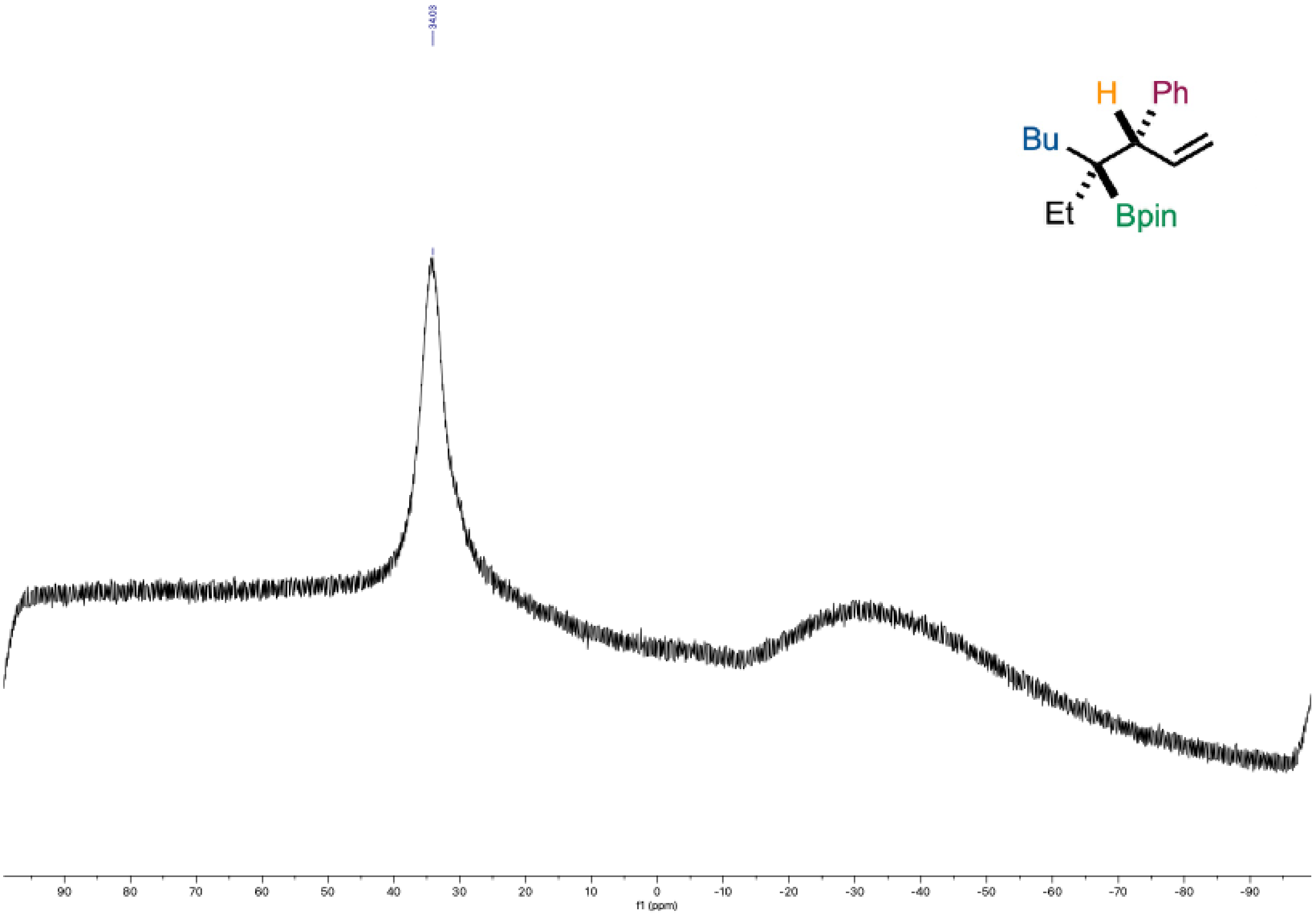

^{11}B NMR spectrum (128 MHz, $CDCl_3$)

2-((3*R,4*R**)-4-Ethyl-3-phenyloct-1-en-4-yl)-4,4,5,5-tetramethyl-1,3,2-dioxaborolane 7b**

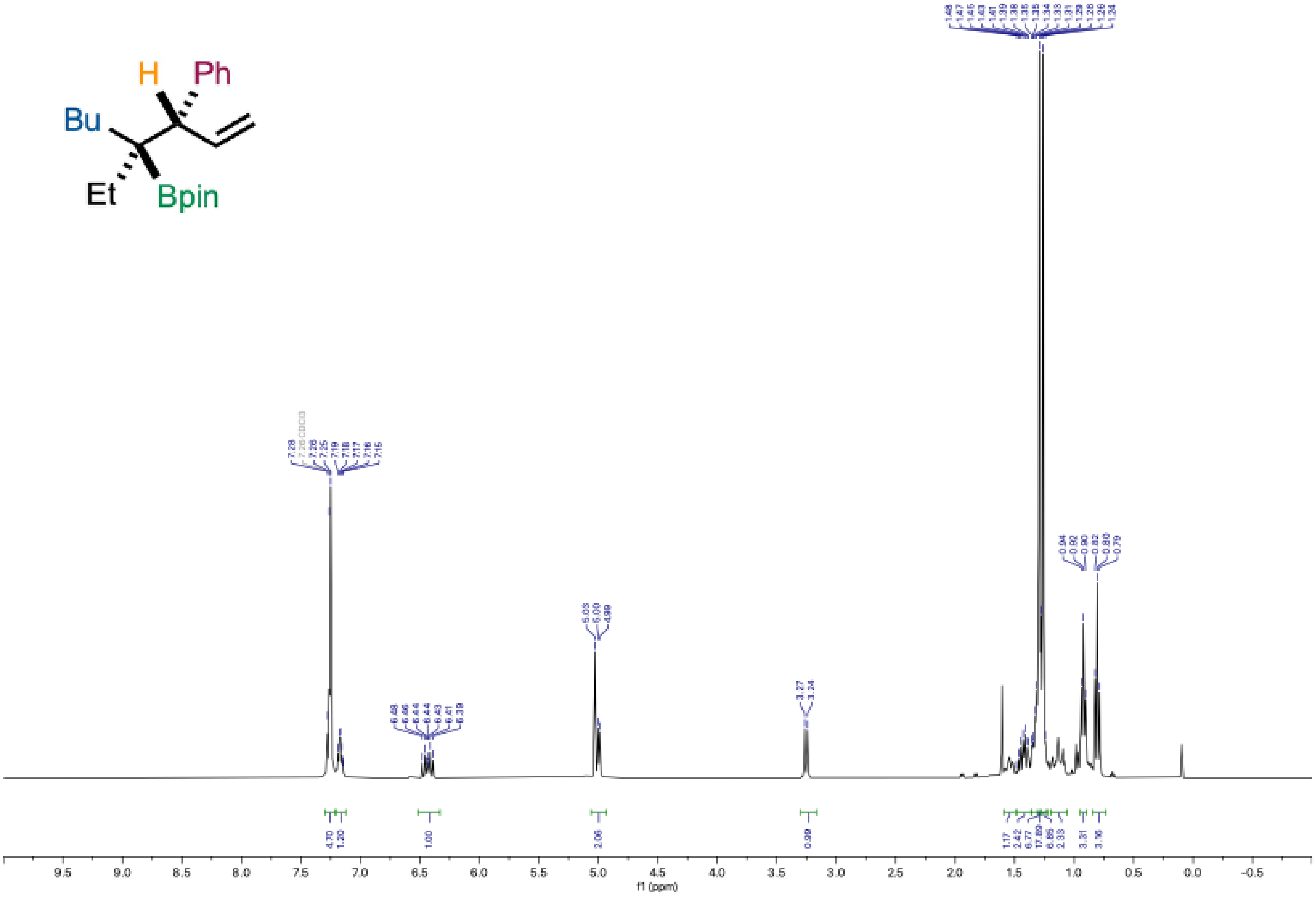

[1]**H NMR** spectrum (400 MHz, $CDCl_3$)

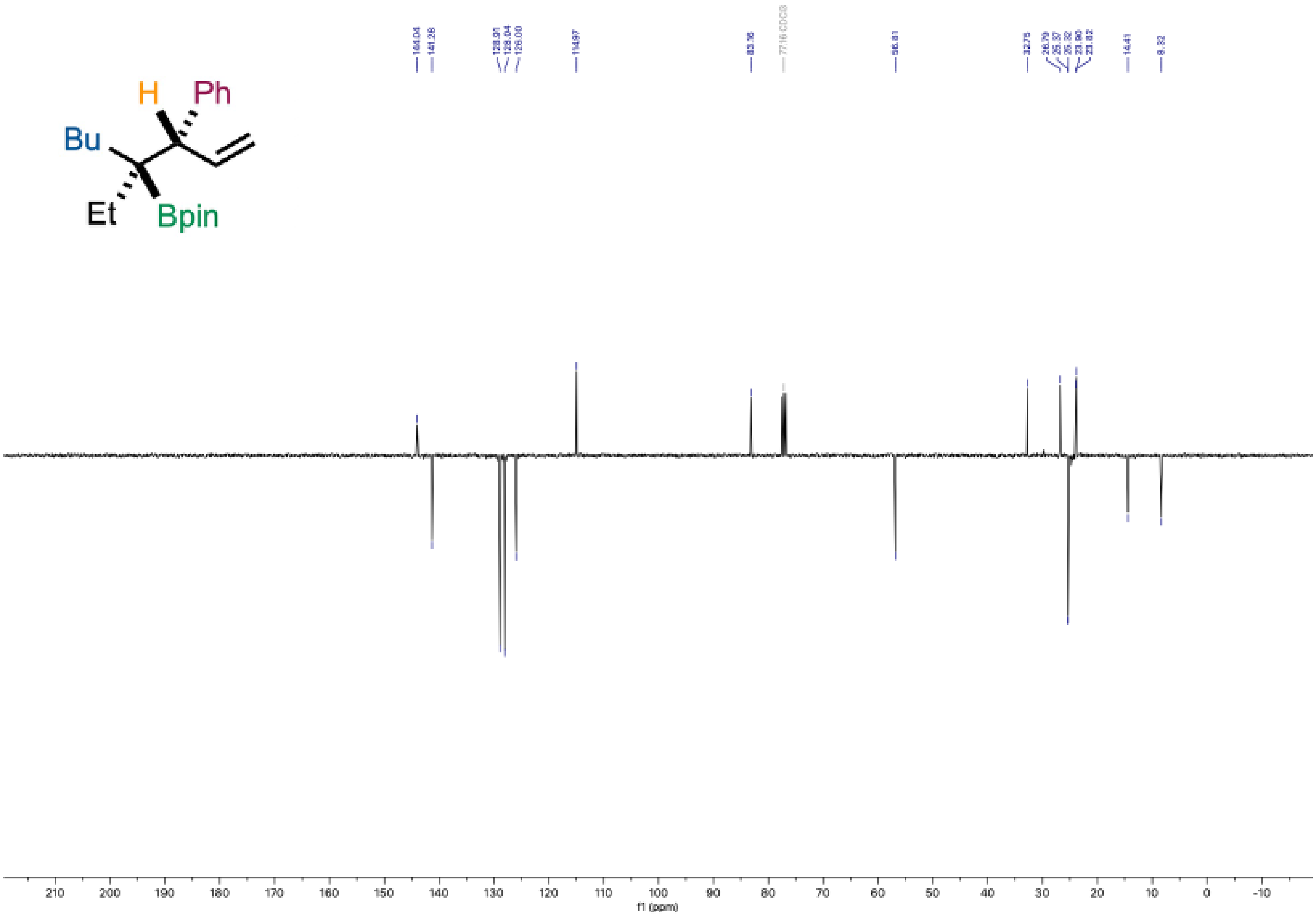

^{13}C NMR spectrum (101 MHz, $CDCl_3$)

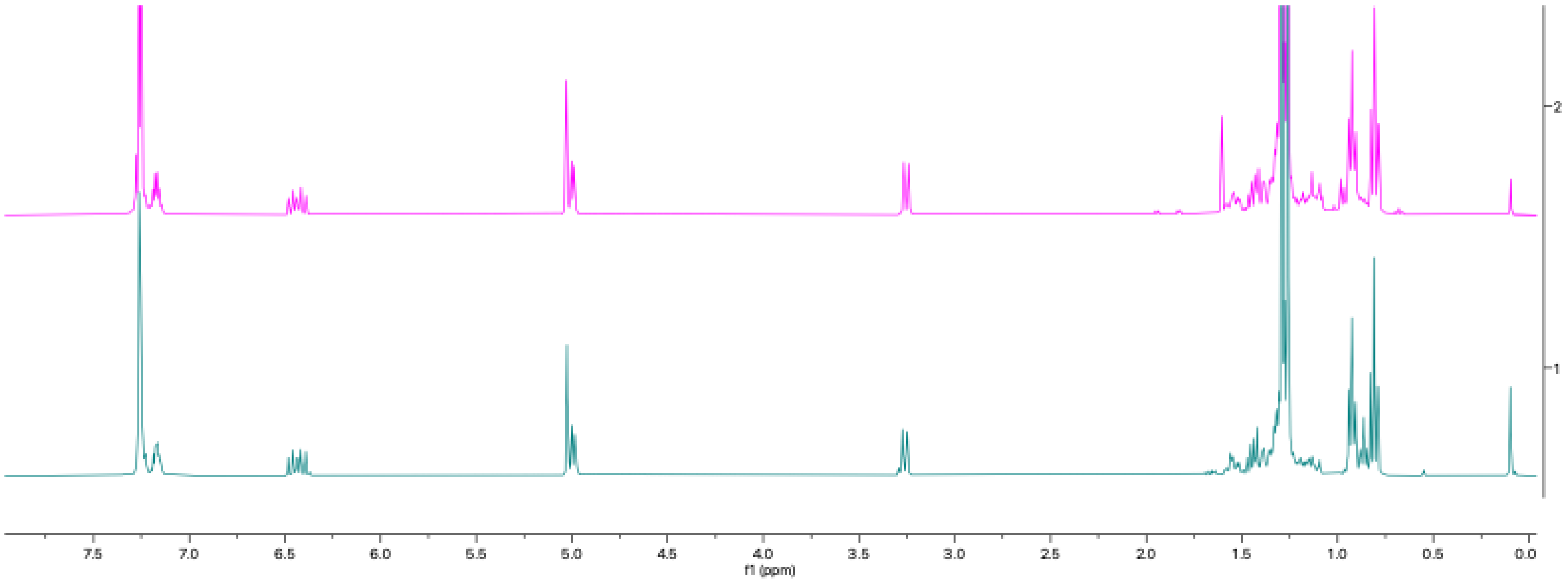

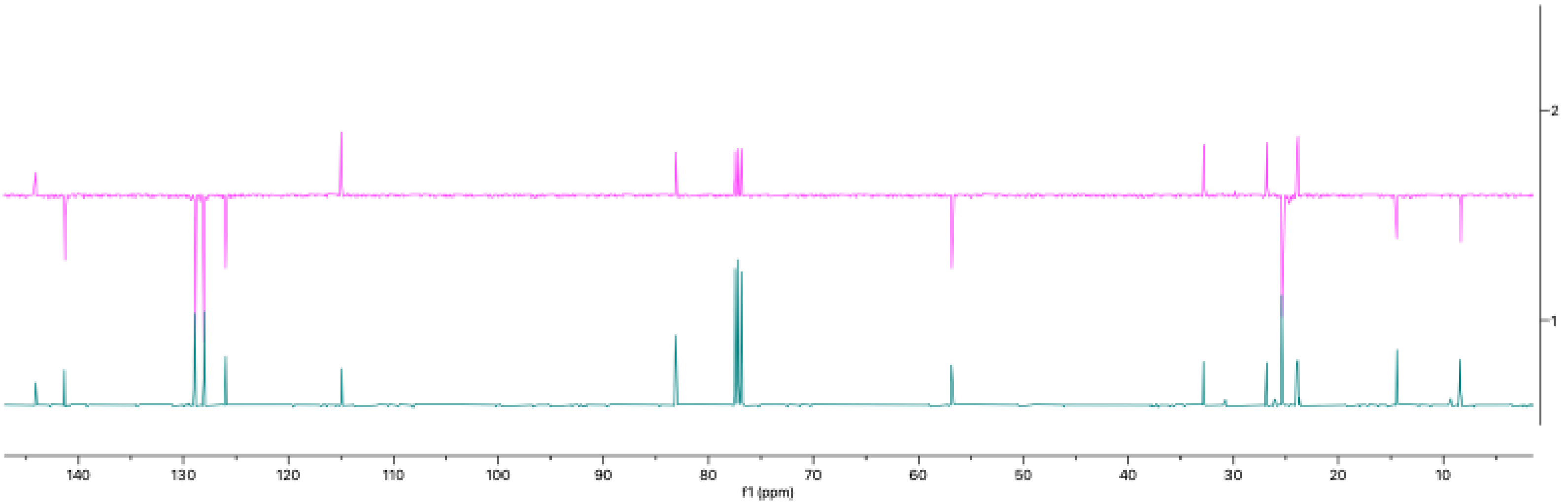

Stacked ^{1}H (top) and ^{13}C (bottom) NMR spectra of **7b** (pink) and the literature reference (teal).[3b]

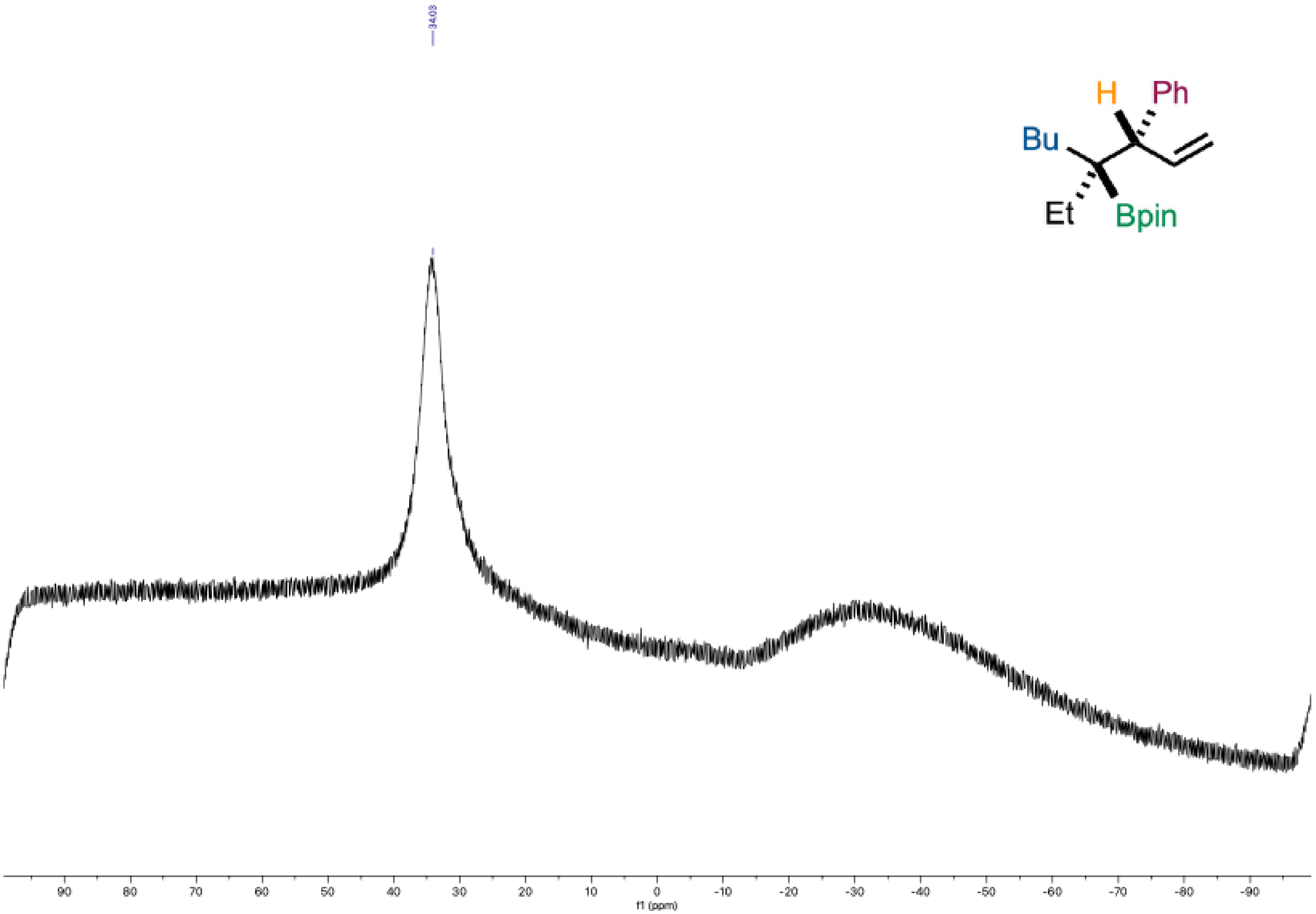

11B NMR spectrum (128 MHz, $CDCl_3$)

2-((3*R,4*R**)-4-Ethyl-6-methyl-3-phenylhept-1-en-4-yl)-4,4,5,5-tetramethyl-1,3,2-dioxaborolane 7c**

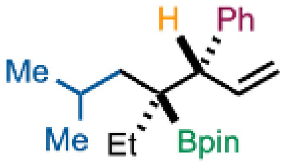

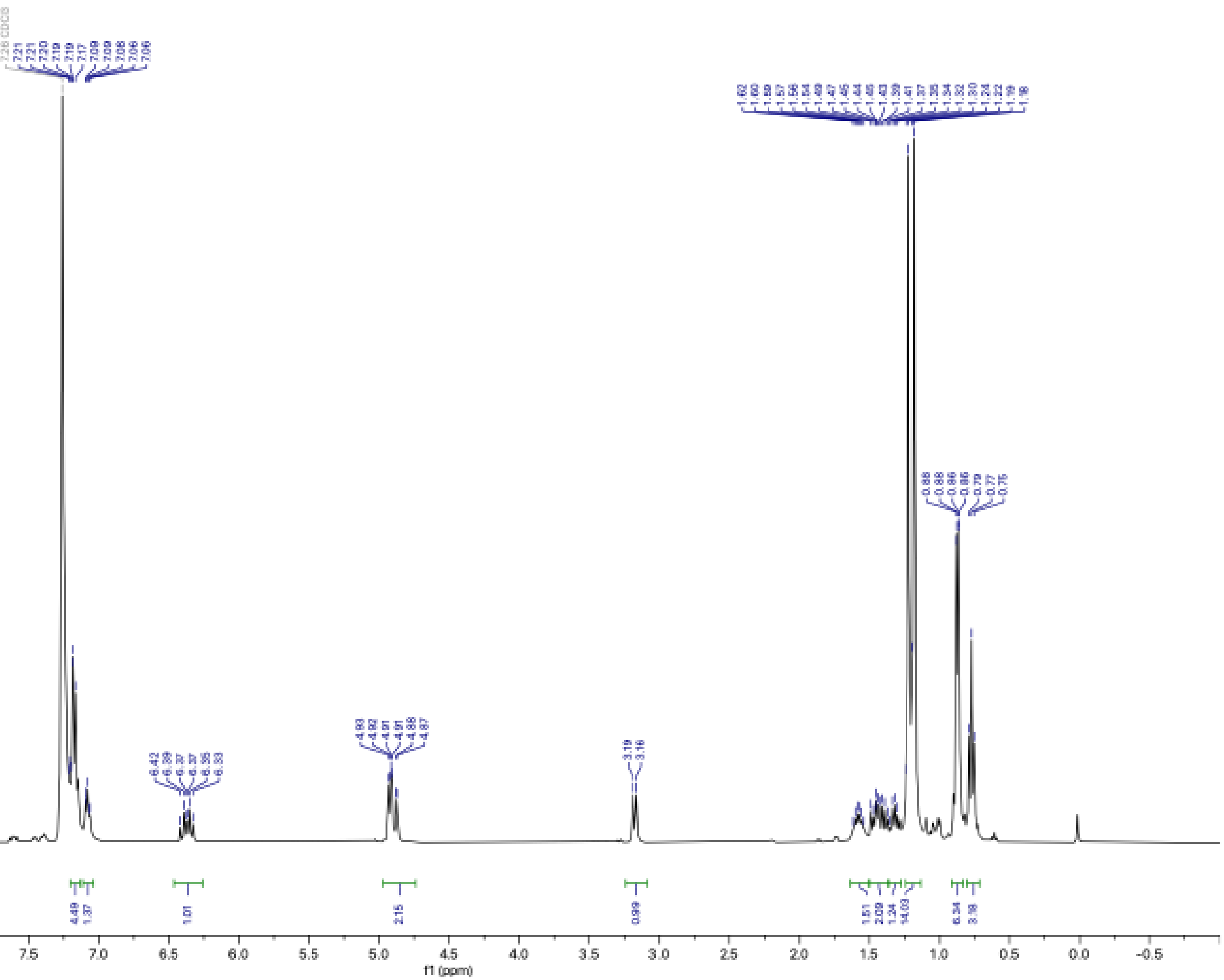

^{1}H NMR spectrum (400 MHz, $CDCl_3$)

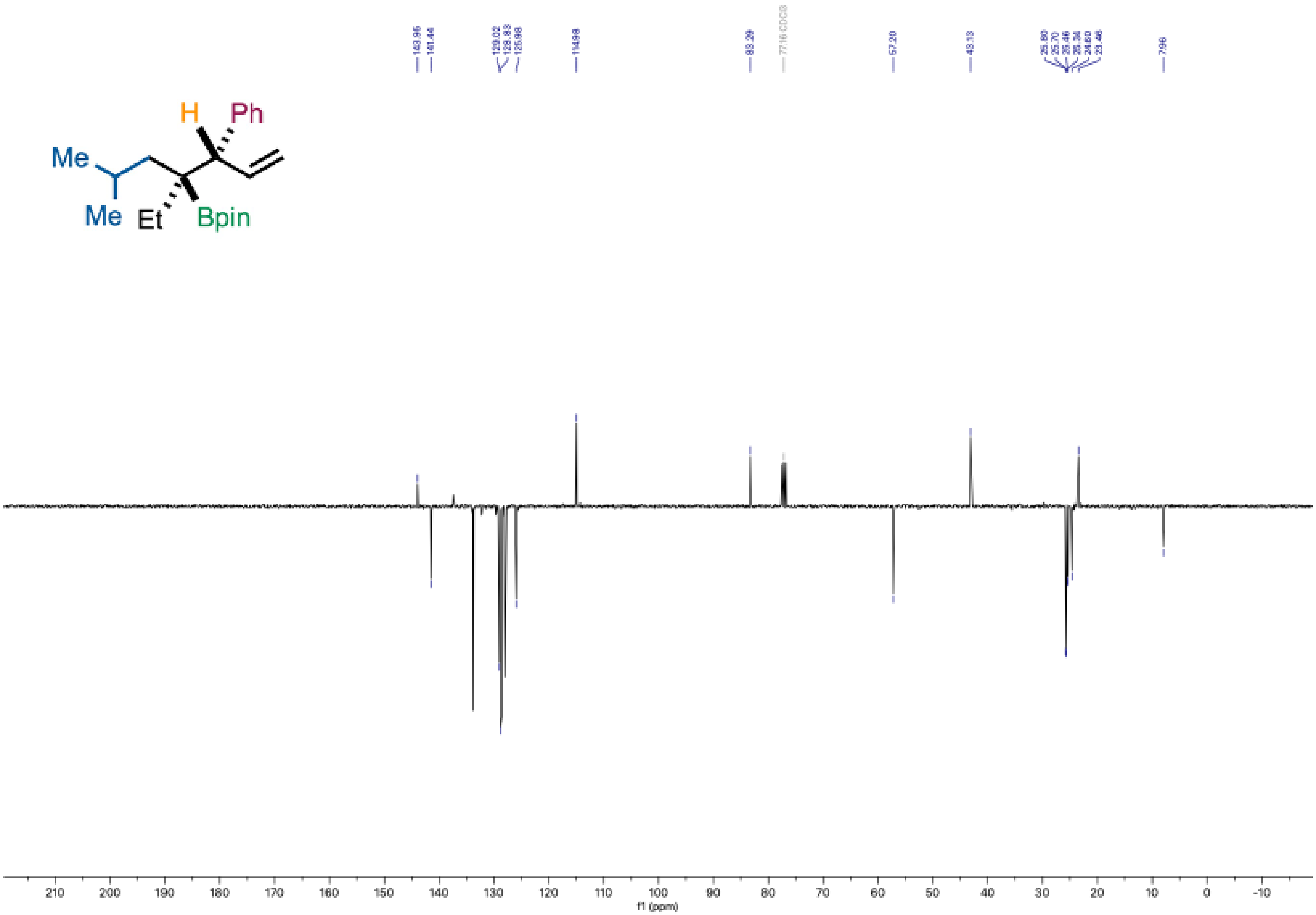

^{13}C NMR spectrum (101 MHz, $CDCl_3$)

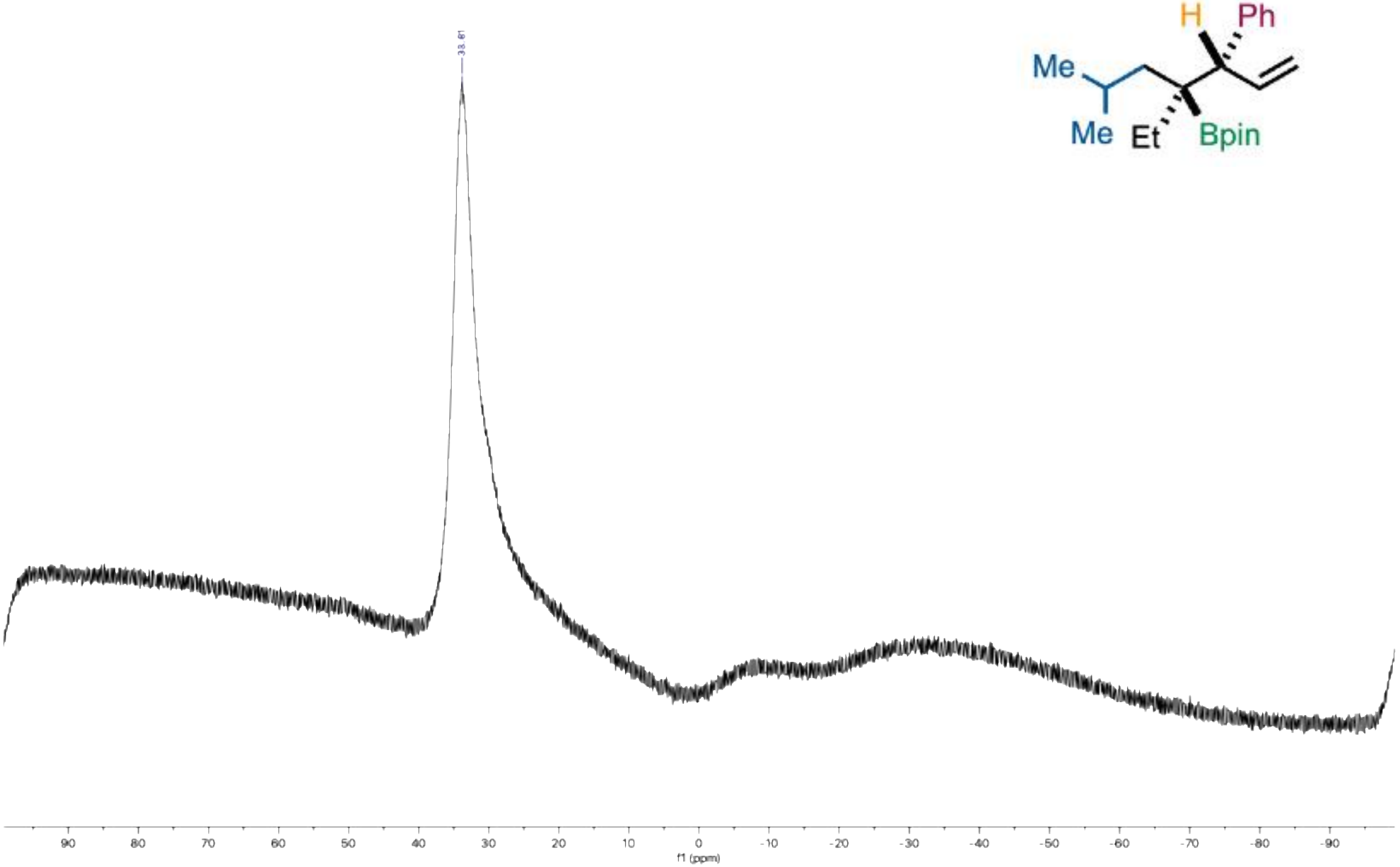

^{11}B NMR spectrum (128 MHz, $CDCl_3$)

4,4,5,5-Tetramethyl-2-((3*R*,4*R*)-4-methyl-3-phenyldodec-1-en-4-yl)-1,3,2-dioxaborolane **7d**

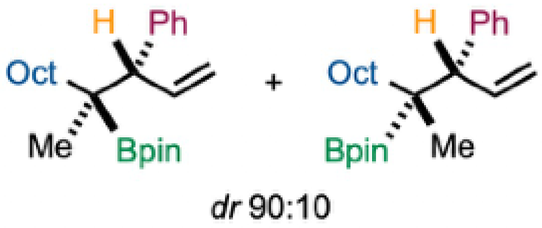

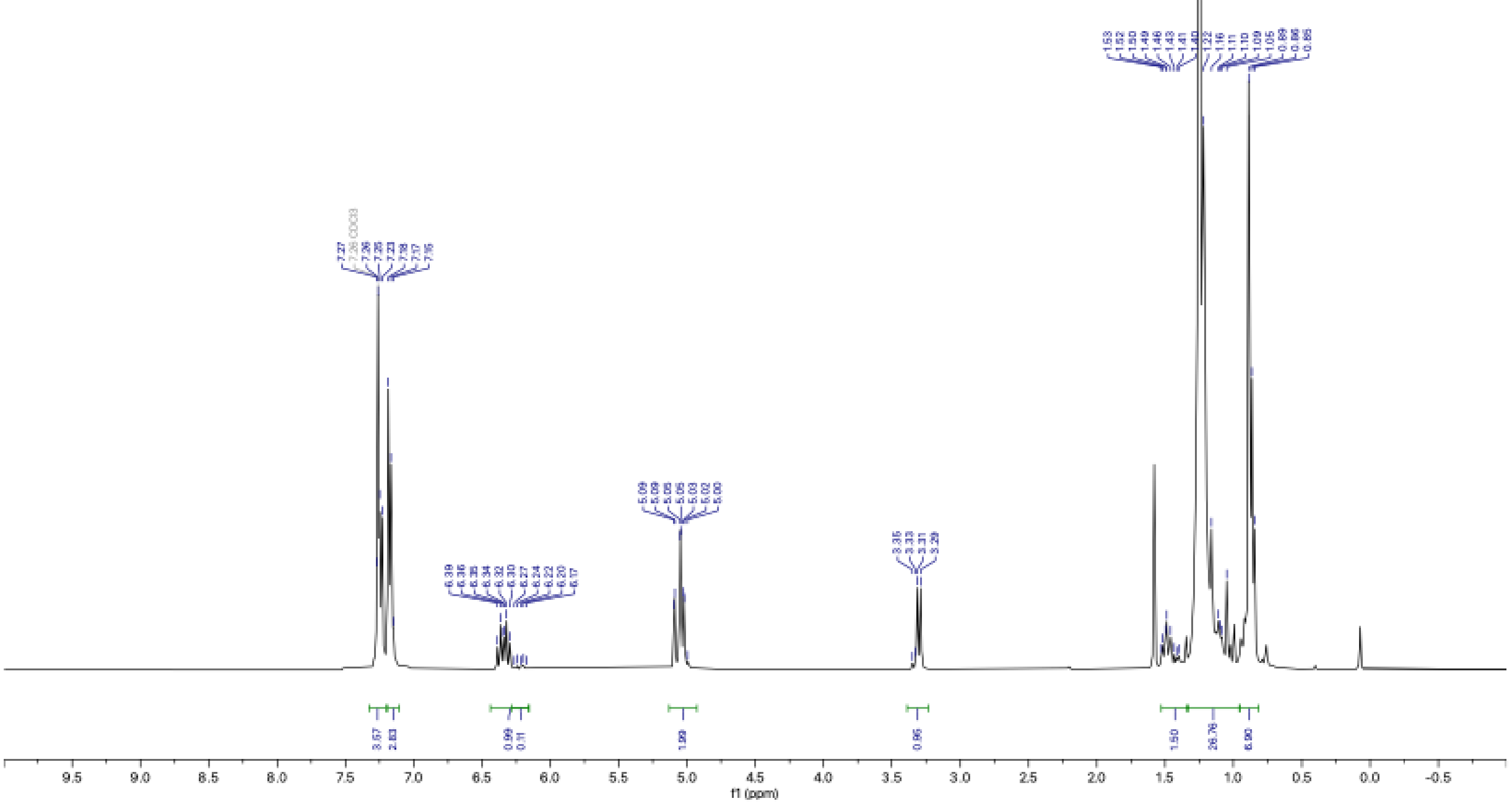

^{1}H NMR spectrum (400 MHz, $CDCl_3$)

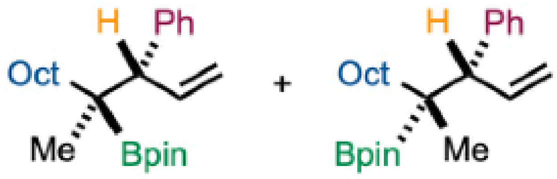

dr 90:10

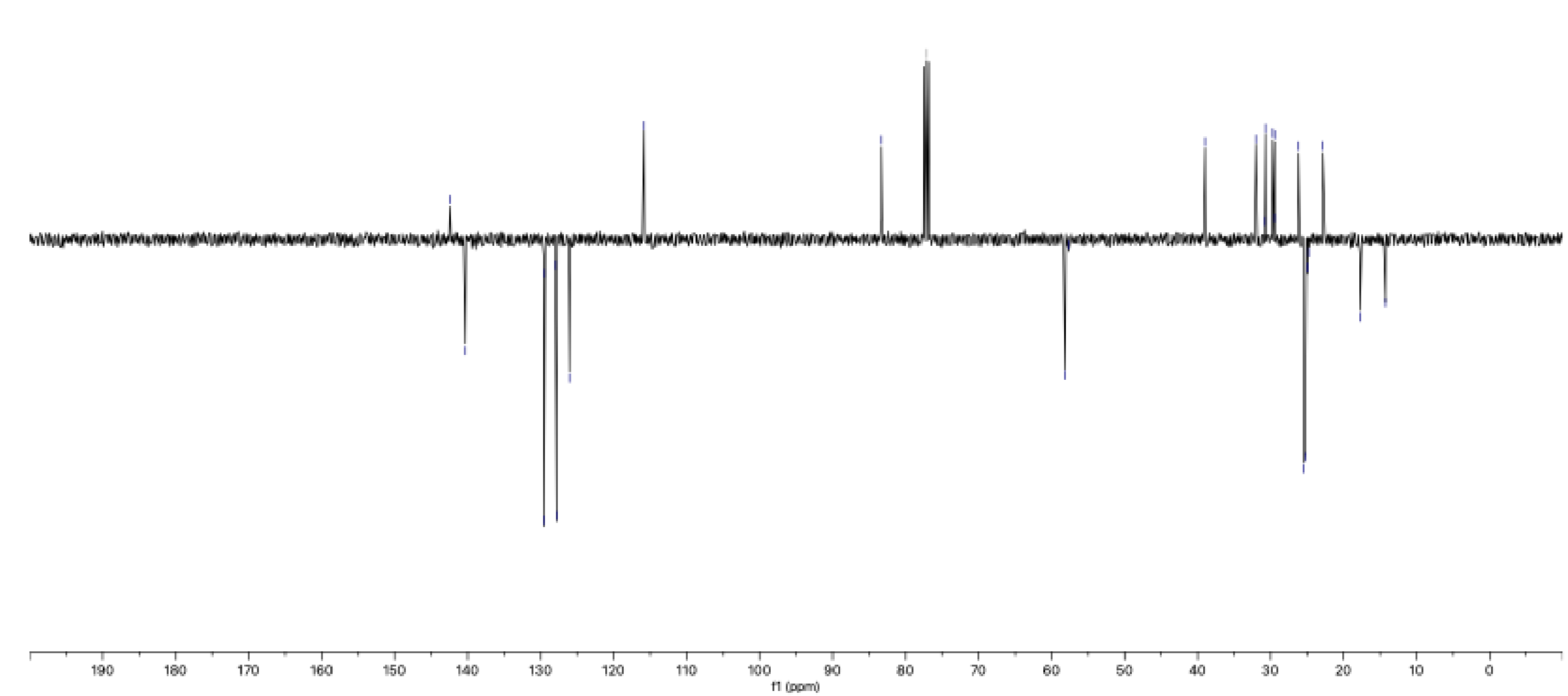

^{13}**C NMR** spectrum (101 MHz, $CDCl_3$)

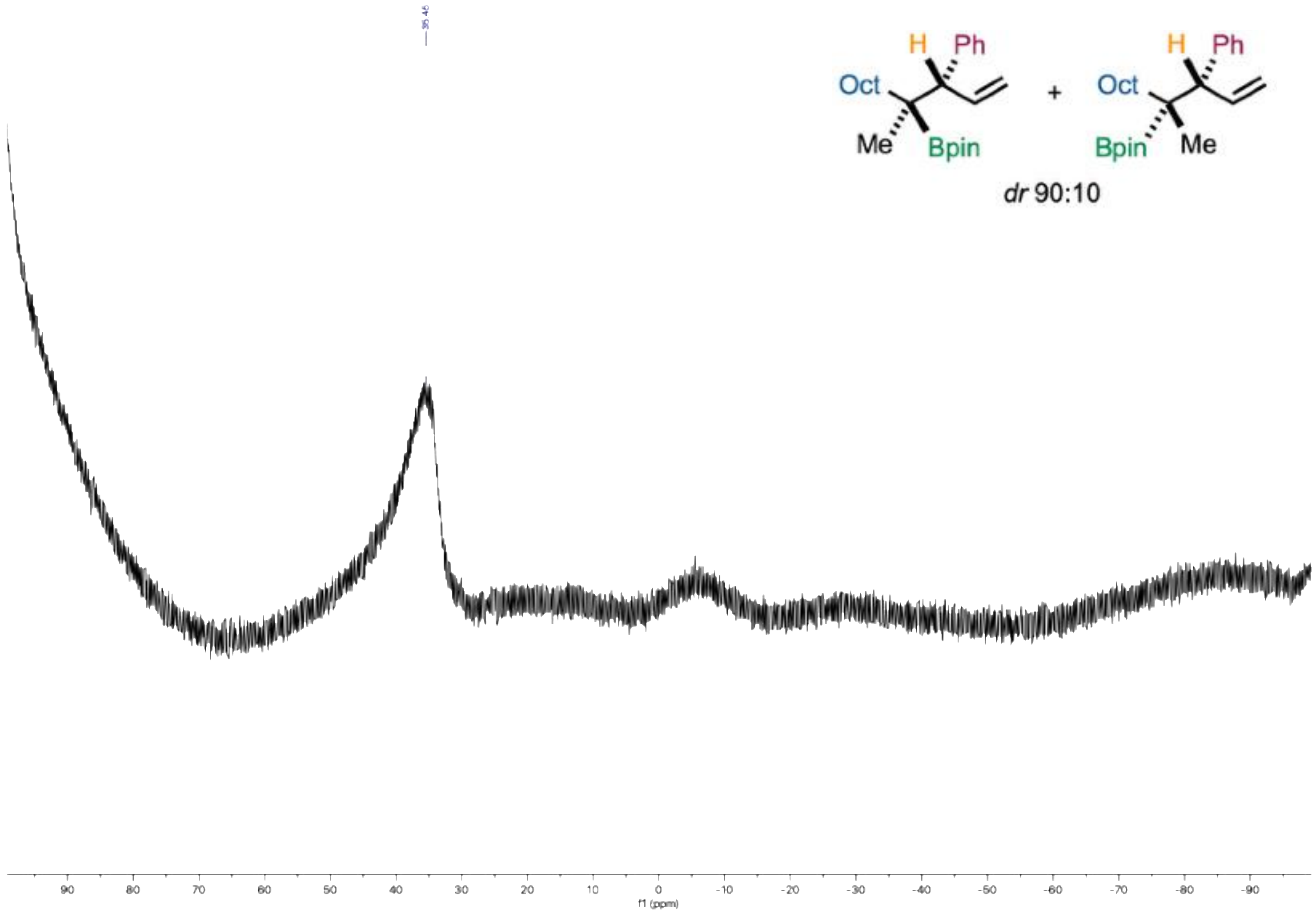

^{11}B NMR spectrum (128 MHz, $CDCl_3$)

4,4,5,5-Tetramethyl-2-((3*R,4*R**)-4-methyl-7-phenyl-3-(*p*-tolyl)hept-1-en-4-yl)-1,3,2-dioxaborolane 7e**

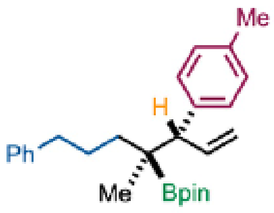

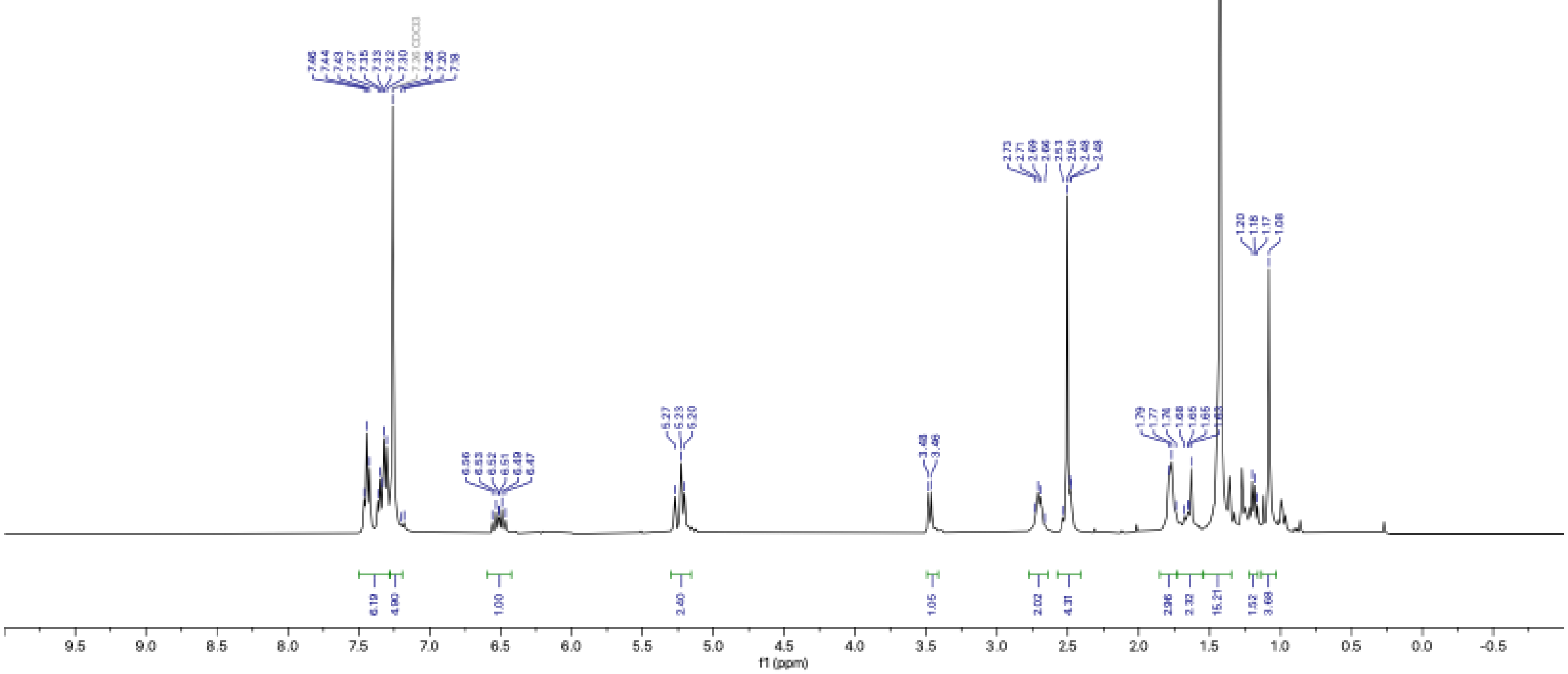

^{1}H NMR spectrum (400 MHz, $CDCl_3$)

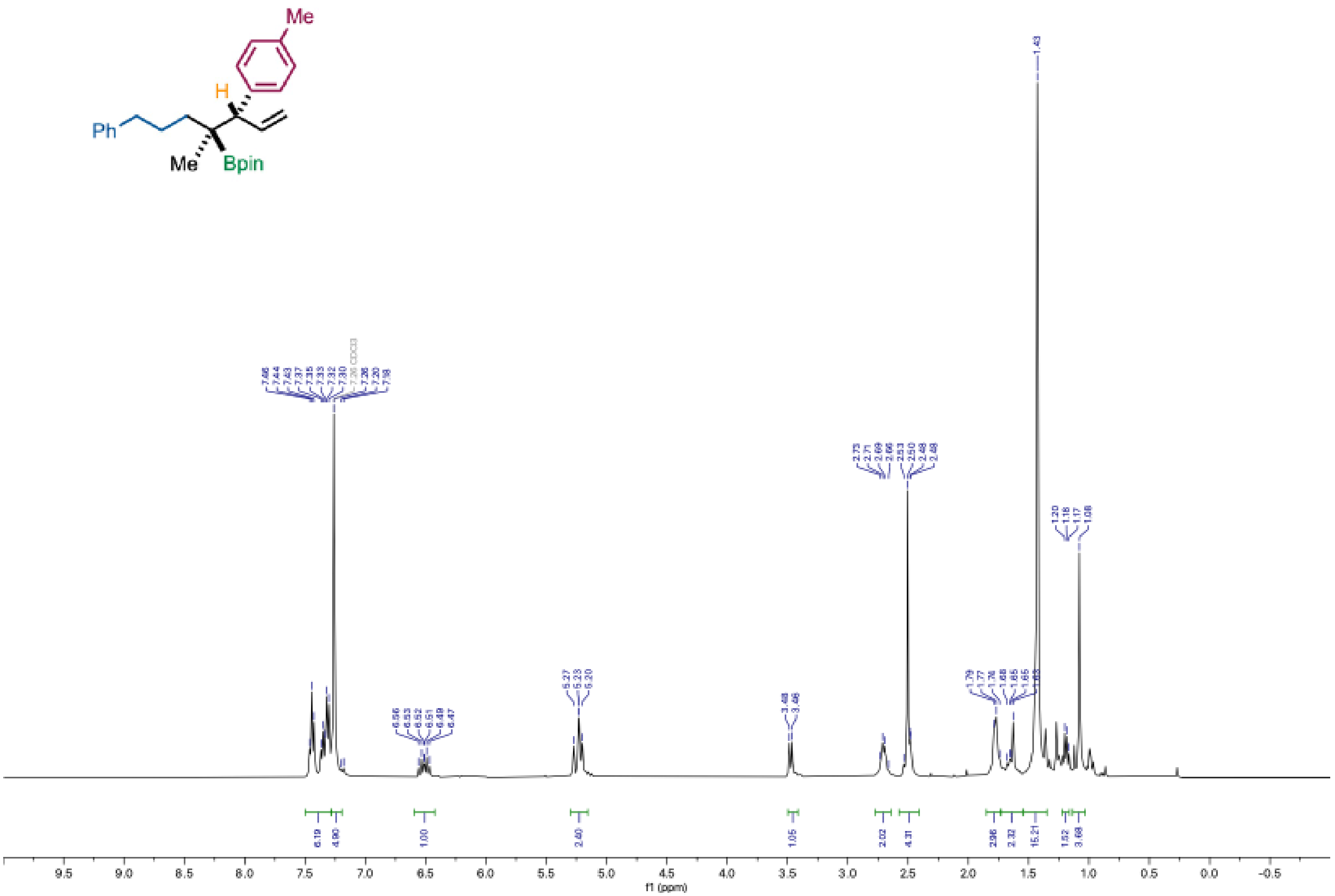

^{13}C NMR spectrum (101 MHz, $CDCl_3$)

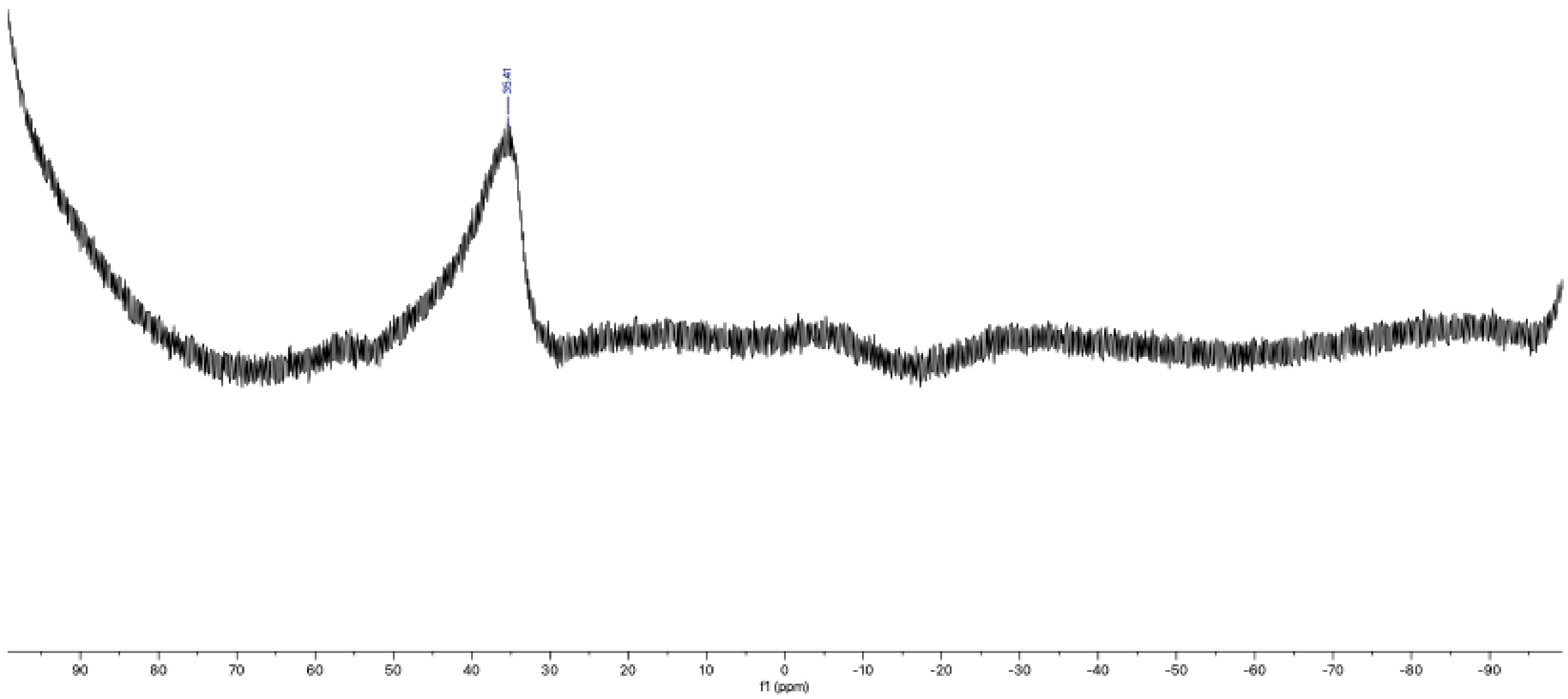

^{11}B NMR spectrum (128 MHz, $CDCl_3$)

4,4,5,5-Tetramethyl-2-((3*R,4*R**)-4-methyl-3-(*p*-tolyl)oct-1-en-4-yl)-1,3,2-dioxaborolane 7f**

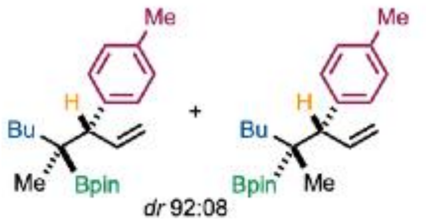

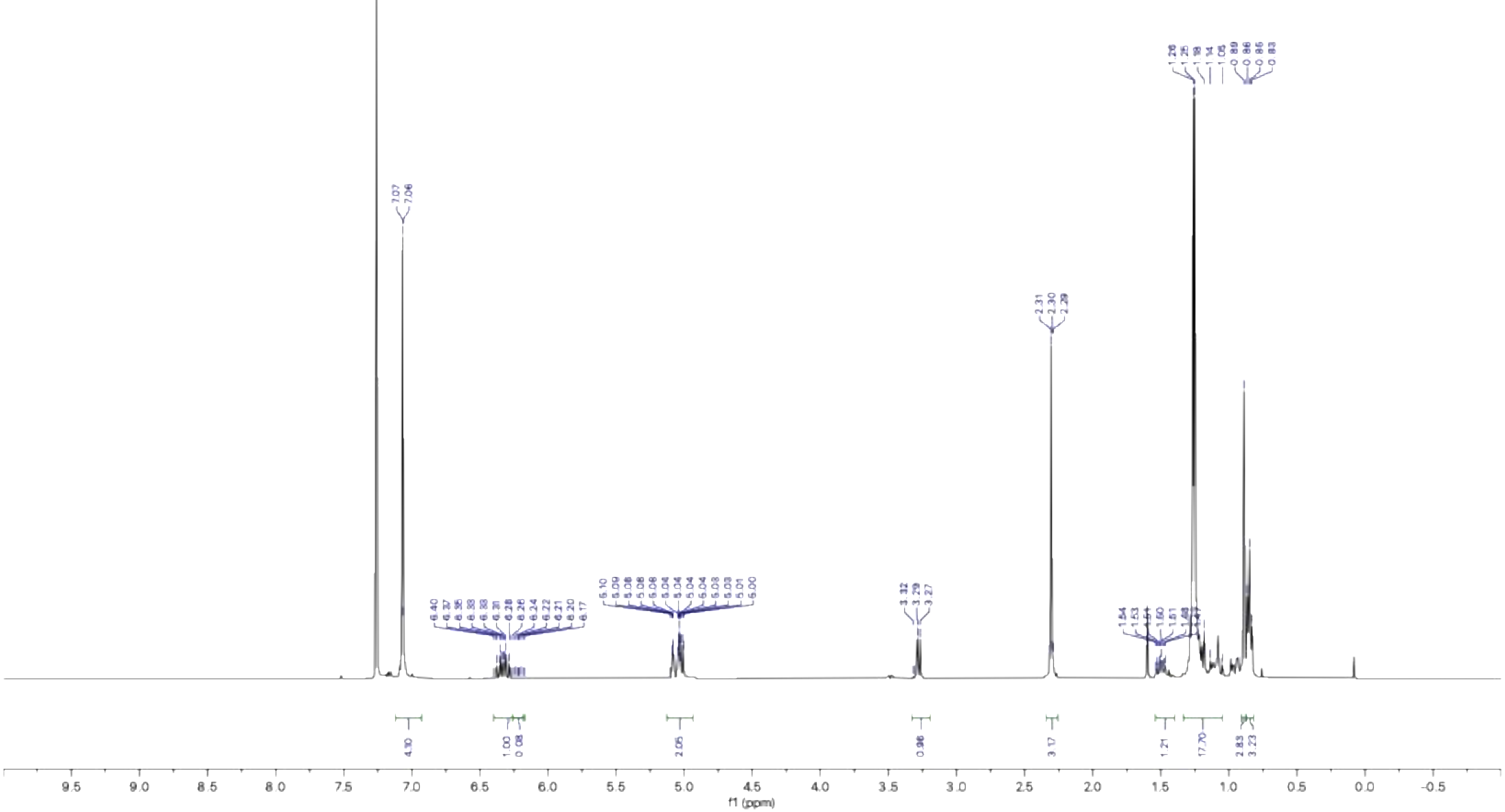

^{1}H NMR spectrum (400 MHz, $CDCl_3$)

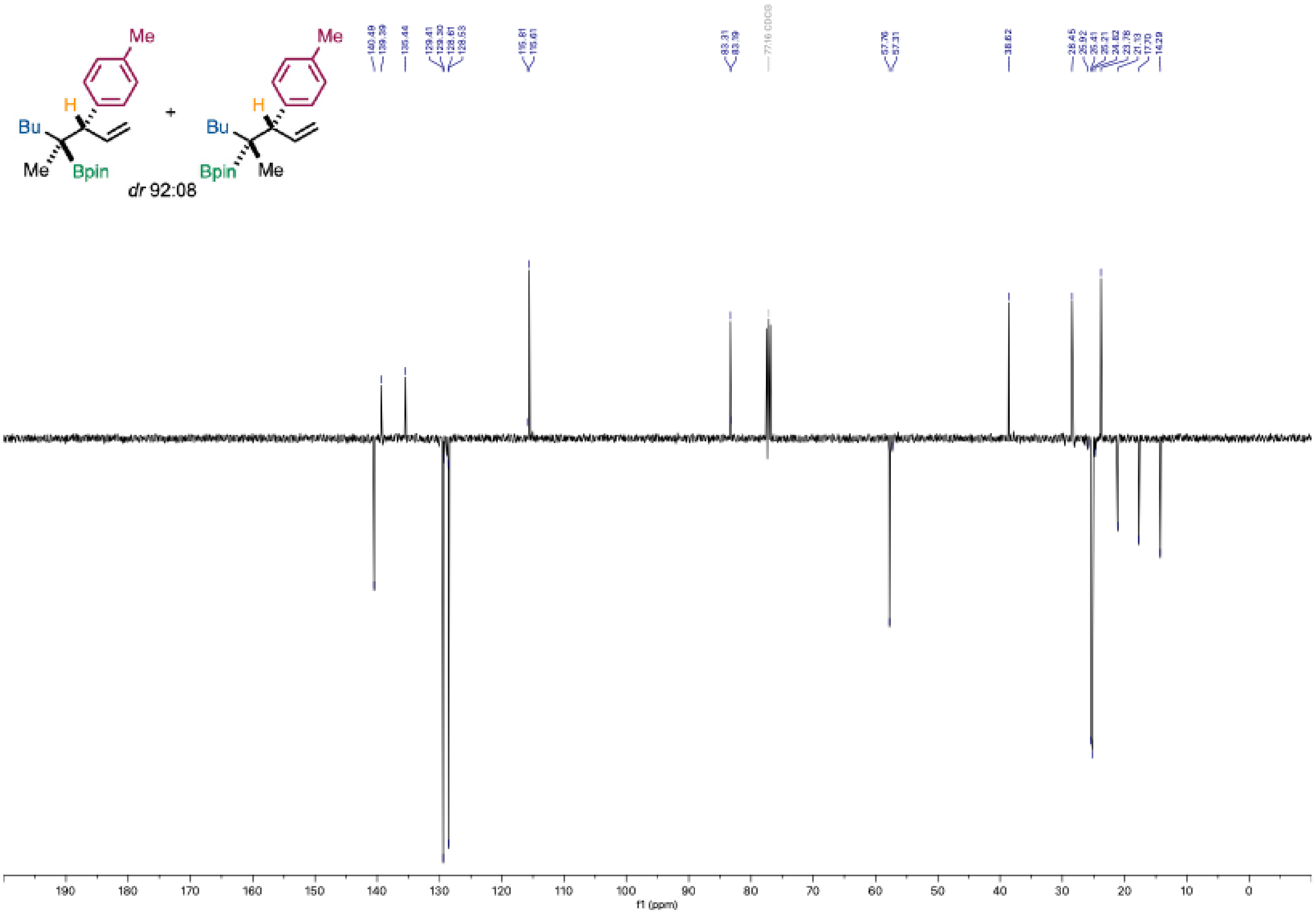

^{13}C NMR spectrum (101 MHz, $CDCl_3$)

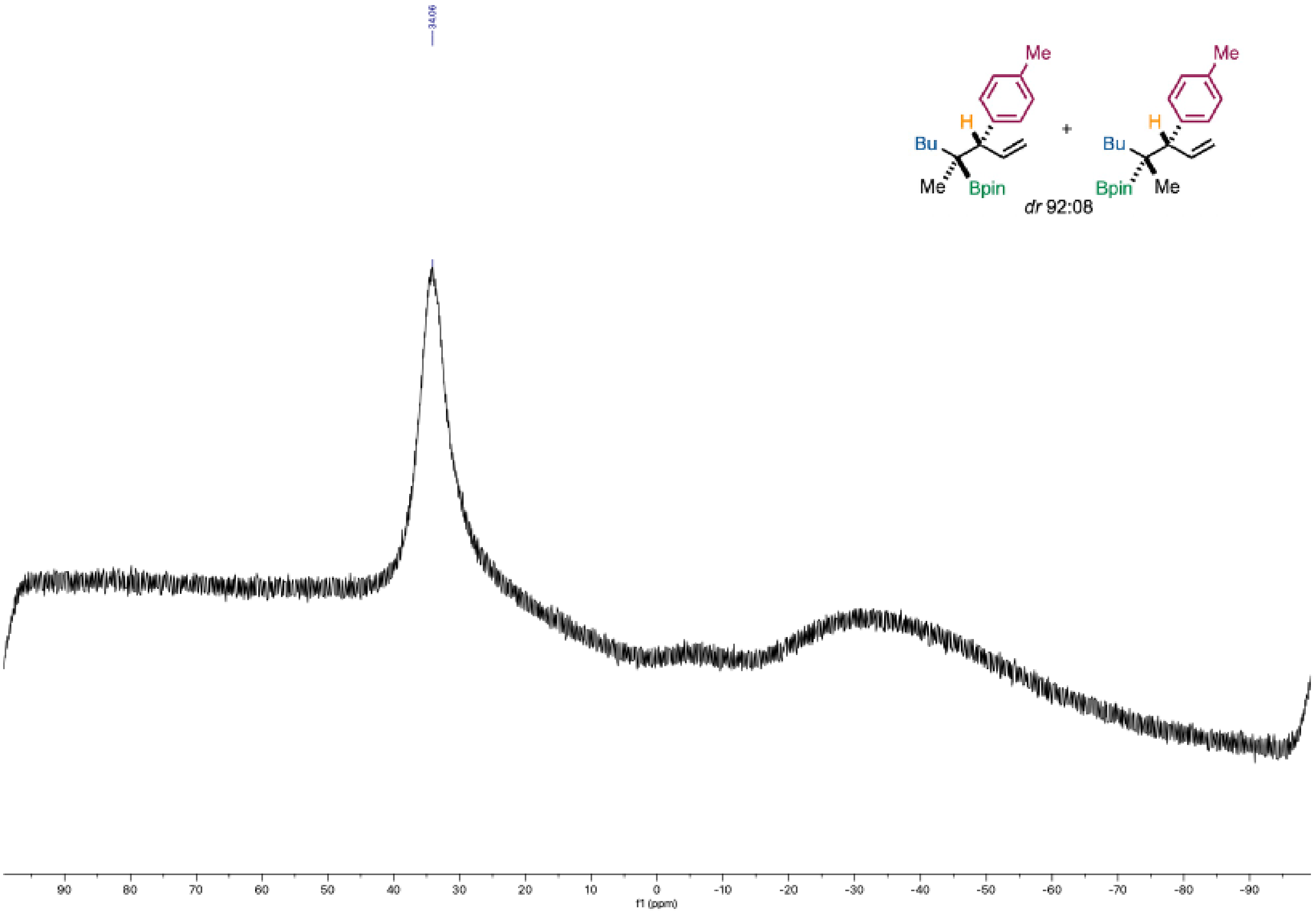

^{11}B NMR spectrum (128 MHz, $CDCl_3$)

4,4,5,5-Tetramethyl-2-((3*R,4*R**)-4-methyl-3-(*p*-tolyl)oct-tetradec-1-en-4-yl)-1,3,2-dioxaborolane 7g**

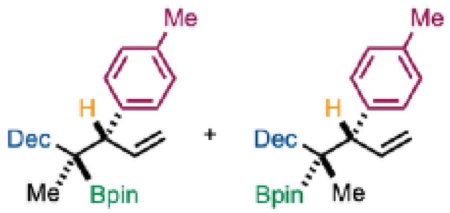

dr 89:11

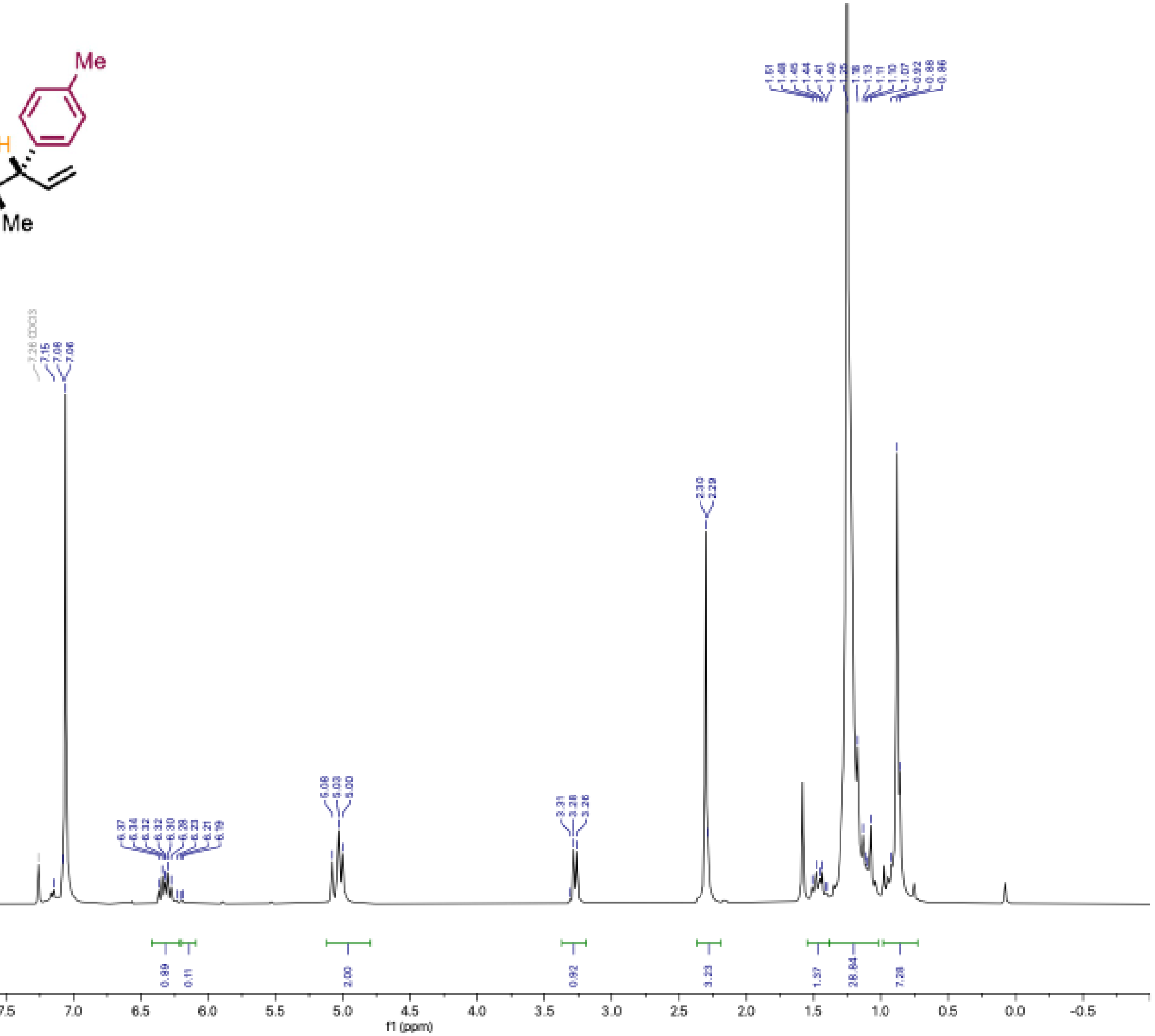

[1]**H NMR** spectrum (400 MHz, $CDCl_3$)

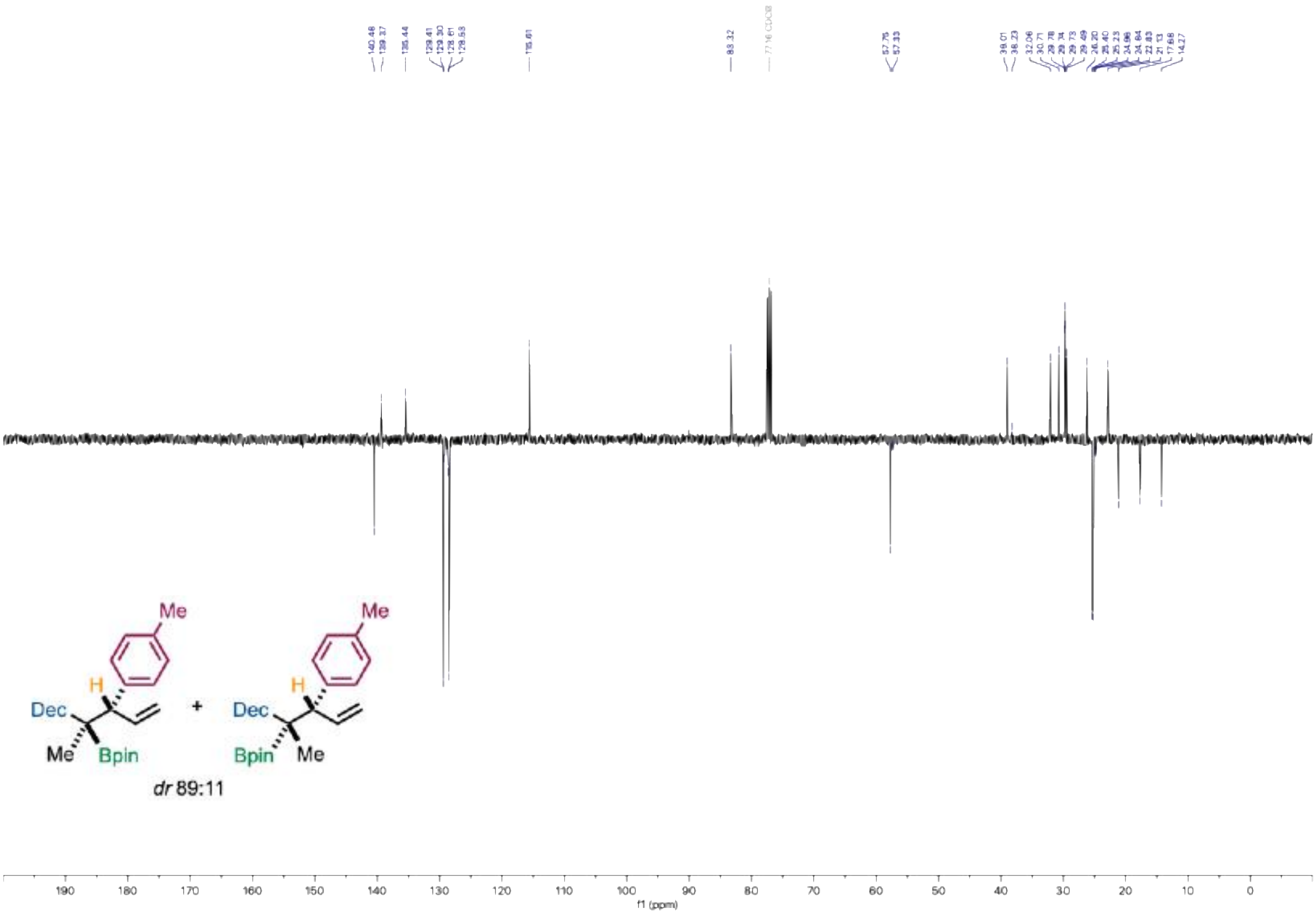

^{13}C NMR spectrum (101 MHz, $CDCl_3$)

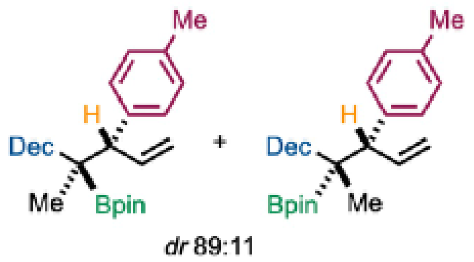

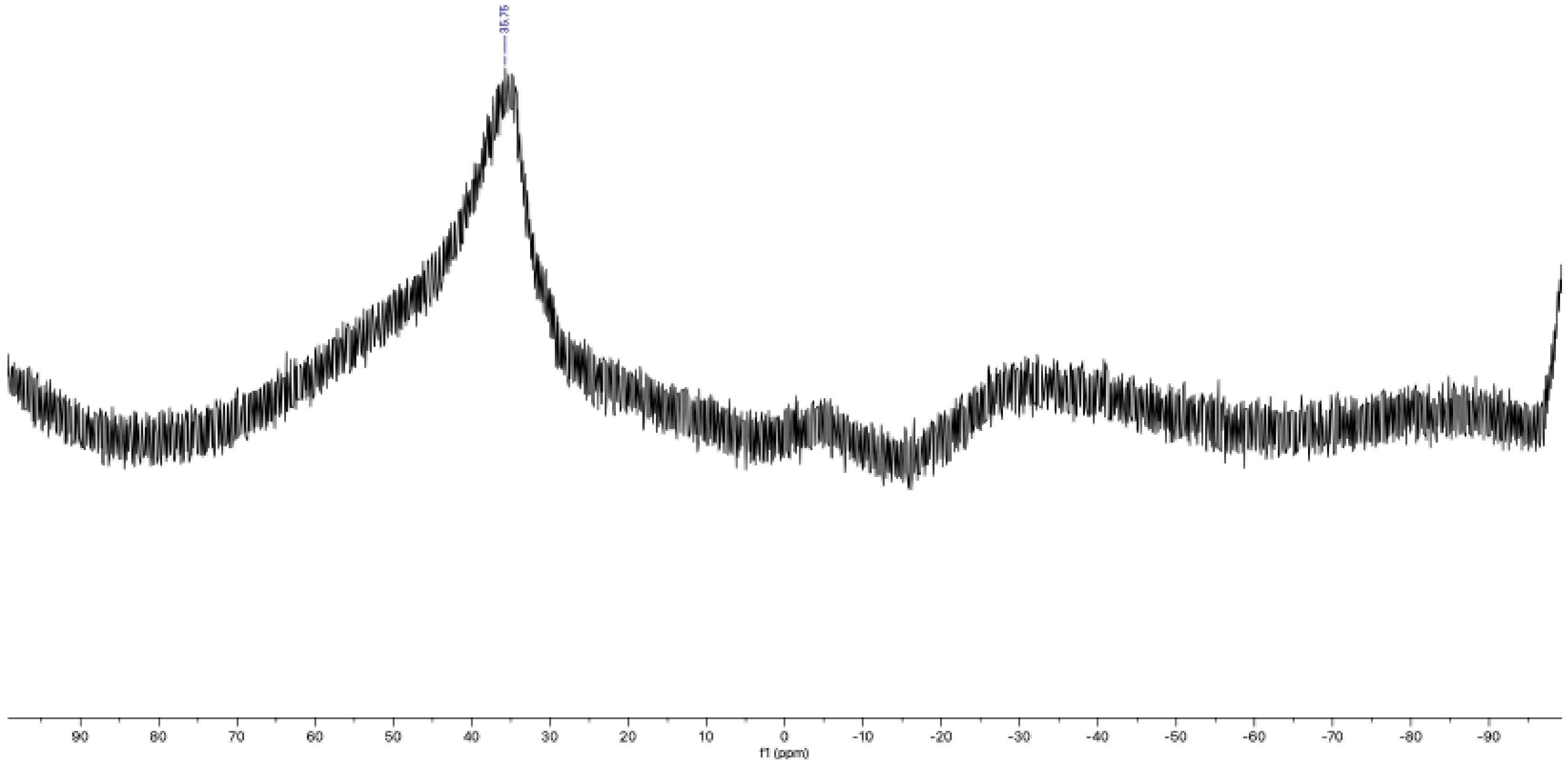

^{11}**B NMR** spectrum (128 MHz, $CDCl_3$)

4,4,5,5-Tetramethyl-2-((3*R**,4*R**)-4-methyl-3-(4-(trifluoromethyl)phenyl)tetradec-1-en-4-yl)-1,3,2-dioxaborolane 7h

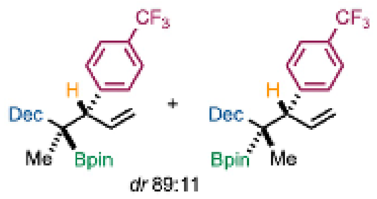

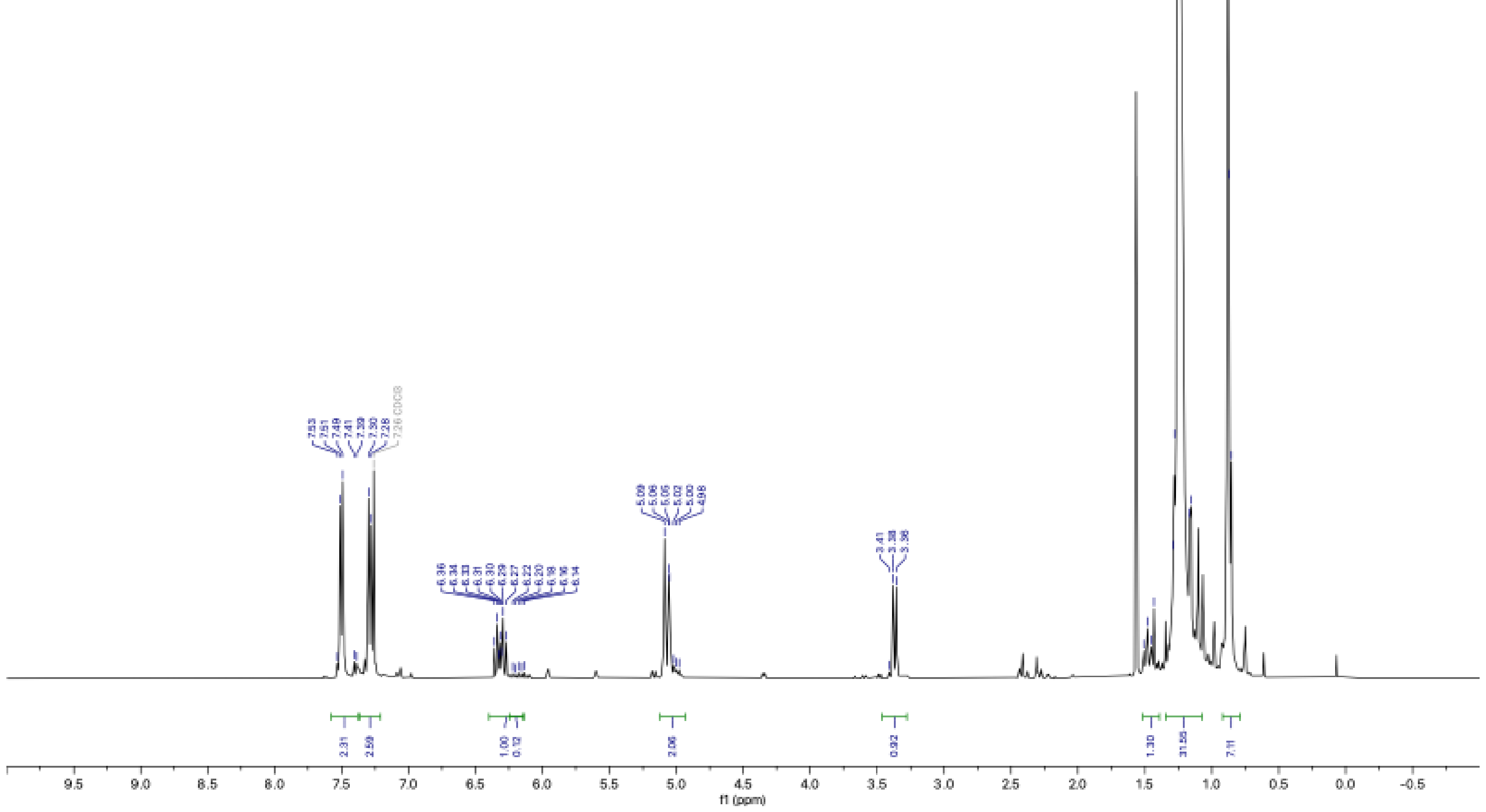

[1]**H NMR** spectrum (400 MHz, $CDCl_3$)

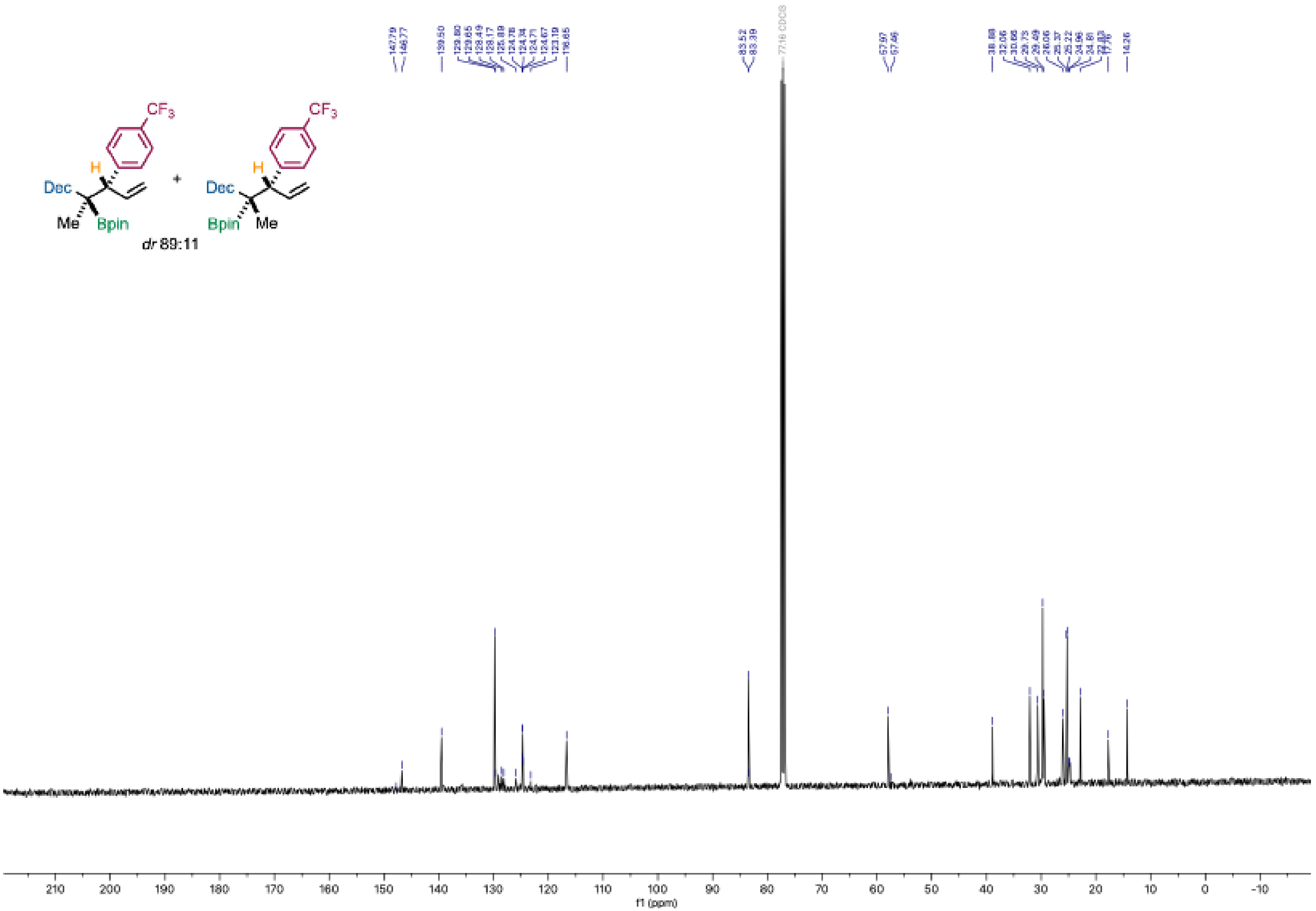

^{13}**C NMR** spectrum (101 MHz, $CDCl_3$)

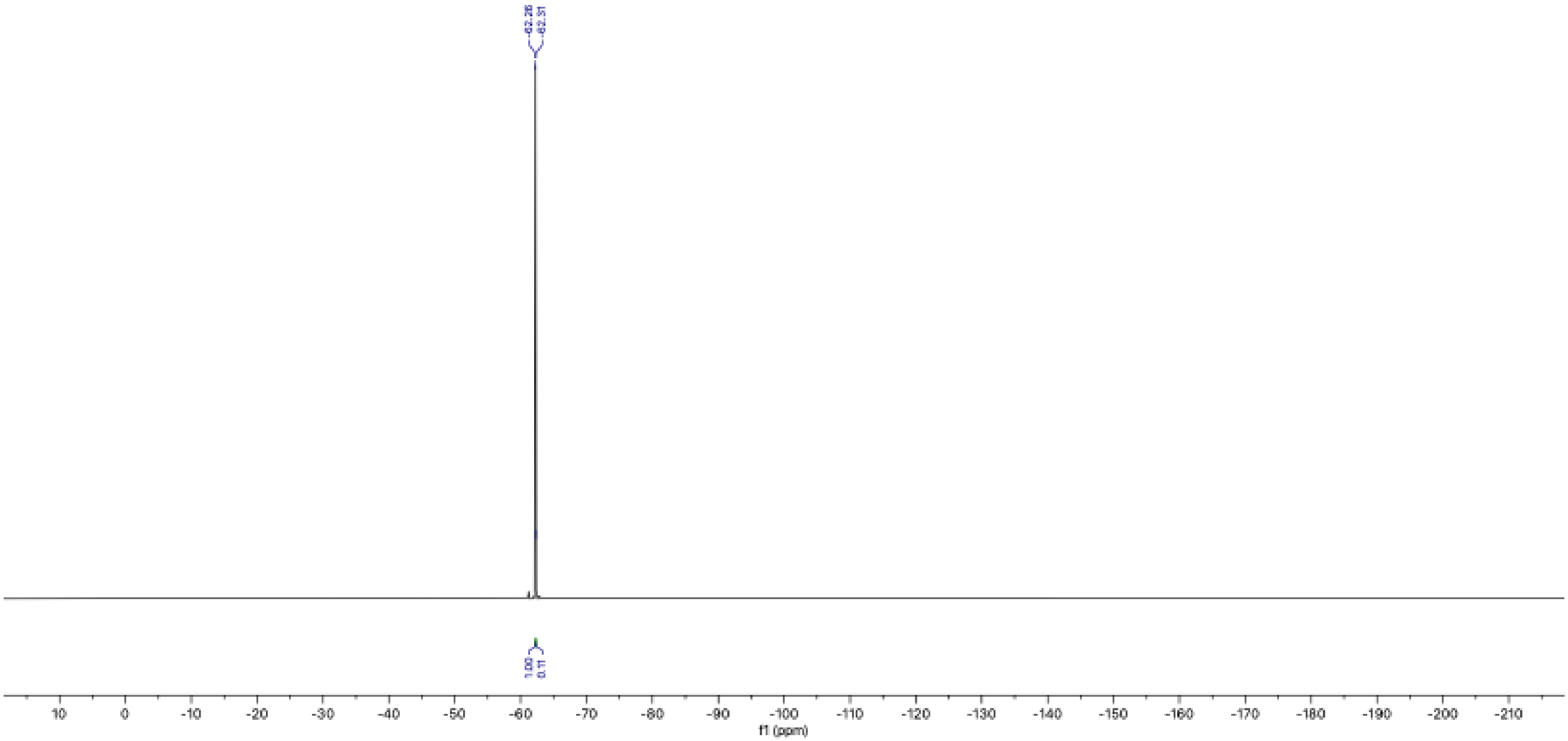

^{19}F NMR spectrum (377 MHz, $CDCl_3$)

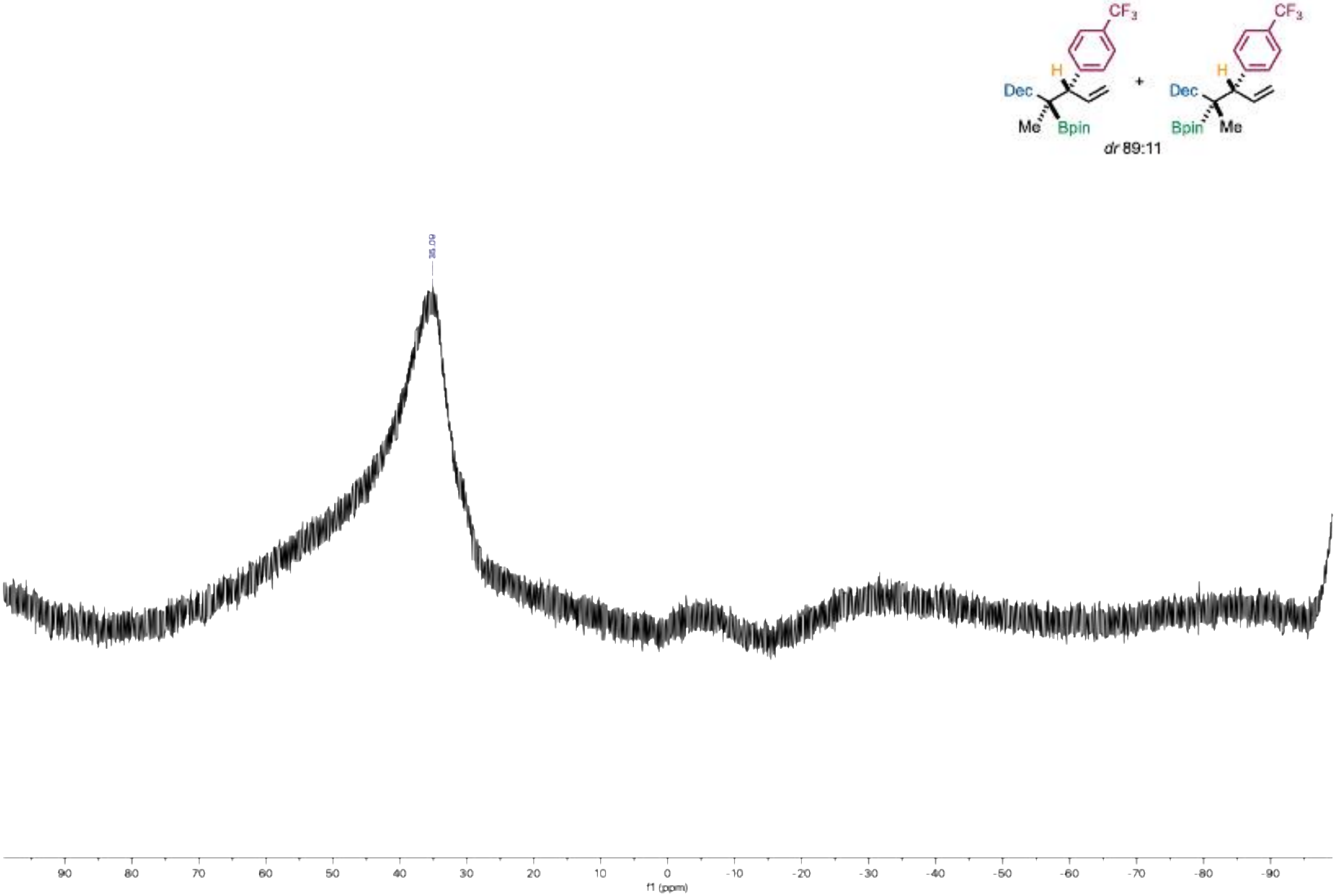

^{11}B NMR spectrum (128 MHz, $CDCl_3$)

2-((3*R,4*R**)-3-(4-Chlorophenyl)-4-(3-phenylpropyl)oct-1-en-4-yl)-4,4,5,5-tetramethyl-1,3,2-dioxaborolane 7i**

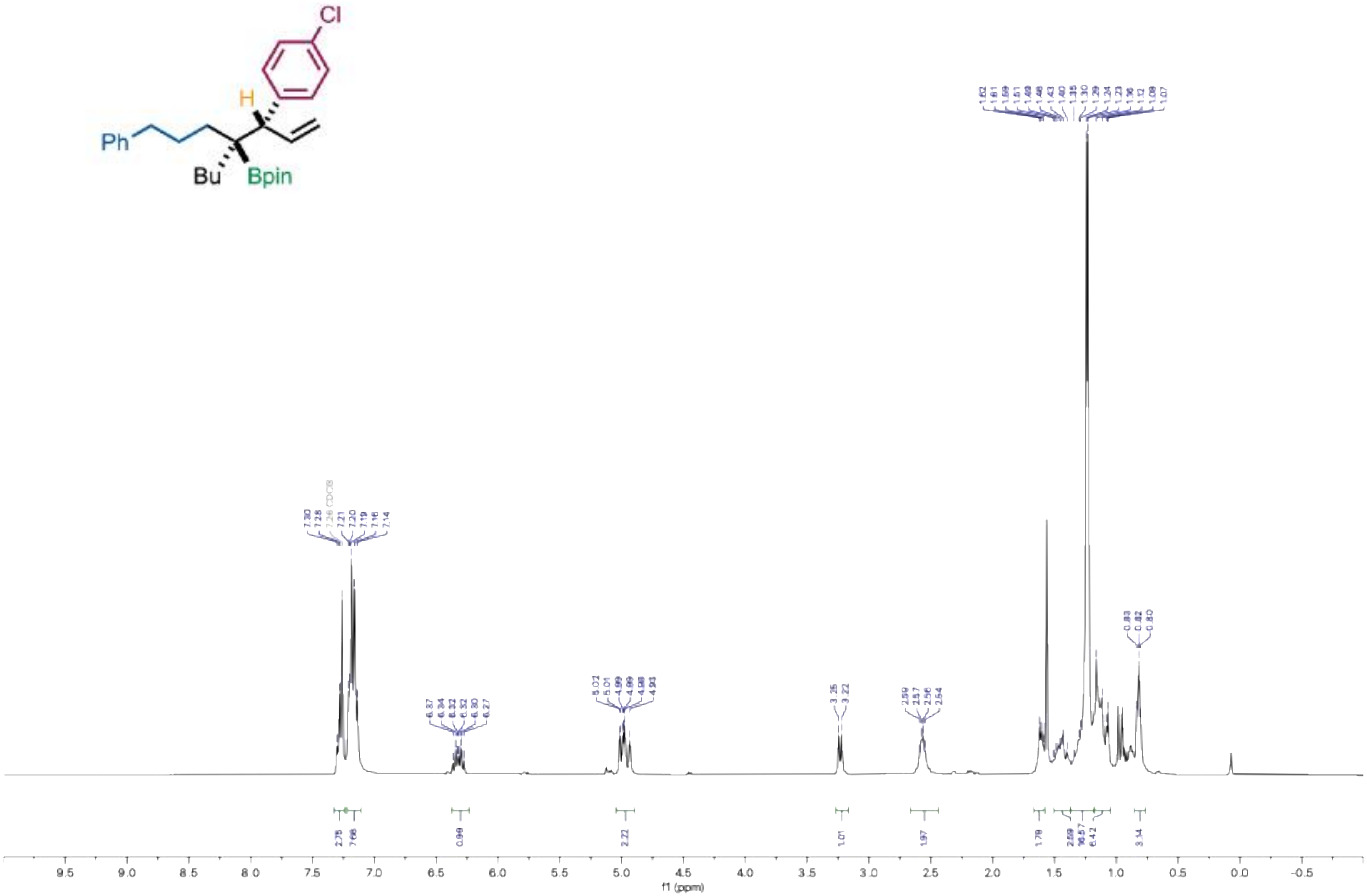

^{1}**H NMR** spectrum (400 MHz, $CDCl_3$)

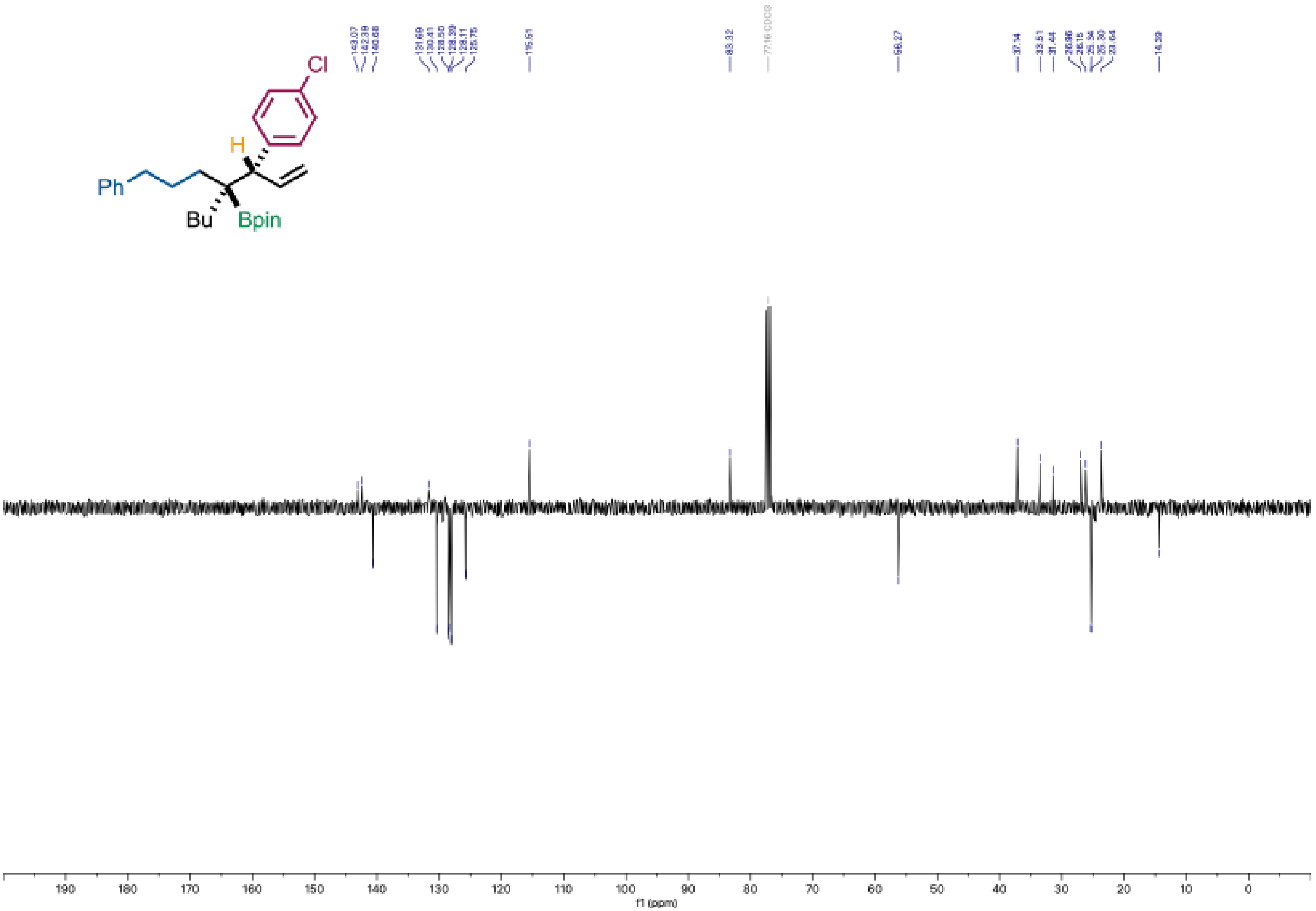

^{13}C NMR spectrum (101 MHz, $CDCl_3$)

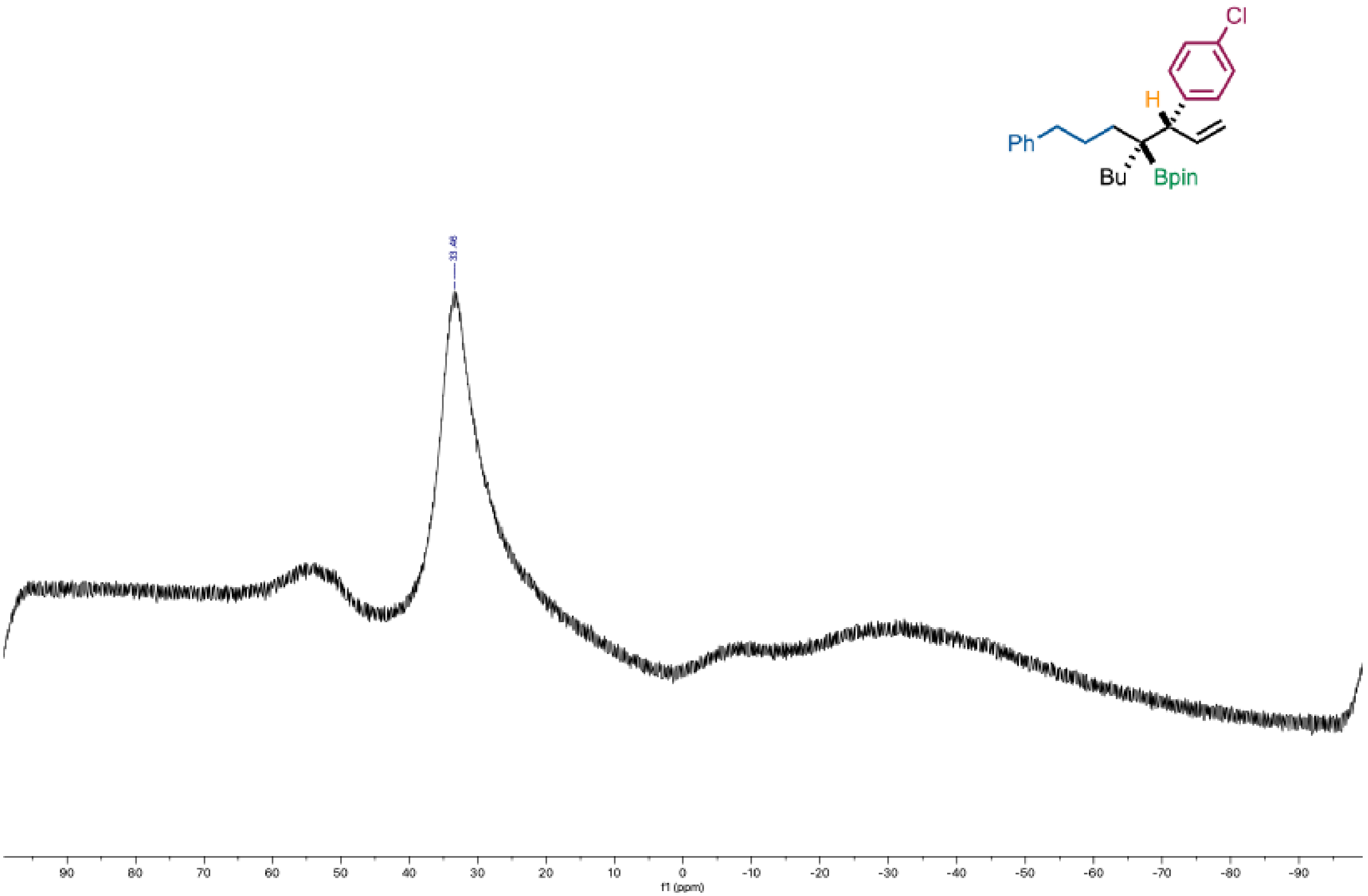

^{11}B NMR spectrum (128 MHz, $CDCl_3$)

2-((3*R,4*S**)-3-(4-Chlorophenyl)-4-methyloct-1-en-4-yl)-4,4,5,5-tetramethyl-1,3,2-dioxaborolane 7j**

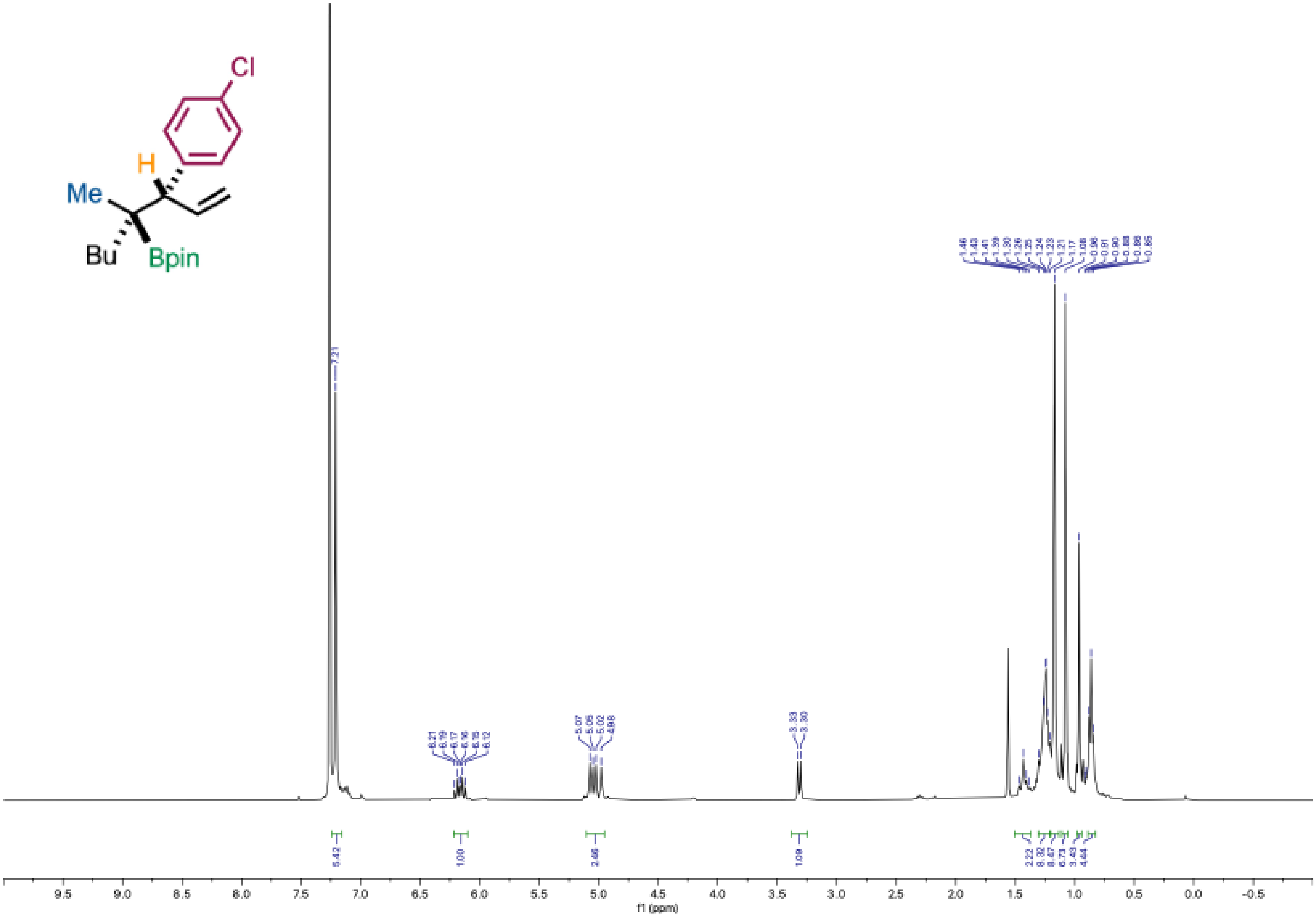

^{1}H NMR spectrum (400 MHz, $CDCl_3$)

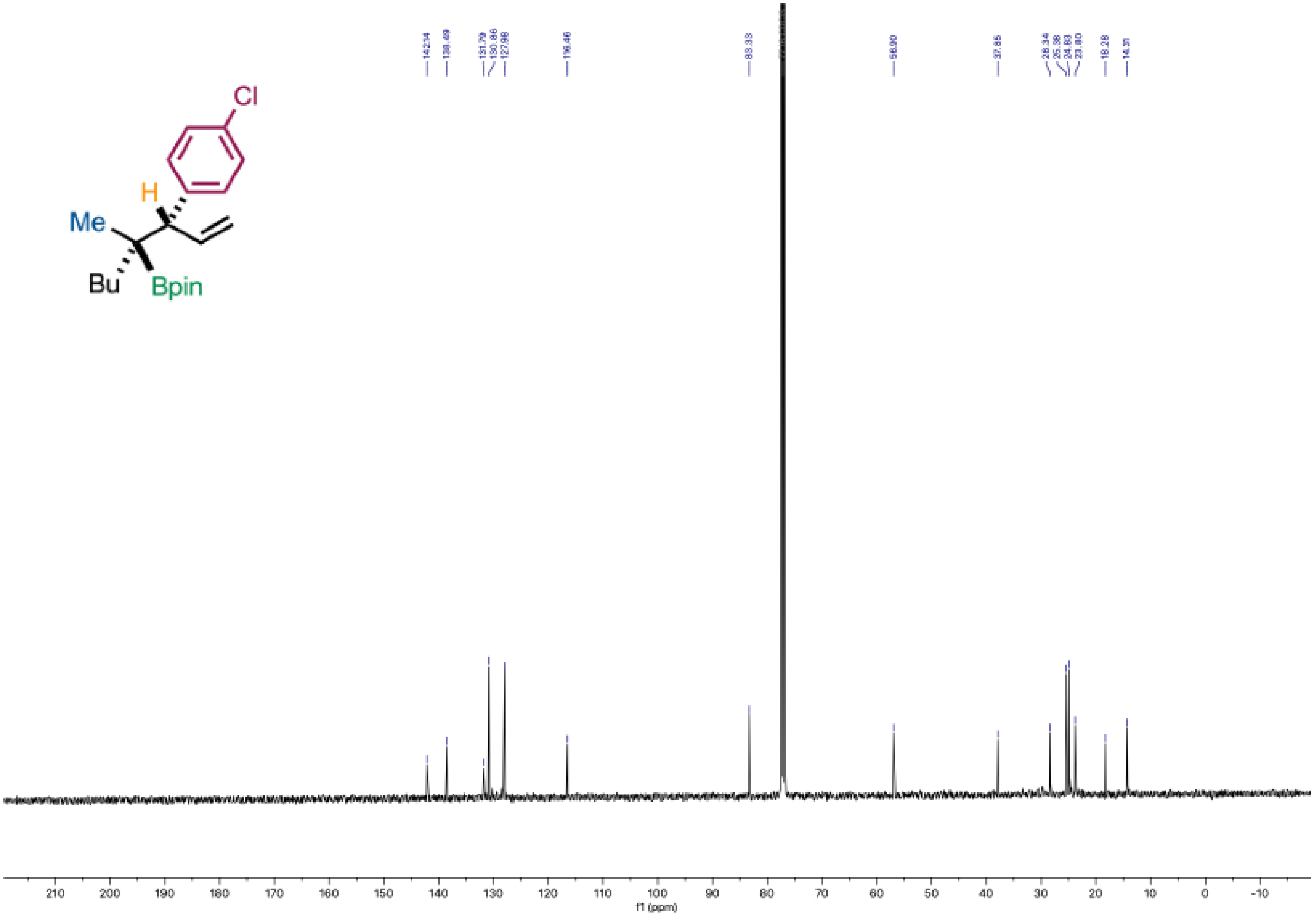

^{13}**C NMR** spectrum (101 MHz, $CDCl_3$)

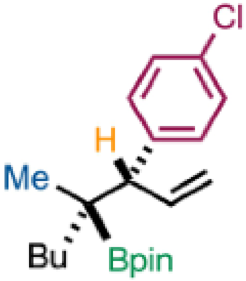

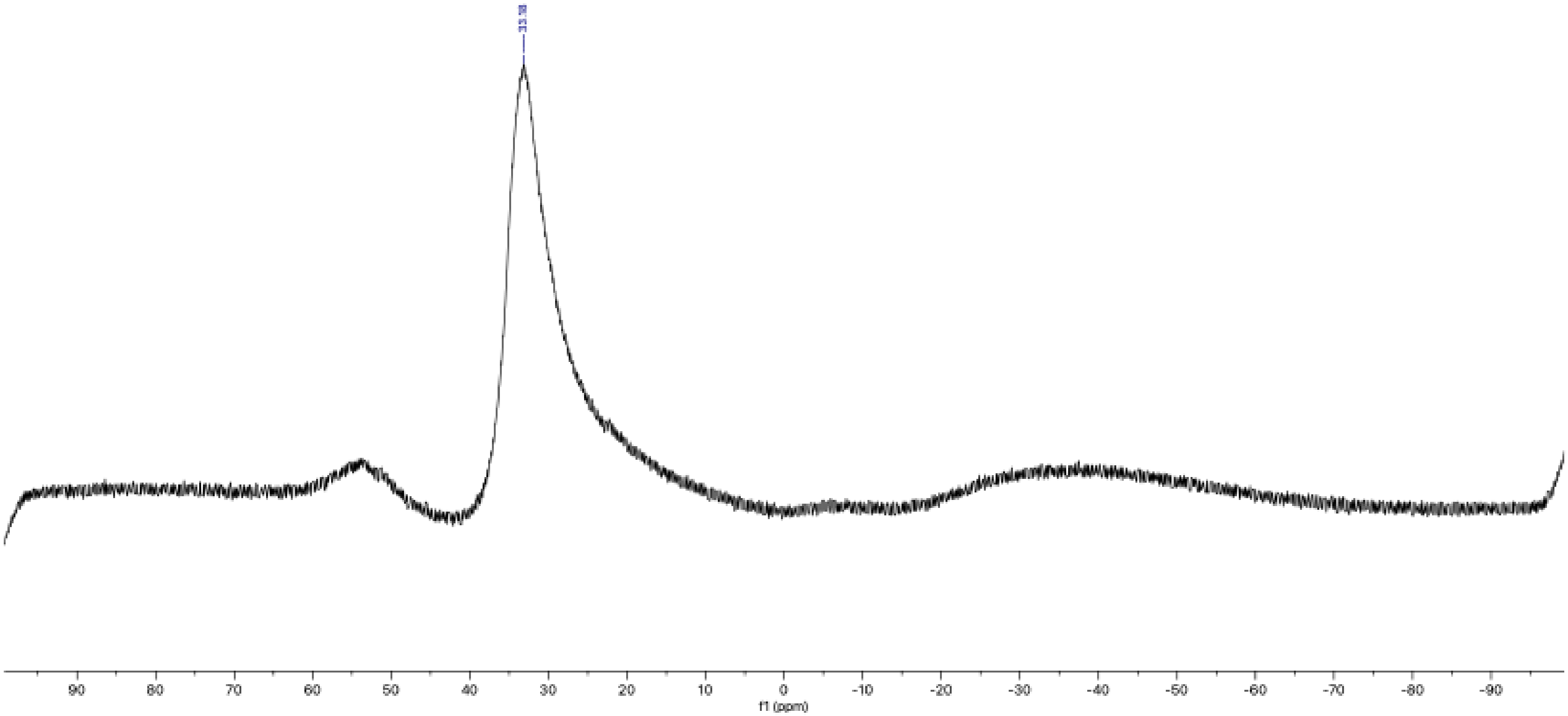

^{11}B NMR spectrum (128 MHz, $CDCl_3$)

2-((3*R,4*R**)-4-Ethyl-3-phenylhepta-1,6-dien-4-yl)-4,4,5,5-tetramethyl-1,3,2-dioxaborolane 7k**

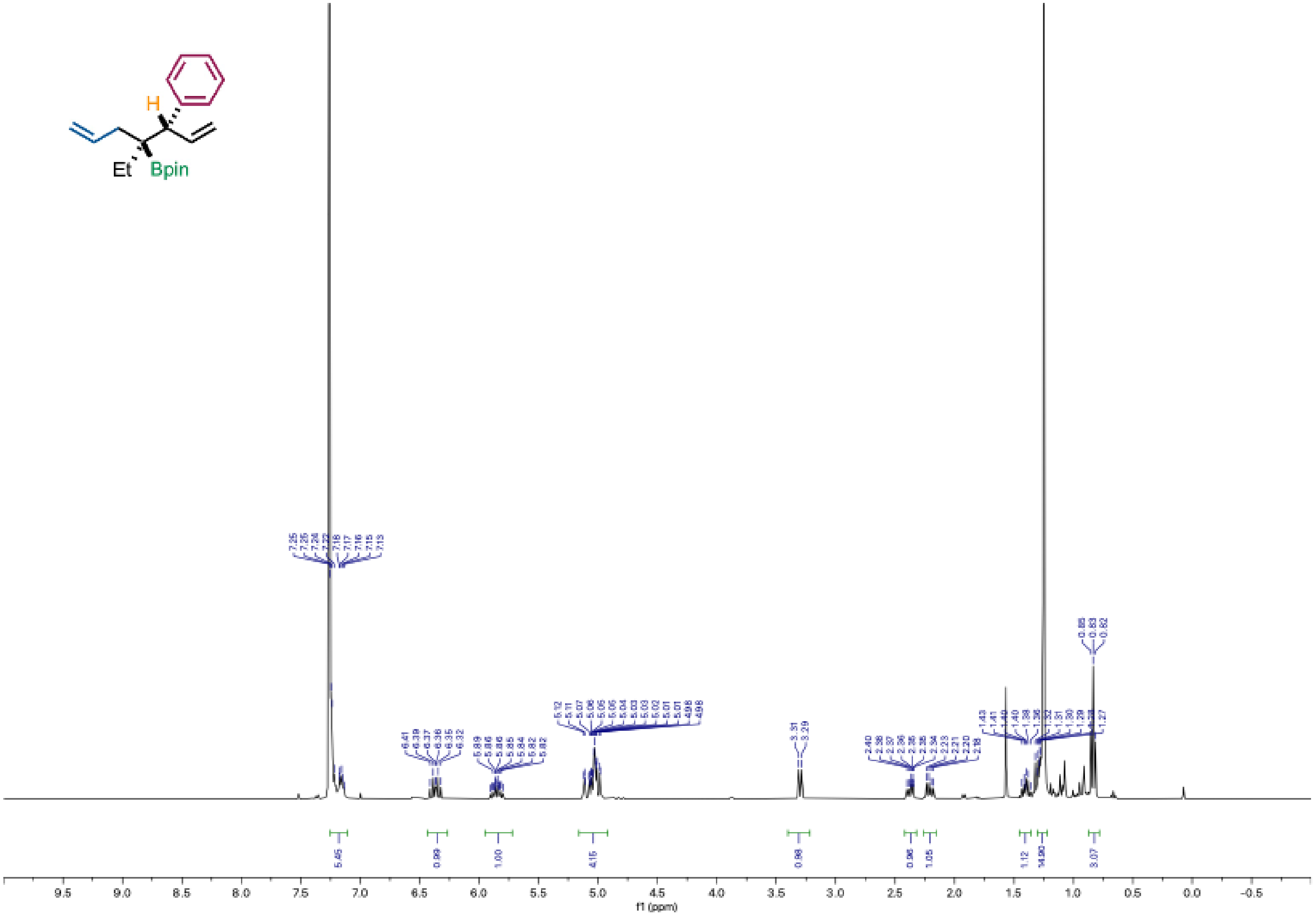

^{1}H NMR spectrum (400 MHz, $CDCl_3$)

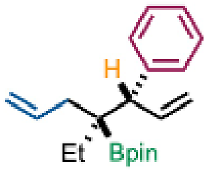

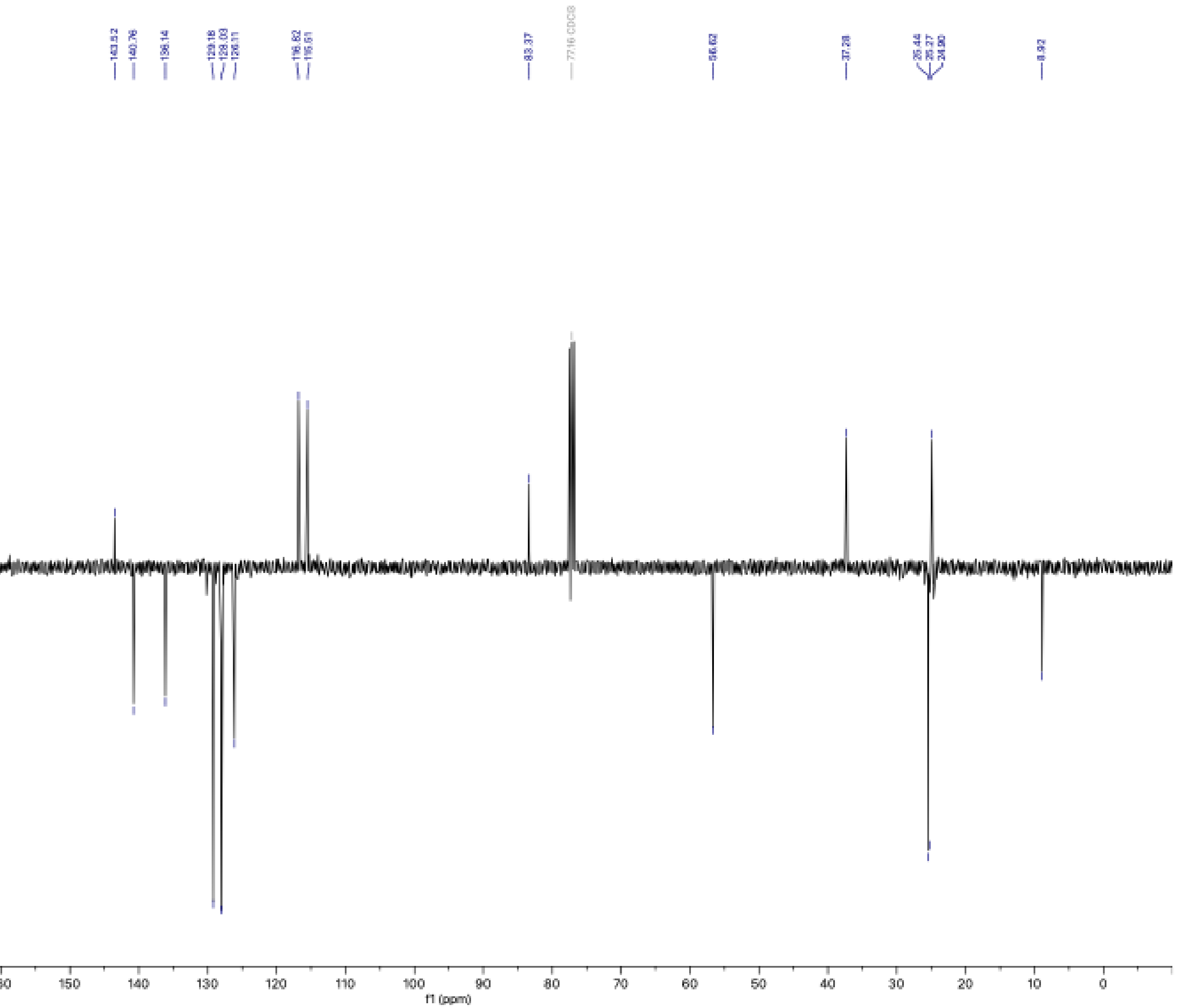

^{13}**C NMR** spectrum (101 MHz, $CDCl_3$)

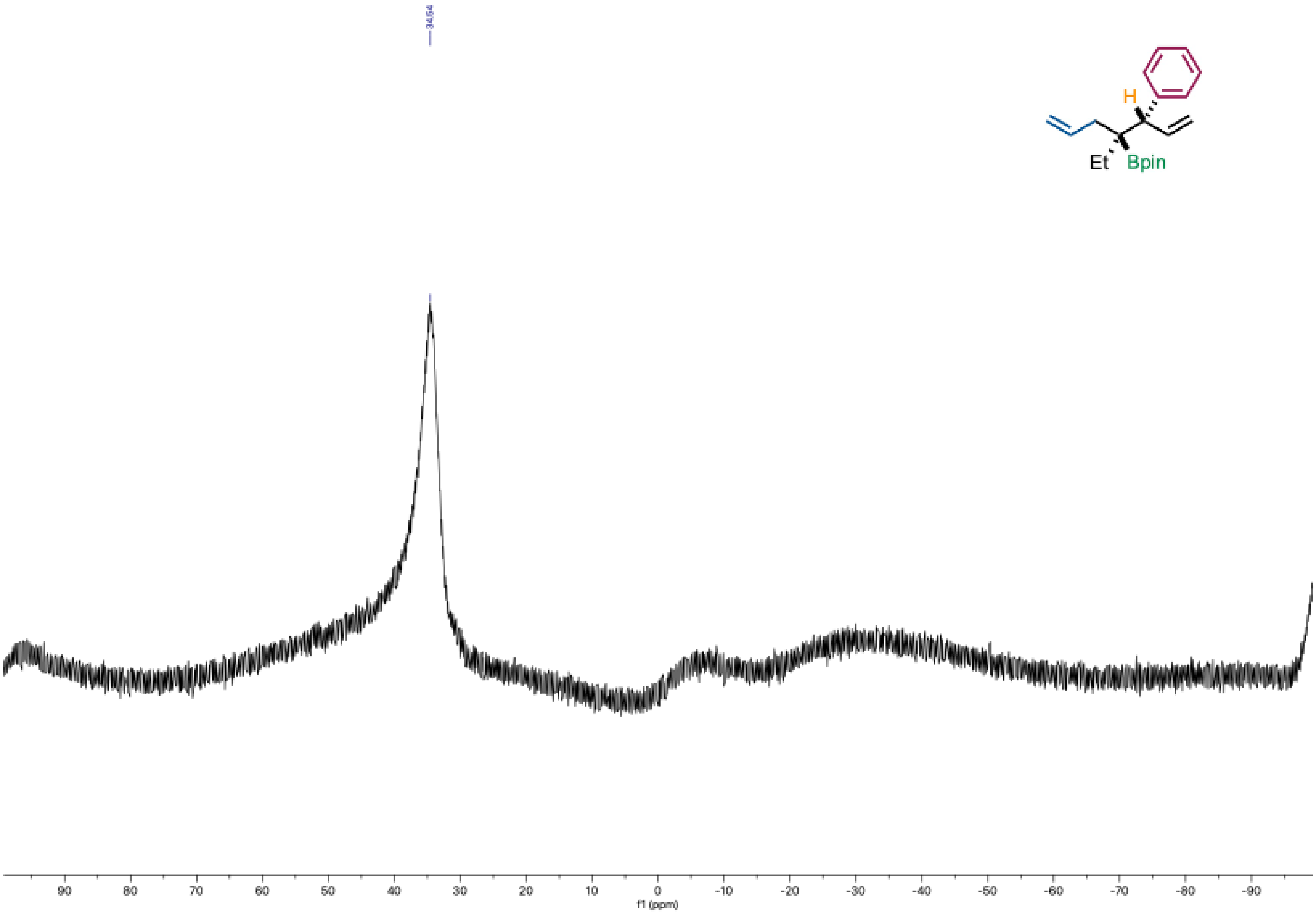

^{11}B NMR spectrum (128 MHz, $CDCl_3$)

4,4,5,5-tetramethyl-2-((3*R,4*R**)-4-methyl-3-(*p*-tolyl)hepta-1,6-dien-4-yl)-1,3,2-dioxaborolane 7l**

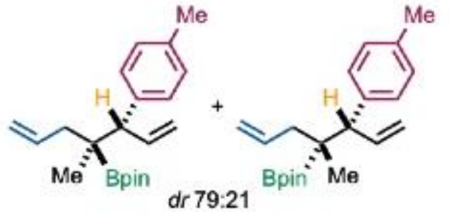

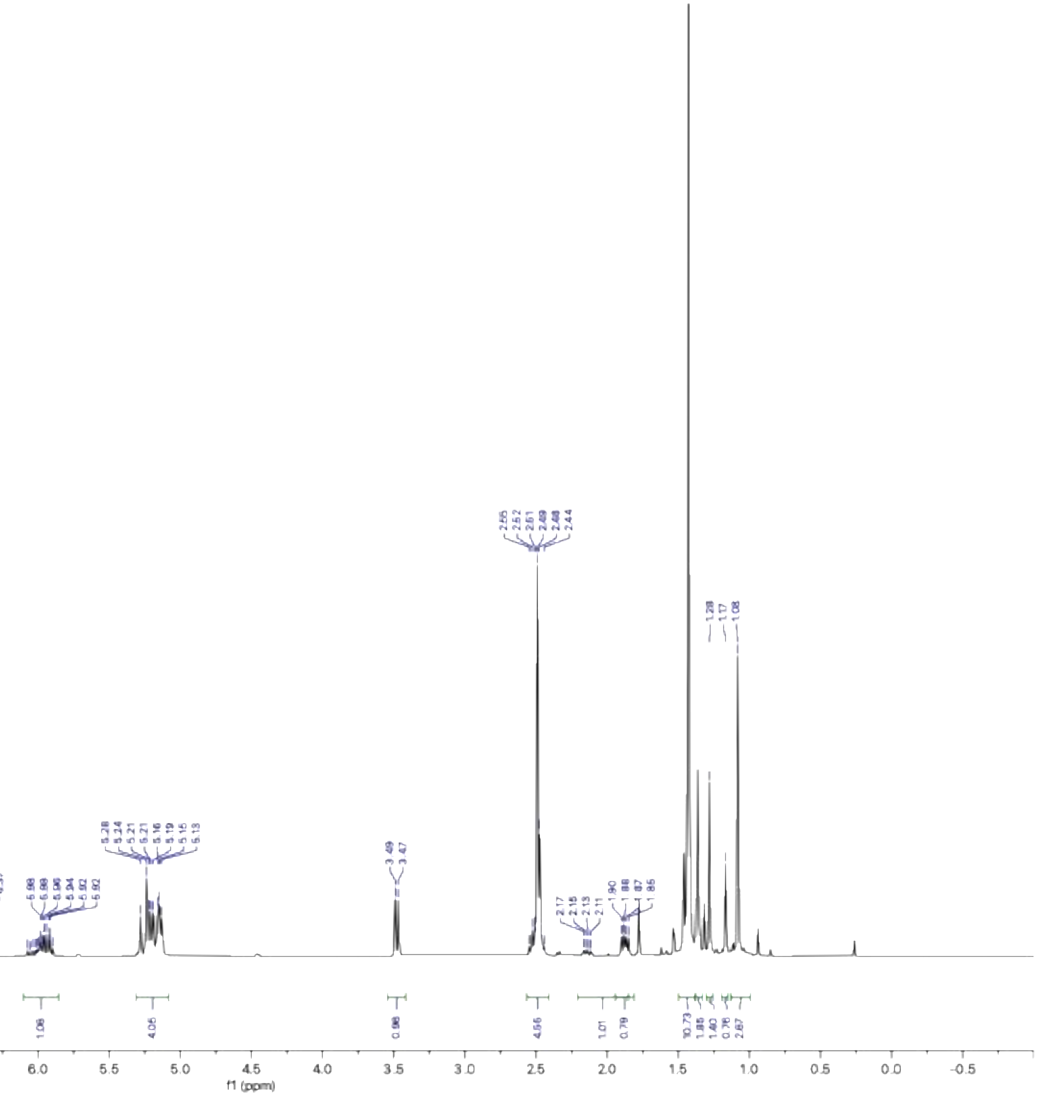

^{1}H NMR spectrum (400 MHz, $CDCl_3$)

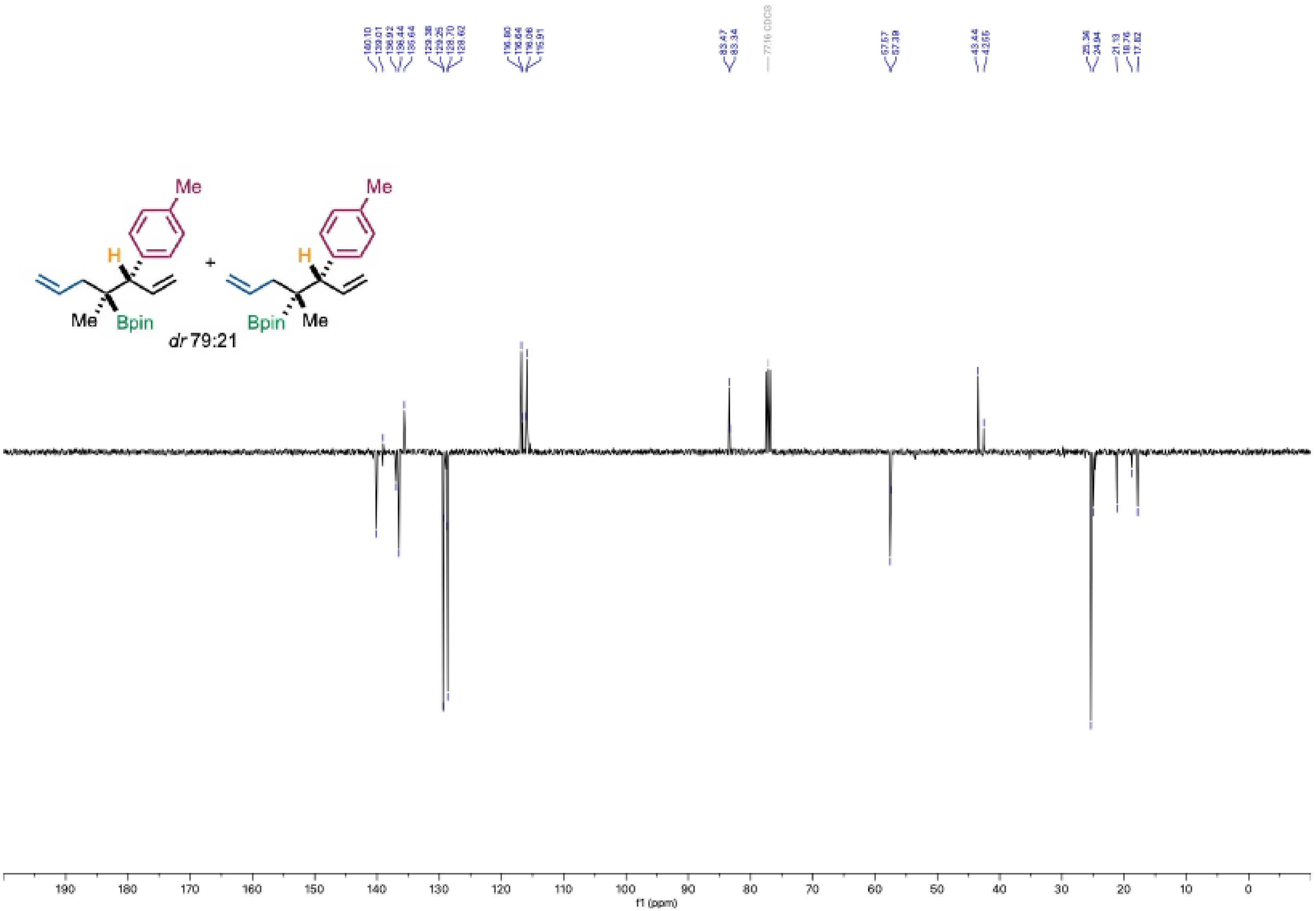

^{13}**C NMR** spectrum (101 MHz, $CDCl_3$)

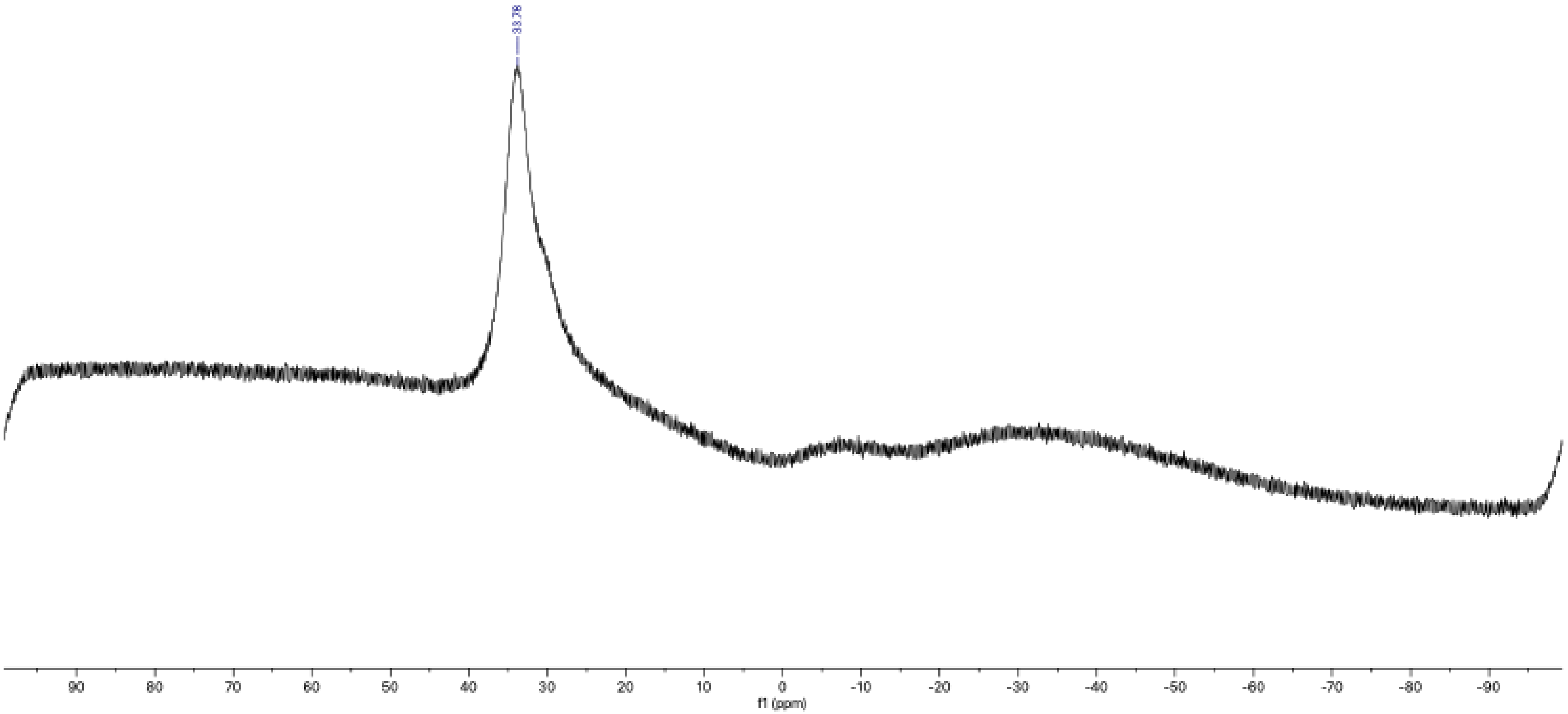

^{11}**B NMR** spectrum (128 MHz, $CDCl_3$)

2-((4*R,5*R**)-4-Ethyl-2-methyl-5-phenylhepta-1,6-dien-4-yl)-4,4,5,5-tetramethyl-1,3,2-dioxaborolane 7m**

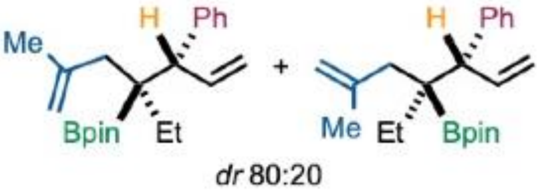

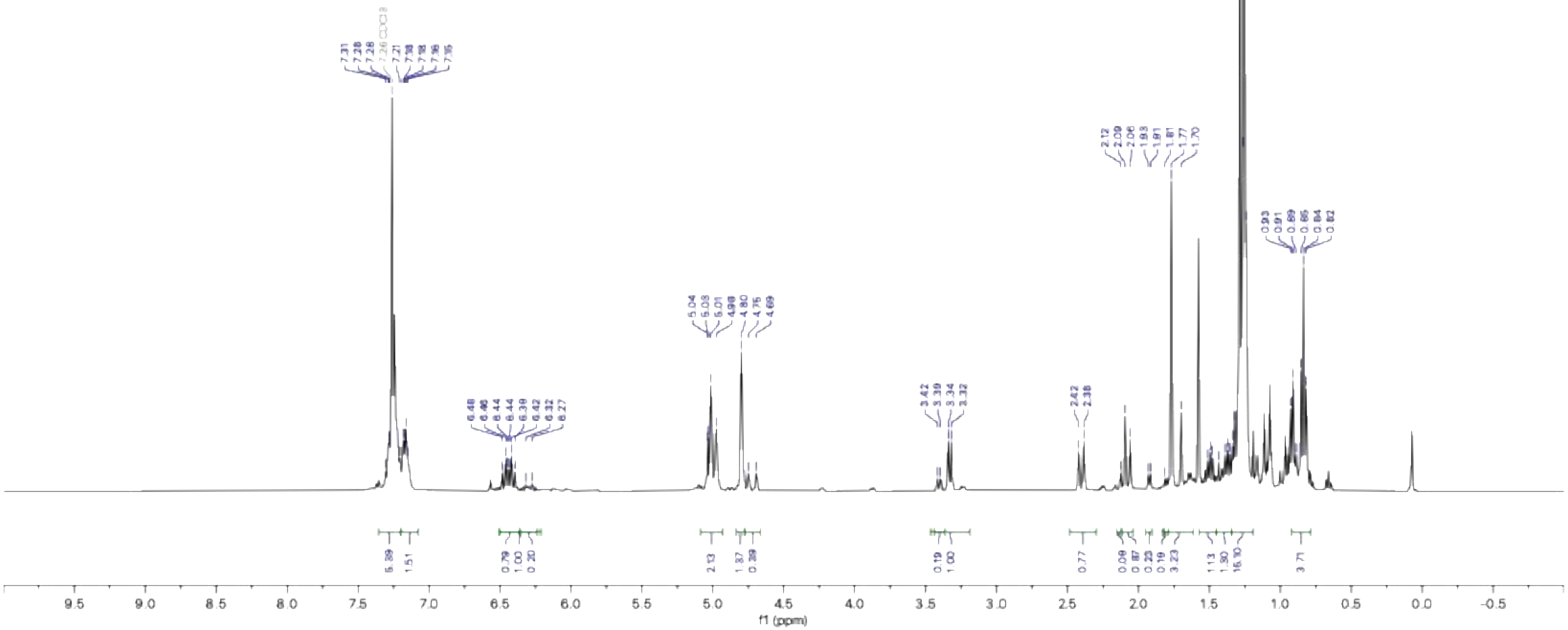

^{1}H NMR spectrum (400 MHz, $CDCl_3$)

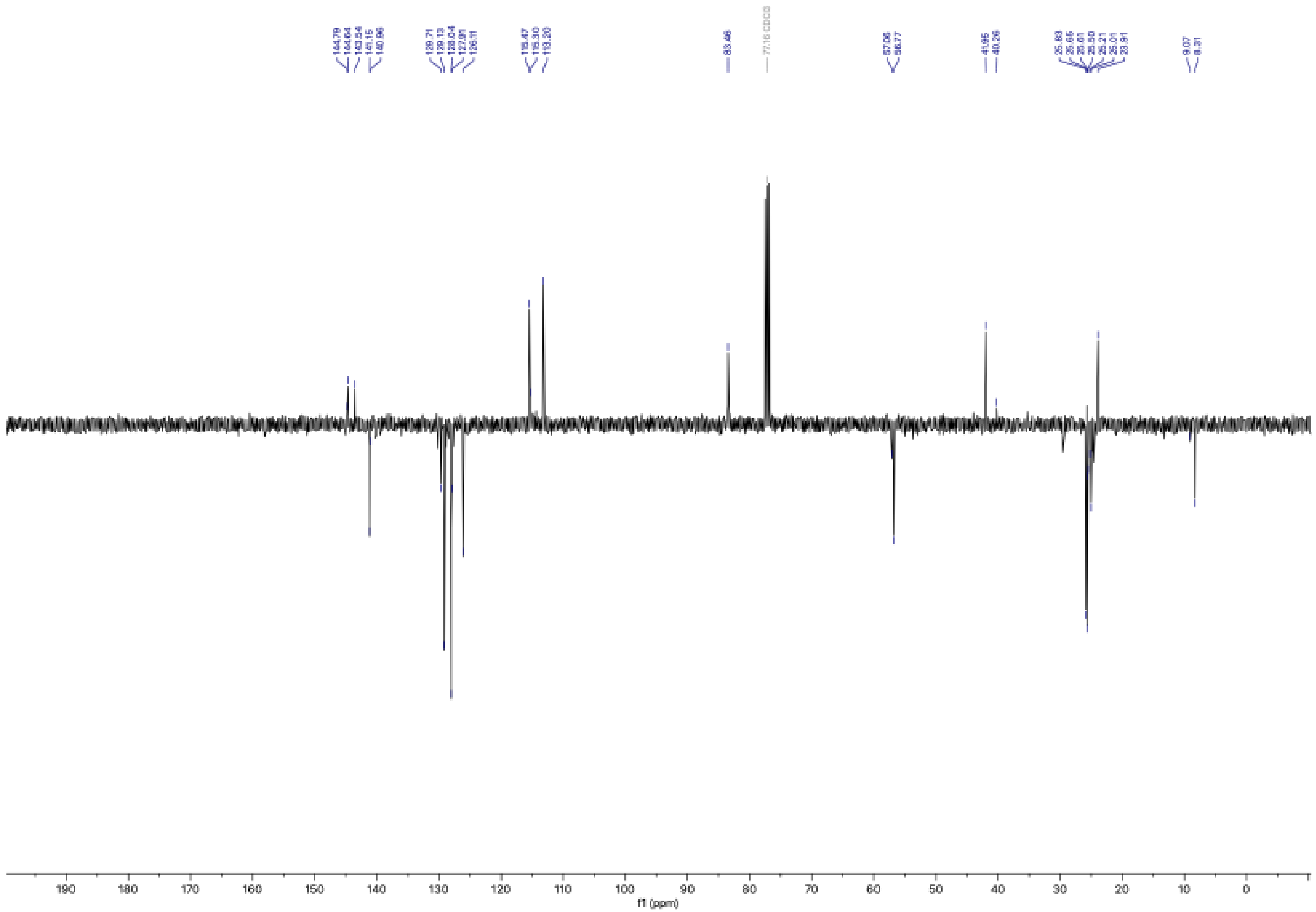

^{13}C NMR spectrum (101 MHz, $CDCl_3$)

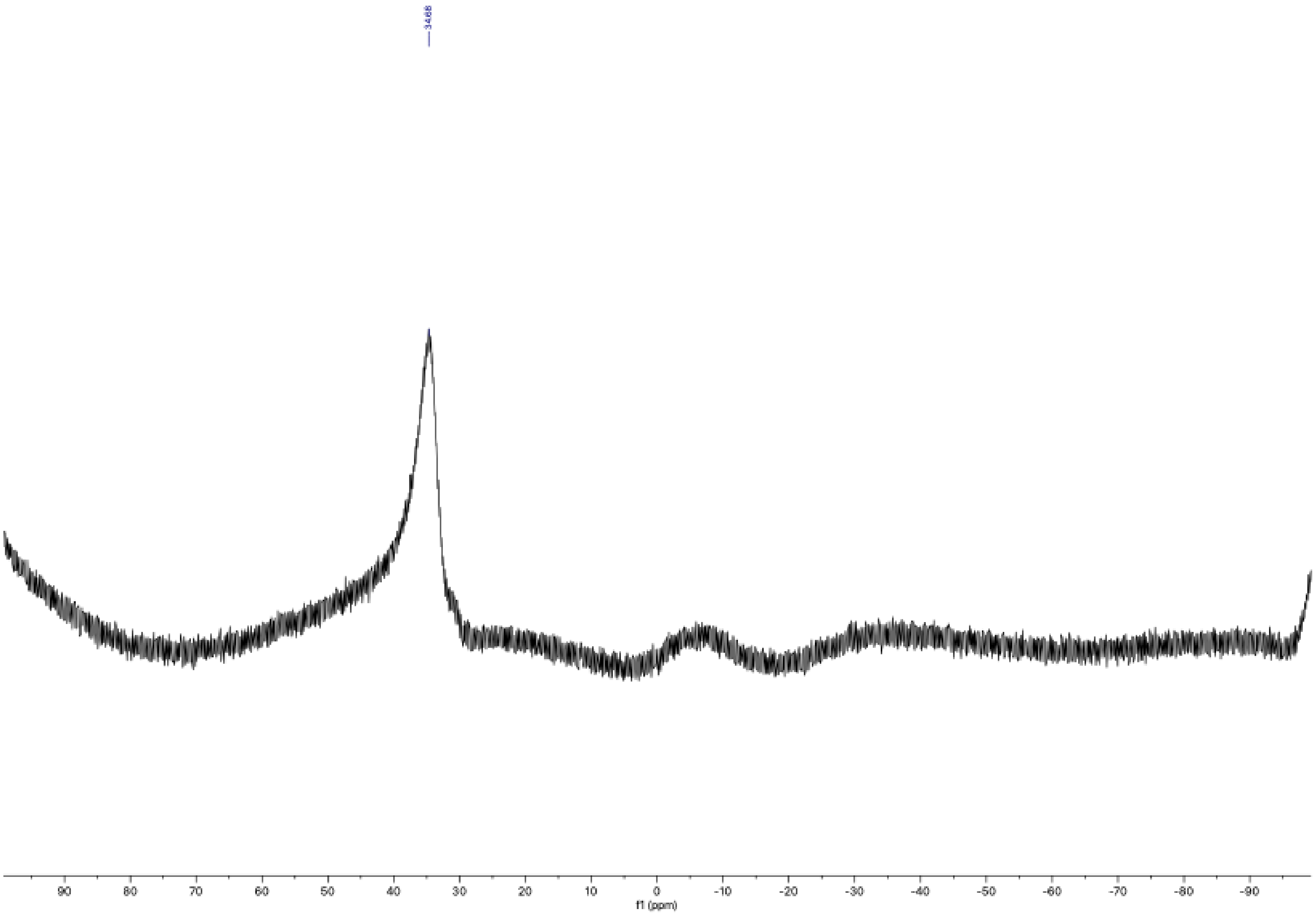

^{11}**B NMR** spectrum (128 MHz, $CDCl_3$)

(3*S**,4*R**)-3-Ethyl-2-methyl-4-phenyl-3-(4,4,5,5-tetramethyl-1,3,2-dioxaborolan-2-yl)hex-5-en-2-ol 7n

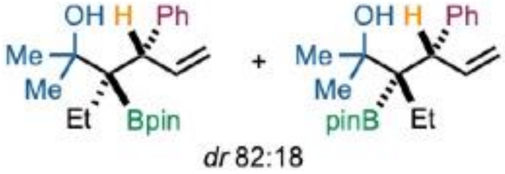

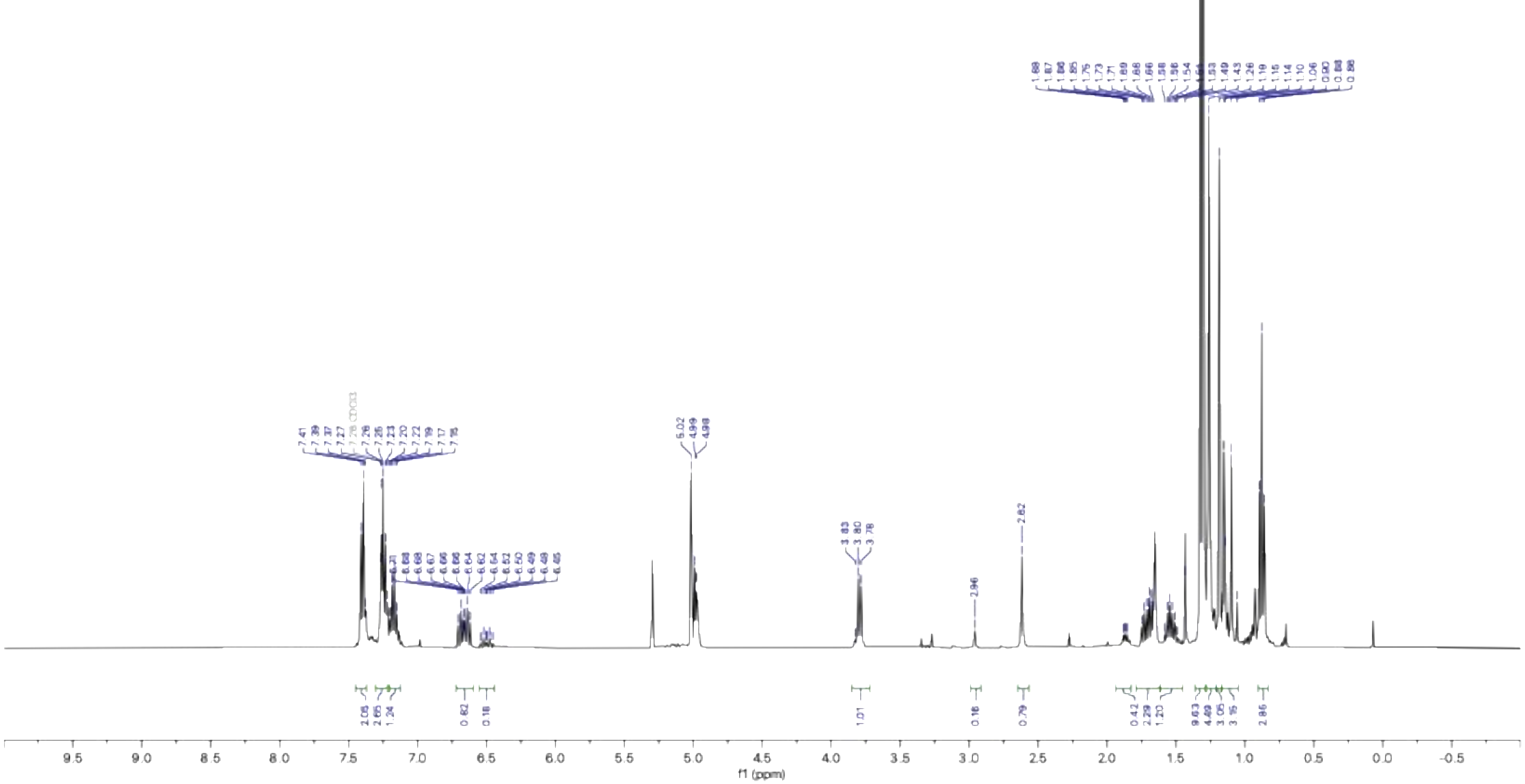

[1]**H NMR** spectrum (400 MHz, $CDCl_3$)

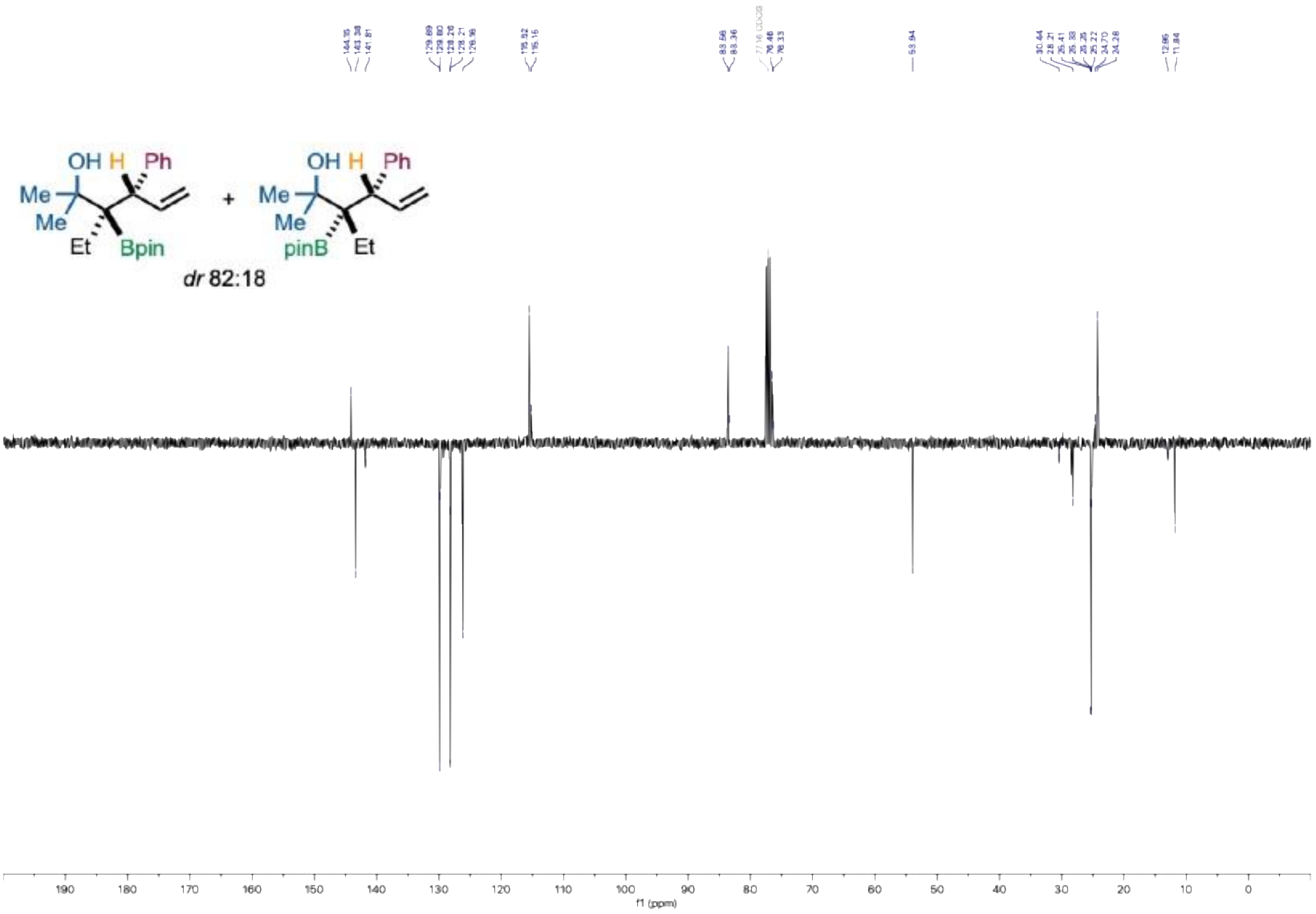

^{13}C NMR spectrum (101 MHz, $CDCl_3$)

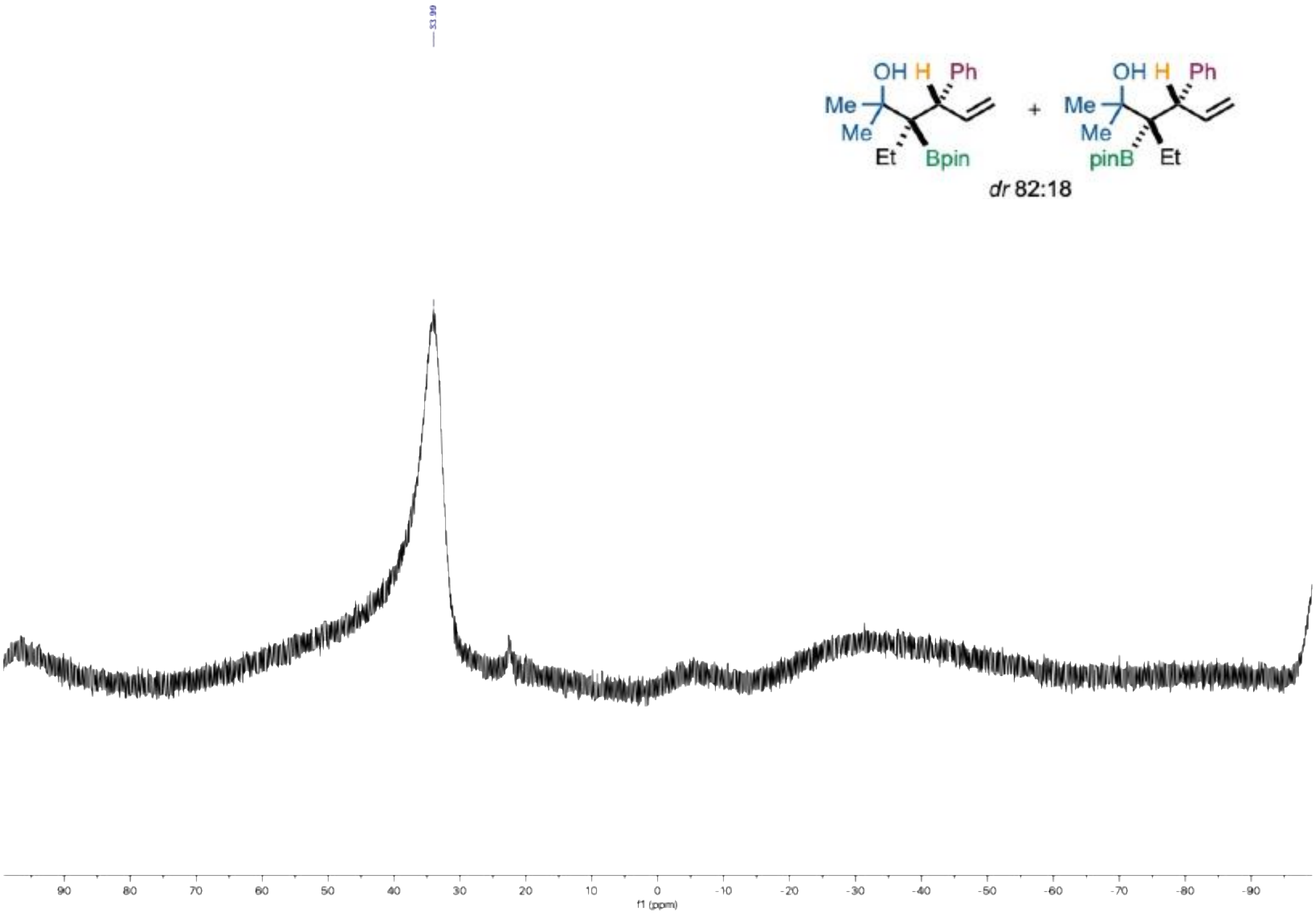

^{11}B NMR spectrum (128 MHz, $CDCl_3$)

4,4,5,5-tetramethyl-2-((3*S,4*S**)-3-(Methylthio)-4-phenylhex-5-en-3-yl)-1,3,2-dioxaborolane 7o**

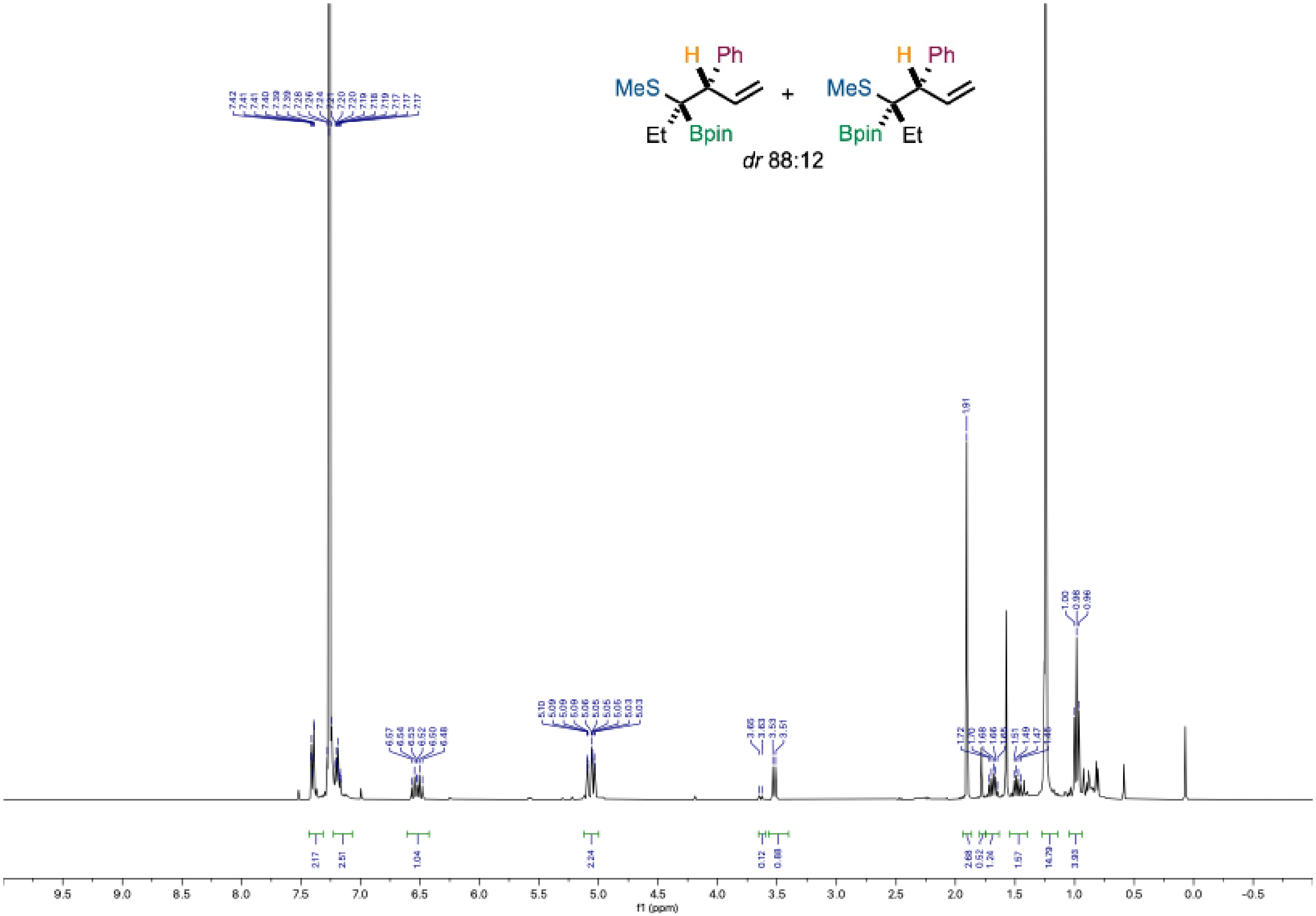

[1]**H NMR** spectrum (400 MHz, $CDCl_3$)

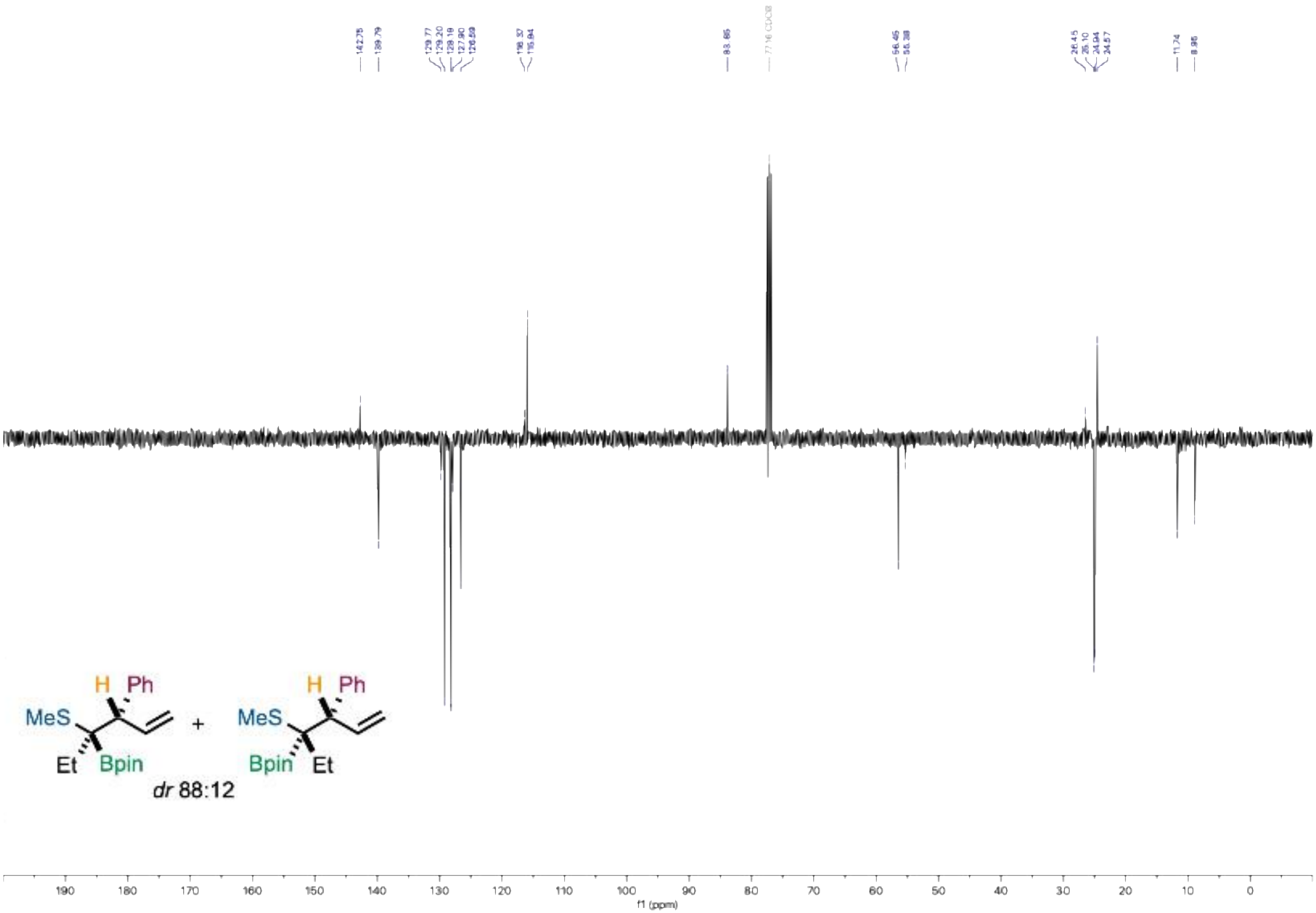

^{13}C NMR spectrum (101 MHz, $CDCl_3$)

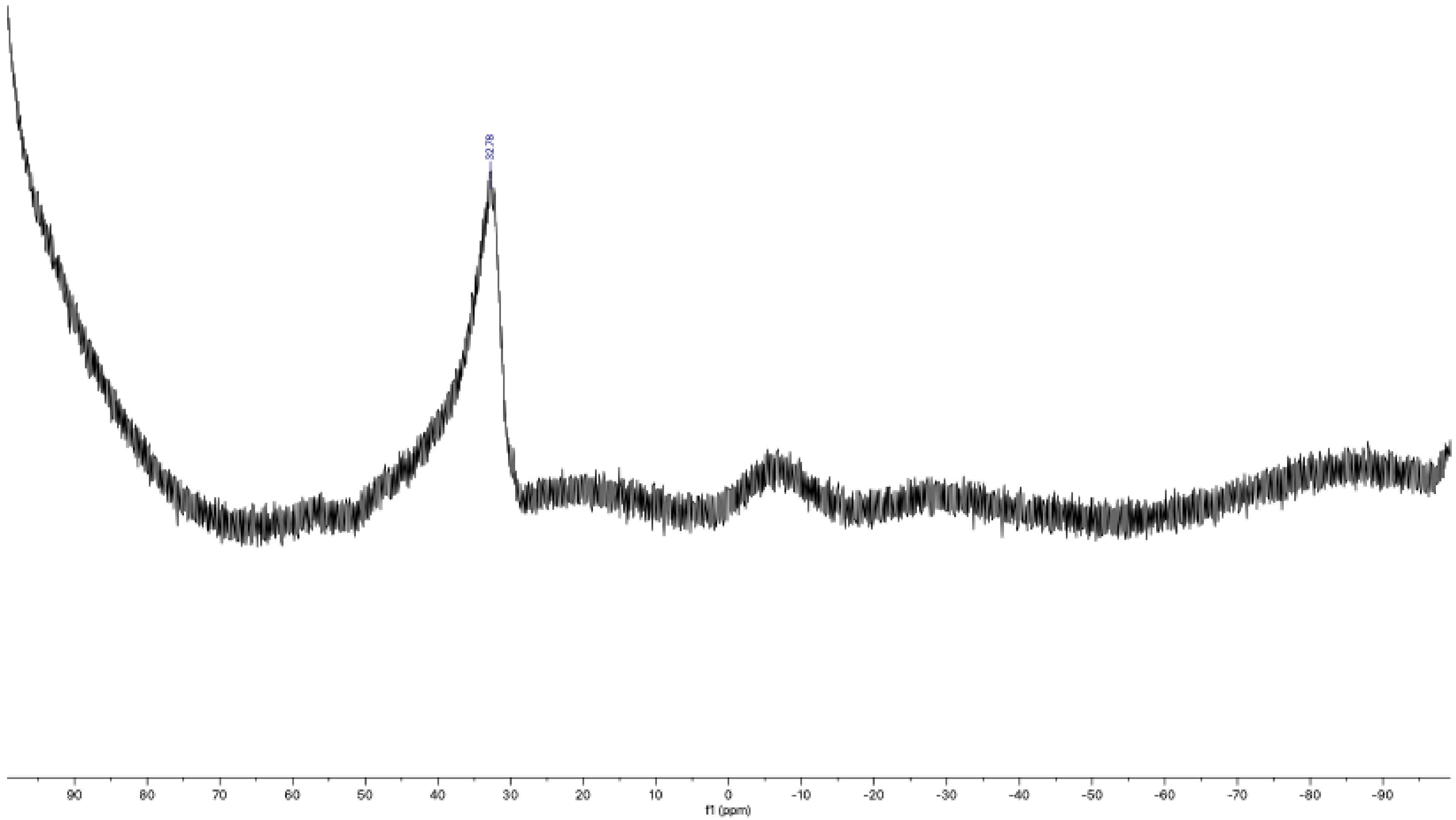

^{11}B NMR spectrum (128 MHz, $CDCl_3$)

2-((3*R,4*R**)-3-Benzyl-4-phenylhex-5-en-3-yl)-4,4,5,5-tetramethyl-1,3,2-dioxaborolane 7p**

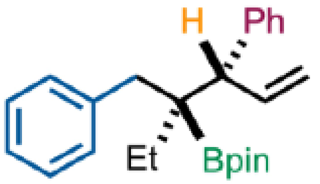

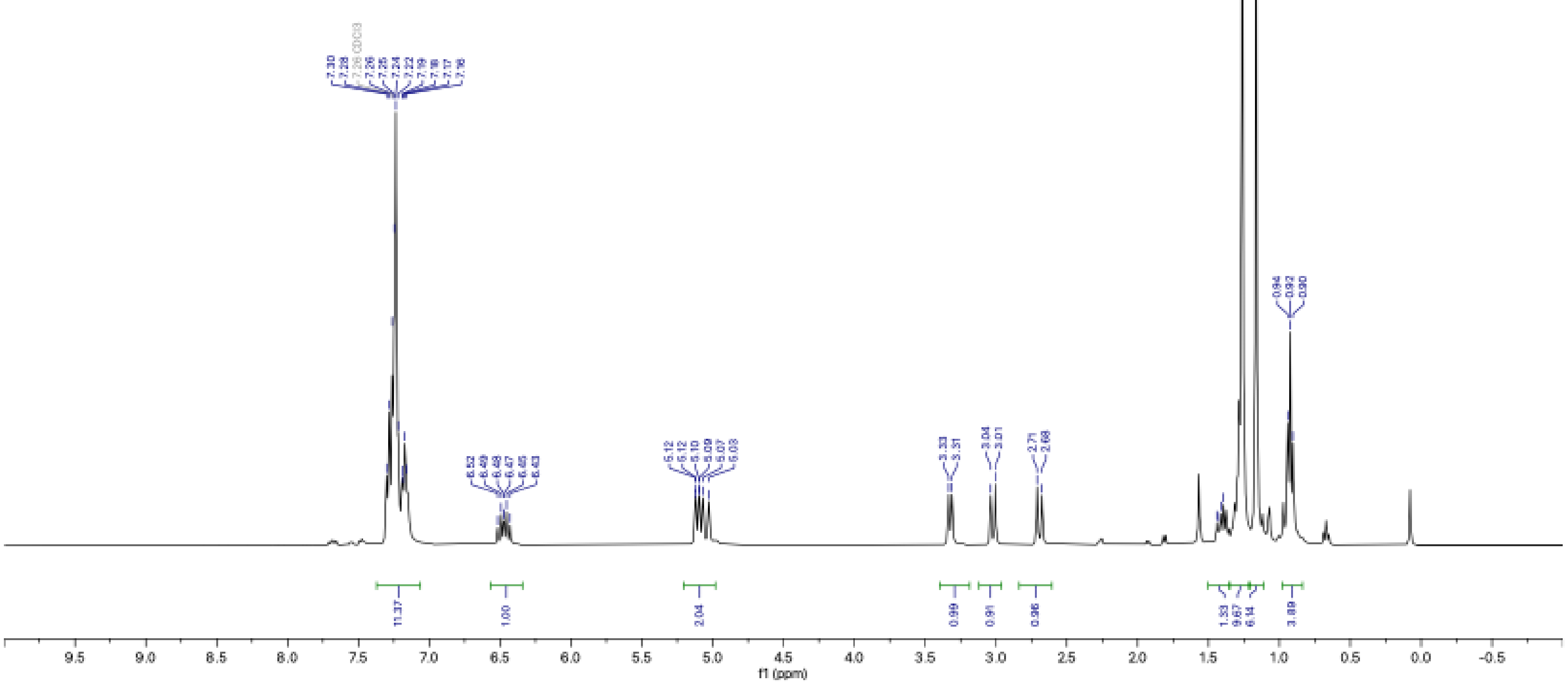

^{1}H NMR spectrum (400 MHz, $CDCl_3$)

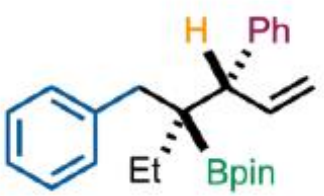

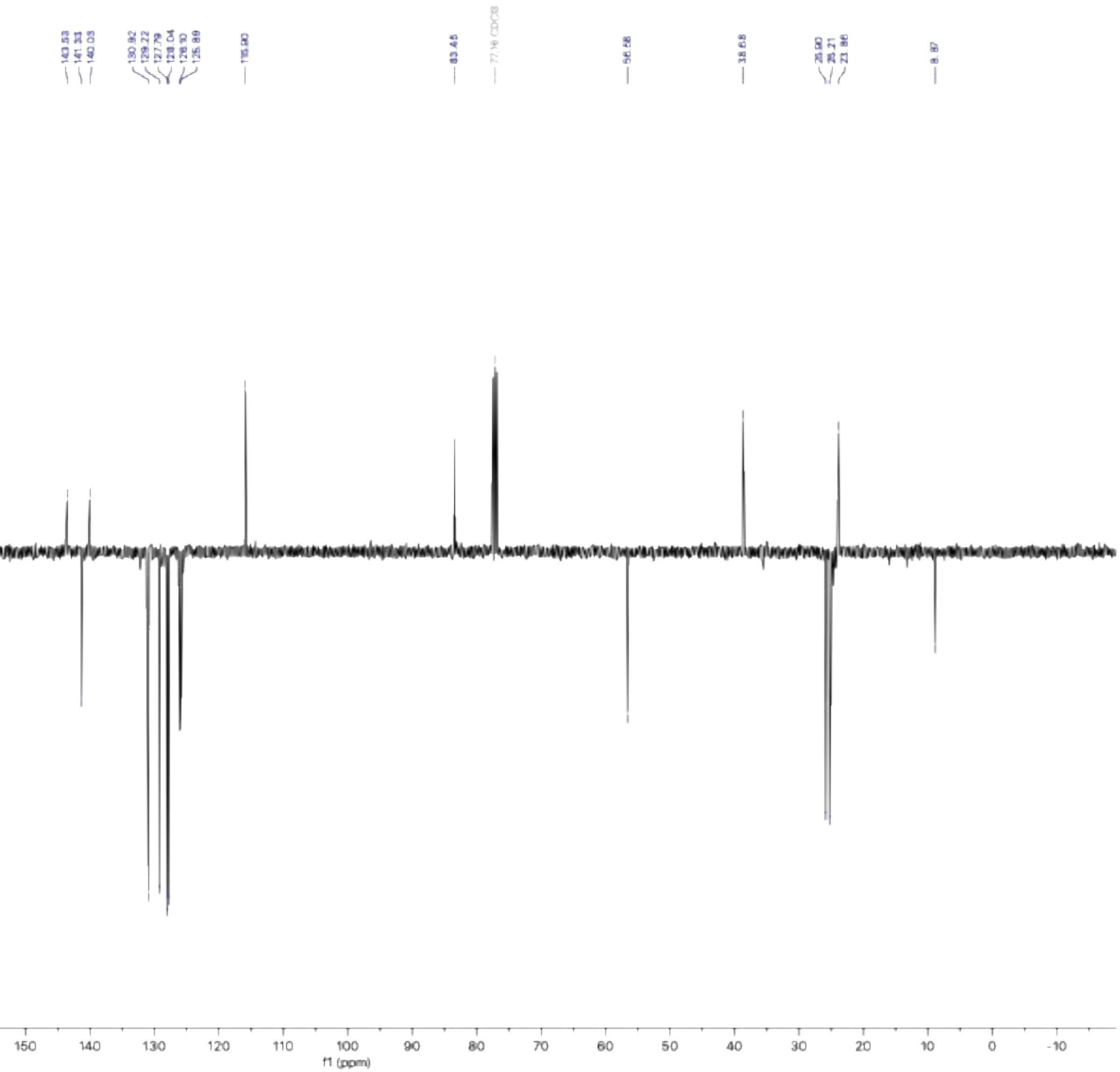

^{13}**C NMR** spectrum (101 MHz, $CDCl_3$)

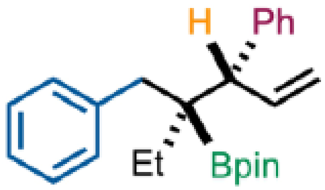

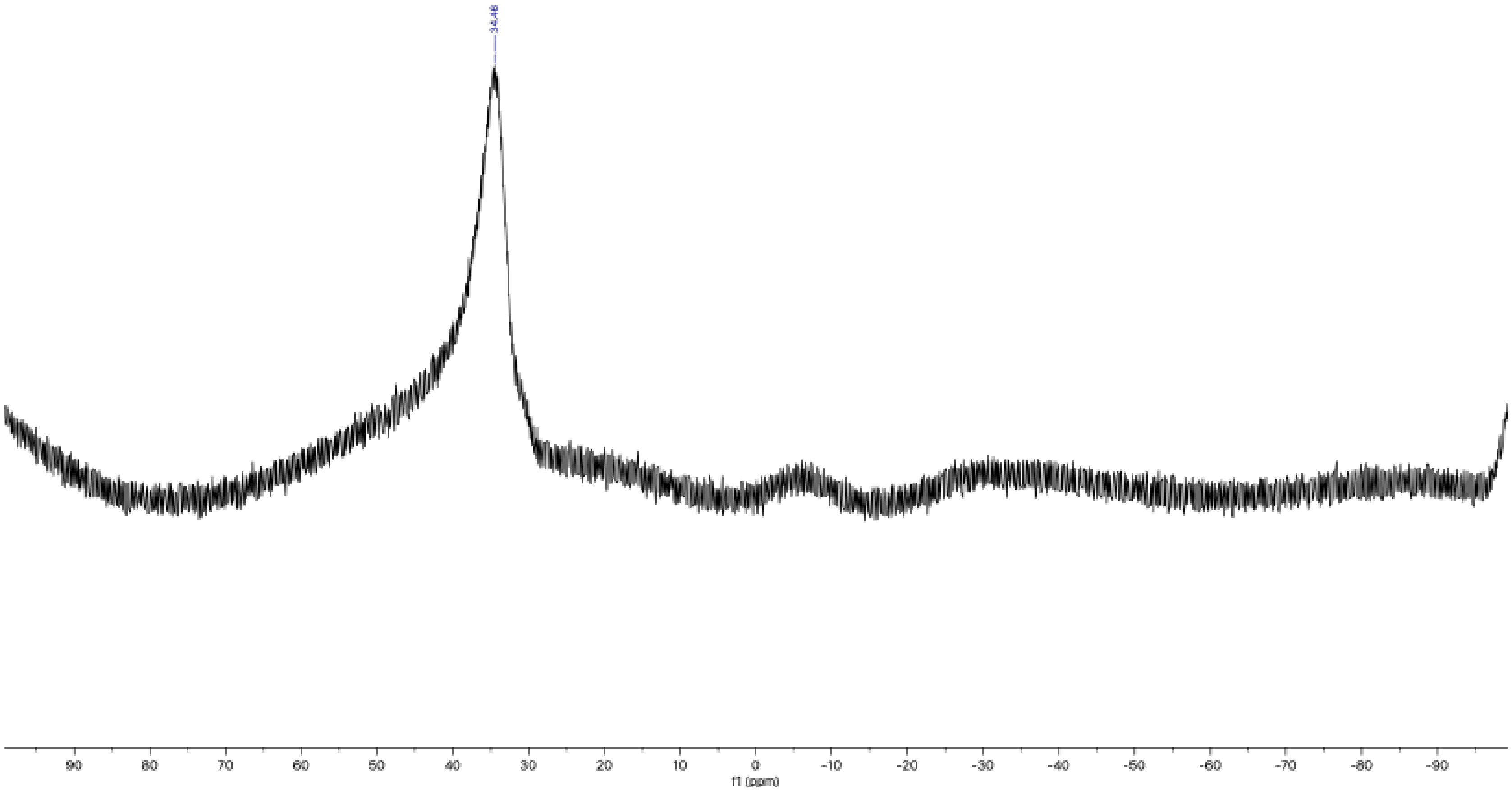

^{11}B NMR spectrum (128 MHz, $CDCl_3$)

2,2'-((2*R,3*R**)-2-Methyl-3-(*p*-tolyl)pent-4-ene-1,2-diyl)bis(4,4,5,5-tetramethyl-1,3,2-dioxaborolane) 7q**

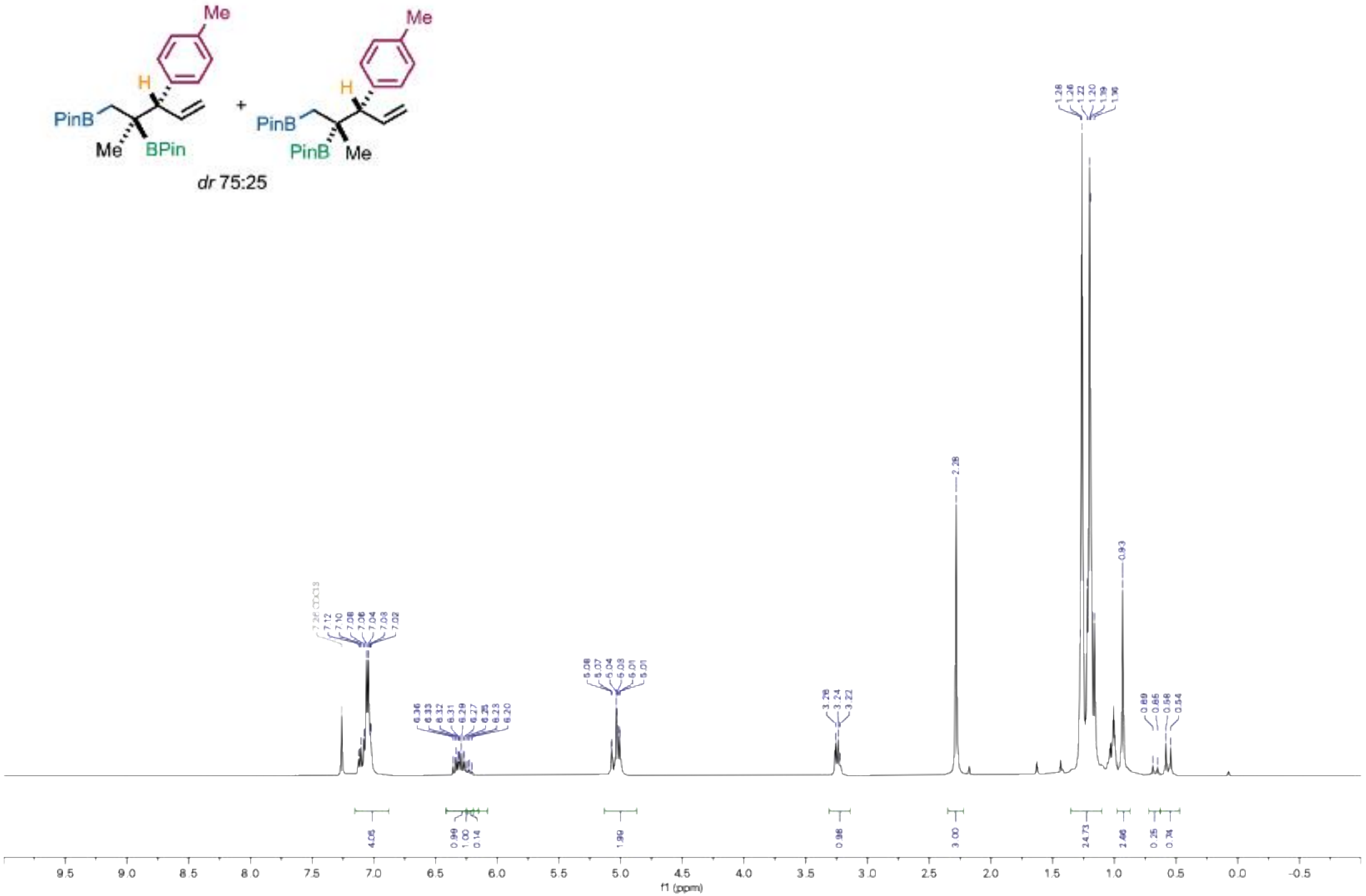

^{1}H NMR spectrum (400 MHz, $CDCl_3$)

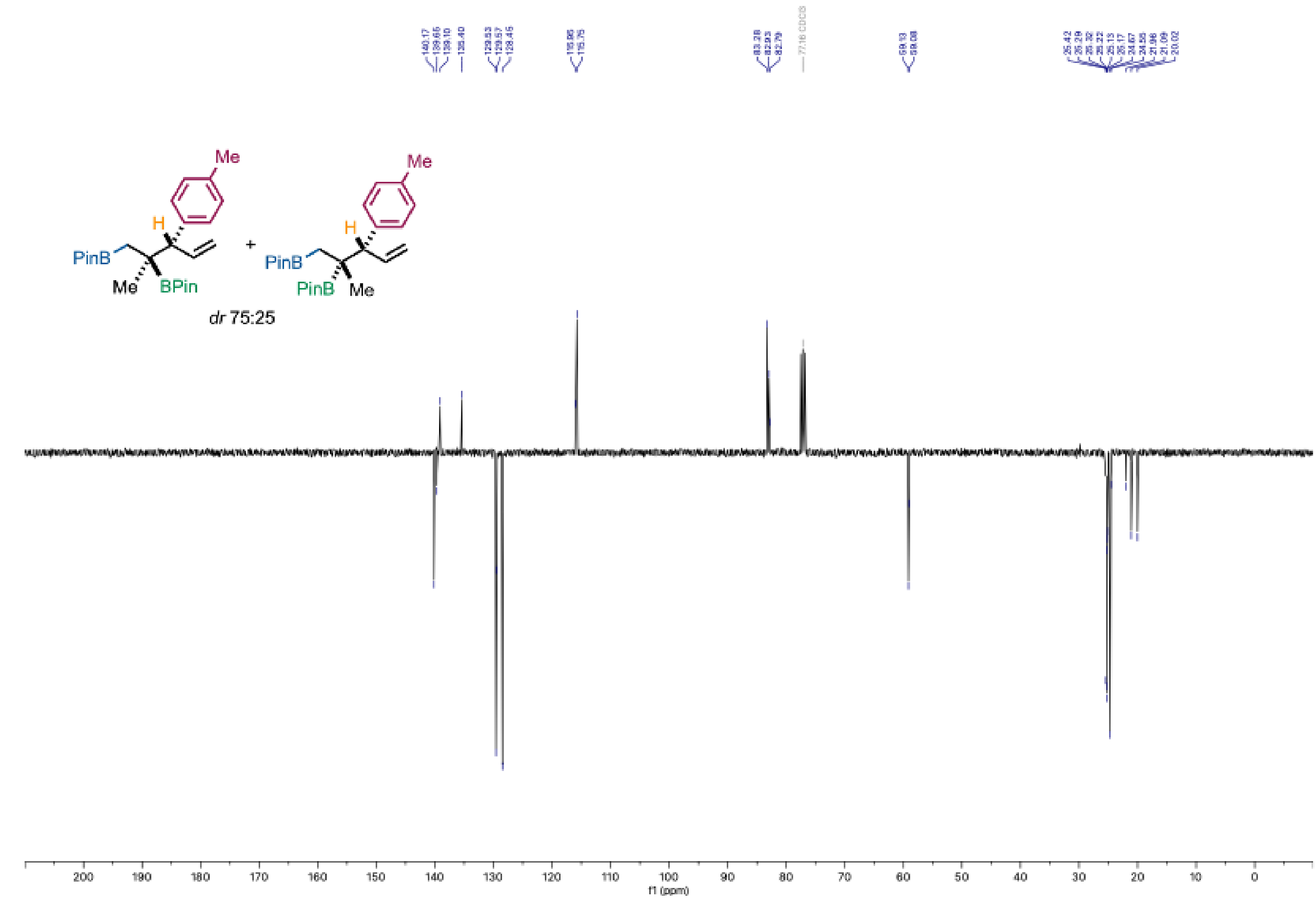

^{13}C NMR spectrum (101 MHz, $CDCl_3$)

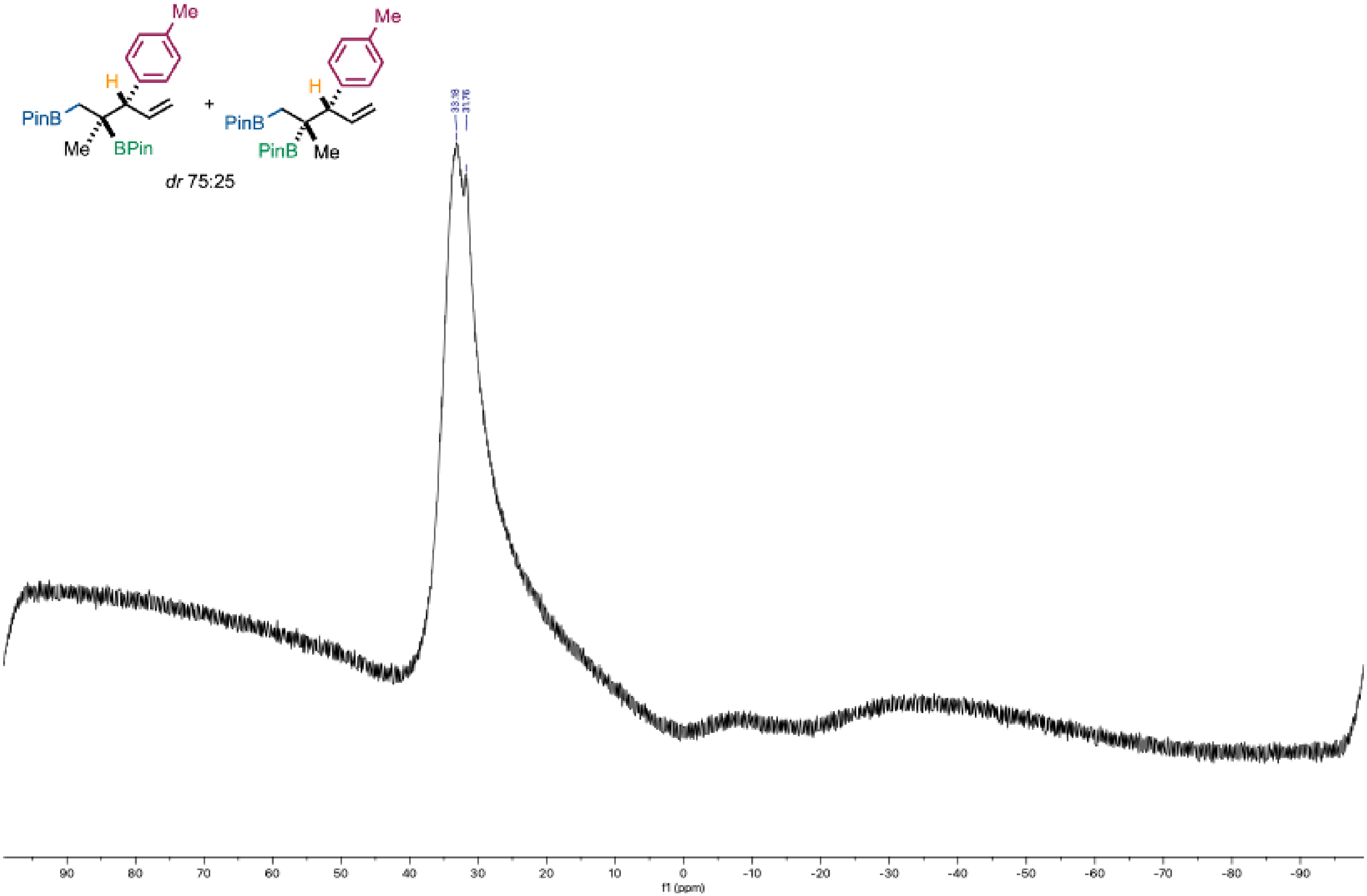

^{11}B NMR spectrum (128 MHz, $CDCl_3$)

4,4,5,5-Tetramethyl-2-(1-(1-phenylallyl)cyclobutyl)-1,3,2-dioxaborolane 7r

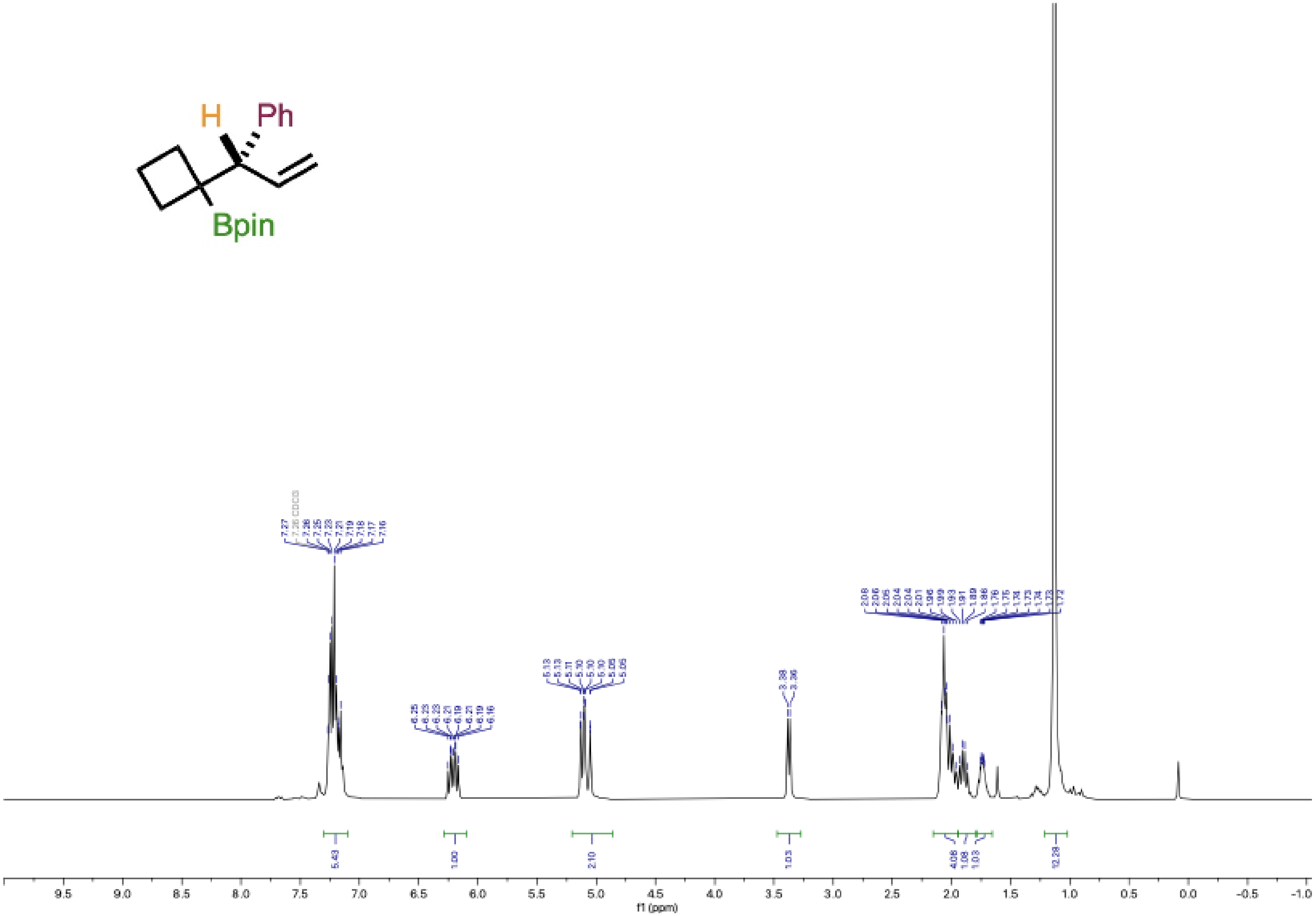

^{1}H NMR spectrum (400 MHz, $CDCl_3$)

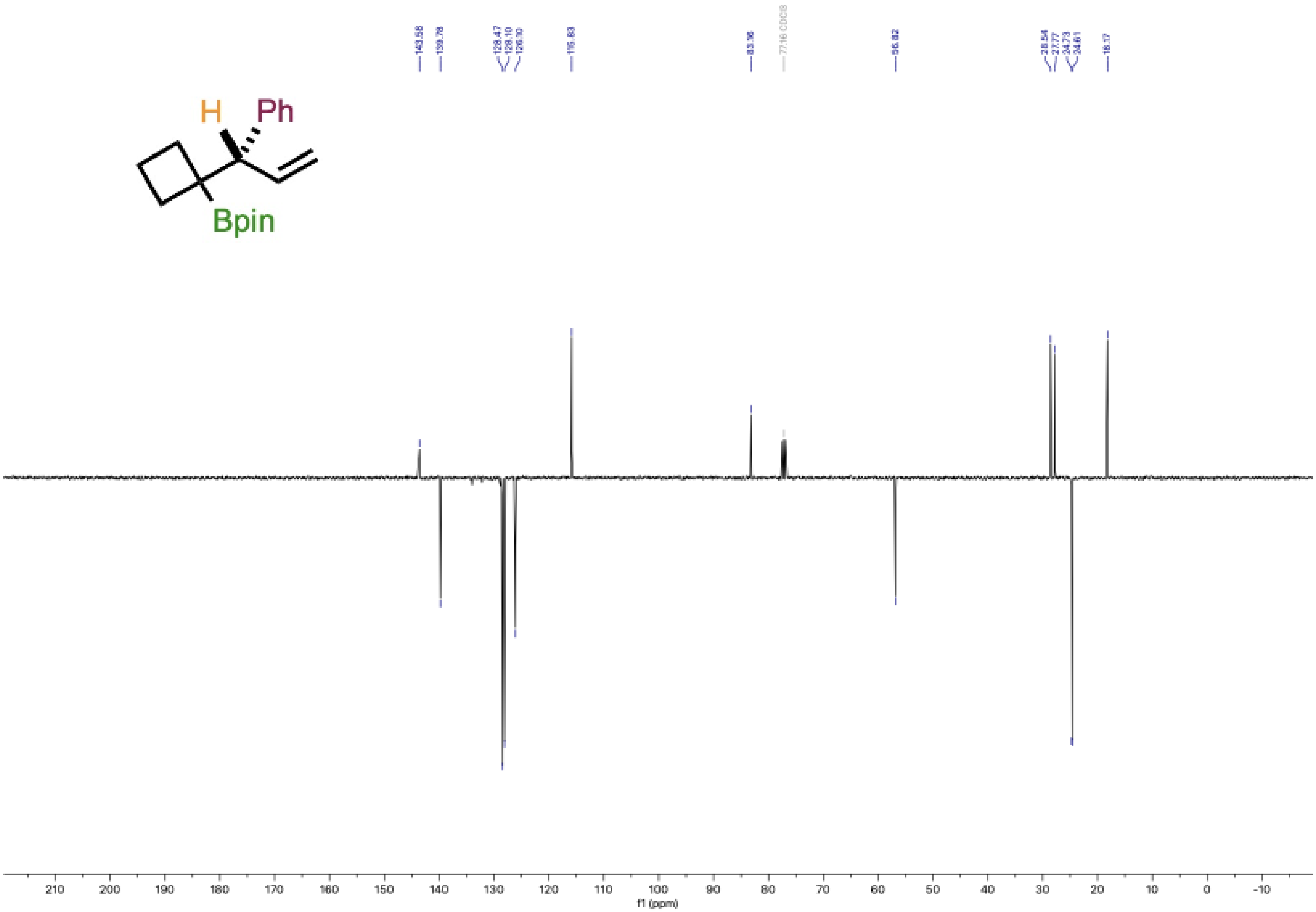

^{13}C NMR spectrum (101 MHz, $CDCl_3$)

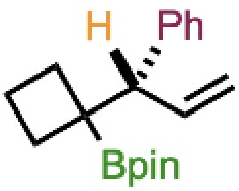

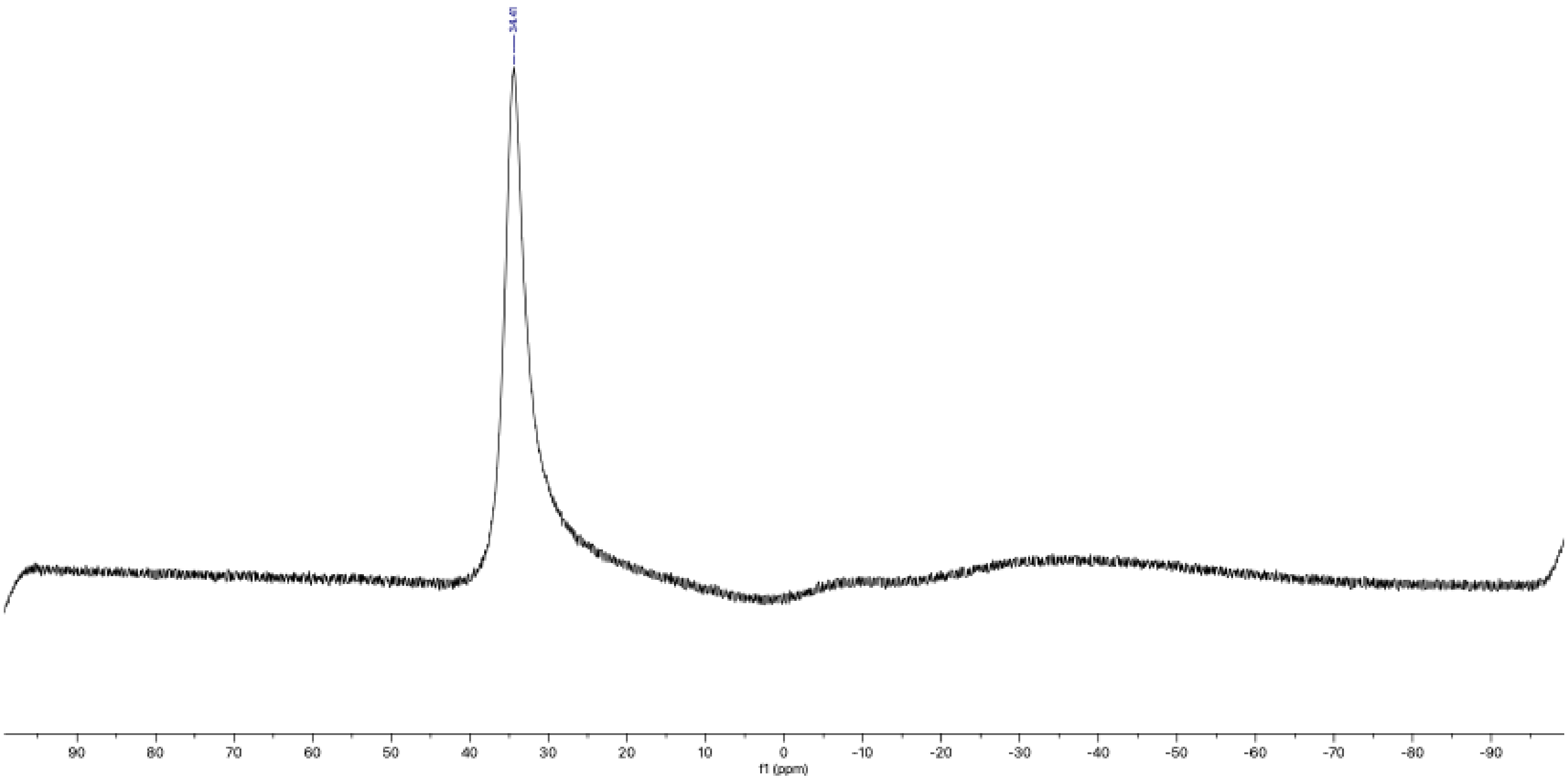

¹¹B NMR spectrum (128 MHz, $CDCl_3$)

(1*R,2*S**,3*S**)-1,2-dimethyl-2-(4,4,5,5-tetramethyl-1,3,2-dioxaborolan-2-yl)-3-(4-(trifluoromethyl)phenyl)-3-vinylcyclobutan-1-ol 7s**

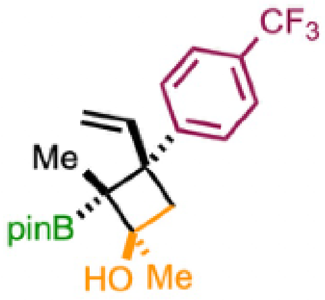

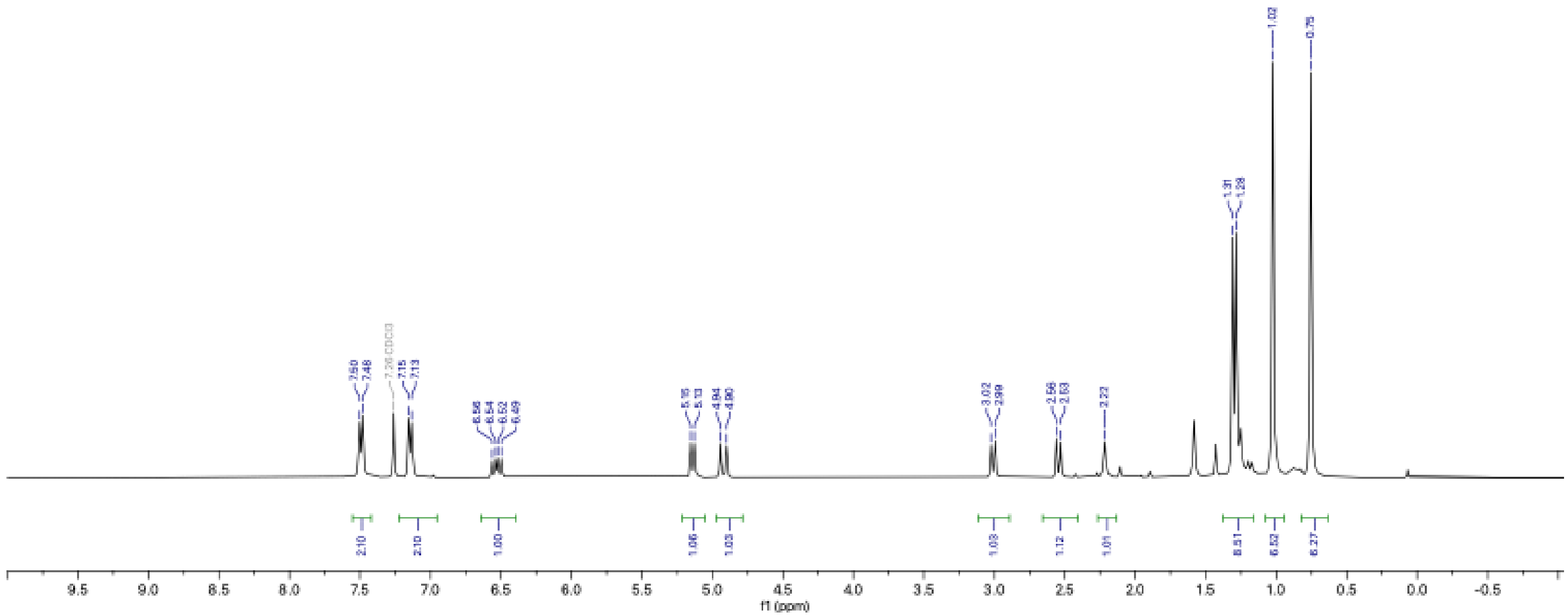

^{1}H NMR spectrum (400 MHz, $CDCl_3$)

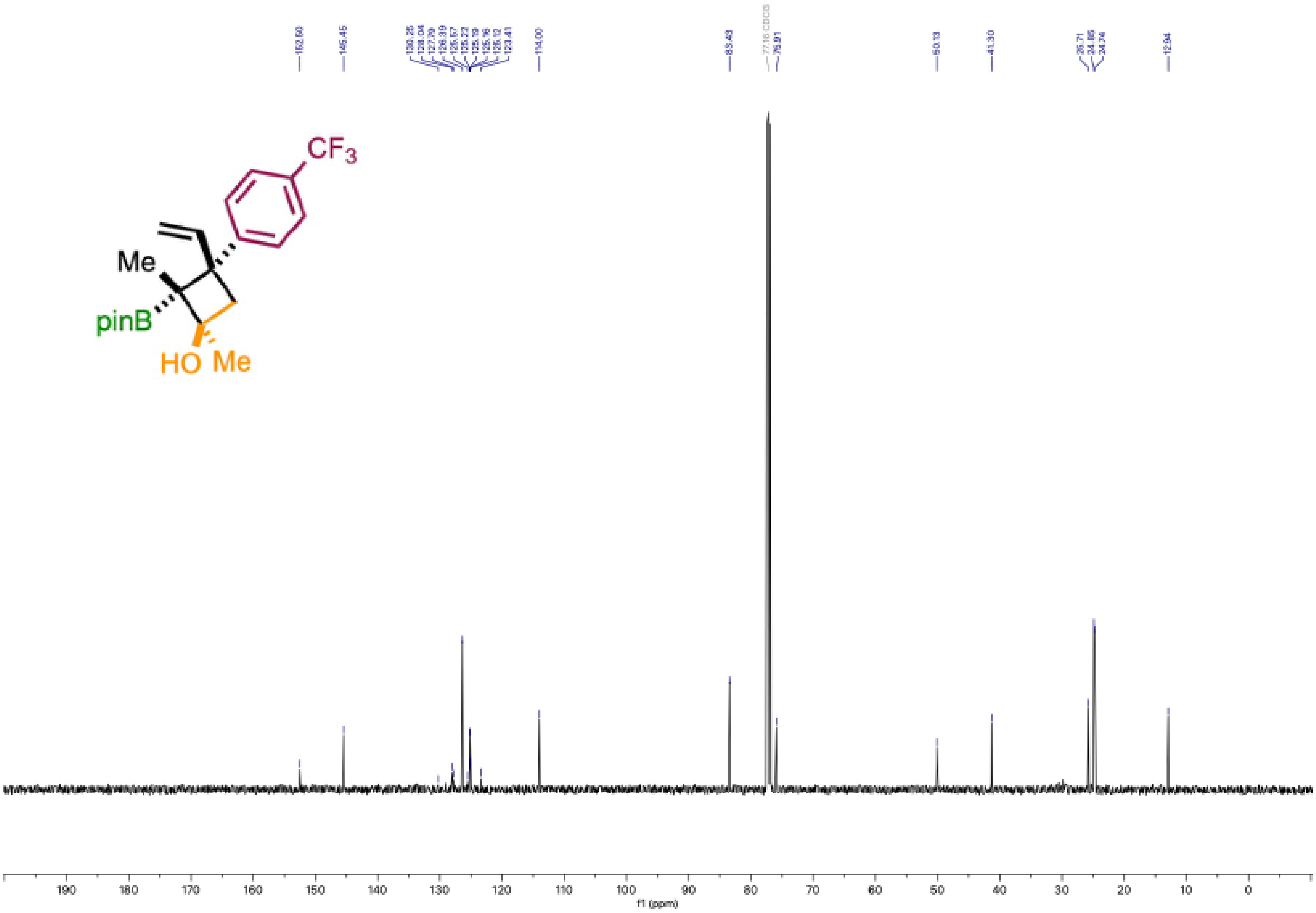

^{13}C NMR spectrum (101 MHz, $CDCl_3$)

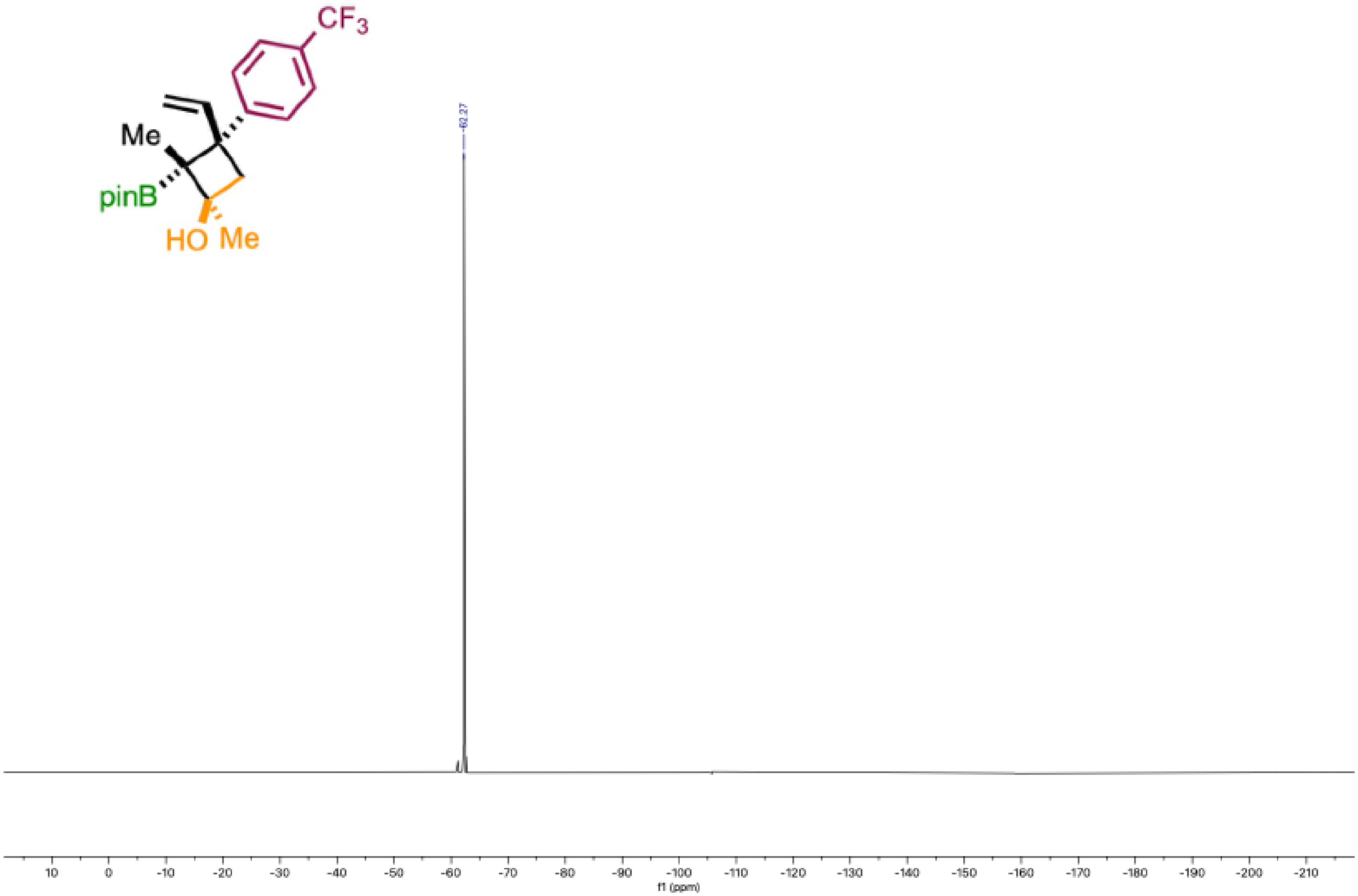

^{19}F NMR spectrum (377 MHz, $CDCl_3$)

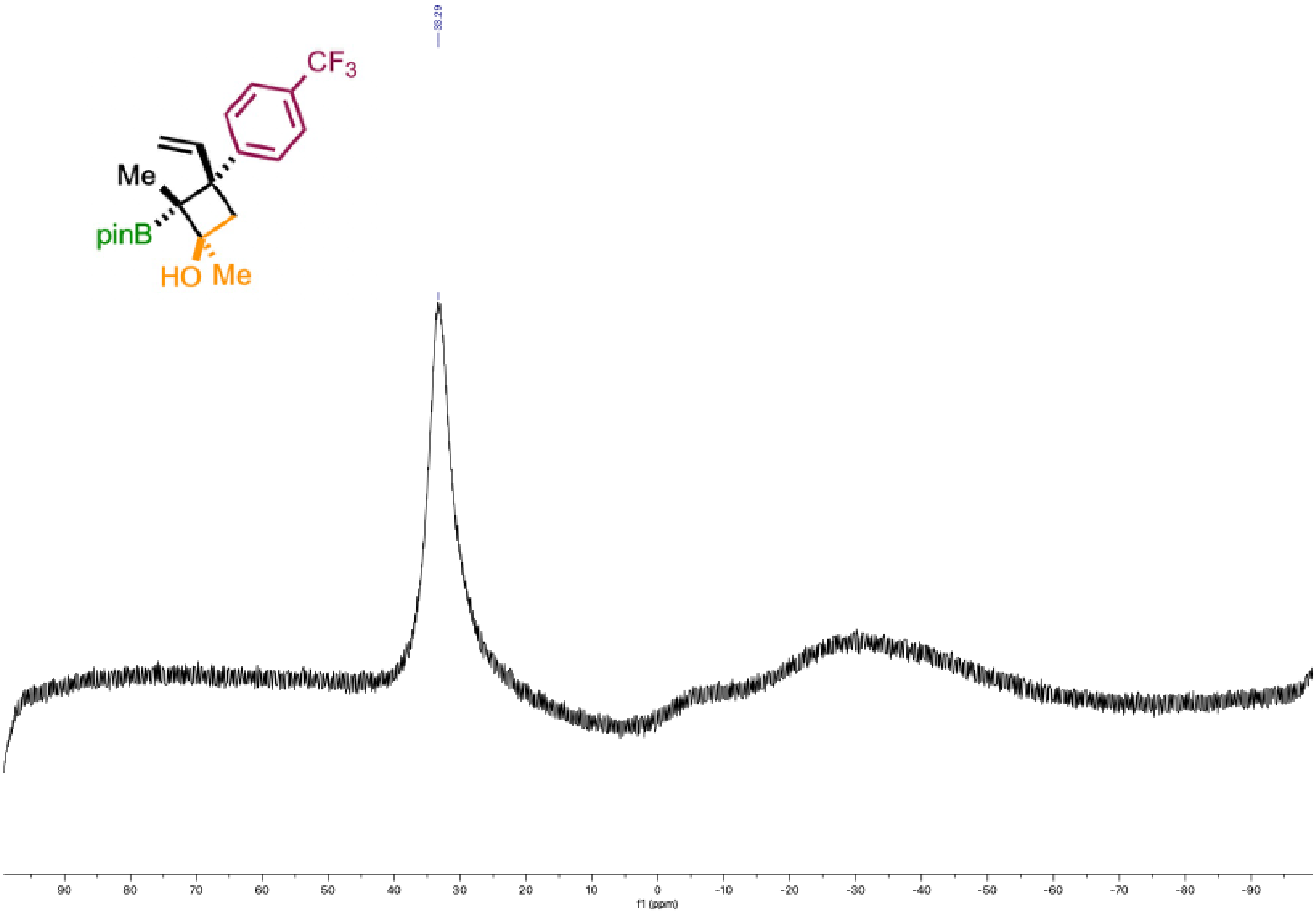

^{11}B NMR spectrum (128 MHz, $CDCl_3$)

6. References

(1) (a) Cohen, Y.; Augustin, A. U.; Levy, L.; Jones, P. G.; Werz, D. B.; Marek, I. Regio- and Diastereoselective Copper-Catalyzed Carbomagnesiation for the Synthesis of Penta- and Hexa-Substituted Cyclopropanes. *Angew. Chem. Int. Ed.* **2021**, *60* , 11804-11808. (b) Thigpen, A. B., Jr.; Fuchs, R. Synthesis and ionization constants of meta- and para-substituted 1-phenyl-2-methylcyclopropene-3-carboxylic acids. *J. Org. Chem.* **1969**, *34*, 505-509. (c) Sercel, Z. P.; Marek, I. General palladium-catalyzed cross coupling of cyclopropenyl esters. *Chem. Sci.* **2025**, *16*, 12162-12167.

(2) Fordyce, E. A. F.; Luebbers, T.; Lam, H. W. Synthesis and Application of Alkenylstannanes Derived from Base-Sensitive Cyclopropenes. *Org. Lett.* **2008**, *10*, 3993-3996.

(3) (a) Augustin, A. U.; Di Silvio, S.; Marek, I. Borylated Cyclopropanes as Spring-Loaded Entities: Access to Vicinal Tertiary and Quaternary Carbon Stereocenters in Acyclic Systems. *J. Am. Chem. Soc.* **2022**, *144*, 16298-16302. (b) Pavlíčková, T.; Stöckl, Y.; Marek, I. Synthesis and Functionalization of Tertiary Propargylic Boronic Esters by Alkynyllithium-Mediated 1,2-Metalate Rearrangement of Borylated Cyclopropanes. *Org. Lett.* **2022**, *24*, 8901-8906.

(4) Burchat, A. F.; Chong, J. M.; Nielsen, N. Titration of alkyllithiums with a simple reagent to a blue endpoint. *J. Organomet. Chem.* **1997**, *542*, 281-283.

(5) Dolomanov, O. V.; Bourhis, L.J.; Gildea, R. J.; Howard, J. A. K.; Puschmann, H. OLEX2: a complete structure solution, refinement and analysis program *J. Appl. Cryst.* **2009**, *42*, 339-341.

(6) Bourhis, L. J.; Dolomanov, O. V.; Gildea, R. J;, Howard, J. A. K.; Puschmann, H. The anatomy of a comprehensive constrained, restrained refinement program for the modern computing environment - Olex2 dissected. *Acta Cryst.* **2015**, *A71*, 59-75.

(7) Sheldrick, G.M. Crystal structure refinement with SHELXL. *Acta Cryst.* **2015**, *C71*, 3-8.

(8) Mercury Software from CCDC: http://www.ccdc.cam.ac.uk/Solutions/CSDSystem/Pages/Mercury.aspx.

(9) Neese, F. The ORCA program system. *Wiley Interdiscip. Rev.: Comput. Mol. Sci.* **2012**, *2*, 73–78.

(10) Becke, A. D. Density-functional exchange-energy approximation with correct asymptotic behavior. *Phys. Rev. A.* **1988**, *38*, 3098-3100.

(11) Perdew, J. P. Density-functional approximation for the correlation energy of the inhomogeneous electron gas. *Phys. Rev. B Condens*. **1986**, *34*, 7406-7406.

(12) Perdew, J. P. Density-functional approximation for the correlation energy of the inhomogeneous electron gas. *Physical review B*. **1986**, *33*, 8822-8824.

(13) Grimme, S. Accurate description of van der Waals complexes by density functional theory including empirical corrections. *J. Comput. Chem*. **2004**, *25*, 1463-1473.

(14) Weigend, F.; Ahlrichs, R. Balanced basis sets of split valence, triple zeta valence and quadruple zeta valence quality for H to Rn: Design and assessment of accuracy. *Phys. Chem. Chem. Phys*. **2005**, *7*, 3297-3305.

(15) Zhao, Y.; Truhlar, D. G. The M06 suite of density functionals for main group thermochemistry, thermochemical kinetics, noncovalent interactions, excited states, and transition elements: two new functionals and systematic testing of four M06-class functionals and 12 other functionals. *Theor. Chem. Acc.* **2008**, *120*, 215-241.

(16) Izsák, R.; Neese, F. An overlap fitted chain of spheres exchange method. *J. Chem. Phys.* **2011**,*135,* 144105.

(17) Bryantsev, V. S.; Diallo, M. S.; Goddard Iii, W. A. Calculation of solvation free energies of charged solutes using mixed cluster/continuum models. *J. Phys. Chem. B*. **2008**, *112*, 9709-9719.

(18) Cohen, A.; Kaushansky, A.; Marek, I. Mechanistic insights on the selectivity of the tandem Heck–ring-opening of cyclopropyldiol derivatives. *JACS Au*. **2022,** *2*, 687-696.

(19) Pracht, P.; Bohle, F.; Grimme, S. Automated exploration of the low-energy chemical space with fast quantum chemical methods. *Phys. Chem. Chem. Phys.* **2020**, *22*, 7169-7192.
